# Organocatalytic atroposelective construction of axially chiral arylquinones

**DOI:** 10.1038/s41467-019-12269-4

**Published:** 2019-09-19

**Authors:** Shuai Zhu, Ye-Hui Chen, Yong-Bin Wang, Peiyuan Yu, Shao-Yu Li, Shao-Hua Xiang, Jun-Qi Wang, Jian Xiao, Bin Tan

**Affiliations:** 1 Shenzhen Grubbs Institute, Department of Chemistry, Southern University of Science and Technology, Shenzhen, 518055 China; 2 Academy for Advanced Interdisciplinary Studies, Southern University of Science and Technology, Shenzhen, 518055 China; 3 Department of Biology, Southern University of Science and Technology, Shenzhen, 518055 China; 4 College of Chemistry and Pharmaceutical Sciences, Qingdao Agricultural University, Qingdao, 266109 China

**Keywords:** Asymmetric catalysis, Organocatalysis, Synthetic chemistry methodology

## Abstract

Atropisomeric biaryl motifs are ubiquitous in chiral catalysts and ligands. Numerous efficient strategies have been developed for the synthesis of axially chiral biaryls. In contrast, the asymmetric construction of *o*-quinone-aryl atropisomers has yet to be realized. Inspired by the rapid progress of the chemistry of biaryls, here we present our initial investigations about the atroposelective construction of axially chiral arylquinones by a bifunctional chiral phosphoric acid-catalyzed asymmetric conjugate addition and central-to-axial chirality conversion. With o-naphthoquinone as both the electrophile and the oxidant, three types of arylation counterparts, namely 2-naphthylamines, 2-naphthols and indoles, are utilized to assemble a series of atropisomeric scaffolds in good yields and excellent enantioselectivities. This approach not only expands the axially chiral library but also offers a route to a class of potential, chiral biomimetic catalysts.

## Abstract (web summary)

Axially chiral compounds have widespread use in asymmetric catalysis. Here, the authors disclose a highly enantioselective synthesis of axially chiral *o*-naphthoquinones by chiral phosphoric acid catalysis and central-to-axial chirality conversion.

## Introduction

**Fig. 1:**
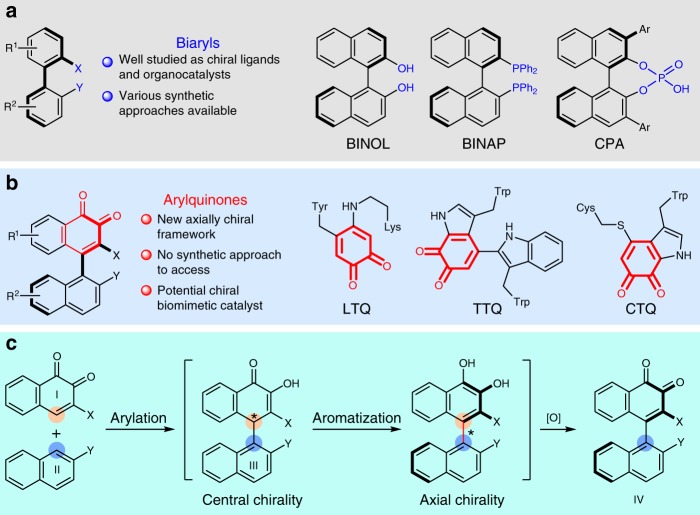
Axially chiral biaryls vs. axially chiral arylquinones and their synthesis. **a** Biaryls as chiral ligands and organocatalysts. **b** *o*-Quinone structures in biomimetic catalysts. **c** Our designed approach to access arylquinone atropisomer

Enantioenriched axially chiral biaryls are of great significance due to the ubiquitous relevance in natural products^1,2^, bioactive molecules^3,4^ and functional materials^5,6^. They also constitute the core structural motif of various chiral catalysts and ligands for asymmetric transformations^7,8^. Particularly, the emergence of BINOL^9,10^ and its derivatives^11–13^ brought about remarkable improvement of asymmetric catalysis domain and altered the scenario that the acquirement of highly enantioenriched products from prochiral substrates via routinely chemical approaches remained extremely challenging half a century ago. The past couple of decades have witnessed the rapid progress of the biaryls based chemistry and a series of efficient synthetic strategies have been developed for the construction of chiral biaryls (Fig. 1a)^14–16^. In stark contrast, despite only slight alteration in molecular structure, the *o*-quinone-aryl atropisomer has been still unrevealed and its asymmetric synthesis has never been materialized till now, to the best of our knowledge. On the other hand, the *o*-quinones occur prevalently in bioactive natural products^17,18^ and are widely recognized as cofactors in numerous quinoproteins (Fig. 1b)^19–22^. Mechanistic studies reveal the role of quinone cofactors as the actual active centers while metals necessitate the regeneration of the quinone cofactors in these transformations. In addition, they were found to be useful synthetic intermediates for many valuable transformations^23,24^. In this regard, the enantioselective construction of hitherto unknown atropisomeric aryl *o*-quinone not only brings about a class of axially chiral atropisomers but also may provide a class of potential chiral biomimetic catalysts.

On the basis of the recent advance in the construction of axially chiral backbones^25–34^ and our continuous understanding of this field^35^, we envisaged that the atroposelective synthesis of the axially chiral *o*-quinone is conceivable via arylation of *o*-naphthoquinone promoted by organocatalyst. First, enantioselective arylation of *o*-naphthoquinone I with aryl nucleophile II provides central chiral intermediate III in the presence of chiral organocatalyst. Subsequent aromatization and oxidation result in the transfer of central to axial chirality, furnishing the excepted axially chiral aryl *o*-naphthoquinone IV. Ideally, the oxidative capability of excess *o*-naphthoquinone I will facilitate the oxidation reaction (Fig. 1c). However, related research becomes more appealing and challenging when the planar aromatic ring was replaced by an *o*-quinone as compared to biaryls and then several challenges need to be taken into consideration: (1) appropriate substituents should be equipped on *o*-naphthoquinone and/or nucleophile to enhance the reactivity and restrict the axial rotation; (2) an appropriate bifunctional organocatalyst is essential for simultaneous activation of nucleophile and electrophile in appropriate chiral environment; strong background reaction is detrimental to the stereocontrol; (3) entire oxidation process should proceed under mild conditions to circumvent the racemization of the axial chirality.

Herein we disclose the discovery of axially chiral *o*-naphthoquinones and their enantioselective construction in the presence of bifunctional chiral phosphoric acid catalyst via central-to-axial chirality conversion. A range of atropisomeric *o*-naphthoquinone frameworks are synthesized in good yields and excellent enantiocontrol with *o*-naphthoquinone as the electrophile and oxidant, 2-naphthylamine, 2-naphthol or indole as the arylation reagent.

## Results

### Optimization of the reaction conditions

Considering the aforementioned factors, we selected *o*-naphthoquinone **1a–1** with an ester group as the model electrophile and 2-naphthylamine **2a–1** as the aryl donor to construct axially chiral backbone under the activation of chiral phosphoric acid^36,37^. The introduction of ester group was thought to enhance the interaction between quinone and catalyst while strengthens the stability of chiral axis of the product in line with the reported results^38^. Also, we expected the excessive *o*-naphthoquinone to serve as oxidant to de-aromatize naphthol intermediate. To verify the feasibility of our design, 10 mol% of chiral phosphoric acid (**CPA1**) was utilized to promote the reaction of **1a–1** (2.2 eq.) and **2a–1** (1.0 eq.) in CH_2_Cl_2_ at room temperature. Fortunately, the desired product **3a–1** was afforded in 4 h in 32% isolated yield (Table 1, entry 1). Notably, this compound displayed axial chirality based on the chiral stationary high-performance liquid chromatography (HPLC) analysis. Despite only inapparent enantiomeric excess (4% ee), our proposed hypothesis was substantiated by direct experimental evidence and the enantioenriched atropisomeric *o*-naphthoquinone was realized. Following optimization focused on the evaluation of various **CPA** and BINOL-derived **CPA4** with a bulky 2,4,6-triisopropylphenyl substituent on the 3,3′-position was found to be the best catalyst, providing **3a–1** in excellent yield and enantioselectivity (87% yield, 99% ee) in only 20 min (Table 1, entries 2–11). Other solvents were surveyed to further improve the reaction but in futile (Table 1, entries 12–16). Further investigations revealed that 1 mol% catalyst loading was optimal to uphold the high yield, as well as enantiocontrol of the current reaction (Table 1, entry 18). The optimized conditions were then concluded as follows: **1a–1** (0.22 mol), **2a–1** (0.10 mol) and **CPA4** (1 mol%) in 2 mL of CH_2_Cl_2_ and performed at room temperature for 20 min.

**Table 1:** Reaction optimization with 2-naphthylamine^*a*^


entry	CPA	solvent	time (min)	yield (%)^*b*^	ee (%)^*c*^
1	(*R*)-**CPA1**	CH_2_Cl_2_	240	32	–4
2	(*R*)-**CPA2**	CH_2_Cl_2_	240	38	–36
3	(*R*)-**CPA3**	CH_2_Cl_2_	240	27	–51
4	(*S*)-**CPA4**	CH_2_Cl_2_	20	87	99
5	(*R*)-**CPA5**	CH_2_Cl_2_	20	85	99
6	(*R*)-**CPA6**	CH_2_Cl_2_	240	36	–83
7	(*R*)-**CPA7**	CH_2_Cl_2_	240	57	–47
8	(*R*)-**CPA8**	CH_2_Cl_2_	240	52	–30
9	(*R*)-**CPA9**	CH_2_Cl_2_	240	24	–28
10	(*R*)-**CPA10**	CH_2_Cl_2_	240	35	–14
11	(*R*)-**CPA11**	CH_2_Cl_2_	240	38	–42
12	(*S*)-**CPA4**	toluene	20	60	99
13	(*S*)-**CPA4**	CHCl_3_	20	82	99
14	(*S*)-**CPA4**	EtOAc	20	43	68
15	(*S*)-**CPA4**	DCE	20	86	99
16	(*S*)-**CPA4**	ether	20	62	83
17^*d*^	(*S*)-**CPA4**	CH_2_Cl_2_	20	86	99
18^*e*^	(*S*)-**CPA4**	CH_2_Cl_2_	20	86	99

^*a*^Reaction conditions: **1a–1** (0.22 mmol), **2a–1** (0.10 mmol), solvent (2 mL) under Ar atmosphere, unless noted otherwise. ^*b*^Isolated yields. ^*c*^Determined by HPLC analysis on a chiral stationary phase. ^*d*^Conducted with 5 mol% of **CPA4**. ^*e*^Conducted with 1 mol% of **CPA4**

### Substrate scope

With the optimized conditions in hand, we next set out to explore the substrate scope of this reaction by examining derivatives of 2-naphthylamine and *o*-naphthoquinone. Overall, all the tested substrates gave the expected axially chiral arylquinones in good yields with excellent atroposelectivities (Table 2). Firstly, replacement of methyl group on ester by an ethyl or benzyl group provided the adduct **3a–2** or **3a–3** in lower yield and enantioselectivity. The introduction of a substituent on the aromatic ring of *o*-naphthoquinones (**3a–4**~**3a–7**) led to slight yield reduction. A wide range of 2-naphthylamines with different substituents or substitution patterns were evaluated under the standard conditions. In most cases, the desired axially chiral arylquinones (**3a–8**~**3a–14**) could be furnished with up to 99% ee. A certain degree of variation in chemical yield was detected for each reaction. Notably, no desired product was obtained under standard conditions when *N*,*N*-dimethyl-2-naphthylamine was used, indicating the importance of N–H bond for this reaction.

**Table 2:**
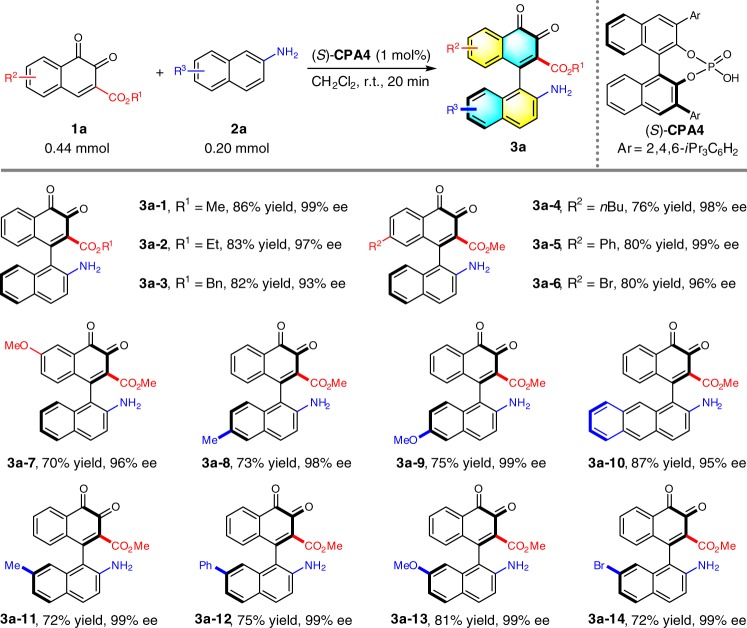
Substrate scope with 2-naphthylamine^*a*^

^*a*^Reaction conditions: **1a** (0.44 mmol), **2a** (0.20 mmol), (*S*)-**CPA4** (1 mol%), CH_2_Cl_2_ (4 mL), room temperature for 20 min under Ar atmosphere. Isolated yields were provided and ee values were determined by HPLC analysis on a chiral stationary phase

To further expand the generality of this strategy, we applied the optimized conditions to the reaction of *o*-naphthoquinone **1a–1** and phenyl protected 2-naphthylamine **2b–1**. Disappointedly, less than 10% of conversion was detected after 10 h under this set of conditions. While previous studies suggested the indicative effects of **CPA** catalyst on reaction outcomes, the re-optimization was initiated by screening of various **CPA**. Our optimization efforts revealed that H_8_**–**BINOL derived **CPA6** could provide the most optimal results at room temperature for 10 h, affording the product **3b–1** in 93% yield and 99% ee. Further studies showed CH_2_Cl_2_ remained as the most efficient reaction solvent. While 10 mol% catalyst loading could not bring about any improvement for the reaction results, 1 mol% catalyst loading resulted in chemical yield decrement of 3 mol% (Supplementary Table 2). The re-optimized conditions were then applied to a wide spectrum of *o*-naphthoquinones and phenyl protected 2-naphthylamines (Table 3). The influence of the ester groups on the *o*-naphthoquinone was first investigated. Slightly compromised reaction yield and selectivity were observed when methyl group was replaced with bulkier ethyl (**3b–2**), benzyl (**3b–3**) or isopropyl group (**3b–4**). Nonetheless, additional substituent introduced on the aromatic ring of *o*-naphthoquinone exerted limited effects to the reaction outcomes (**3b–5**~**3b–8**). Halide functionality (**3b–9** to **3b–11**), electron-donating (**3b–12** and **3b–13**) and electron-withdrawing groups (**3b–14**) on phenyl protecting group were all well tolerated to give the corresponding products in satisfactory results. To verify the utility of the current organocatalytic atroposelective reaction, a gram-scale synthesis of **3b–7** was performed under the standard reaction conditions. As presented in Table 3, the desired axially chiral product was furnished with the same enantiocontrol but in higher yield, inferring this protocol is suitable for the large-scale production.

**Table 3:**
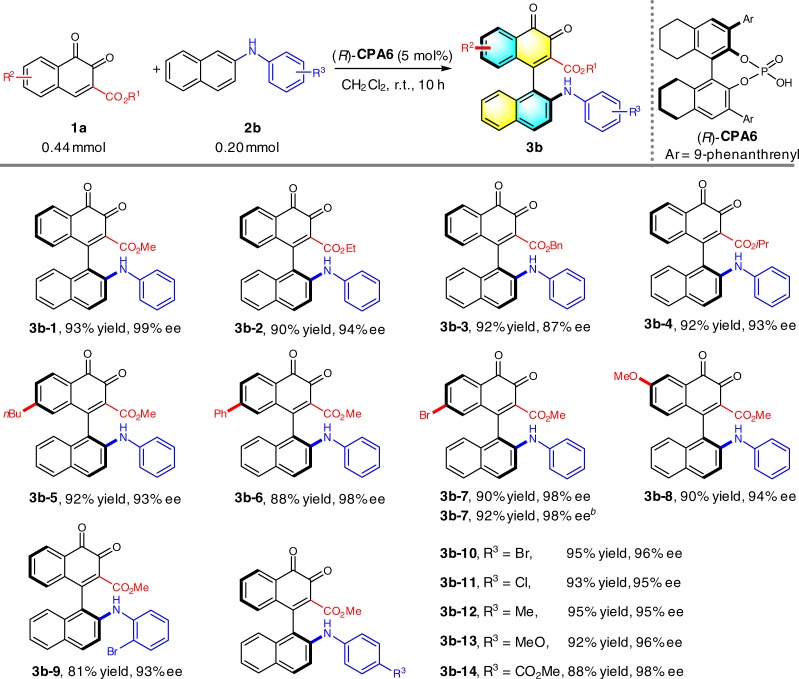
Substrate scope with *N*-arylnaphthalen-2-amine^*a*^

^*a*^Reaction conditions: **1a** (0.44 mmol), **2b** (0.20 mmol), (*R*)-**CPA6** (5 mol%), CH_2_Cl_2_ (4 mL), room temperature for 10 h under Ar atmosphere. Isolated yields were provided and ee values were determined by HPLC analysis on a chiral stationary phase. ^*b*^Gram-scale reaction: **1a–6** (4.4 mmol), **2b–1** (2.0 mmol)

In our original design, the ester group was proposed to enhance the interaction between **CPA** and substrate as well as the stability of the chiral axis. However, the C-3 substituent of *o*-naphthoquinones has been demonstrated to reduce the catalytic capability significantly^39^. Encouraged by our initial results, we then attempted to explore the practicability for the synthesis of axially chiral arylquinones without an ester group. The more challenging and rewarding project commenced with *o*-naphthoquinone **1b–1** as the model electrophile. Compound **2b–1** was anticipated to be an appropriate nucleophile since the bulky protecting group might be conducive to restrict the axial rotation. The reaction was conducted in CH_2_Cl_2_ with 10 mol% **CPA4** as the catalyst. Gratifyingly, the desired product **3c–1** was provided in 92% yield and 83% ee. No improved result arose by screening a variety of **CPA**s including **CPA6** (78% yield, 46% ee), the optimal catalyst for the reaction of phenyl protected 2-naphthylamines and *o*-naphthoquinones with an ester group. Not surprisingly, CH_2_Cl_2_ proved to be the most efficient solvent. The enantioselectivity was augmented to 93% when the reaction was performed at –30 °C, albeit lower yield (63%). The reduction of the catalyst loading led to obvious yield and enantioselectivity depletion (Supplementary Table 3). The optimal reaction conditions were then applied to other four substrates. As depicted in Table 4, all these reactions could deliver the desired products (**3c–2** to **3c–5**) in excellent stereocontrol at the expense of lower yields. Although limited substrate scope was established at this stage, the successful construction of this axially chiral backbone offered more possibility to realize the application in catalysis.

**Table 4:**
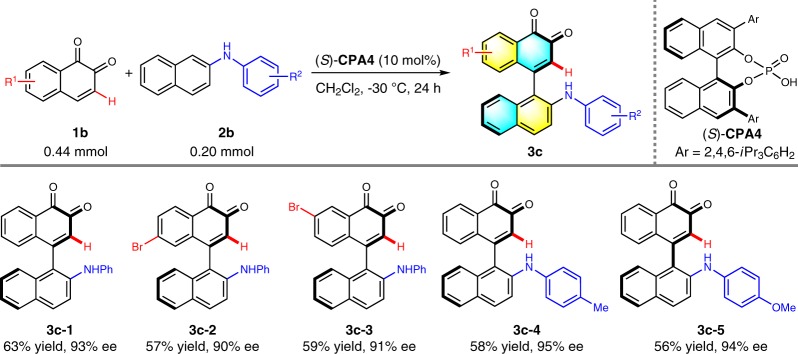
Substrate scope with *o*-naphthoquinone without ester group^*a*^

^*a*^Reaction conditions: **1b** (0.44 mmol), **2b** (0.20 mmol), (*S*)-**CPA4** (10 mol%), CH_2_Cl_2_ (4 mL), –30 °C for 24 h under Ar atmosphere. Isolated yields were provided and ee values were determined by HPLC analysis on a chiral stationary phase

To further broaden the scope of axially chiral arylquinone family, we moved to seek other applicable nucleophiles upon accomplishing the investigation with 2-naphthylamine and its derivatives. 2-Naphthol, which has proven impressive ability to act as aryl nucleophiles for construction of BINOL derivatives^40,41^, then came to our mind. Employing 2-naphthol **2c** as the aryl donor, we again envisaged the prohibitory role of an ester moiety on *o*-naphthoquinone for hindering the rotation of chiral axis. Careful screening of the conditions presented the following optimal system: H_8_–BINOL-derived **CPA7** as the catalyst, BF_4_Li as the additive, and the reaction was conducted in CH_2_Cl_2_ at −10 °C for 24 h to give the desired product **3d–1** in 90% yield and 92% ee (Supplementary Table 5). This protocol has shown excellent applicability and compatibility to several analogs (Table 5). All products (**3d–2**~**3d–7**) were obtained in excellent yields and enantioselectivities respectively.

**Table 5:**
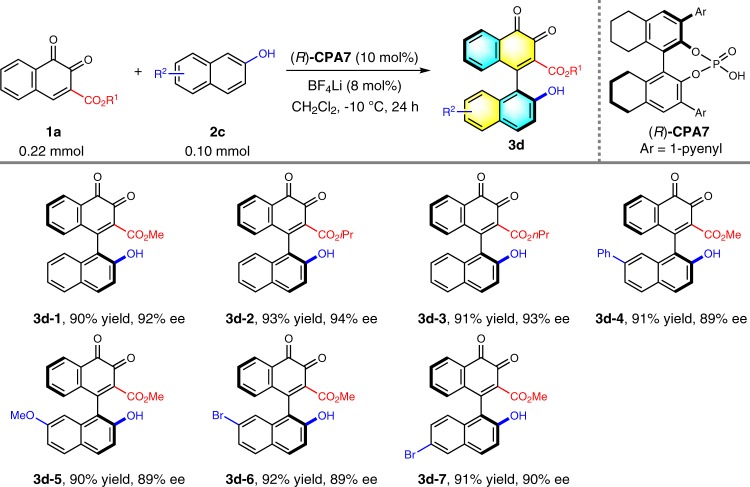
Substrate scope with 2-naphthol^*a*^

^*a*^Reaction conditions: **1a** (0.22 mmol), **2c** (0.10 mmol), (*R*)-**CPA7** (10 mol%), CH_2_Cl_2_ (4 mL), BF_4_Li (8 mol%), –10 °C for 24 h under Ar atmosphere. Isolated yields were provided and ee values were determined by HPLC analysis on a chiral stationary phase

Besides, indole has been validated to be a competent nucleophile^42,43^ under acidic conditions for the synthesis of indolylbenzoquinone^44,45^, which is widespread in various natural products with remarkable bioactivity. However, to the best of our knowledge, there is no facile asymmetric construction of axially chiral indolyl-*o*-naphthoquinone to-date. To test the relevance of indole nucleophile in our protocol, **CPA4** (10 mol%) was utilized to promote the reaction of **1a–1** and **2d–1** in CH_2_Cl_2_ for 24 h under Ar atmosphere which led to the corresponding product in 44% ee. Extensive efforts dedicated in a massive parallel screening of conditions were unfruitful with only moderate enantiocontrol (76% ee) attained. Interestingly, the bromide-containing substrate **1c–1** provided the product **3e–1** efficiently with 89% ee with the optimal catalyst **CPA4** in CH_2_Cl_2_. Subsequent optimization further affirmed the superior reaction competency of CH_2_Cl_2_ compared to other solvents in the presence of **CPA4**. The reaction selectivity was dictated by catalyst loading in which lower catalyst loading gave lower reaction efficiency in return. Notably, the enantioselectivity was improved to 96% ee when the reaction was performed at 0 °C without diminution of the yield (95%). On the contrary, alteration of other factors such as reaction time and molarity proved futile. The optimized conditions were then concluded as follows: **1c–1** 0.20 mol, **2d–1** 0.10 mol, 10 mol% **CPA4** in CH_2_Cl_2_ and the reaction was performed at 0 °C for 24 h under air atmosphere (Supplementary Table 6).

With the optimal conditions in hand, we next explored the substrate scope and the limitation of this reaction. Firstly, the effect of the substituent on the indole ring was tested. As summarized in Table 6, indole nucleophile with either an electron donating group (**3e–2**, **3e–3**) or a halide group (**3e–4**, **3e–5**) could be utilized to give the desired product in excellent yields and enantioselectivities. Likewise, both chlorine (**3e–6**) and iodine group (**3e–7**) substituted *o*-naphthoquinone electrophiles were very well tolerated under the conditions to give the axially chiral products with similar outcome and high chemical stability apart from the bromine counterpart. The isopentyl group could also act as the axial rotation resistance group to furnish product **3e–8** in consistently excellent enantioselectivity. Noteworthy, the stereoselectivity was completely lost (0% ee) when 2-*tert*-butyl-1-methylindole **2d–7** was used as the nucleophile for this reaction, indicating that the N–H bond of indole is crucial on the stereocontrol.

**Table 6:**
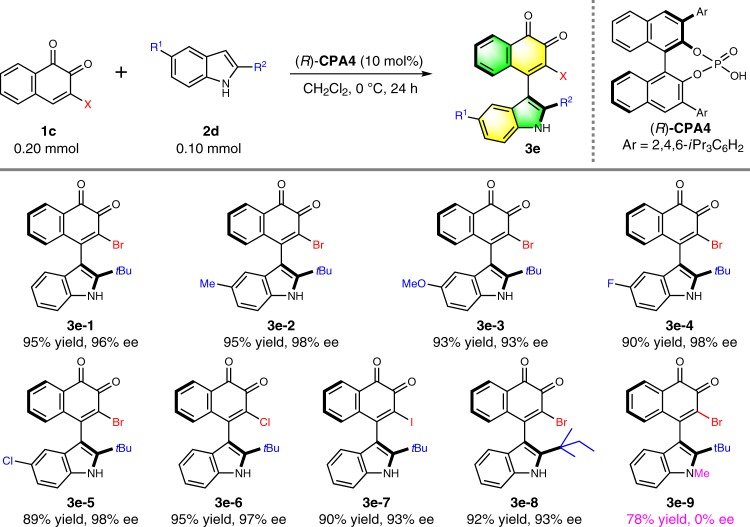
Substrate Scope with Indole^*a*^

^*a*^Reaction conditions: **1c** (0.20 mmol), **2d** (0.10 mmol), (*R*)-**CPA4** (10 mol%) in CH_2_Cl_2_ (1 mL) at 0 °C for 24 h under Ar atmosphere. Isolated yields were provided and ee values were determined by HPLC analysis on a chiral stationary phase

### Transformations and asymmetric catalysis applications

**Fig. 2:**
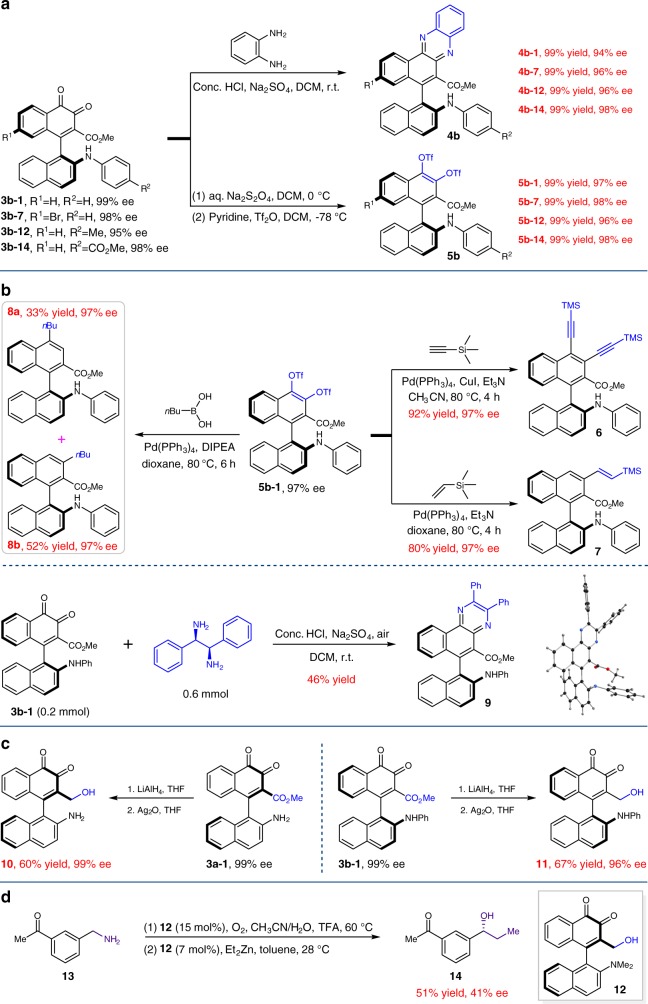
Synthetic transformations and catalytic applications. **a** Synthetic transformations of **3b**. **b** Cross-coupling reactions with **5b–1**, the synthesis of compound **9** and its X-ray structure. **c** The reduction of the ester group on **3a–1** and **3b–1**. **d** Applications in asymmetric catalysis

To demonstrate the potential of the developed approach, synthetic transformations were then performed based on the generated products **3b**. Phenazine scaffold constitutes the core structures of various natural products with attractive bioactivities^46–48^. Moreover, it could sever as essential backbone for synthetic dyes. Accordingly, the synthesis of phenazine containing molecules to enrich the diversity of the library is of significant importance. Gratifyingly, the expected phenazine could be readily attained from *o*-quinone. The reactions of representative **3b** with benzene-1,2-diamine in the presence of concentrated HCl and sodium sulfate proceeded smoothly to yield the corresponding products **4b** in quantitative yields with almost identical enantioselectivities (Fig. 2a). Subsequently, sodium dithionite was utilized to reduce the *o*-quinone moiety. The generated *o*-dihydroxybenzene intermediates were then trapped by Tf_2_O to give the more stable structures **5b** in quantitative yields. Negligible deteriorations of stereochemical integrity were detected for all these reactions (Fig. 2a). Notably, the resultant phenyl trifluoromethanesulfonate provides opportunities for downstream coupling reactions, allowing quick access to a variety of structurally diversified atropisomeric binaphthyls. Interestingly, utilizing **5b–1** as the starting material, the Sonogashira coupling reaction proceeded smoothly to give bis-substituted product **6** with ethynyltrimethylsilane in 92% yield, while axially chiral molecule **7** with one olefinic group was produced efficiently by the Heck reaction. Apart from that, the Suzuki coupling reaction provided a mixture of **8a** and **8b** with *n-*butylboronic acid as the coupling partner. Likewise, no stereochemical integrity loss was observed for all these reactions (Fig. 2b). Next, the absolute configuration of **3b–1** was deduced to be (*S*) by X-ray crystallographic analysis of its derivative **9**, which could be prepared by the treatment of **3b–1** with (1 *R*,2 *R*)-1,2-diphenylethane-1,2-diamine under the identical conditions for the synthesis of compounds **4** (CCDC: 1833698), and those of other products (**3a–3e**) were assigned by analogy in accordance with the corresponding absolute configuration of **CPA**s. Subsequently, the ester group could be readily converted to alcohol with lithium aluminum hydride as the reductive agent, affording arylquinone **10** or **11** with acceptable yield, respectively (Fig. 2c). Finally, the synthetic and catalytic applications of the obtained axially chiral *o*-naphthoquinones were investigated. As shown in Fig. 2d, compound **12**, prepared from compound **10** by reductive amination, acted as an oxidant for the transformation of benzylamine **13** to corresponding aldehyde. Subsequently, it severed as a chiral ligand in the presence of reducing agent for the asymmetric addition of ZnEt_2_ to the generated aldehyde, give the desired chiral alcohol **14** in moderate yield for two steps, albeit low enantioselectivity.

### Plausible mechanism

**Fig. 3:**
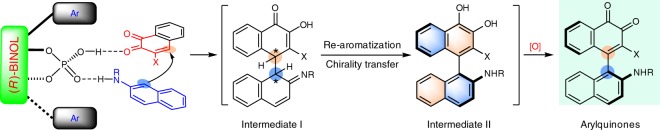
Proposed reaction pathway. The reaction sequence follows chiral phosphoric acid catalyzed asymmetric conjugate addition, re-aromatization with central-to-axial chirality conversion and oxidation

Based on the experimental results and the elegant work of Rodriguez and Bonne involving central-to-axial chirality conversion strategy^49,50^, a plausible reaction pathway was proposed in Fig. 3. The first step of this reaction is the chiral phosphoric acid catalyzed asymmetric conjugate addition to form intermediate I with two chiral centers. The following re-aromatization with central-to-axial chirality conversion gives atropisomeric intermediate II. Finally, oxidation by the use of excessive *o*-quinone furnished the excepted axially chiral arylquinone. Chiral phosphoric acid performed as a bifunctional organocatalyst to simultaneously activate 2-naphthylamine/2-naphthol/indole and *o*-naphthoquinone by multiple H-bonding and promote the first step of enantioselective nucleophilic addition to produce intermediate I. The rapid re-aromatization of this intermediate leads to the axially chiral intermediate II and eventually to the final product.

## Discussion

In summary, we have discovered the axial chirality of *o*-naphthoquinones and provided the enantioselective conjugate addition strategy for the construction of axially chiral arylquinones with chiral phosphoric acid as the catalyst via sequential conjugated addition, chirality transfer and oxidation. With *o*-naphthoquinone as the electrophile and oxidant, three types of arylation nucleophiles, 2-naphthylamines, 2-naphthols, as well as indoles, were efficiently employed to outline the spectrum for axially chiral skeletons in good yields and excellent enantioselectivities. The resulted products could be converted to phenazine containing molecules and other useful axial chiral binaphthyls by downstream transformations. The arylquinone is not only an efficient reagent for the oxidation of amine, but also an applicable chiral ligand in asymmetric catalysis. Further investigations are underway to exploit the catalytic and synthetic applications in our laboratory.

## Methods

### General procedure for asymmetric synthesis (*R*)-**3a**

To a mixture of (*S*)-**CPA4** (1 mol%), *o*-naphthoquinone **1a** (0.44 mmol) and nucleophile **2a** (0.20 mmol) in 10 mL Schlenk tube was added CH_2_Cl_2_ (4.0 mL) under Ar atmosphere. Then the reaction mixture was stirred vigorously at room temperature until **2a** was completely consumed. Typical reaction time was about 20 min. The resulting mixture was concentrated under reduced pressure and purified by flash chromatography on silica gel (eluent: PE/EA = 4/1) to afford the corresponding axially chiral arylquinones (*R*)**−3a**.

### General procedure for asymmetric synthesis of (*S*)-**3b**

To a mixture of (*R*)-**CPA6** (5 mol%), *o*-naphthoquinone **1a** (0.44 mmol) and nucleophile **2b** (0.20 mmol) in 10 mL Schlenk tube was added CH_2_Cl_2_ (4.0 mL) under Ar atmosphere. Then the reaction mixture was stirred vigorously at room temperature until **2b** was completely consumed. Typical reaction time was about 10 h. The resulting mixture was concentrated under reduced pressure and purified by flash chromatography on silica gel (eluent: PE/EA = 6/1) to yield the corresponding axially chiral arylquinones (*S*)-**3b**.

### General procedure for asymmetric synthesis of (*R*)-**3c**

To a mixture of (*S*)-**CPA4** (10 mol%) and nucleophile **2b** (0.20 mmol) in 10 mL Schlenk tube was added CH_2_Cl_2_ (4.0 mL) under Ar atmosphere. The reaction mixture was stirred for 15 min at −30 °C before *o*-naphthoquinone **1b** (0.44 mmol) was added under Ar atmosphere. Then the reaction was stirred vigorously at −30 °C until **2b** was completely consumed. Typical reaction time was about 24 h. The resulting mixture was concentrated under reduced pressure and purified by flash chromatography on silica gel (eluent: PE/EA = 6/1) to give rise to the corresponding axially chiral arylquinones (*R*)-**3c**.

### General procedure for asymmetric synthesis of (*S*)-**3d**

To a mixture of (*R*)-**CPA7** (10 mol%) and BF_4_Li (8 mol%) in 10 mL Schlenk tube was added CH_2_Cl_2_ (4.0 mL) under Ar atmosphere. The reaction mixture was stirred for 1 h at room temperature. After that, *o*-naphthoquinone **1a** (0.22 mmol) and **2c** (0.10 mmol) were added and the mixture was stirred vigorously at −10 °C until **2c** was completely consumed. Typical reaction time was about 24 h. The resulting mixture was concentrated under reduced pressure and purified by flash chromatography on silica gel (eluent: PE/EA = 6/1) to produce the corresponding axially chiral arylquinones (*S*)-**3d**.

### General procedure for asymmetric synthesis of (*S*)-**3e**

To a mixture of (*R*)-**CPA4** (10 mol%) and indole derivative **2d** (0.10 mmol) in 10 mL Schlenk tube was added CH_2_Cl_2_ (1.0 mL) under Ar atmosphere. The reaction mixture was stirred at 0 °C for 20 min. After that, a solution of *o*-naphthoquinone **1c** (0.20 mmol) in CH_2_Cl_2_ (1.0 mL) was added dropwise at 0 °C. Then the reaction mixture was stirred vigorously at 0 °C until **2d** was completely consumed (monitored by thin-layer chromatography). Typical reaction time was about 24 h. The resulting mixture was concentrated under reduced pressure and purified by flash chromatography on silica gel (eluent: PE/DCM = 1/2) to give the corresponding axially chiral arylquinones (*S*)-**3e**.

## Supplementary information

Supplementary Information

## Data Availability

The X-ray crystallographic coordinates for structures reported in this Article have been deposited at the Cambridge Crystallographic Data Centre (CCDC), under deposition number CCDC 1833698. These data can be obtained free of charge from The Cambridge Crystallographic Data Centre via http://www.ccdc.cam.ac.uk/data_request/cif. Supplementary information and chemical compound information are available in the online version of the paper. For NMR analysis and HPLC traces of the compounds in this article, see Supplementary Figures. Reprints and permissions information is available online at www.nature.com/reprints. Correspondence and requests for materials should be addressed to B.T.
