# Supplementary material for: Organocatalytic atroposelective construction of axially chiral arylquinones
Source: Nat Commun. 2019 Sep 19;10:4268. doi: 10.1038/s41467-019-12269-4 (PMC6753127; doi:10.1038/s41467-019-12269-4)
Supplement: Supplementary file 1 — Supplementary Information [file 41467_2019_12269_MOESM1_ESM.pdf]

Supplementary Information

Organocatalytic Atroposelective Construction of Axially Chiral Arylquinones

Zhu *et al.*

Supplementary Tables

Supplementary Table 1. Optimization of the reaction conditions with 2-naphthamine nucleophile[a]

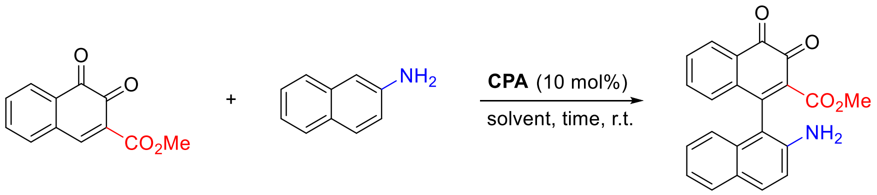

1a-1 2a-1 3a-1

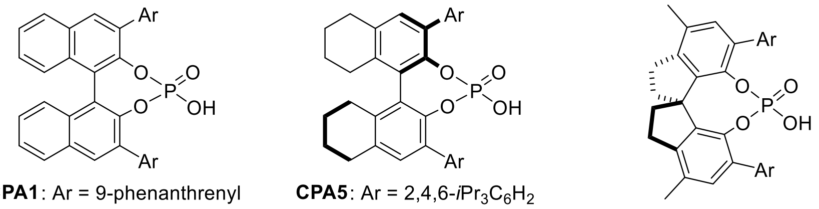

CPA1: Ar = 9-phenanthrenyl
CPA2: Ar = 3,5-$(CF_3)_2C_6H_3$
CPA3: Ar = 4-PhC_6H_4
CPA4: Ar = 2,4,6-*i*$Pr_3C_6H_2$

CPA5: Ar = 2,4,6-*i*$Pr_3C_6H_2$
CPA6: Ar = 9-phenanthrenyl
CPA7: Ar = 1-pyenyl
CPA8: Ar = 2-naphthyl

CPA9: Ar = 2,4,6-*i*$Pr_3C_6H_2$
CPA10: Ar = 4-$CF_3C_6H_4$
CPA11: Ar = 4-PhC_6H_4

CPA12: Ar = C_6F_5
CPA13: Ar = 3,5-$(NO_2)_2C_6H_3$
CPA14: Ar = 1-naphthyl
CPA15: Ar = 9-anthryl
CPA16: Ar = 4-CF_3Ph
CPA17: Ar = 4-OMePh

CPA18: Ar = 3,5-$Ph_2C_6H_3$
CPA19: Ar = 4-*t*BuC_6H_4
CPA20: Ar = Ph_3Si

CPA21: Ar = 9-phenanthrenyl

entry	CPA	solvent	time (min)	yield (%)[b]	ee (%)[c]
**1**	**(*R*)-CPA1**	CH_2Cl_2	240	32	-4
**2**	**(*R*)-CPA2**	CH_2Cl_2	240	38	-36
**3**	**(*R*)-CPA3**	CH_2Cl_2	240	27	-51
**4**	**(*S*)-CPA4**	CH_2Cl_2	20	87	99
**5**	**(*R*)-CPA5**	CH_2Cl_2	20	85	-99
**6**	**(*R*)-CPA6**	CH_2Cl_2	240	36	-83
**7**	**(*R*)-CPA7**	CH_2Cl_2	240	57	-47
**8**	**(*R*)-CPA8**	CH_2Cl_2	240	52	-30
**9**	**(*R*)-CPA9**	CH_2Cl_2	240	24	-28
**10**	**(*R*)-CPA10**	CH_2Cl_2	240	35	-14
**11**	**(*R*)-CPA11**	CH_2Cl_2	240	38	-42
**12**	**(*R*)-CPA12**	CH_2Cl_2	240	trace	-
**13**	**(*R*)-CPA13**	CH_2Cl_2	240	43	-30
**14**	**(*R*)-CPA16**	CH_2Cl_2	240	36	-53
**15**	**(*R*)-CPA17**	CH_2Cl_2	240	40	-23
**16**	**(*R*)-CPA18**	CH_2Cl_2	240	51	-3
**17**	**(*R*)-CPA19**	CH_2Cl_2	240	47	-23
**18**	**(*R*)-CPA20**	CH_2Cl_2	240	40	-5
**19**	**(*S*)-CPA4**	dioxane	20	NR	-

**20**	**(*S*)-CPA4**	THF	20	NR	-
**21**	**(*S*)-CPA4**	toluene	20	60	99
**22**	**(*S*)-CPA4**	$CHCl_3$	20	82	99
**23**	**(*S*)-CPA4**	hexane	20	NR	-
**24**	**(*S*)-CPA4**	EtOAc	20	43	68
**25**	**(*S*)-CPA4**	DCE	20	86	99
**26**	**(*S*)-CPA4**	CH_3OH	20	NR	-
**27**	**(*S*)-CPA4**	ether	20	62	83
**28**	**(*S*)-CPA4**	CH_3CN	20	NR	-
**29**[d]	**(*S*)-CPA4**	CH_2Cl_2	20	86	99
**30**[e]	**(*S*)-CPA4**	CH_2Cl_2	20	86	99

[a] Conditions: **1a-1** (0.22 mmol), **2a-1** (0.10 mmol), solvent (2 mL) at Ar atmosphere, unless noted otherwise. [b] Isolated yield. [c] Determined by HPLC analysis on a chiral stationary phase. [d] 5 mol% **CPA4**. [e] 1 mol% **CPA4**.

Supplementary Table 2. Optimization of the reaction conditions with phenyl protected 2-naphthamine[a]

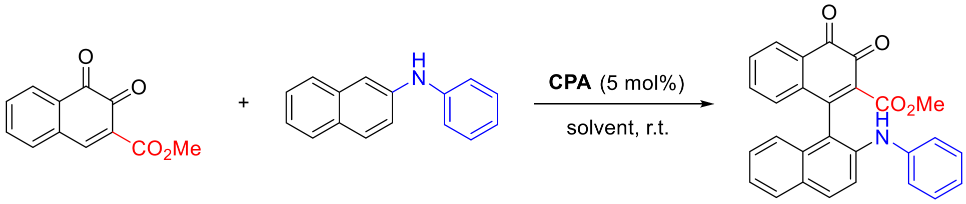

1a-1 **2b-1** **3b-1**

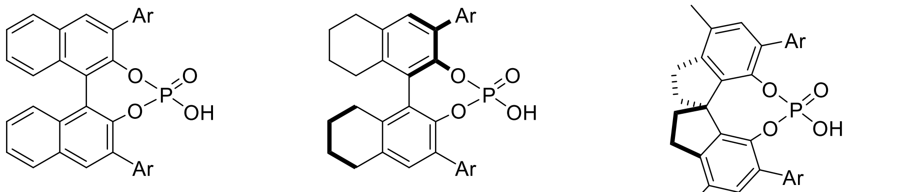

CPA1: Ar = 9-phenanthrenyl
CPA2: Ar = 3,5-$(CF_3)_2C_6H_3$
CPA3: Ar = 4-PhC_6H_4
CPA4: Ar = 2,4,6-*i*$Pr_3C_6H_2$

CPA5: Ar = 2,4,6-*i*$Pr_3C_6H_2$
CPA6: Ar = 9-phenanthrenyl
CPA7: Ar = 1-pyenyl
CPA8: Ar = 2-naphthyl

CPA9: Ar = 2,4,6-*i*$Pr_3C_6H_2$
CPA10: Ar = 4-$CF_3C_6H_4$
CPA11: Ar = 4-PhC_6H_4

CPA12: Ar = C_6F_5
CPA13: Ar = 3,5-$(NO_2)_2C_6H_3$
CPA16: Ar = 4-CF_3Ph
CPA17: Ar = 4-OMePh

CPA18: Ar = 3,5-$Ph_2C_6H_3$
CPA19: Ar = 4-*t*BuC_6H_4
CPA20: Ar = Ph_3Si

CPA21: Ar = 9-phenanthrenyl

entry	CPA	solvent	yield (%)[b]	ee (%)[c]
**1**	**(*R*)-CPA1**	CH_2Cl_2	86	-76
**2**	**(*R*)-CPA2**	CH_2Cl_2	81	-43
**3**	**(*R*)-CPA3**	CH_2Cl_2	82	-51
**4**	**(*S*)-CPA4**	CH_2Cl_2	16	24
**5**	**(*R*)-CPA5**	CH_2Cl_2	24	-32
**6**	**(*R*)-CPA6**	CH_2Cl_2	94	-99
**7**	**(*R*)-CPA7**	CH_2Cl_2	72	-94
**8**	**(*R*)-CPA8**	CH_2Cl_2	70	-81
**9**	**(*R*)-CPA11**	CH_2Cl_2	35	-6
**10**	**(*R*)-CPA12**	CH_2Cl_2	84	-81
**11**	**(*R*)-CPA13**	CH_2Cl_2	65	-35
**12**	**(*R*)-CPA16**	CH_2Cl_2	82	-51
**13**	**(*R*)-CPA17**	CH_2Cl_2	84	-33
**14**[d]	**(*R*)-CPA6**	CH_2Cl_2	90	-99
**15**[e]	**(*R*)-CPA6**	CH_2Cl_2	94	-99
**16**	**(*R*)-CPA6**	toluene	70	-99
**17**	**(*R*)-CPA6**	$CHCl_3$	85	-99
**18**	**(*R*)-CPA6**	DCE	90	-99
**19**	**(*R*)-CPA6**	CH_3OH	NR	-
**20**	**(*R*)-CPA6**	ether	45	-72

[a] Conditions: **1a-1** (0.22 mmol), **2b-1** (0.10 mmol), solvent (2 mL) at Ar atmosphere, unless noted otherwise. [b] Isolated yield. [c] Determined by HPLC analysis on a chiral stationary phase. [d] The reaction was conducted with 1 mol% **CPA6**. [e] The reaction was conducted with 10 mol% **CPA6**.

Supplementary Table 3. Optimization of the reaction conditions with *o*-naphthoquinone[a]

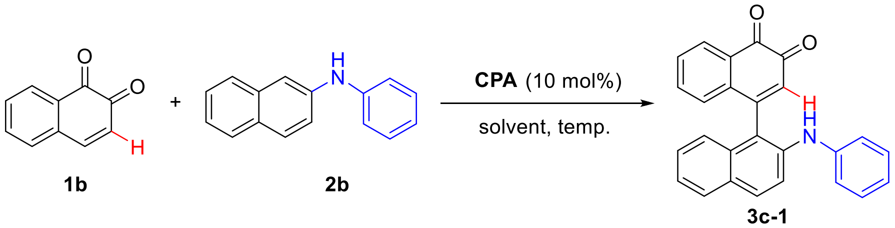

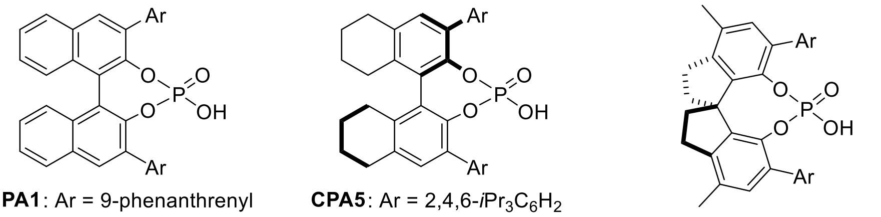

CPA1: Ar = 9-phenanthrenyl
CPA2: Ar = 3,5-$(CF_3)_2C_6H_3$
CPA3: Ar = 4-PhC_6H_4
CPA4: Ar = 2,4,6-*i*$Pr_3C_6H_2$

CPA5: Ar = 2,4,6-*i*$Pr_3C_6H_2$
CPA6: Ar = 9-phenanthrenyl
CPA7: Ar = 1-pyenyl
CPA8: Ar = 2-naphthyl

CPA9: Ar = 2,4,6-*i*$Pr_3C_6H_2$
CPA10: Ar = 4-$CF_3C_6H_4$
CPA11: Ar = 4-PhC_6H_4

entry	CPA	solvent	time (h)	yield (%)[b]	ee (%)[c]
**1**	**(*R*)-CPA1**	CH_2Cl_2	12	63	58
**2**	**(*R*)-CPA2**	CH_2Cl_2	12		
**3**	**(*R*)-CPA3**	CH_2Cl_2	12	42	32
**4**	**(*S*)-CPA4**	CH_2Cl_2	12	92	-83
**5**	**(*R*)-CPA5**	CH_2Cl_2	12	62	65
**6**	**(*R*)-CPA6**	CH_2Cl_2	12	78	46
**7**	**(*R*)-CPA7**	CH_2Cl_2	12	81	42
**8**	**(*R*)-CPA8**	CH_2Cl_2	12	83	-30
**9**	**(*R*)-CPA9**	CH_2Cl_2	12	trace	
**10**	**(*S*)-CPA4**	toluene	12	90	-78
**11**	**(*S*)-CPA4**	xylene	12	91	-77
**12**	**(*S*)-CPA4**	PhBr	12	67	-82
**13**	**(*S*)-CPA4**	EtOAc	12	NR	
**14**	**(*S*)-CPA4**	acetone	12	NR	
**15**	**(*S*)-CPA4**	hexane	12	55	-72
**16**	**(*S*)-CPA4**	DCE	12	80	-82
**17**	**(*S*)-CPA4**	DCM	24	75	-88
**18**	**(*S*)-CPA4**	DCM	24	63	-93
**19**	**(*S*)-CPA4**	DCM	24	30	-92
**20**[d]	**(*S*)-CPA4**	DCM	24	46	-89
**21**[e]	**(*S*)-CPA4**	DCM	24	54	-92

[a] Conditions: **1b-1** (0.22 mmol), **2a-1** (0.10 mmol), solvent (2 mL) at Ar atmosphere, unless noted otherwise. [b] Isolated yield. [c] Determined by HPLC analysis on a chiral stationary phase. [d] The reaction was conducted with 1 mol% **CPA4**; [e] The reaction was conducted with 5 mol% **CPA4**.

Supplementary Table 4. Optimization of the reaction conditions with 2-naphthol by screening the chiral phosphoric acids and solvents[a]

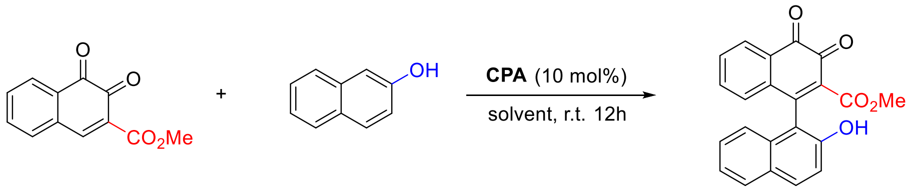

1a-1 **2c-1** **3d-1**

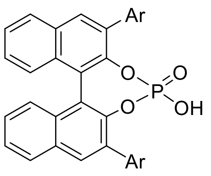

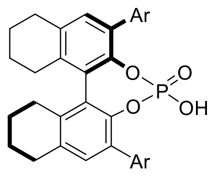

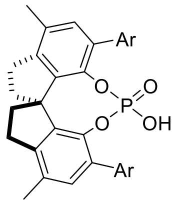

CPA1: Ar = 9-phenanthrenyl
CPA2: Ar = 3,5-$(CF_3)_2C_6H_3$
CPA3: Ar = 4-PhC_6H_4
CPA4: Ar = 2,4,6-*i*$Pr_3C_6H_2$

CPA12: Ar = C_6F_5
CPA13: Ar = 3,5-$(NO_2)_2C_6H_3$
CPA14: Ar = 1-naphthyl
CPA15: Ar = 9-anthryl
CPA16: Ar = 4-CF_3Ph
CPA17: Ar = 4-OMePh

CPA5: Ar = 2,4,6-*i*$Pr_3C_6H_2$
CPA6: Ar = 9-phenanthrenyl
CPA7: Ar = 1-pyenyl
CPA8: Ar = 2-naphthyl

CPA18: Ar = 3,5-$Ph_2C_6H_3$
CPA19: Ar = 4-*t*BuC_6H_4
CPA20: Ar = Ph_3Si

CPA9: Ar = 2,4,6-*i*$Pr_3C_6H_2$
CPA10: Ar = 4-$CF_3C_6H_4$
CPA11: Ar = 4-PhC_6H_4

CPA21: Ar = 9-phenanthrenyl

entry	CPA	solvent	yield (%)[b]	ee (%)[c]
**1**	**(*R*)-CPA1**	CH_2Cl_2	82	-20
**2**	**(*R*)-CPA2**	CH_2Cl_2	73	-6
**3**	**(*R*)-CPA3**	CH_2Cl_2	75	-32
**4**	**(*S*)-CPA4**	CH_2Cl_2	79	35
**5**	**(*R*)-CPA5**	CH_2Cl_2	81	-25
**6**	**(*R*)-CPA6**	CH_2Cl_2	78	-31
**7**	**(*R*)-CPA7**	CH_2Cl_2	80	-65
**8**	**(*R*)-CPA8**	CH_2Cl_2	72	-55
**9**	**(*R*)-CPA9**	CH_2Cl_2	56	-4
**10**	**(*R*)-CPA10**	CH_2Cl_2	83	-7
**11**	**(*R*)-CPA13**	CH_2Cl_2	74	-23
**12**	**(*R*)-CPA14**	CH_2Cl_2	77	-31
**13**	**(*R*)-CPA16**	CH_2Cl_2	83	-11
**14**	**(*R*)-CPA17**	CH_2Cl_2	78	-12
**15**	**(*R*)-CPA18**	CH_2Cl_2	84	-4
**16**	**(*R*)-CPA19**	CH_2Cl_2	82	-45
**17**	**(*R*)-CPA21**	CH_2Cl_2	77	-7
**18**	**(*R*)-CPA7**	DCE	84	-49
**19**	**(*R*)-CPA7**	$CHCl_3$	81	-58
**20**	**(*R*)-CPA7**	$(CHCl_2)_2$	74	-24
**21**	**(*R*)-CPA7**	CH_3CN	76	-55

**22**	**(*R*)-CPA7**	THF	NR	-
**23**	**(*R*)-CPA7**	pentane	NR	-
**24**	**(*R*)-CPA7**	EtOAc	78	-22
**25**	**(*R*)-CPA7**	benzene	67	-49

[a] Conditions: **1a-1** (0.22 mmol), **2c-1** (0.10 mmol), solvent (2 mL) at Ar atmosphere, unless noted otherwise. [b] Isolated yield. [c] Determined by HPLC analysis on a chiral stationary phase.

Supplementary Table 5. Optimization of the reaction conditions with 2-naphthol by screening the temperatures and additives[a]

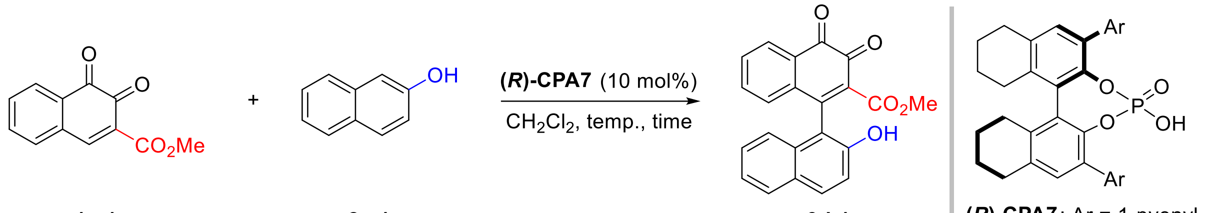

entry	additive	T (℃)	time (h)	yield (%)[b]	ee (%)[c]
**1**		0	24	88	-77
**2**		-10	24	90	-83
**3**		-20	24	91	-78
**4**		-30	24	84	-69
**5**[d]		-10	24	86	-83
**6**[e]		-10	24	88	-88
**7**[f]		-10	24	89	-88
**8**	$Cu(PO_3)_2$	-10	24	86	-73
**9**	CsCl	-10	24	84	34
**10**	$Pd(OAc)_2$	-10	24	85	-82
**11**	$InCl_3$	-10	24	86	-74
**12**	InF_3	-10	24	85	-51
**13**	$BiCl_3$	-10	24	88	-19
**14**	BF_4Li	-10	24	90	-92
**15**	$CoBr_2$	-10	24	87	-73
**16**	BF_4Na	-10	24	90	-86

[a] Conditions: **1a-1** (0.22 mmol), **2c-1** (0.10 mmol), catalyst (10 mol%), additive (8 mol%), solvent (2 mL) at Ar atmosphere, unless noted otherwise. [b] Isolated yield. [c] Determined by HPLC analysis on a chiral stationary phase. [d] The reaction was conducted with 1 mL of CH_2Cl_2. [e] The reaction was conducted with 3 mL of CH_2Cl_2. [f] The reaction was conducted with 4 mL of CH_2Cl_2.

Supplementary Table 6. Optimization of the reaction conditions with indole[a]

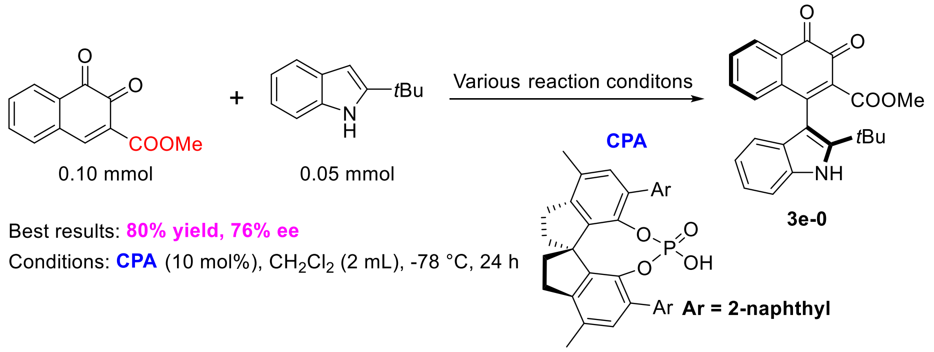

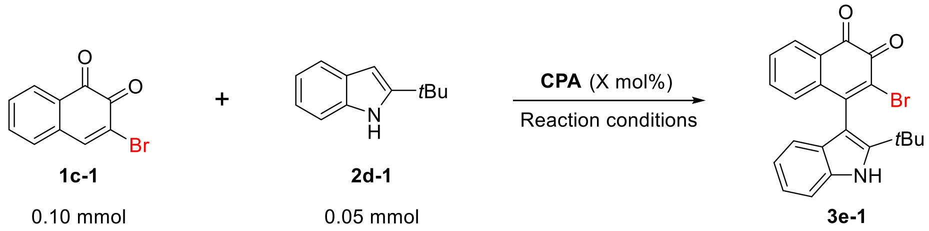

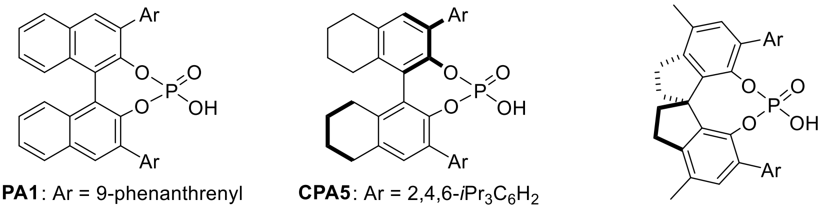

CPA1: Ar = 9-phenanthrenyl
CPA2: Ar = 3,5-$(CF_3)_2C_6H_3$
CPA3: Ar = 4-PhC_6H_4
CPA4: Ar = 2,4,6-*i*$Pr_3C_6H_2$

CPA5: Ar = 2,4,6-*i*$Pr_3C_6H_2$
CPA6: Ar = 9-phenanthrenyl
CPA7: Ar = 1-pyenyl
CPA8: Ar = 2-naphthyl

CPA9: Ar = 2,4,6-*i*$Pr_3C_6H_2$
CPA10: Ar = 4-$CF_3C_6H_4$
CPA11: Ar = 4-PhC_6H_4

CPA12: Ar = C_6F_5
CPA13: Ar = 3,5-$(NO_2)_2C_6H_3$
CPA14: Ar = 1-naphthyl
CPA15: Ar = 9-anthryl
CPA16: Ar = 4-CF_3Ph
CPA17: Ar = 4-OMePh

CPA18: Ar = 3,5-$Ph_2C_6H_3$
CPA19: Ar = 4-*t*BuC_6H_4
CPA20: Ar = Ph_3Si

CPA21: Ar = 9-phenanthrenyl

entry	CPA	X	solvent	temp.	time	yield (%)[b]	ee (%)[c]
1	**(*R*)-CPA3**	10	CH_2Cl_2	r.t.	24 h	94	17
**2**	**(*R*)-CPA4**	10	CH_2Cl_2	r.t.	24 h	97	89
3	**(*R*)-CPA14**	10	CH_2Cl_2	r.t.	24 h	88	5
4	**(*R*)-CPA15**	10	CH_2Cl_2	r.t.	24 h	92	59
5	**(*R*)-CPA16**	10	CH_2Cl_2	r.t.	24 h	85	6
6	**(*R*)-CPA4**	10	toluene	r.t.	24 h	92	88
7	**(*R*)-CPA4**	10	DCE	r.t.	24 h	95	88
8	**(*R*)-CPA4**	10	$CHCl_3$	r.t.	24 h	93	66
9	**(*R*)-CPA4**	10	benzene	r.t.	24 h	94	88
10	**(*R*)-CPA4**	10	chlorobenzene	r.t.	24 h	93	87
11	**(*R*)-CPA4**	5	CH_2Cl_2	r.t.	24 h	92	85
12	**(*R*)-CPA4**	2.5	CH_2Cl_2	r.t.	24 h	90	82
**13**	**(*R*)-CPA4**	10	CH_2Cl_2	0 ℃	24 h	97(95)[d]	96

14	**(*R*)-CPA4**	10	CH_2Cl_2	-10 ℃	24 h	92	96
15[e]	**(*R*)-CPA4**	10	CH_2Cl_2	0 ℃	24 h	82	96
16[f]	**(*R*)-CPA4**	10	CH_2Cl_2	0 ℃	24 h	97	96
17	**(*R*)-CPA4**	10	CH_2Cl_2	0 ℃	12 h	85	96
18	**(*R*)-CPA4**	10	CH_2Cl_2	0 ℃	36 h	97	96
19[g]	**(*R*)-CPA4**	10	CH_2Cl_2	0 ℃	24 h	97	92
20[h]	**(*R*)-CPA4**	10	CH_2Cl_2	0 ℃	24 h	97	96

[a] Conditions: **1c-1** (0.10 mmol), **2d-1** (0.05 mmol), solvent (2 mL) at Ar atmosphere, unless noted otherwise. [b] Determined by HPLC analysis on a chiral stationary phase. [c] Determined by ^{1}H-NMR analysis of crude mixture with 1,3,5-triethylbenzene as an internal standard. [d] Isolated yield. [e] Conducted with 0.075 mmol **1c-1**. [f] Conducted with 0.15 mmol **1c-1**. [g] Conducted in 1 mL CH_2Cl_2. [h] Conducted in 3 mL CH_2Cl_2.

Supplementary Table 7. Crystal data and structure refinement for 9.

Empirical formula	$C_{42}H_{29}N_3O_2$
Formula weight	607.68
Temperature/K	100
Crystal system	monoclinic
Space group	$P2_1$
a/Å	9.5643(8)
b/Å	36.917(3)
c/Å	9.9590(8)
α/°	90
β/°	116.810(3)
γ/°	90
Volume/Å^3	3138.4(5)
Z	4
ρ_{calc}g/cm^3	1.286
μ/mm^{-1}	0.627
F(000)	1272.0
Crystal size/mm^3	0.35 × 0.18 × 0.15
Radiation	CuKα (λ = 1.54178)
2Θ range for data collection/ °	4.788 to 130.676
Index ranges	$-11 \leq h \leq 11$, $-43 \leq k \leq 43$, $-11 \leq l \leq 11$
Reflections collected	35073
Independent reflections	10620 [R_{int} = 0.0623, R_{sigma} = 0.0610]
Data/restraints/parameters	10620/1/850
Goodness-of-fit on F^2	1.058
Final R indexes [I>=2σ (I)]	R_1 = 0.0424, wR_2 = 0.1055
Final R indexes [all data]	R_1 = 0.0462, wR_2 = 0.1088
Largest diff. peak/hole / e Å^{-3}	0.34/-0.25
Flack parameter	-0.02(16)

Supplementary Notes

Supplementary Note 1

General Information

Chemicals were purchased from commercial suppliers and used without further purification unless otherwise stated. Chiral phosphoric acid (**CPA**) was purchased from Daicel Chiral Technologies (China). Analytical thin layer chromatography (TLC) was performed on precoated silica gel 60 GF254 plates. Flash column chromatography was performed using Tsingdao silica gel (60, particle size 0.040-0.063 mm). Visualization on TLC was achieved by use of UV light (254 nm) or iodine. ^{1}H and ^{13}C NMR spectra were recorded on Bruker DPX 400 and Bruker DPX 500 spectrometer using $CDCl_3$, Acetone-d_6 or DMSO-d_6 with tetramethylsilane (TMS) as internal standard. The chemical shifts are expressed in ppm and coupling constants are given in Hz. Data for ^{1}H NMR are recorded as follows: chemical shift (δ, ppm), multiplicity (s = singlet; d = doublet; t = triplet; q = quartet; p = pentet; m = multiplet; br = broad), coupling constant (Hz), integration. Data for ^{13}C NMR are reported in terms of chemical shift (δ, ppm). Mass spectrometric data were obtained using Bruker Apex IV RTMS. The enantiomeric excess values were determined by chiral HPLC with a Shimadzu SIL-20A instrument and Daicel chiral columns. High resolution mass spectroscopy (HRMS) analyses were performed at a Bruker Daltonics. Inc mass instrument (ESI), Thermo Scientific. Q-Exactive (HESI) and Thermo Scientific. Orbitrap Fusion (HESI).

Supplementary Note 2

Procedure for the Syntheses of Substrates

(a) Syntheses of the *o*-naphthoquinones

Substituted *o*-naphthoquinones 1a-1~1a-3, 1a-6~1a-9 were prepared according to the following procedure.

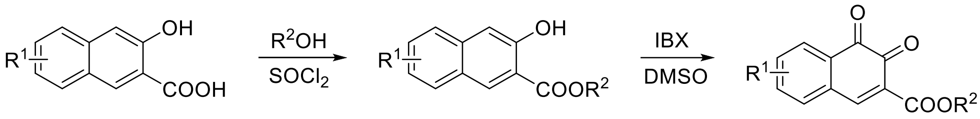

To a solution of 3-Hydroxy-2-naphthoic acid 53.2 mmol in 100 mL corresponding alcohol was added 10 mL thionyl chloride dropwise at 0 ℃. Then the reaction mixture was stirred for 10 min before heated to reflux for another 10 h. After cooling to room temperature, yellow solid was precipitated. The precipitate was collected by filtration and washed with 500 mL water. The collected solid was dried by vacuum oven to quantitatively yield a yellow solid. Subsequently, 10 mmol of the obtained solid was dissolved in 100 mL DMSO and 3.36 g (12 mmol) IBX was added in portions. The reaction mixture was stirred at room temperature for 6 h. After the reaction completion (monitored by TLC), the mixture was washed with water (100 mL*2), saturated sodium bicarbonate solution (100 mL*3) and brine (100 mL*2) The organic solution was dried over anhydrous Na_2SO_4, filtered and concentrated to give the residue. Then the residue was dissolved in minimum DCM and added plenty of PE, red solid was precipitated gradually. The precipitation was dried by vacuum oven to give **1a** as the pure product.

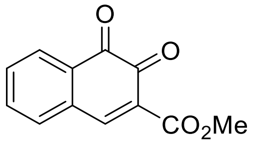

1a-1

Methyl 3,4-dioxo-3,4-dihydronaphthalene-2-carboxylate (1a-1)

The title compound was prepared in 95% yield according to the general procedure.

^{1}H NMR (500 MHz, $CDCl_3$) δ 8.25 (s, 1H), 8.15 (d, J = 7.6 Hz, 1H), 7.73 (t, J = 7.5 Hz, 1H), 7.64 (t, J = 7.6 Hz, 1H), 7.54 (d, J = 7.5 Hz, 1H), 3.92 (s, 3H);

^{13}C NMR (125 MHz, $CDCl_3$) δ 178.12, 177.00, 163.77, 150.57, 136.17, 132.94, 132.91, 132.24, 132.07, 130.67, 129.13, 52.91.

HRMS (ESI) calcd for [M+H] $C_{12}H_{9}O_{4}$, m/z: 217.0495, found: 217.0490.

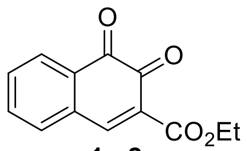

1a-2

Ethyl 3,4-dioxo-3,4-dihydronaphthalene-2-carboxylate (1a-2)

The title compound was prepared in 96% yield according to the general procedure.

^{1}H NMR (400 MHz, $CDCl_3$) δ 8.20 (s, 1H), 8.17-8.12 (m, 1H), 7.73 (td, *J* = 7.5, 1.4 Hz, 1H), 7.62 (td, *J* = 7.6, 1.3 Hz, 1H), 7.54 (dd, *J* = 7.5, 1.2 Hz, 1H), 4.37 (q, *J* = 7.1 Hz, 2H), 1.38 (t, *J* = 7.1 Hz, 3H);

^{13}C NMR (100 MHz, $CDCl_3$) δ 178.21, 177.12, 163.21, 149.88, 136.11, 133.05, 132.75, 132.22, 131.94, 130.61, 129.63, 62.02, 14.32.

HRMS (ESI) calcd for [M+H] $C_{13}H_{11}O_{4}$, m/z: 231.0652, found: 231.0647.

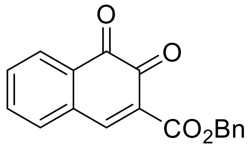

1a-3

Benzyl 3,4-dioxo-3,4-dihydronaphthalene-2-carboxylate (1a-3)

The title compound was prepared in 95% yield according to the general procedure.

^{1}H NMR (500 MHz, $CDCl_3$) δ 8.23 (s, 1H), 8.15 (dd, *J* = 7.6, 1.3 Hz, 1H), 7.72 (td, *J* = 7.6, 1.4 Hz, 1H), 7.63 (td, *J* = 7.6, 1.2 Hz, 1H), 7.53 (d, *J* = 7.5 Hz, 1H), 7.48 (d, *J* = 7.0 Hz, 2H), 7.42-7.37 (m, 2H), 7.36-7.32 (m, 1H), 5.36 (s, 2H).

^{13}C NMR (125 MHz, $CDCl_3$) δ 178.08, 176.94, 163.02, 150.47, 136.14, 135.39, 132.93, 132.91, 132.22, 132.09, 130.67, 129.15, 128.78, 128.54, 128.30, 67.53.

HRMS (ESI) calcd for [M+H] $C_{18}H_{13}O_{4}$, m/z: 293.0808, found: 293.0799.

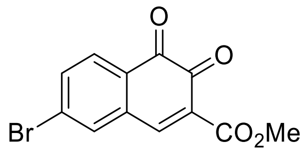

1a-6

Methyl 7-bromo-3,4-dioxo-3,4-dihydronaphthalene-2-carboxylate (1a-6)

The title compound was prepared in 95% yield according to the general procedure.

^{1}H NMR (500 MHz, CDCl$_3$) δ 8.17 (s, 1H), 8.01 (d, *J* = 8.2 Hz, 1H), 7.78 (dd, *J* = 8.2, 1.8 Hz, 1H), 7.71 (d, *J* = 1.8 Hz, 1H), 3.93 (s, 3H).

^{13}C NMR (125 MHz, CDCl$_3$) δ 177.26, 176.44, 163.39, 148.66, 135.71, 134.56, 134.27, 131.86, 131.63, 130.70, 130.35, 53.06.

HRMS (ESI) calcd for [M+H] $C_{12}H_8O_4{}^{79}Br$, m/z: 294.9600, found: 294.9593; $C_{12}H_8O_4{}^{81}Br$, m/z: 296.9585, found: 296.9563.

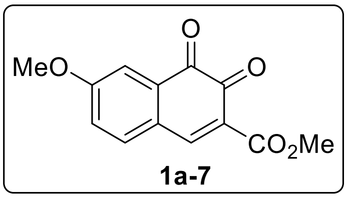

Methyl 6-methoxy-3,4-dioxo-3,4-dihydronaphthalene-2-carboxylate (1a-7)

The title compound was prepared in 95% yield according to the general procedure.

^{1}H NMR (400 MHz, DMSO-*d*6) δ 7.43 (s, 1H), 7.11 (d, *J* = 8.6 Hz, 1H), 6.60 (d, *J* = 2.6 Hz, 1H), 6.34 (dd, *J* = 8.6, 2.6 Hz, 1H), 3.06 (s, 3H), 2.95 (s, 3H).

^{13}C NMR (100 MHz, DMSO-*d*6) δ 177.05, 176.37, 165.21, 163.87, 148.96, 135.48, 132.32, 129.67, 125.95, 118.20, 117.32, 56.62, 52.62.

HRMS (ESI) calcd for [M+H] $C_{13}H_{11}O_5$, m/z: 247.0601, found: 247.0594.

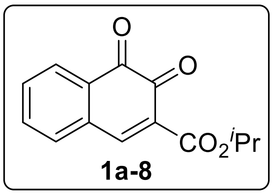

Isopropyl 3,4-dioxo-3,4-dihydronaphthalene-2-carboxylate (1a-8)

The title compound was prepared in 96% yield according to the general procedure.

^{1}H NMR (500 MHz, CDCl$_3$) δ 8.19-8.05 (m, 2H), 7.72 (td, *J* = 7.6, 1.2 Hz, 1H), 7.61 (td, *J* = 7.6, 1.1 Hz, 1H), 7.53 (d, *J* = 7.6 Hz, 1H), 5.26-5.18 (m, 1H), 1.36 (d, *J* = 6.3 Hz, 6H).

^{13}C NMR (125 MHz, CDCl$_3$) δ 178.25, 177.18, 162.66, 149.33, 136.09, 133.10, 132.64, 132.14, 131.86, 130.57, 130.02, 69.82, 21.94.

HRMS (ESI) calcd for [M+H] $C_{14}H_{13}O_4$, m/z: 245.0808, found: 245.0800.

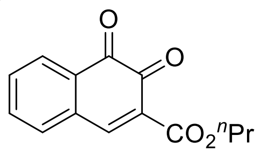

1a-9

Propyl 3,4-dioxo-3,4-dihydronaphthalene-2-carboxylate (1a-9)

The title compound was prepared in 96% yield according to the general procedure.

^{1}H NMR (400 MHz, DMSO-*d*$_6$) δ 8.24 (s, 1H), 7.94 (d, *J* = 7.6 Hz, 1H), 7.80-7.72 (m, 2H), 7.63 (td, *J* = 7.4, 1.5 Hz, 1H), 4.15 (t, *J* = 6.5 Hz, 2H), 1.66 (h, *J* = 7.1 Hz, 2H).

^{13}C NMR (100 MHz, DMSO-*d*$_6$) δ 177.17, 176.06, 163.26, 148.29, 135.26, 132.94, 132.60, 132.21, 132.08, 129.14, 128.81, 66.33, 21.61, 10.40.

HRMS (ESI) calcd for [M+H] $C_{14}H_{13}O_4$, m/z: 245.0808, found: 245.0805.

Substituted *o*-naphthoquinone 1a-4~1a-5 were prepared according to the following procedure.

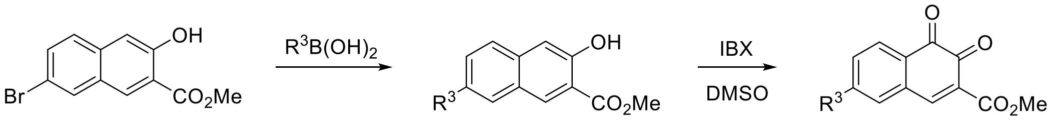

1a-4~1a-5

An oven-dried Schlenk tube containing a stirring bar was charged with $Pd(OAc)_2$ (22.4 mg, 2 mol%), SPhos (82.0 mg, 4 mol%), K_3PO_4 (1.48 g, 7 mmol), corresponding boronic acid 7 mmol and methyl 7-bromo-3,4-dioxo-3,4-dihydronaphthalene-2-carboxylate 1.47 g (5 mmol). The Schlenk tube was evacuated and back-filled with dry argon (this sequence was repeated for three times). Then, dry toluene (10 mL) was added by syringe. The mixture was then stirred in a pre-heated oil bath (110 ℃) for 12 h. The mixture was allowed to cool to room temperature, diluted with EtOAc (10 mL) and filtered through a Celite® plug, eluted with additional EtOAc (30 mL). The filtrate was concentrated and purified by column chromatography on silica gel (eluent: hexane/EA=8:1). The esterification product was obtained as a yellow solid (1.19 g, 92% yield). Then the yellow solid was dissolved in 100 mL DMSO and 1.55 g (5.5 mmol) IBX was added in portions. The reaction mixture was stirred at room temperature for 6 h. After the reaction completion (monitored by TLC), the mixture was washed with water (100 mL*2), saturated sodium bicarbonate solution (100 mL*3) and brine (100 mL*2). The organic solution was dried over anhydrous Na_2SO_4, filtered and

concentrated to give the residue. Then the residue was dissolved in minimum DCM and added plenty of PE, yellow solid was precipitated gradually. The precipitation was dried by vacuum oven to give **1a** as pure product.

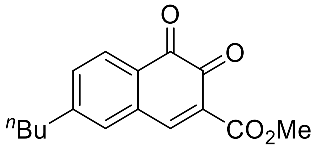

1a-4

Methyl 7-butyl-3,4-dioxo-3,4-dihydronaphthalene-2-carboxylate (1a-4)

The title compound was prepared in 95% yield according to the general procedure**.**

^{1}H NMR (500 MHz, CDCl$_3$) δ 8.21 (s, 1H), 8.06 (d, *J* = 7.8 Hz, 1H), 7.42 (dd, *J* = 7.9, 1.5 Hz, 1H), 7.33 (d, *J* = 1.6 Hz, 1H), 3.91 (s, 3H), 2.72 (t, *J* = 7.7 Hz, 2H), 1.82-1.56 (m, 2H), 1.42-1.33 (m, 2H), 0.95 (t, *J* = 7.3 Hz, 3H).

^{13}C NMR (125 MHz, CDCl$_3$) δ 177.78, 177.36, 163.88, 152.71, 150.92, 132.98, 132.91, 132.22, 130.96, 130.11, 128.99, 52.85, 35.94, 32.98, 22.38, 13.98.

HRMS (ESI) calcd for [M+H] $C_{16}H_{17}O_4$, m/z: 273.1121, found: 273.1113.

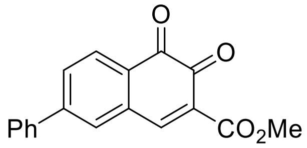

1a-5

Methyl 3,4-dioxo-7-phenyl-3,4-dihydronaphthalene-2-carboxylate (1a-5)

The title compound was prepared in 95% yield according to the general procedure.

^{1}H NMR (400 MHz, CDCl$_3$) δ 8.31 (s, 1H), 8.21 (d, *J* = 7.9 Hz, 1H), 7.82 (dd, *J* = 8.0, 1.7 Hz, 1H), 7.74 (d, *J* = 1.8 Hz, 1H), 7.69-7.61 (m, 2H), 7.57-7.44 (m, 3H), 3.92 (s, 3H).

^{13}C NMR (100 MHz, CDCl$_3$) δ 177.74, 177.15, 163.78, 150.42, 149.11, 138.34, 133.48, 131.36, 130.97, 130.69, 130.63, 129.60, 129.46, 127.33, 52.90.

HRMS (ESI) calcd for [M+H] $C_{18}H_{13}O_4$, m/z: 293.0808, found: 293.0798.

Substituted *o*-naphthoquinones 1c-1~1c-3 were prepared according to following procedure.

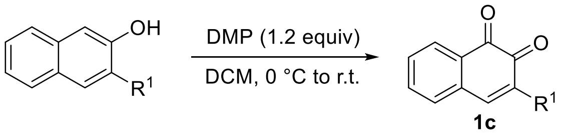

To a solution of Dess-Martin periodinane (DMP, 6.0 mmol) in 30 mL CH_2Cl_2 was added a solution of 3-R^1-2-naphthol (5.0 mmol) in 20 mL CH_2Cl_2 dropwise at 0 ℃. Then the reaction mixture was stirred for 12 h at room temperature, the mixture was washed with saturated sodium bicarbonate solution (20 mL*3) and brine (20 mL*2). The organic solution was dried over anhydrous Na_2SO_4, filtered and concentrated under reduced pressure. Then the residue was purified by column chromatography on silica gel (eluent: PE/DCM=1:1) to give the desired product.

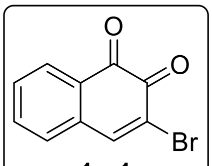

1c-1

Compound **1c-1** was prepared in 65% yield according to the general procedure.

^{1}H NMR (400 MHz, $CDCl_3$) δ 8.07 (d, *J* = 8.0 Hz, 1H), 7.84 (s, 1H), 7.66-7.62 (m, 1H), 7.51 (t, *J* = 8.0 Hz, 1H), 7.32 (d, *J* = 8.0 Hz, 1H).

^{13}C NMR (100 MHz, $CDCl_3$) δ 176.87, 174.67, 146.33, 136.40, 134.71, 131.27, 130.98, 130.70, 129.75, 122.69.

HRMS (ESI) calcd for [M+H] $C_{10}H_6{}^{79}BrO_2$, m/z: 236.9551, found: 236.9555; [M+H] $C_{10}H_6{}^{81}BrO_2$, m/z: 238.9531, found: 238.9535.

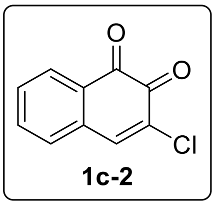

1c-2

Compound **1c-2** was prepared in 60% yield according to the general procedure.

^{1}H NMR (400 MHz, $CDCl_3$) δ 8.09 (d, *J* = 8.0 Hz, 1H), 7.66 (t, *J* = 8.0 Hz, 1H), 7.60 (s, 1H), 7.51 (t, *J* = 8.0 Hz, 1H), 7.34 (d, *J* = 8.0 Hz, 1H).

^{13}C NMR (100 MHz, $CDCl_3$) δ 177.46, 174.79, 142.19, 136.44, 133.90, 131.70, 131.17, 131.01, 130.51, 129.88.

HRMS (ESI) calcd for [M+H] $C_{10}H_6{}^{35}ClO_2$, m/z: 193.0056, found: 193.0057; [M+H]

$C_{10}H_6{}^{37}ClO_2$, m/z: 195.0027, found: 195.0029.

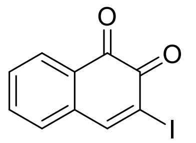

1c-3

Compound **1c-3** was prepared in 60% yield according to the general procedure.

^{1}H NMR (400 MHz, CDCl$_3$) δ 8.16 (s, 1H), 8.08 (d, *J* = 8.0 Hz, 1H), 7.65 (t, *J* = 8.0 Hz, 1H), 7.53 (t, *J* = 8.0 Hz, 1H), 7.31 (d, *J* = 8.0 Hz, 1H).

^{13}C NMR (100 MHz, CDCl$_3$) δ 175.77, 175.66, 153.92, 136.32, 136.16, 131.45, 131.22, 130.87, 129.41, 101.19.

HRMS (ESI) calcd for [M+H] $C_{10}H_6IO_2$, m/z: 284.94070, found: 284.93967.

(b) Synthesis of the substituted 2-aminonaphthalene

Compounds **2a-1**, **2a-3**, **2a-4** are commercial available. Compounds **2a-2, 2a-5~2a-8** were synthesized according to the literature.[1]

(c) Synthesis of the substituted *N*-phenylnaphthalen-2-amine

Compounds **2b-1**, **2b-5** are commercial available. Compounds **2b-2~2b-4, 2b-6, 2b-7** were synthesized according to the literature.[2]

(d) Syntheses of substituted 2-naphthols

Substituted 2-naphthol **2c-2** was prepared according to the following procedure. Compounds **2c-1**, **2c-3~2c-5** are commercial available.

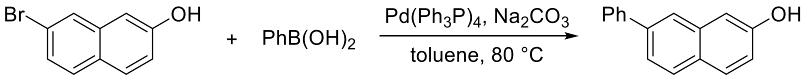

2c-4 2c-2

A mixture of 2-naphthol derivatives 10 mmol, phenylboronic acid 15 mmol, 2% aqueous solution of sodium carbonate (2 equiv), and Pd(PPh$_3$)$_4$ (0.1 equiv) in toluene was stirred at 80 ℃ under nitrogen for 16 h. The reaction mixture was cooled to room temperature and then water was added. The mixture was extracted with dichloromethane, washed with brine, dried over MgSO$_4$, and concentrated. The product was purified by column chromatography (eluent:

PE/EA=8:1).

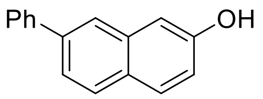

2c-2

7-Phenylnaphthalen-2-ol (2c-2)

The title compound was prepared in 85% yield according to the general procedure.

^{1}H NMR (400 MHz, DMSO-d_6) δ 9.73 (s, 1H), 7.89 (d, *J* = 1.7 Hz, 1H), 7.75 (d, *J* = 8.5 Hz, 1H), 7.71-7.64 (m, 3H), 7.47 (dd, *J* = 8.5, 1.8 Hz, 1H), 7.39 (t, *J* = 7.7 Hz, 2H), 7.31-7.24 (m, 1H), 7.16 (d, *J* = 2.3 Hz, 1H), 7.02 (dd, *J* = 8.8, 2.4 Hz, 1H).

^{13}C NMR (100 MHz, DMSO-d_6) δ 155.74, 140.33, 137.73, 135.00, 129.08, 128.94, 128.28, 127.41, 127.01, 126.94, 123.61, 121.88, 118.83, 109.16.

HRMS (ESI) calcd for [M+H] $C_{16}H_{17}O_4$, m/z: 219.0815, found: 219.0812.

(e) Substituted indoles 2d-1~2d-6 were prepared according to literature methods.[3]

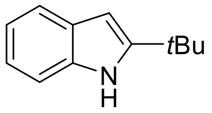

2d-1

Compound **2d-1** was prepared in 95% yield.

^{1}H NMR (400 MHz, CDCl$_3$) δ 7.96 (s, 1H), 7.68 (d, *J* = 8.0 Hz, 1H), 7.40 (d, *J* = 8.0 Hz, 1H), 7.27-7.18 (m, 2H), 6.37 (s, 1H), 1.48 (s, 9H).

^{13}C NMR (100 MHz, CDCl$_3$) δ 148.89, 135.86, 128.59, 121.15, 120.07, 119.69, 110.51, 97.03, 31.88, 30.37.

HRMS (ESI) calcd for [M+H] $C_{12}H_{16}N$, m/z: 174.1277, found: 174.1276.

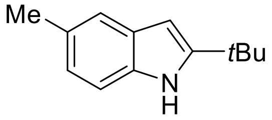

2d-2

Compound **2d-2** was prepared in 95% yield.

^{1}H NMR (400 MHz, CDCl$_3$) δ 7.87 (s, 1H), 7.39 (s, 1H), 7.26 (d, *J* = 8.0 Hz, 1H), 7.02 (d, *J* = 8.0 Hz, 1H), 6.24-6.23 (m, 1H), 2.49 (s, 3H), 1.43 (s, 9H).

^{13}C NMR (100 MHz, $CDCl_3$) δ 149.01, 134.14, 128.89, 128.83, 122.64, 119.81, 110.11, 96.56, 31.92, 30.41, 21.57.

HRMS (ESI) calcd for [M+H] $C_{13}H_{18}N$, m/z: 188.1434, found: 188.1428.

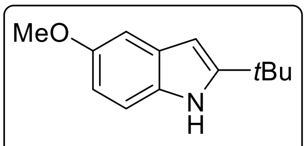

2d-3

Compound **2d-3** was prepared in 92% yield.

^{1}H NMR (400 MHz, $CDCl_3$) δ 7.93 (s, 1H), 7.23 (d, *J* = 8.0 Hz, 1H), 7.08 (s, 1H), 6.85-6.82 (m, 1H), 6.24-6.23 (m, 1H), 3.88 (s, 3H), 1.41 (s, 9H).

^{13}C NMR (100 MHz, $CDCl_3$) δ 154.16, 149.83, 131.00, 129.03, 111.17, 111.03, 102.29, 96.93, 56.08, 31.97, 30.40.

HRMS (ESI) calcd for [M+H] $C_{13}H_{18}ON$, m/z: 204.1383, found: 204.1377.

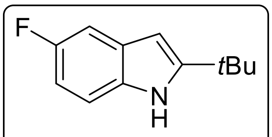

2d-4

Compound **2d-4** was prepared in 75% yield.

^{1}H NMR (400 MHz, $CDCl_3$) δ 7.95 (s, 1H), 7.24-7.19 (m, 2H), 6.91-6.86 (m, 1H), 6.25-6.24 (m, 1H), 1.38 (s, 9H).

^{13}C NMR (100 MHz, $CDCl_3$) δ 159.21, 156.89, 150.86, 132.34, 129.06, 128.96, 111.01, 110.91, 109.36, 109.10, 105.06, 104.83, 97.40, 97.35, 32.05, 30.36.

HRMS (ESI) calcd for [M+H] $C_{12}H_{15}FN$, m/z: 192.1183, found: 192.1177.

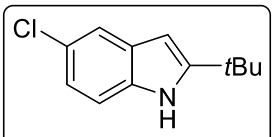

2d-5

Compound **2d-5** was prepared in 90% yield.

^{1}H NMR (400 MHz, $CDCl_3$) δ 7.98 (s, 1H), 7.54 (s, 1H), 7.23 (d, *J* = 8.0 Hz, 1H), 7.12-7.10 (m, 1H), 6.25-6.24 (m, 1H), 1.41 (s, 9H).

^{13}C NMR (100 MHz, $CDCl_3$) δ 150.47, 134.20, 129.76, 125.18, 121.30, 119.45, 111.45, 96.91,

31.99, 30.30.

HRMS (ESI) calcd for [M+H] $C_{12}H_{15}{}^{35}ClN$, m/z: 208.0893, found: 208.0894; $C_{12}H_{15}{}^{37}ClN$, m/z: 210.0864, found: 210.0868.

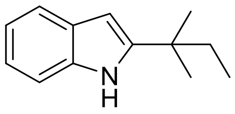

2d-6

Compound **2d-6** was prepared in 95% yield.

^{1}H NMR (400 MHz, $CDCl_3$) δ 7.93 (s, 1H), 7.62 (d, *J* = 8.0 Hz, 1H), 7.37 (d, *J* = 8.0 Hz, 1H), 7.20-7.11 (m, 2H), 6.32-6.31 (m, 1H), 1.72 (q, *J* = 8.0 Hz, 2H), 1.40 (s, 6H), 0.83 (t, *J* = 8.0 Hz, 3H).

^{13}C NMR (100 MHz, $CDCl_3$) δ 147.36, 135.90, 128.61, 121.05, 119.99, 119.59, 110.44, 98.46, 36.04, 35.40, 27.63, 9.28.

HRMS (ESI) calcd for [M+H] $C_{13}H_{18}N$, m/z: 188.1434, found: 188.1434.

Supplementary Note 3

General Experimental Procedures for the Syntheses of Racemates

General procedure for preparation of racemic compound 3a

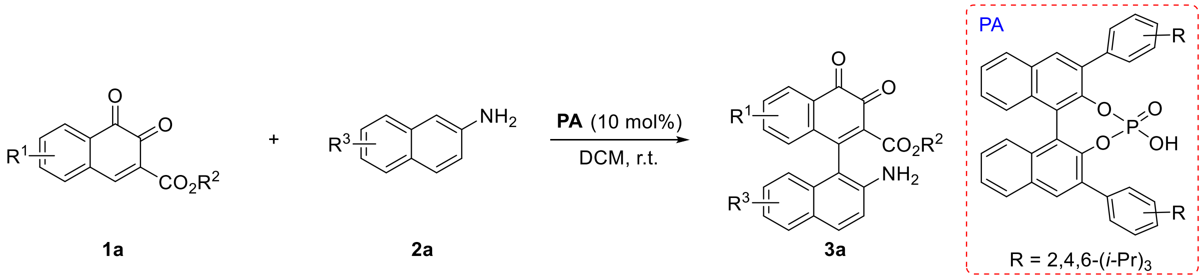

To a mixture of **CPA4** (10 mol%, *R*:*S*=1:1), *o*-naphthoquinone (0.22 mmol) and 2-naphthamine (0.10 mmol) in 10 mL Schlenk tube was added CH_2Cl_2 (2.0 mL) under Ar atmosphere. Then the reaction mixture was stirred vigorously at room temperature until the nucleophile was completely consumed (monitored by thin-layer chromatography). Typical reaction time was about 20 min. After that, the resulting mixture was concentrated under reduced pressure and purified by flash chromatography on silica gel (eluent: PE/EA = 4/1) to afford the corresponding racemic aryl *o*-naphthoquinones **3a**.

General procedure for preparation of racemic compound 3b-3d

To a mixture of diphenyl phosphonate (10 mol%), *o*-naphthoquinone (0.22 mmol) and nucleophile (0.10 mmol) in 10 mL Schlenk tube was added CH_2Cl_2 (2.0 mL) under Ar atmosphere. Then the reaction mixture was stirred vigorously at room temperature until the nucleophile was completely consumed (monitored by TLC). After that, the resulting mixture was concentrated under reduced pressure and purified by flash chromatography on silica gel to afford the corresponding racemic aryl *o*-naphthoquinones **3b-3d**.

General procedure for preparation of racemic compound 3e

To a mixture of diphenyl phosphonate (10 mol%) and indole **2d** (0.10 mmol) in 10 mL Schlenk tube was added CH_2Cl_2 (1.0 mL) under Ar atmosphere. Then the reaction mixture was stirred at 0 ℃ for 20 minutes. After that, a solution of *o*-naphthoquinone **1c** (0.20 mmol) in CH_2Cl_2 (1.0 mL) was added dropwise at 0 ℃. Then the reaction mixture was stirred for 24 h at 0 ℃. The resultant was concentrated under reduced pressure and purified by flash chromatography on silica gel (eluent: PE/DCM=1:2) to afford the corresponding racemic product **3e**.

Supplementary Note 4

General Experimental Procedures for the Syntheses of Chiral Compounds

General Procedure for Asymmetric Syntheses of Arylquinones (*R*)-3a

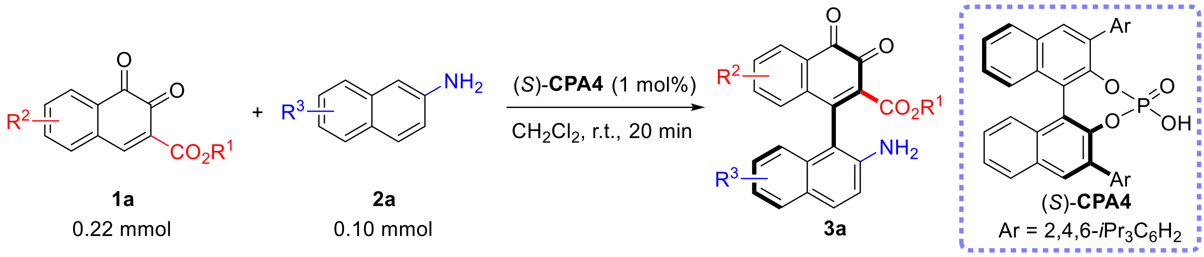

To a mixture of (*S*)-**CPA4** (1 mol%), *o*-naphthoquinone **1a** (0.44 mmol) and nucleophile **2a** (0.20 mmol) in 10 mL Schlenk tube was added CH_2Cl_2 (4.0 mL) under Ar atmosphere. Then the reaction mixture was stirred vigorously at room temperature until the nucleophile completely consumed (monitored by thin-layer chromatography). Typical reaction time was about 20 min. After that, the resulting mixture was concentrated under reduced pressure and purified by flash chromatography on silica gel (eluent: PE/EA = 4/1) to afford the corresponding axially chiral aryl *o*-naphthoquinones (*R*)-**3a**.

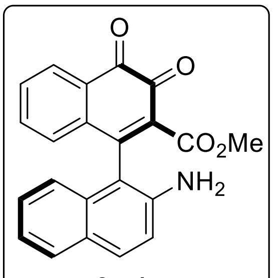

3a-1

(*R*)-Methyl 2'-amino-3,4-dioxo-3,4-dihydro-[1,1'-binaphthalene]-2-carboxylate ((*R*)-3a-1)

According to the general procedure, **(*R*)-3a-1** was obtained in **86% yield** with **99% ee**.

^{1}H NMR (400 MHz, DMSO-*d6*) δ 8.03 (dd, *J* = 7.6, 1.4 Hz, 1H), 7.66 (t, *J* = 9.3 Hz, 2H), 7.50 (td, *J* = 7.5, 1.2 Hz, 1H), 7.45-7.37 (m, 2H), 7.21-7.16 (m, 1H), 7.10-7.05 (m, 1H), 7.01 (d, *J* = 8.8 Hz, 1H), 6.58 (dd, *J* = 7.7, 1.1 Hz, 1H), 5.43 (s, 2H), 3.21 (s, 3H).

^{13}C NMR (125 MHz, $CDCl_3$) δ 177.98, 176.81, 164.34, 151.79, 141.26, 136.33, 135.57, 133.67, 131.95, 131.90, 131.66, 131.08, 130.62, 129.92, 128.20, 127.59, 127.48, 123.44, 123.11, 118.34, 110.81, 52.47.

HRMS (ESI) calcd for [M+H] $C_{22}H_{16}NO_4$, m/z: 358.1074, found: 358.1063.

HPLC analysis: CHIRALCEL IB, hexane/isopropanol = 60/40, 1.0 mL/min, λ = 254 nm, t_R (major) = 8.9 min, t_R (minor) = 21.4 min, ee = 99%.

Supplementary Figure 1. Chiral HPLC spectrum of racemic **3a-1**

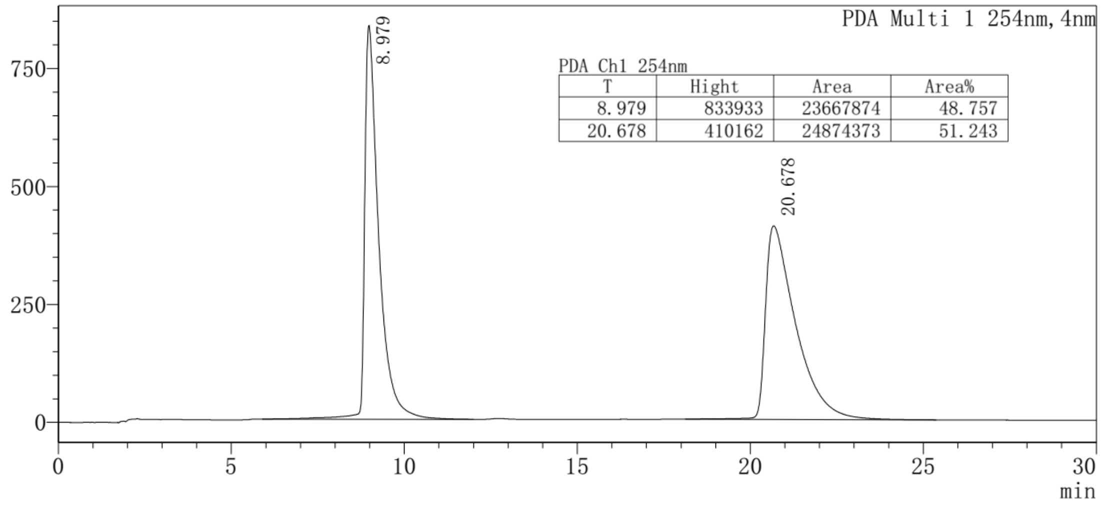

Supplementary Figure 2. Chiral HPLC spectrum of **(*R*)-3a-1**

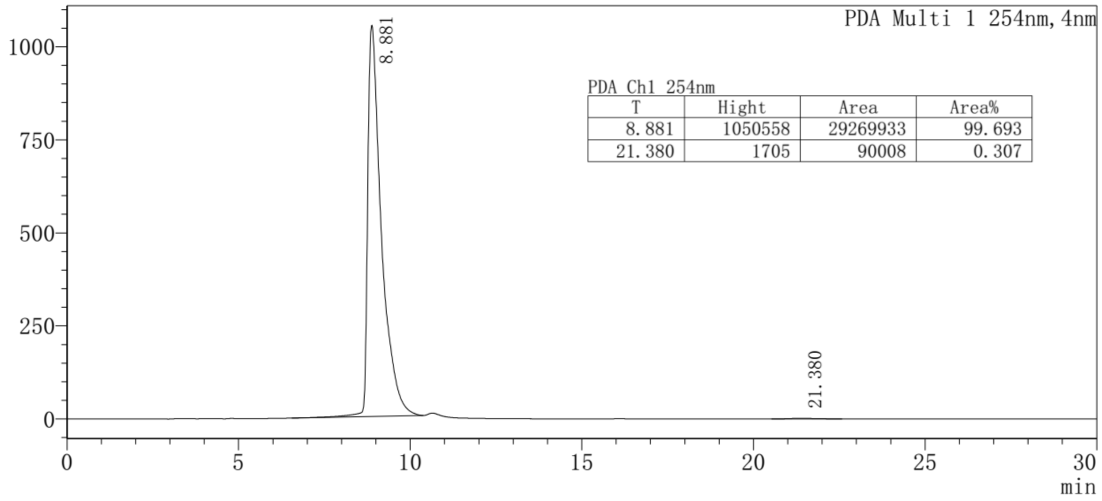

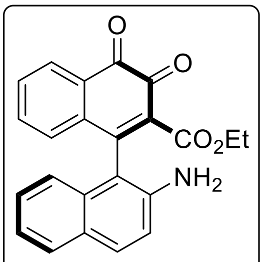

3a-2

(*R*)-Ethyl 2'-amino-3,4-dioxo-3,4-dihydro-[1,1'-binaphthalene]-2-carboxylate ((*R*)-3a-2)

According to the general procedure, **(*R*)-3a-2** was obtained in **83% yield** with **97% ee**.

^{1}H NMR (500 MHz, $CDCl_3$) δ 8.24 (dd, J = 7.6, 1.4 Hz, 1H), 7.79 (d, J = 8.8 Hz, 1H), 7.75 (d, J = 8.0 Hz, 1H), 7.55 (td, J = 7.6, 1.2 Hz, 1H), 7.47-7.41 (m, 2H), 7.37-7.33 (m, 1H), 7.30-7.26 (m, 1H), 7.04 (d, J = 8.8 Hz, 1H), 6.88 (d, J = 7.8 Hz, 1H), 4.03 (s, 2H), 3.95-3.75 (m, 2H), 0.55 (t, J = 7.1 Hz, 3H).

^{13}C NMR (125 MHz, $CDCl_3$) δ 178.19, 176.92, 163.81, 151.64, 141.45, 136.42, 135.78, 133.82, 132.14, 131.99, 131.73, 131.06, 130.69, 129.91, 128.22, 127.63, 127.48, 123.71, 123.13, 118.42, 110.85, 61.56, 13.27.

HRMS (ESI) calcd for [M+H] $C_{23}H_{18}NO_4$, m/z: 372.1230, found: 372.1227.

HPLC analysis: CHIRALCEL AD-H, hexane/isopropanol = 70/30, 1.0 mL/min, λ = 254 nm, t_R (major) = 10.7 min, t_R (minor) = 32.6 min, ee = 97%.

Supplementary Figure 3. Chiral HPLC spectrum of racemic **3a-2**

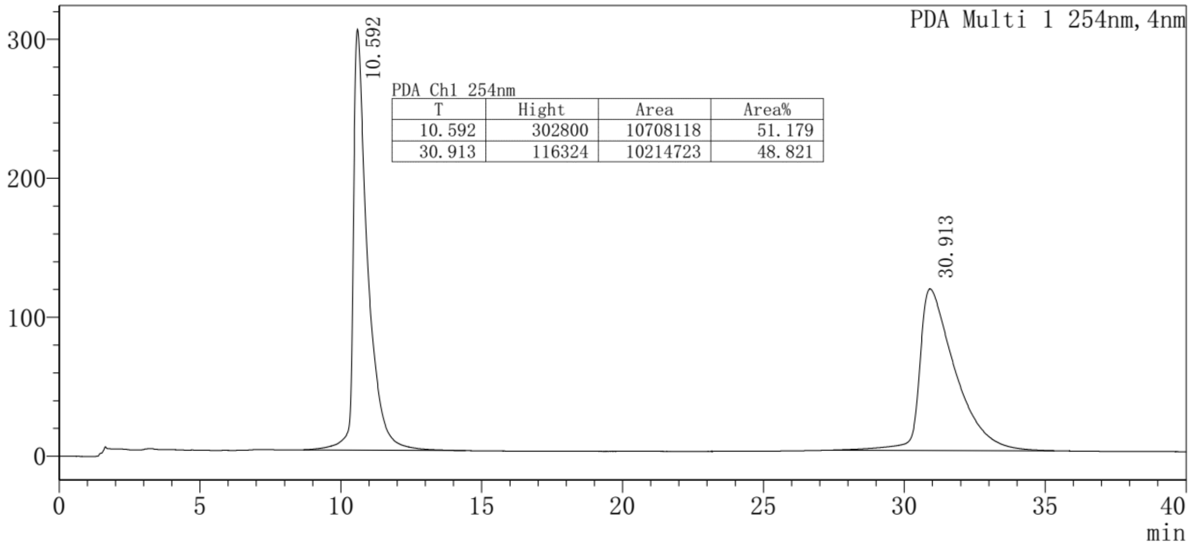

Supplementary Figure 4. Chiral HPLC spectrum of **(*R*)-3a-2**

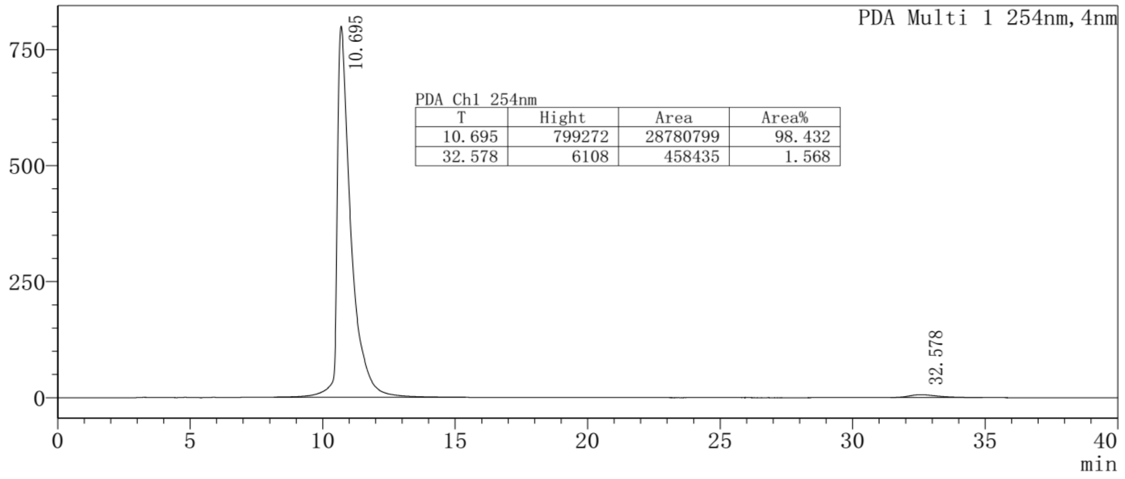

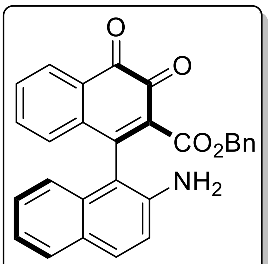

3a-3

(*R*)-Benzyl 2’-amino-3,4-dioxo-3,4-dihydro-[1,1’-binaphthalene]-2-carboxylate ((*R*)-3a-3)

According to the general procedure, **(*R*)-3a-3** was obtained in **82% yield** with **93% ee**.

^{1}H NMR (500 MHz, $CDCl_3$) δ 8.23 (dd, *J* = 7.6, 1.4 Hz, 1H), 7.77-7.73 (m, 2H), 7.53 (td, *J* = 7.5, 1.2 Hz, 1H), 7.43 (td, *J* = 7.7, 1.4 Hz, 1H), 7.39 (dd, *J* = 8.1, 1.7 Hz, 1H), 7.32-7.26 (m, 2H), 7.20-7.16 (m, 1H), 7.09 (t, *J* = 7.7 Hz, 2H), 6.94 (d, *J* = 8.8 Hz, 1H), 6.83 (dd, *J* = 7.8, 1.2 Hz, 1H), 6.70-6.68 (m, 2H), 4.88-4.81 (m, 2H), 3.78 (s, 2H).

^{13}C NMR (125 MHz, $CDCl_3$) δ 178.08, 176.86, 163.80, 151.83, 141.45, 136.41, 135.46, 134.53, 133.81, 132.10, 132.03, 131.73, 131.13, 130.69, 130.01, 128.36, 128.34, 128.12, 128.08, 127.65, 127.64, 123.63, 123.17, 118.54, 110.67, 67.53.

HRMS (ESI) calcd for [M+H] $C_{28}H_{20}NO_4$, m/z: 434.1387, found: 434.1385.

HPLC analysis: CHIRALCEL IA, hexane/isopropanol = 60/40, 1.0 mL/min, λ = 254 nm, t_R (minor) = 15.0 min, t_R (major) = 26.9 min, ee = 93%.

Supplementary Figure 5. Chiral HPLC spectrum of racemic **3a-3**

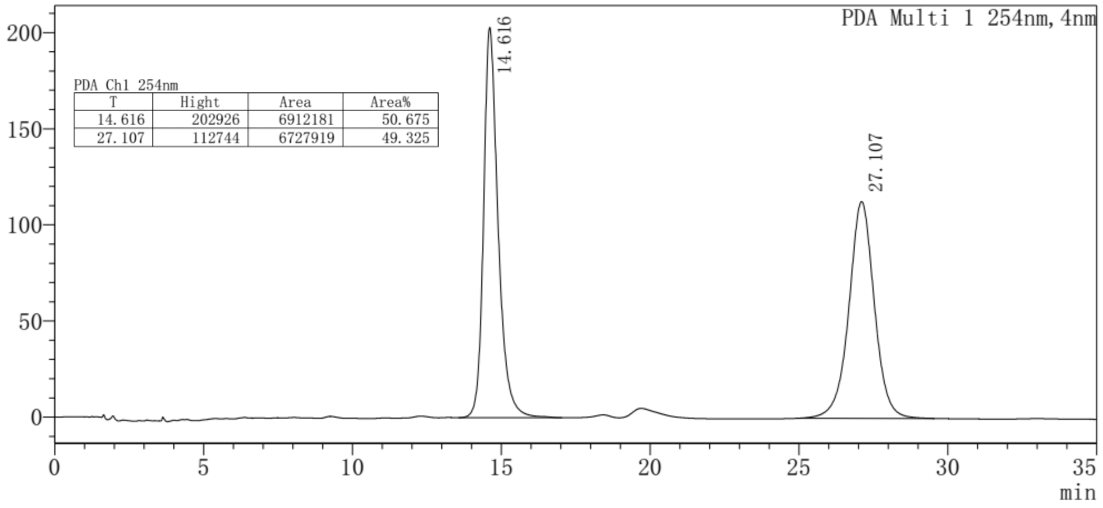

Supplementary Figure 6. Chiral HPLC spectrum of **(*R*)-3a-3**

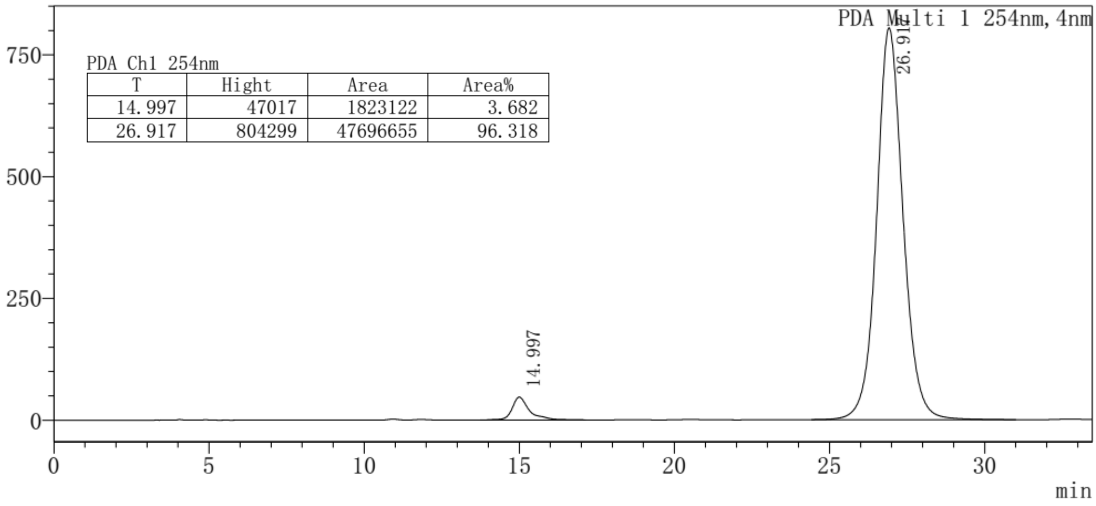

T	Hight	Area	Area%
14.997	47017	1823122	3.682
26.917	804299	47696655	96.318

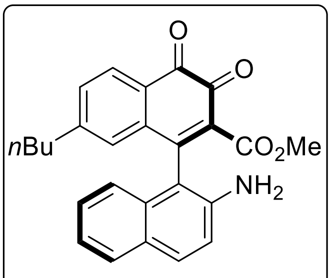

3a-4

(*R*)-Methyl 2'-amino-7-butyl-3,4-dioxo-3,4-dihydro-[1,1'-binaphthalene]-2-carboxylate ((*R*)-3a-4)

According to the general procedure, **(*R*)-3a-4** was obtained in **76% yield** with **98% ee**.

^{1}H NMR (400 MHz, $CDCl_3$) δ 8.15 (d, *J* = 7.8 Hz, 1H), 7.79 (d, *J* = 8.8 Hz, 1H), 7.76 (d, *J* = 8.0 Hz, 1H), 7.40 (d, *J* = 8.3 Hz, 1H), 7.37-7.32 (m, 2H), 7.30-7.25 (m, 1H), 7.06 (d, *J* = 8.8 Hz, 1H), 6.61 (d, *J* = 1.7 Hz, 1H), 4.04 (s, 2H), 3.37 (s, 3H), 2.44-2.39 (m, 2H), 1.38-1.30 (m, 2H), 1.15-1.08 (m, 2H), 0.74 (t, *J* = 7.3 Hz, 3H).

^{13}C NMR (100 MHz, $CDCl_3$) δ 177.78, 177.31, 164.55, 152.87, 152.10, 141.44, 135.54, 133.69, 132.13, 131.92, 131.07, 131.04, 130.19, 129.65, 128.20, 127.60, 127.42, 123.63, 123.05, 118.43, 110.87, 52.45, 35.79, 32.54, 22.00, 13.74.

HRMS (ESI) calcd for [M+H] $C_{26}H_{24}NO_4$, m/z: 414.1700, found: 414.1696.

HPLC analysis: CHIRALCEL IA, hexane/isopropanol = 70/30, 1.0 mL/min, λ = 254 nm, t_R (minor) = 16.1 min, t_R (major) = 21.1 min, ee = 98%.

Supplementary Figure 7. Chiral HPLC spectrum of racemic **3a-4**

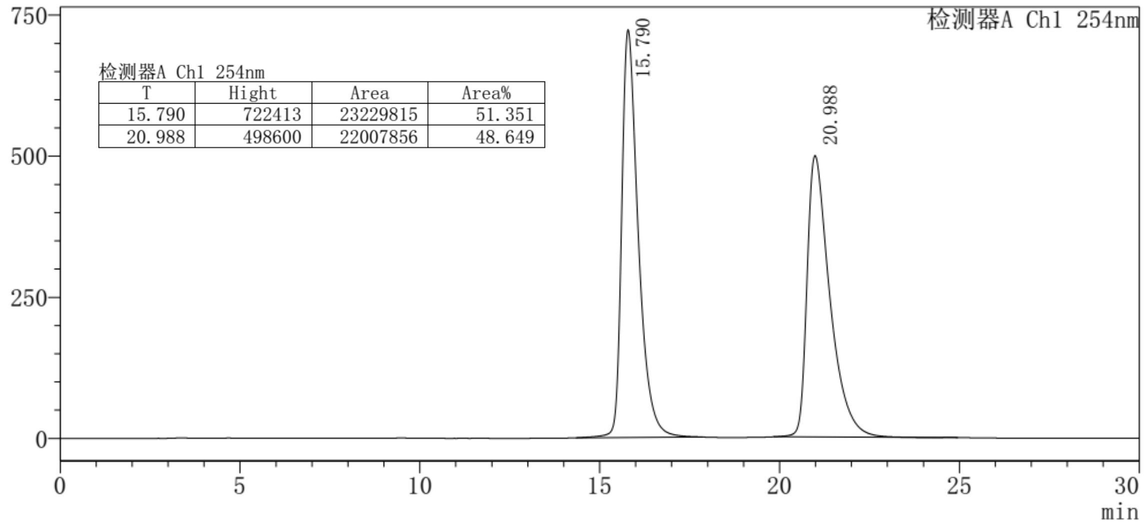

Supplementary Figure 8. Chiral HPLC spectrum of **(*R*)-3a-4**

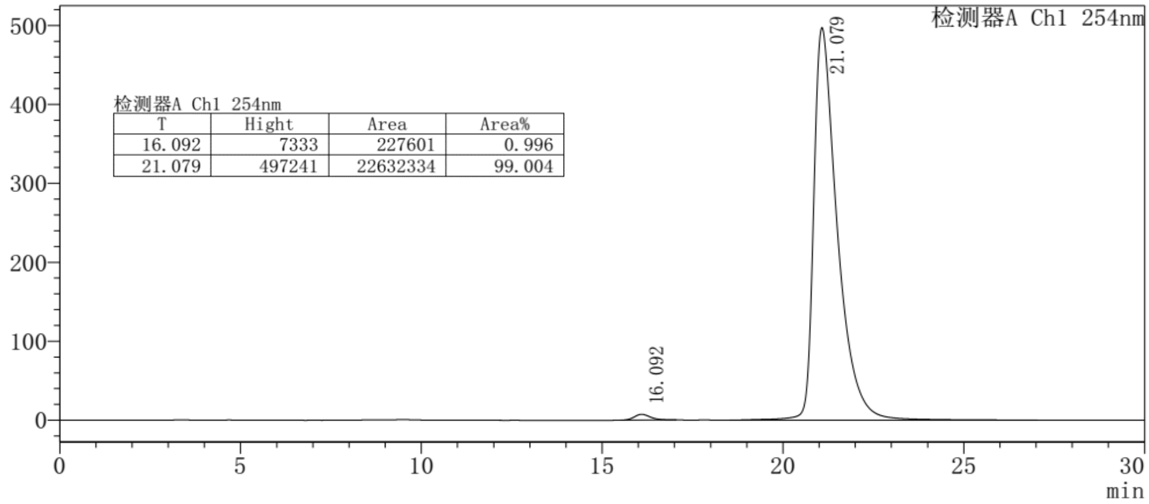

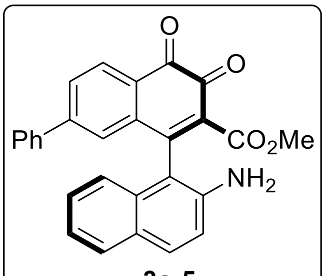

3a-5

(*R*)-Methyl 2'-amino-3,4-dioxo-7-phenyl-3,4-dihydro-[1,1'-binaphthalene]-2-carboxylate ((*R*)-3a-5)

According to the general procedure, **(*R*)-3a-5** was obtained in **80% yield** with **99% ee**.

^{1}H NMR (400 MHz, $CDCl_3$) δ 8.33 (d, *J* = 7.9 Hz, 1H), 7.82 (d, *J* = 8.8 Hz, 1H), 7.77 (td, *J* = 7.8, 1.4 Hz, 2H), 7.49 (d, *J* = 8.4 Hz, 1H), 7.41-7.36 (m, 1H), 7.35-7.32 (m, 3H), 7.32-7.25 (m, 3H), 7.10-7.07 (m, 2H), 4.10 (s, 2H), 3.41 (s, 3H).

^{13}C NMR (100 MHz, $CDCl_3$) δ 177.79, 177.15, 164.47, 151.80, 149.12, 141.53, 138.62, 135.96, 134.28, 132.08, 131.40, 131.28, 130.33, 130.23, 129.15, 129.12, 128.64, 128.32, 127.64, 127.55, 127.21, 123.53, 123.13, 118.42, 110.63, 52.51.

HRMS (ESI) calcd for [M+H] $C_{28}H_{20}NO_4$, m/z: 434.1387, found: 434.1392.

HPLC analysis: CHIRALCEL IA, hexane/isopropanol = 70/30, 1.0 mL/min, λ = 254 nm, t_R (minor) = 18.3 min, t_R (major) = 22.2 min, ee = 99%.

Supplementary Figure 9. Chiral HPLC spectrum of racemic **3a-5**

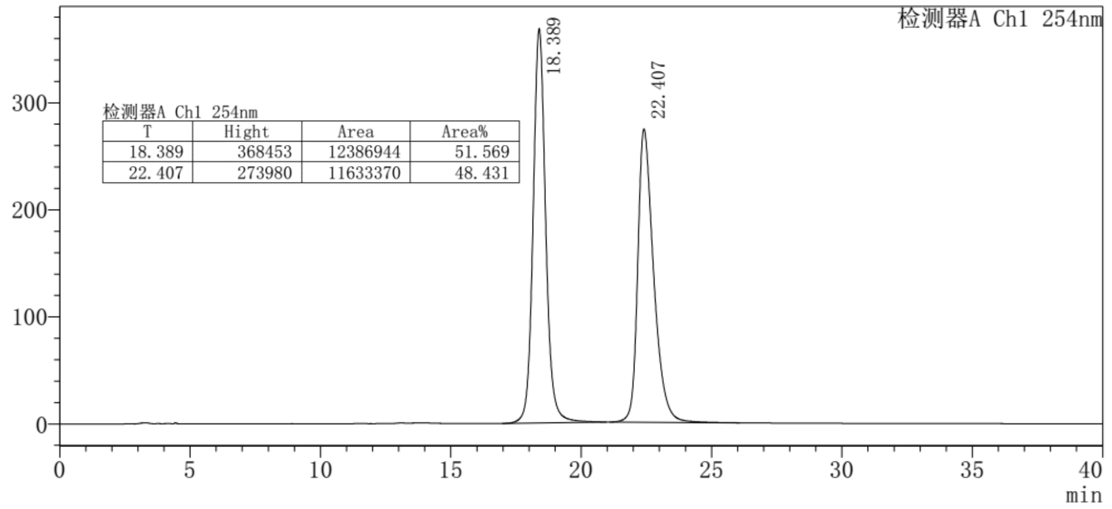

Supplementary Figure 10. Chiral HPLC spectrum of **(*R*)-3a-5**

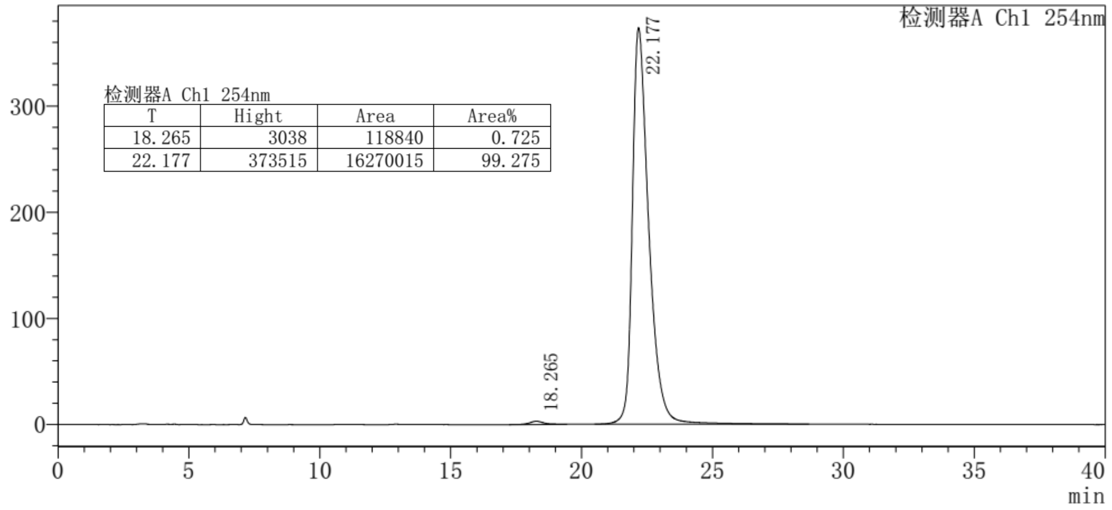

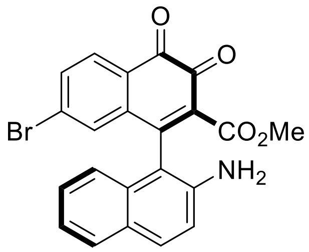

3a-6

(*R*)-Methyl 2’-amino-7-bromo-3,4-dioxo-3,4-dihydro-[1,1’-binaphthalene]-2-carboxylate ((*R*)-3a-6)

According to the general procedure, **(*R*)-3a-6** was obtained in **80% yield** with **96% ee**.

^{1}H NMR (500 MHz, CDCl$_3$) δ 8.09 (d, *J* = 8.2 Hz, 1H), 7.81 (d, *J* = 8.8 Hz, 1H), 7.77 (d, *J* = 8.0 Hz, 1H), 7.70 (dd, *J* = 8.2, 1.8 Hz, 1H), 7.46-7.35 (m, 2H), 7.33-7.29 (m, 1H), 7.05 (d, *J* = 8.7 Hz, 1H), 6.94 (d, *J* = 1.8 Hz, 1H), 3.98 (s, 2H), 3.39 (s, 3H).

^{13}C NMR (125 MHz, CDCl$_3$) δ 177.33, 176.41, 164.09, 150.58, 141.47, 136.83, 135.35, 135.17, 132.57, 132.45, 131.91, 131.79, 131.60, 130.37, 128.47, 127.80, 127.78, 123.38, 123.28, 118.51, 110.07, 52.68.

HRMS (ESI) calcd for [M+H] $C_{22}H_{15}NO_4{}^{79}Br$, m/z: 436.0179, found: 436.0186; $C_{22}H_{15}NO_4{}^{81}Br$, m/z: 438.0159, found: 438.0160.

HPLC analysis: CHIRALCEL IA, hexane/isopropanol = 50/50, 1.0 mL/min, λ = 254 nm, t_R (minor) = 19.0 min, t_R (major) = 37.6 min, ee = 96%.

Supplementary Figure 11. Chiral HPLC spectrum of racemic **3a-6**

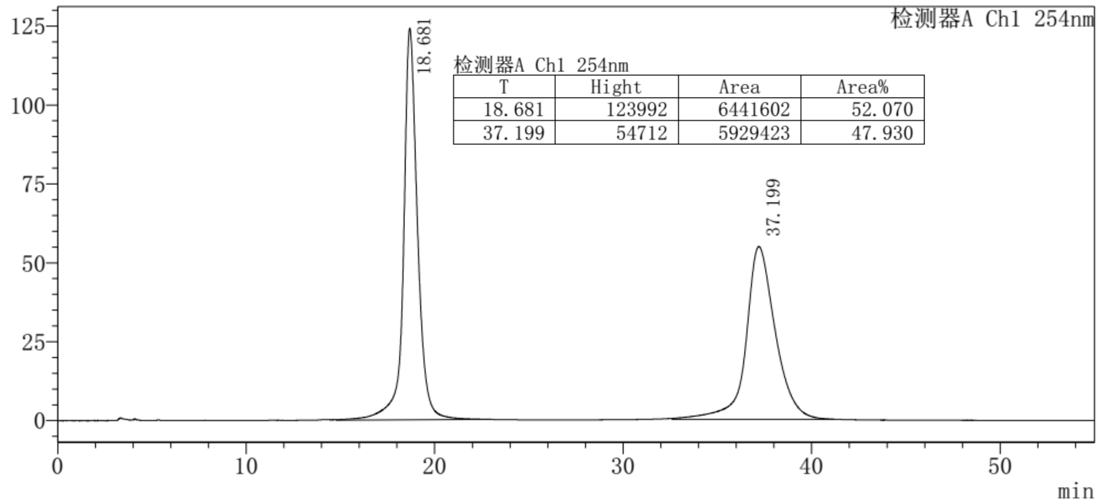

Supplementary Figure 12. Chiral HPLC spectrum of **(*R*)-3a-6**

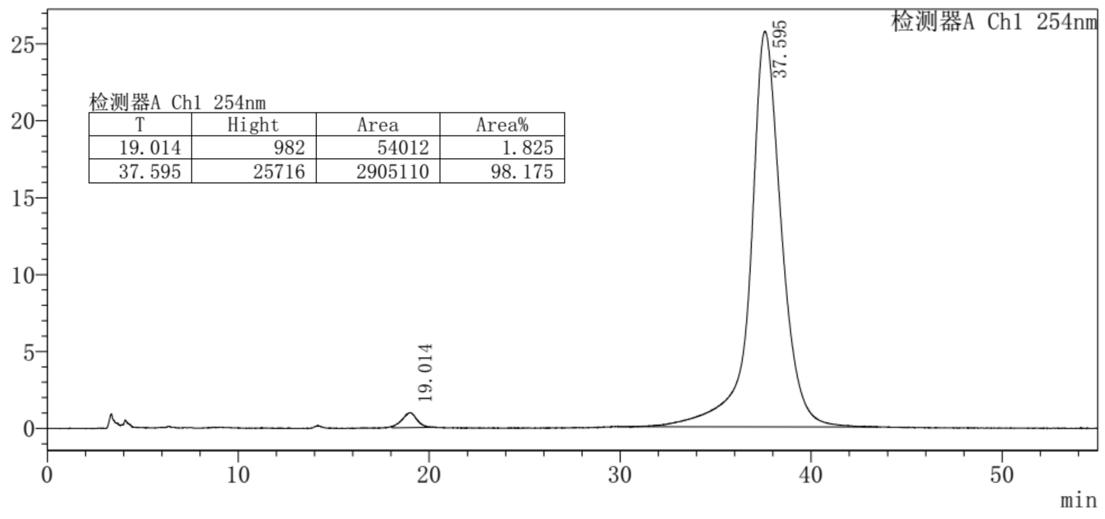

检测器A Ch1 254nm

T	Hight	Area	Area%
19.014	982	54012	1.825
37.595	25716	2905110	98.175

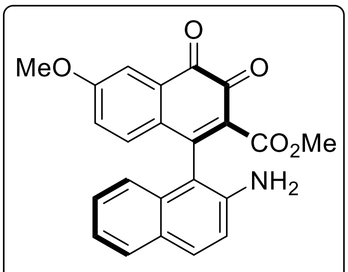

3a-7

(*R*)-Methyl

2'-amino-6-methoxy-3,4-dioxo-3,4-dihydro-[1,1'-binaphthalene]-2-carboxylate

((*R*)-3a-7)

According to the general procedure, **(*R*)-3a-7** was obtained in **70% yield** with **96% ee**.

^{1}H NMR (400 MHz, $CDCl_3$) δ 8.22 (d, *J* = 8.6 Hz, 1H), 7.77 (d, *J* = 8.7 Hz, 1H), 7.73 (d, *J* = 8.0 Hz, 1H), 7.41 (dd, *J* = 8.4, 1.2 Hz, 1H), 7.37-7.32 (m, 1H), 7.39 - 7.24 (m, 1H), 7.03 (d, *J* = 8.8 Hz, 1H), 6.96 (dd, *J* = 8.6, 2.5 Hz, 1H), 6.32 (d, *J* = 2.5 Hz, 1H), 4.06 (s, 2H), 3.65 (s, 3H), 3.37 (s, 3H).

^{13}C NMR (100 MHz, $CDCl_3$) δ 177.59, 176.46, 166.13, 164.46, 151.13, 141.54, 136.21, 136.00, 133.86, 132.10, 131.13, 128.22, 127.56, 127.47, 125.06, 123.50, 123.03, 118.39, 117.11, 115.04, 110.56, 55.81, 52.45.

HRMS (ESI) calcd for [M+H] $C_{23}H_{18}NO_5$, m/z: 388.1180, found: 388.1176.

HPLC analysis: CHIRALCEL IA, hexane/isopropanol = 50/50, 1.0 mL/min, λ = 254 nm, t_R (minor) = 16.2 min, t_R (major) = 24.2 min, ee = 96%.

Supplementary Figure 13. Chiral HPLC spectrum of racemic **3a-7**

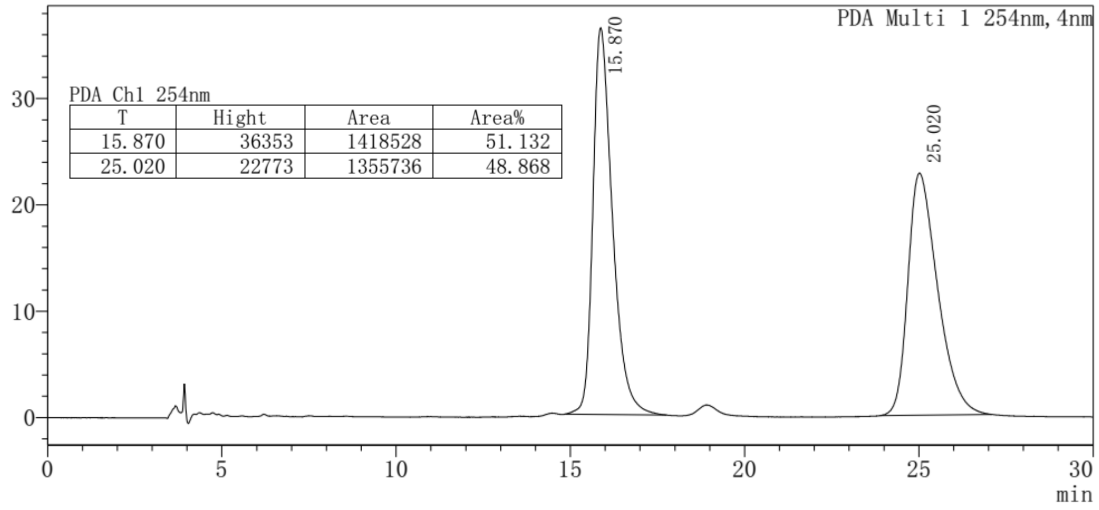

Supplementary Figure 14. Chiral HPLC spectrum of **(*R*)-3a-7**

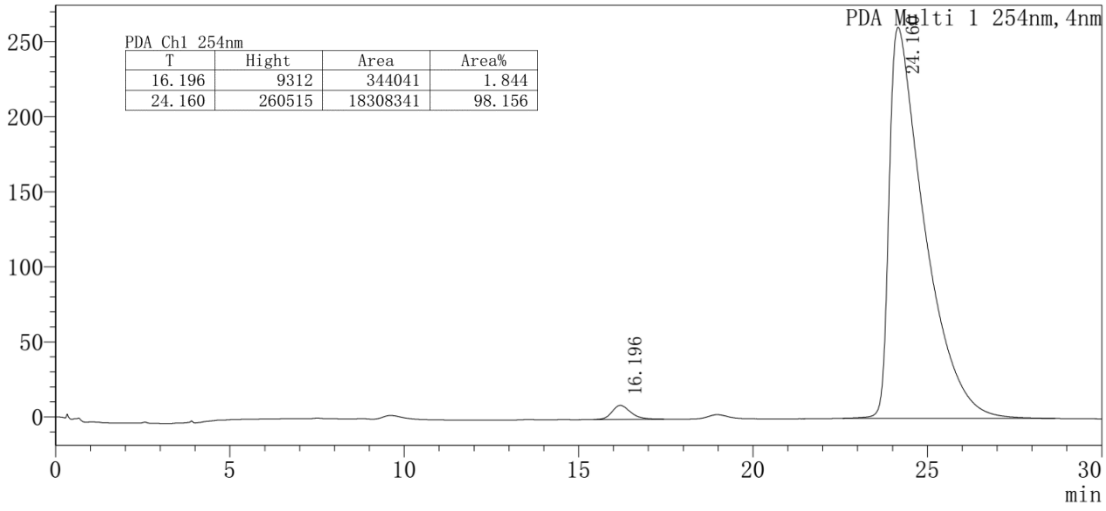

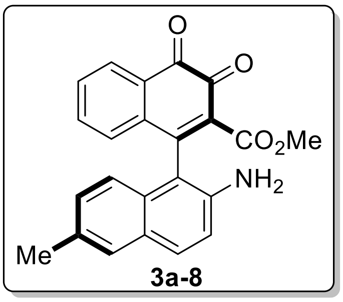

(*R*)-Methyl

2'-amino-6'-methyl-3,4-dioxo-3,4-dihydro-[1,1'-binaphthalene]-2-carboxylate ((*R*)-3a-8)

According to the general procedure, **(*R*)-3a-8** was obtained in **73% yield** with **98% ee**.

^{1}H NMR (500 MHz, Acetone-d_6) δ 8.16 (dd, J = 7.6, 1.3 Hz, 1H), 7.69 (d, J = 8.8 Hz, 1H), 7.63-7.59 (m, 1H), 7.54-7.51 (m, 2H), 7.49 (d, J = 8.6 Hz, 1H), 7.12 (dd, J = 8.8, 1.3 Hz, 2H), 6.81 (dd, J = 7.8, 1.1 Hz, 1H), 5.05 (s, 2H), 3.32 (s, 3H), 2.39 (s, 3H).

^{13}C NMR (125 MHz, Acetone-*d*$_6$) δ 178.95, 177.72, 165.14, 151.32, 143.27, 137.02, 136.08, 135.44, 133.67, 132.00, 131.79, 131.70, 130.48, 130.09, 129.85, 129.56, 127.92, 127.86, 124.52, 119.18, 110.19, 51.92, 21.22.

HRMS (ESI) calcd for [M+H] $C_{23}H_{17}NO_4$, m/z: 372.1230, found: 372.1228.

HPLC analysis: CHIRALCEL IA, hexane/isopropanol = 50/50, 1.0 mL/min, λ = 254 nm, t_R (minor) = 13.3 min, t_R (major) = 26.8 min, ee = 98%.

Supplementary Figure 15. Chiral HPLC spectrum of racemic **3a-8**

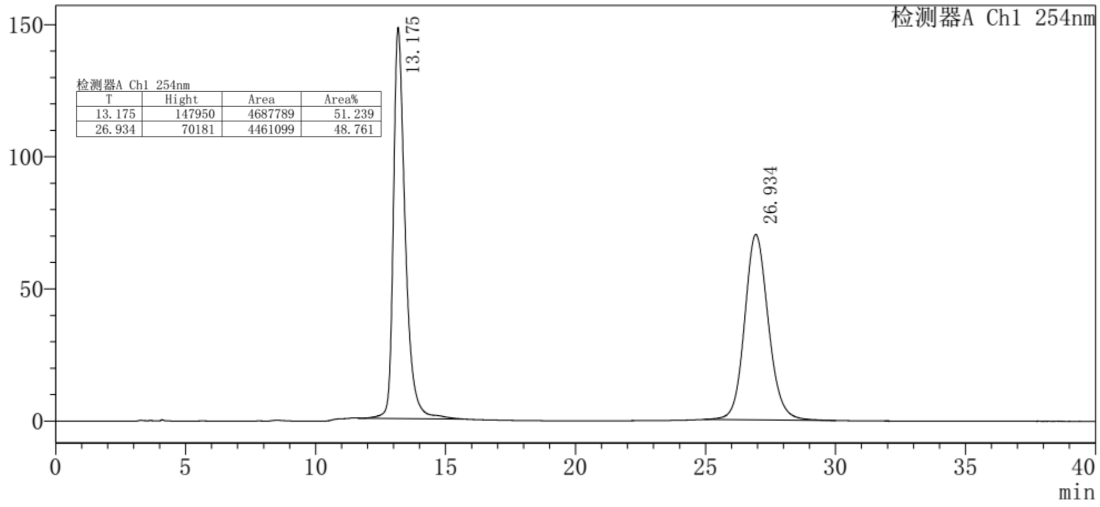

Supplementary Figure 16. Chiral HPLC spectrum of **(*R*)-3a-8**

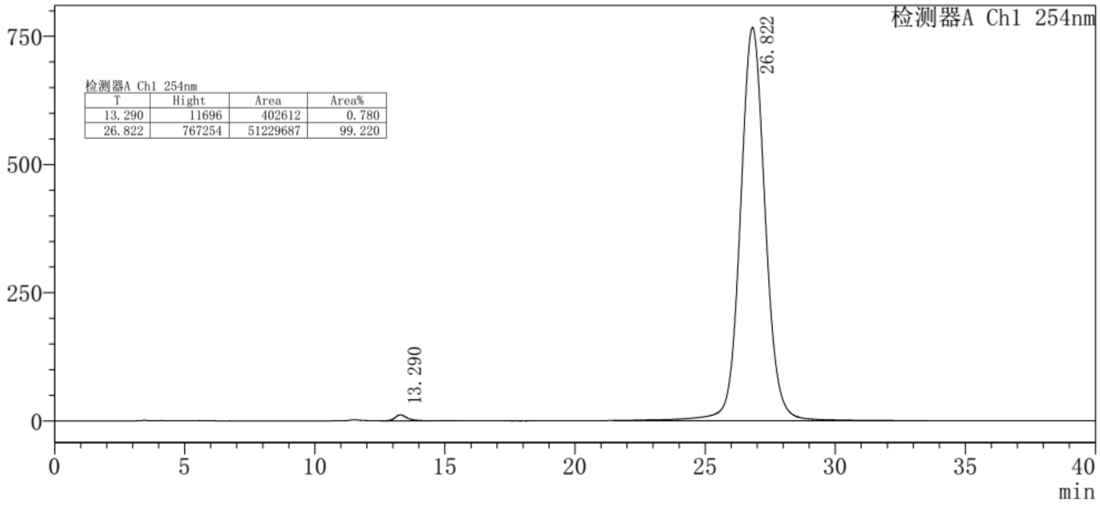

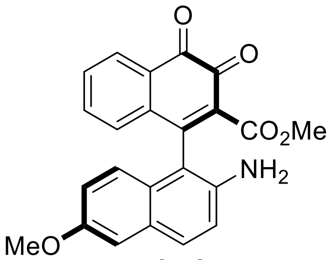

3a-9

(*R*)-Methyl

2’-amino-6’-methoxy-3,4-dioxo-3,4-dihydro-[1,1’-binaphthalene]-2-carboxylate

((*R*)-3a-9)

According to the general procedure, **(*R*)-3a-9** was obtained in **75% yield** with **99% ee**.

^{1}H NMR (400 MHz, CDCl$_3$) δ 8.23 (d, *J* = 7.6 Hz, 1H), 7.70 (d, *J* = 8.8 Hz, 1H), 7.54 (t, *J* = 7.6 Hz, 1H), 7.44 (td, *J* = 7.6, 1.3 Hz, 1H), 7.33 (d, *J* = 9.1 Hz, 1H), 7.10 (d, *J* = 2.6 Hz, 1H), 7.06-7.02 (m, 2H), 6.82 (d, *J* = 7.8 Hz, 1H), 3.89 (s, 3H), 3.42 (s, 3H).

^{13}C NMR (100 MHz, CDCl$_3$) δ 178.10, 176.90, 164.47, 155.84, 151.96, 139.58, 136.41, 135.60, 133.87, 132.04, 131.79, 130.70, 130.07, 129.94, 128.78, 127.23, 125.12, 119.80, 119.15, 111.77, 107.01, 55.46, 52.59.

HRMS (ESI) calcd for [M+H] $C_{23}H_{17}NO_5$, m/z: 388.1179, found: 388.1175.

HPLC analysis: CHIRALCEL IA, hexane/isopropanol = 70/30, 1.0 mL/min, λ = 254 nm, t_R (minor) = 25.4 min, t_R (major) = 38.7 min, ee = 99%.

Supplementary Figure 17. Chiral HPLC spectrum of racemic **3a-9**

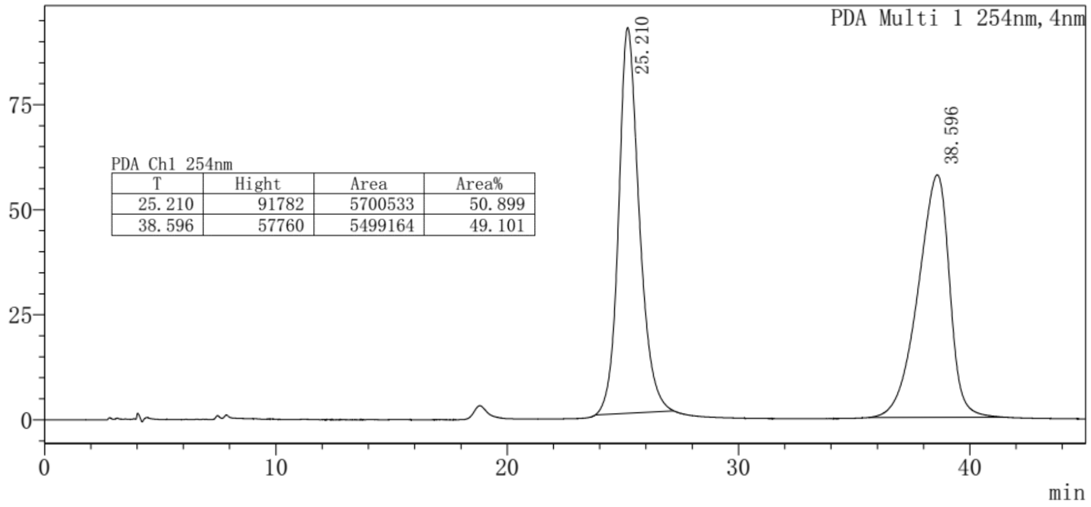

Supplementary Figure 18. Chiral HPLC spectrum of **(*R*)-3a-9**

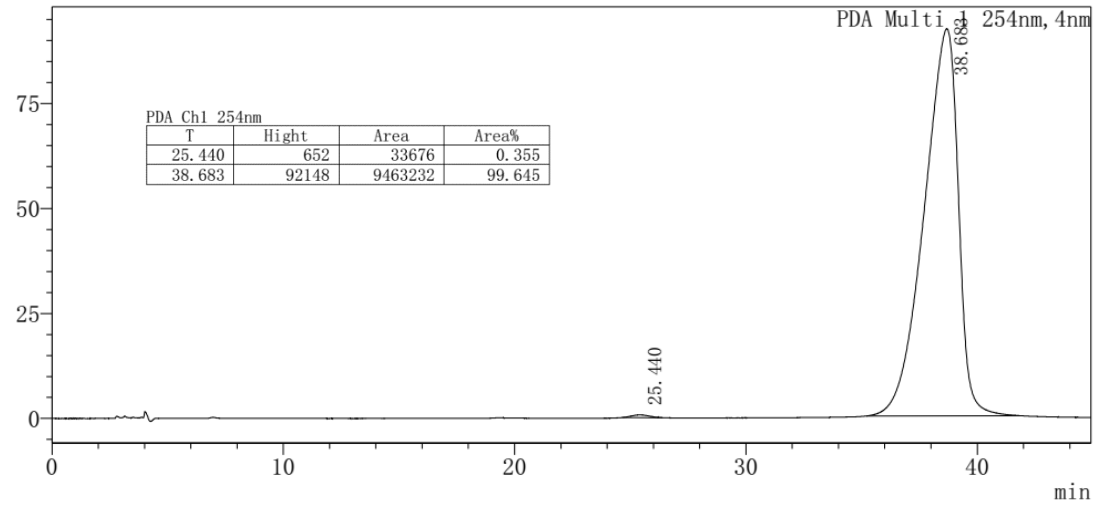

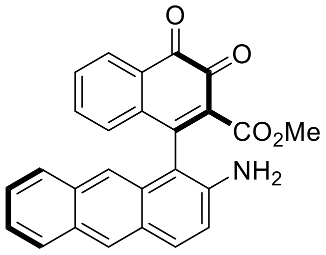

3a-10

(*R*)-Methyl 1-(2-aminoanthracen-1-yl)-3,4-dioxo-3,4-dihydronaphthalene-2-carboxylate ((*R*)-3a-10)

According to the general procedure, **(*R*)-3a-10** was obtained in **87% yield** with **95% ee**.

^{1}H NMR (500 MHz, Acetone-*d*$_6$) δ 8.37 (s, 1H), 8.18 (dd, *J* = 7.7, 1.4 Hz, 1H), 8.16 (s, 1H), 7.99 (d, *J* = 9.1 Hz, 1H), 7.96-7.94 (m, 1H), 7.81-7.71 (m, 1H), 7.60 (td, *J* = 7.6, 1.2 Hz, 1H), 7.48 (td, *J* = 7.7, 1.5 Hz, 1H), 7.35-7.30 (m, 2H), 7.24 (d, *J* = 9.1 Hz, 1H), 6.91 (dd, *J* = 7.8, 1.1 Hz, 1H), 5.37 (s, 2H), 3.28 (s, 3H).

^{13}C NMR (125 MHz, Acetone-*d*$_6$) δ 179.08, 177.87, 165.19, 151.40, 143.30, 137.41, 136.17, 135.42, 133.80, 133.64, 132.13, 132.03, 131.64, 130.17, 130.11, 129.91, 128.91, 128.43, 127.57, 127.36, 126.28, 124.65, 121.54, 121.07, 107.20, 51.90.

HRMS (ESI) calcd for [M+H] $C_{26}H_{17}NO_4$, m/z: 408.1230, found: 408.1230.

HPLC analysis: CHIRALCEL IA, hexane/isopropanol = 50/50, 1.0 mL/min, λ = 254 nm, t_R (minor) = 11.7 min, t_R (major) = 21.9 min, ee = 95%.

Supplementary Figure 19. Chiral HPLC spectrum of racemic **3a-10**

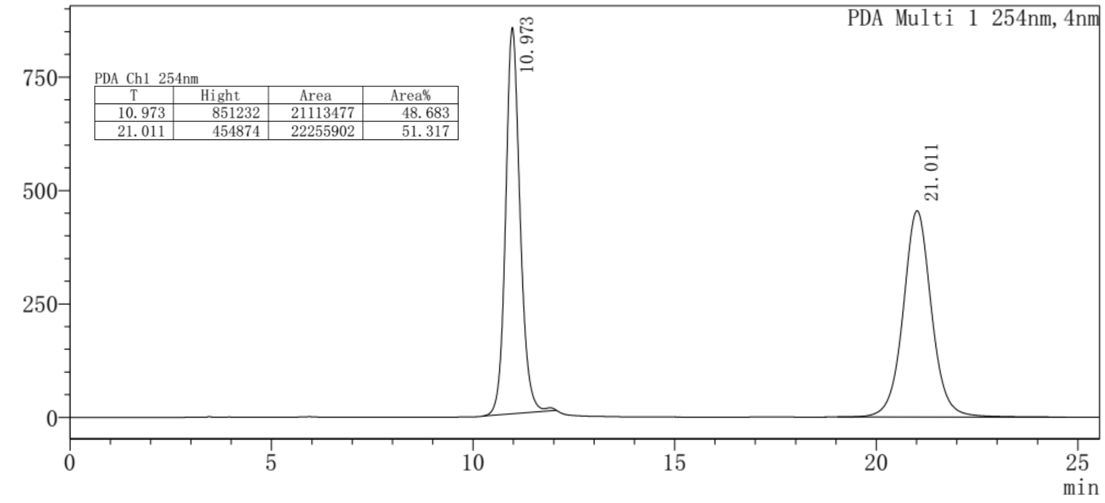

Supplementary Figure 20. Chiral HPLC spectrum of **(*R*)-3a-10**

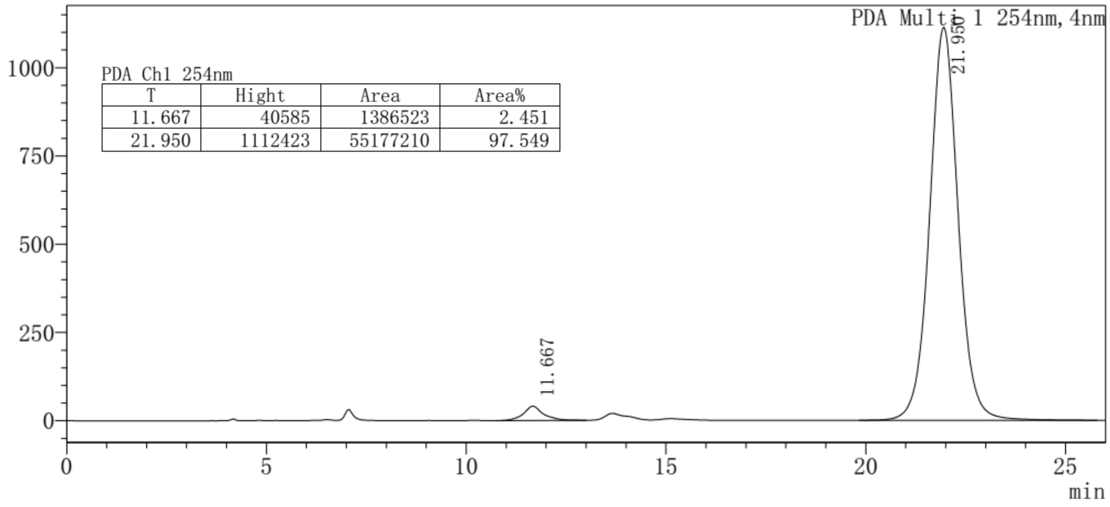

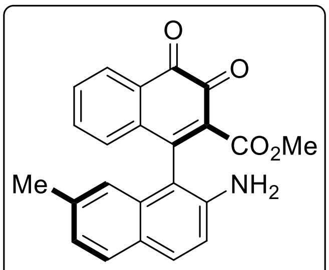

3a-11

(*R*)-Methyl 2'-amino-7'-methyl-3,4-dioxo-3,4-dihydro-[1,1'-binaphthalene]-2-carboxylate ((*R*)-3a-11)

According to the general procedure, **(*R*)-3a-9** was obtained in **72% yield** with **99% ee**.

^{1}H NMR (500 MHz, $CDCl_3$) δ 8.17 (d, *J* = 7.3 Hz, 1H), 7.80 (d, *J* = 8.0 Hz, 1H), 7.70 (d, *J* = 8.4 Hz, 1H), 7.52 (t, *J* = 7.5 Hz, 1H), 7.42 (t, *J* = 7.5 Hz, 1H), 7.24-7.19 (m, 3H), 6.82 (d, *J* = 7.5

Hz, 1H), 5.01 (s, 2H), 3.35 (s, 3H), 2.38 (s, 3H).

^{13}C NMR (125 MHz, $CDCl_3$) δ 178.12, 176.90, 164.60, 151.36, 137.81, 136.66, 135.08, 133.70, 132.22, 131.80, 131.59, 131.09, 130.83, 130.77, 128.24, 127.50, 126.88, 123.12, 118.87, 52.80, 22.13.

HRMS (ESI) calcd for [M+H] $C_{23}H_{17}NO_4$, m/z: 372.1230, found: 372.1229.

HPLC analysis: CHIRALCEL IA, hexane/isopropanol = 60/40, 1.0 mL/min, λ = 254 nm, t_R (minor) = 11.5 min, t_R (major) = 24.5 min, ee = 99%.

Supplementary Figure 21. Chiral HPLC spectrum of racemic **3a-11**

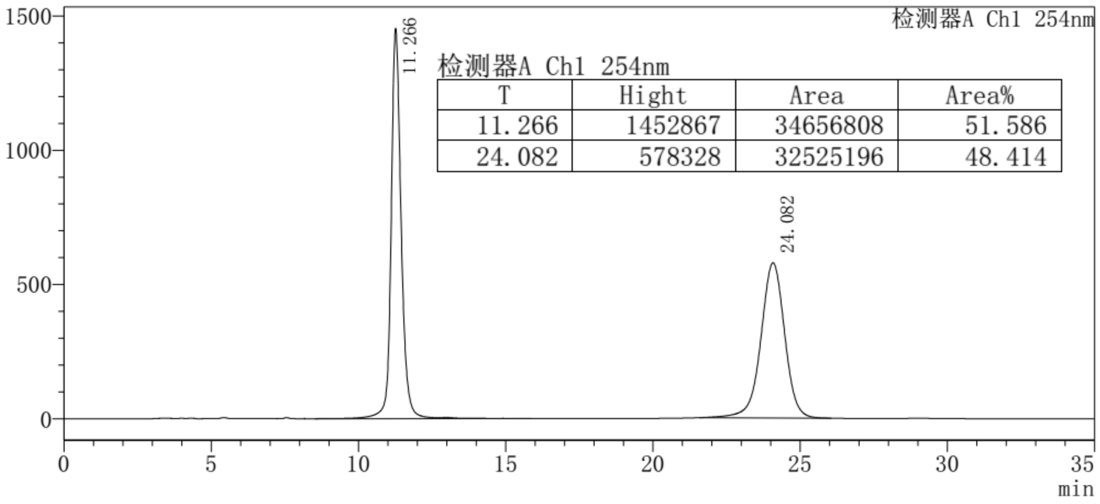

Supplementary Figure 22. Chiral HPLC spectrum of **(*R*)-3a-11**

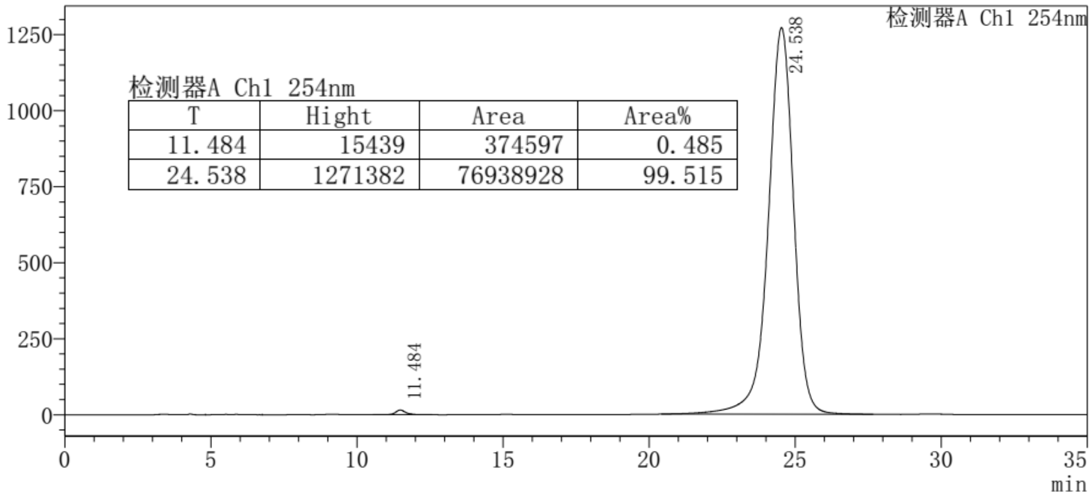

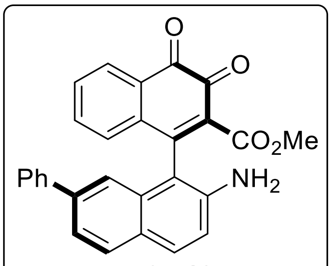

3a-12

(*R*)-Methyl

2’-amino-3,4-dioxo-7’-phenyl-3,4-dihydro-[1,1’-binaphthalene]-2-carboxylate

((*R*)-3a-12)

According to the general procedure, **(*R*)-3a-12** was obtained in **75% yield** with **99% ee**.

^{1}H NMR (500 MHz, $CDCl_3$) δ 8.25 (dd, *J* = 7.6, 1.4 Hz, 1H), 7.97 (d, *J* = 1.8 Hz, 1H), 7.85 (d, *J* = 8.7 Hz, 1H), 7.67 (dd, *J* = 8.2, 1.3 Hz, 2H), 7.63 (dd, *J* = 8.7, 2.0 Hz, 1H), 7.56 (td, *J* = 7.6, 1.2 Hz, 1H), 7.49-7.44 (m, 4H), 7.37-7.33 (m, 1H), 7.08 (d, *J* = 8.8 Hz, 1H), 6.89 (dd, *J* = 7.8, 1.1 Hz, 1H), 3.72 (s, 3H), 3.43 (s, 3H).

^{13}C NMR (125 MHz, $CDCl_3$) δ 178.11, 176.93, 164.46, 151.80, 141.53, 140.78, 136.46, 135.79, 135.66, 133.76, 132.08, 131.77, 131.46, 131.25, 130.73, 130.06, 128.97, 127.91, 127.28, 127.14, 127.10, 126.16, 124.05, 118.89, 110.68, 52.64.

HRMS (ESI) calcd for [M+H] $C_{28}H_{19}NO_4$, m/z: 434.1387, found: 434.1384.

HPLC analysis: CHIRALCEL IA, hexane/isopropanol = 50/50, 1.0 mL/min, λ = 254 nm, t_R (minor) = 11.4 min, t_R (major) = 20.3 min, ee = 99%.

Supplementary Figure 23. Chiral HPLC spectrum of racemic **3a-12**

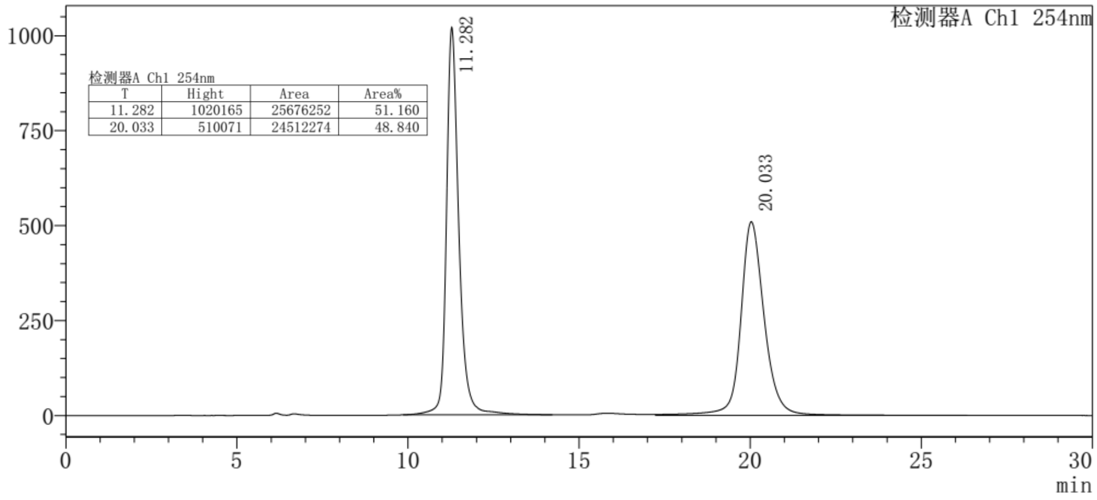

Supplementary Figure 24. Chiral HPLC spectrum of **(*R*)-3a-12**

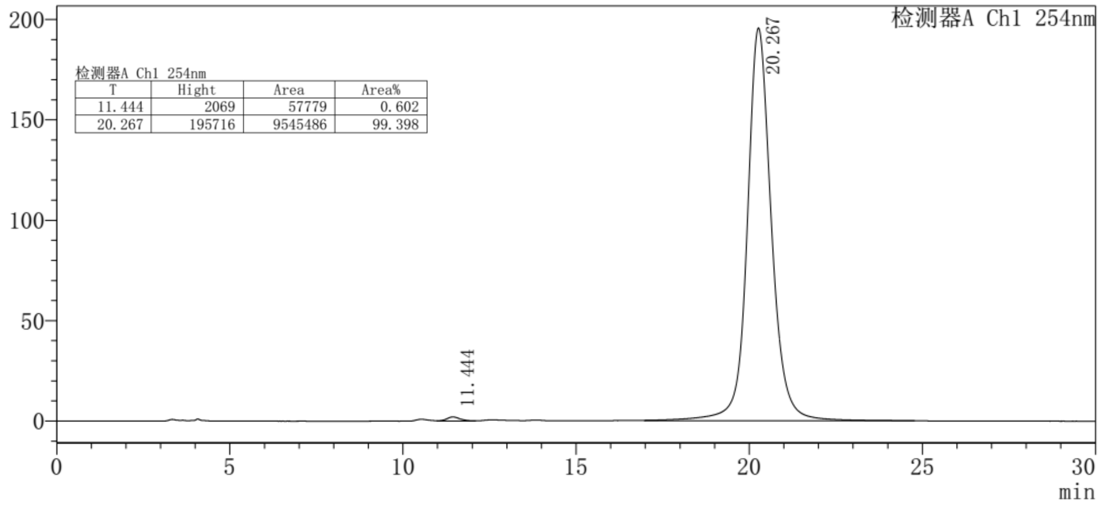

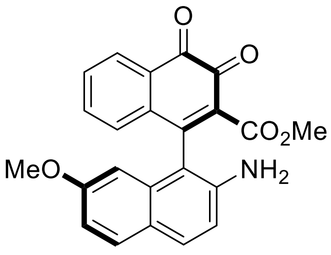

3a-13

(*R*)-methyl 2'-amino-7'-methoxy-3,4-dioxo-3,4-dihydro-[1,1'-binaphthalene]-2-carboxylate ((*R*)-3a-13)

According to the general procedure, **(*R*)-3a-13** was obtained in **81% yield** with **99% ee**.

^{1}H NMR (500 MHz, CDCl$_3$) δ 8.23 (dd, *J* = 7.6, 1.4 Hz, 1H), 7.70 (dd, *J* = 8.7, 0.7 Hz, 1H), 7.65 (d, *J* = 8.9 Hz, 1H), 7.55 (td, *J* = 7.6, 1.2 Hz, 1H), 7.46 (td, *J* = 7.6, 1.5 Hz, 1H), 6.94 (dd, *J* = 8.9, 2.4 Hz, 1H), 6.90-6.86 (m, 2H), 6.67 (d, *J* = 2.4 Hz, 1H), 3.98 (s, 2H), 3.72 (s, 3H), 3.42 (s, 3H).

^{13}C NMR (125 MHz, CDCl$_3$) δ 178.18, 177.01, 164.44, 158.94, 152.07, 142.01, 136.49, 135.68, 133.57, 133.25, 132.06, 131.82, 130.91, 130.72, 130.01, 129.88, 123.04, 115.88, 114.87, 110.21, 103.04, 55.51, 52.60.

HRMS (ESI) calcd for [M+H] $C_{23}H_{17}NO_5$, m/z: 388.1179, found: 388.1175.

HPLC analysis: CHIRALCEL IB, hexane/isopropanol = 60/40, 1.0 mL/min, λ = 254 nm, t_R (major) = 13.5 min, t_R (minor) = 37.3 min, ee = 99%.

Supplementary Figure 25. Chiral HPLC spectrum of racemic **3a-13**

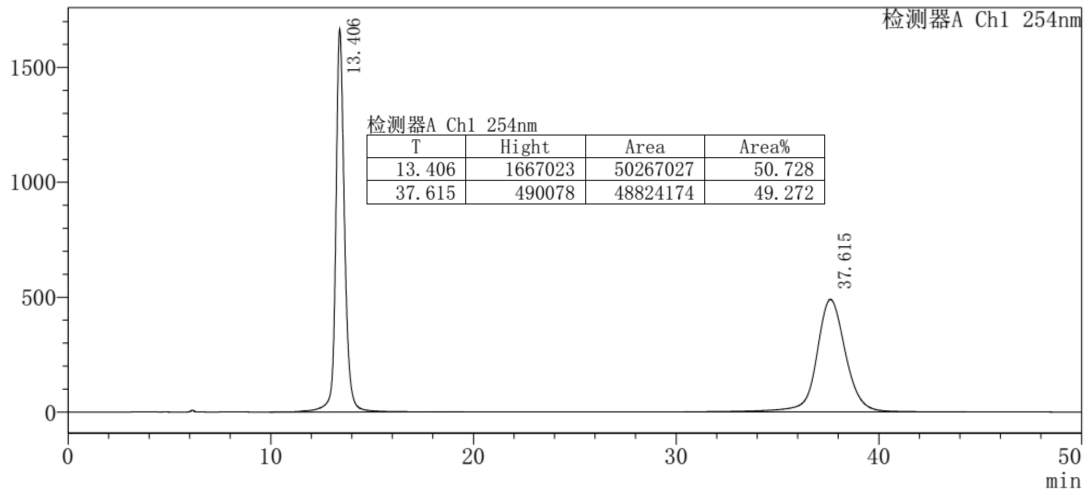

Supplementary Figure 26. Chiral HPLC spectrum of **(*R*)-3a-13**

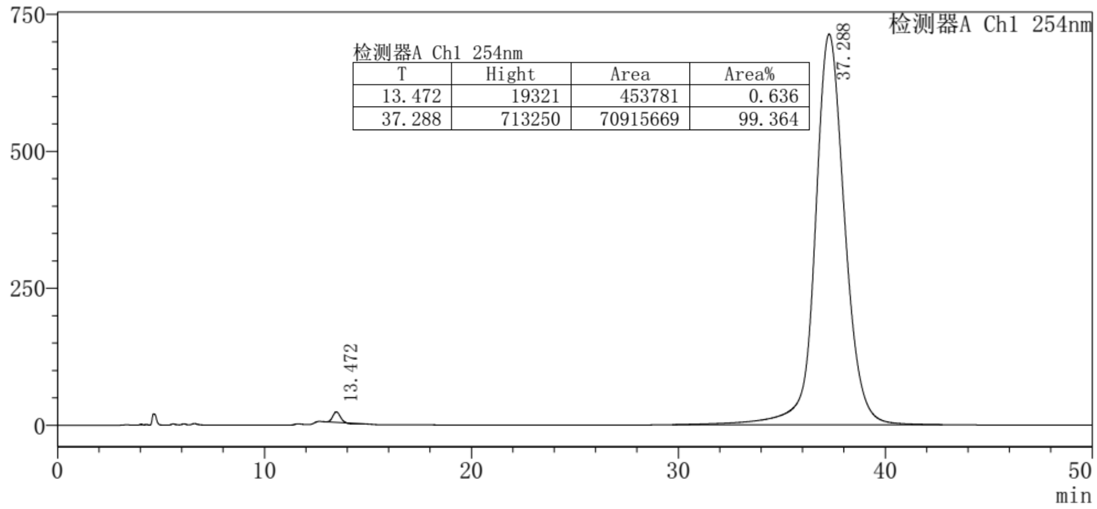

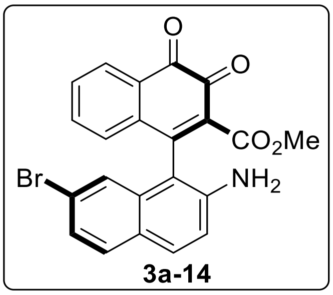

(*R*)-Methyl

2'-amino-7'-bromo-3,4-dioxo-3,4-dihydro-[1,1'-binaphthalene]-2-carboxylate

((*R*)-3a-14)

According to the general procedure, **(*R*)-3a-14** was obtained in **72% yield** with **99% ee**.

^{1}H NMR (500 MHz, $CDCl_3$) δ 8.23 (dd, *J* = 7.6, 1.5 Hz, 1H), 7.74 (dd, *J* = 8.9, 0.8 Hz, 1H), 7.61 (d, *J* = 8.6 Hz, 1H), 7.57 (td, *J* = 7.6, 1.2 Hz, 1H), 7.52 (d, *J* = 1.9 Hz, 1H), 7.47 (td, *J* = 7.6,

1.5 Hz, 1H), 7.34 (dd, J = 8.6, 1.9 Hz, 1H), 7.04 (d, J = 8.8 Hz, 1H), 6.82 (dd, J = 7.8, 1.1 Hz, 1H), 3.41 (s, 3H).

^{13}C NMR (125 MHz, $CDCl_3$) δ 177.96, 176.85, 164.29, 150.89, 142.34, 136.50, 135.80, 133.37, 133.25, 132.25, 131.82, 131.06, 130.88, 129.92, 129.85, 126.46, 125.97, 125.39, 122.03, 118.75, 109.60, 52.61.

HRMS (ESI) calcd for [M+H] $C_{22}H_{15}{}^{79}BrNO_4$, m/z: 436.0179, found: 436.0177; $C_{22}H_{15}{}^{81}BrNO_4$, m/z: 438.0159, found: 438.0157.

HPLC analysis: CHIRALCEL IB, hexane/isopropanol = 50/50, 1.0 mL/min, λ = 254 nm, t_R (major) = 7.8 min, t_R (minor) = 18.4 min, ee = 99%.

Supplementary Figure 27. Chiral HPLC spectrum of racemic **3a-14**

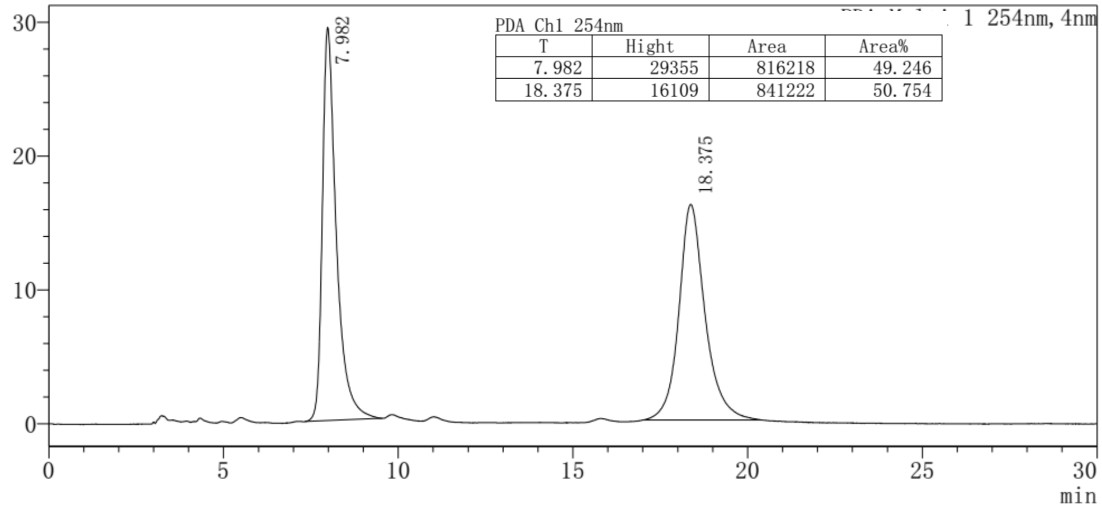

Supplementary Figure 28. Chiral HPLC spectrum of **(*R*)-3a-14**

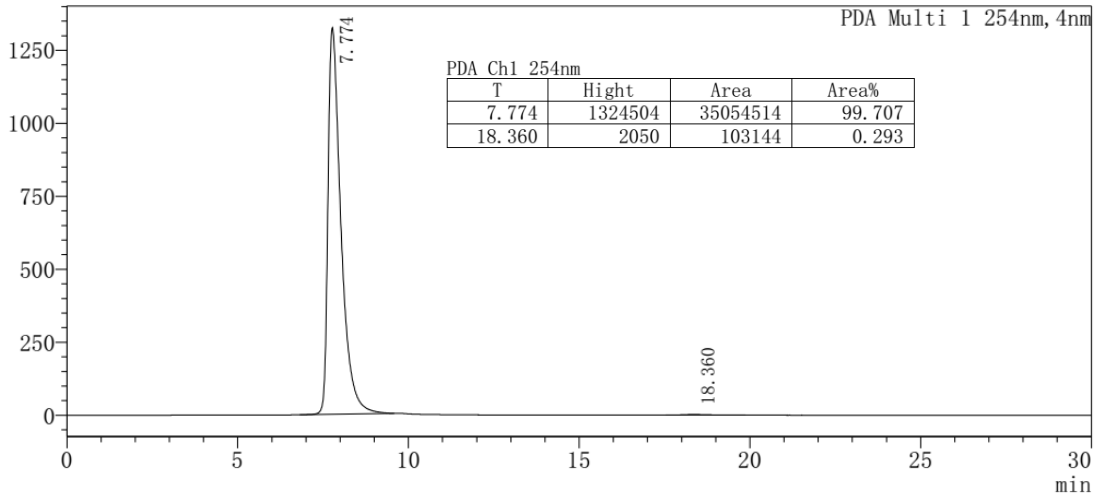

General Procedure for Asymmetric Syntheses of Arylquinones (*S*)-3b

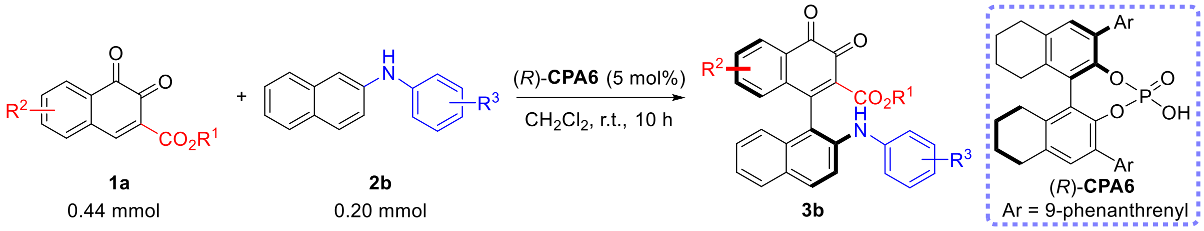

To a mixture of (*R*)-**CPA6** (5 mol%), *o*-naphthoquinone **1a** (0.44 mmol) and nucleophile **2b** (0.20 mmol) in 10 mL Schlenk tube was added CH_2Cl_2 (4.0 mL) under Ar atmosphere. Then the reaction mixture was stirred vigorously at room temperature until the nucleophile completely consumed (monitored by thin-layer chromatography). Typical reaction time was about 10 hours. After that, the resulting mixture was concentrated under reduced pressure and purified by flash chromatography on silica gel (eluent: PE/EA = 6/1) to afford the corresponding axially chiral aryl *o*-naphthoquinones (*S*)-**3b**.

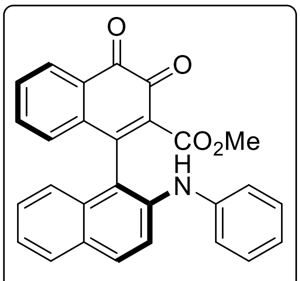

3b-1

(*S*)-Methyl 3,4-dioxo-2'-(phenylamino)-3,4-dihydro-[1,1'-binaphthalene]-2-carboxylate ((*S*)-3b-1)

According to the general procedure, **(*S*)-3b-1** was obtained in **93% yield** with **99% ee**.

^{1}H NMR (400 MHz, DMSO-*d*$_6$) δ 8.05 (dd, *J* = 7.6, 1.4 Hz, 1H), 7.85 (d, *J* = 9.1 Hz, 1H), 7.80 (d, *J* = 7.7 Hz, 1H), 7.64 (d, *J* = 8.2 Hz, 1H), 7.54-7.50 (m, 1H), 7.47-7.40 (m, 2H), 7.38 (s, 1H), 7.34-7.25 (m, 2H), 7.25-7.20 (m, 2H), 7.03 (d, *J* = 7.8 Hz, 2H), 6.94-6.89 (m, 1H), 6.70 (d, *J* = 7.7 Hz, 1H), 3.22 (s, 3H);

^{13}C NMR (100 MHz, DMSO-*d*$_6$) δ 177.59, 176.11, 164.50, 148.30, 142.54, 139.15, 135.79, 134.82, 134.44, 132.96, 132.16, 131.07, 129.83, 129.09, 128.64, 128.24, 127.87, 127.73, 126.81, 124.46, 123.18, 121.92, 120.20, 117.42, 114.97, 51.64.

HRMS (ESI) calcd for [M+H] $C_{28}H_{20}NO_4$, m/z: 434.1387, found: 434.1379.

HPLC analysis: CHIRALCEL IB, hexane/isopropanol = 60/40, 1.0 mL/min, λ = 254 nm, t_R (minor) = 7.6 min, t_R (major) = 8.6 min, ee = 99%.

Supplementary Figure 29. Chiral HPLC spectrum of racemic **3b-1**

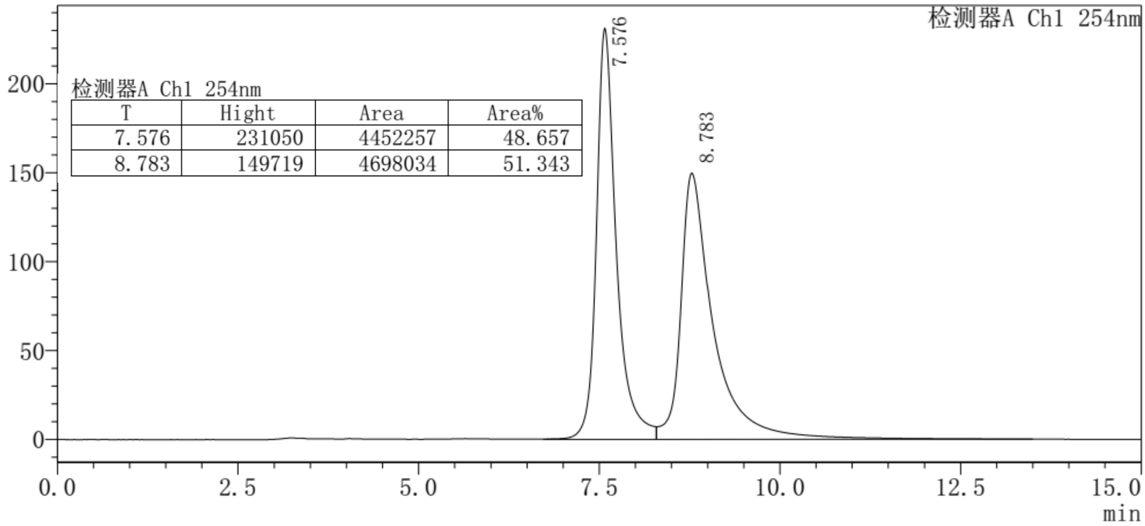

Supplementary Figure 30. Chiral HPLC spectrum of **(*S*)-3b-1**

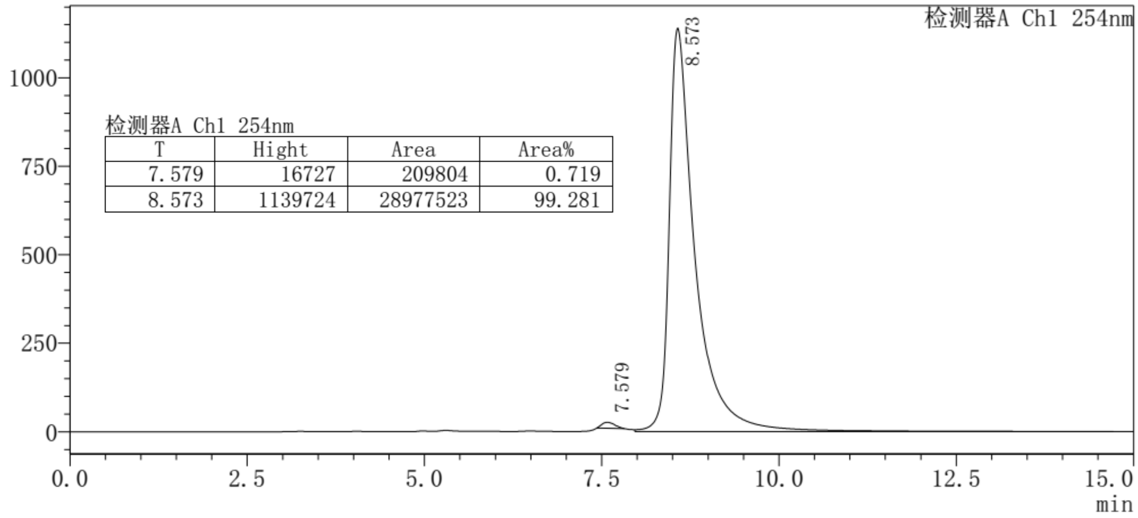

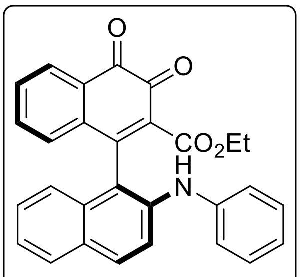

3b-2

(*S*)-Ethyl 3,4-dioxo-2'-(phenylamino)-3,4-dihydro-[1,1'-binaphthalene]-2-carboxylate ((*S*)-3b-2)

According to the general procedure, **(*S*)-3b-2** was obtained in **90% yield** with **94% ee**.

^{1}H NMR (500 MHz, CDCl$_3$) δ 8.22 (dd, J = 7.7, 1.3 Hz, 1H), 7.86 (d, J = 9.0 Hz, 1H), 7.81 (d, J = 7.1 Hz, 1H), 7.61 (d, J = 8.9 Hz, 1H), 7.54-7.50 (m, 2H), 7.45-7.35 (m, 3H), 7.27-7.23 (m, 3H), 7.02 (d, J = 8.3 Hz, 2H), 6.95 (td, J = 7.4, 1.1 Hz, 1H), 6.91 (d, J = 7.8 Hz, 1H), 6.10 (s, 1H), 4.00-3.87 (m, 2H), 0.66 (t, J = 7.1 Hz, 3H).

^{13}C NMR (125 MHz, CDCl$_3$) δ 178.14, 176.40, 164.13, 151.87, 142.60, 139.10, 136.33, 136.11, 134.32, 132.09, 132.04, 131.79, 130.78, 129.56, 129.39, 128.35, 127.63, 124.54, 124.26, 121.95, 119.21, 118.85, 117.83, 61.93, 13.47.

HRMS (ESI) calcd for [M+H] $C_{29}H_{22}NO_4$, m/z: 448.1543, found: 448.1542.

HPLC analysis: CHIRALCEL AD-H, hexane/isopropanol = 70/30, 1.0 mL/min, λ = 254 nm, t_R (major) = 10.8 min, t_R (minor) = 14.6 min, ee = 94%.

Supplementary Figure 31. Chiral HPLC spectrum of racemic **3b-2**

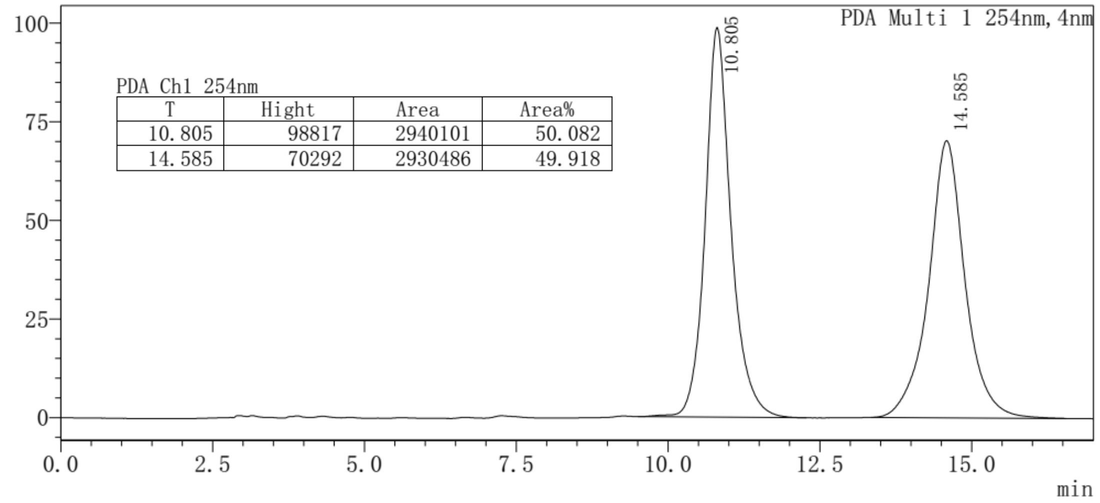

Supplementary Figure 32. Chiral HPLC spectrum of **(*S*)-3b-2**

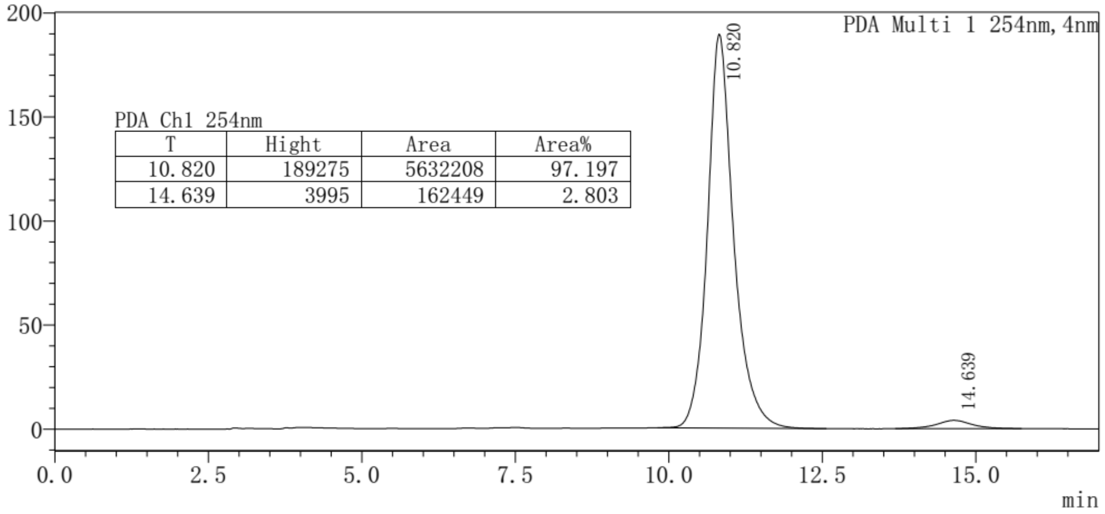

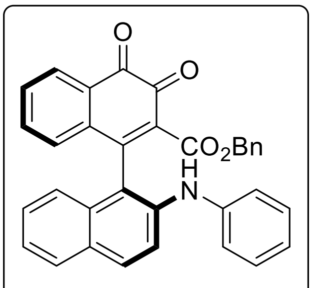

3b-3

(*S*)-Benzyl 3,4-dioxo-2’-(phenylamino)-3,4-dihydro-[1,1’-binaphthalene]-2-carboxylate ((*S*)-3b-3)

According to the general procedure, **(*S*)-3b-3** was obtained in **92% yield** with **87% ee**.

^{1}H NMR (400 MHz, DMSO-*d6*) δ 8.04 (dd, *J* = 7.6, 1.5 Hz, 1H), 7.86-7.80 (m, 2H), 7.67-7.64 (m, 1H), 7.52 (td, *J* = 7.5, 1.3 Hz, 1H), 7.45-7.37 (m, 3H), 7.30-7.26 (m, 2H), 7.21-7.13 (m, 3H), 7.07 (t, *J* = 7.4 Hz, 2H), 6.94-6.87 (m, 3H), 6.71 (d, *J* = 8.3 Hz, 2H), 4.81-4.69 (m, 2H).

^{13}C NMR (100 MHz, DMSO-*d6*) δ 177.58, 176.11, 163.87, 148.19, 142.32, 139.27, 135.70, 135.04, 134.83, 134.46, 133.01, 132.32, 131.08, 129.90, 129.04, 128.63, 128.21, 128.07, 127.92, 127.75, 127.71, 127.40, 126.90, 124.53, 123.14, 121.98, 120.44, 117.17, 114.61, 66.16.

HRMS (ESI) calcd for [M+H] $C_{34}H_{24}NO_4$, m/z: 510.1700, found: 510.1699.

HPLC analysis: CHIRALCEL AD-3, hexane/isopropanol = 80/20, 0.8 mL/min, λ = 254 nm, t_R (major) = 15.6 min, t_R (minor) = 23.8 min, ee = 87%.

Supplementary Figure 33. Chiral HPLC spectrum of racemic **3b-3**

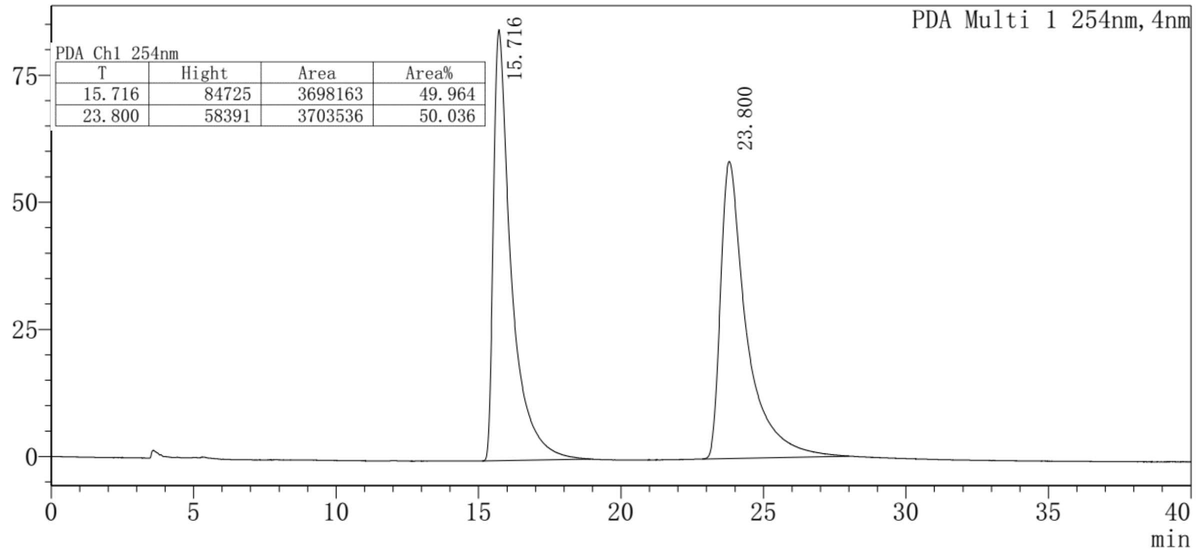

Supplementary Figure 34. Chiral HPLC spectrum of **(*S*)-3b-3**

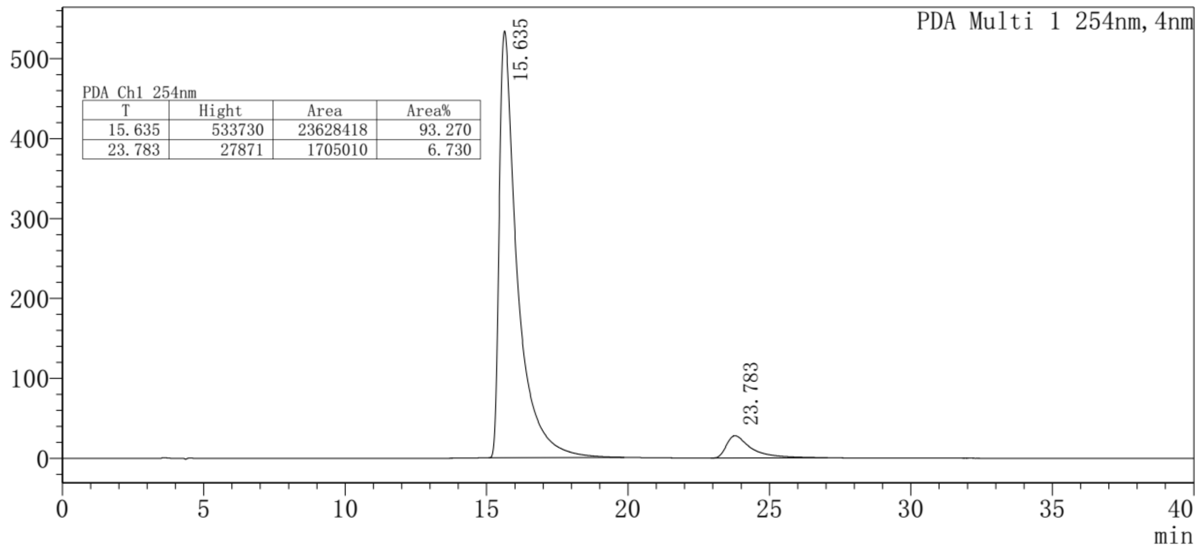

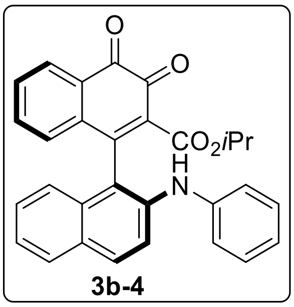

(*S*)-Isopropyl 3,4-dioxo-2’-(phenylamino)-3,4-dihydro-[1,1’-binaphthalene]-2-carboxylate ((*S*)-3b-4)

According to the general procedure, **(*S*)-3b-4** was obtained in **92% yield** with **93% ee**.

^{1}H NMR (500 MHz, CDCl$_3$) δ 8.21 (dd, *J* = 7.6, 1.4 Hz, 1H), 7.86 (d, *J* = 9.0 Hz, 1H), 7.81 (dd, *J* = 7.7, 1.6 Hz, 1H), 7.62 (d, *J* = 9.0 Hz, 1H), 7.54 (d, *J* = 8.3 Hz, 1H), 7.51 (td, *J* = 7.6, 1.2 Hz, 1H), 7.45-7.35 (m, 4H), 7.27-7.23 (m, 2H), 7.05-7.02 (m, 2H), 6.97-6.92 (m, 2H), 6.11 (s, 1H), 4.81 (p, *J* = 6.3 Hz, 1H), 0.84 (d, *J* = 6.2 Hz, 3H), 0.60 (d, *J* = 6.2 Hz, 3H).

^{13}C NMR (125 MHz, CDCl$_3$) δ 178.17, 176.36, 163.51, 151.46, 142.54, 139.07, 136.30, 136.25, 134.32, 132.07, 131.99, 131.76, 130.70, 129.54, 129.35, 129.24, 128.27, 127.55, 124.48, 124.31, 121.91, 119.08, 118.84, 117.76, 69.86, 21.18, 20.86.

HRMS (ESI) calcd for [M+H] $C_{30}H_{23}NO_4$, m/z: 462.1700, found: 462.1695.

HPLC analysis: CHIRALCEL AD-3, hexane/isopropanol = 80/20, 1.0 mL/min, λ = 254 nm, t_R (major) = 13.7 min, t_R (minor) = 28.3 min, ee = 93%.

Supplementary Figure 35. Chiral HPLC spectrum of racemic **3b-4**

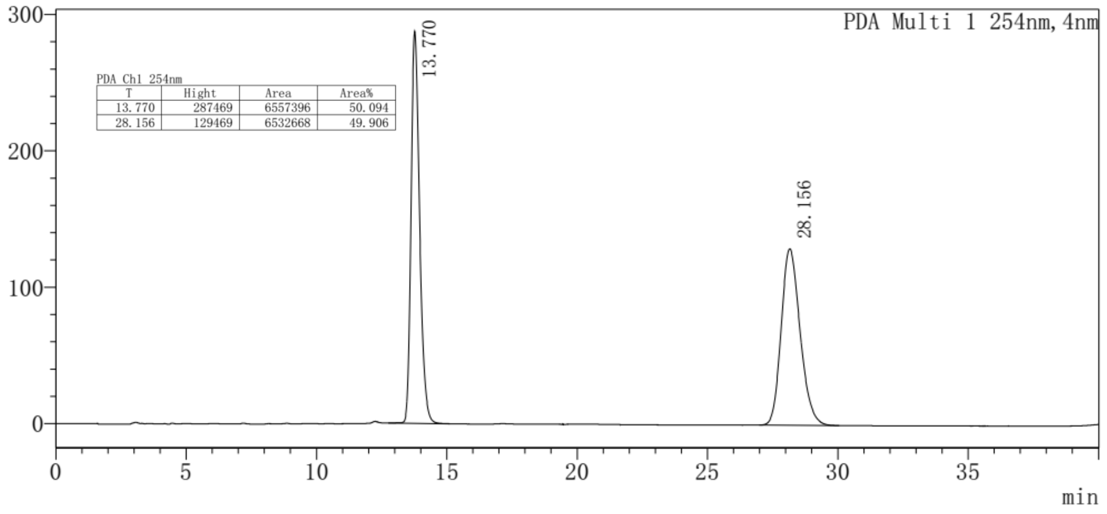

Supplementary Figure 36. Chiral HPLC spectrum of **(*S*)-3b-4**

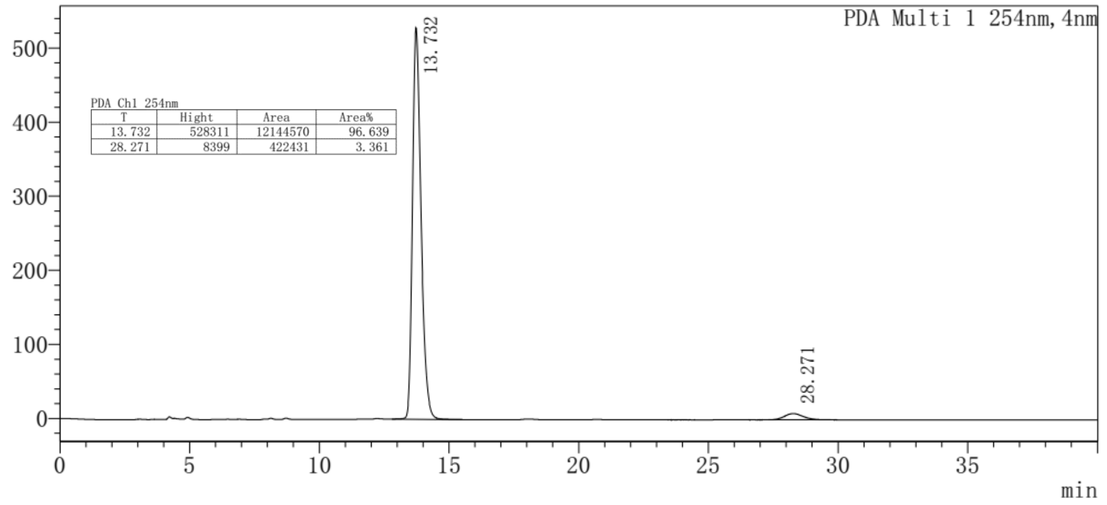

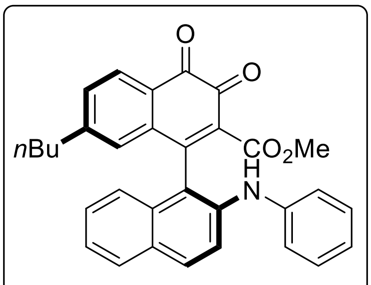

3b-5

(*S*)-Methyl 7-butyl-3,4-dioxo-2'-(phenylamino)-3,4-dihydro-[1,1'-binaphthalene]-2-carboxylate ((*S*)-3b-5)

According to the general procedure, **(*S*)-3b-5** was obtained in **92% yield** with **93% ee**.

^{1}H NMR (500 MHz, $CDCl_3$) δ 8.12 (d, *J* = 7.7 Hz, 1H), 7.88 (d, *J* = 9.0 Hz, 1H), 7.83 (dd, *J* = 6.8, 2.4 Hz, 1H), 7.63 (d, *J* = 9.0 Hz, 1H), 7.54 (d, *J* = 9.0 Hz, 1H), 7.39 (tt, *J* = 6.9, 5.3 Hz, 2H),

7.30 (dd, J = 7.9, 1.5 Hz, 1H), 7.23 (t, J = 7.7 Hz, 2H), 7.01 (d, J = 8.3 Hz, 2H), 6.93 (t, J = 7.3 Hz, 1H), 6.66 (s, 1H), 6.11 (s, 1H), 3.44 (s, 2H), 2.40 (t, J = 7.6 Hz, 2H), 1.34-1.26 (m, 2H), 1.15-1.06 (m, 2H), 0.74 (t, J = 7.3 Hz, 3H).

^{13}C NMR (125 MHz, $CDCl_3$) δ 177.74, 176.79, 164.87, 152.80, 152.34, 142.80, 138.88, 135.76, 134.15, 132.10, 131.96, 131.07, 130.78, 129.68, 129.65, 129.49, 129.45, 128.35, 127.55, 124.54, 124.18, 121.76, 119.54, 118.47, 118.25, 52.70, 35.94, 32.78, 22.09, 13.82.

HRMS (ESI) calcd for [M+H] $C_{32}H_{28}NO_4$, m/z: 490.2013, found: 490.2016.

HPLC analysis: CHIRALCEL AD-H, hexane/isopropanol = 80/20, 1.0 mL/min, λ = 254 nm, t_R (major) = 10.4 min, t_R (minor) = 15.3 min, ee = 93%.

Supplementary Figure 37. Chiral HPLC spectrum of racemic **3b-5**

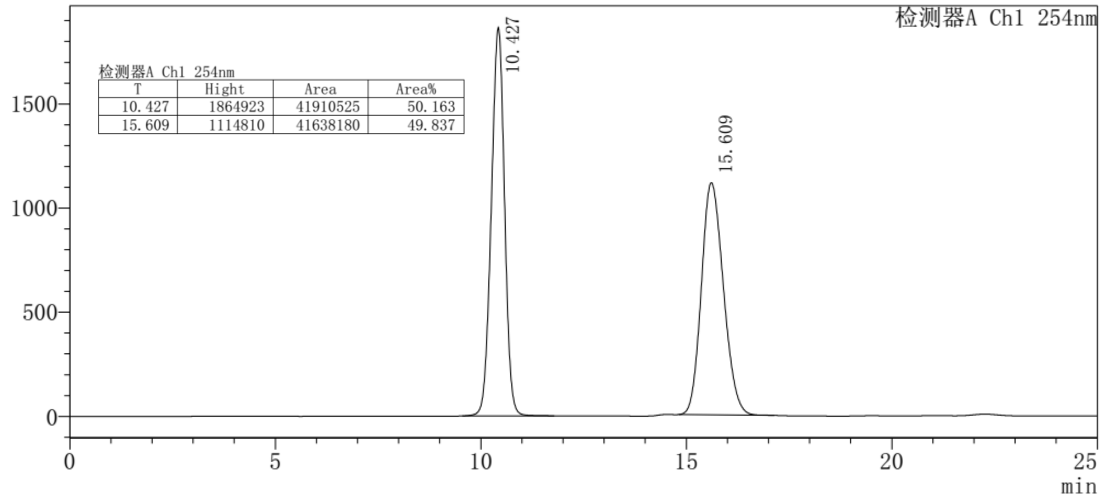

Supplementary Figure 38. Chiral HPLC spectrum of **(*S*)-3b-5**

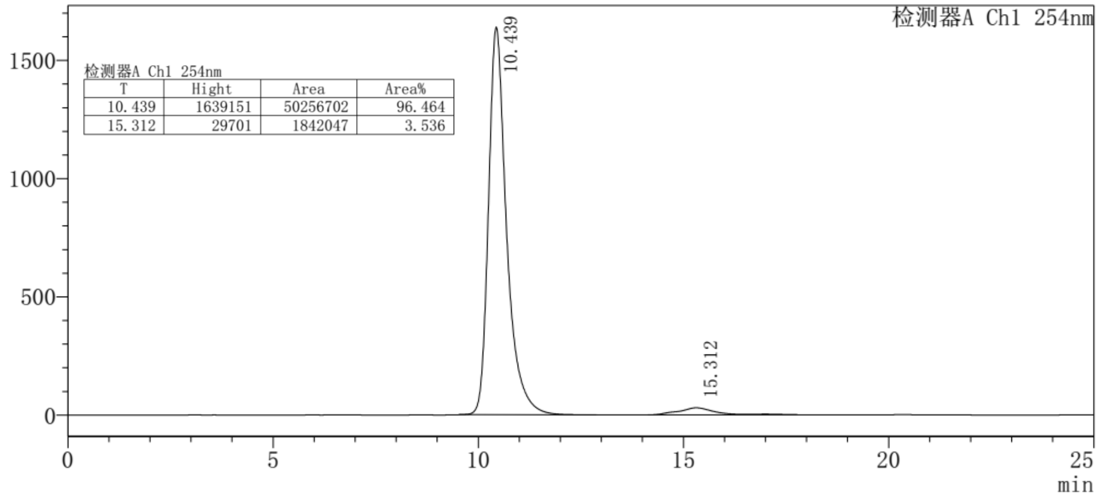

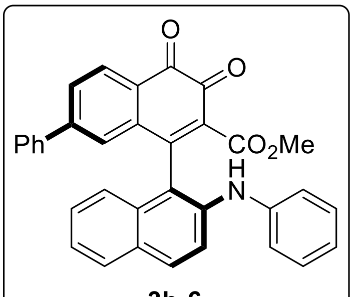

3b-6

(*S*)-Methyl 3,4-dioxo-7-phenyl-2'-(phenylamino)-3,4-dihydro-[1,1'-binaphthalene]-2-carboxylate ((*S*)-3b-6)

According to the general procedure, **(*S*)-3b-6** was obtained in **88% yield** with **98% ee**.

^{1}H NMR (500 MHz, $CDCl_3$) δ 8.28 (d, *J* = 7.9 Hz, 1H), 7.88 (d, *J* = 9.0 Hz, 1H), 7.82 (d, *J* = 7.4 Hz, 1H), 7.69 (dd, *J* = 8.0, 1.7 Hz, 1H), 7.65 (d, *J* = 8.9 Hz, 1H), 7.57 (d, *J* = 8.5 Hz, 1H), 7.43-7.36 (m, 2H), 7.35-7.31 (m, 3H), 7.26-7.21 (m, 4H), 7.08-7.04 (m, 3H), 6.94 (t, *J* = 7.4 Hz, 1H), 6.16 (s, 1H), 3.46 (s, 3H).

^{13}C NMR (125 MHz, $CDCl_3$) δ 177.74, 176.65, 164.80, 152.04, 149.29, 142.75, 138.90, 138.80, 136.18, 134.73, 131.95, 131.43, 130.99, 130.53, 130.31, 129.54, 129.49, 129.16, 129.13, 128.46, 128.24, 127.68, 127.28, 124.63, 124.10, 121.86, 119.50, 118.50, 117.98, 52.79.

HRMS (ESI) calcd for [M+H] $C_{34}H_{24}NO_4$, m/z: 510.1700, found: 510.1705.

HPLC analysis: CHIRALCEL IB, hexane/isopropanol = 80/20, 1.0 mL/min, λ = 254 nm, t_R (minor) = 14.8 min, t_R (major) = 19.2 min, ee = 98%.

Supplementary Figure 39. Chiral HPLC spectrum of racemic **3b-6**

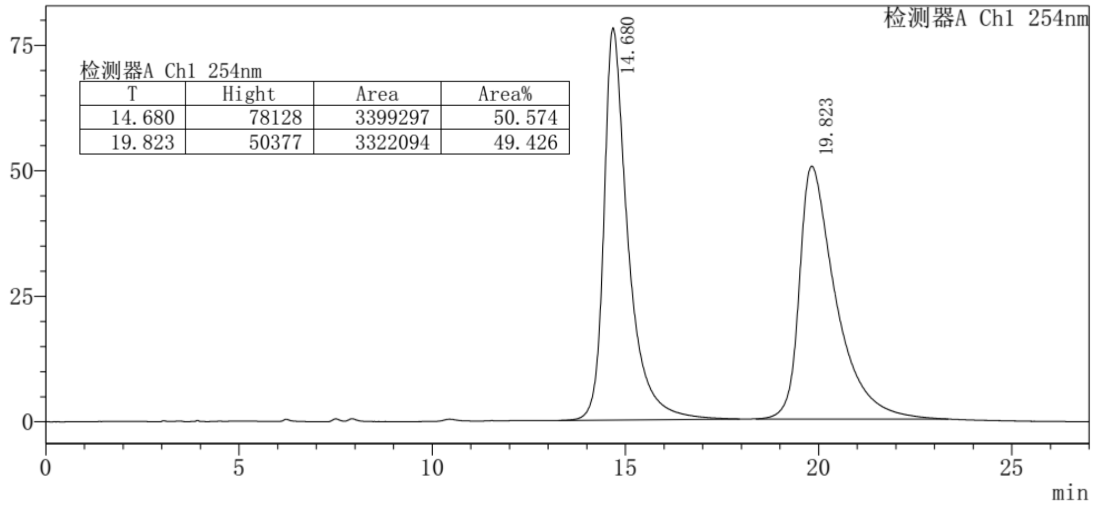

检测器A Ch1 254nm

T	Hight	Area	Area%
14.680	78128	3399297	50.574
19.823	50377	3322094	49.426

Supplementary Figure 40. Chiral HPLC spectrum of **(*S*)-3b-6**

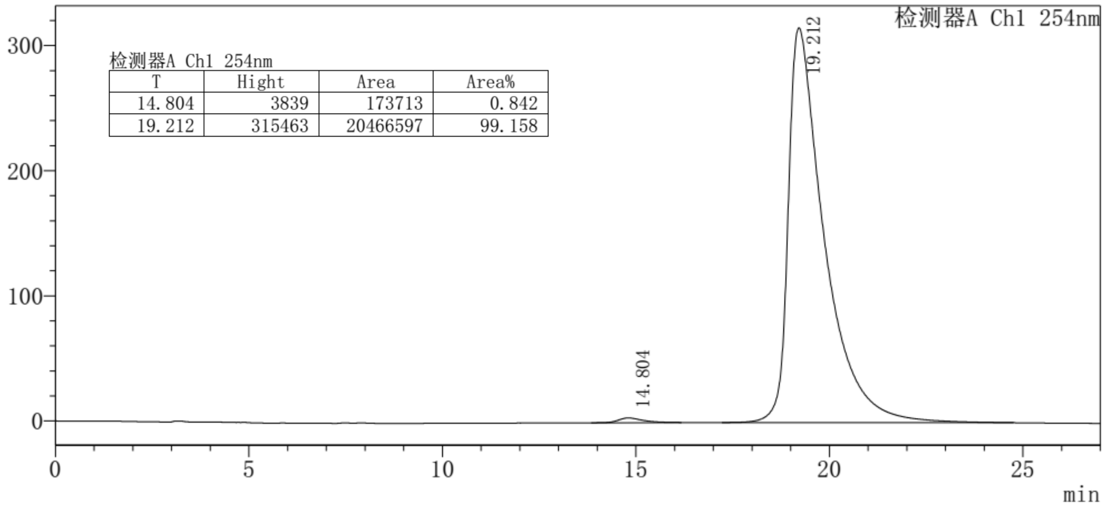

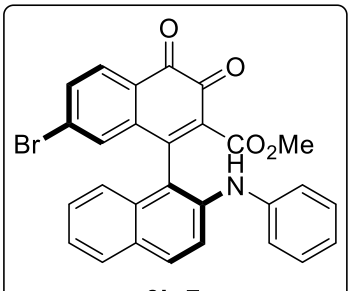

3b-7

(*S*)-Methyl 7-bromo-3,4-dioxo-2'-(phenylamino)-3,4-dihydro-[1,1'-binaphthalene]-2-carboxylate ((*S*)-3b-7)

According to the general procedure, **(*S*)-3b-7** was obtained in **90% yield** with **98% ee**.

^{1}H NMR (500 MHz, CDCl$_3$) δ 8.03 (d, *J* = 8.3 Hz, 1H), 7.89 (d, *J* = 9.0 Hz, 1H), 7.84 (dd, *J* = 7.6, 1.7 Hz, 1H), 7.65-7.60 (m, 2H), 7.51 (d, *J* = 7.5 Hz, 1H), 7.45-7.38 (m, 2H), 7.26 (t, *J* = 7.8 Hz, 1H), 7.02 (dd, *J* = 7.8, 1.4 Hz, 3H), 6.98-6.94 (m, 1H), 6.07 (s, 1H), 3.43 (s, 3H).

^{13}C NMR (125 MHz, CDCl$_3$) δ 177.30, 175.85, 164.39, 150.90, 142.57, 139.26, 136.84, 135.80, 135.11, 132.17, 132.07, 131.85, 131.63, 131.27, 130.34, 129.59, 129.55, 128.56, 127.81, 124.73, 123.77, 122.09, 119.51, 118.81, 118.78, 117.36, 52.83.

HRMS (ESI) calcd for [M+H] $C_{28}H_{19}{}^{79}BrNO_4$, m/z: 512.0492, found: 512.0483; $C_{28}H_{19}{}^{81}BrNO_4$, m/z: 514.0472, found: 514.0490.

HPLC analysis: CHIRALCEL AD-H, hexane/isopropanol = 80/20, 1.0 mL/min, λ = 254 nm, t_R (minor) = 20.4 min, t_R (major) = 26.6 min, ee = 98%.

Supplementary Figure 41. Chiral HPLC spectrum of racemic **3b-7**

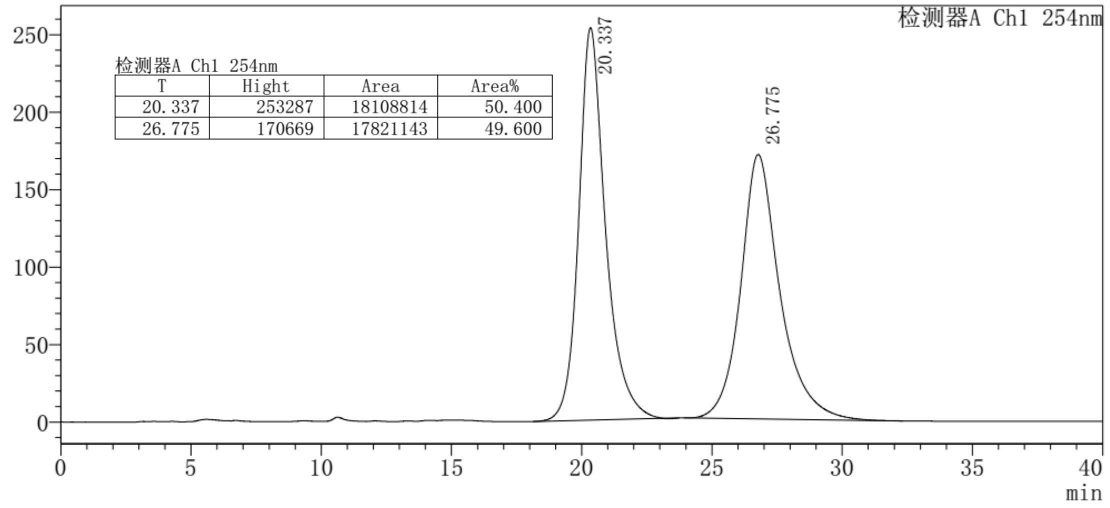

Supplementary Figure 42. Chiral HPLC spectrum of **(*S*)-3b-7**

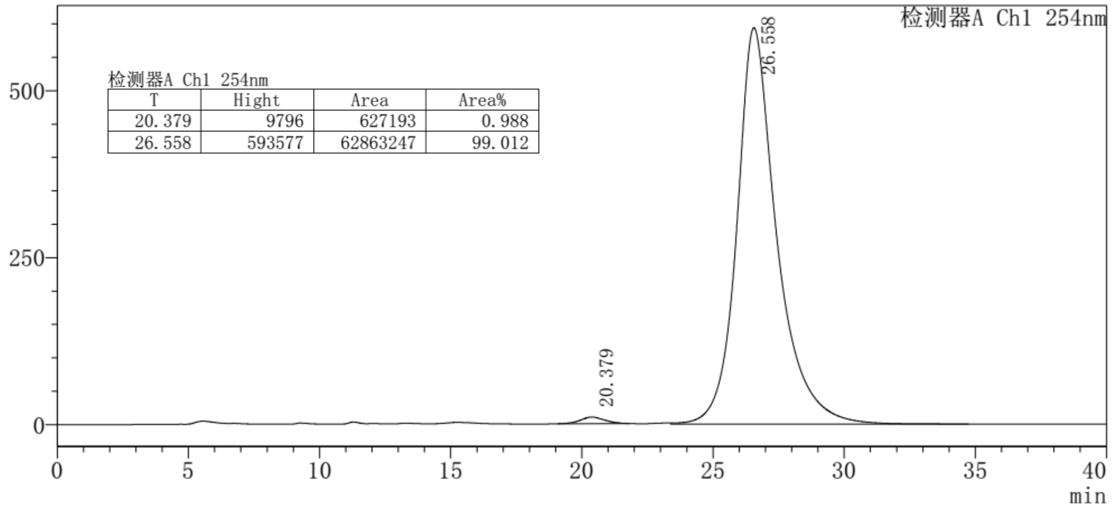

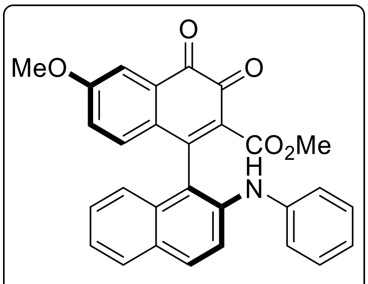

3b-8

(*S*)-Methyl 6-methoxy-3,4-dioxo-2'-(phenylamino)-3,4-dihydro-[1,1'-binaphthalene]-2-carboxylate ((*S*)-3b-8)

According to the general procedure, **(*S*)-3b-8** was obtained in **90% yield** with **94% ee**.

^{1}H NMR (500 MHz, $CDCl_3$) δ 8.21 (d, *J* = 8.5 Hz, 1H), 7.85 (d, *J* = 9.0 Hz, 1H), 7.81 (d, *J* =

7.4 Hz, 1H), 7.62 (d, J = 8.9 Hz, 1H), 7.52 (d, J = 8.1 Hz, 1H), 7.42-7.35 (m, 2H), 7.25 (t, J = 7.7 Hz, 2H), 7.03 (d, J = 8.2 Hz, 2H), 6.97-6.91 (m, 2H), 6.36 (d, J = 2.4 Hz, 1H), 6.13 (s, 1H), 3.66 (s, 3H), 3.45 (s, 3H).

^{13}C NMR (125 MHz, CDCl$_3$) δ 177.11, 176.42, 166.06, 164.82, 151.37, 142.70, 139.01, 136.49, 136.45, 133.91, 132.01, 130.85, 129.53, 129.36, 128.38, 127.64, 125.11, 124.50, 124.09, 121.84, 119.25, 118.66, 117.68, 116.63, 115.19, 55.86, 52.75.

HRMS (ESI) calcd for [M+H] $C_{29}H_{22}NO_5$, m/z: 464.1493, found: 464.1477.

HPLC analysis: CHIRALCEL AD-H, hexane/isopropanol = 70/30, 1.0 mL/min, λ = 254 nm, t_R (major) = 13.9 min, t_R (minor) = 15.9 min, ee = 94%.

Supplementary Figure 43. Chiral HPLC spectrum of racemic **3b-8**

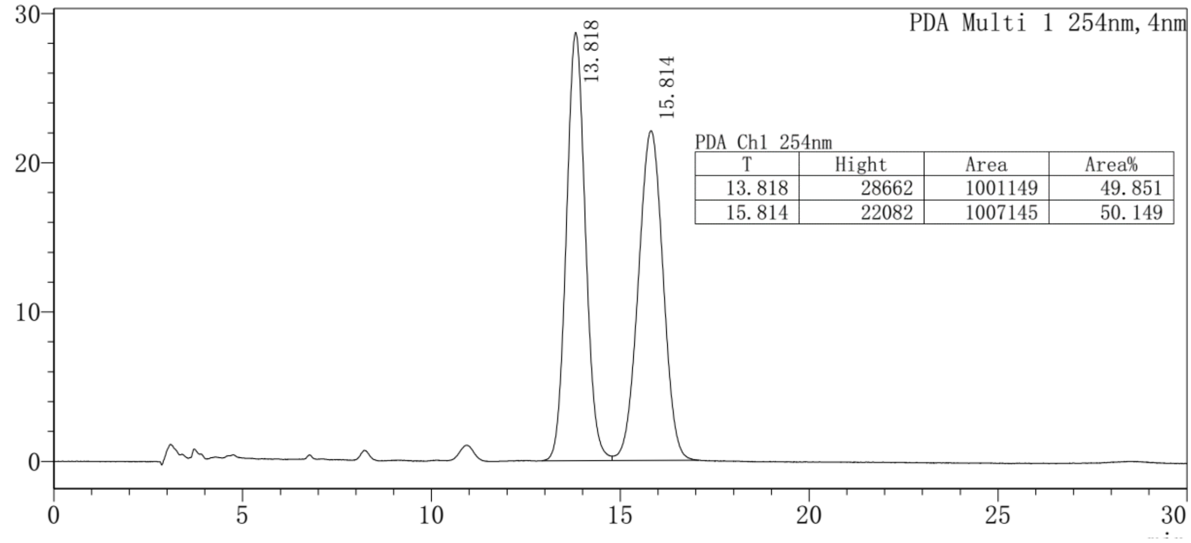

Supplementary Figure 44. Chiral HPLC spectrum of **(*S*)-3b-8**

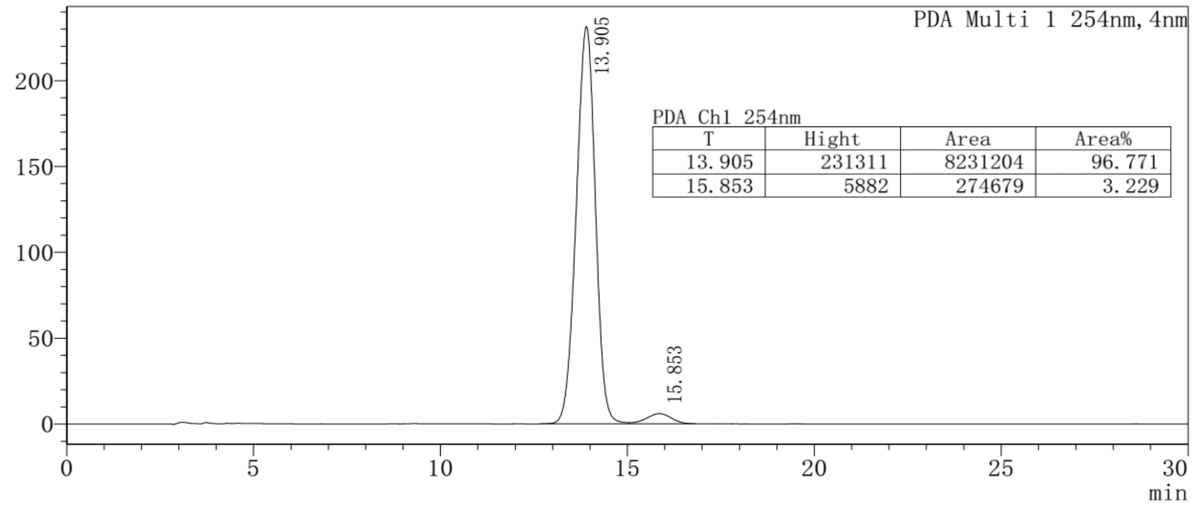

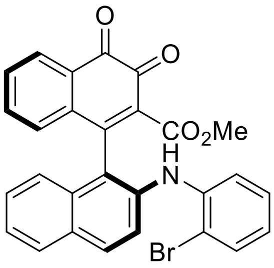

3b-9

(*S*)-Methyl 2'-((2-bromophenyl)amino)-3,4-dioxo-3,4-dihydro-[1,1'-binaphthalene]-2-carboxylate ((*S*)-3b-9)

According to the general procedure, **(*S*)-3b-9** was obtained in **81% yield** with **93% ee**.

^{1}H NMR (500 MHz, $CDCl_3$) δ 8.20 (dd, *J* = 7.6, 1.5 Hz, 1H), 7.91 (d, *J* = 8.9 Hz, 1H), 7.84 (dt, *J* = 7.0, 3.5 Hz, 1H), 7.62 (d, *J* = 9.0 Hz, 1H), 7.55-7.49 (m, 2H), 7.45-7.39 (m, 3H), 7.13 (t, *J* = 2.0 Hz, 1H), 7.07 (t, *J* = 8.0 Hz, 1H), 7.01 (dt, *J* = 8.2, 1.2 Hz, 1H), 7.02-6.99 (m, 1H), 6.82 (dd, *J* = 7.8, 1.2 Hz, 1H), 6.27 (s, 1H), 3.45 (s, 3H).

^{13}C NMR (126 MHz, $CDCl_3$) δ 177.94, 176.38, 164.89, 151.90, 144.56, 137.95, 136.33, 135.53, 134.20, 132.18, 131.83, 131.67, 131.08, 130.79, 130.77, 129.87, 129.52, 128.46, 127.80, 125.08, 124.28, 124.20, 123.16, 120.49, 120.06, 119.57, 116.54, 52.86.

HRMS (ESI) calcd for [M+H] $C_{28}H_{18}{}^{79}BrNO_4$, m/z: 512.0489, found: 512.0486; $C_{28}H_{18}{}^{81}BrNO_4$, m/z: 514.0472, found: 514.0469.

HPLC analysis: CHIRALCEL AD-3, hexane/isopropanol = 80/20, 1.0 mL/min, λ = 254 nm, t_R (major) = 9.0 min, t_R (minor) = 12.0 min, ee = 93%.

Supplementary Figure 45. Chiral HPLC spectrum of racemic **3b-9**

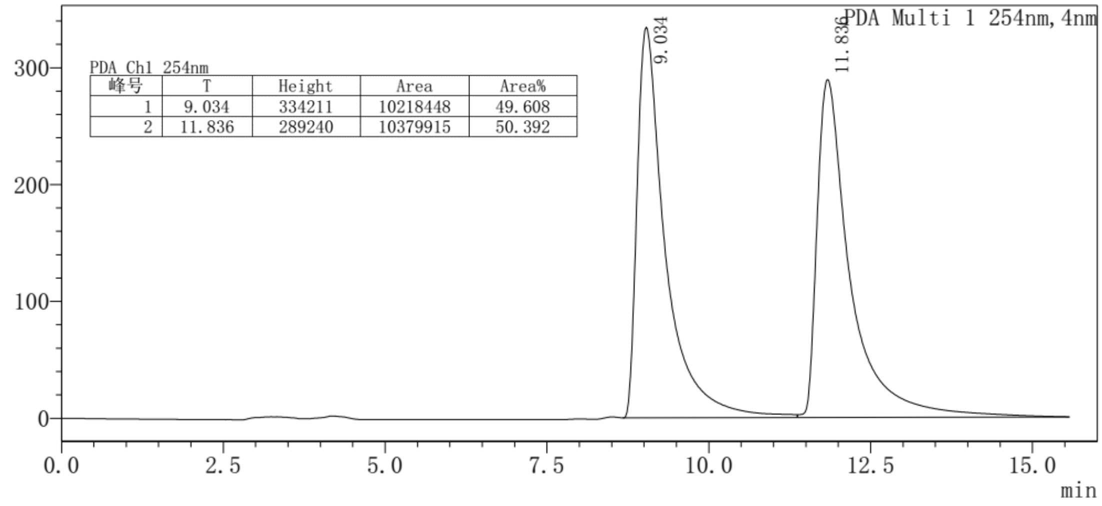

Supplementary Figure 46. Chiral HPLC spectrum of **(*S*)-3b-9**

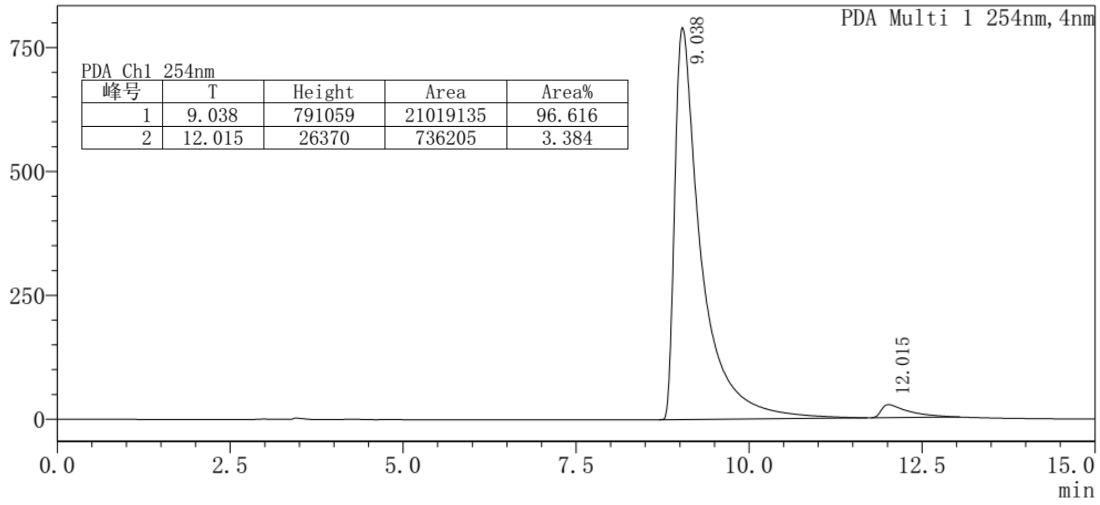

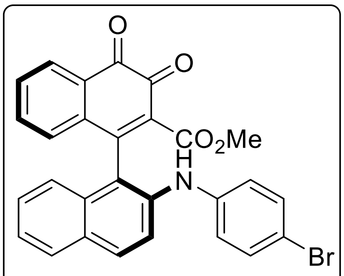

3b-10

(*S*)-Methyl 2'-((4-bromophenyl)amino)-3,4-dioxo-3,4-dihydro-[1,1'-binaphthalene]-2-carboxylate ((*S*)-3b-10)

According to the general procedure, **(*S*)-3b-10** was obtained in **95% yield** with **96% ee**.

^{1}H NMR (500 MHz, $CDCl_3$) δ 8.18 (dd, *J* = 7.5, 1.5 Hz, 1H), 7.89 (d, *J* = 9.0 Hz, 1H), 7.85-7.82 (m, 1H), 7.57 (d, *J* = 9.0 Hz, 1H), 7.54-7.49 (m, 2H), 7.44-7.38 (m, 3H), 7.33-7.30

(m, 2H), 6.91-6.87 (m, 2H), 6.83 (d, J = 7.7 Hz, 1H), 6.24 (s, 1H), 3.43 (s, 3H).

^{13}C NMR (125 MHz, $CDCl_3$) δ 177.97, 176.39, 164.84, 151.99, 142.07, 138.40, 136.29, 135.62, 134.21, 132.33, 132.17, 131.85, 131.70, 130.98, 130.74, 129.63, 129.45, 128.43, 127.76, 124.86, 124.16, 119.89, 119.58, 118.82, 113.64, 52.80.

HRMS (ESI) calcd for [M+H] $C_{28}H_{18}{}^{79}BrNO_4$, m/z: 512.0492, found: 512.0486; $C_{28}H_{18}{}^{81}BrNO_4$, m/z: 514.0472, found: 514.0464.

HPLC analysis: CHIRALCEL OD-H, hexane/isopropanol = 80/20, 1.0 mL/min, λ = 254 nm, t_R (major) = 16.1 min, t_R (minor) = 22.8 min, ee = 96%.

Supplementary Figure 47. Chiral HPLC spectrum of racemic **3b-10**

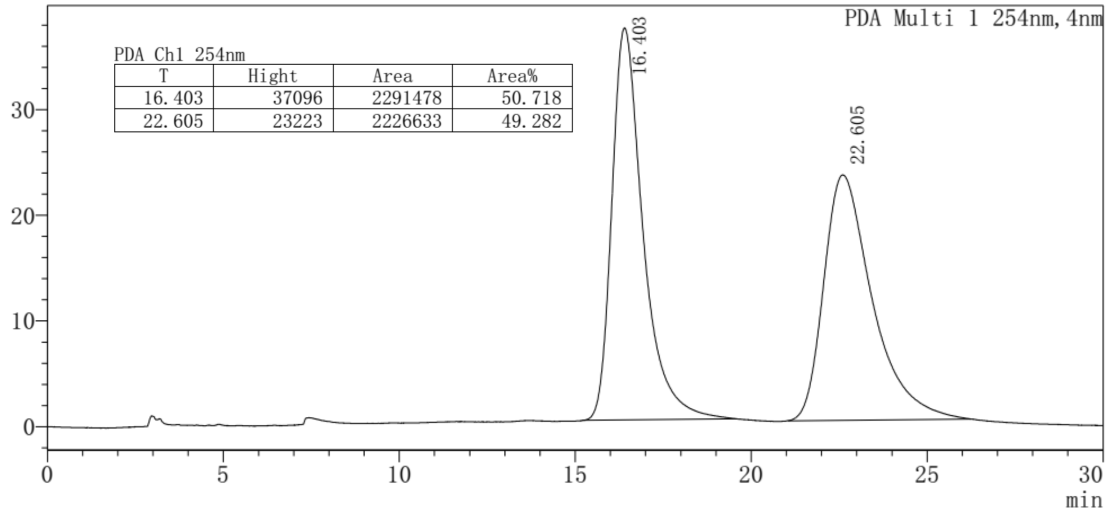

Supplementary Figure 48. Chiral HPLC spectrum of **(*S*)-3b-10**

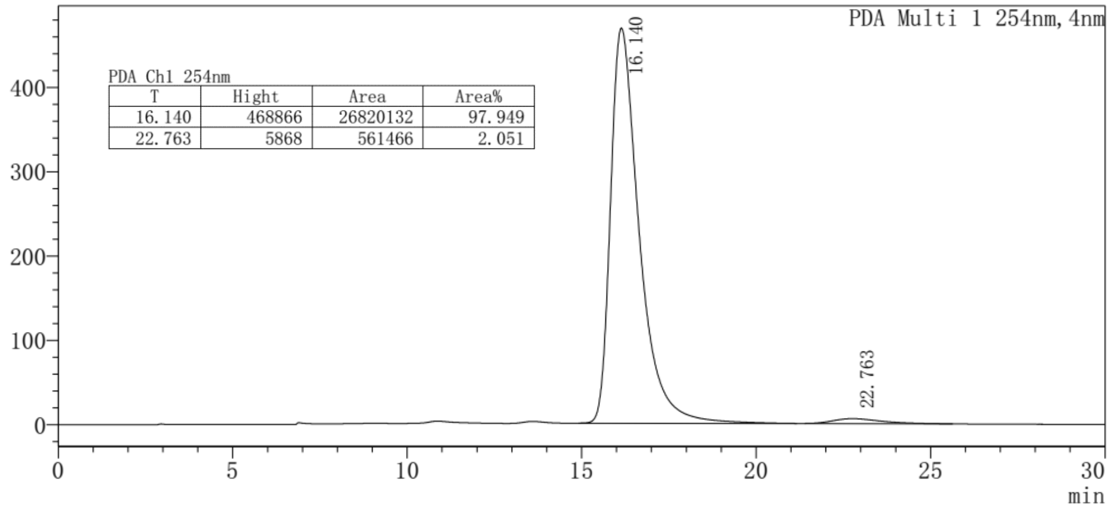

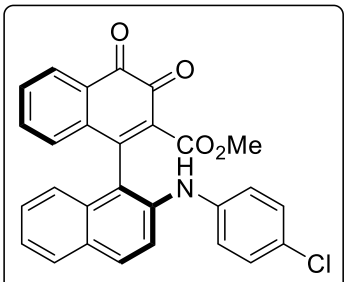

3b-11

(*S*)-Methyl 2’-((4-chlorophenyl)amino)-3,4-dioxo-3,4-dihydro-[1,1’-binaphthalene]-2-carboxylate ((*S*)-3b-11)

According to the general procedure, **(*S*)-3b-11** was obtained in **93% yield** with **95% ee**.

^{1}H NMR (500 MHz, CDCl$_3$) δ 8.21 (dd, *J* = 7.6, 1.5 Hz, 1H), 7.88 (d, *J* = 9.0 Hz, 1H), 7.88-7.81 (m, 1H), 7.56 (d, *J* = 9.0 Hz, 1H), 7.52 (td, *J* = 7.6, 1.2 Hz, 2H), 7.43 (dd, *J* = 7.7, 1.5 Hz, 1H), 7.42-7.38 (m, 2H), 7.20-7.16 (m, 2H), 6.96-6.92 (m, 2H), 6.83 (dd, *J* = 7.9, 1.2 Hz, 1H), 6.17 (s, 1H), 3.45 (s, 3H).

^{13}C NMR (125 MHz, CDCl$_3$) δ 178.00, 176.42, 164.88, 152.00, 141.51, 138.62, 136.31, 135.71, 134.23, 132.19, 131.88, 131.75, 131.01, 130.79, 129.59, 129.48, 128.45, 127.78, 126.53, 124.83, 124.16, 119.73, 119.43, 118.56, 52.84.

HRMS (ESI) calcd for [M+H] $C_{28}H_{18}{}^{35}ClNO_4$, m/z: 468.0997, found: 468.0980; $C_{28}H_{18}{}^{37}ClNO_4$, m/z: 470.0965, found: 470.0974.

HPLC analysis: CHIRALCEL OD-H, hexane/isopropanol = 80/20, 1.0 mL/min, λ = 254 nm, t_R (major) = 14.9 min, t_R (minor) = 20.5 min, ee = 95%.

Supplementary Figure 49. Chiral HPLC spectrum of racemic **3b-11**

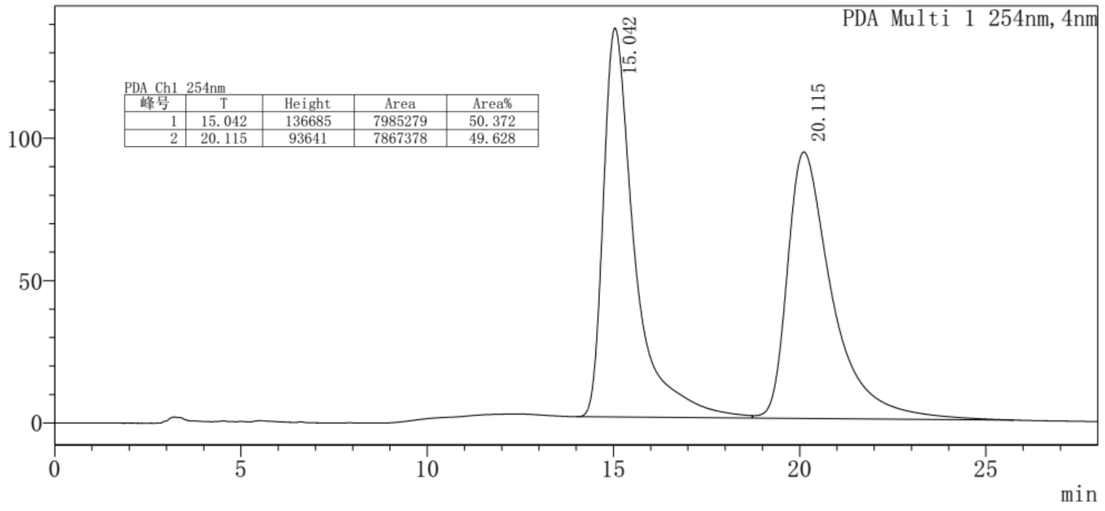

Supplementary Figure 50. Chiral HPLC spectrum of **(*S*)-3b-11**

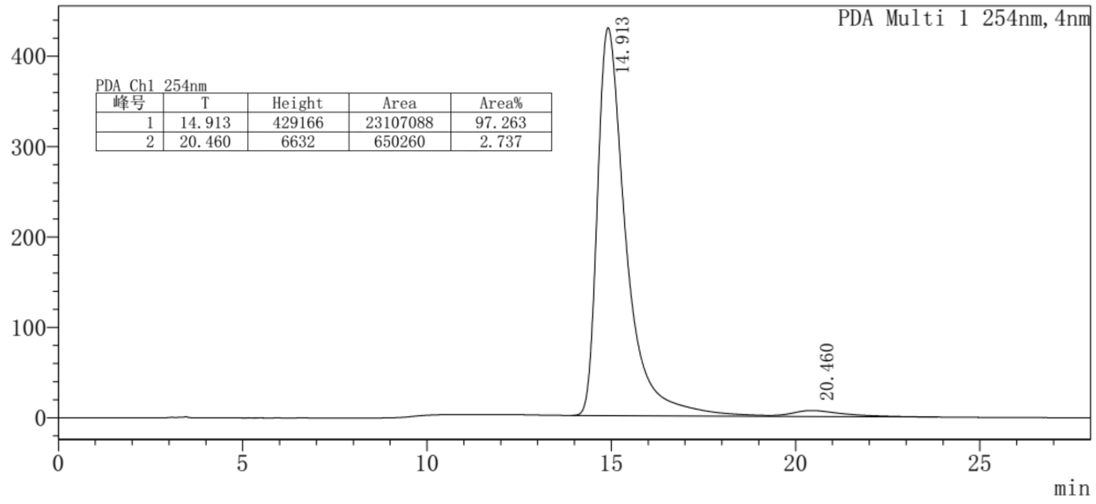

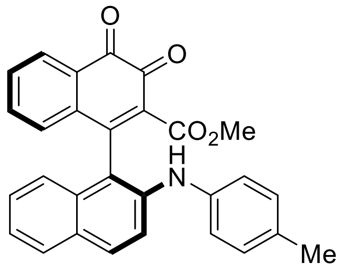

3b-12

(*S*)-Methyl 3,4-dioxo-2'-(p-tolylamino)-3,4-dihydro-[1,1'-binaphthalene]-2-carboxylate ((*S*)-3b-12)

According to the general procedure, **(*S*)-3b-12** was obtained in **95% yield** with **95% ee**.

^{1}H NMR (500 MHz, CDCl$_3$) δ 8.22 (dd, *J* = 7.6, 1.4 Hz, 1H), 7.83 (d, *J* = 9.0 Hz, 1H), 7.80 (dd, *J* = 7.9, 1.5 Hz, 1H), 7.55 (d, *J* = 9.2 Hz, 1H), 7.52-7.48 (m, 2H), 7.43 (td, *J* = 7.6, 1.4 Hz, 1H), 7.40-7.33 (m, 2H), 7.07 (d, *J* = 8.1 Hz, 2H), 6.95 (d, *J* = 8.3 Hz, 2H), 6.89 (dd, *J* = 7.9, 1.1 Hz, 1H), 6.00 (s, 1H), 3.46 (s, 3H), 2.29 (s, 3H).

^{13}C NMR (125 MHz, CDCl$_3$) δ 178.09, 176.47, 164.71, 152.28, 139.83, 139.74, 136.33, 135.97, 134.26, 132.10, 131.98, 131.82, 130.80, 130.72, 130.06, 129.57, 129.02, 128.38, 127.60, 124.20, 123.93, 119.79, 118.66, 116.52, 52.75, 20.85.

HRMS (ESI) calcd for [M+H] $C_{29}H_{21}NO_4$, m/z: 448.1543, found: 448.1531.

HPLC analysis: CHIRALCEL OD-H, hexane/isopropanol = 80/20, 1.0 mL/min, λ = 254 nm, t_R (major) = 12.5 min, t_R (minor) = 17.1 min, ee = 95%.

Supplementary Figure 51. Chiral HPLC spectrum of racemic **3b-12**

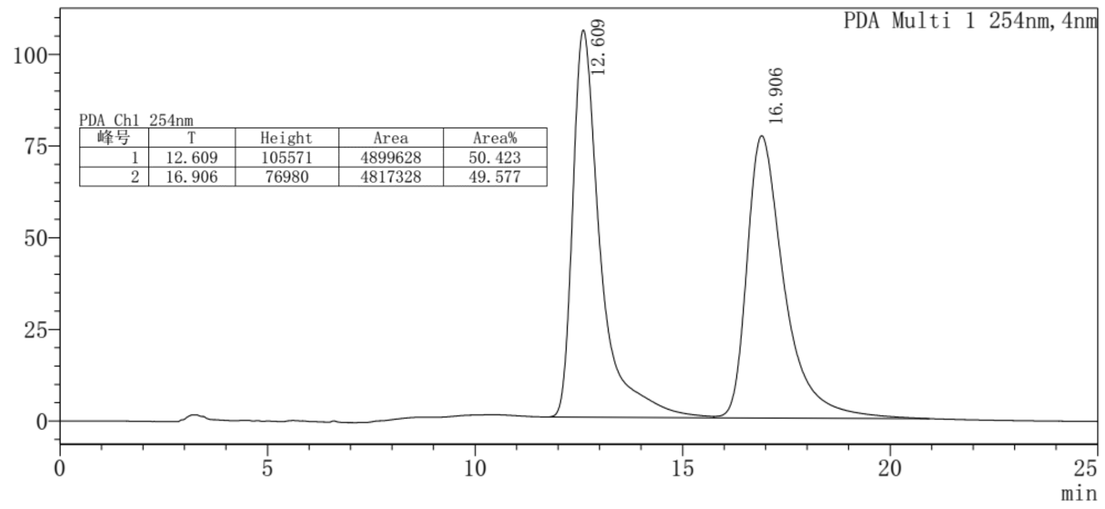

Supplementary Figure 52. Chiral HPLC spectrum of **(*S*)-3b-12**

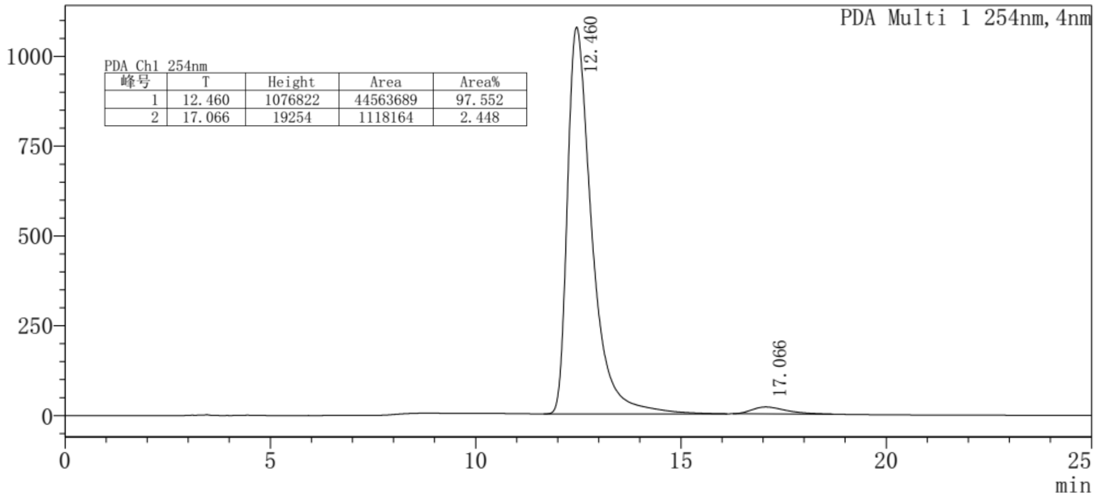

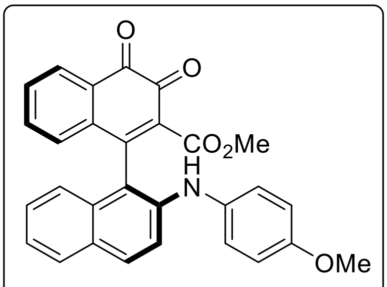

3b-13

(*S*)-Methyl 2'-((4-methoxyphenyl)amino)-3,4-dioxo-3,4-dihydro-[1,1'-binaphthalene]-2-carboxylate ((*S*)-3b-13)

According to the general procedure, **(*S*)-3b-13** was obtained in **92% yield** with **96% ee**.

^{1}H NMR (500 MHz, DMSO-d_6) δ 8.05 (dd, *J* = 7.5, 1.4 Hz, 1H), 7.78 (d, *J* = 9.1 Hz, 1H), 7.75 (dd, *J* = 8.2, 1.3 Hz, 1H), 7.58 (dd, *J* = 8.4, 1.1 Hz, 1H), 7.53 (td, *J* = 7.5, 1.2 Hz, 1H), 7.45 (td,

J = 7.6, 1.5 Hz, 1H), 7.28 (ddd, J = 8.3, 6.7, 1.4 Hz, 1H), 7.25-7.19 (m, 3H), 7.02-6.98 (m, 2H), 6.88-6.84 (m, 2H), 6.71 (dd, J = 7.8, 1.1 Hz, 1H), 5.72 (s, 1H), 3.68 (s, 3H), 3.25 (s, 3H).

^{13}C NMR (125 MHz, DMSO-d_6) δ 177.61, 176.24, 164.56, 155.44, 148.51, 140.79, 135.94, 135.13, 134.83, 134.44, 133.04, 132.26, 131.05, 129.87, 128.62, 128.28, 127.86, 127.03, 126.77, 124.15, 123.99, 122.55, 116.21, 114.47, 112.70, 55.24, 54.95, 51.65.

HRMS (ESI) calcd for [M+H] $C_{29}H_{21}NO_5$, m/z: 464.1493, found: 464.1477.

HPLC analysis: CHIRALCEL OD-H, hexane/isopropanol = 80/20, 1.0 mL/min, λ = 254 nm, t_R (major) = 17.9 min, t_R (minor) = 28.8 min, ee = 96%.

Supplementary Figure 53. Chiral HPLC spectrum of racemic **3b-13**

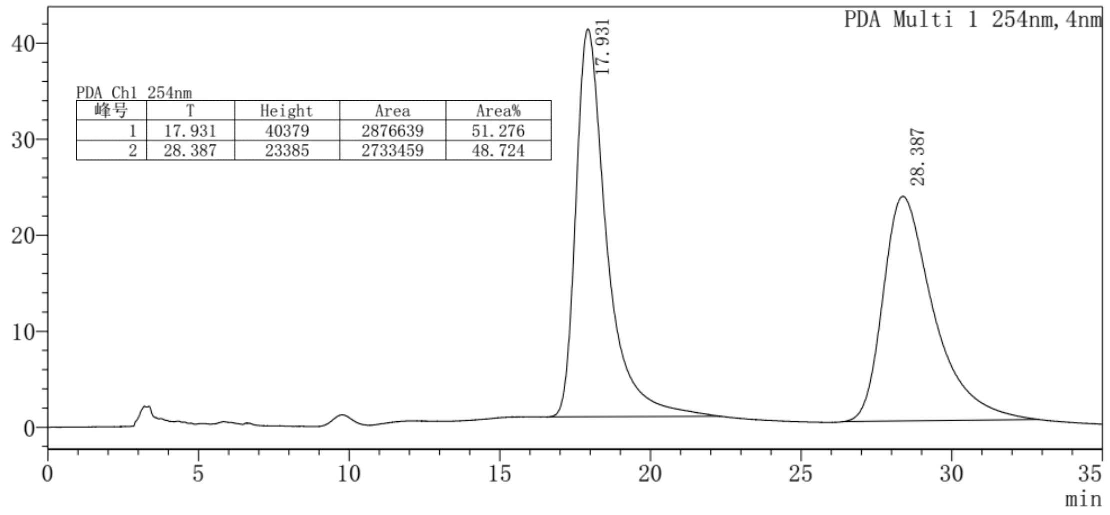

Supplementary Figure 54. Chiral HPLC spectrum of **(*S*)-3b-13**

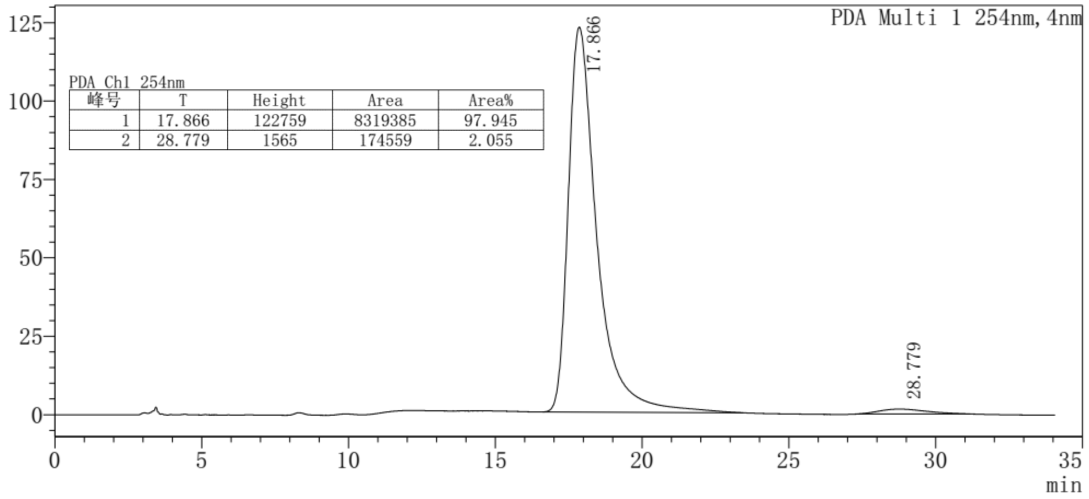

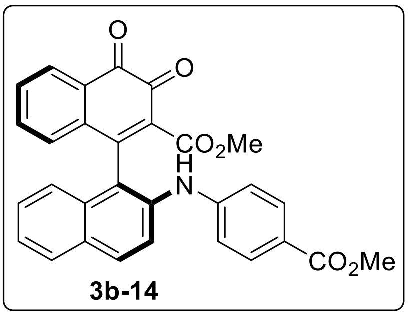

(*S*)-Methyl 2'-((4-(methoxycarbonyl)phenyl)amino)-3,4-dioxo-3,4-dihydro-[1,1'-binaphthalene]-2-carboxylate ((*S*)-3b-14)

According to the general procedure, **(*S*)-3b-14** was obtained in **88% yield** with **98% ee**.

^{1}H NMR (500 MHz, $CDCl_3$) δ 8.17 (dd, *J* = 7.6, 1.4 Hz, 1H), 7.95 (d, *J* = 8.9 Hz, 1H), 7.89-7.84 (m, 3H), 7.68 (d, *J* = 8.8 Hz, 1H), 7.56 (dd, *J* = 8.0, 1.5 Hz, 1H), 7.50-7.37 (m, 4H), 6.93 (d, *J* = 8.7 Hz, 2H), 6.80 (dd, *J* = 7.8, 1.1 Hz, 1H), 6.65 (s, 1H), 3.83 (s, 3H), 3.44 (s, 3H).

^{13}C NMR (125 MHz, $CDCl_3$) δ 177.88, 176.35, 166.94, 164.99, 151.80, 147.75, 136.99, 136.23, 135.26, 134.24, 132.17, 131.74, 131.62, 131.48, 131.03, 130.73, 130.38, 129.46, 128.48, 127.84, 125.54, 124.46, 121.86, 121.45, 121.37, 115.52, 52.87, 51.88.

HRMS (ESI) calcd for [M+H] $C_{30}H_{21}NO_6$, m/z: 492.1442, found: 492.1437.

HPLC analysis: CHIRALCEL OD-H, hexane/isopropanol = 60/40, 1.0 mL/min, λ = 254 nm, t_R (minor) = 13.4 min, t_R (major) = 25.1 min, ee = 98%.

Supplementary Figure 55. Chiral HPLC spectrum of racemic **3b-14**

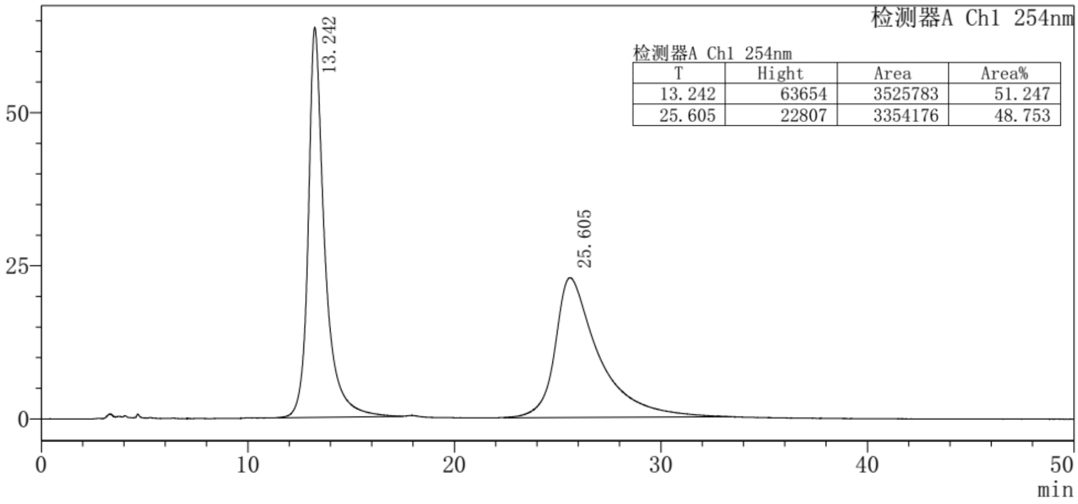

检测器A Ch1 254nm

T	Hight	Area	Area%
13.242	63654	3525783	51.247
25.605	22807	3354176	48.753

Supplementary Figure 56. Chiral HPLC spectrum of **(*S*)-3b-14**

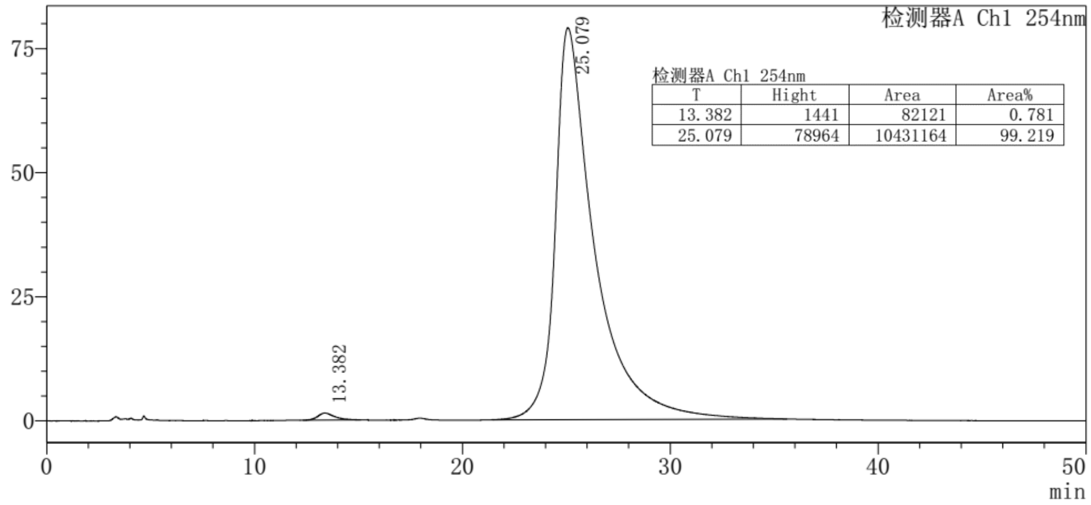

检测器A Ch1 254nm

T	Hight	Area	Area%
13.382	1441	82121	0.781
25.079	78964	10431164	99.219

General Procedure for Asymmetric Syntheses of Arylquinones (*R*)-3c

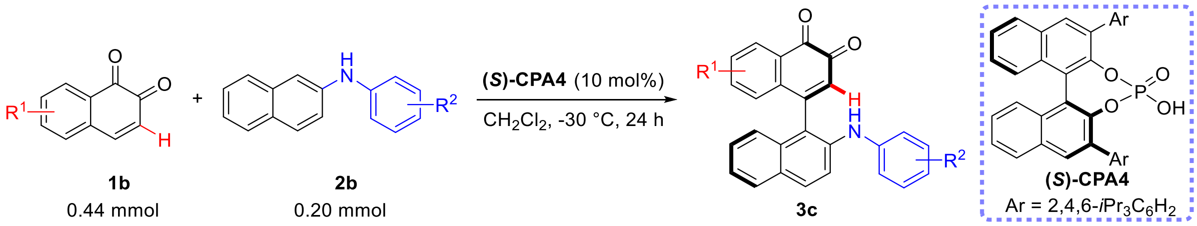

To a mixture of (*S*)-**CPA4** (10 mol%) and nucleophile **2b** (0.20 mmol) in 10 mL Schlenk tube was added CH_2Cl_2 (4.0 mL) under Ar atmosphere. The reaction mixture was stirred for 15 min at -30 ℃ before *o*-naphthoquinone **1b** (0.44 mmol) was added under Ar atmosphere. Then the reaction was stirred vigorously at -30 ℃ until the nucleophile completely consumed (monitored by thin-layer chromatography). Typical reaction time was about 24 hours. After that, the resulting mixture was concentrated under reduced pressure and purified by flash chromatography on silica gel (eluent: PE/EA = 6/1) to afford the corresponding axially chiral arylquinones (*R*)-**3c**.

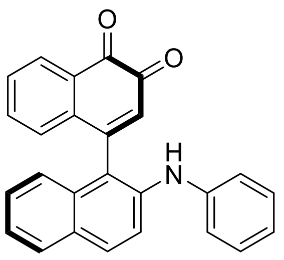

3c-1

(*R*)-2'-(Phenylamino)-[1,1'-binaphthalene]-3,4-dione ((*R*)-3c-1)

According to the general procedure, **(*R*)-3c-1** was obtained in **63% yield** with **93% ee**.

^{1}H NMR (400 MHz, DMSO-*d*$_6$) δ 8.07 (dd, *J* = 7.6, 1.6 Hz, 1H), 7.92 (d, *J* = 9.2 Hz, 1H), 7.87 (dd, *J* = 7.6, 2.0 Hz, 1H), 7.76 (dd, *J* = 8.0, 1.2 Hz, 1H), 7.66-7.48 (m, 3H), 7.47-7.43 (m, 1H), 7.41-7.29 (m, 2H), 7.26-7.21 (m, 2H), 7.07 (dd, *J* = 8.8, 1.2 Hz, 2H), 6.94-6.90 (m, 1H), 6.79 (dd, *J* = 8.0, 1.2 Hz, 1H), 6.45 (s, 1H);

^{13}C NMR (100 MHz, DMSO-*d*$_6$) δ 179.55, 179.38, 150.88, 142.87, 138.82, 135.58, 134.91, 132.89, 132.52, 131.83, 130.41, 129.54, 129.16, 128.71, 128.29, 128.15, 127.37, 127.03, 124.19, 123.42, 121.62, 119.58, 118.23.

HRMS (ESI) calcd for [M+H] $C_{26}H_{18}NO_2$, m/z: 376.1332, found: 376.1330.

HPLC analysis: CHIRALCEL IA, hexane/isopropanol = 70/30, 1.0 mL/min, λ = 254 nm, t_R

(minor) = 5.7 min, t_R (major) = 7.4 min, ee = 93%.

Supplementary Figure 57. Chiral HPLC spectrum of racemic **3c-1**

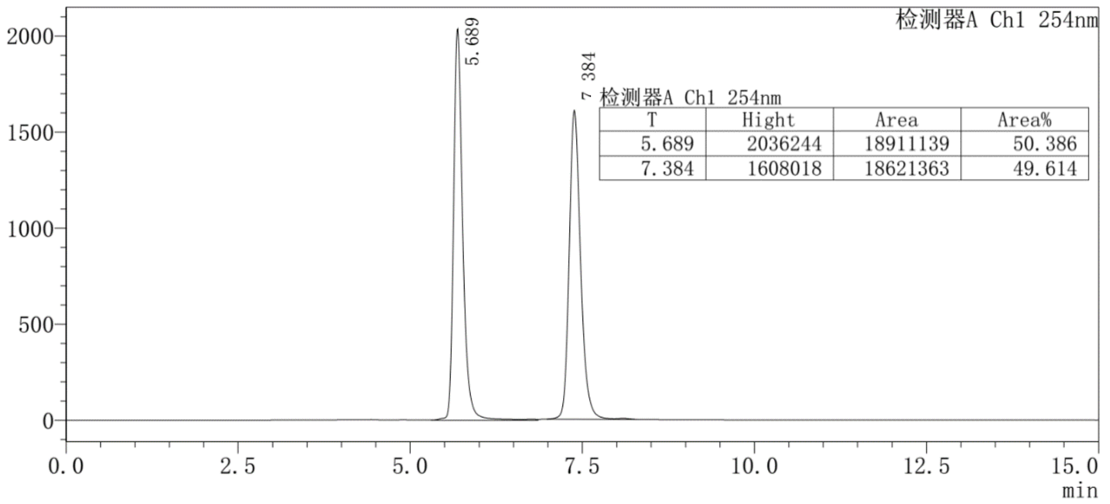

Supplementary Figure 58. Chiral HPLC spectrum of **(*R*)-3c-1**

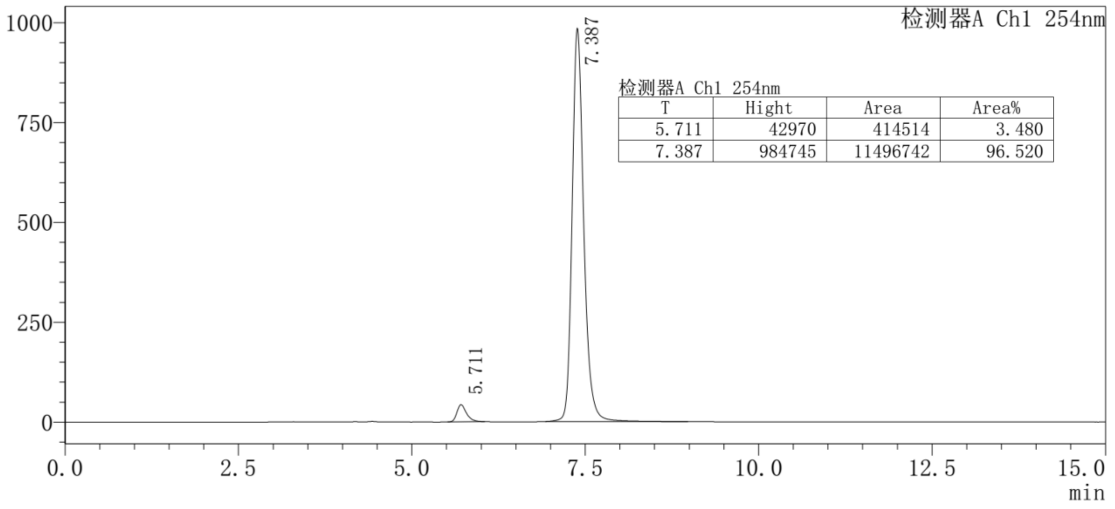

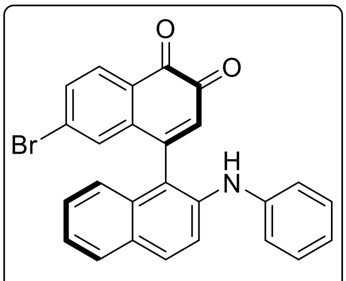

3c-2

(*R*)-7-Bromo-2'-(phenylamino)-[1,1'-binaphthalene]-3,4-dione ((*R*)-3c-2)

According to the general procedure, **(*R*)-3c-2** was obtained in **57% yield** with **90% ee**.

^{1}H NMR (400 MHz, DMSO-d_6) δ 7.89 (d, *J* = 8.2 Hz, 1H), 7.85 (d, *J* = 9.1 Hz, 1H), 7.83-7.78 (m, 1H), 7.73 (dd, *J* = 8.3, 1.2 Hz, 1H), 7.63 (dd, *J* = 8.2, 1.9 Hz, 1H), 7.50-7.39 (m,

2H), 7.35-7.31 (m, 1H), 7.29-7.25 (m, 1H), 7.20-7.08 (m, 2H), 7.02-6.91 (m, 2H), 6.86-6.82 (m, 1H), 6.77 (d, *J* = 1.9 Hz, 1H), 6.44 (s, 1H).

^{13}C NMR (100 MHz, DMSO-d_6) δ 179.39, 178.95, 149.31, 143.20, 139.30, 137.74, 133.71, 133.46, 132.82, 132.43, 130.84, 130.34, 129.72, 129.65, 129.08, 128.82, 128.68, 127.64, 124.43, 124.06, 122.09, 119.66, 118.88, 118.13.

HRMS (ESI) calcd for [M+H] $C_{26}H_{17}NO_2{}^{79}Br$, m/z: 454.0437, found: 454.0440; $C_{26}H_{17}NO_2{}^{81}Br$, m/z: 456.0417, found: 456.0420.

HPLC analysis: CHIRALCEL IA, hexane/isopropanol = 70/30, 1.0 mL/min, λ = 254 nm, t_R (minor) = 8.6 min, t_R (major) = 12.5 min, ee = 90%.

Supplementary Figure 59. Chiral HPLC spectrum of racemic **3c-2**

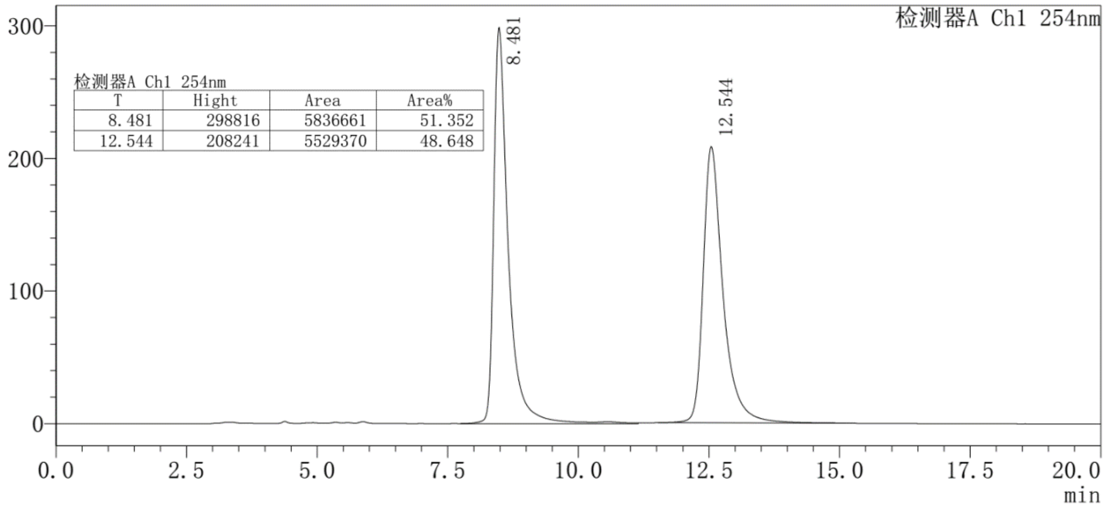

Supplementary Figure 60. Chiral HPLC spectrum of **(*R*)-3c-2**

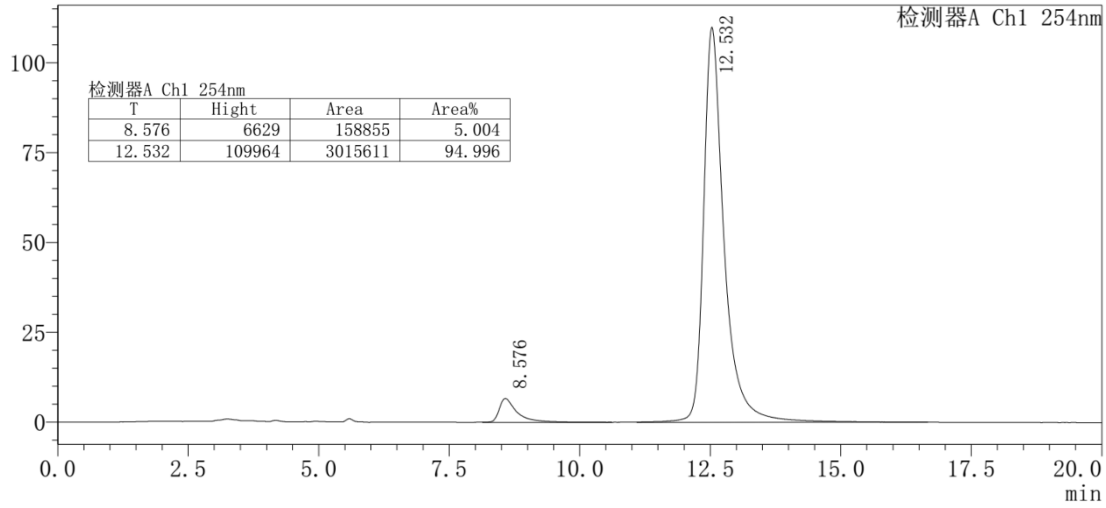

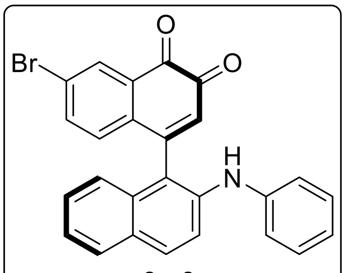

3c-3

(*R*)-6-Bromo-2'-(phenylamino)-[1,1'-binaphthalene]-3,4-dione ((*R*)-3c-3)

According to the general procedure, **(*R*)-3c-3** was obtained in **59% yield** with **91% ee**.

^{1}H NMR (400 MHz, DMSO-d_6) δ 8.11 (s, 1H), 7.92-7.86 (m, 2H), 7.75 (d, *J* = 8.4 Hz, 1H), 7.65 (d, *J* = 8.4 Hz, 1H), 7.52 (d, *J* = 9.0 Hz, 1H), 7.46 (s, 1H), 7.40-7.31 (m, 2H), 7.26-7.23 (m, 2H), 7.07 (d, *J* = 7.9 Hz, 2H), 6.95-6.91 (m, 1H), 6.71 (d, *J* = 8.4 Hz, 1H), 6.49 (s, 1H);

^{13}C NMR (100 MHz, DMSO-d_6) δ 179.25, 178.57, 150.16, 143.08, 139.31, 137.52, 135.00, 132.87, 132.62, 131.10, 130.10, 129.72, 129.61, 128.65, 128.58, 127.50, 124.46, 124.27, 123.86, 122.21, 120.15, 118.45, 118.06, 79.62.

HRMS (ESI) calcd for [M+H] $C_{26}H_{17}NO_2{}^{79}Br$, m/z: 454.0437, found: 454.0435; $C_{26}H_{17}NO_2{}^{81}Br$, m/z: 456.0417, found: 456.0418.

HPLC analysis: CHIRALCEL IB, hexane/isopropanol = 80/20, 1.0 mL/min, λ = 254 nm, t_R (minor) = 8.2 min, t_R (major) = 14.5 min, ee = 91%.

Supplementary Figure 61. Chiral HPLC spectrum of racemic **3c-3**

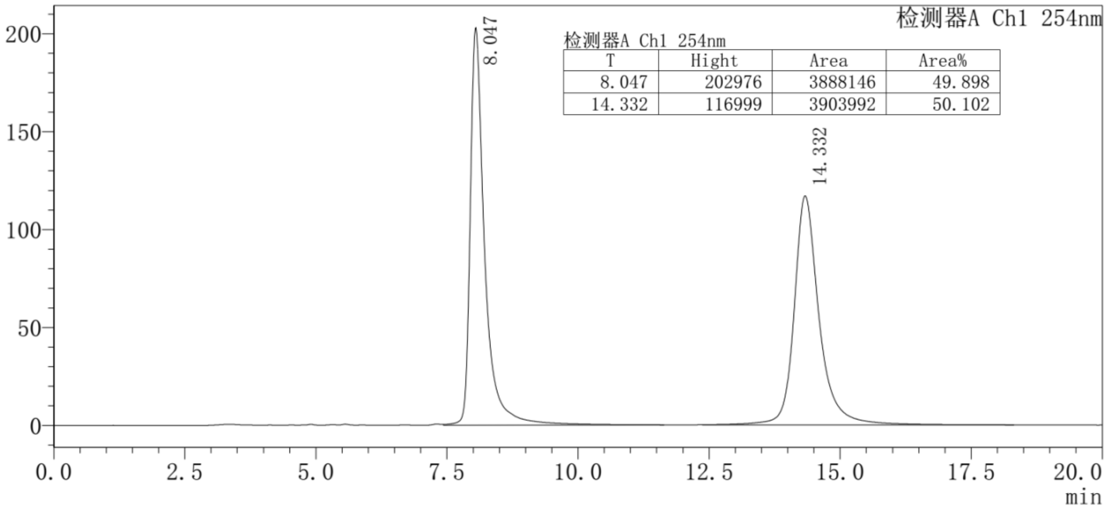

Supplementary Figure 62. Chiral HPLC spectrum of **(*R*)-3c-3**

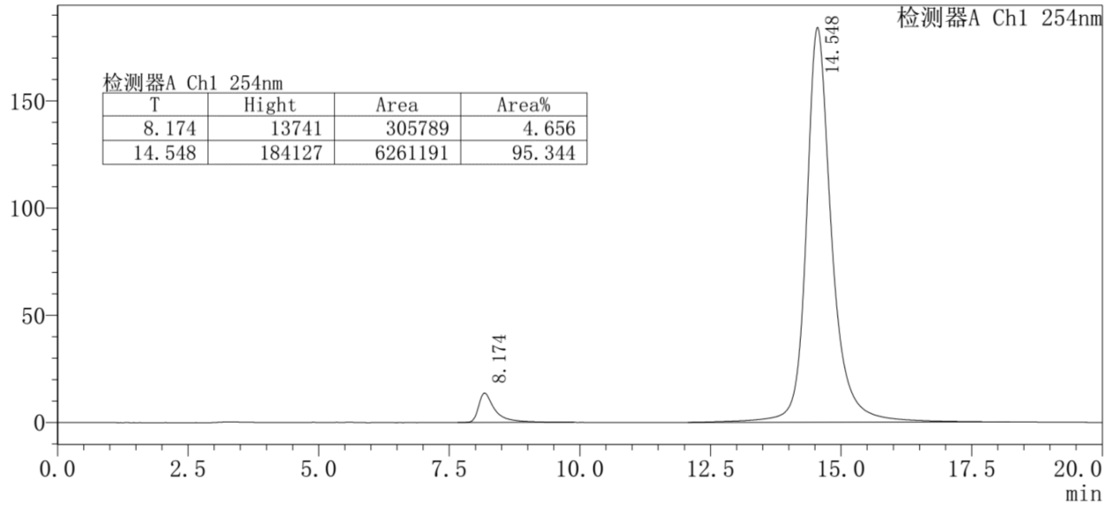

T	Hight	Area	Area%
8.174	13741	305789	4.656
14.548	184127	6261191	95.344

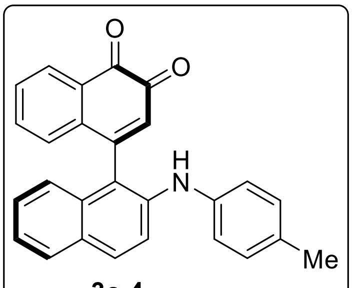

3c-4

(*R*)-2'-(*p*-Tolylamino)-[1,1'-binaphthalene]-3,4-dione ((*R*)-3c-4)

According to the general procedure, **(*R*)-3c-4** was obtained in **58% yield** with **95% ee**.

^{1}H NMR (400 MHz, DMSO-d_6) δ 8.07 (dd, *J* = 7.6, 1.5 Hz, 1H), 7.94-7.79 (m, 2H), 7.71 (d, *J* = 8.3 Hz, 1H), 7.62-7.49 (m, 1H), 7.49-7.42 (m, 2H), 7.40-7.24 (m, 3H), 7.16-6.91 (m, 4H), 6.86-6.74 (m, 1H), 6.43 (s, 1H), 2.22 (s, 3H).

^{13}C NMR (100 MHz, DMSO-d_6) δ 179.96, 179.79, 151.39, 140.48, 139.85, 135.98, 135.30, 133.35, 132.94, 132.28, 131.40, 130.80, 130.03, 129.90, 129.07, 128.52, 128.33, 127.75, 127.39, 124.39, 123.49, 120.90, 118.05, 117.53, 79.62, 20.82.

HRMS (ESI) calcd for [M+H] $C_{27}H_{20}NO_2$, m/z: 390.1489, found: 390.1487.

HPLC analysis: CHIRALCEL IA, hexane/isopropanol = 70/30, 1.0 mL/min, λ = 254 nm, t_R (minor) = 6.6 min, t_R (major) = 8.2 min, ee = 95%.

Supplementary Figure 63. Chiral HPLC spectrum of racemic **3c-4**

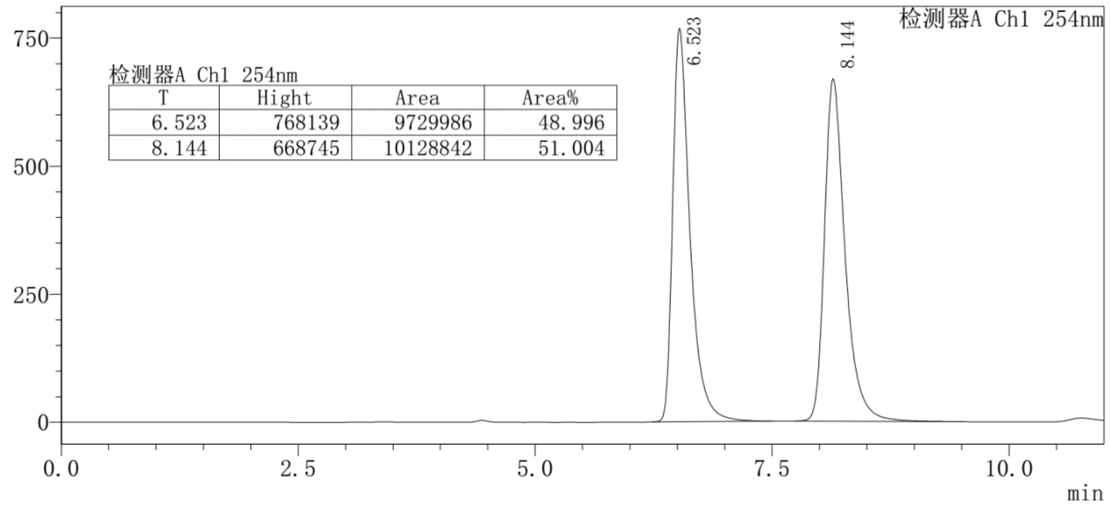

Supplementary Figure 64. Chiral HPLC spectrum of **(*R*)-3c-4**

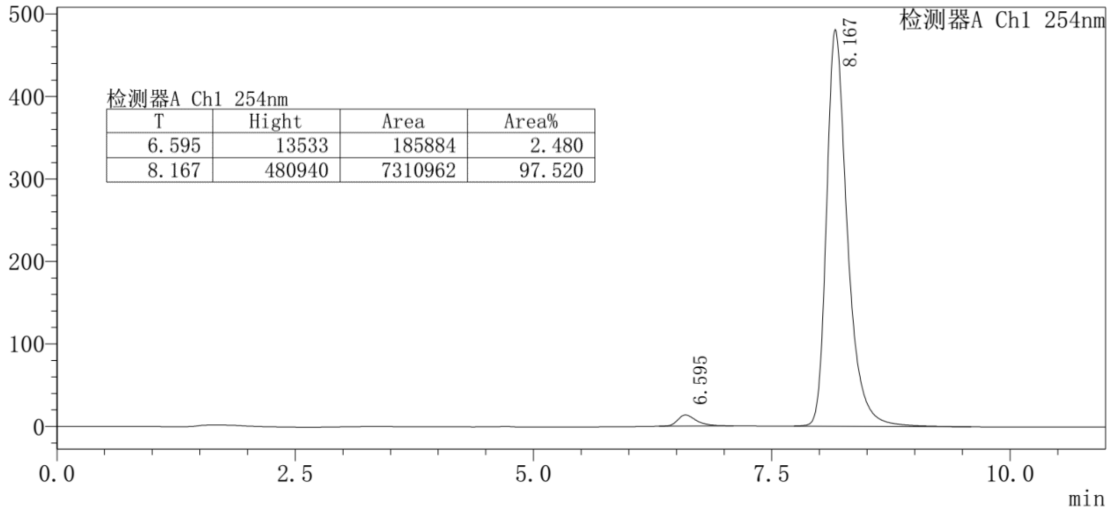

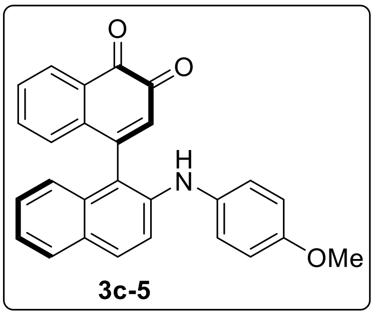

(*R*)-2'-((4-Methoxyphenyl)amino)-[1,1'-binaphthalene]-3,4-dione ((*R*)-3c-5)

According to the general procedure, **(*R*)-3c-5** was obtained in **56% yield** with **94% ee**.

^{1}H NMR (400 MHz, DMSO-d_6) δ 7.95 (dd, *J* = 7.5, 1.5 Hz, 1H), 7.73-7.67 (m, 2H), 7.55 (d, *J* = 8.4 Hz, 1H), 7.43-7.38 (m, 1H), 7.37-7.32 (m, 1H), 7.24-7.10 (m, 5H), 6.90 (d, *J* = 8.9 Hz, 2H), 6.74 (d, *J* = 8.9 Hz, 2H), 6.68 (dd, *J* = 7.7, 1.2 Hz, 1H), 3.57 (s, 3H);

^{13}C NMR (100 MHz, DMSO-*d*$_6$) δ 179.68, 179.44, 155.31, 151.24, 140.45, 135.64, 135.42, 134.96, 133.06, 132.60, 131.99, 130.46, 129.60, 128.72, 128.17, 127.56, 127.43, 127.04, 123.84, 123.46, 122.79, 116.92, 115.85, 114.57, 55.31.

HRMS (ESI) calcd for [M+H] $C_{27}H_{20}NO_3$, m/z: 406.1438, found: 406.1436.

HPLC analysis: CHIRALCEL IA, hexane/isopropanol = 60/40, 1.0 mL/min, λ = 254 nm, t_R (minor) = 6.9 min, t_R (major) = 8.1 min, ee = 94%.

Supplementary Figure 65. Chiral HPLC spectrum of racemic **3c-5**

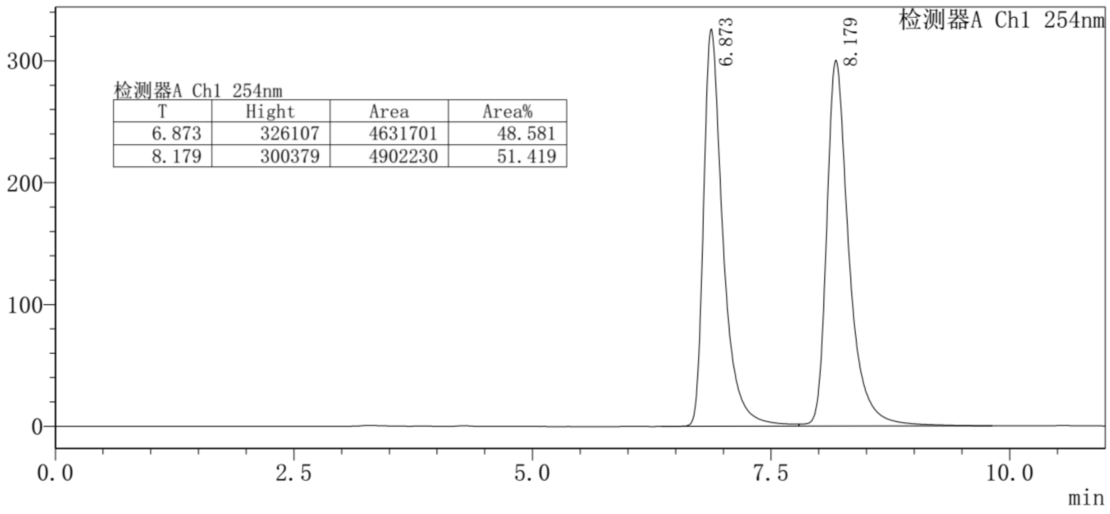

检测器A Ch1 254nm

T	Hight	Area	Area%
6.873	326107	4631701	48.581
8.179	300379	4902230	51.419

Supplementary Figure 66. Chiral HPLC spectrum of **(*R*)-3c-5**

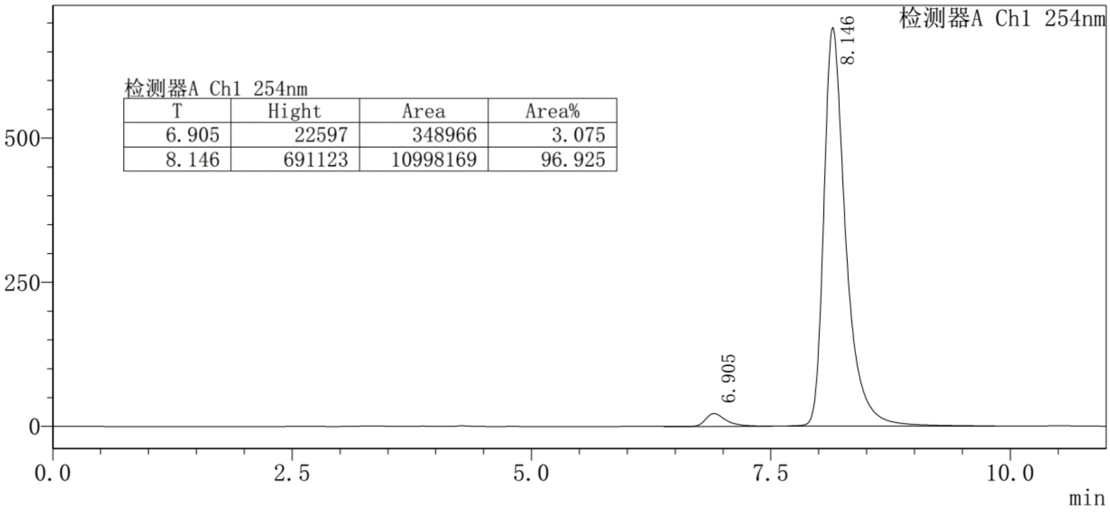

检测器A Ch1 254nm

T	Hight	Area	Area%
6.905	22597	348966	3.075
8.146	691123	10998169	96.925

General Procedure for Asymmetric Syntheses of Arylquinones (*S*)-3d

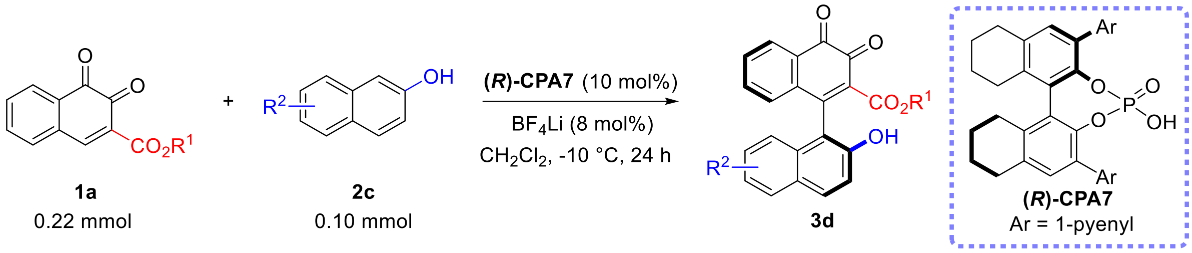

To a mixture of (*R*)-**CPA7** (10 mol%) and BF_4Li (8 mol%) in 10 mL Schlenk tube was added CH_2Cl_2 (4.0 mL) under Ar atmosphere. The reaction mixture was stirred for 1 hour at room temperature. After that, *o*-naphthoquinone **1a** (0.22 mmol) and **2c** (0.10 mmol) were added and the mixture was then stirred vigorously at -10 ℃ until the nucleophile completely consumed (monitored by thin-layer chromatography). Typical reaction time was about 24 hours. The resulting mixture was concentrated under reduced pressure and purified by flash chromatography on silica gel (eluent: PE/EA = 6/1) to afford the corresponding axially chiral arylquinones (*S*)-**3d**.

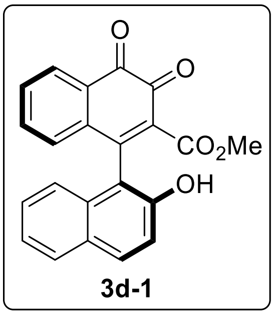

(*S*)-Methyl 2'-hydroxy-3,4-dioxo-3,4-dihydro-[1,1'-binaphthalene]-2-carboxylate ((*S*)-3d-1)

According to the general procedure, **(*S*)-3d-1** was obtained in **90% yield** with **92% ee**.

^{1}H NMR (400 MHz, DMSO-*d*$_6$) δ 9.86 (s, 1H), 8.11 (dd, *J* = 7.6, 1.4 Hz, 1H), 7.93 (d, *J* = 9.0 Hz, 1H), 7.86 (dd, *J* = 7.8, 1.6 Hz, 1H), 7.72 (d, *J* = 8.1 Hz, 1H), 7.62-7.57 (m, 1H), 7.54-7.49 (m, 1H), 7.38-7.26 (m, 4H), 6.70 (dd, *J* = 7.8, 1.2 Hz, 1H), 3.25 (s, 3H);

^{13}C NMR (100 MHz, DMSO-*d*$_6$) δ 177.76, 176.84, 164.78, 152.24, 150.03, 135.66, 135.06, 134.88, 132.58, 132.44, 131.65, 131.24, 129.47, 129.37, 128.30, 127.88, 127.10, 125.06, 123.58, 118.60, 113.50, 52.04.

HRMS (ESI) calcd for [M-H] $C_{22}H_{13}O_5$, m/z: 357.0769, found: 357.0768.

HPLC analysis: CHIRALCEL IB, hexane/isopropanol = 80/20, 1.0 mL/min, λ = 254 nm, t_R (minor) = 5.9 min, t_R (major) = 8.8 min, ee = 92%.

Supplementary Figure 67. Chiral HPLC spectrum of racemic **3d-1**

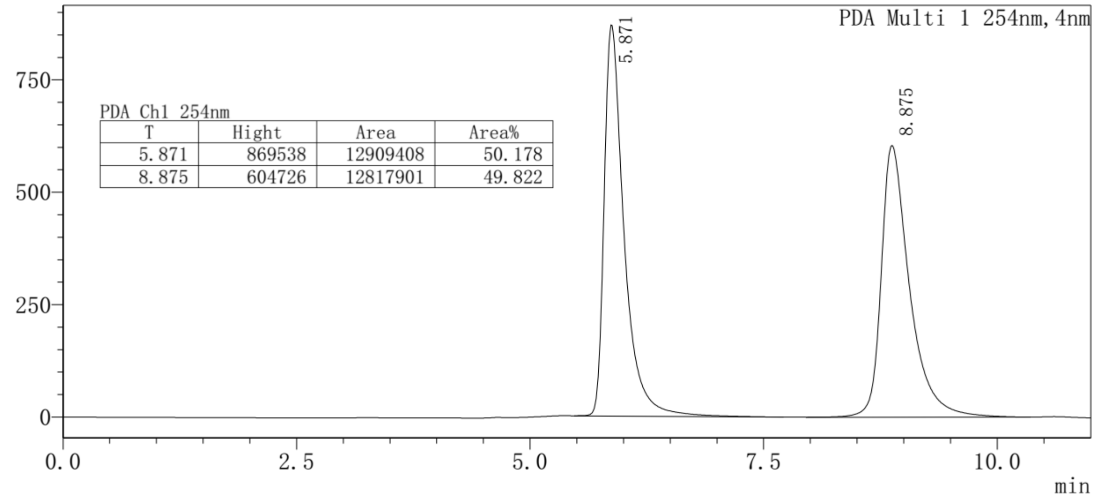

Supplementary Figure 68. Chiral HPLC spectrum of **(*S*)-3d-1**

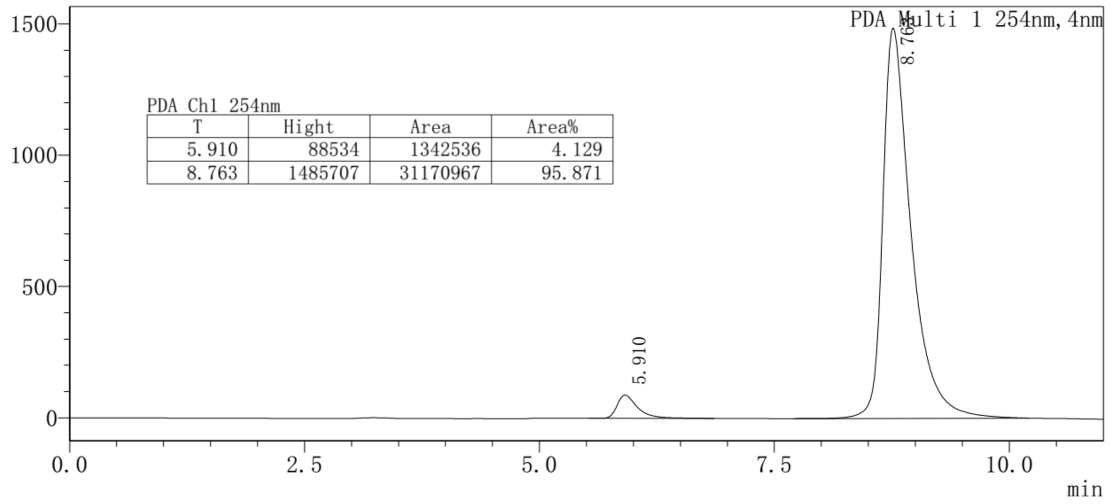

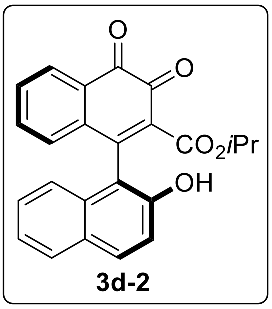

(*S*)-Isopropyl 2'-hydroxy-3,4-dioxo-3,4-dihydro-[1,1'-binaphthalene]-2-carboxylate ((S)-3d-2)

According to the general procedure, **(*S*)-3d-2** was obtained in **93% yield** with **94% ee**.

^{1}H NMR (400 MHz, DMSO-d_6) δ 9.85 (s, 1H), 8.09 (dd, J = 7.5, 1.4 Hz, 1H), 7.93 (d, J = 8.9 Hz, 1H), 7.86 (dd, J = 8.0, 1.5 Hz, 1H), 7.72 (d, J = 8.2 Hz, 1H), 7.61-7.56 (m, 1H), 7.54-7.49 (m, 1H), 7.39-7.26 (m, 3H), 6.74 (dd, J = 7.7, 1.1 Hz, 1H), 4.59-4.49 (m, 1H), 0.66 (d, J = 6.2 Hz, 3H), 0.42 (d, J = 6.2 Hz, 3H).

^{13}C NMR (100 MHz, DMSO-d_6) δ 177.85, 176.93, 163.40, 152.36, 149.71, 135.65, 135.15, 134.95, 132.84, 132.38, 131.56, 131.08, 129.37, 129.30, 128.17, 127.94, 126.95, 125.32, 123.52, 118.57, 113.51, 68.17, 21.23, 20.73.

HRMS (ESI) calcd for [M-H] $C_{24}H_{17}O_5$, m/z: 385.1082, found: 385.1081.

HPLC analysis: CHIRALCEL ID, hexane/isopropanol = 60/40, 1.0 mL/min, λ = 254 nm, t_R (minor) = 10.8 min, t_R (major) = 17.0 min, ee = 94%.

Supplementary Figure 69. Chiral HPLC spectrum of racemic **3d-2**

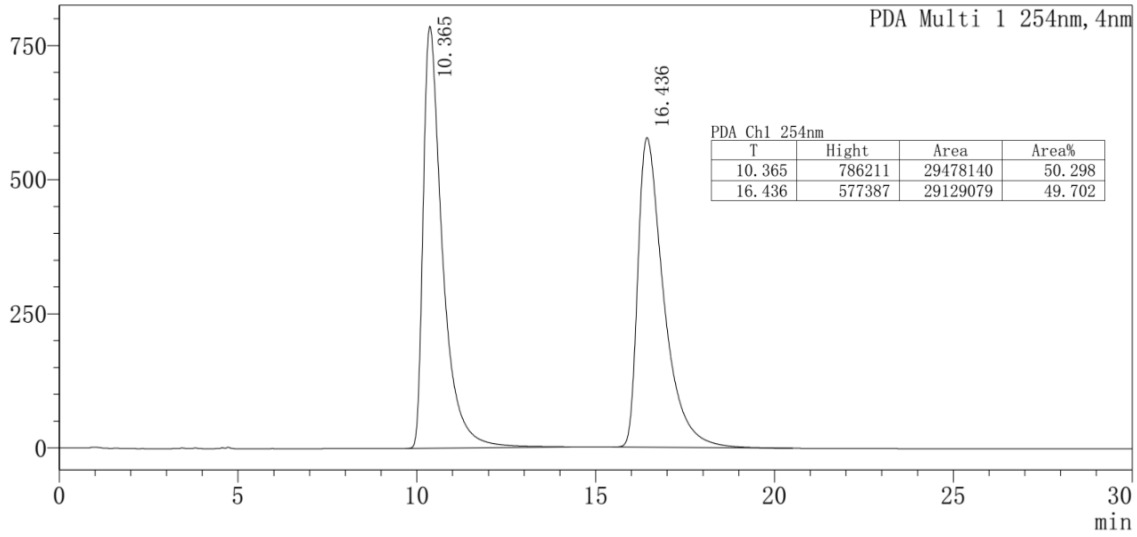

Supplementary Figure 70. Chiral HPLC spectrum of **(*S*)-3d-2**

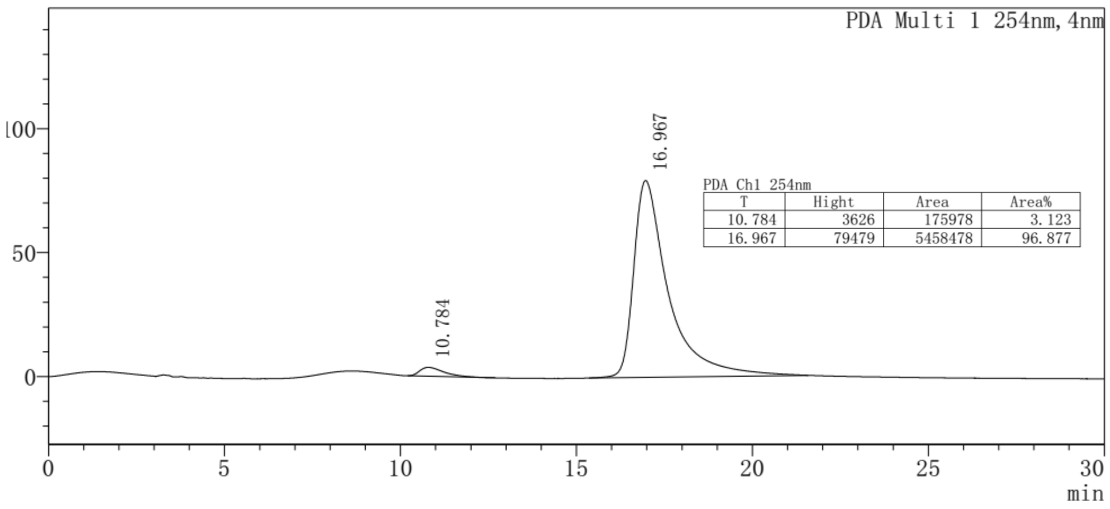

T	Hight	Area	Area%
10.784	3626	175978	3.123
16.967	79479	5458478	96.877

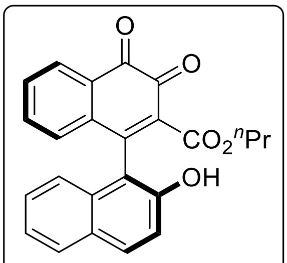

3d-3

(*S*)-Propyl 2'-hydroxy-3,4-dioxo-3,4-dihydro-[1,1'-binaphthalene]-2-carboxylate ((*S*)-3d-3)

According to the general procedure, **(*S*)-3d-3** was obtained in **91% yield** with **93% ee**.

^{1}H NMR (400 MHz, DMSO-d_6) δ 9.83 (s, 1H), 8.09 (dd, *J* = 7.6, 1.4 Hz, 1H), 7.92 (d, *J* = 9.0 Hz, 1H), 7.85 (dd, *J* = 7.7, 1.6 Hz, 1H), 7.75-7.68 (m, 1H), 7.62-7.57 (m, 1H), 7.55-7.50 (m, 1H), 7.39-7.28 (m, 2H), 7.26 (d, *J* = 8.9 Hz, 1H), 6.70 (dd, *J* = 7.7, 1.1 Hz, 1H), 3.68-3.58 (m, 2H), 0.98-0.82 (m, 2H), 0.44-0.39 (m, 3H).

^{13}C NMR (100 MHz, DMSO-d_6) δ 177.79, 176.90, 164.17, 152.26, 149.68, 135.66, 135.17, 134.92, 132.73, 132.40, 131.58, 131.13, 129.39, 129.35, 128.23, 127.92, 127.06, 125.17, 123.53, 118.57, 113.49, 66.24, 21.41, 10.30.

HRMS (ESI) calcd for [M-H] $C_{24}H_{17}O_5$, m/z: 385.1082, found: 385.1081.

HPLC analysis: CHIRALCEL AD-H, hexane/isopropanol = 60/40, 1.0 mL/min, λ = 254 nm, t_R (minor) = 5.5 min, t_R (major) = 8.8 min, ee = 93%.

Supplementary Figure 71. Chiral HPLC spectrum of racemic **3d-3**

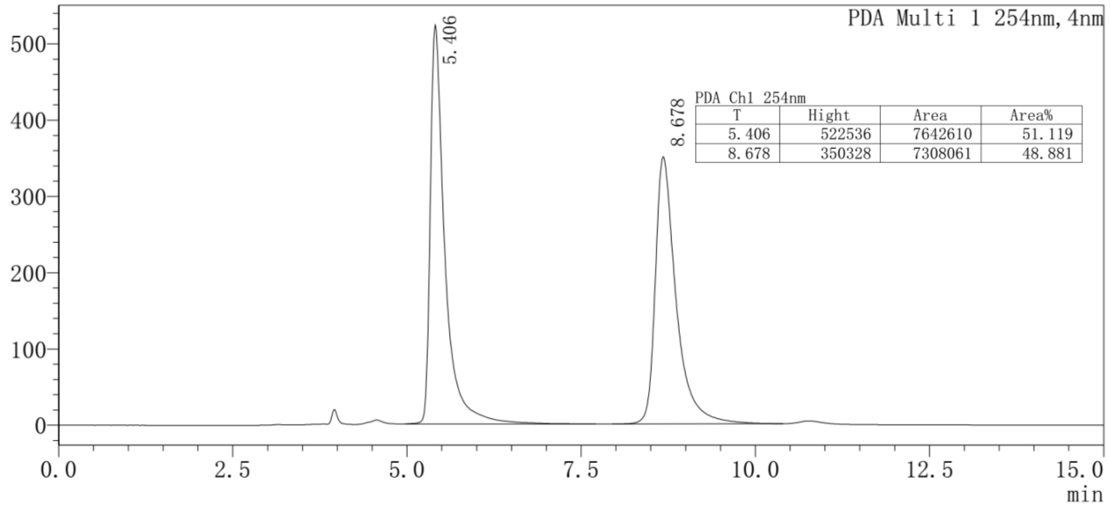

Supplementary Figure 72. Chiral HPLC spectrum of **(*S*)-3d-3**

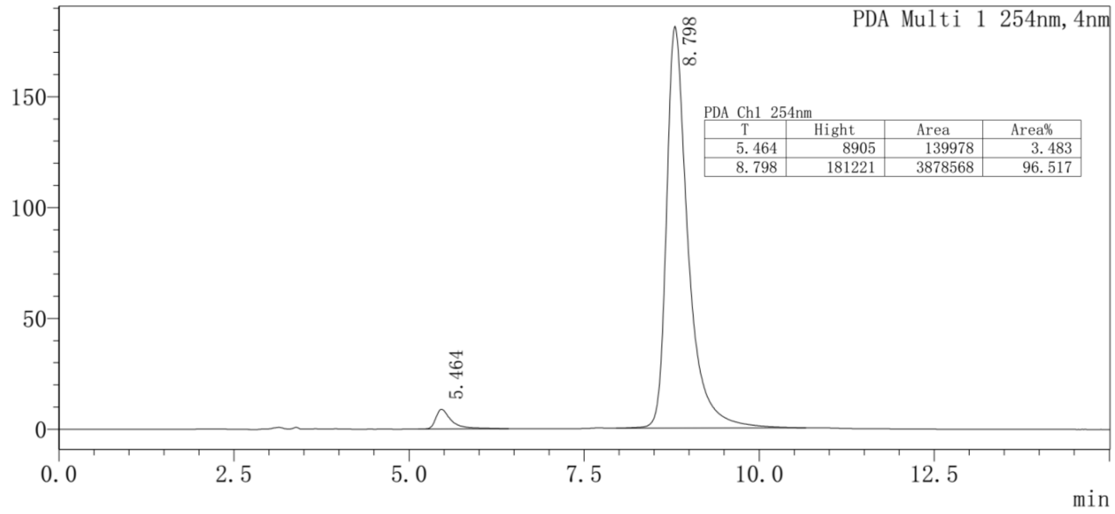

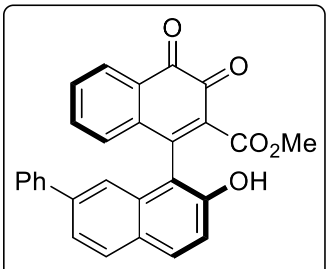

3d-4

(*S*)-Methyl 2'-hydroxy-3,4-dioxo-7'-phenyl-3,4-dihydro-[1,1'-binaphthalene]-2-carboxylate ((*S*)-3d-4)

According to the general procedure, **(*S*)-3d-4** was obtained in **91% yield** with **89% ee**.

^{1}H NMR (400 MHz, DMSO-d_6) δ 9.90 (s, 1H), 8.11 (dd, *J* = 7.6, 1.5 Hz, 1H), 8.02-7.85 (m, 3H), 7.70-7.61 (m, 3H), 7.56-7.61 (m, 1H), 7.55-7.49 (m, 1H), 7.45-7.41 (m, 2H), 7.36-7.25 (m, 2H), 6.78 (dd, *J* = 7.8, 1.3 Hz, 1H), 3.26 (s, 3H).

^{13}C NMR (100 MHz, DMSO-d_6) δ 177.78, 176.81, 164.98, 152.75, 149.53, 140.69, 138.90, 135.52, 135.26, 134.89, 132.75, 132.63, 131.56, 131.05, 129.39, 129.27, 129.07, 127.88, 127.74, 127.23, 122.97, 122.41, 118.66, 113.93, 52.07.

HRMS (ESI) calcd for [M-H] $C_{28}H_{17}O_5$, m/z: 433.1082, found: 433.1077.

HPLC analysis: CHIRALCEL AD-H, hexane/isopropanol = 60/40, 1.0 mL/min, λ = 254 nm, t_R (major) = 4.8 min, t_R (minor) = 6.3 min, ee = 89%.

Supplementary Figure 73. Chiral HPLC spectrum of racemic **3d-4**

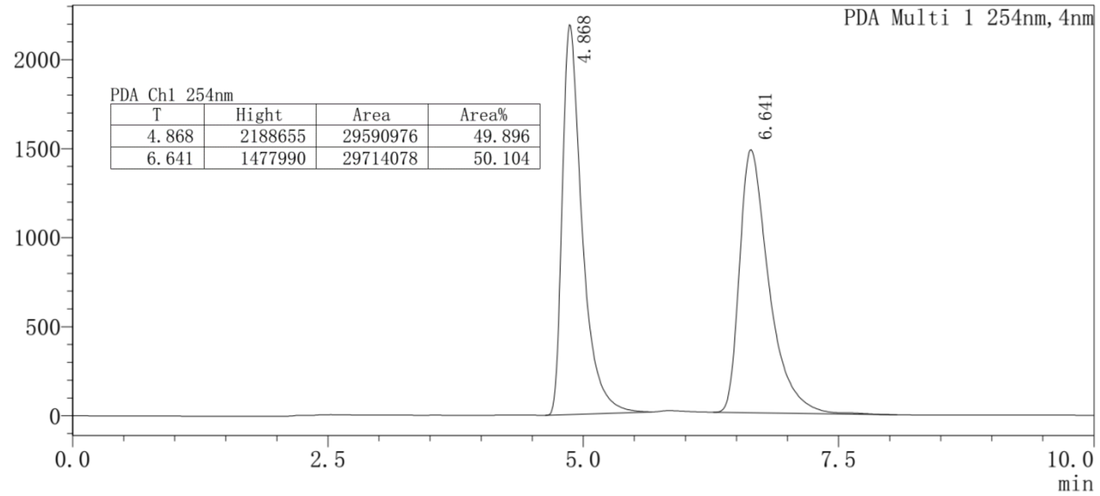

Supplementary Figure 74. Chiral HPLC spectrum of **(*S*)-3d-4**

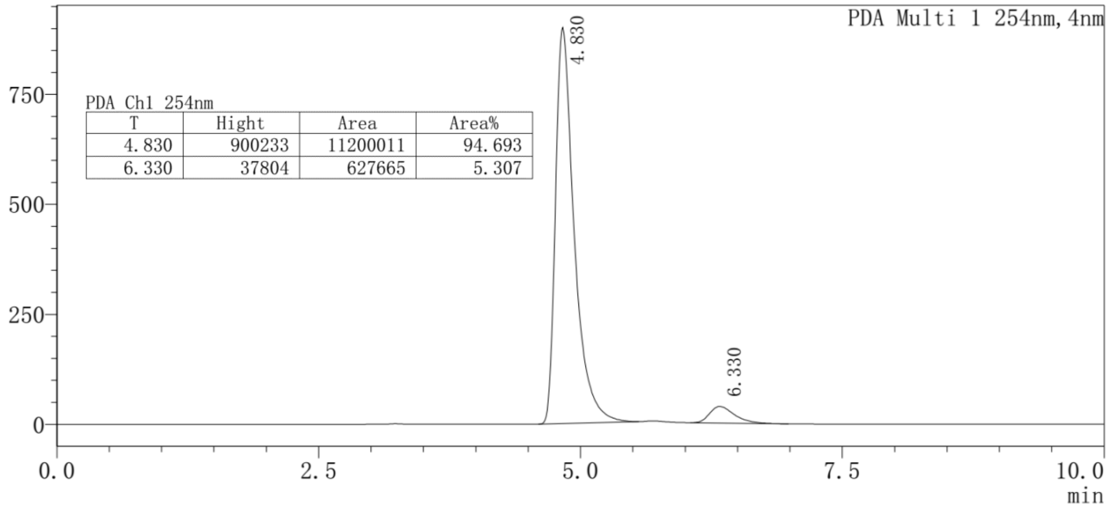

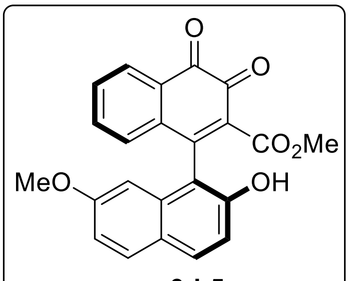

3d-5

(*S*)-Methyl 2'-hydroxy-7'-methoxy-3,4-dioxo-3,4-dihydro-[1,1'-binaphthalene]-2-carboxylate ((*S*)-3d-5)

According to the general procedure, **(*S*)-3d-5** was obtained in **90% yield** with **89% ee**.

^{1}H NMR (400 MHz, DMSO-*d*$_6$) δ 9.77 (s, 1H), 8.11 (dd, *J* = 7.5, 1.5 Hz, 1H), 7.83 (d, *J* = 8.9 Hz, 1H), 7.77 (d, *J* = 8.9 Hz, 1H), 7.62-7.57 (m, 1H), 7.56-7.51 (m, 1H), 7.10 (d, *J* = 8.9 Hz, 1H), 7.06 (d, *J* = 2.5 Hz, 1H), 6.99 (dd, *J* = 8.9, 2.5 Hz, 1H), 6.75 (dd, *J* = 7.7, 1.3 Hz, 1H), 3.70 (s, 3H), 3.27 (s, 3H).

^{13}C NMR (100 MHz, DMSO-*d*$_6$) δ 177.84, 176.90, 164.87, 158.45, 152.87, 150.00, 135.58, 135.04, 134.75, 133.88, 132.56, 131.55, 130.98, 129.95, 129.40, 129.26, 123.33, 115.82, 115.25, 112.86, 104.66, 55.88, 52.04.

HRMS (ESI) calcd for [M+H] $C_{23}H_{15}O_6$, m/z: 387.0874, found: 387.0874.

HPLC analysis: CHIRALCEL IB, hexane/isopropanol = 60/40, 1.0 mL/min, λ = 254 nm, t_R (minor) = 6.4 min, t_R (major) = 7.6 min, ee = 89%.

Supplementary Figure 75. Chiral HPLC spectrum of racemic **3d-5**

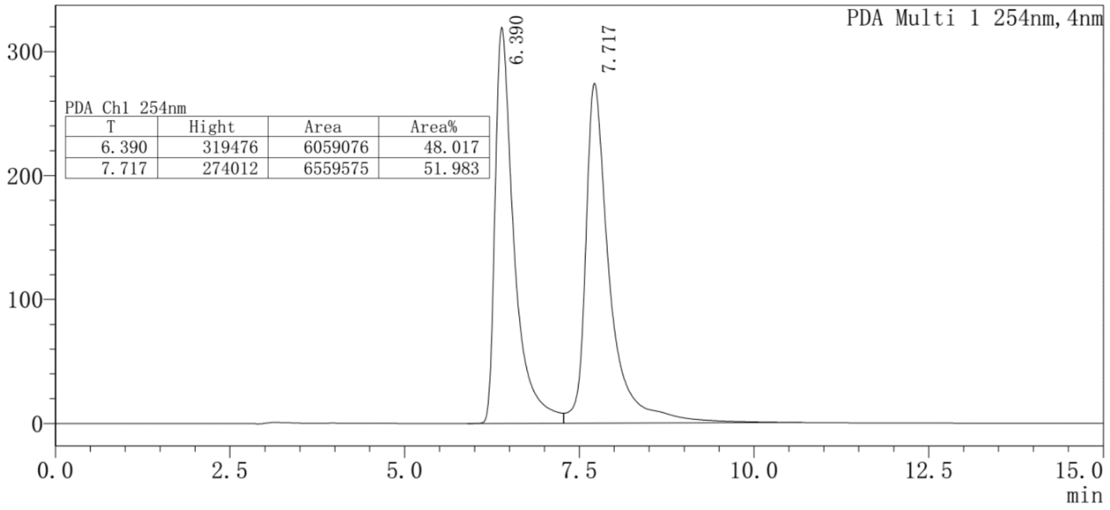

T	Hight	Area	Area%
6.390	319476	6059076	48.017
7.717	274012	6559575	51.983

Supplementary Figure 76. Chiral HPLC spectrum of **(*S*)-3d-5**

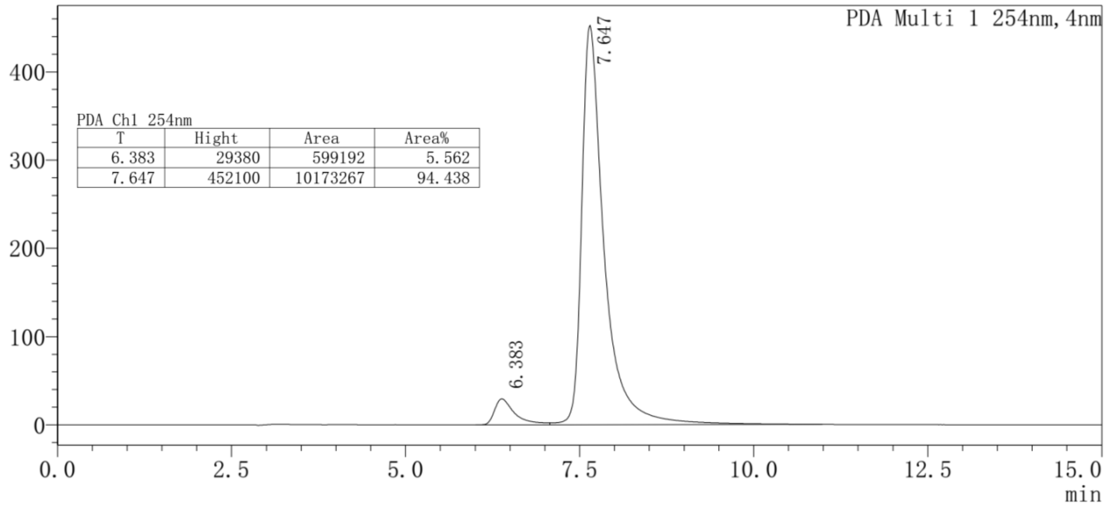

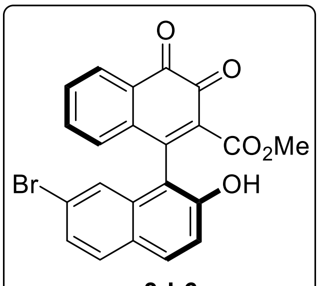

3d-6

(*S*)-Methyl 7'-bromo-2'-hydroxy-3,4-dioxo-3,4-dihydro-[1,1'-binaphthalene]-2-carboxylate ((*S*)-3d-6)

According to the general procedure, **(*S*)-3d-6** was obtained in **92% yield** with **89% ee**.

^{1}H NMR (400 MHz, DMSO-d_6) δ 10.13 (s, 1H), 8.12 (dd, *J* = 7.6, 1.5 Hz, 1H), 8.04 (d, *J* = 1.9 Hz, 1H), 7.99-7.90 (m, 1H), 7.83 (d, *J* = 8.8 Hz, 1H), 7.62-7.58 (m, 1H), 7.55-7.50 (m, 1H), 7.43 (dd, *J* = 8.7, 2.0 Hz, 1H), 7.33 (d, *J* = 9.0 Hz, 1H), 6.72 (dd, *J* = 7.8, 1.3 Hz, 1H), 3.28 (s, 3H);

^{13}C NMR (100 MHz, DMSO-d_6) δ 177.58, 176.81, 164.82, 153.32, 148.87, 135.54, 135.21, 134.66, 133.99, 132.70, 131.65, 131.41, 130.54, 129.30, 129.27, 126.66, 126.47, 121.23, 119.14, 113.01, 52.10.

HRMS (ESI) calcd for [M-H] $C_{22}H_{12}O_5{}^{79}Br$, m/z: 434.9874, found: 434.9870; $C_{22}H_{12}O_5{}^{81}Br$, m/z: 436.9853, found: 436.9848.

HPLC analysis: CHIRALCEL IB, hexane/isopropanol = 60/40, 1.0 mL/min, λ = 254 nm, t_R (minor) = 6.6 min, t_R (major) = 12.3 min, ee = 89%.

Supplementary Figure 77. Chiral HPLC spectrum of racemic **3d-6**

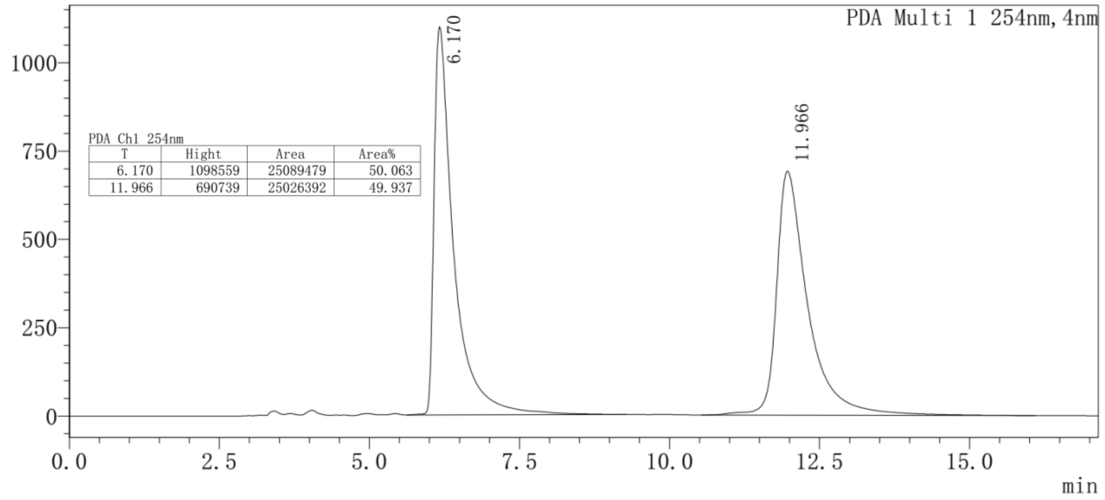

Supplementary Figure 78. Chiral HPLC spectrum of **(*S*)-3d-6**

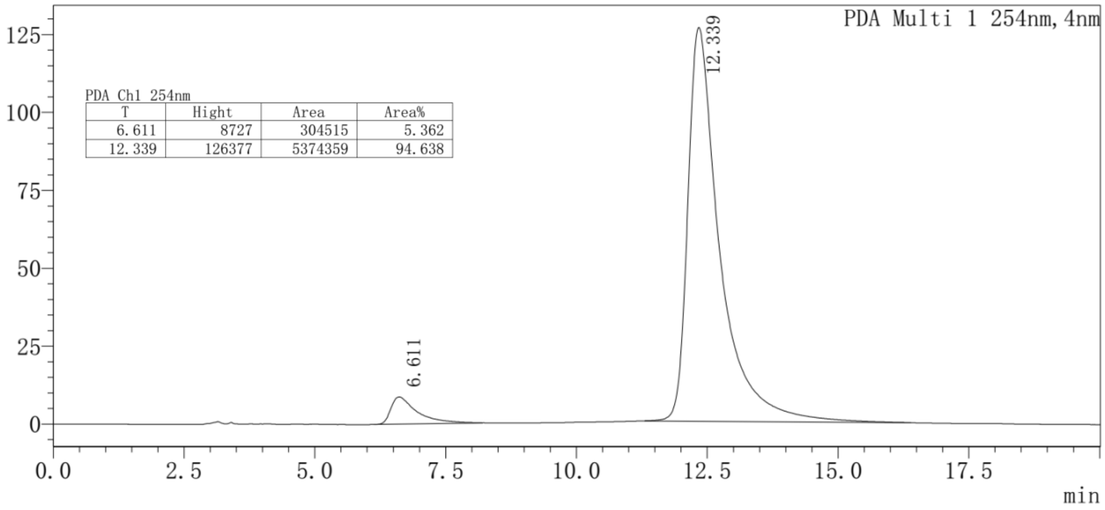

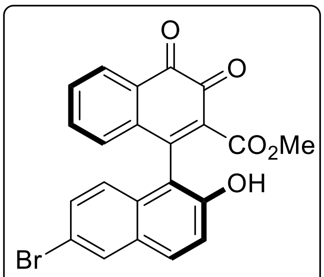

3d-7

(*S*)-Methyl 6'-bromo-2'-hydroxy-3,4-dioxo-3,4-dihydro-[1,1'-binaphthalene]-2-carboxylate ((*S*)-3d-7)

According to the general procedure, **(*S*)-3d-7** was obtained in **91% yield** with **90% ee**.

^{1}H NMR (400 MHz, DMSO-d_6) δ 9.97 (s, 1H), 8.07-7.89 (m, 2H), 7.81 (d, J = 9.0 Hz, 1H), 7.59 (d, J = 9.0 Hz, 1H), 7.47 (td, J = 7.5, 1.2 Hz, 1H), 7.43-7.32 (m, 2H), 7.21 (d, J = 9.0 Hz, 1H), 6.57 (dd, J = 7.8, 1.2 Hz, 1H), 3.14 (s, 3H).

^{13}C NMR (100 MHz, DMSO-d_6) δ 177.62, 176.74, 164.75, 152.81, 149.25, 135.66, 135.09, 134.68, 132.49, 131.71, 131.30, 130.55, 130.10, 129.88, 129.40, 129.38, 129.15, 127.44, 119.85, 116.56, 113.76, 52.15.

HRMS (ESI) calcd for [M-H] $C_{22}H_{12}O_5{}^{79}Br$, m/z: 434.9874, found: 434.9871; $C_{22}H_{12}O_5{}^{81}Br$, m/z: 436.9853, found: 436.9850.

HPLC analysis: CHIRALCEL IB, hexane/isopropanol = 60/40, 1.0 mL/min, λ = 254 nm, t_R (minor) = 6.3 min, t_R (major) = 10.7 min, ee = 90%.

Supplementary Figure 79. Chiral HPLC spectrum of racemic **3d-7**

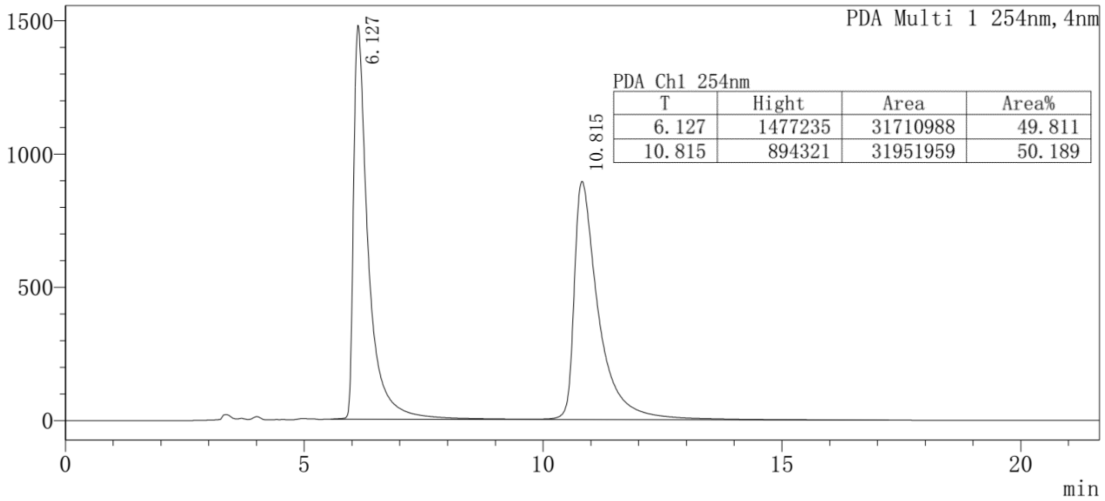

Supplementary Figure 80. Chiral HPLC spectrum of **(*S*)-3d-7**

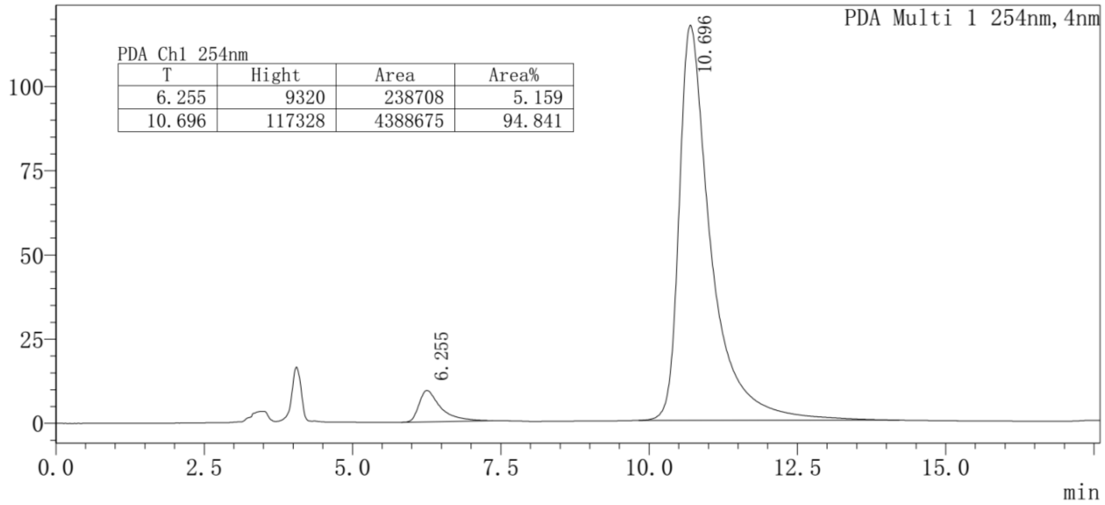

General Procedure for Asymmetric Syntheses of Arylquinones (*S*)-3e

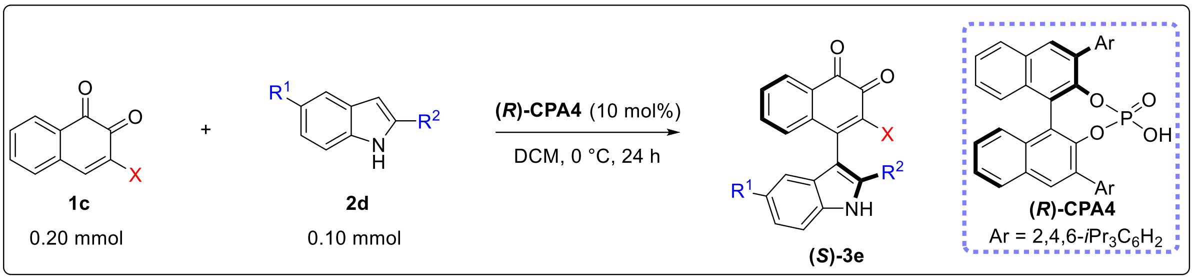

To a mixture of (*R*)-**CPA4** (10 mol%) and **2d** (0.10 mmol) in 10 mL Schlenk tube was added CH_2Cl_2 (1.0 mL). Then the reaction mixture was stirred at 0 ℃ for 20 minutes. After that a solution of **1c** (0.20 mmol) in CH_2Cl_2 (1.0 mL) was added dropwise at 0 ℃. Then the reaction mixture was stirred for 24 h at 0 ℃. The resulting mixture was concentrated under reduced pressure and purified by flash chromatography on silica gel (eluent: PE/DCM = 1/2) to afford the corresponding product **(*S*)-3e.**

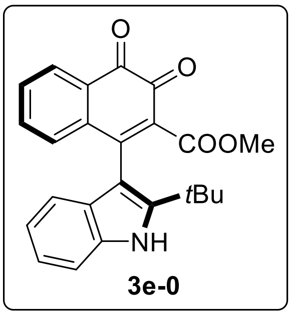

Compound **3e-0** was obtained in **80% yield** with **76% ee** (For detailed reaction conditions see Supplementary Table **5**).

^{1}H NMR (400 MHz, CDCl$_3$) δ 8.39 (s, 1H), 8.22-8.18 (m, 1H), 7.55-7.48 (m, 2H), 7.37 (d, *J* = 8.0 Hz, 1H), 7.28 (d, *J* = 12.0 Hz, 1H), 7.18 (t, *J* = 8.0 Hz, 1H), 7.12-7.10 (m, 1H), 7.04 (t, *J* = 8.0 Hz, 1H), 3.42(s, 3H), 1.39 (s, 9H).

^{13}C NMR (100 MHz, CDCl$_3$) δ 178.37, 177.14, 164.66, 152.17, 143.93, 136.50, 135.99, 135.19, 135.00, 131.53, 130.96, 130.83, 130.31, 127.86, 122.61, 120.47, 118.97, 110.48, 103.98, 52.24, 33.91, 30.47.

HRMS (ESI) calcd for [M+H] $C_{24}H_{22}O_4N$, m/z: 388.1543, found: 388.1543.

HPLC analysis: CHIRALCEL IA, hexane/isopropanol = 85/15, 1.0 mL/min, λ = 214 nm, t_R (major) = 13.9 min, t_R (minor) =12.1 min, ee = 76%.

Supplementary Figure 81. Chiral HPLC spectrum of racemic **3e-0**

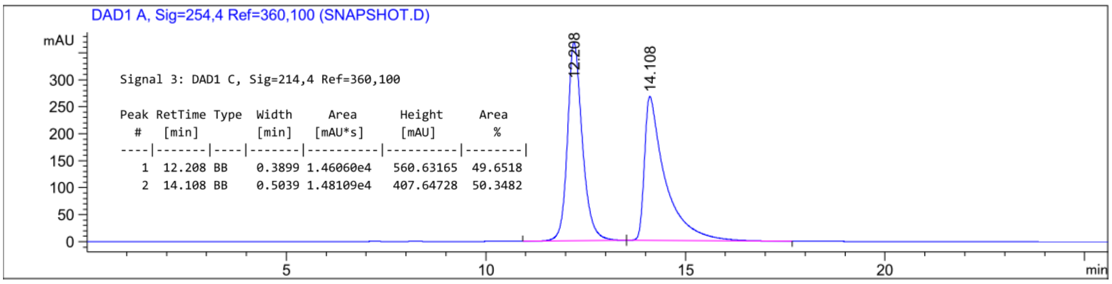

Supplementary Figure 82. Chiral HPLC spectrum of best results of compound **3e-0**

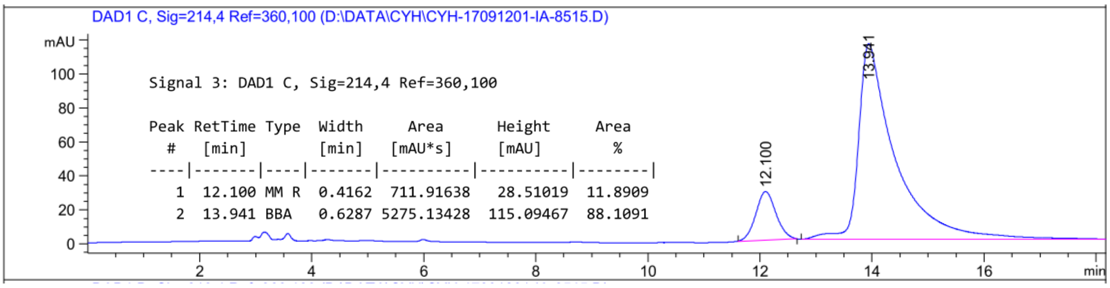

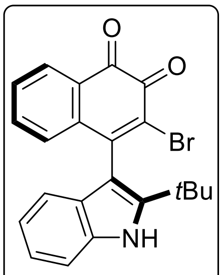

3e-1

According to the general procedure, **(*S*)-3e-1** was obtained in **95% yield** with **96% ee**.

^{1}H NMR (400 MHz, $CDCl_3$) δ 8.38 (s, 1H), 8.19-8.18 (m, 1H), 7.50-7.42 (m, 3H), 7.20-7.08 (m, 4H), 1.40 (s, 9H).

^{13}C NMR (100 MHz, $CDCl_3$) δ 177.89, 174.99, 154.33, 142.79, 137.14, 136.30, 135.15, 130.97, 130.79, 130.56, 130.05, 127.80, 126.78, 122.73, 120.59, 118.51, 110.82, 107.89, 33.71, 30.28.

HRMS (ESI) calcd for [M+H] $C_{22}H_{19}{}^{79}BrO_2N$, m/z: 408.0599, found: 408.0596; $C_{22}H_{19}{}^{81}BrO_2N$, m/z: 410.0579, found: 410.0580.

HPLC analysis: CHIRALCEL IA, hexane/isopropanol = 80/20, 1.0 mL/min, λ = 254 nm, t_R (major) = 6.7 min, t_R (minor) = 9.5 min, ee = 95%.

Supplementary Figure 83. Chiral HPLC spectrum of racemic **3e-1**

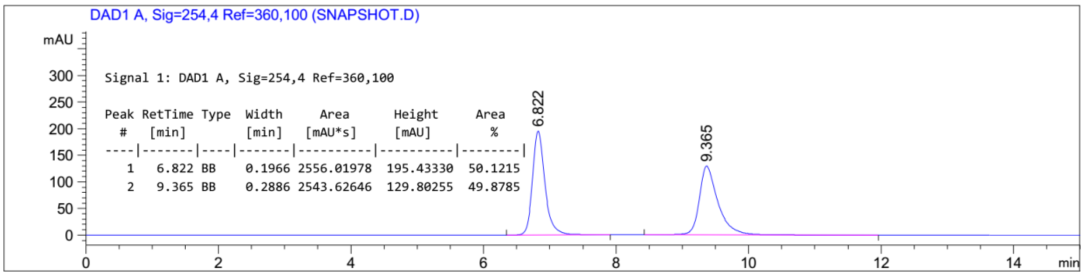

Supplementary Figure 84. Chiral HPLC spectrum of **(*S*)-3e-1**

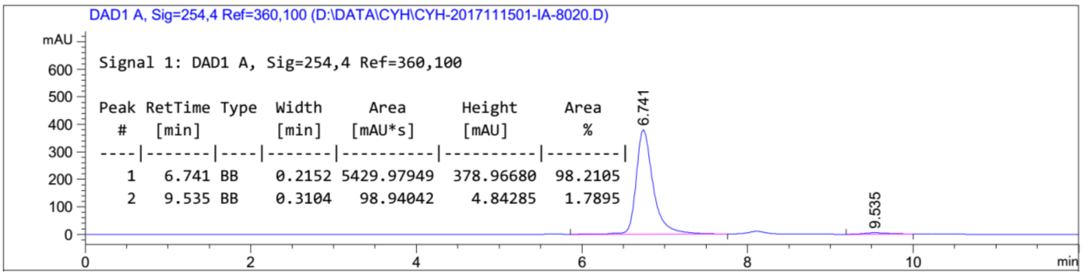

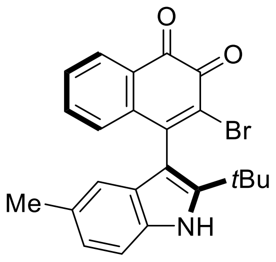

3e-2

According to the general procedure, **(*S*)-3e-2** was obtained in **95% yield** with **98% ee**.

^{1}H NMR (400 MHz, CDCl$_3$) δ 8.26 (s, 1H), 8.21-8.17 (m, 1H), 7.55-7.45 (m, 2H), 7.32 (d, *J* = 12.0 Hz, 1H), 7.10-7.08 (m, 1H), 7.05-7.03 (m, 1H), 6.93 (s, 1H), 2.35 (s, 3H), 1.38 (s, 9H).

^{13}C NMR (100 MHz, CDCl$_3$) δ 177.94, 175.04, 154.56, 142.78, 137.17, 136.30, 133.40, 130.93, 130.84, 130.53, 130.05, 129.99, 127.76, 127.01, 124.29, 118.09, 110.46, 107.48, 33.69, 30.28, 21.56.

HRMS (ESI) calcd for [M+H] $C_{23}H_{21}{}^{79}BrO_2N$, m/z: 422.0756, found: 422.0754; $C_{23}H_{21}{}^{81}BrO_2N$, m/z: 424.0735, found: 424.0736.

HPLC analysis: CHIRALCEL IA, hexane/isopropanol = 80/20, 1.0 mL/min, λ = 254 nm, t_R (major) = 6.2 min, t_R (minor) = 7.7 min, ee = 98%.

Supplementary Figure 85. Chiral HPLC spectrum of racemic **3e-2**

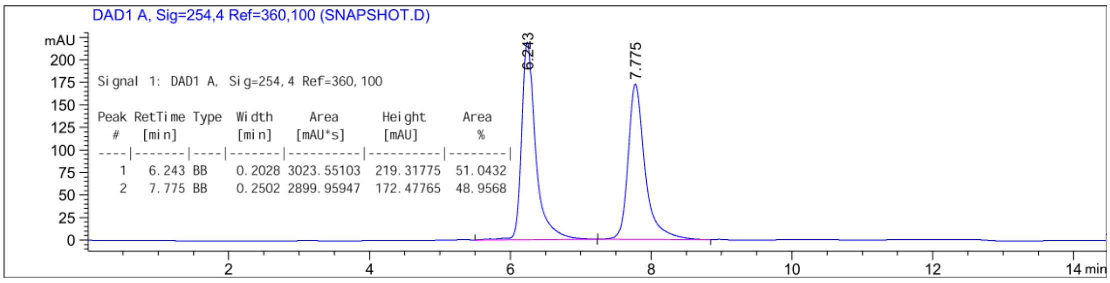

Supplementary Figure 86. Chiral HPLC spectrum of **(*S*)-3e-2**

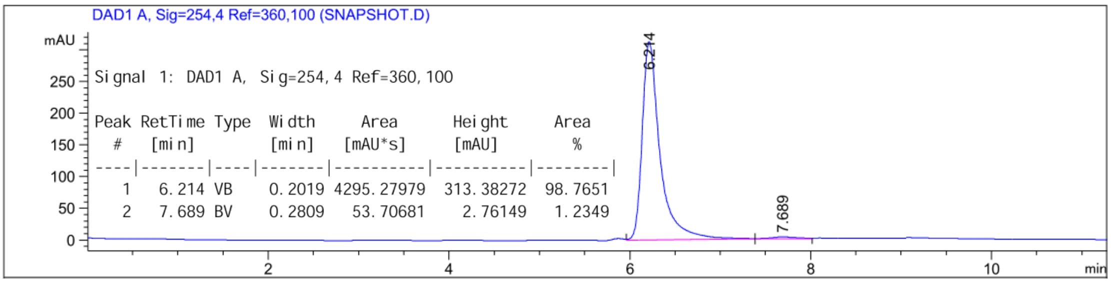

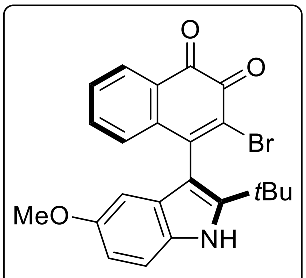

3e-3

According to the general procedure, **(*S*)-3e-3** was obtained in **93% yield** with **93% ee**.

^{1}H NMR (400 MHz, $CDCl_3$) δ 8.31 (s, 1H), 8.19-8.17 (m, 1H), 7.53-7.46 (m, 2H), 7.32 (d, *J* = 8.0 Hz, 1H), 7.11 (d, *J* = 8.0 Hz, 1H), 6.88-6.83 (m, 1H), 6.57-6.56 (m, 1H), 3.73 (s, 3H), 1.37 (s, 9H).

^{13}C NMR (100 MHz, $CDCl_3$) δ 177.93, 175.08, 154.81, 154.41, 143.46, 137.09, 136.32, 130.99, 130.83, 130.56, 130.09, 130.07, 127.79, 127.25, 112.62, 111.63, 107.78, 100.29, 56.03, 33.75, 30.30.

HRMS (ESI) calcd for [M+H] $C_{23}H_{21}{}^{79}BrO_3N$, m/z: 438.0705, found: 438.0703; $C_{23}H_{21}{}^{81}BrO_3N$, m/z: 440.0684, found: 440.0687.

HPLC analysis: CHIRALCEL IA, hexane/isopropanol = 80/20, 1.0 mL/min, λ = 254 nm, t_R (major) = 8.0 min, t_R (minor) = 9.7 min, ee = 93%.

Supplementary Figure 87. Chiral HPLC spectrum of racemic **3e-3**

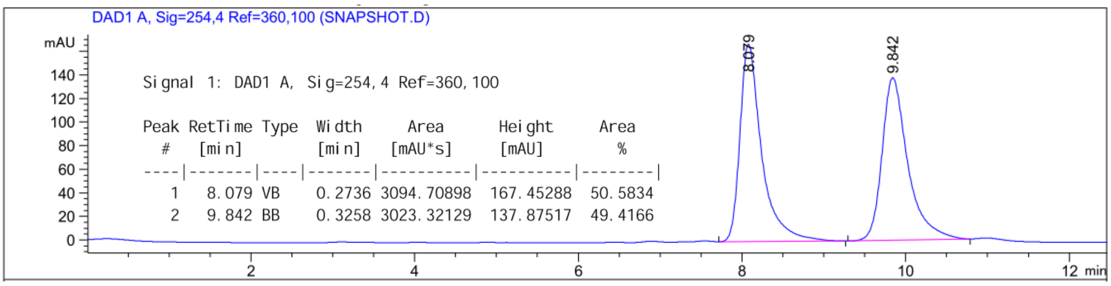

Supplementary Figure 88. Chiral HPLC spectrum of **(*S*)-3e-3**

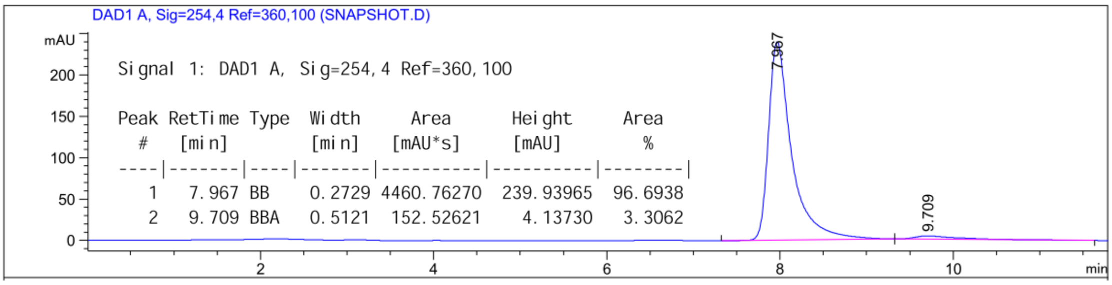

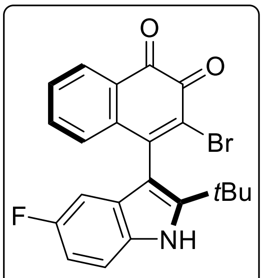

3e-4

According to the general procedure, **(*S*)-3e-4** was obtained in **90% yield** with **98% ee**.

^{1}H NMR (400 MHz, $CDCl_3$) δ 8.38 (s, 1H), 8.20-8.17 (m, 1H), 7.54-7.47 (m, 2H), 7.35-7.32 (m, 1H), 7.06 (d, *J* = 8.0 Hz, 1H), 6.98-6.93 (m, 1H), 6.82-6.79 (m, 1H), 1.38 (s, 9H).

^{13}C NMR (100 MHz, $CDCl_3$) δ 177.74, 174.91, 153.57, 144.72, 136.91, 136.32, 131.56, 131.11, 130.71, 130.57, 130.10, 127.94, 127.27, 127.17, 111.65, 111.55, 111.17, 110.91, 103.73, 103.49, 33.82, 30.23.

HRMS (ESI) calcd for [M+H] $C_{22}H_{18}F^{79}BrO_2N$, m/z: 426.0505, found: 426.0508; $C_{22}H_{18}F^{81}BrO_2N$, m/z: 428.0484, found: 428.0483.

HPLC analysis: CHIRALCEL IA, hexane/isopropanol = 80/20, 1.0 mL/min, λ = 254 nm, t_R (major) = 6.6 min, t_R (minor) = 8.8 min, ee = 98%.

Supplementary Figure 89. Chiral HPLC spectrum of racemic **3e-4**

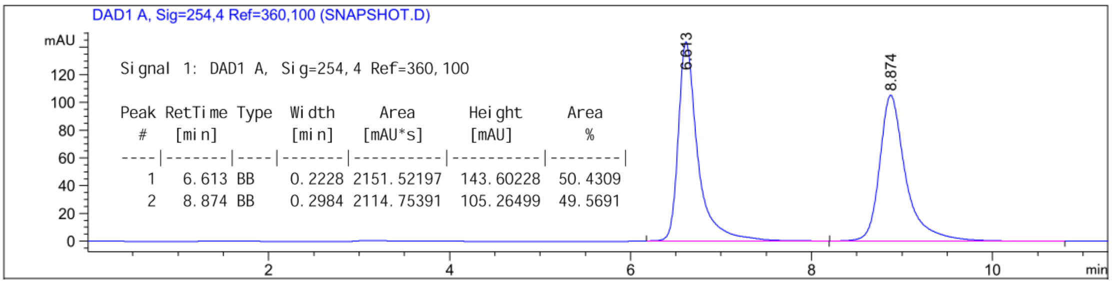

Supplementary Figure 90. Chiral HPLC spectrum of **(*S*)-3e-4**

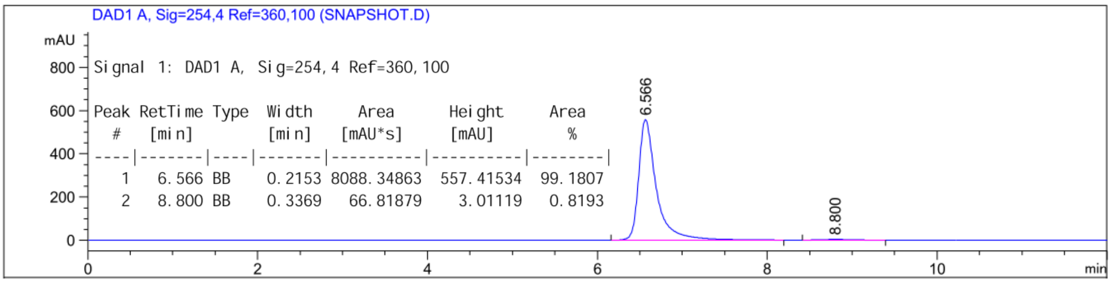

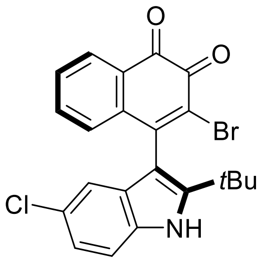

3e-5

According to the general procedure, **(*S*)-3e-5** was obtained in **89% yield** with **98% ee**.

^{1}H NMR (400 MHz, CDCl$_3$) δ 8.43 (s, 1H), 8.20-8.18 (m, 1H), 7.54-7.47 (m, 2H), 7.35 (d, *J* = 8.0 Hz, 1H), 7.17 (d, *J* = 8.0 Hz, 1H), 7.12-7.10 (m, 1H), 7.04 (d, *J* = 8.0 Hz, 1H), 1.38 (s, 9H).

^{13}C NMR (100 MHz, CDCl$_3$) δ 177.63, 174.54, 153.35, 144.38, 136.87, 136.36, 133.50, 131.14, 130.75, 130.54, 130.09, 128.00, 127.83, 126.32, 123.07, 117.89, 111.94, 107.60, 33.80, 30.19.

HRMS (ESI) calcd for [M+H] $C_{22}H_{18}{}^{35}Cl^{79}BrO_2N$, m/z: 442.0209, found: 442.0210; $C_{22}H_{18}{}^{35}Cl^{81}BrO_2N$, m/z: 444.0189, found: 444.0188; $C_{22}H_{18}{}^{37}Cl^{79}BrO_2N$, m/z: 444.0180, found: 444.0183; $C_{22}H_{18}{}^{37}Cl^{81}BrO_2N$, m/z: 446.0159, found: 446.0160.

HPLC analysis: CHIRALCEL IA, hexane/isopropanol = 80/20, 1.0 mL/min, λ = 254 nm, t_R (major) = 6.3 min, t_R (minor) = 9.9 min, ee = 98%.

Supplementary Figure 91. Chiral HPLC spectrum of racemic **3e-5**

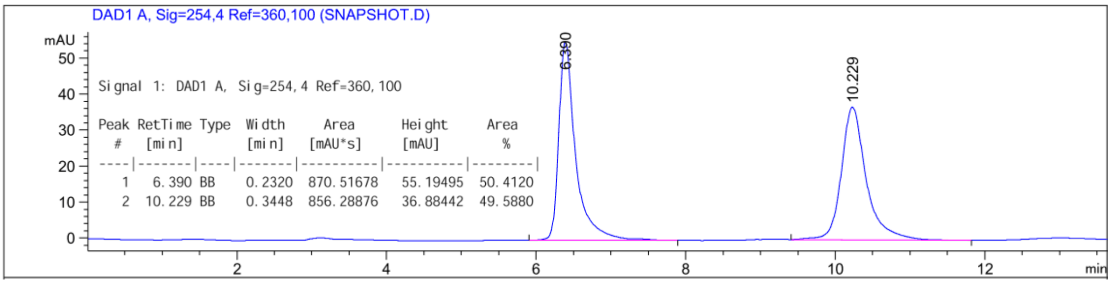

Supplementary Figure 92. Chiral HPLC spectrum of **(*S*)-3e-5**

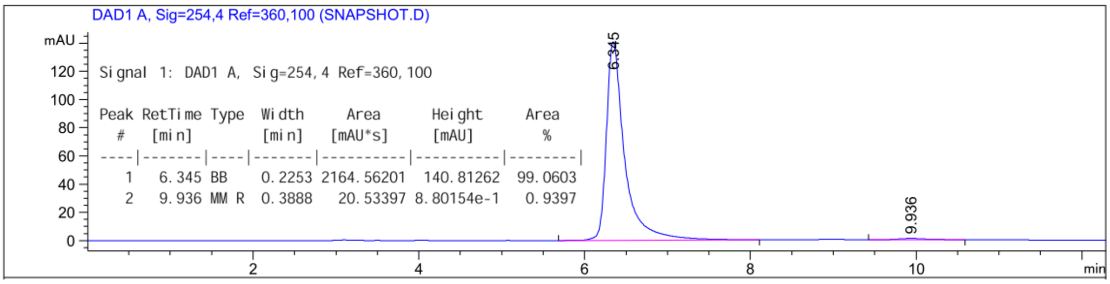

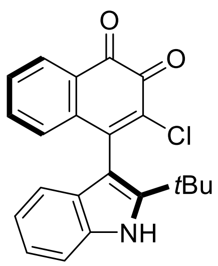

3e-6

According to the general procedure, **(*S*)-3e-6** was obtained in **95% yield** with **97% ee**.

^{1}H NMR (400 MHz, Acetone-d$_6$) δ 10.54 (s, 1H), 8.13-8.11 (m, 1H), 7.62-7.56 (m, 2H), 7.42 (d, *J* = 8.0 Hz, 1H), 7.29 (d, *J* = 8.0 Hz, 1H), 7.13-7.07 (m, 2H), 6.93 (t, *J* = 8.0 Hz, 1H), 1.43 (s, 9H).

^{13}C NMR (100 MHz, Acetone-d$_6$) δ 178.66, 175.04, 150.67, 144.58, 137.86, 136.85, 136.35, 131.65, 131.24, 130.92, 130.10, 128.11, 122.62, 120.37, 119.22, 111.73, 111.68, 105.72, 34.33, 30.42.

HRMS (ESI) calcd for [M+H] $C_{22}H_{19}{}^{35}ClO_2N$, m/z: 364.1104, found: 364.1108; $C_{22}H_{19}{}^{37}ClO_2N$, m/z: 366.1075, found: 366.1078.

HPLC analysis: CHIRALCEL IA, hexane/isopropanol = 80/20, 1.0 mL/min, λ = 254 nm, t_R (major) = 7.0 min, t_R (minor) = 9.7 min, ee = 97%.

Supplementary Figure 93. Chiral HPLC spectrum of racemic **3e-6**

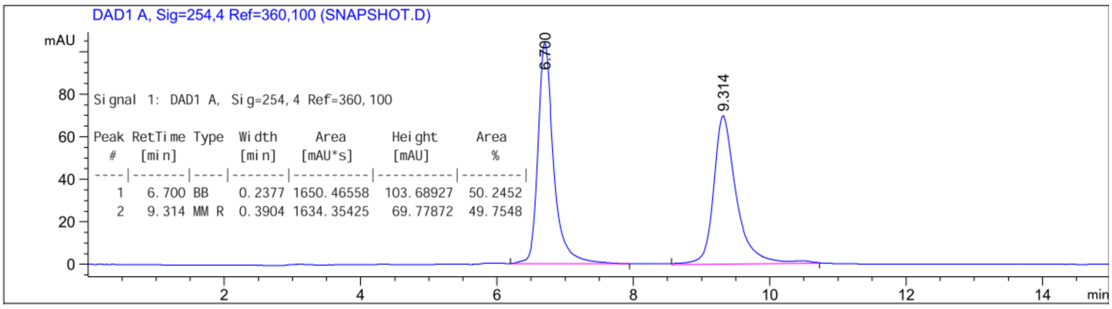

Supplementary Figure 94. Chiral HPLC spectrum of **(*S*)-3e-6**

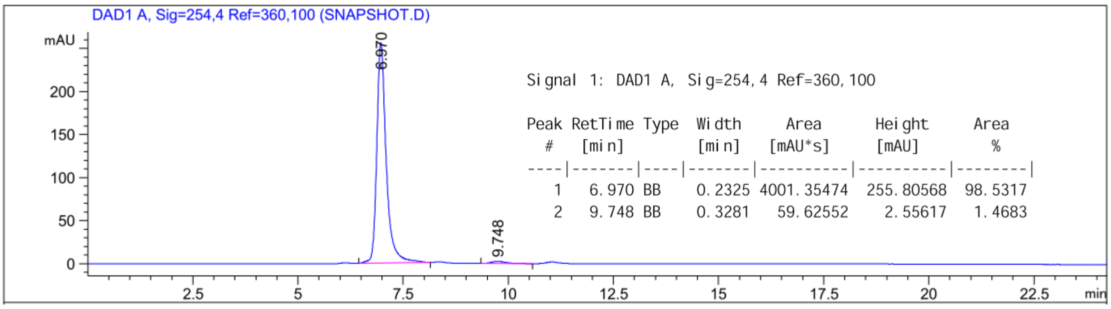

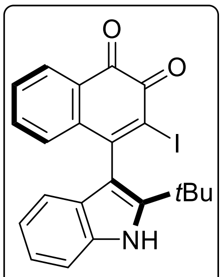

3e-7

According to the general procedure, **(*S*)-3e-7** was obtained in **90% yield** with **93% ee**.

^{1}H NMR (400 MHz, CDCl$_3$) δ 8.35 (s, 1H), 8.19-8.17 (m, 1H), 7.49-7.47 (m, 2H), 7.43 (d, *J* = 8.0 Hz, 1H), 7.25-7.20 (m, 1H), 7.17 (d, *J* = 8.0 Hz, 1H), 7.08-7.04 (m, 2H), 1.39 (s, 9H).

^{13}C NMR (100 MHz, CDCl$_3$) δ 178.23, 174.87, 150.88, 143.38, 136.69, 136.26, 135.13, 133.74, 130.83, 130.57, 130.46, 130.00, 127.11, 122.73, 120.63, 118.48, 110.81, 105.29, 33.68, 30.26.

HRMS (ESI) calcd for [M+H] $C_{22}H_{19}IO_2N$, m/z: 456.0455, found: 456.0438.

HPLC analysis: CHIRALCEL IA, hexane/isopropanol = 80/20, 1.0 mL/min, λ = 254 nm, t_R (major) = 7.0 min, t_R (minor) = 9.7 min, ee = 93%.

Supplementary Figure 95. Chiral HPLC spectrum of racemic **3e-7**

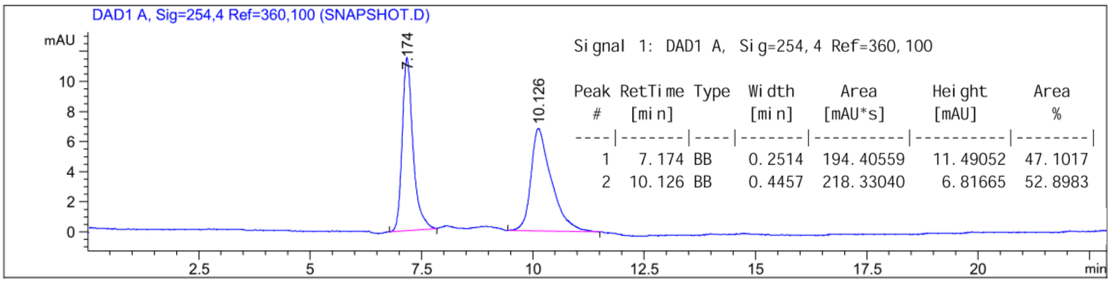

Supplementary Figure 96. Chiral HPLC spectrum of **(*S*)-3e-7**

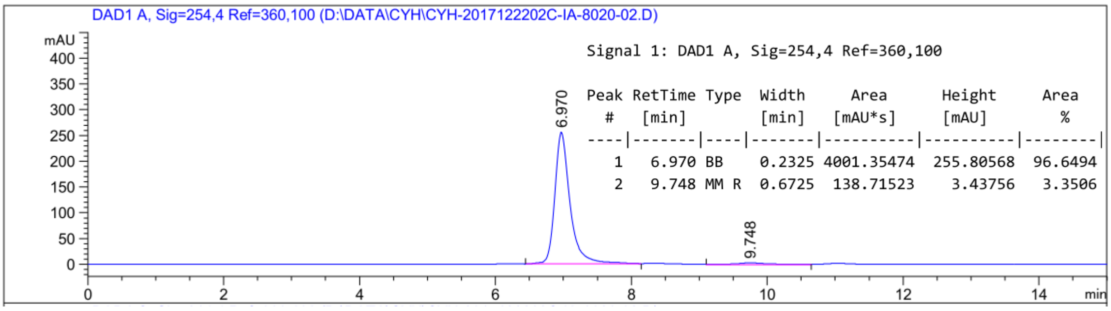

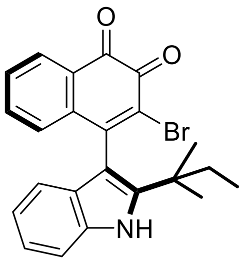

3e-8

According to the general procedure, **(*S*)-3e-8** was obtained in **92% yield** with **93% ee**.

^{1}H NMR (400 MHz, $CDCl_3$) δ 8.31 (s, 1H), 8.19-8.17 (m, 1H), 7.52-7.42 (m, 3H), 7.23 (t, *J* = 8.0 Hz, 1H), 7.18 (d, *J* = 8.0 Hz, 1H), 7.10-7.05 (m, 2H), 1.81-1.67 (m, 2H), 1.34 (s, 3H), 1.32 (s, 3H), 0.82 (t, *J* = 8.0 Hz, 3H).

^{13}C NMR (100 MHz, $CDCl_3$) δ 177.90, 175.01, 154.26, 141.87, 137.19, 136.18, 135.20, 130.95, 130.84, 130.55, 130.02, 127.82, 126.82, 122.72, 120.54, 118.50, 110.79, 109.17, 37.39, 36.82, 27.52, 27.22, 9.64.

HRMS (ESI) calcd for [M-H] $C_{23}H_{19}{}^{79}BrO_2N$, m/z: 420.0599, found: 420.0601; $C_{23}H_{19}{}^{81}BrO_2N$, m/z: 422.0579, found: 422.0581.

HPLC analysis: CHIRALCEL IA, hexane/isopropanol = 80/20, 1.0 mL/min, λ = 254 nm, t_R (major) = 6.5 min, t_R (minor) = 8.6 min, ee = 93%.

Supplementary Figure 97. Chiral HPLC spectrum of racemic **3e-8**

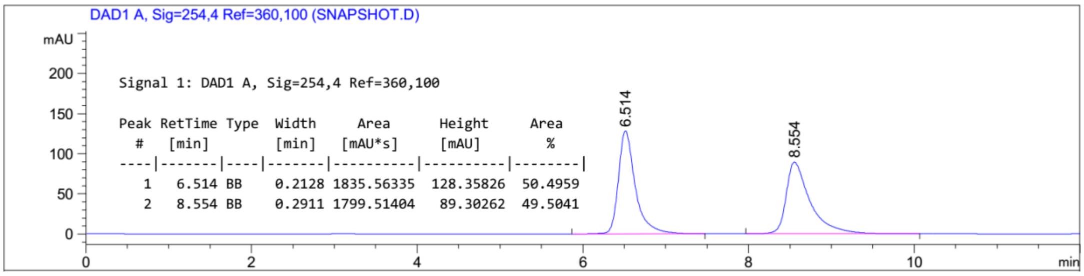

Supplementary Figure 98. Chiral HPLC spectrum of **(*S*)-3e-8**

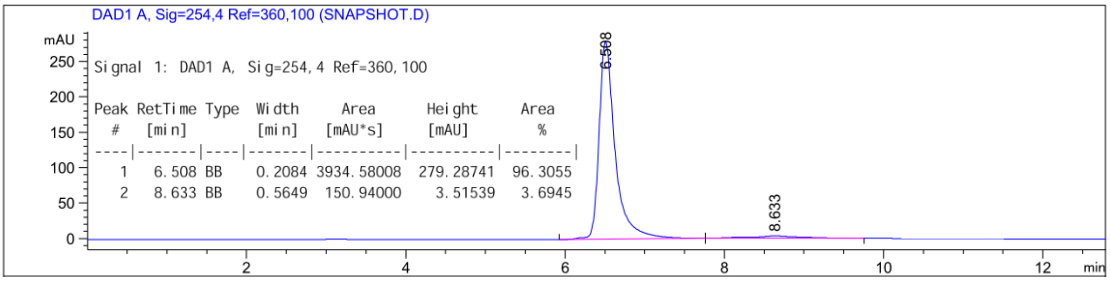

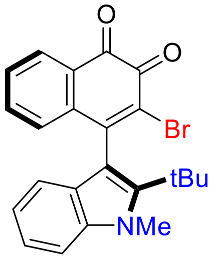

(*S*)-3e-9

According to the general procedure, **(*S*)-3e-9** was obtained in **78% yield** with **0% ee**.

^{1}H NMR (400 MHz, $CDCl_3$) δ 8.21-8.19 (m, 1H), 7.54-7.46 (m, 2H), 7.40 (d, *J* = 8.0 Hz, 1H), 7.31-7.27 (m, 1H), 7.16 (d, *J* = 8.0 Hz, 1H), 7.10-7.05 (m, 2H), 4.05 (s, 3H), 1.51 (s, 9H).

^{13}C NMR (100 MHz, $CDCl_3$) δ 177.89, 174.91, 155.68, 143.31, 138.38, 137.36, 136.30, 130.86, 130.59, 130.48, 130.05, 127.69, 125.54, 122.57, 120.45, 118.49, 109.16, 108.76, 34.26, 33.42, 30.24.

HRMS (ESI) calcd for [M+H] $C_{23}H_{21}{}^{79}BrNO_2$, m/z: 422.0756, found: 422.0748; $C_{23}H_{21}{}^{81}BrNO_2$, m/z: 424.0735, found: 424.0738.

HPLC analysis: CHIRALCEL ID, hexane/isopropanol = 80/20, 1.0 mL/min, λ = 254 nm, t_R (major) = 18.3 min, t_R (minor) = 20.2 min, ee = 0%.

Supplementary Figure 99. Chiral HPLC spectrum of racemic **3e-9**

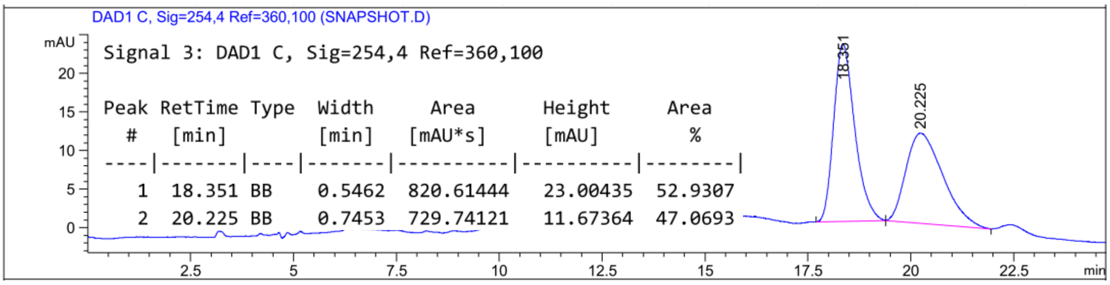

Supplementary Figure 100. Chiral HPLC spectrum of **(*S*)-3e-9**

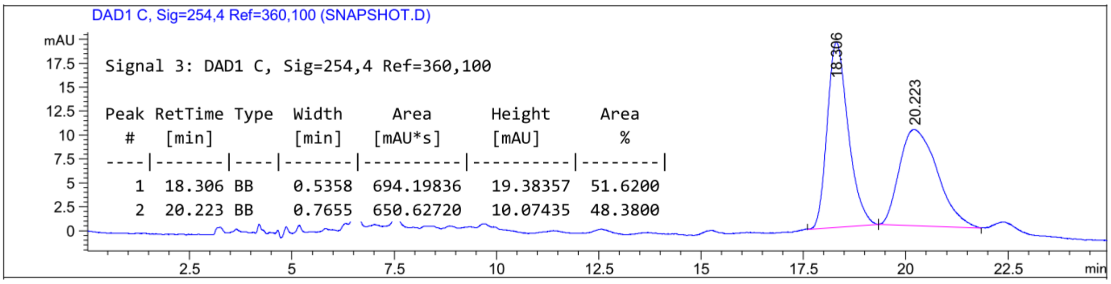

Supplementary Note 5

Studies of the Configurational Stability

Experimental measurement of enantiomerization barrier for 3c-1

The enantiomerization barrier, corresponding to barrier to rotation for **3c-1** atropisomers, was obtained by kinetic of racemization of an enantiomer via chiral HPLC analysis. The slope of the first-order kinetic line gives the racemization constant (K racemization = 2 x K enantiomerization). Eyring equation gives the enantiomerization barrier from enantiomerization constant (k enantiomerization), R = 8.31451 $J*K^{-1}*mol^{-1}$, h = $6.62608*10^{-34}$ J/s and k_B = $1.38066*10^{-23}$ J/K.

Solvent: CH_3CN, Temperature: 80 ℃

Time (min)	% first eluted enantiomer (%t)	ln((%t - 50)/(%t_0 - 50))
0	99.81	0
120	98.56	-0.02541562
300	97.58	-0.04580326
480	97.09	-0.05615510
1380	92.82	-0.15121048
2160	89.76	-0.22535439
2880	86.70	-0.30516657
3600	82.94	-0.41352804
4320	81.25	-0.46619639

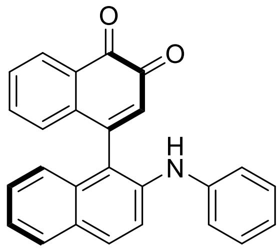

3c-1

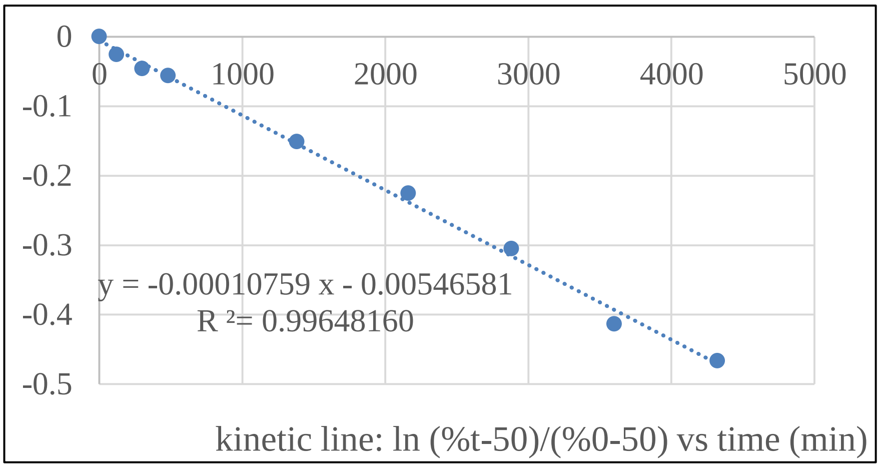

K racemization = 1.79949×10^{-6} S^{-1}
K enantiomerization = 8.9974×10^{-7} S^{-1}
ΔG enantiomerization = 127.9 KJ/mol
Half-life time $t_{1/2}$ = 385193 seconds = 6420 minutes = 107 hours = 4.46 days
T = 25 ℃, $t_{1/2}$ = 60.13 years

Supplementary Note 6

Synthetic Transformations

General Procedure for Synthetic Transformation from 3b to 4b

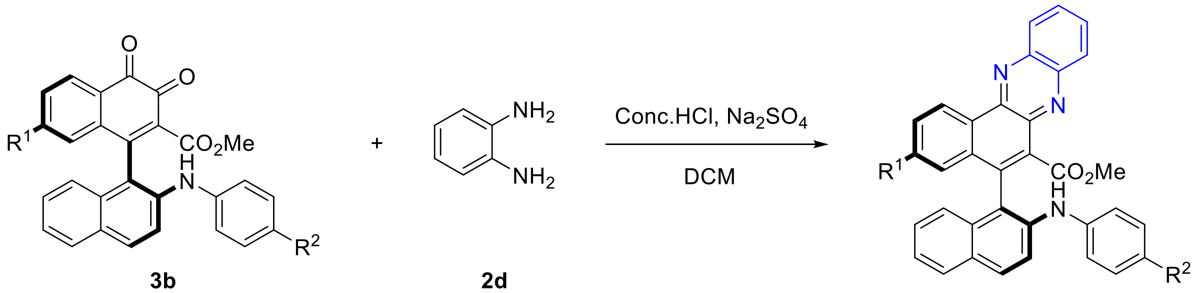

3b (0.30 mmol), **2d** (0.90 mmol) and Na_2SO_4 (50 mg) was dissolved in 10 mL CH_2Cl_2, then Conc. HCl (20 μL) was added. The reaction was stirred for 5 min under air atmosphere. After completion, the mixture was extracted with CH_2Cl_2, dried over Na_2SO_4, and the solvent was removed under reduced pressure. The residue was purified by flash column chromatography with CH_2Cl_2 to give yellow solid **4b**.

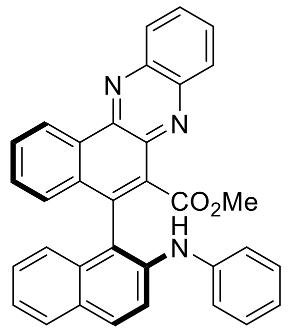

4b-1

(*S*)-Methyl 5-(2-(phenylamino)naphthalen-1-yl)benzo[*a*]phenazine-6-carboxylate ((*S*)-4b-1)

According to the general procedure, **(*S*)-4b-1** was obtained in **99% yield** with **94% ee**.

^{1}H NMR (500 MHz, CDCl$_3$) δ 9.53 (dd, *J* = 8.1, 1.2 Hz, 1H), 8.42 (dd, *J* = 8.4, 1.6 Hz, 1H), 8.32 (dd, *J* = 8.3, 1.6 Hz, 1H), 7.94-7.85 (m, 4H), 7.80 (t, *J* = 7.5 Hz, 1H), 7.75 (d, *J* = 9.0 Hz, 1H), 7.57 (t, *J* = 7.3 Hz, 1H), 7.44 (d, *J* = 8.1 Hz, 1H), 7.36-7.32 (m, 1H), 7.28-7.18 (m, 4H), 7.04 (d, *J* = 7.9 Hz, 2H), 6.90 (t, *J* = 7.3 Hz, 1H), 6.04 (s, 1H), 3.67 (s, 3H).

^{13}C NMR (125 MHz, CDCl$_3$) δ 168.41, 142.95, 142.89, 142.50, 141.97, 140.66, 139.84, 137.81, 134.31, 133.72, 132.23, 131.79, 130.74, 130.56, 130.42, 130.02, 129.96, 129.74,

129.30, 129.28, 129.22, 128.01, 127.21, 126.99, 125.66, 125.07, 123.87, 121.51, 119.08, 118.96, 118.85, 52.60.

HRMS (ESI) calcd for [M+H] $C_{34}H_{24}O_2N_3$, m/z: 506.1863, found: 506.1859.

HPLC analysis: CHIRALCEL IA, hexane/isopropanol = 60/40, 1.0 mL/min, λ = 254 nm, t_R (major) = 4.8 min, t_R (minor) = 7.3 min, ee = 94%.

M.P.: decomposed over 160 ℃.

Supplementary Figure 101. Chiral HPLC spectrum of racemic **4b-1**

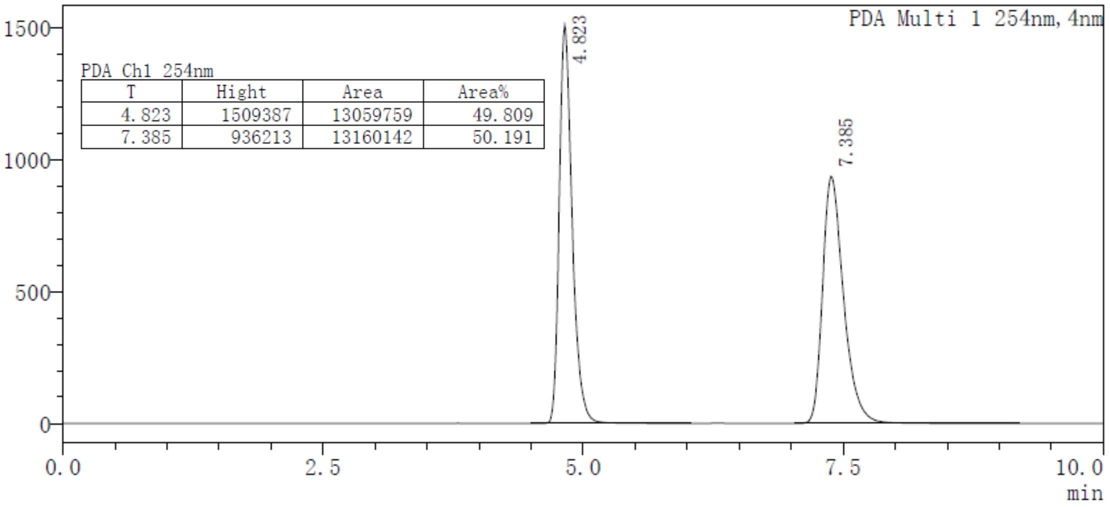

Supplementary Figure 102. Chiral HPLC spectrum of **(*S*)-4b-1**

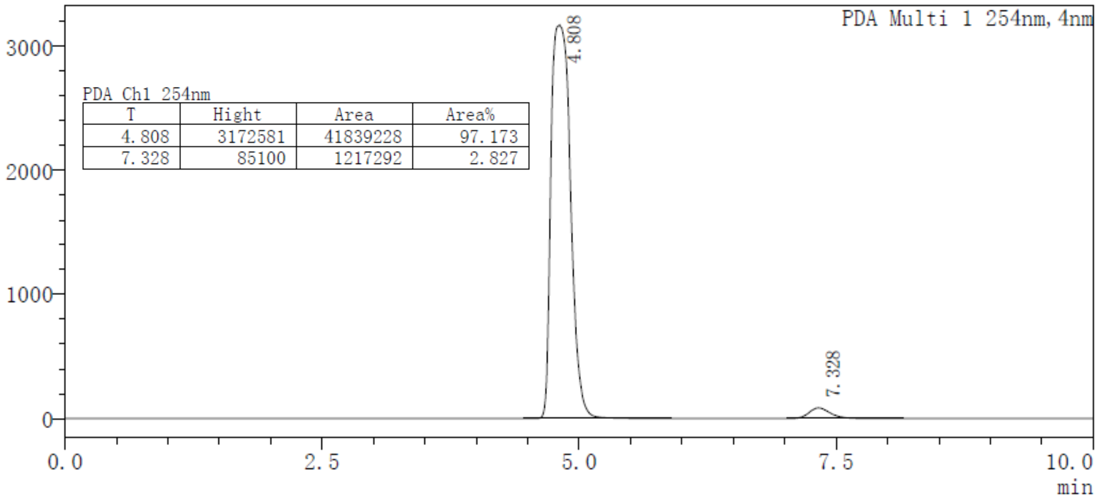

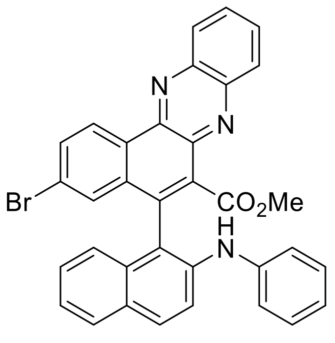

4b-7

(*S*)-Methyl 3-bromo-5-(2-(phenylamino)naphthalen-1-yl)benzo[a]phenazine-6-carboxylate ((*S*)-4b-7)

According to the general procedure, **(*S*)-4b-7** was obtained in **99% yield** with **96% ee**.

M.P.: decomposed over 160 ℃.

^{1}H NMR (500 MHz, $CDCl_3$) δ 9.31 (d, *J* = 8.7 Hz, 1H), 8.40 (dd, *J* = 8.2, 1.3 Hz, 1H), 8.30 (dd, *J* = 8.2, 1.6 Hz, 1H), 7.98-7.88 (m, 4H), 7.84 (dd, *J* = 8.6, 2.0 Hz, 1H), 7.76 (d, *J* = 9.0 Hz, 1H), 7.58 (d, *J* = 1.9 Hz, 1H), 7.40-7.36 (m, 1H), 7.32-7.22 (m, 4H), 7.08 (d, *J* = 7.3 Hz, 2H), 6.96-6.92 (m, 1H), 6.01 (s, 1H), 3.67 (s, 3H).

^{13}C NMR (125 MHz, $CDCl_3$) δ 167.98, 142.98, 142.86, 142.55, 141.42, 140.32, 140.05, 136.70, 135.57, 133.73, 133.49, 132.46, 131.08, 130.69, 130.50, 130.43, 130.00, 129.69, 129.44, 129.40, 128.15, 127.30, 127.15, 125.60, 124.81, 124.04, 121.74, 119.10, 118.95, 118.39, 52.64.

HRMS (ESI) calcd for [M+H] $C_{34}H_{23}O_2N_3{}^{79}Br$, m/z: 584.0968, found: 584.0964; $C_{34}H_{23}O_2N_3{}^{81}Br$, m/z: 586.0948, found: 586.0944.

HPLC analysis: CHIRALCEL IA, hexane/isopropanol = 60/40, 1.0 mL/min, λ = 254 nm, t_R (major) = 4.9 min, t_R (minor) = 6.9 min, ee = 96%.

Supplementary Figure 103. Chiral HPLC spectrum of racemic **4b-7**

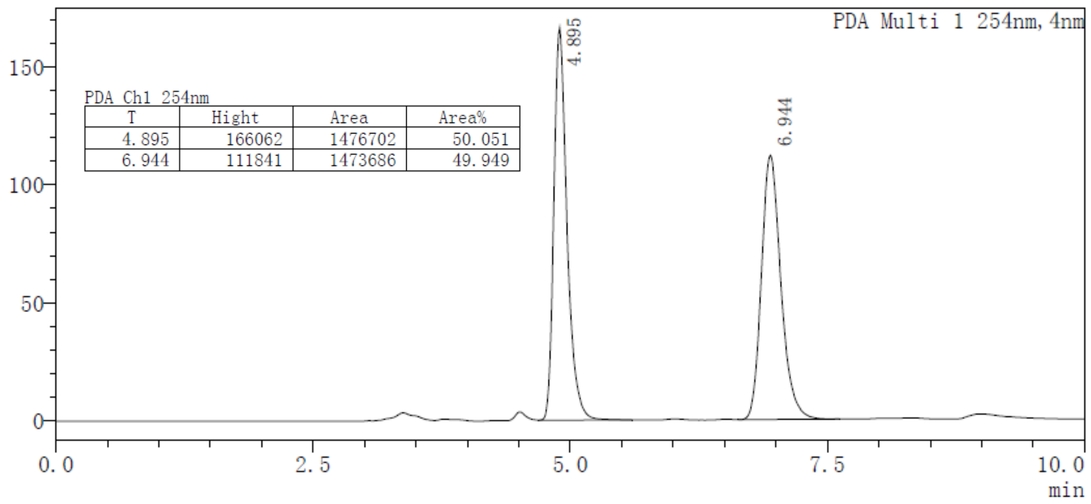

Supplementary Figure 104. Chiral HPLC spectrum of **(*S*)-4b-7**

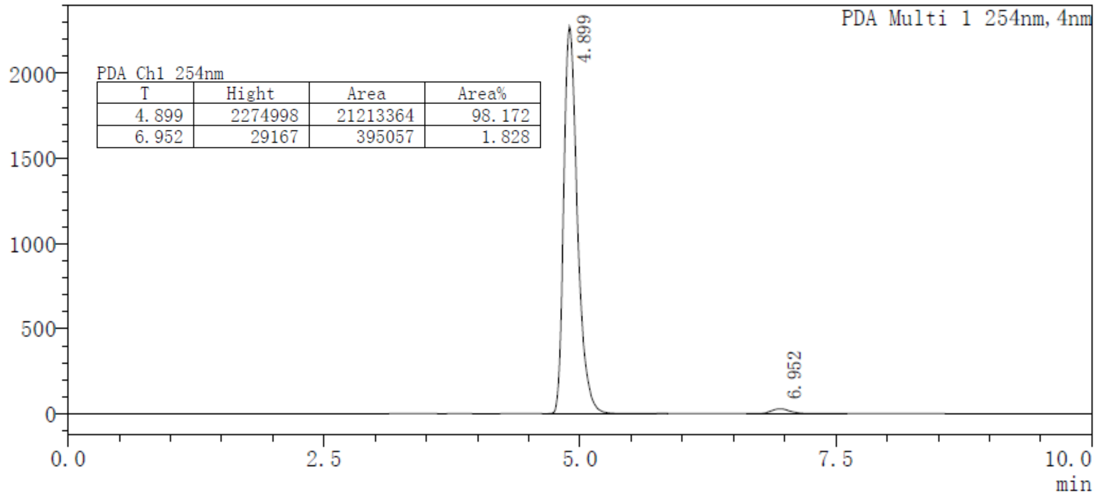

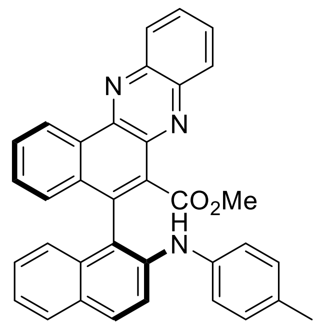

4b-12

(*S*)-Methyl 5-(2-(p-tolylamino)naphthalen-1-yl)benzo[a]phenazine-6-carboxylate ((*S*)-4b-12)

According to the general procedure, **(*S*)-4b-12** was obtained in **99% yield** with **96% ee**.

M.P.: decomposed over 160 ℃.

^{1}H NMR (500 MHz, $CDCl_3$) δ 9.56 (dd, *J* = 8.1, 1.2 Hz, 1H), 8.45 (dd, *J* = 8.3, 1.6 Hz, 1H),

8.35 (dd, J = 8.4, 1.5 Hz, 1H), 7.96-7.86 (m, 4H), 7.83 (ddd, J = 8.1, 7.0, 1.2 Hz, 1H), 7.71 (d, J = 8.9 Hz, 1H), 7.60 (ddd, J = 8.3, 7.1, 1.3 Hz, 1H), 7.49 (dd, J = 8.2, 1.1 Hz, 1H), 7.34 (ddd, J = 8.1, 6.4, 1.6 Hz, 1H), 7.30-7.23 (m, 2H), 7.06 (d, J = 8.2 Hz, 2H), 7.00 (d, J = 8.4 Hz, 2H), 5.94 (s, 1H), 3.70 (s, 3H), 2.29 (s, 3H).

^{13}C NMR (125 MHz, $CDCl_3$) δ 168.33, 142.87, 142.47, 141.98, 140.70, 140.54, 140.18, 137.86, 134.41, 133.73, 132.24, 131.81, 131.47, 130.70, 130.56, 130.39, 129.98, 129.95, 129.84, 129.73, 129.21, 128.94, 127.98, 127.23, 126.93, 125.65, 124.91, 123.54, 119.96, 118.23, 117.84, 52.55, 20.80.

HRMS (ESI) calcd for [M+H] $C_{35}H_{26}O_2N_3$, m/z: 520.2020, found: 520.2015.

HPLC analysis: CHIRALCEL IA, hexane/isopropanol = 60/40, 1.0 mL/min, λ = 254 nm, t_R (major) = 4.9 min, t_R (minor) = 10.6 min, ee = 96%.

Supplementary Figure 105. Chiral HPLC spectrum of racemic **4b-12**

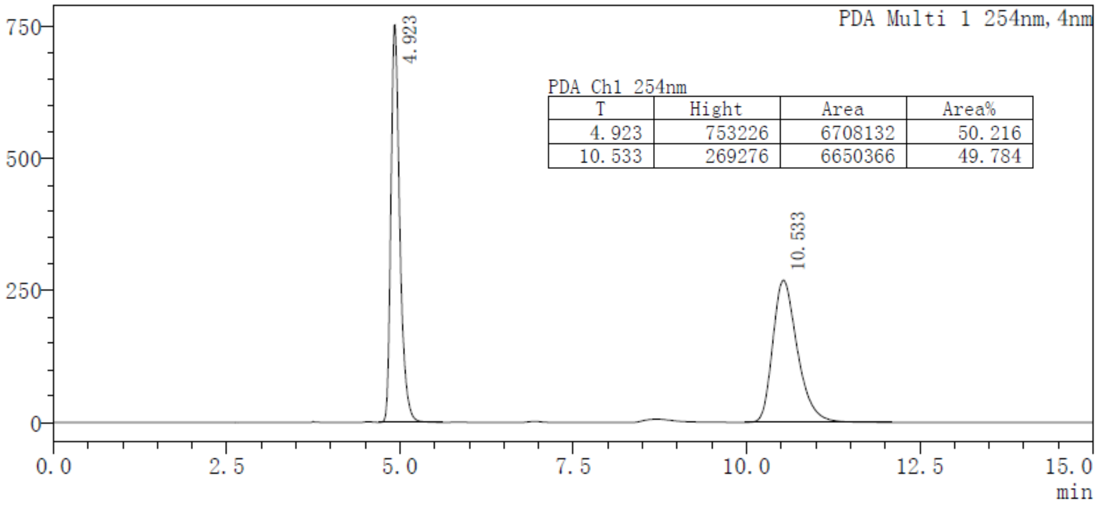

Supplementary Figure 106. Chiral HPLC spectrum of **(*S*)-4b-12**

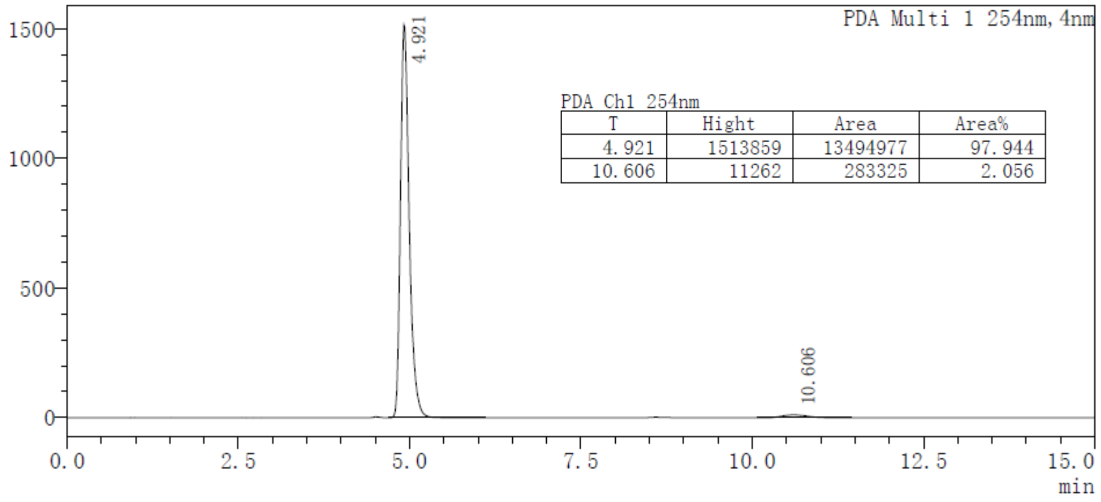

T	Hight	Area	Area%
4.921	1513859	13494977	97.944
10.606	11262	283325	2.056

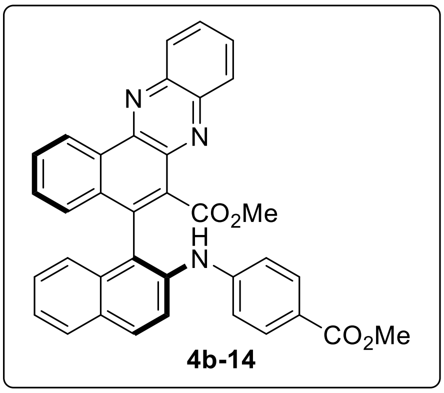

(*S*)-Methyl

5-(2-((4-(methoxycarbonyl)phenyl)amino)naphthalen-1-yl)benzo[a]phenazine-6-carboxylate ((*S*)-4b-14)

According to the general procedure, **(*S*)-4b-14** was obtained in **99% yield** with **98% ee**.

M.P.: 129-131 ℃.

^{1}H NMR (500 MHz, $CDCl_3$) δ 9.48 (d, *J* = 8.2 Hz, 1H), 8.38 (dd, *J* = 8.4, 1.5 Hz, 1H), 8.28 (dd, *J* = 8.4, 1.5 Hz, 1H), 8.00 (d, *J* = 8.9 Hz, 1H), 7.91-7.83 (m, 3H), 7.80 (t, *J* = 9.0 Hz, 3H), 7.75 (t, *J* = 7.5 Hz, 1H), 7.51 (t, *J* = 7.6 Hz, 1H), 7.42-7.38 (m, 1H), 7.33 (d, *J* = 8.1 Hz, 1H), 7.30-7.24 (m, 1H), 6.91 (d, *J* = 8.5 Hz, 2H), 6.51 (s, 1H), 3.81 (s, 3H), 3.65 (s, 3H).

^{13}C NMR (125 MHz, $CDCl_3$) δ 168.69, 166.96, 147.96, 142.85, 142.49, 141.86, 140.44, 137.67, 137.38, 133.89, 133.54, 132.09, 131.66, 131.34, 130.80, 130.49, 130.48, 130.34, 130.24, 129.88, 129.72, 129.24, 128.09, 127.21, 127.06, 125.65, 125.51, 124.97, 123.02, 121.35, 120.97, 115.47, 52.70, 51.77.

HRMS (ESI) calcd for [M+H] $C_{36}H_{26}O_4N_3$, m/z: 564.1918, found: 564.1914.

HPLC analysis: CHIRALCEL IA, hexane/isopropanol = 60/40, 1.0 mL/min, λ = 254 nm, t_R (major) = 6.3 min, t_R (minor) = 18.2 min, ee = 98%.

Supplementary Figure 107. Chiral HPLC spectrum of racemic **4b-14**

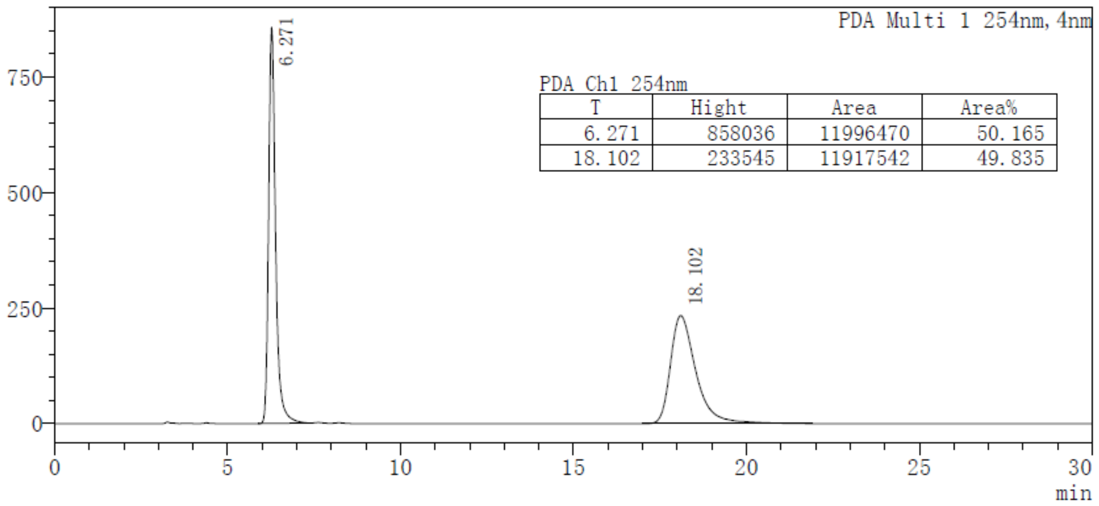

T	Hight	Area	Area%
6.271	858036	11996470	50.165
18.102	233545	11917542	49.835

Supplementary Figure 108. Chiral HPLC spectrum of **(*S*)-4b-14**

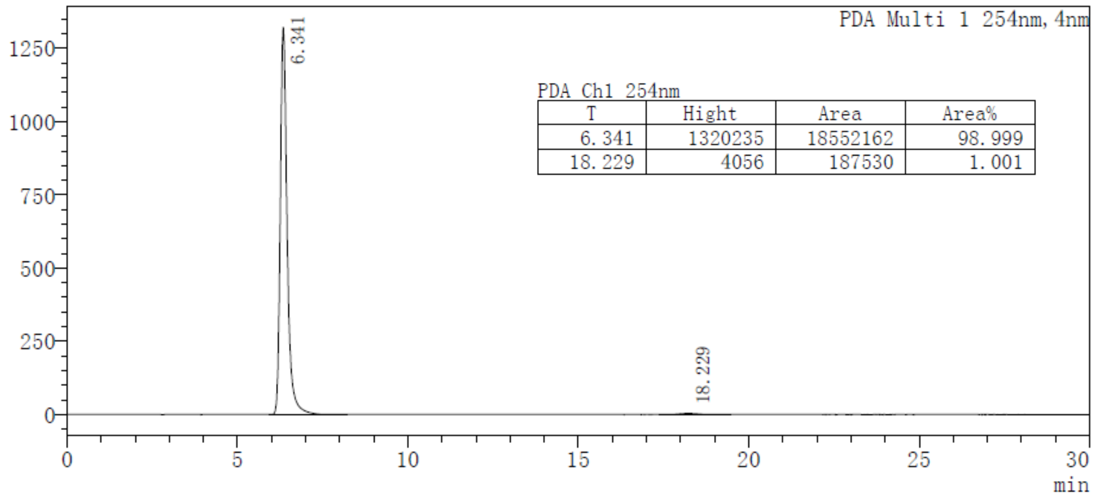

T	Hight	Area	Area%
6.341	1320235	18552162	98.999
18.229	4056	187530	1.001

General Procedure for Synthetic Transformation from 3b to 5b

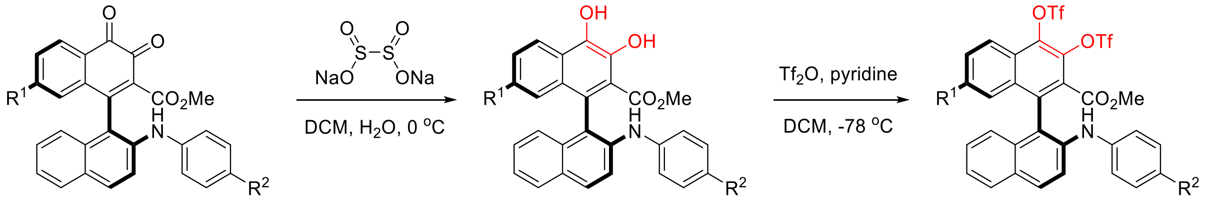

Sodium dithionite (1.5 mmol) was dissolved in 5 mL water and added to a solution of **3b** (0.3 mmol) in CH_2Cl_2 (10 mL), the reaction was completed when the colour changed to yellow. Then the mixture was extracted with CH_2Cl_2, dried over Na_2SO_4, and the solvent was removed under reduced pressure to give *o*-dihydroxybenzene intermediate as a yellow solid. Subsequently, the obtained solid was dissolved in anhydrous CH_2Cl_2 under argon atmosphere and cooled to -78 ℃, then pyridine (0.7 mmol) was added and stirred for 5 min. After that, trifluoromethanesulfonic anhydride (0.5 mmol) was added and stirred for another 2 hours. After completion, the mixture was washed with 3M HCl, dried over Na_2SO_4, the solvent was removed under reduced pressure to give **5b**.

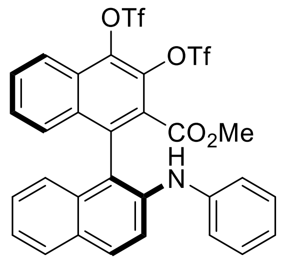

5b-1

(*S*)-Methyl 2'-(phenylamino)-3,4-bis(((trifluoromethyl)sulfonyl)oxy)-[1,1'-binaphthalene]-2-carboxylate ((*S*)-5b-1)

According to the general procedure, **(*S*)-5b-1** was obtained in **99% yield** with **97% ee**.

^{1}H NMR (400 MHz, $CDCl_3$) δ 8.27 (d, *J* = 8.6 Hz, 1H), 7.92 (d, *J* = 8.9 Hz, 1H), 7.84 (d, *J* = 7.5 Hz, 1H), 7.79 (dt, *J* = 8.4, 4.0 Hz, 1H), 7.68 (d, *J* = 9.0 Hz, 1H), 7.52-7.50 (m, 2H), 7.34 (ddd, *J* = 8.1, 6.8, 1.2 Hz, 1H), 7.29-7.20 (m, 3H), 6.99 (dd, *J* = 10.5, 1.5 Hz, 2H), 6.94 (tt, *J* = 7.3, 1.1 Hz, 1H), 6.81 (dd, *J* = 8.3, 1.1 Hz, 1H), 5.57 (s, 1H), 3.53 (s, 3H).

^{13}C NMR (125 MHz, CDCl$_3$) δ 163.88, 142.63, 140.39, 138.53, 136.67, 134.90, 133.60, 132.95, 130.82, 130.37, 129.84, 129.41, 129.30, 128.52, 128.22, 128.16, 127.53, 127.42, 124.14, 124.00, 122.40, 121.91, 119.11, 118.82, 118.73 (q, *J* = 12.8 Hz), 118.61 (q, *J* = 12.8 Hz), 118.44, 53.03.

^{19}F NMR (376 MHz, CDCl$_3$) δ -72.37 (q, *J* = 3.2 Hz), -72.81 (q, *J* = 3.3 Hz).

HRMS (ESI) calcd for [M+H] $C_{30}H_{20}O_8NF_6S_2$, m/z: 700.0529, found: 700.0529.

HPLC analysis: CHIRALCEL IA, hexane/isopropanol = 80/20, 1.0 mL/min, λ = 254 nm, t_R (major) = 3.6 min, t_R (minor) = 4.7 min, ee = 97%.

Supplementary Figure 109. Chiral HPLC spectrum of racemic **5b-1**

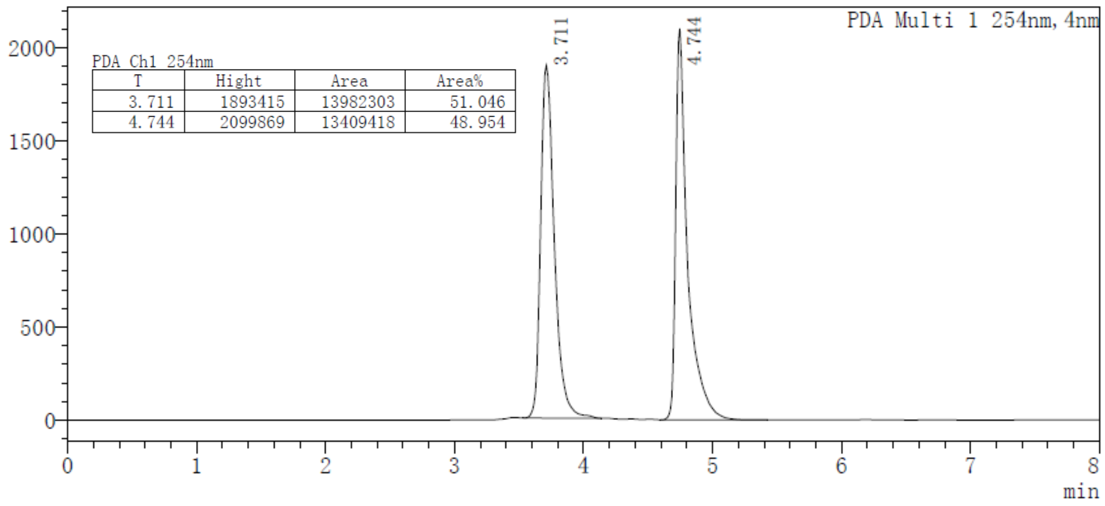

Supplementary Figure 110. Chiral HPLC spectrum of **(*S*)-5b-1**

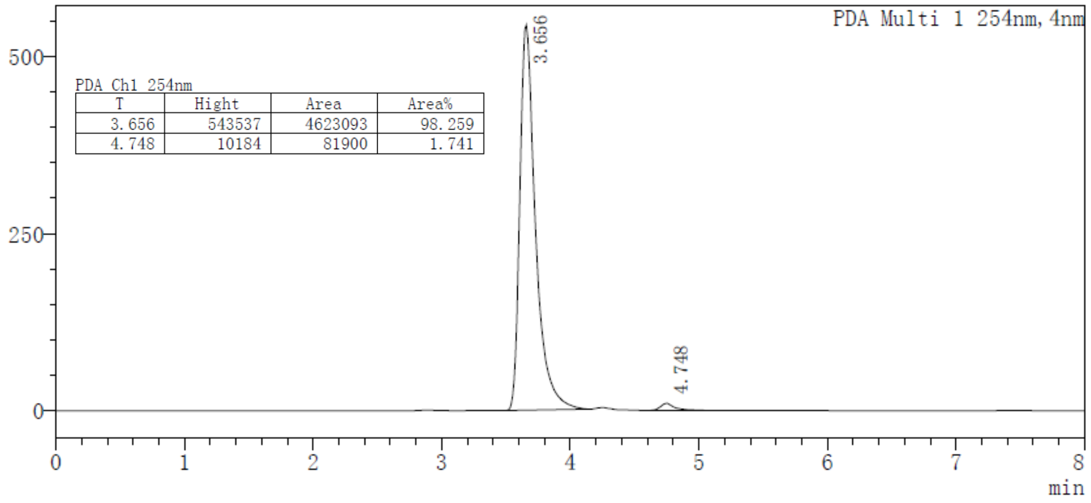

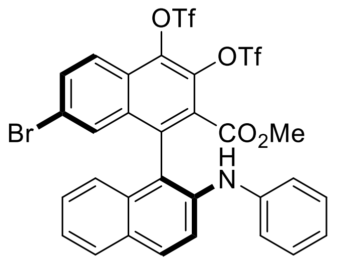

5b-7

(*S*)-Methyl 7-bromo-2'-(phenylamino)-3,4-bis(((trifluoromethyl)sulfonyl)oxy)-[1,1'-binaphthalene]-2-carboxylate ((*S*)-5b-7)

According to the general procedure, **(*S*)-5b-1** was obtained in **99% yield** with **98% ee**.

^{1}H NMR (500 MHz, $CDCl_3$) δ 7.97 (d, *J* = 9.0 Hz, 1H), 7.79 (d, *J* = 9.0 Hz, 1H), 7.72-7.67 (m, 2H), 7.55-7.52 (m, 2H), 7.20 (t, *J* = 7.2 Hz, 1H), 7.16 (t, *J* = 7.6 Hz, 1H), 7.12-7.07 (m, 2H), 6.87 (d, *J* = 7.9 Hz, 2H), 6.80 (t, *J* = 7.4 Hz, 1H), 6.69 (d, *J* = 8.3 Hz, 1H), 5.05 (s, 1H), 3.37 (s, 3H).

^{13}C NMR (125 MHz, $CDCl_3$) δ 163.55, 142.53, 140.55, 137.63, 136.70, 135.01, 134.36, 133.96, 133.34, 130.79, 129.53, 129.49, 129.43, 128.36, 127.61, 127.17, 125.06, 124.22, 124.00, 123.84, 122.10, 119.20, 119.03, 118.68 (q, *J* = 12.8 Hz), 118.56 (q, *J* = 12.8 Hz), 117.88, 53.14.

^{19}F NMR (376 MHz, **$CDCl_3$**) δ -72.35 (q, *J* = 3.1 Hz), -72.79 (q, *J* = 2.9 Hz).

HRMS (ESI) calcd for [M+H] $C_{30}H_{19}O_8N^{79}BrF_6S_2$, m/z: 777.9634, found: 777.9649; $C_{30}H_{19}O_8N^{81}BrF_6S_2$, m/z: 779.9614, found: 779.9609.

HPLC analysis: CHIRALCEL IA, hexane/isopropanol = 80/20, 1.0 mL/min, λ = 254 nm, t_R (major) = 3.5 min, t_R (minor) = 3.9 min, ee = 98%.

Supplementary Figure 111. Chiral HPLC spectrum of racemic **5b-7**

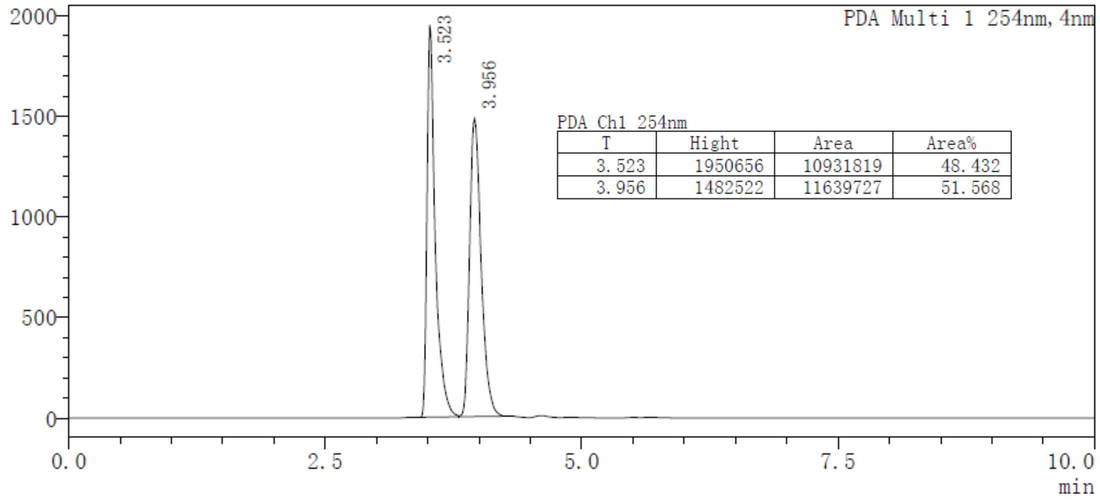

Chiral

Supplementary Figure 112. HPLC spectrum of **(*S*)-5b-7**

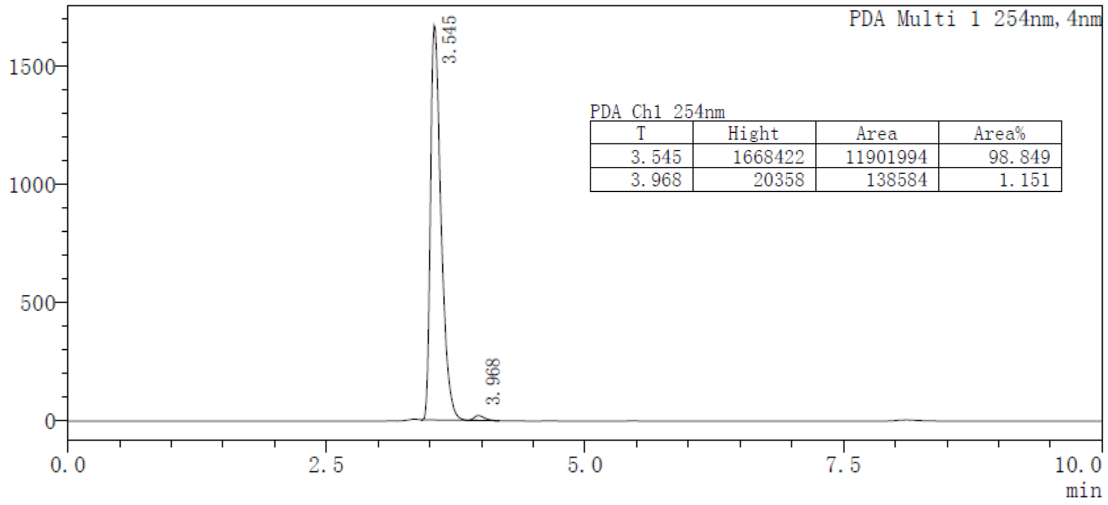

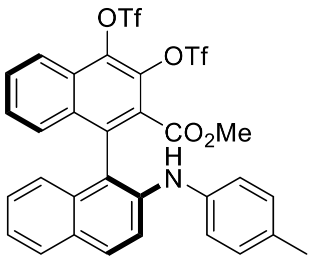

5b-12

(*S*)-Methyl 2'-(p-tolylamino)-3,4-bis(((trifluoromethyl)sulfonyl)oxy)-[1,1'-binaphthalene]-2-carboxylate ((*S*)-5b-12)

According to the general procedure, **(*S*)-5b-1** was obtained in **99% yield** with **96% ee**.

^{1}H NMR (500 MHz, $CDCl_3$) δ 8.34 (d, *J* = 8.5 Hz, 1H), 7.94 (d, *J* = 9.1 Hz, 1H), 7.87 (dd, *J* =

8.1, 1.3 Hz, 1H), 7.83 (ddd, J = 8.3, 6.7, 1.3 Hz, 1H), 7.67 (d, J = 9.0 Hz, 1H), 7.59 (d, J = 8.5 Hz, 1H), 7.54 (ddd, J = 8.5, 6.8, 1.1 Hz, 1H), 7.37 - 7.33 (m, 2H), 7.30 (ddd, J = 8.3, 6.8, 1.4 Hz, 1H), 7.11 (d, J = 8.0 Hz, 2H), 7.00 (d, J = 8.3 Hz, 2H), 6.87 (d, J = 8.3 Hz, 1H), 5.03 (s, 1H), 3.57 (s, 3H), 2.33 (s, 3H).

^{13}C NMR (125 MHz, CDCl$_3$) δ 163.84, 141.04, 139.80, 138.52, 136.68, 134.93, 133.64, 132.98, 132.04, 130.80, 130.35, 129.97, 129.83, 128.95, 128.54, 128.30, 128.19, 127.58, 127.37, 123.98, 123.69, 122.40, 120.24, 118.73 (q, J = 12.8 Hz), 118.61 (q, J = 12.8 Hz), 118.17, 117.13, 52.98, 20.76.

^{19}F NMR (376 MHz, CDCl$_3$) δ -72.35 (q, J = 3.3 Hz), -72.79 (q, J = 3.1 Hz).

HRMS (ESI) calcd for [M+H] $C_{31}H_{22}O_8NF_6S_2$, m/z: 714.0686, found: 714.0682.

HPLC analysis: CHIRALCEL IA, hexane/isopropanol = 80/20, 1.0 mL/min, λ = 254 nm, t_R (major) = 3.7 min, t_R (minor) = 6.2 min, ee = 96%.

Supplementary Figure 113. Chiral HPLC spectrum of racemic **5b-12**

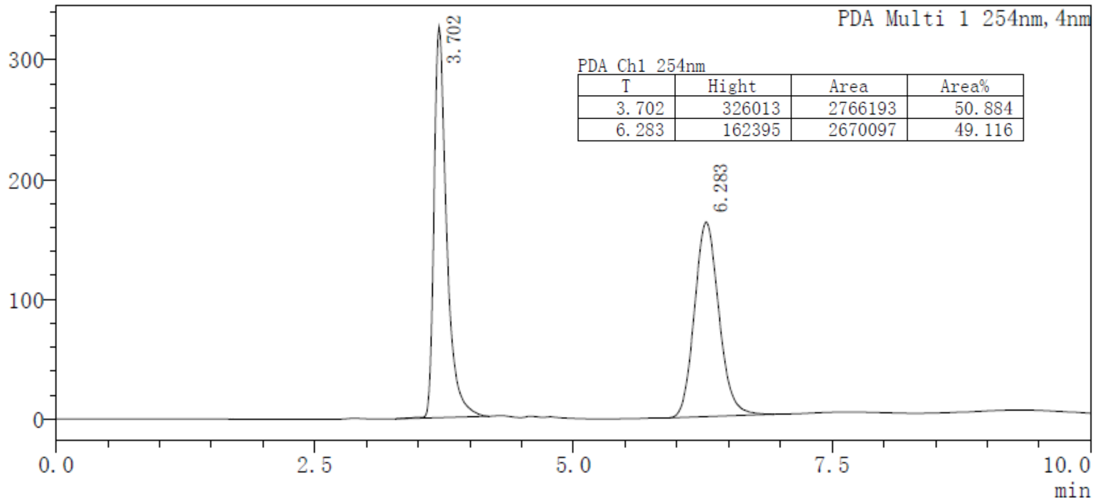

Supplementary Figure 114. Chiral HPLC spectrum of **(*S*)-5b-12**

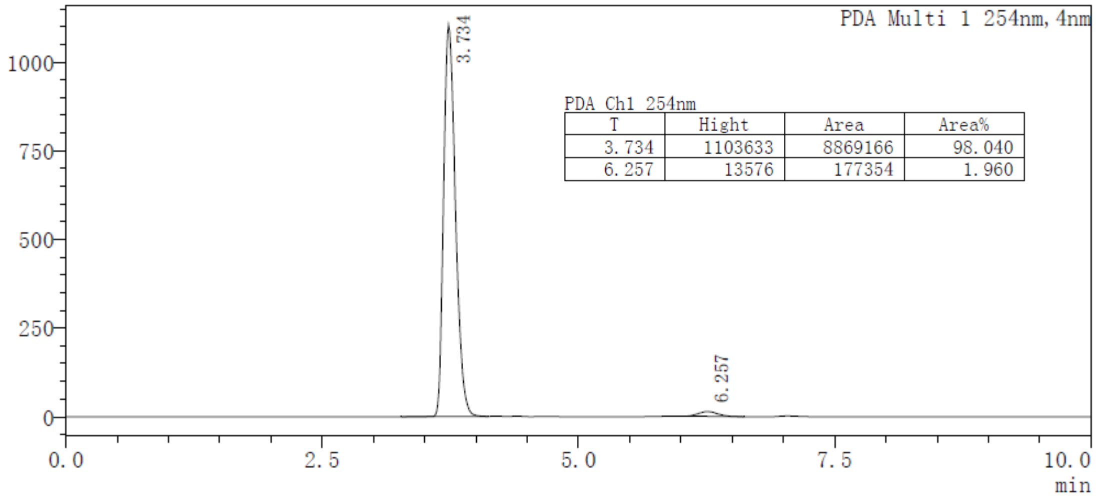

T	Hight	Area	Area%
3.734	1103633	8869166	98.040
6.257	13576	177354	1.960

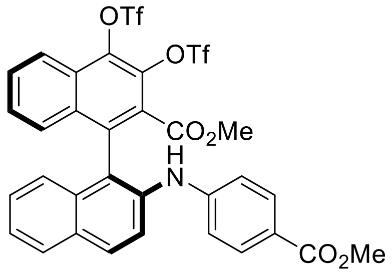

5b-14

(*S*)-Methyl 2'-((4-(methoxycarbonyl)phenyl)amino)-3,4-bis(((trifluoromethyl)sulfonyl)oxy)-[1,1'-binaphthalene]-2-carboxylate ((*S*)-5b-14)

According to the general procedure, **(*S*)-5b-14** was obtained in **99% yield** with **98% ee**.

^{1}H NMR (400 MHz, CDCl$_3$) δ 8.24 (d, *J* = 8.5 Hz, 1H), 7.99 (d, *J* = 8.9 Hz, 1H), 7.89 (d, *J* = 8.1 Hz, 1H), 7.83 (d, *J* = 8.8 Hz, 2H), 7.79 - 7.72 (m, 2H), 7.47 (ddd, *J* = 8.2, 6.9, 1.2 Hz, 1H), 7.43 - 7.37 (m, 2H), 7.29 (ddd, *J* = 8.5, 7.0, 1.3 Hz, 1H), 6.88 - 6.81 (m, 3H), 6.00 (s, 1H), 3.84 (s, 3H), 3.53 (s, 3H).

^{19}F NMR (376 MHz, CDCl$_3$) δ -72.36 (q, *J* = 3.2 Hz), -72.78 (q, *J* = 3.4 Hz).

^{13}C NMR (125 MHz, CDCl$_3$) δ 166.90, 164.00, 147.76, 138.23, 136.65, 134.76, 133.38, 132.77, 131.40, 130.86, 130.57, 130.42, 129.83, 128.40, 128.29, 127.60, 127.35, 125.11, 124.60, 122.64, 122.38, 121.65, 121.14, 118.67 (q, *J* = 12.8 Hz), 118.57 (q, *J* = 12.8 Hz), 115.36, 53.17, 51.76.

HRMS (ESI) calcd for [M+H] $C_{32}H_{22}O_{10}NF_6S_2$, m/z: 758.0584, found: 758.0588.

HPLC analysis: CHIRALCEL IA, hexane/isopropanol = 80/20, 1.0 mL/min, λ = 254 nm, t_R (major) = 5.3 min, t_R (minor) = 11.2 min, ee = 98%.

Supplementary Figure 115. Chiral HPLC spectrum of racemic **5b-14**

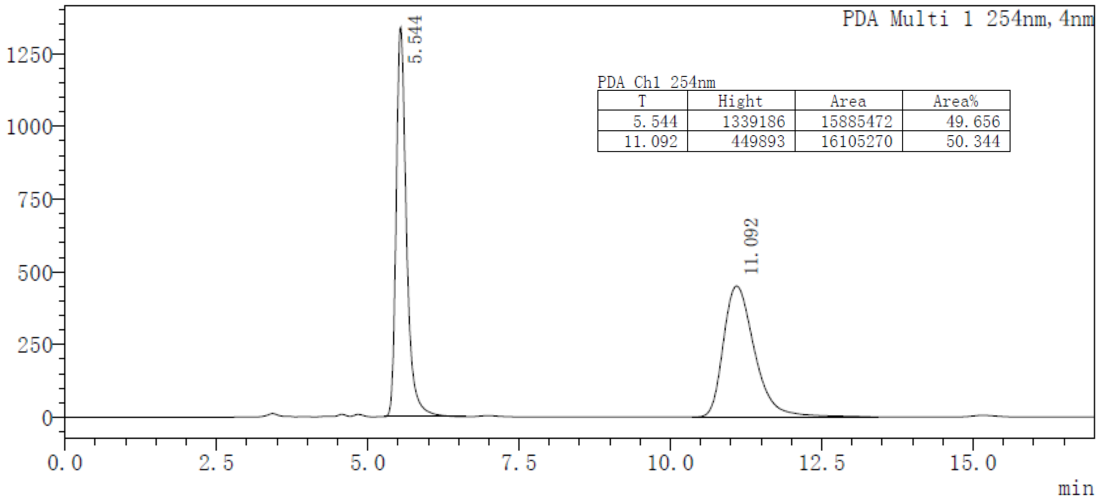

Supplementary Figure 116. Chiral HPLC spectrum of **(*S*)-5b-14**

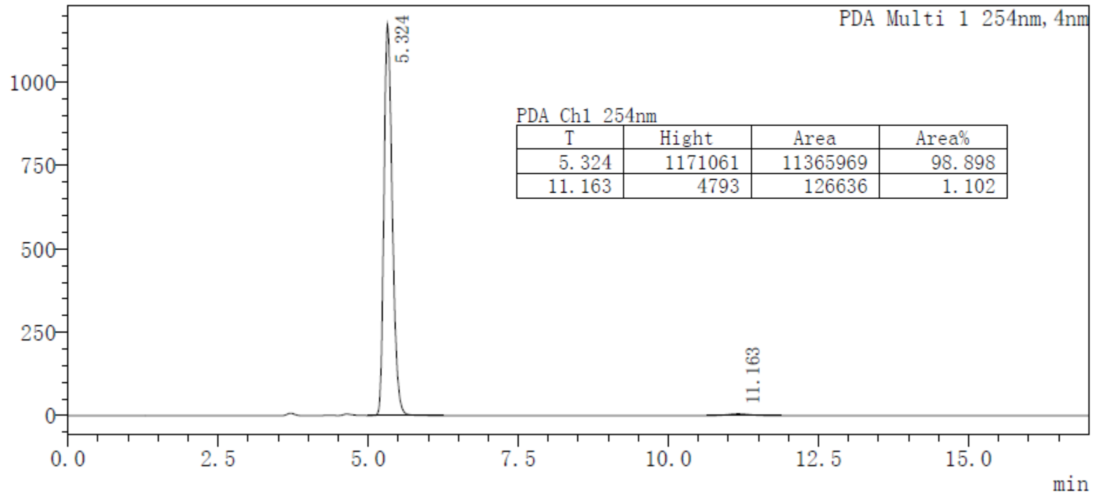

Versatile Coupling Reactions

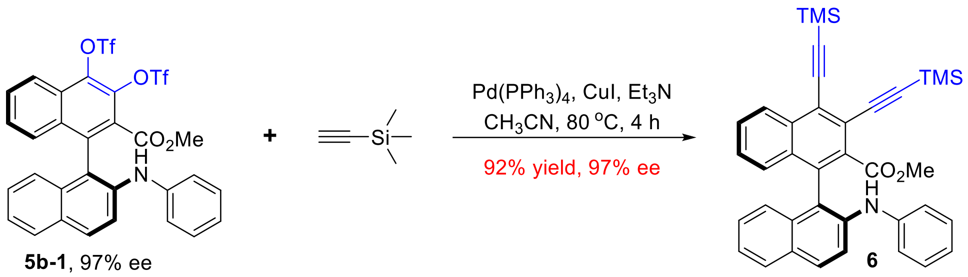

(*S*)-Methyl -2'-(phenylamino)-3,4-bis((trimethylsilyl)ethynyl)-[1,1'-binaphthalene]-2-carboxylate ((*S*)-6)

Compound **5b-1** (0.20 mmol), $Pd(PPh_3)_4$ (0.02 mmol) and copper iodide (0.04 mmol) were placed in a 10 mL Schlenk tube under argon atmosphere. Then trimethylsilylacetylene (0.5 mL), triethylamine (1.5 mL) and dioxane (1.5 mL) were added via syringes. The Schlenk tube was sealed and the solution was allowed to stir at 80 ℃ for 4 hours. After completion, the reaction mixture was concentrated under reduced pressure and the residue was purified by column chromatography to give compound **(*S*)-6** in **92% yield** with **97% ee**.

^{1}H NMR (400 MHz, $CDCl_3$) δ 7.99 (d, *J* = 8.5 Hz, 1H), 7.74 (d, *J* = 8.9 Hz, 1H), 7.69 (d, *J* = 8.0 Hz, 1H), 7.57 (d, *J* = 8.9 Hz, 1H), 7.36 (t, *J* = 7.4 Hz, 1H), 7.21-7.04 (m, 7H), 6.88 (t, *J* = 8.1 Hz, 3H), 6.79 (t, *J* = 7.2 Hz, 1H), 5.59 (s, 1H), 3.25 (s, 3H), 0.29 (s, 9H), 0.18 (s, 9H).

^{13}C NMR (100 MHz, $CDCl_3$) δ 168.00, 143.25, 141.38, 140.01, 139.57, 134.60, 132.46, 132.36, 131.18, 130.28, 129.27, 129.21, 129.20, 129.12, 128.72, 128.59, 127.77, 126.60, 126.56, 126.37, 126.29, 125.41, 125.28, 123.39, 122.73, 121.26, 119.88, 118.82, 118.25, 51.96, 1.20, 0.89.

HRMS (ESI) calcd for [M+H] $C_{38}H_{38}NO_2Si_2$, m/z: 596.2441, found: 596.2436.

HPLC analysis: CHIRALCEL IA, hexane/isopropanol = 95/05, 1.0 mL/min, λ = 254 nm, t_R (major) = 3.5 min, t_R (minor) = 4.5 min, ee = 97%.

Supplementary Figure 117. Chiral HPLC spectrum of racemic **6**

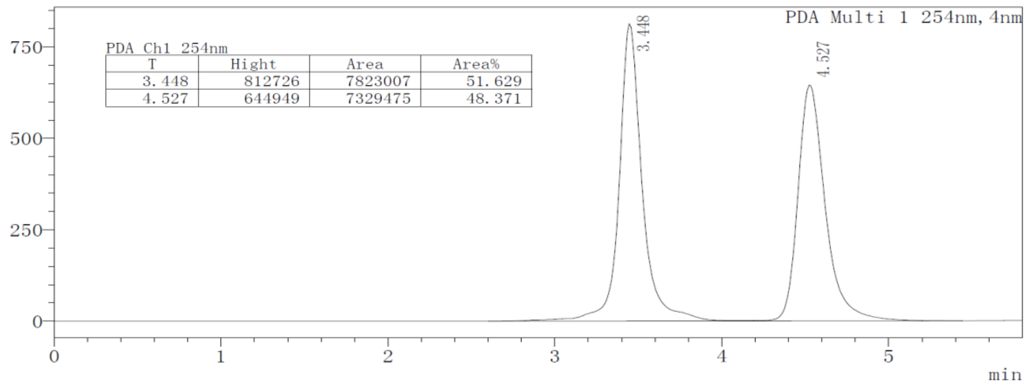

Supplementary Figure 118. Chiral HPLC spectrum of **(*S*)-6**

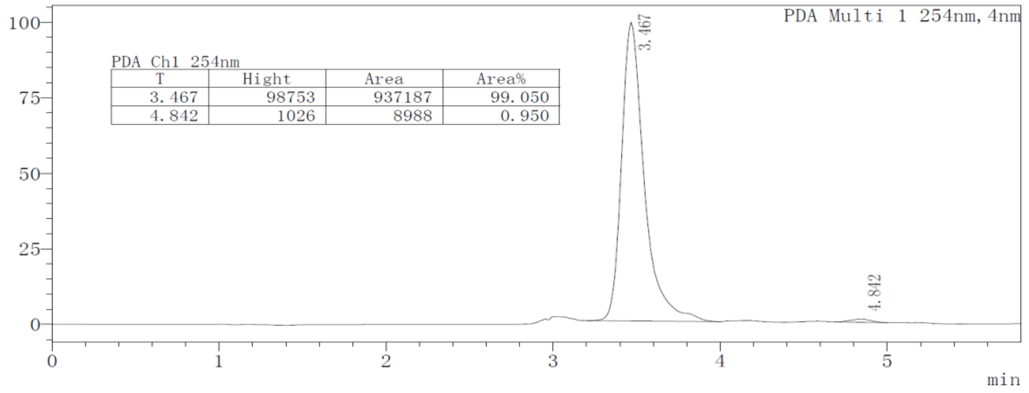

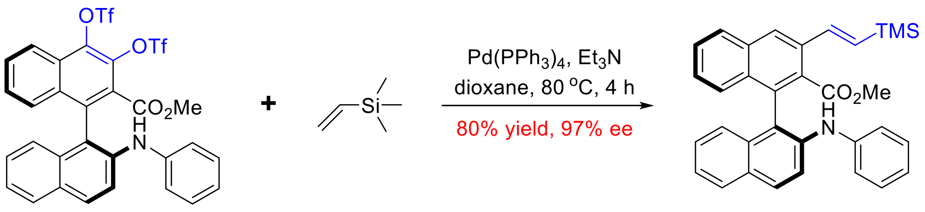

5b-1, 97% ee

7

(*S*)-Methyl-2'-(phenylamino)-3-(2-(trimethylsilyl)vinyl)-[1,1'-binaphthalene]-2-carboxylate ((*S*)-7)

5b-1 (0.20 mmol) and $Pd(PPh_3)_4$ (0.02 mmol) were placed in a 10 mL Schlenk tube under argon atmosphere. Then vinyltrimethylsilane (0.5 mL), triethylamine (1.5 mL) and dioxane (1.5 mL) were added via syringes. The Schlenk tube was sealed and the solution was allowed to stir at 80 ℃ for 4 hours. After completion, the reaction mixture was concentrated under reduced pressure and the residue was purified by column chromatography to give compound **(*S*)-7** in **80% yield** with **97% ee**.

^{1}H NMR (400 MHz, DMSO-d_6) δ 8.43 (s, 1H), 8.10 (d, *J* = 8.2 Hz, 1H), 7.91 (d, *J* = 8.2 Hz, 1H), 7.85 (d, *J* = 8.0 Hz, 1H), 7.58-7.52 (m, 2H), 7.34-7.24 (m, 2H), 7.18-7.10 (m, 4H), 6.97

(d, J = 8.6 Hz, 2H), 6.84-6.79 (m, 2H), 6.68 (d, J = 8.4 Hz, 1H), 6.61 (s, 1H), 3.29 (s, 2H), 0.16 (s, 9H).

^{13}C NMR (100 MHz, DMSO-d$_6$) δ 168.64, 143.57, 140.50, 139.86, 133.82, 133.77, 132.85, 132.69, 132.49, 131.77, 129.08, 128.87, 128.61, 127.81, 127.47, 127.31, 126.35, 125.76, 125.20, 124.51, 123.22, 120.51, 119.95, 119.35, 118.36, 51.59, -1.25.

HRMS (ESI) calcd for [M+H] $C_{33}H_{32}NO_2Si$, m/z: 502.2202, found: 502.2194.

HPLC analysis: CHIRALCEL IA, hexane/isopropanol = 95/05, 1.0 mL/min, λ = 254 nm, t_R (major) = 9.5 min, t_R (minor) = 13.6 min, ee = 97%.

Supplementary Figure 119. Chiral HPLC spectrum of racemic **7**

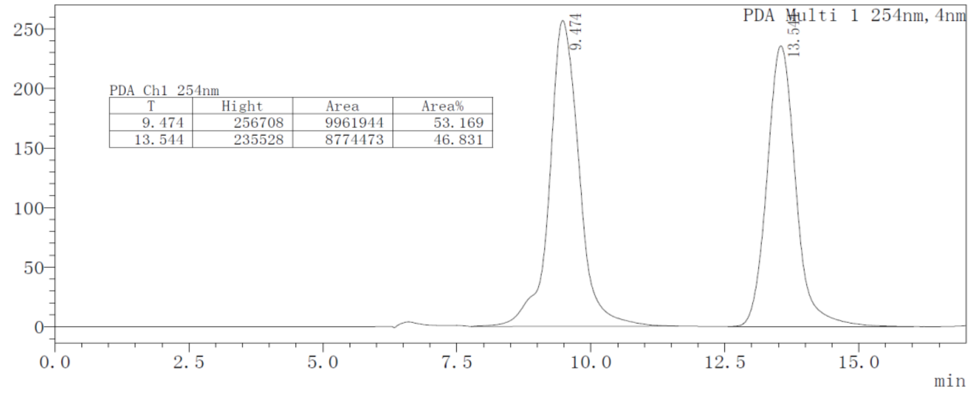

Supplementary Figure 120. Chiral HPLC spectrum of **(*S*)-7**

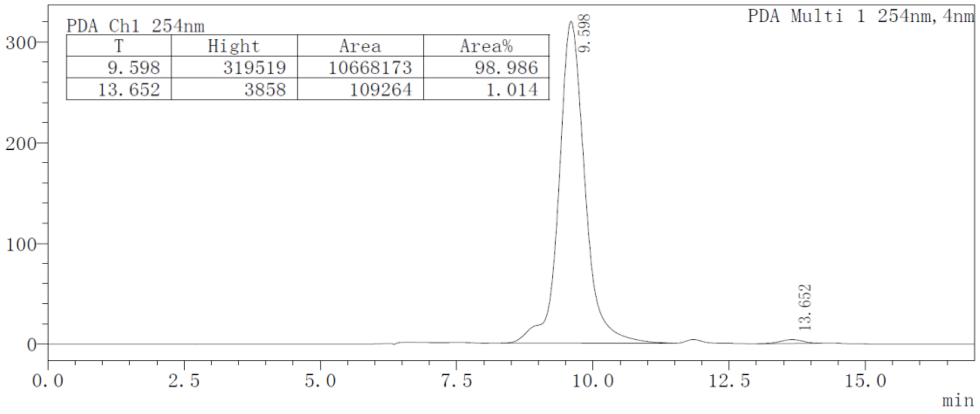

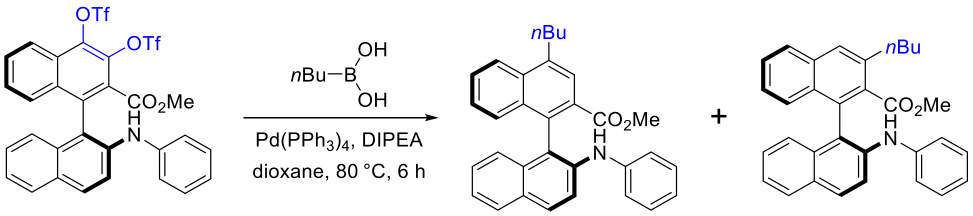

5b-1, 97% ee **8a**, 33% yield, 97% ee **8b**, 52% yield, 97% ee

Compound **5b-1** (0.20 mmol), *n*-butylboronic acid (2.0 mmol) and Pd(PPh$_3$)$_4$ (0.02 mmol) were placed in a 10 mL Schlenk tube under argon atmosphere. Then DIPEA (0.5 mL) and

dioxane (2 mL) were added via syringes. The Schlenk tube was sealed and the solution was allowed to stir at 80 ℃ for 6 hours. After completion, the reaction mixture was concentrated under reduced pressure and the residue was purified by column chromatography to give isolatable compounds **8a** and **8b**.

(*S*)-Methyl-4-butyl-2'-(phenylamino)-[1,1'-binaphthalene]-2-carboxylate ((*S*)-8a)

(*S*)-8a was obtained in **33% yield** with **97% ee**.

^{1}H NMR (400 MHz, DMSO-d_6) δ 8.17 (d, *J* = 8.5 Hz, 1H), 7.90 (s, 1H), 7.88 (d, *J* = 9.0 Hz, 1H), 7.84 (d, *J* = 8.1 Hz, 1H), 7.60-7.55 (m, 1H), 7.53 (d, *J* = 8.9 Hz, 1H), 7.29 (t, 1H), 7.26-7.21 (m, 2H), 7.12-7.05 (m, 3H), 6.88 (d, *J* = 8.1 Hz, 2H), 6.72-6.68 (m, 2H), 6.58 (d, *J* = 8.4 Hz, 1H), 3.40 (s, 3H), 3.15 (dd, *J* = 8.3, 4.9 Hz, 2H), 1.76 (dt, *J* = 15.4, 7.7 Hz, 2H), 1.48 (td, *J* = 14.6, 7.2 Hz, 2H), 0.96 (t, *J* = 7.3 Hz, 3H).

^{13}C NMR (100 MHz, DMSO-d_6) δ 166.75, 144.41, 139.06, 138.83, 134.94, 133.90, 133.50, 132.81, 129.16, 128.80, 128.77, 128.16, 127.89, 127.84, 127.48, 126.50, 126.19, 125.63, 124.19, 124.11, 123.96, 123.25, 120.58, 119.62, 117.25, 51.65, 32.58, 32.15, 22.37, 13.95.

HRMS (ESI) calcd for [M+H] $C_{32}H_{30}NO_2$, m/z: 460.2277, found: 460.2269.

HPLC analysis: CHIRALCEL IA, hexane/isopropanol = 95/05, 1.0 mL/min, λ = 254 nm, t_R (major) = 5.1 min, t_R (minor) = 6.1 min, ee = 97%.

Supplementary Figure 121. Chiral HPLC spectrum of racemic **8a**

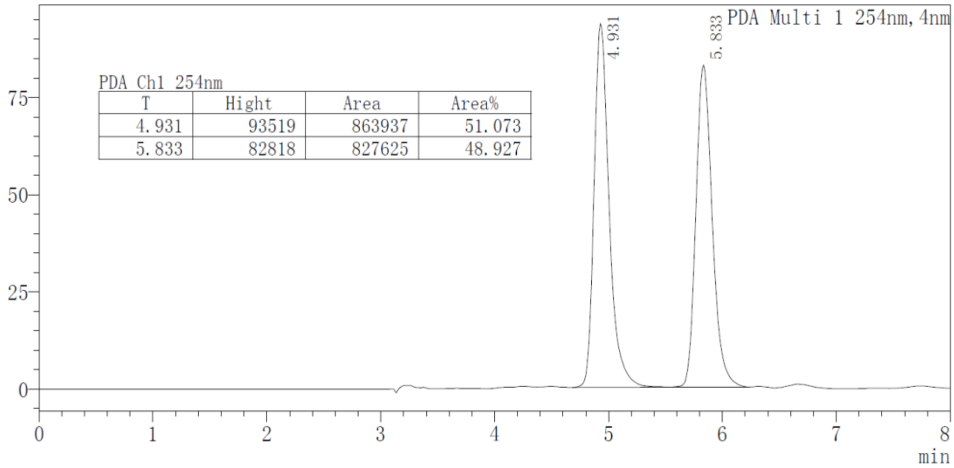

Supplementary Figure 122. Chiral HPLC spectrum of **(*S*)-8a**

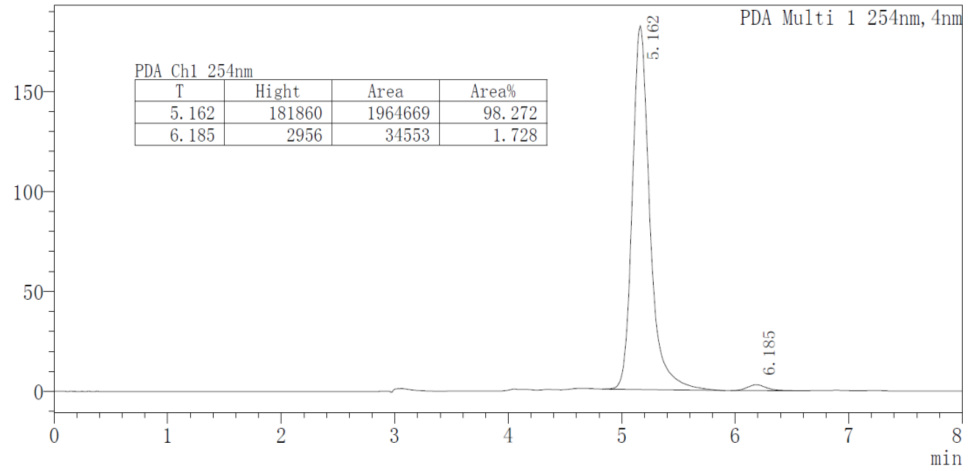

(*S*)-Methyl-3-butyl-2'-(phenylamino)-[1,1'-binaphthalene]-2-carboxylate ((*S*)-8b)

(*S*)-8b was obtained in **52% yield** with **97% ee**.

^{1}H NMR (400 MHz, DMSO-d$_6$) δ 7.96 (d, *J* = 8.3 Hz, 1H), 7.94 (s, 1H), 7.88 (d, *J* = 9.0 Hz, 1H), 7.81 (d, *J* = 8.0 Hz, 1H), 7.53 (d, *J* = 9.0 Hz, 1H), 7.48 (t, *J* = 7.5 Hz, 1H), 7.26-7.20 (m, 2H), 7.13 (t, *J* = 7.9 Hz, 3H), 7.06 (d, *J* = 8.4 Hz, 1H), 6.92 (d, *J* = 7.9 Hz, 2H), 6.78 (t, *J* = 7.3 Hz, 1H), 6.64 (d, *J* = 8.4 Hz, 1H), 6.40 (s, 1H), 3.24 (s, 3H), 2.73 (t, *J* = 7.8 Hz, 2H), 1.65 (dt, *J* = 14.9, 7.6 Hz, 2H), 1.36 (dq, *J* = 14.4, 7.2 Hz, 2H), 0.88 (t, *J* = 7.3 Hz, 3H).

^{13}C NMR (100 MHz, DMSO-d$_6$) δ 168.96, 143.43, 139.70, 136.21, 134.09, 133.88, 133.84, 132.03, 130.55, 129.01, 128.92, 128.62, 127.92, 127.84, 127.78, 127.15, 126.41, 126.34, 125.64, 124.56, 123.19, 120.57, 120.33, 119.14, 118.35, 51.54, 32.92, 32.68, 22.13, 13.83.

HRMS (ESI) calcd for [M+H] $C_{32}H_{30}NO_2$, m/z: 460.2277, found: 460.2267.

HPLC analysis: CHIRALCEL IA, hexane/isopropanol = 95/05, 1.0 mL/min, λ = 254 nm, t_R (major) = 4.6 min, t_R (minor) = 7.2 min, ee = 97%.

Supplementary Figure 123. Chiral HPLC spectrum of racemic **8b**

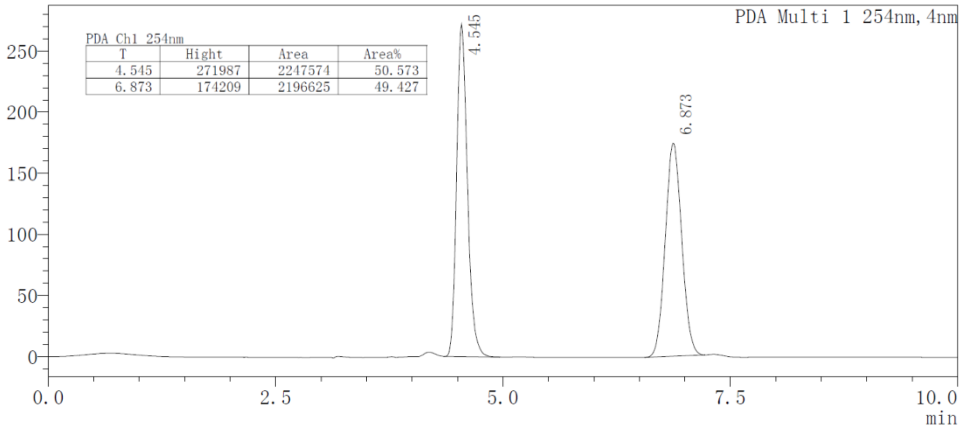

Supplementary Figure 124. Chiral HPLC spectrum of ***(S)*-8b**

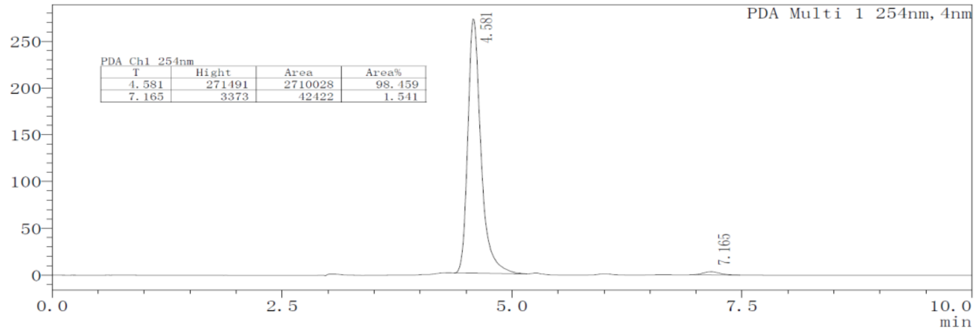

T	Hight	Area	Area%
4.581	271491	2710028	98.459
7.165	3373	42422	1.541

The Synthesis and Characterization of 9

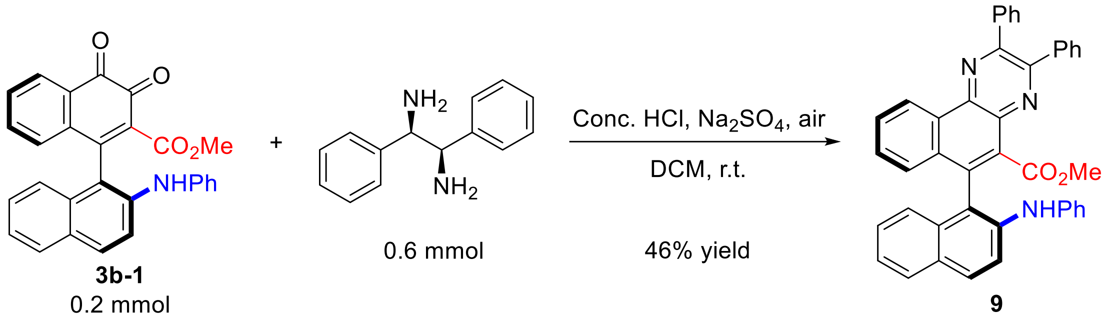

3b-1 (0.20 mmol), **2e** (0.60 mmol) and Na_2SO_4 (50 mg) was dissolved in 10 mL CH_2Cl_2, and Conc. HCl (20 μL) was added. The reaction was stirred for 8 hours under air atmosphere. After completion, the mixture was extracted with CH_2Cl_2, dried over Na_2SO_4, and the solvent was removed under reduced pressure. The residue was purified by column chromatography to give **9** in **46% yield** as a yellow solid.

M.P.: 120-122 ℃.

^{1}H NMR (400 MHz, DMSO-d$_6$) δ 9.30 (d, *J* = 8.1 Hz, 1H), 7.95 (d, *J* = 9.1 Hz, 1H), 7.89-7.82 (m, 2H), 7.66-7.63 (m, 2H), 7.60 (t, *J* = 8.3 Hz, 2H), 7.53-7.49 (m, 2H), 7.45-7.43 (m, 2H), 7.39-7.32 (m, 4H), 7.30-7.25 (m, 2H), 7.22-7.14 (m, 3H), 7.02-6.94 (m, 4H), 6.84 (t, *J* = 7.3 Hz, 1H), 3.41 (s, 3H).

^{13}C NMR (100 MHz, DMSO-d$_6$) δ 167.13, 151.82, 151.59, 143.27, 140.25, 138.84, 138.49, 138.30, 137.48, 135.84, 133.44, 132.99, 132.50, 130.96, 129.85, 129.66, 129.50, 129.03, 128.97, 128.80, 128.69, 128.36, 128.32, 128.29, 127.89, 126.59, 124.42, 124.34, 123.13, 120.96, 119.19, 118.80, 117.74, 51.74.

HRMS (ESI) calcd for [M+H] $C_{42}H_{30}N_3O_2$, m/z: 608.2338, found: 608.2325.

M.P. 120-123 ^{o}C.

HPLC analysis: CHIRALCEL IA, hexane/isopropanol = 70/30, 1.0 mL/min, λ = 254 nm, t_R (major) = 4.6 min, t_R (minor) = 7.2 min, ee = 97%.

Supplementary Figure 125. Chiral HPLC spectrum of racemic **9**

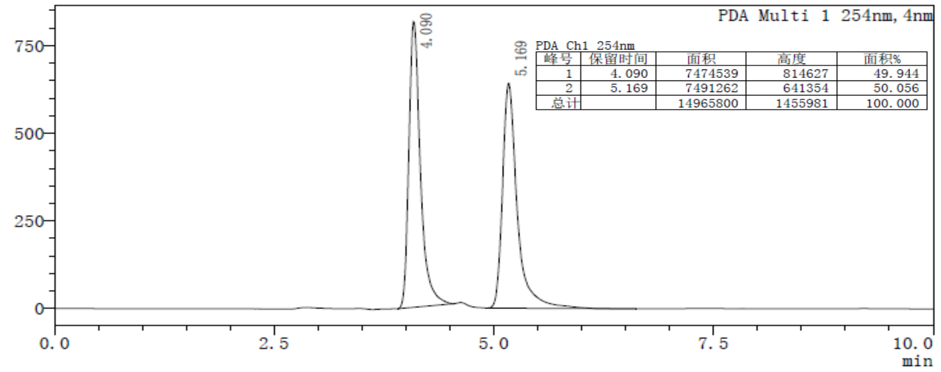

Supplementary Figure 126. Chiral HPLC spectrum of ***(S)*-9**

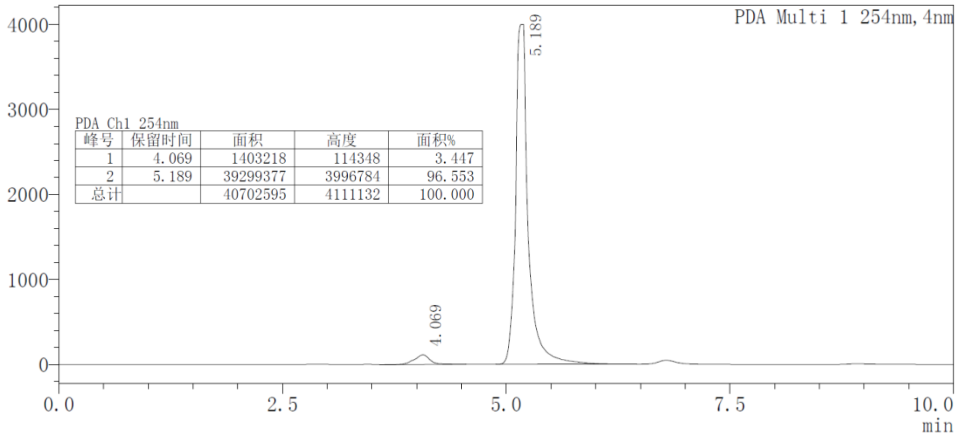

Reduction Reactions

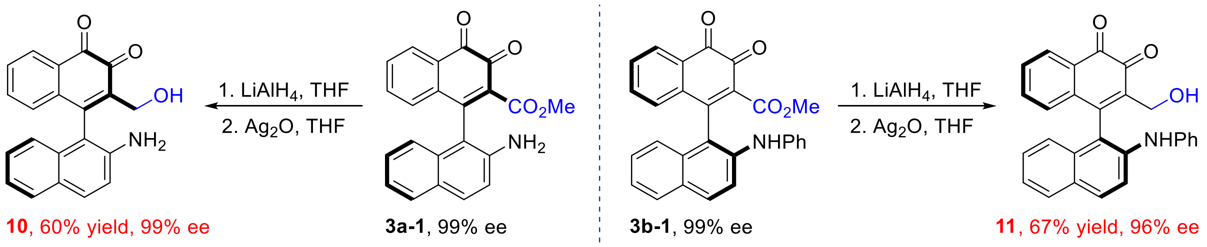

General procedure: *o*-naphthoquinone (**3a-1** or **3b-1**, 0.5 mmol) was dissolved in 20 mL dry THF and cooled to 0 ℃. Then $LiAH_4$ (10 mmol) was added in portion. After stirred for 5 hours, the reaction was cooled to 0 ℃. Water (10 mL) was added and followed 3M HCl (20 mL). The solution was extracted with DCM and dried over Na_2SO_4. The solvent was removed under reduced pressure. Then the residue was dissolved in 20 mL THF and silver oxide (2 mmol) was added. After completion, the residue was filtered through a plug of silica and purified by column chromatography.

(*R*)-2'-Amino-2-(hydroxymethyl)-[1,1'-binaphthalene]-3,4-dione ((*R*)-10)

According to the general procedure, **(*R*)-10** was obtained in **60% yield** with **99% ee**.

^{1}H NMR (400 MHz, DMSO-d$_6$) δ 8.04 (dd, *J* = 7.4, 1.5 Hz, 1H), 7.76 (t, *J* = 8.6 Hz, 2H), 7.49 (td, *J* = 7.4, 1.3 Hz, 1H), 7.44 (td, *J* = 7.6, 1.6 Hz, 1H), 7.38 (d, *J* = 8.4 Hz, 1H), 7.25-7.20 (m, 1H), 7.17-7.10 (m, 2H), 6.55 (dd, *J* = 7.7, 1.0 Hz, 1H), 5.39 (s, 1H), 4.29 (s, 1H), 4.02 (d, *J* = 10.7 Hz, 1H), 3.85 (d, *J* = 10.7 Hz, 1H).

^{13}C NMR (100 MHz, DMSO-d$_6$) δ 180.24, 179.15, 148.61, 142.83, 139.39, 135.82, 135.21, 132.62, 132.32, 129.93, 129.41, 128.57, 128.16, 127.93, 126.57, 126.17, 123.37, 121.34, 118.35, 109.55, 56.19.

HRMS (ESI) calcd for [M+H] $C_{21}H_{16}NO_3$, m/z: 330.1130, found: 330.1123.

HPLC analysis: CHIRALCEL IB, hexane/isopropanol = 70/30, 1.0 mL/min, λ = 254 nm, t_R (major) = 10.7 min, t_R (minor) = 19.3 min, ee = 99%.

Supplementary Figure 127. Chiral HPLC spectrum of racemic **10**

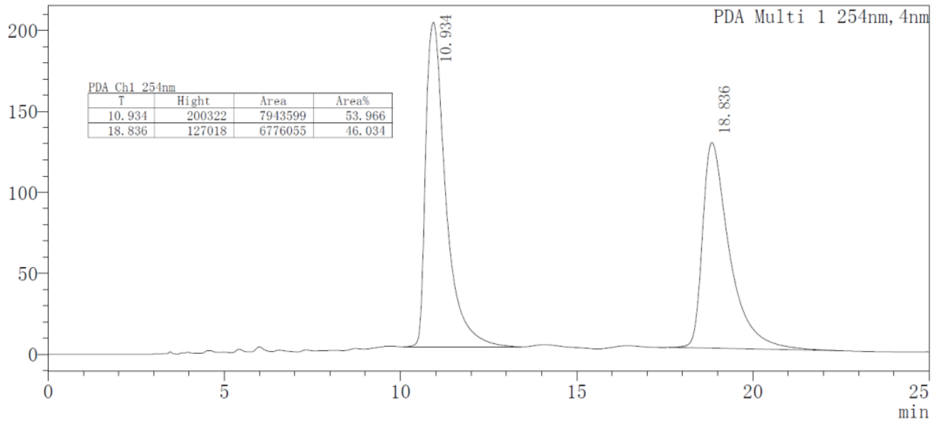

Supplementary Figure 128. Chiral HPLC spectrum of **(*R*)-10**

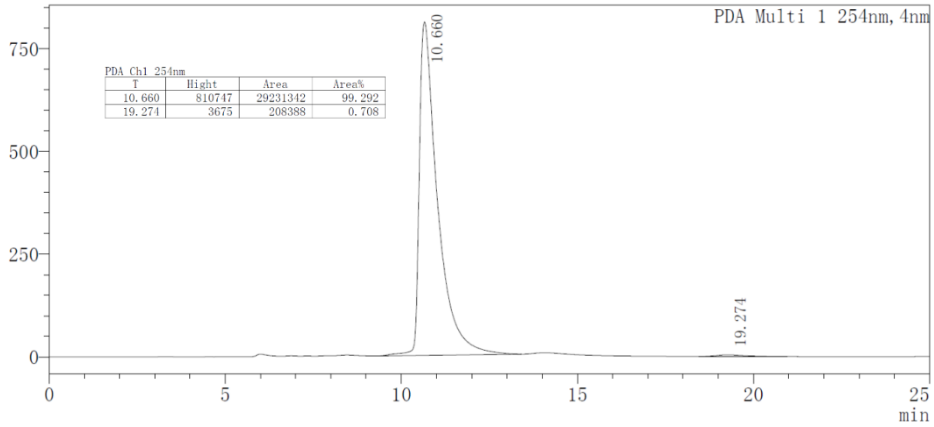

(*S*)-2-(Hydroxymethyl)-2'-(phenylamino)-[1,1'-binaphthalene]-3,4-dione ((*S*)-11)

According to the general procedure, **(*S*)-11** was obtained in **67% yield** with **96% ee**.

^{1}H NMR (400 MHz, CDCl$_3$) δ 8.16 (d, *J* = 7.3 Hz, 1H), 7.90-7.83 (m, 2H), 7.66 (d, *J* = 9.0 Hz, 1H), 7.47-7.36 (m, 5H), 7.28-7.20 (m, 3H), 7.02 (d, *J* = 7.6 Hz, 2H), 6.94 (t, *J* = 7.3 Hz, 1H), 6.84 (d, *J* = 7.5 Hz, 1H), 6.33 (s, 1H), 4.54 (d, *J* = 12.2 Hz, 1H), 3.98 (d, *J* = 12.1 Hz, 1H).

^{13}C NMR (100 MHz, DMSO-d$_6$) δ 179.80, 179.07, 147.84, 142.81, 139.60, 138.86, 136.03, 135.20, 132.31, 130.06, 129.41, 129.12, 128.61, 128.23, 128.04, 127.77, 126.93, 124.41, 123.41, 121.47, 119.43, 118.06, 117.03, 56.02.

HRMS (ESI) calcd for [M+Na] $C_{27}H_{19}NO_3Na$, m/z: 428.1263, found: 428.1256.

HPLC analysis: CHIRALCEL IC, hexane/isopropanol = 80/20, 1.0 mL/min, λ = 254 nm, t_R (minor) = 38.5 min, t_R (major) = 57.2 min, ee = 96%.

Supplementary Figure 129. Chiral HPLC spectrum of racemic **11**

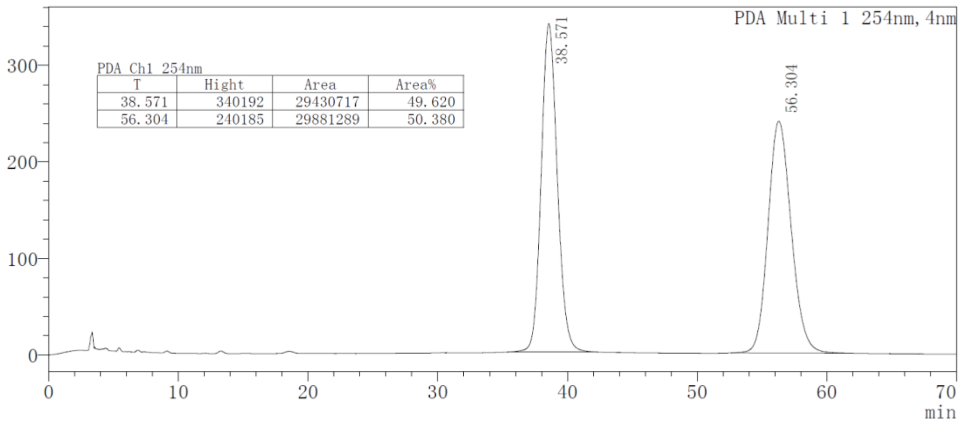

Supplementary Figure 130. Chiral HPLC spectrum of ***(S)*-11**

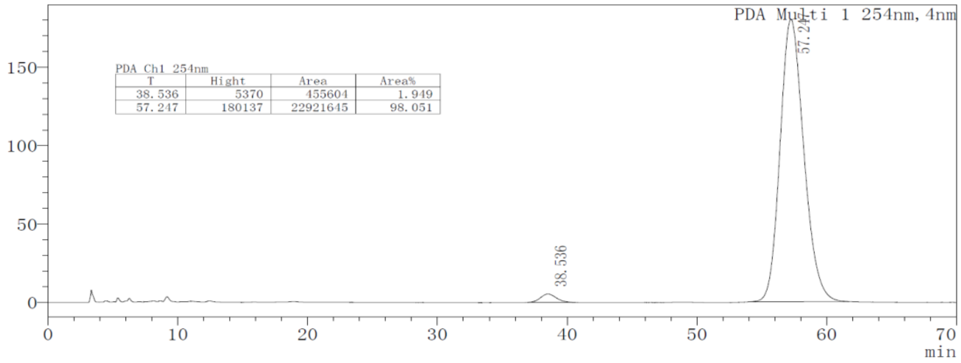

Supplementary Note 7

The Synthesis and Application of Catalyst

Synthesis of the catalyst 12

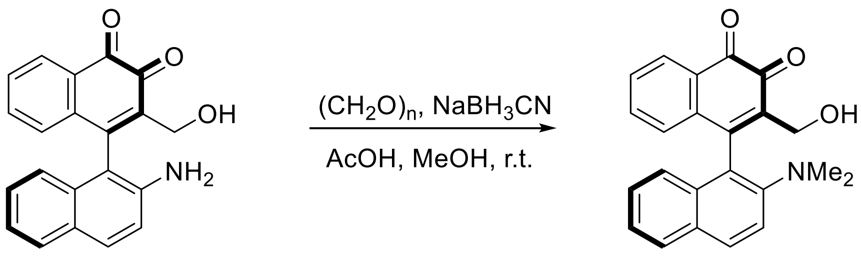

10 **12**

To a stirred solution of compound **10** (223 g, 0.70 mmol) and AcOH (40 µL, 0.70 mmol) in MeOH (10 mL), paraformaldehyde (210 mg, 7 mmol) was added in one portion. After stirred for 0.5 h at room temperature, $NaBH_3CN$ (132 mg, 0.21 mmol) was added slowly, and then the reaction mixture was stirred for 4 h at the same temperature. After additional AcOH (40 µL, 0.70 mmol) was added, the reaction mixture was stirred at room temperature for 0.5 h, and $NaBH_3CN$ (132 mg, 0.21 mmol) was added slowly before the reaction was stirred at room temperature overnight. The reaction mixture was diluted with saturated $NaHCO_3$ solution (30 mL) and extracted with 2*30 mL EA. The combined organic layer was washed with brine, dried over Na_2SO_4 and then stirred under air for about 2 h (the intermediate 1,2-diols was oxidized to the 1,2-naphthoquinone by air). The obtained dark brown organic layer was concentrated under reduced pressure. The crude product was purified by flash column chromatography on silica gel to afford compound **12** (81% yield, 202 mg) as a black solid.

^{1}H NMR (400 MHz, CD_2Cl_2) δ 8.10 (d, *J* = 7.6 Hz, 1H), 7.94 (d, *J* = 8.9 Hz, 1H), 7.81 (d, *J* = 8.1 Hz, 1H), 7.48 (d, *J* = 9.0 Hz, 1H), 7.41-7.24 (m, 5H), 6.64 (d, *J* = 7.8 Hz, 1H), 4.18 (d, *J* = 11.7 Hz, 1H), 3.67 (d, *J* = 11.7 Hz, 1H), 2.65 (s, 6H).

^{13}C NMR (101 MHz, CD_2Cl_2) δ 181.43, 179.57, 152.03, 149.56, 138.11, 137.11, 136.05, 132.24, 131.87, 131.10, 130.84, 130.39, 130.12, 130.04, 128.62, 127.53, 125.29, 125.21, 123.81, 119.43, 58.62, 44.64.

HRMS (ESI) calcd for [M+H] $C_{23}H_{20}NO_3$, m/z: 358.1443, found: 358.1435.

HPLC analysis: CHIRALCEL OD3, hexane/isopropanol = 90/10, 1.0 mL/min, λ = 254 nm, t_R (major) = 21.7 min, t_R (minor) = 25.2 min, ee = 99%.

Supplementary Figure 131. Chiral HPLC spectrum of racemic **12**

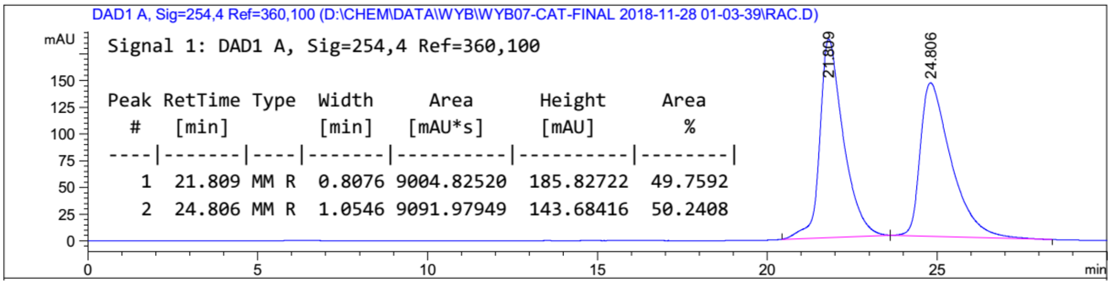

Supplementary Figure 132. Chiral HPLC spectrum of **(*R*)-12**

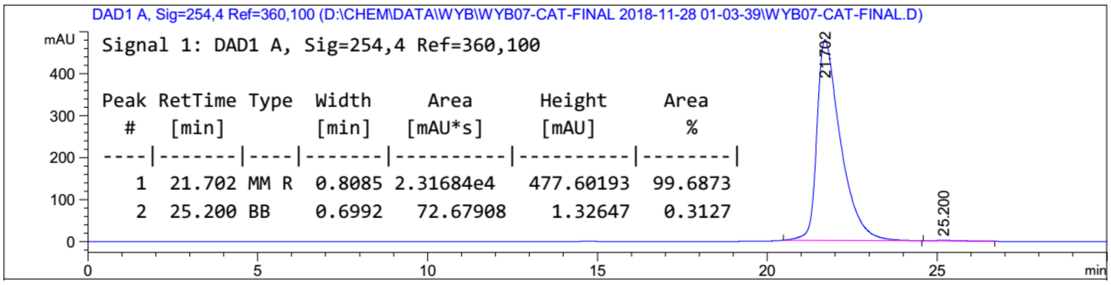

Synthesis of the starting material amine 13

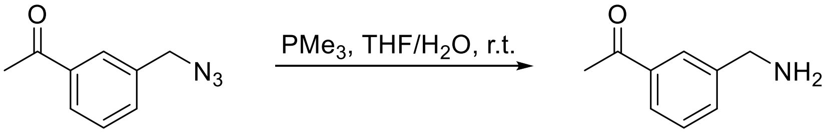

13

To a stirred solution of 1-(3-(azidomethyl)phenyl)ethanone (1.75 g, 10.0 mmol) in THF/H_2O solvent mixture (40 mL, 1:1), PMe_3 (50 mL, 1.0 M in THF) was added slowly at 0 ℃. After stirred at room temperature overnight, the reaction mixture was diluted with water and extracted with 3*60 mL EA. The combined organic layer was extracted with 2*20 mL HCl solution (1 M). The combined aqueous layer was washed with 30 mL EA. After being alkalized with NaOH to pH=12, the aqueous layer was extracted with 2*30 mL EA. The organic layers were combined and washed with brine, dried over Na_2SO_4 and concentrated under reduced pressure to afford compound **13** (1.02 g, 68% yield) as yellowish liquid.

^{1}H NMR (400 MHz, $CDCl_3$) δ 7.92 (s, 1H), 7.84 (d, *J* = 7.7 Hz, 1H), 7.54 (d, *J* = 7.6 Hz, 1H), 7.44 (t, *J* = 7.6 Hz, 1H), 3.95 (s, 2H), 2.61 (d, *J* = 3.9 Hz, 3H), 1.64 (brs, 2H).

^{13}C NMR (101 MHz, $CDCl_3$) δ 198.39, 143.82, 137.51, 132.06, 128.90, 127.06, 126.94, 46.27, 26.84.

Application of catalyst 12

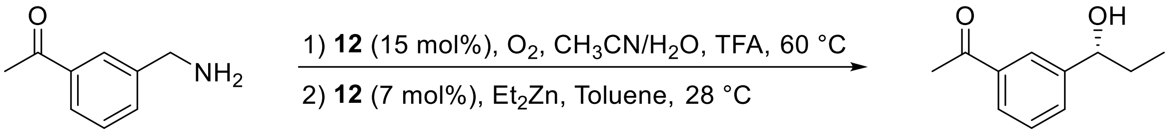

To a stirred solution of compound **13** (14.9 mg, 0.1 mmol) in CH_3CN/H_2O (3.0 mL/0.05 mL), **Cat. 12** (5.4 mg, 0.015 mmol) and TFA (2.2 μL, 0.030 mmol) was added. The flask was evacuated and backfilled with O_2 from an inlet three times. After stirred with a balloon of O_2 gas on top at 60 ℃ for 16 h, the reaction mixture was concentrated under reduced pressure and purified by flash column chromatography on Al_2O_3. A Schlenk tube charged with **Cat. 12** (2.5 mg, 0.007 mmol) was evacuated and filled with argon for three times. Then anhydrous toluene (2.0 mL) and BH_3 (1 M in THF, 14 μL) was added. After stirred for 5 min, the above obtained product and Et_2Zn (1.0 M in Hexane, 0.21 mL) was added to the solution. The reaction was stirred at 28 ℃ for 7 days. The reaction mixture was quenched with EA/H_2O, and the organic layer was separated, dried over Na_2SO_4 and purified by prepared TLC on silica gel to afford the product **14** as colourless oil (9.0 mg, 51% yield, 41% ee).

^{1}H NMR (400 MHz, $CDCl_3$) δ 7.94 (s, 1H), 7.87 (d, *J* = 7.7 Hz, 1H), 7.57 (d, *J* = 7.7 Hz, 1H), 7.45 (t, *J* = 7.7 Hz, 1H), 4.69 (t, *J* = 6.5 Hz, 1H), 2.62 (s, 3H), 2.00 (s, 1H), 1.90-1.70 (m, 2H), 0.93 (t, *J* = 7.4 Hz, 3H).

^{13}C NMR (101 MHz, $CDCl_3$) δ 198.39, 145.31, 137.37, 130.87, 128.83, 127.67, 125.89, 75.67, 32.19, 26.87, 10.16.

HPLC analysis: CHIRALCEL OD3, hexane/isopropanol = 95/5, 0.5 mL/min, λ = 254 nm, t_R (major) = 31.6 min, t_R (minor) = 28.3 min, ee = 41%.

Supplementary Figure 133. Chiral HPLC spectrum of racemic **14**

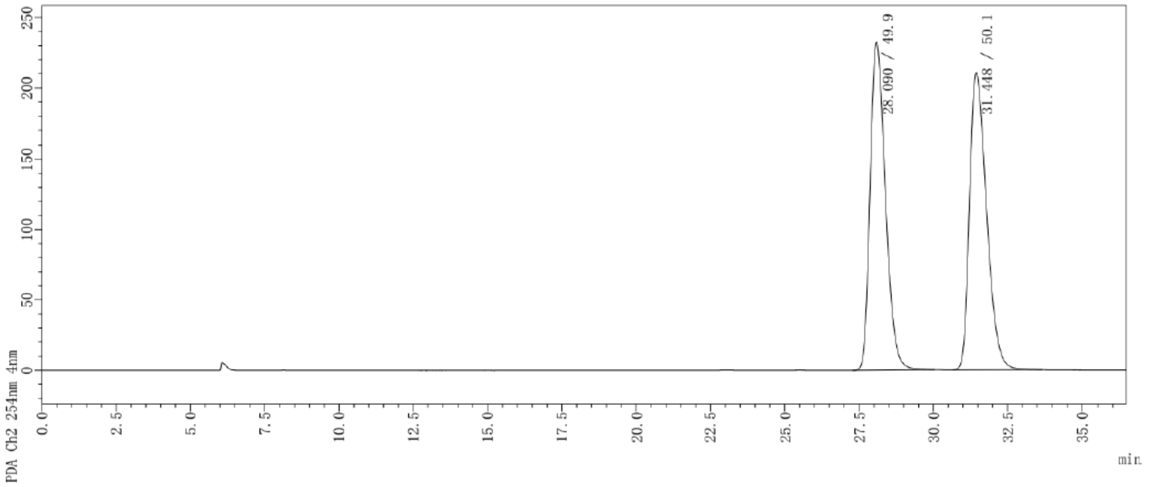

Supplementary Figure 134. Chiral HPLC spectrum of optically active **14**

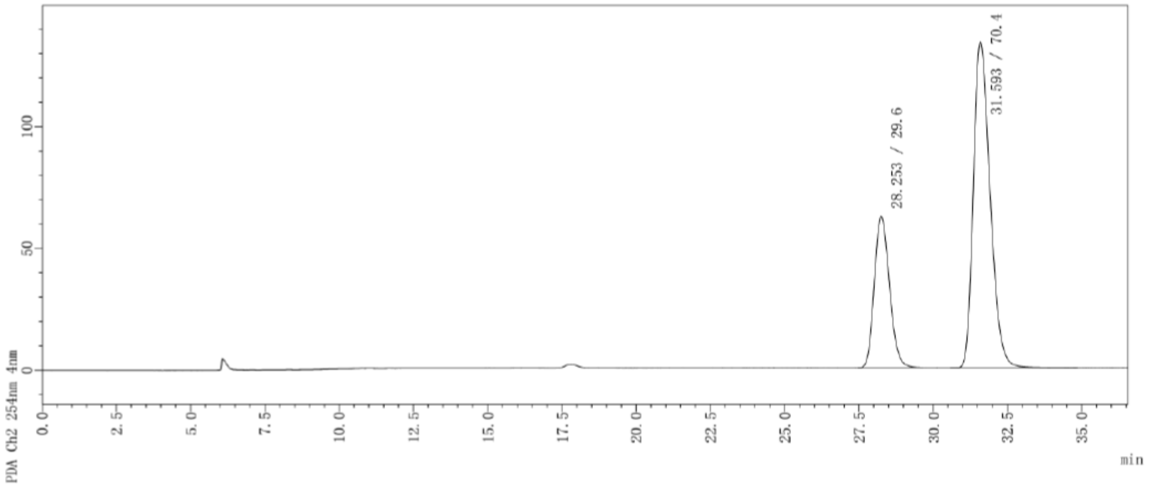

Supplementary Figure 135. Reactions of **1a** and **2c** catalyzed by (*R*)-**CPA7** and (*R*)-**CPA7-Li**.

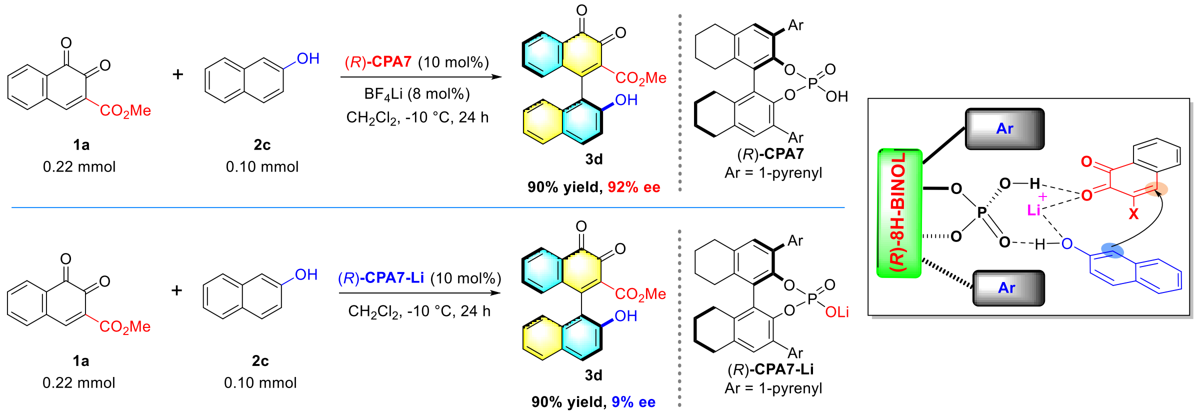

A Lewis acid-assisted catalytic mode may be operating, in which the small lithium ion coordinates to the oxygen atoms both on the quinone and on the naphthol, further enhancing the stereocontrol. However, it is difficult to identify the exact role of BF_4Li with limited experimental results, considering that it only slightly improved the ee from 88% to 92%.

Supplementary Figure 136. Substrates involved in the manuscript.

o-Naphthoquinone derivatives

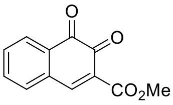

1a-1

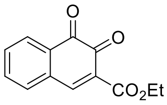

1a-2

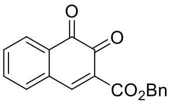

1a-3

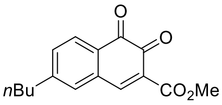

1a-4

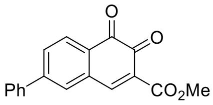

1a-5

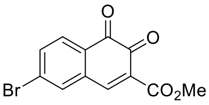

1a-6

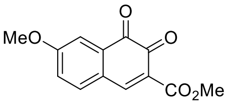

1a-7

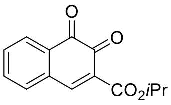

1a-8

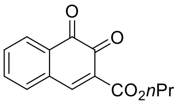

1a-9

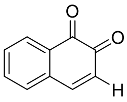

1b-1

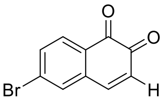

1b-2

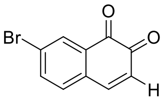

1b-3

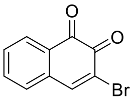

1c-1

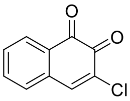

1c-2

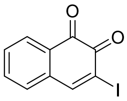

1c-3

Substituted 2-naphthamines

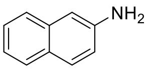

2a-1

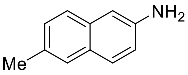

2a-2

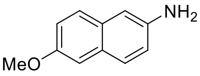

2a-3

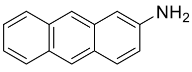

2a-4

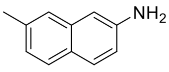

2a-5

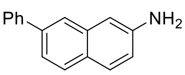

2a-6

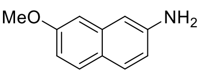

2a-7

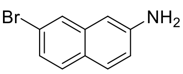

2a-8

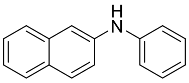

2b-1

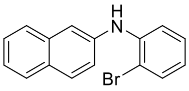

2b-2

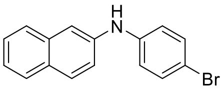

2b-3

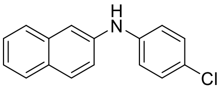

2b-4

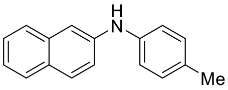

2b-5

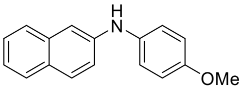

2b-6

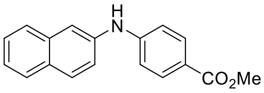

2b-7

Substituted 2-naphthols

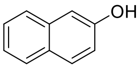

2c-1

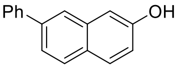

2c-2

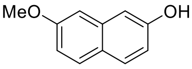

2c-3

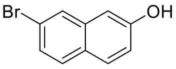

2c-4

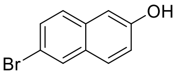

2c-5

Substituted indoles

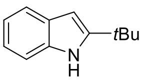

2d-1

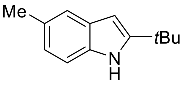

2d-2

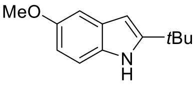

2d-3

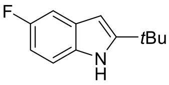

2d-4

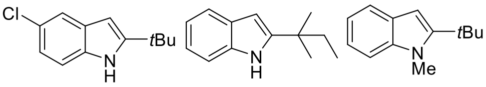

2d-5 2d-6 2d-7

Supplementary Figure 137. X-ray crystal structure of **9**.

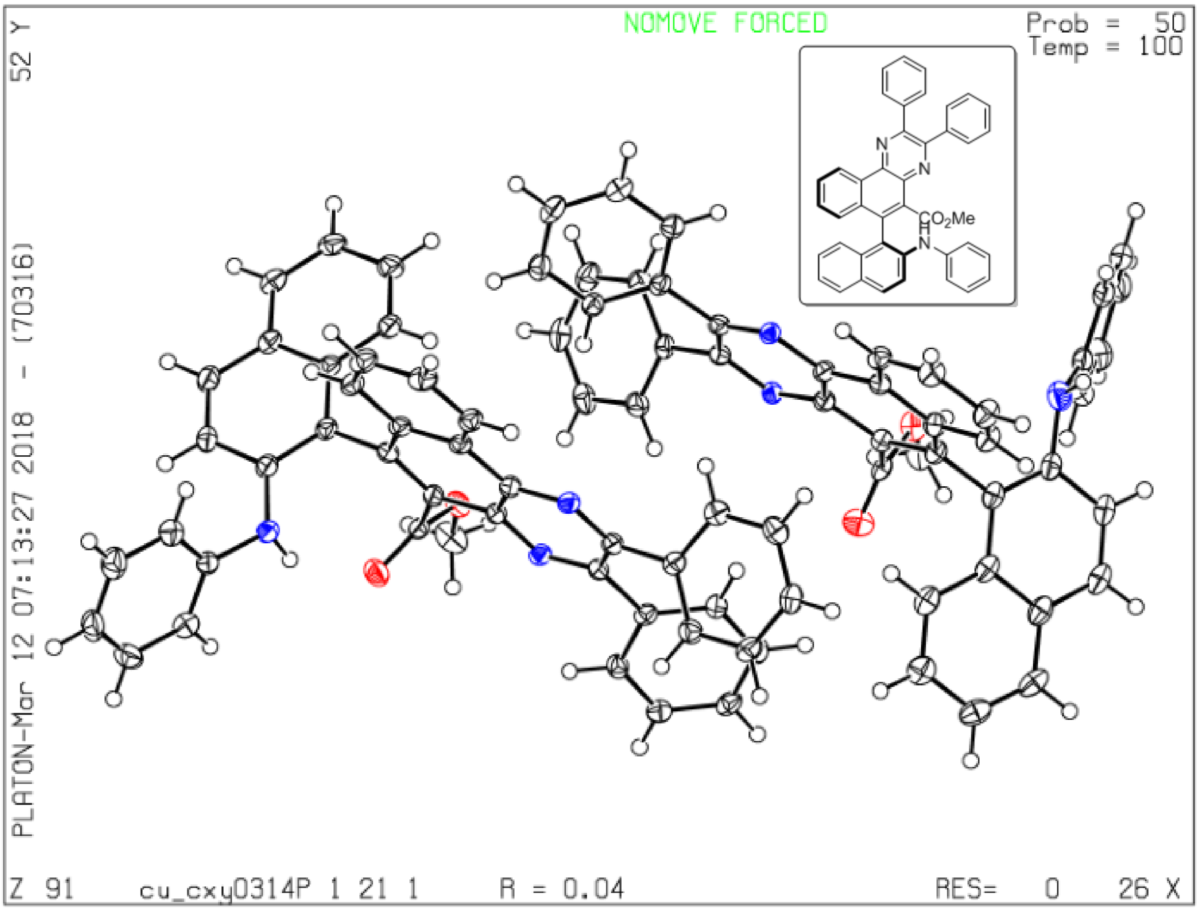

Supplementary Figure 138. ^{1}H NMR of **1a-1**.

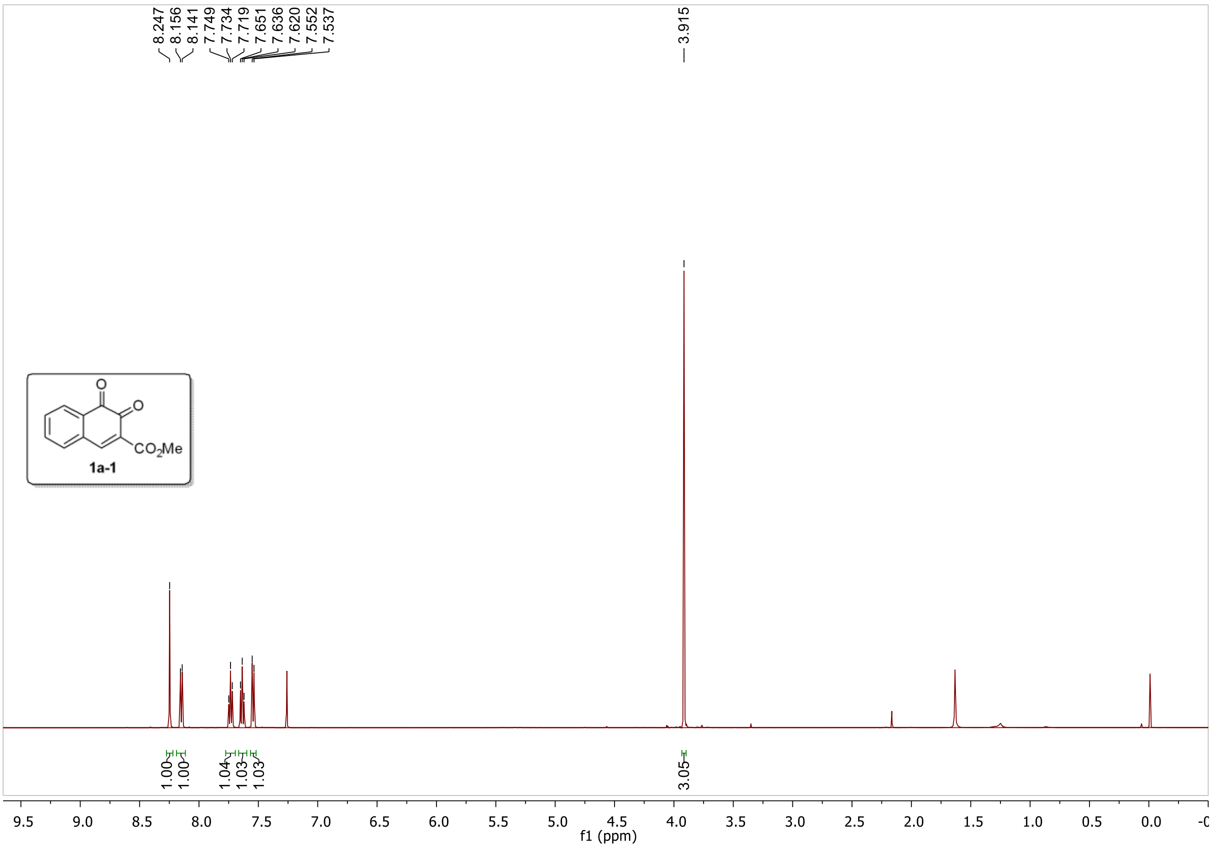

Supplementary Figure 139. ^{13}C NMR of **1a-1**.

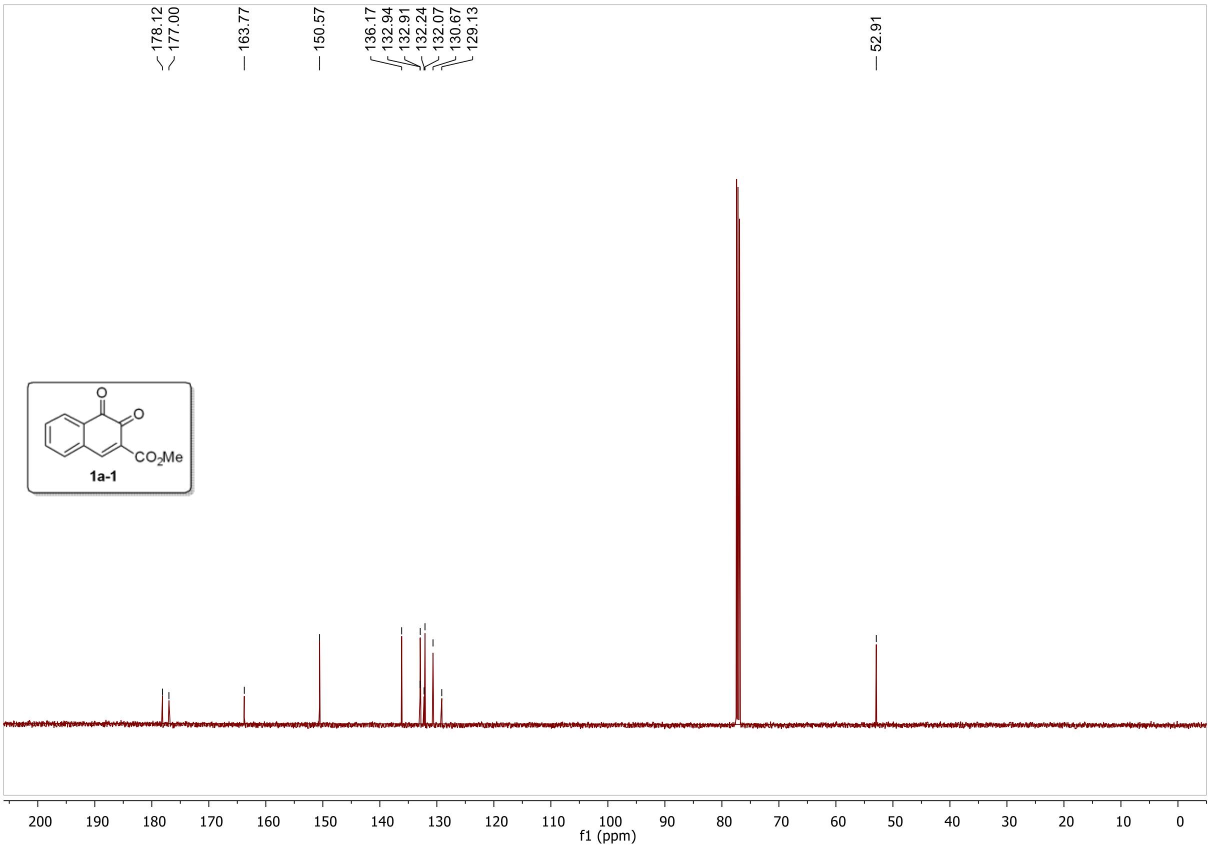

Supplementary Figure 140. ¹H NMR of **1a-2**.

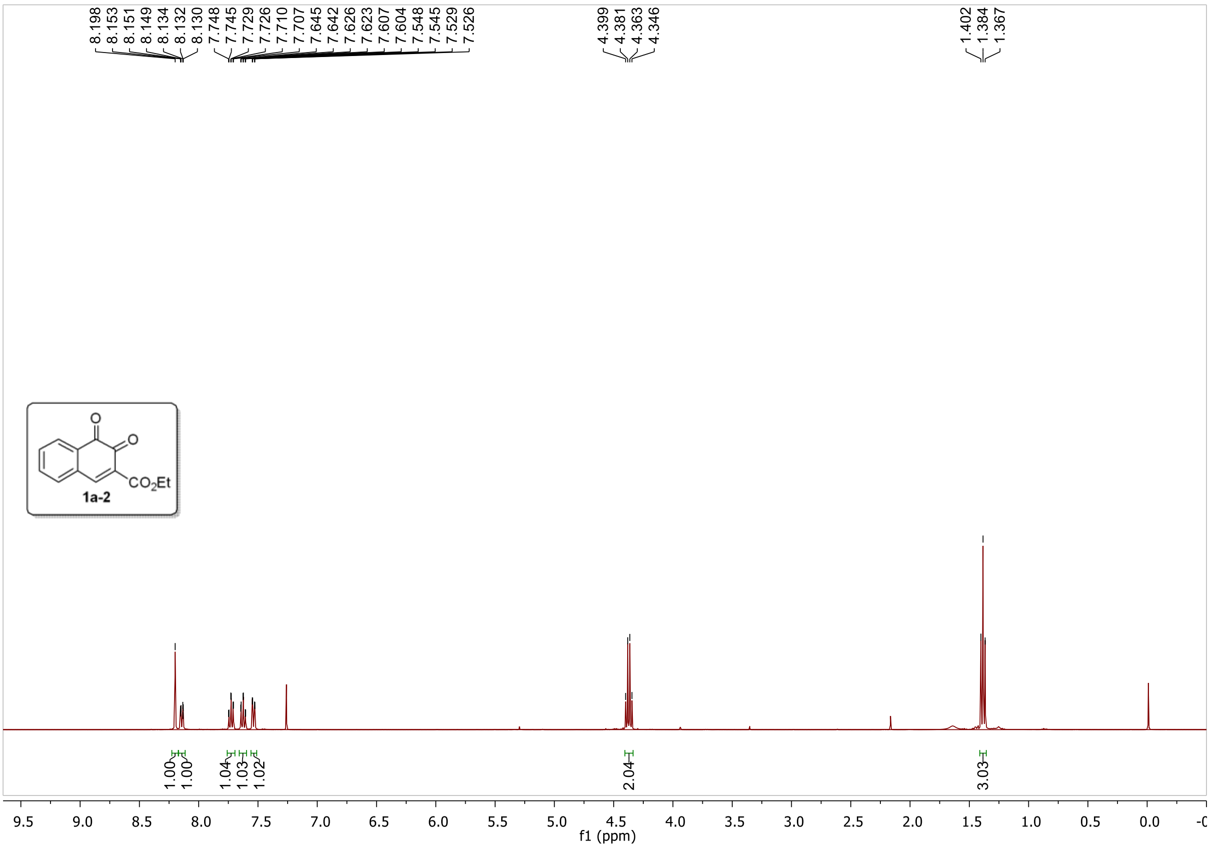

Supplementary Figure 141. ¹³C NMR of **1a-2**.

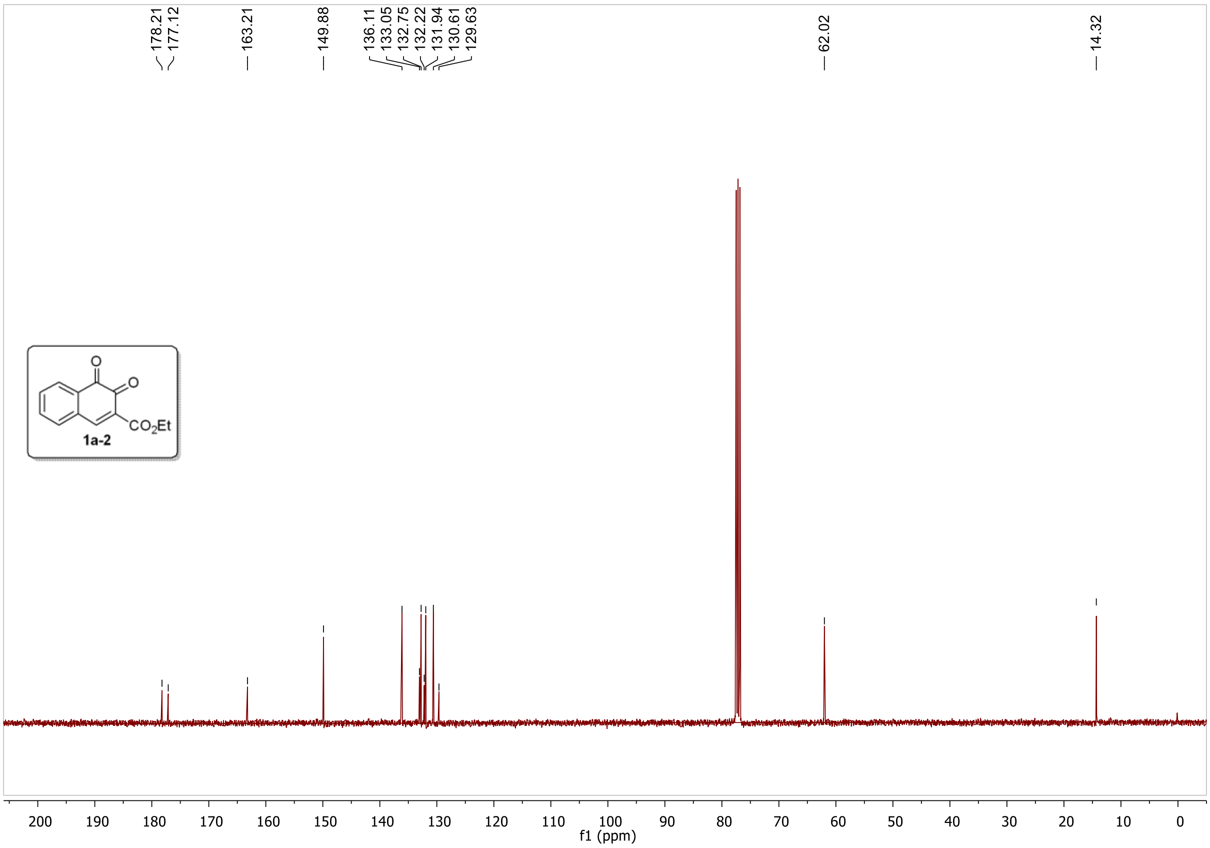

Supplementary Figure 142. ^{1}H NMR of **1a-3**.

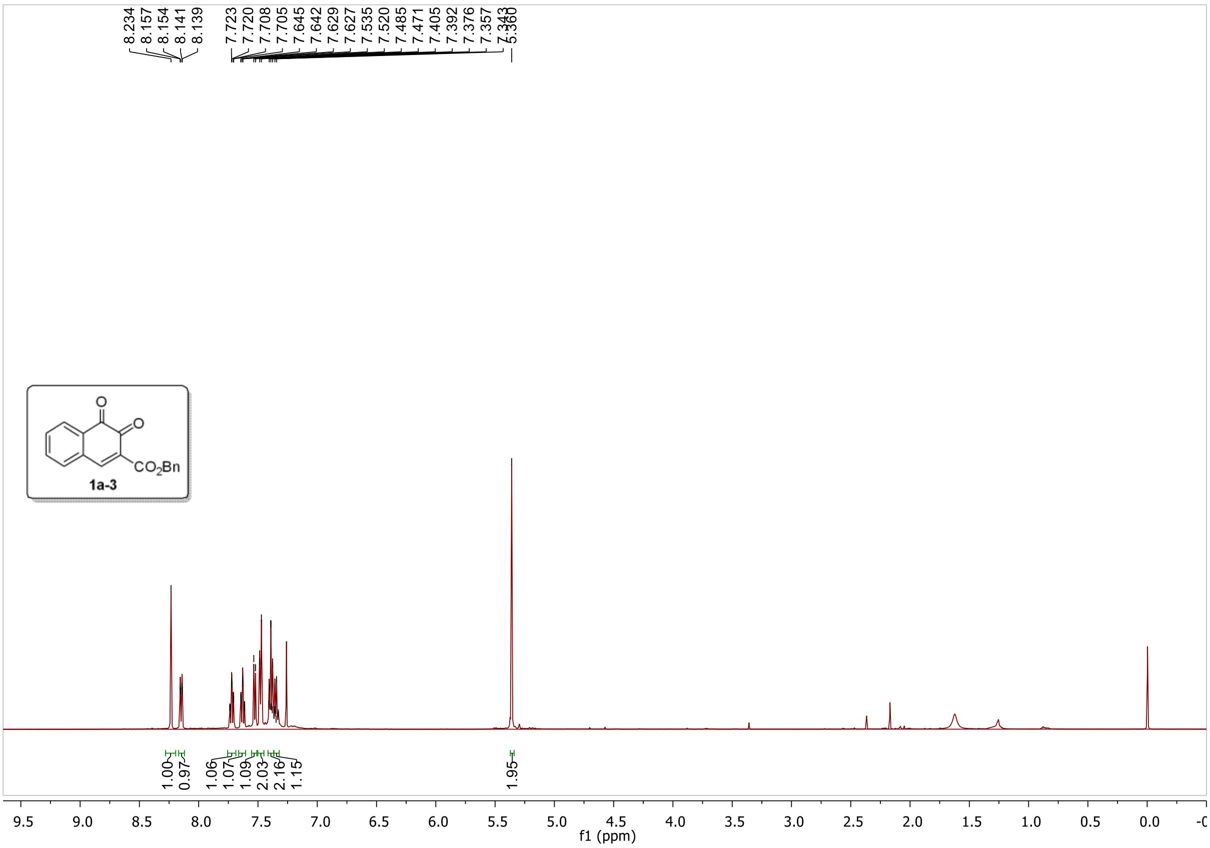

Supplementary Figure 143. ^{13}C NMR of **1a-3**.

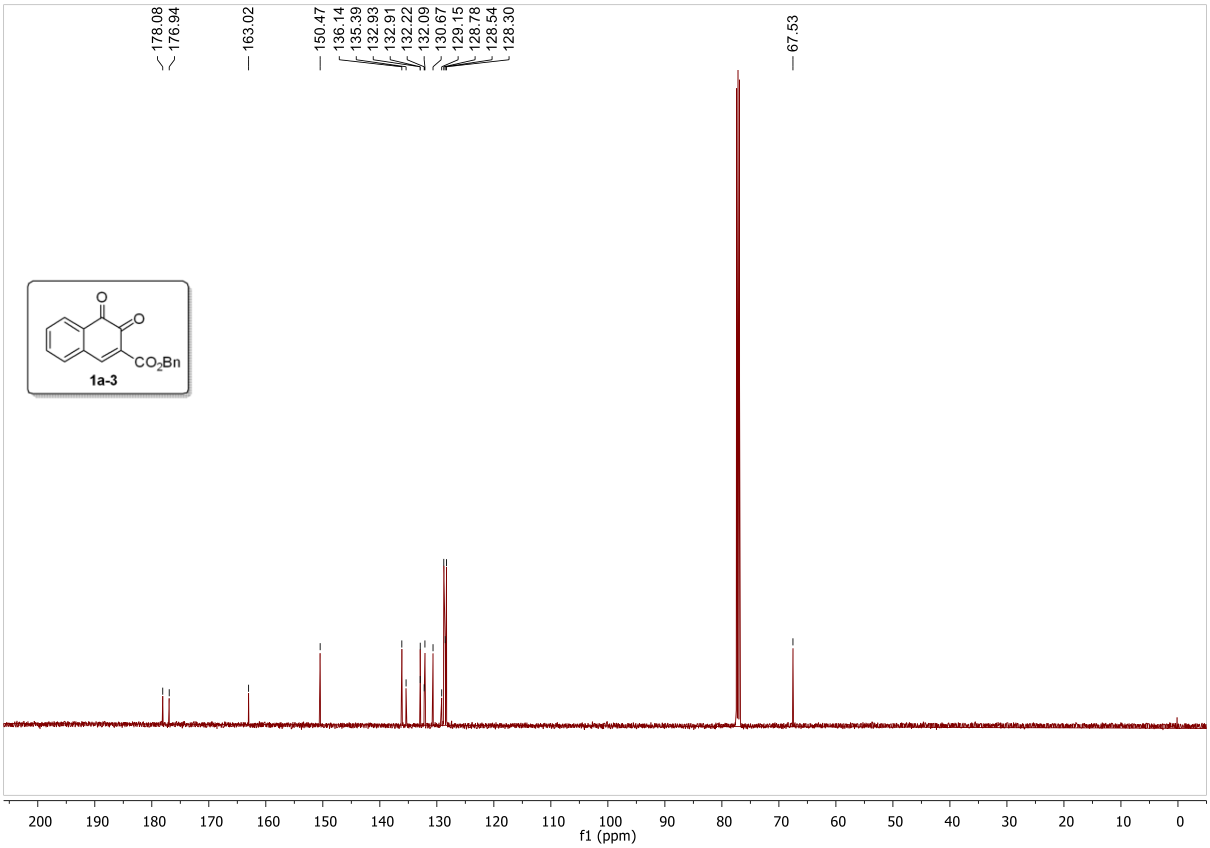

Supplementary Figure 144. ^{1}H NMR of **1a-4**.

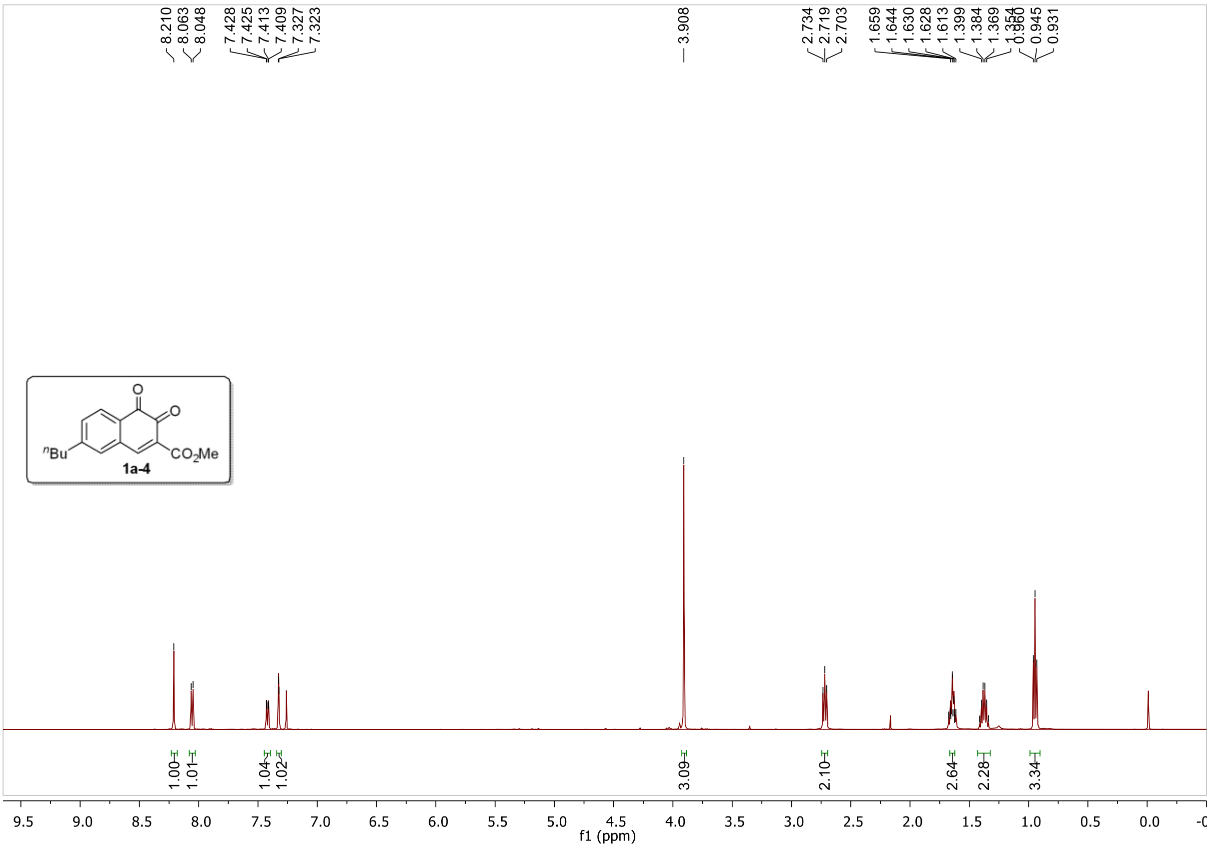

Supplementary Figure 145. ^{13}C NMR of **1a-4**.

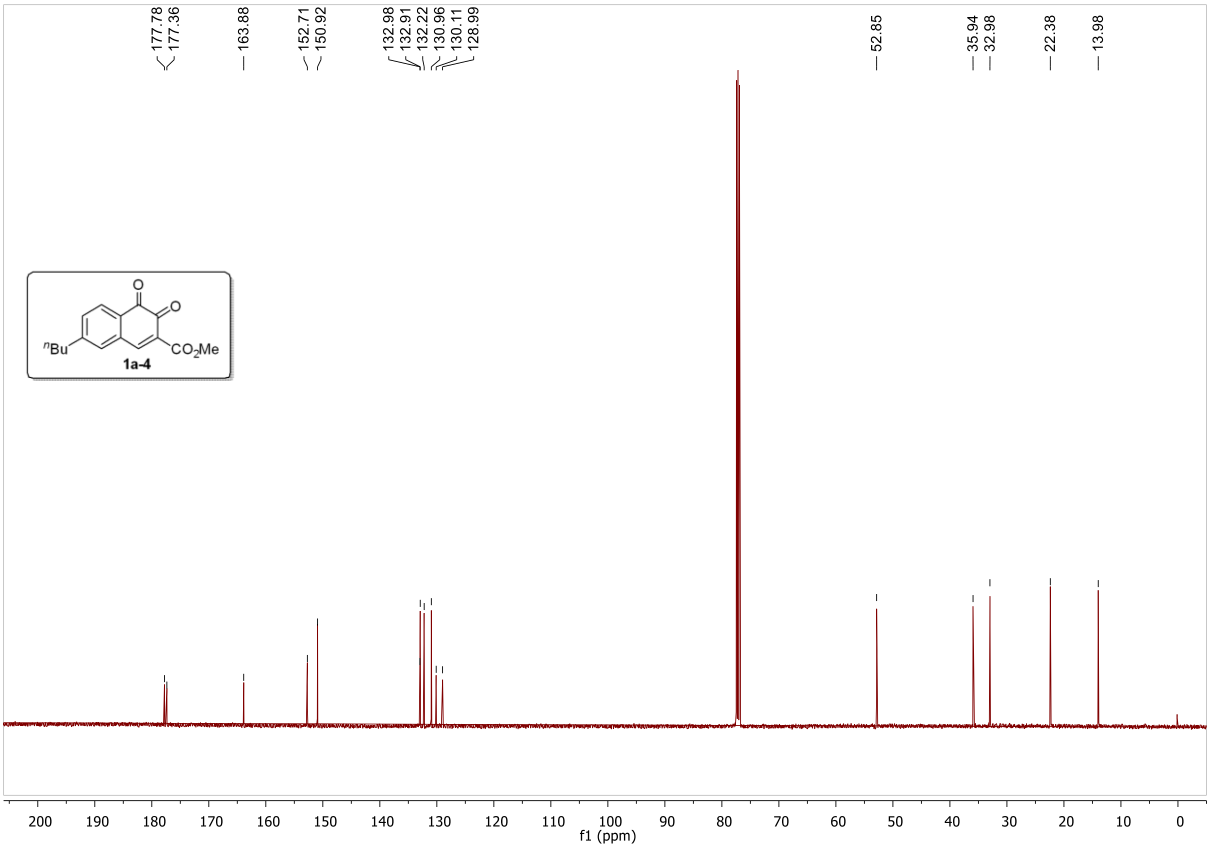

Supplementary Figure 146. ^{1}H NMR of **1a-5**.

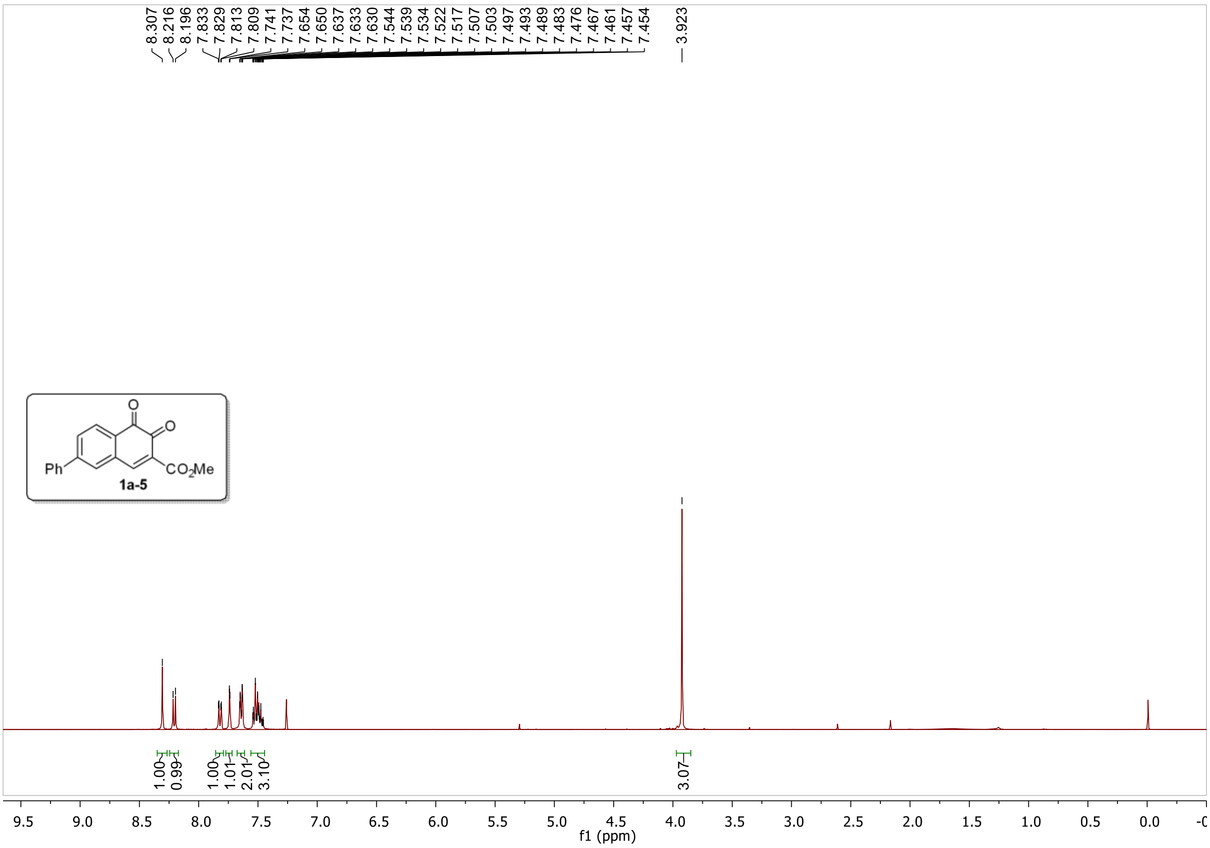

Supplementary Figure 147. ^{13}C NMR of **1a-5**.

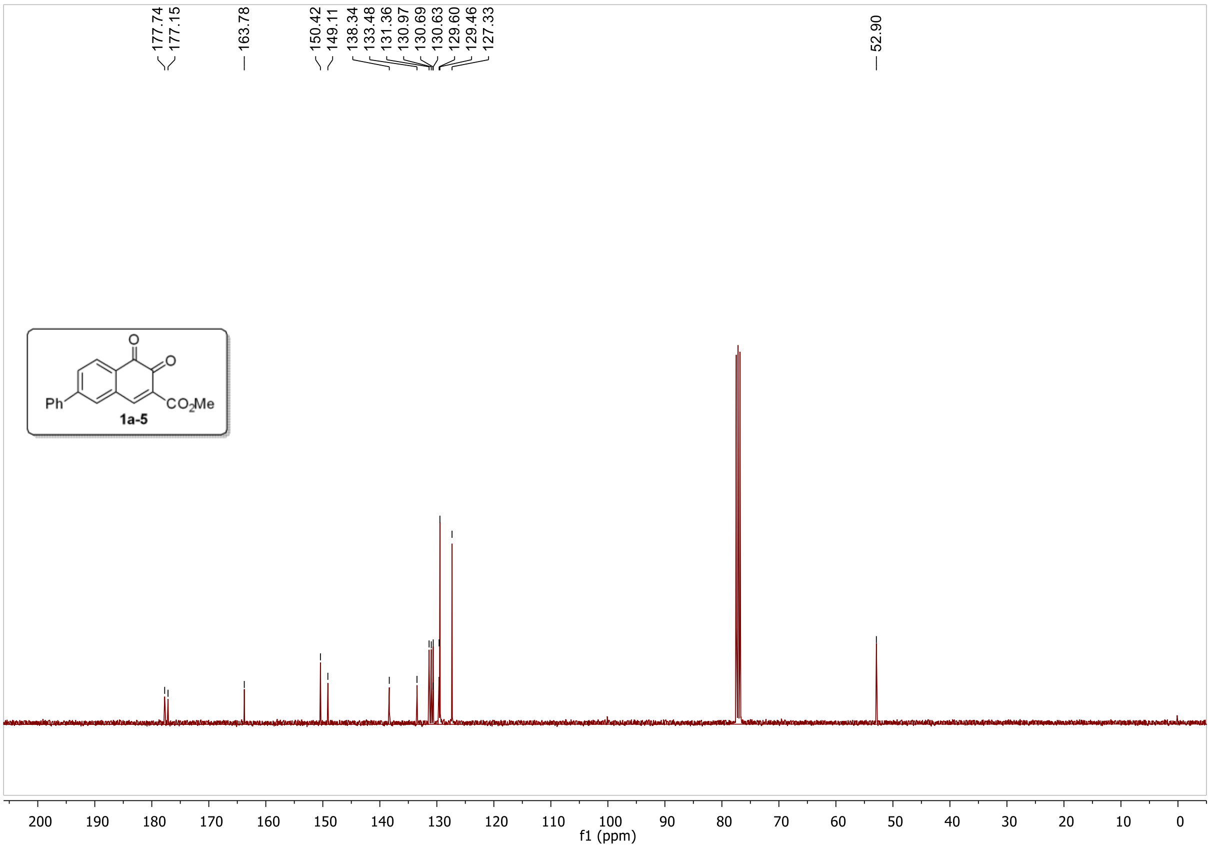

Supplementary Figure 148. ^{1}H NMR of **1a-6**.

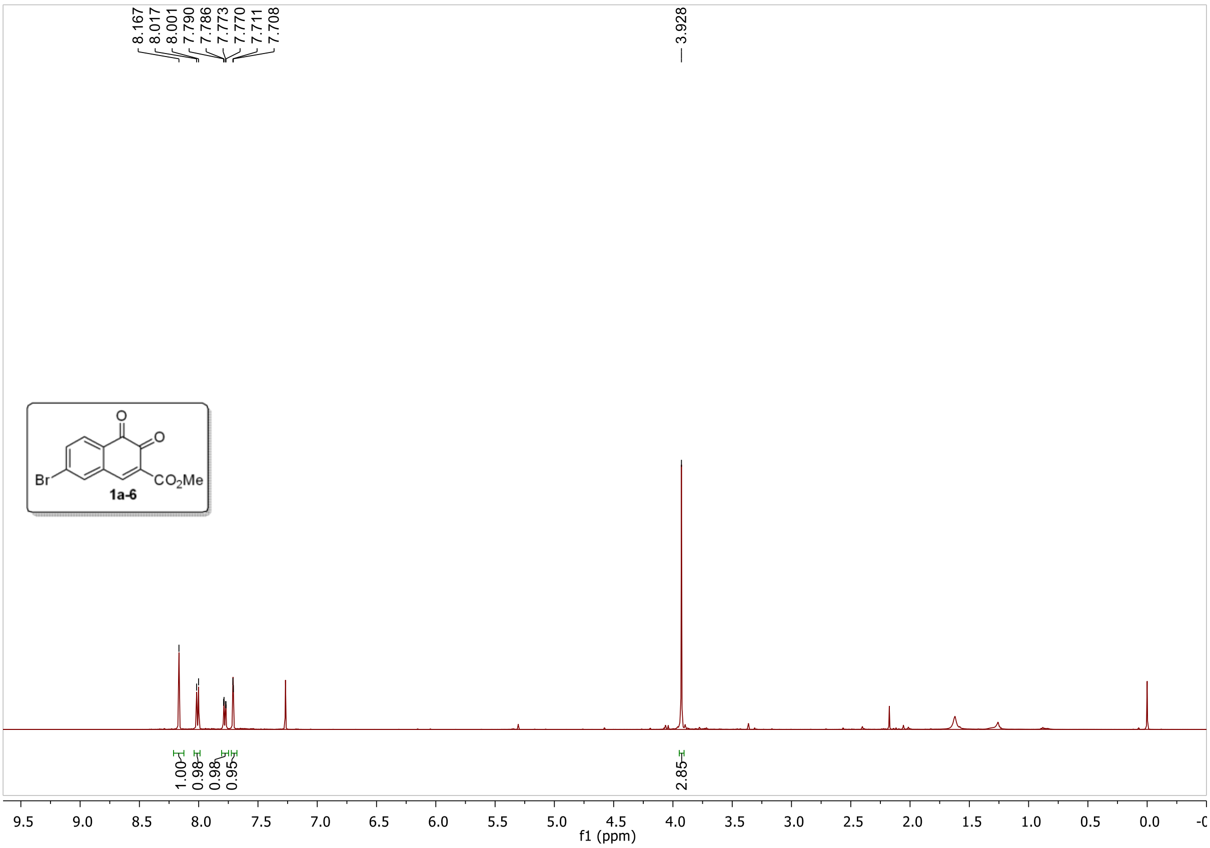

Supplementary Figure 149. ^{13}C NMR of **1a-6**.

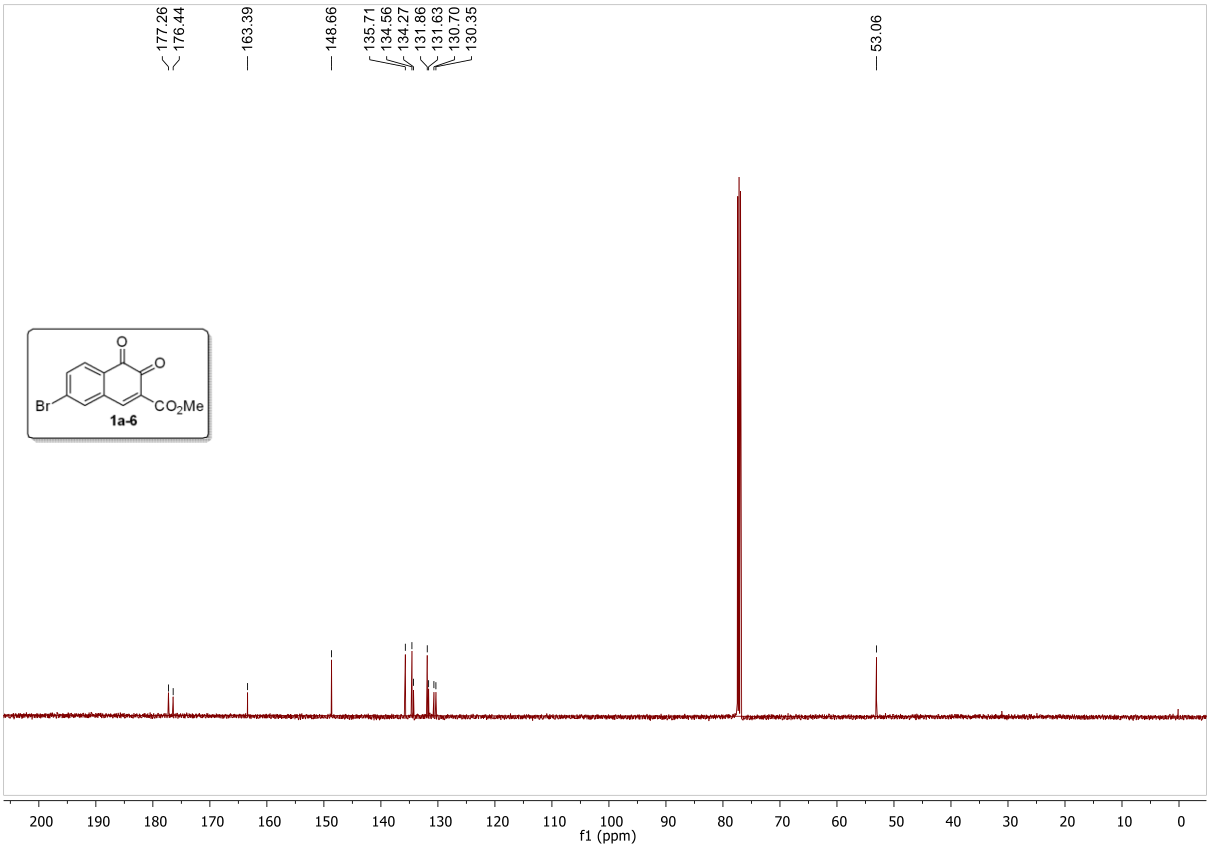

Supplementary Figure 150. ^{1}H NMR of **1a-7**.

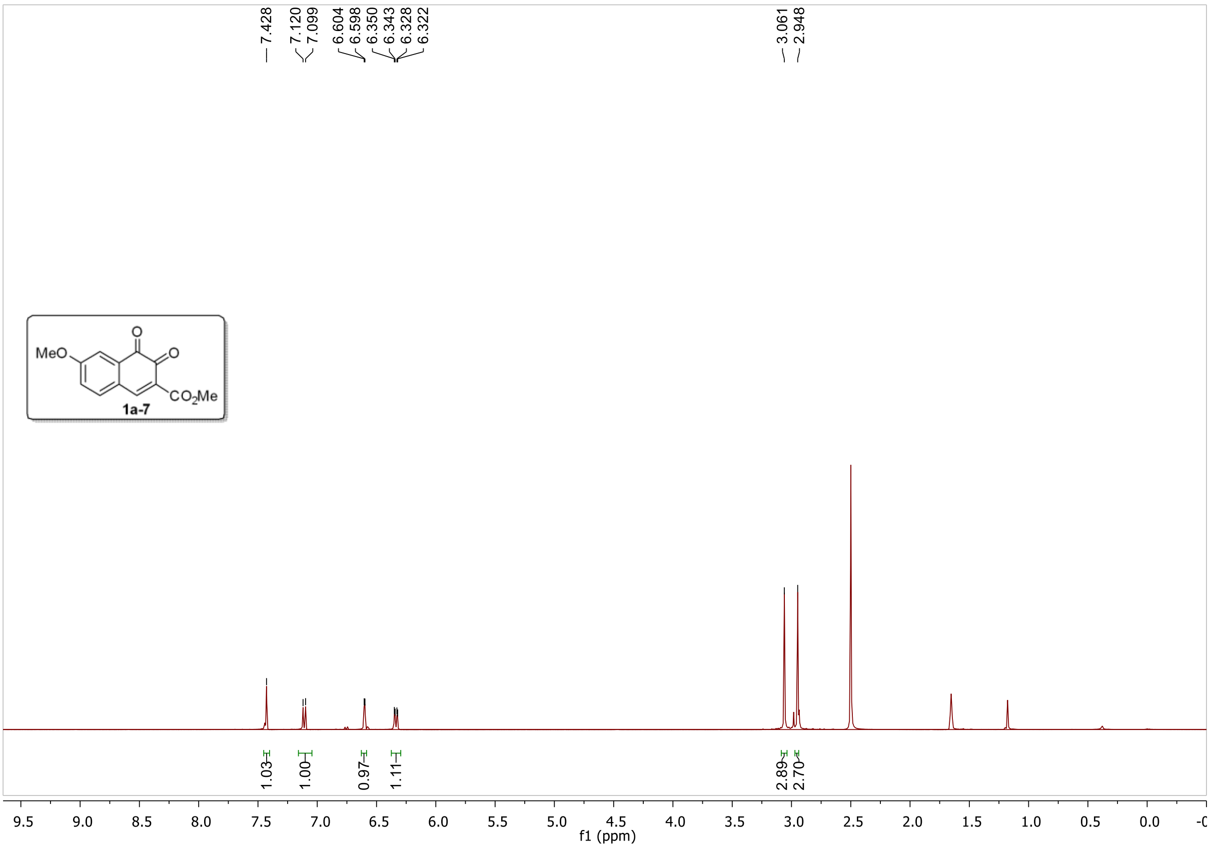

Supplementary Figure 151. ^{13}C NMR of **1a-7**.

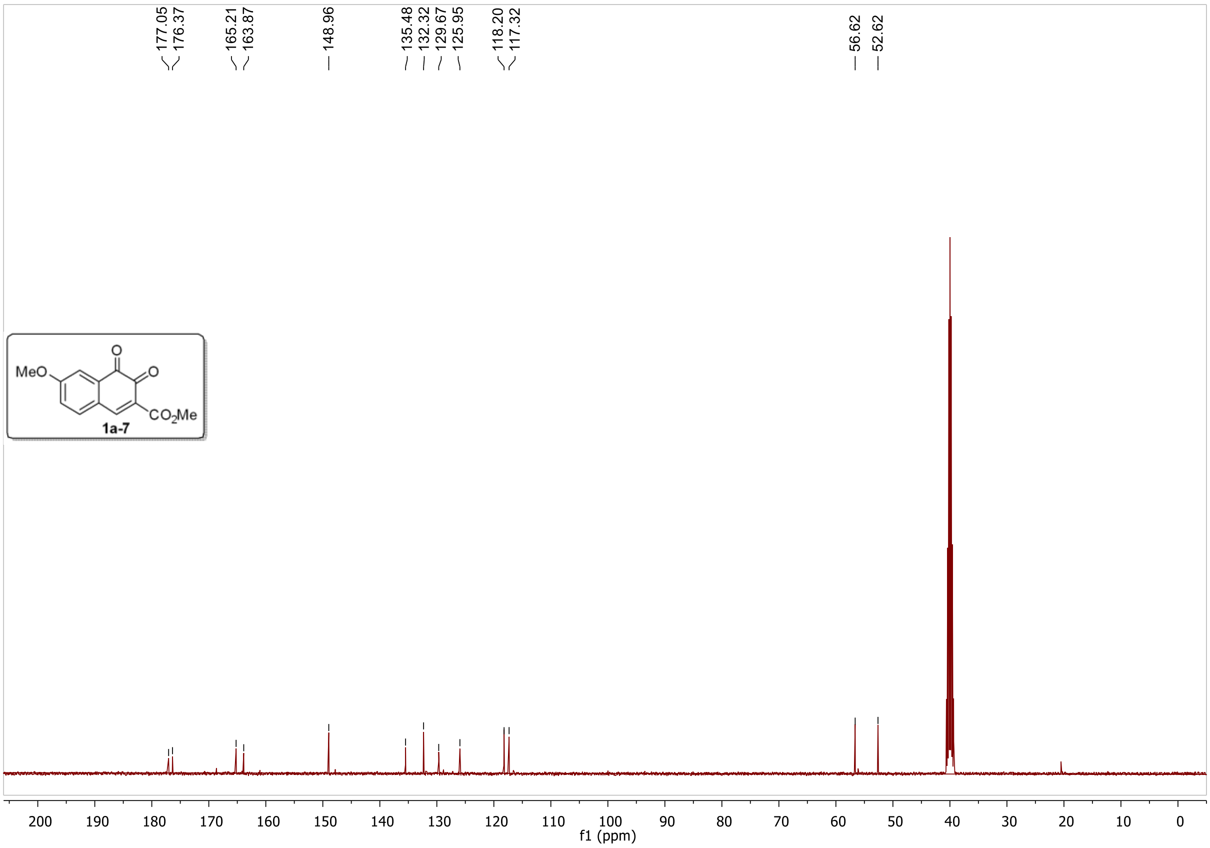

Supplementary Figure 152. ^{1}H NMR of **1a-8**.

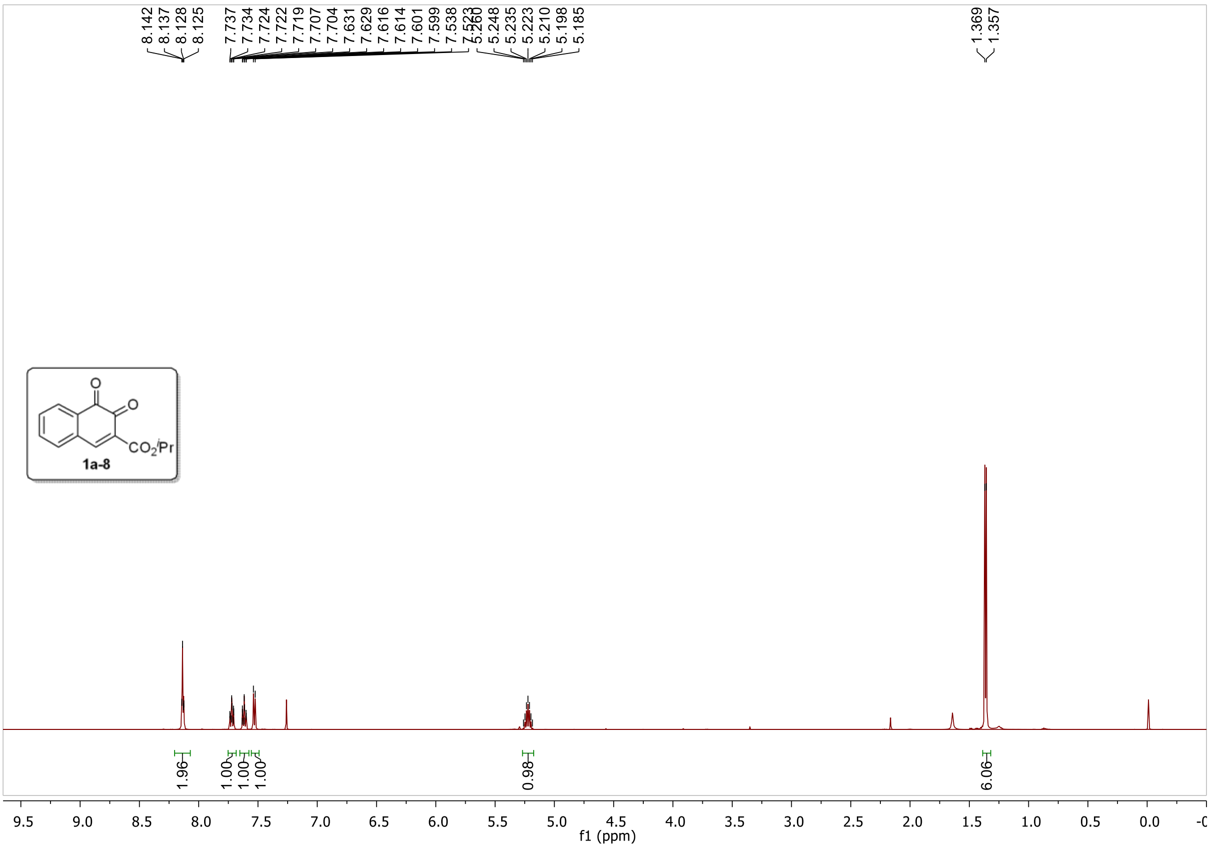

Supplementary Figure 153. ^{13}C NMR of **1a-8**.

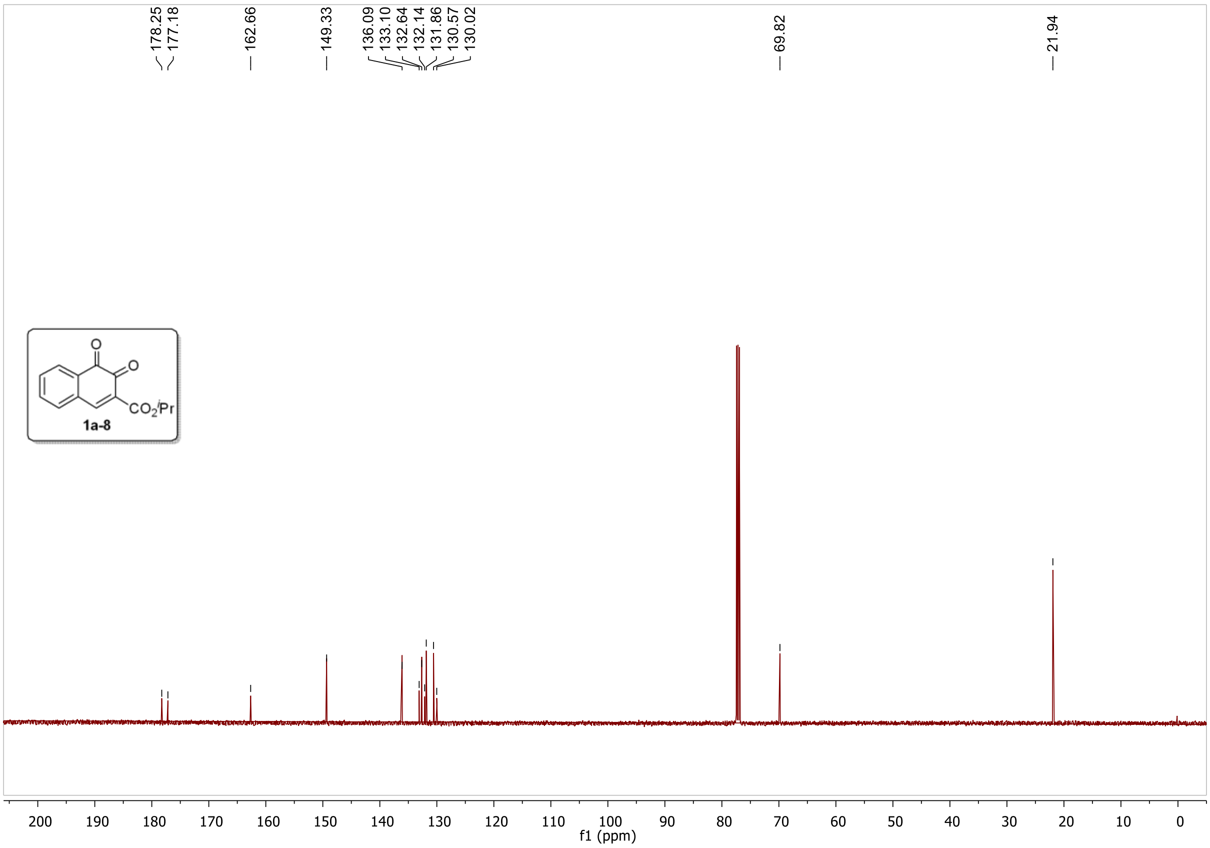

Supplementary Figure 154. ^{1}H NMR of **1a-9**.

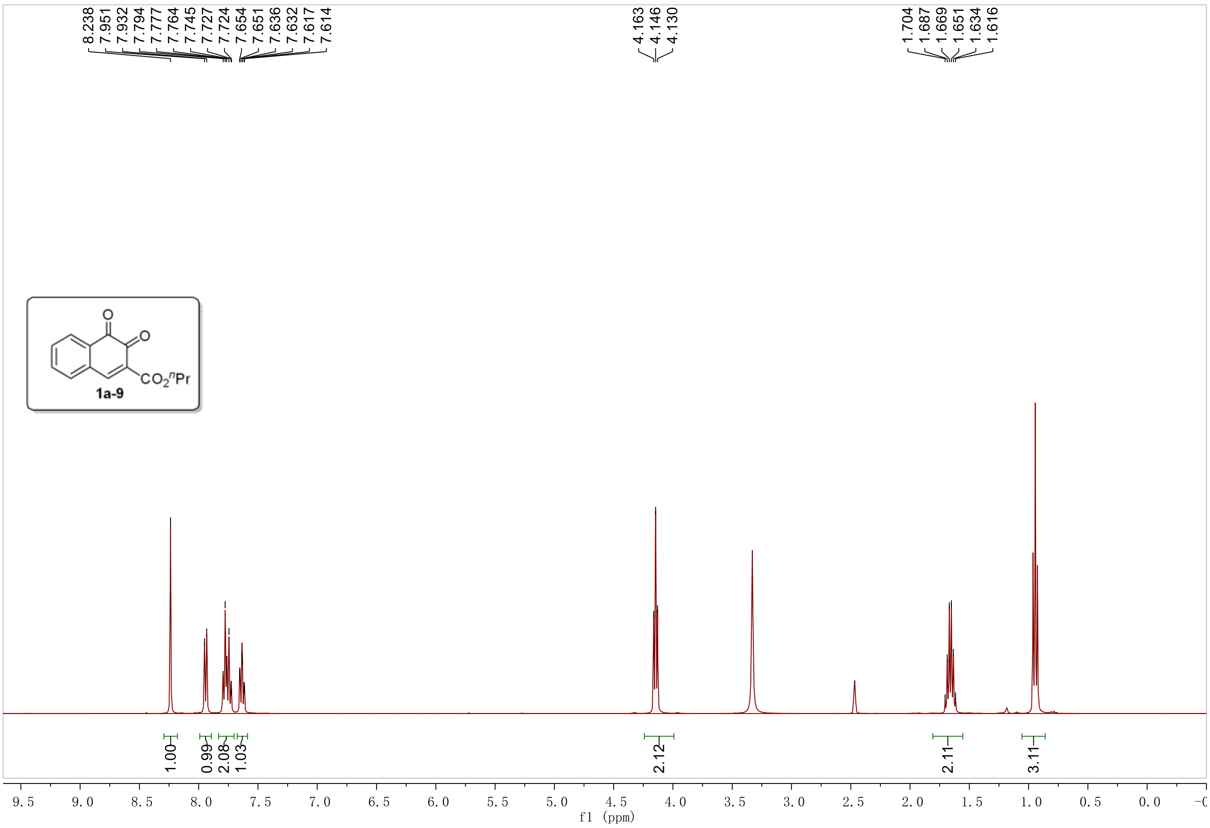

Supplementary Figure 155. ^{13}C NMR of **1a-9**.

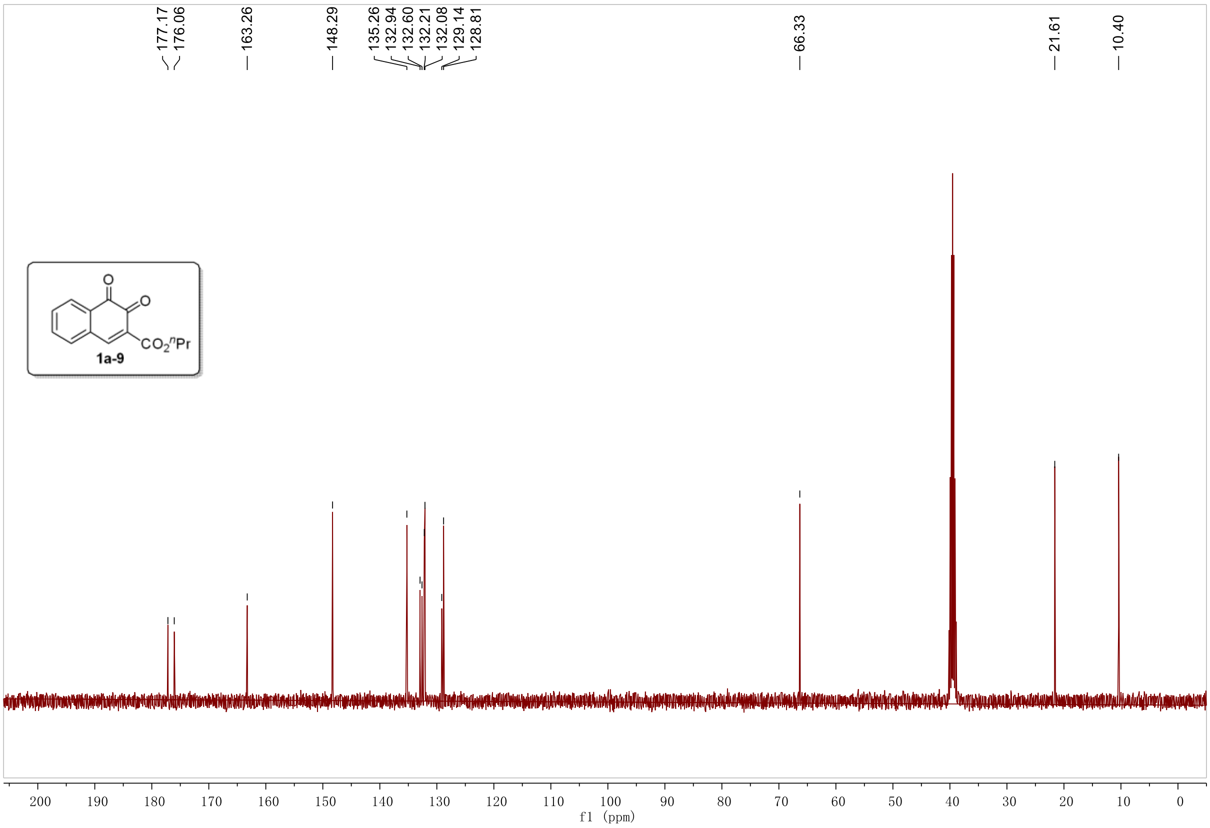

Supplementary Figure 156. ^{1}H NMR of **1c-1**.

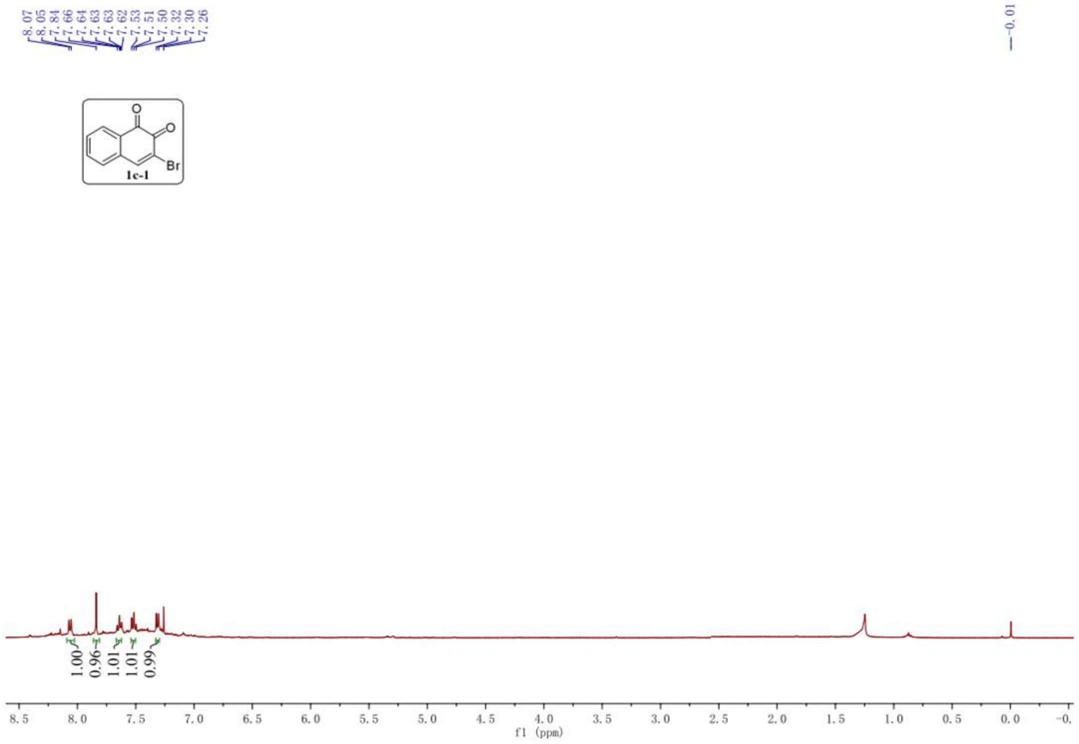

Supplementary Figure 157. ^{13}C NMR of **1c-1**.

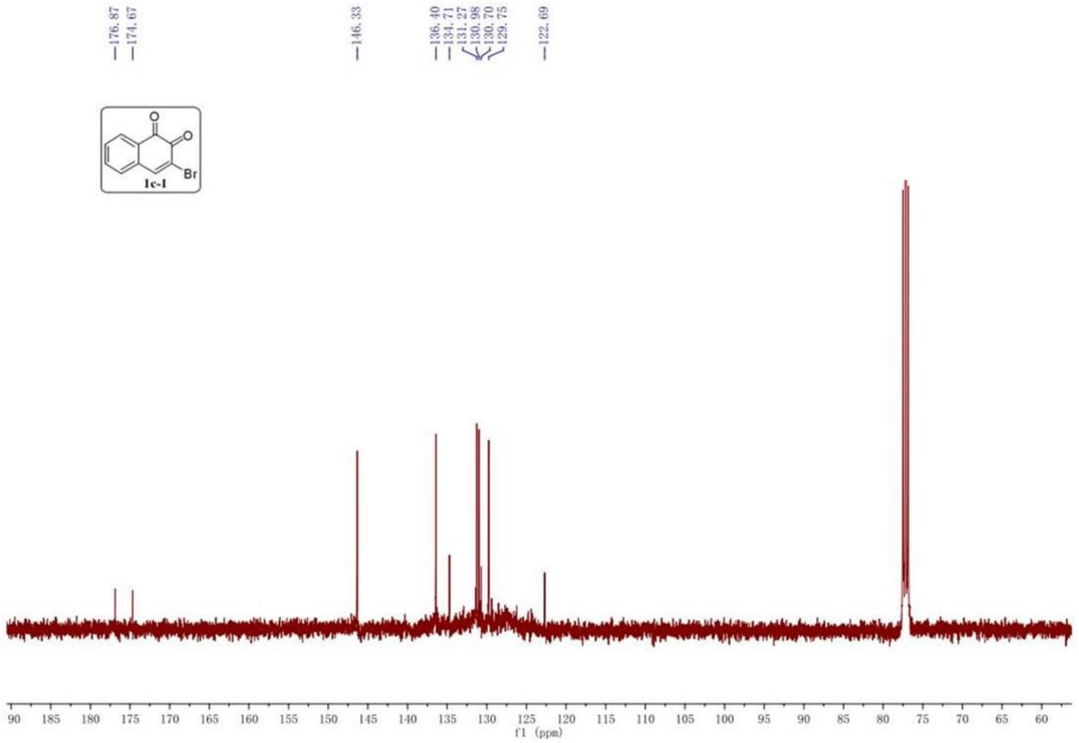

Supplementary Figure 158. ^{1}H NMR of **1c-2**.

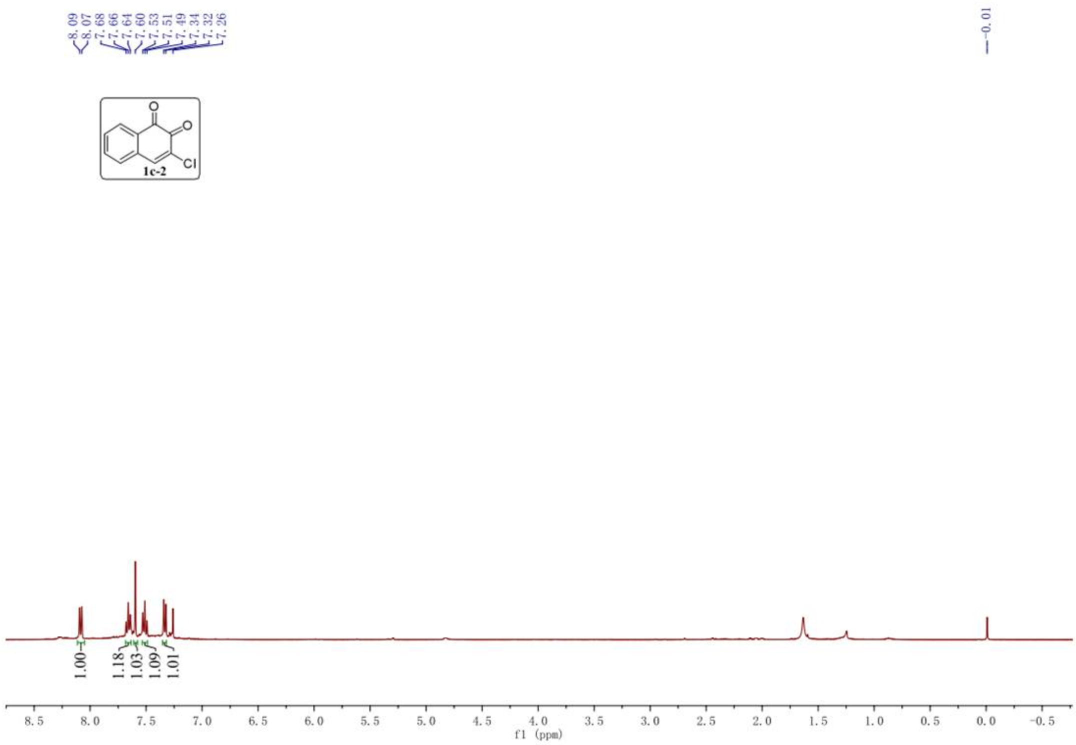

Supplementary Figure 159. ^{13}C NMR of **1c-2**.

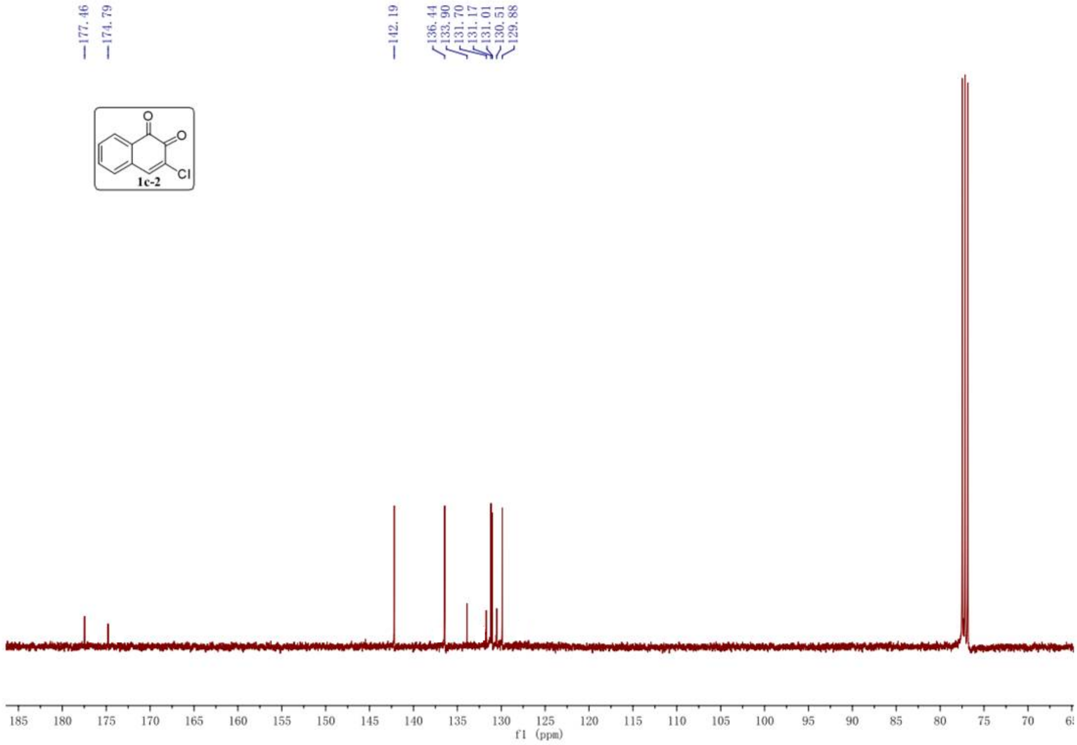

Supplementary Figure 160. ^{1}H NMR of **1c-3**.

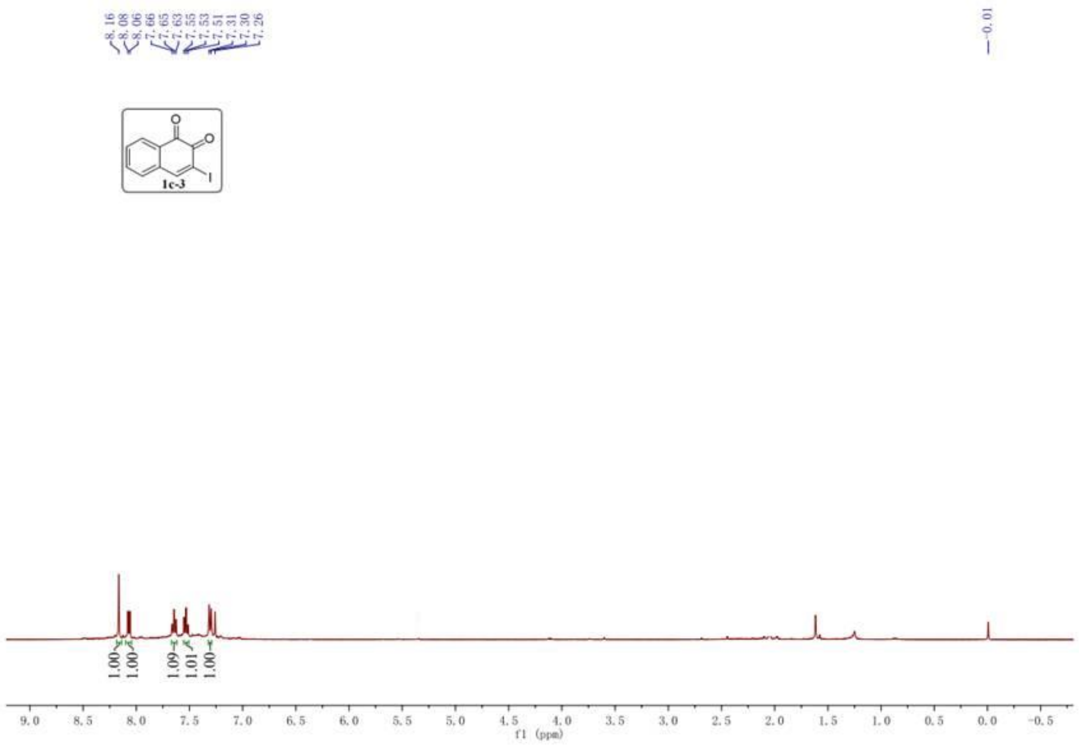

Supplementary Figure 161. ^{13}C NMR of **1c-3**.

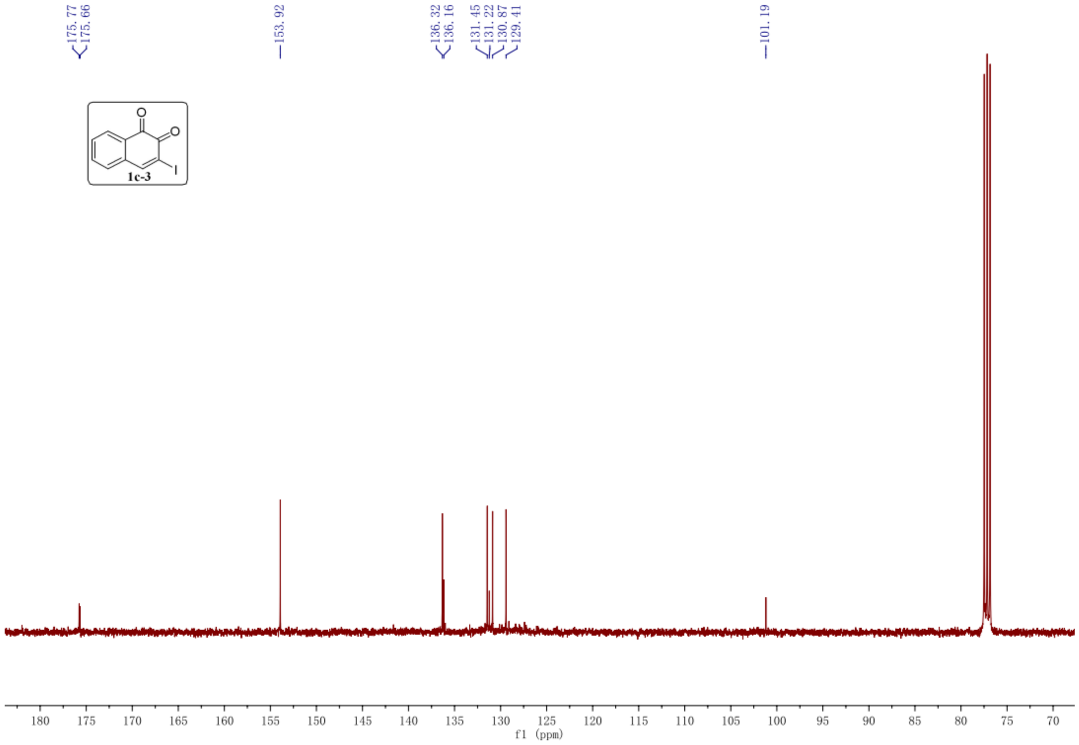

Supplementary Figure 162. ^{1}H NMR of **2c-2**.

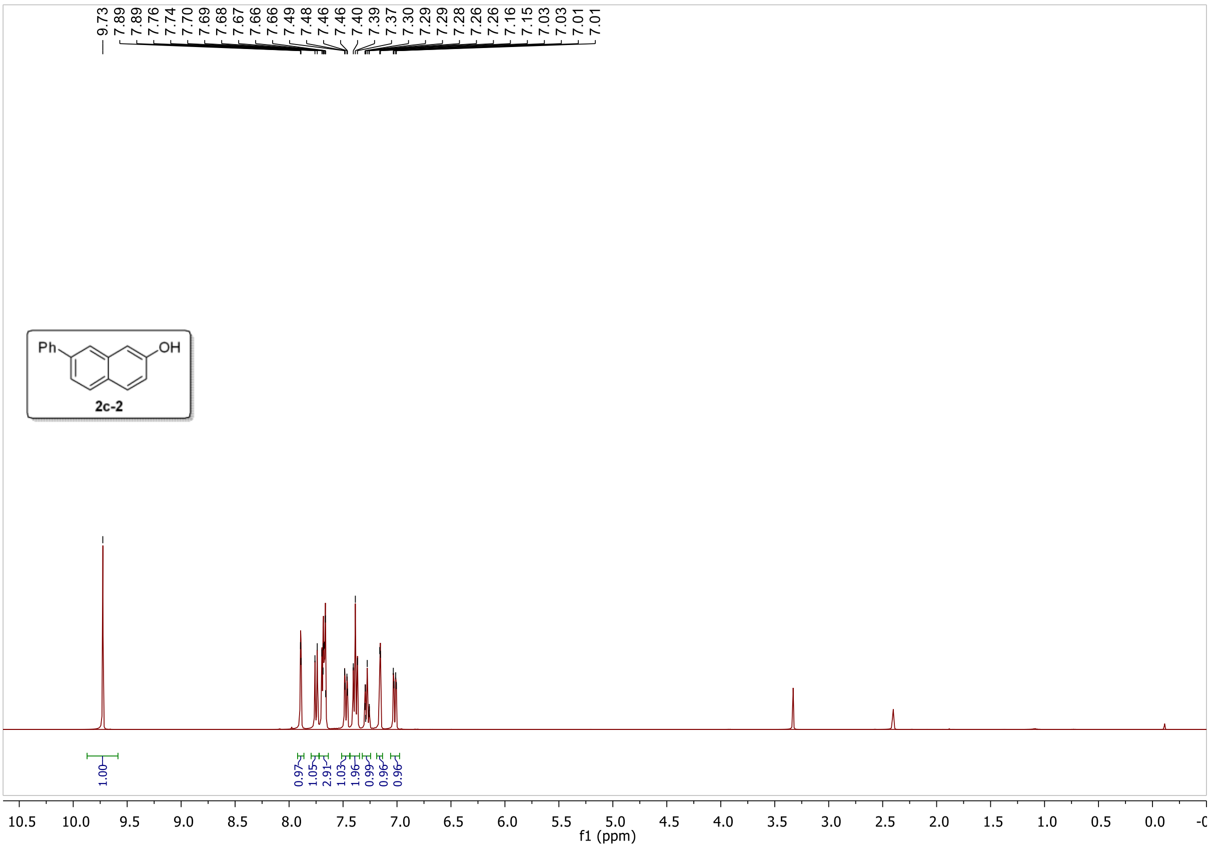

Supplementary Figure 163. ^{13}C NMR of **2c-2**.

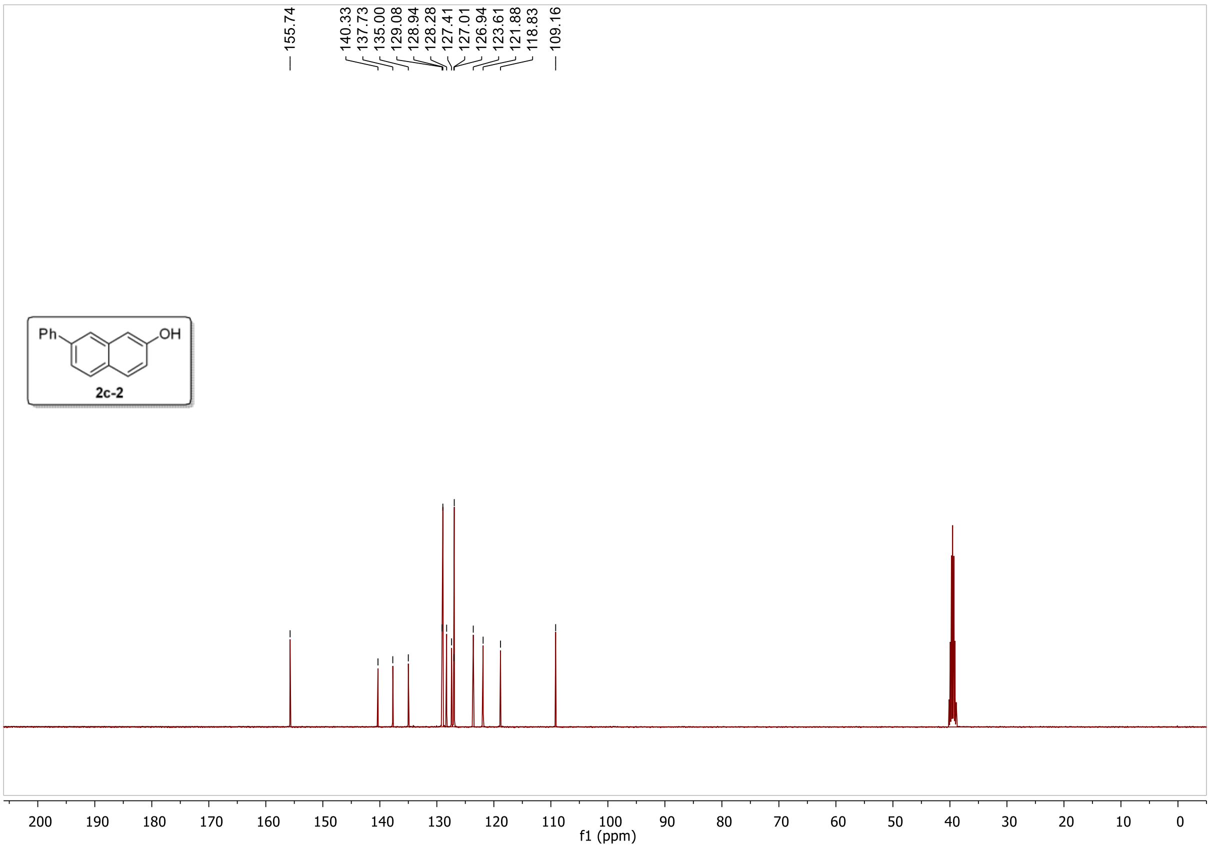

Supplementary Figure 164. ^{1}H NMR of **2d-1**.

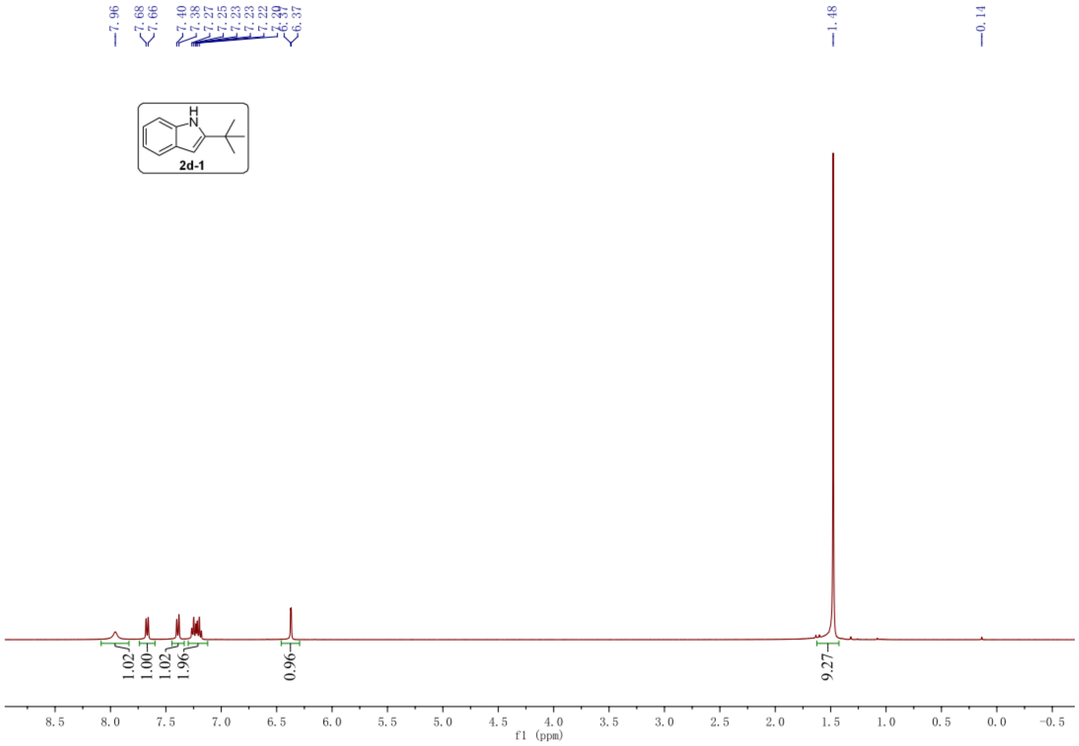

Supplementary Figure 165. ^{13}C NMR of **2d-1**.

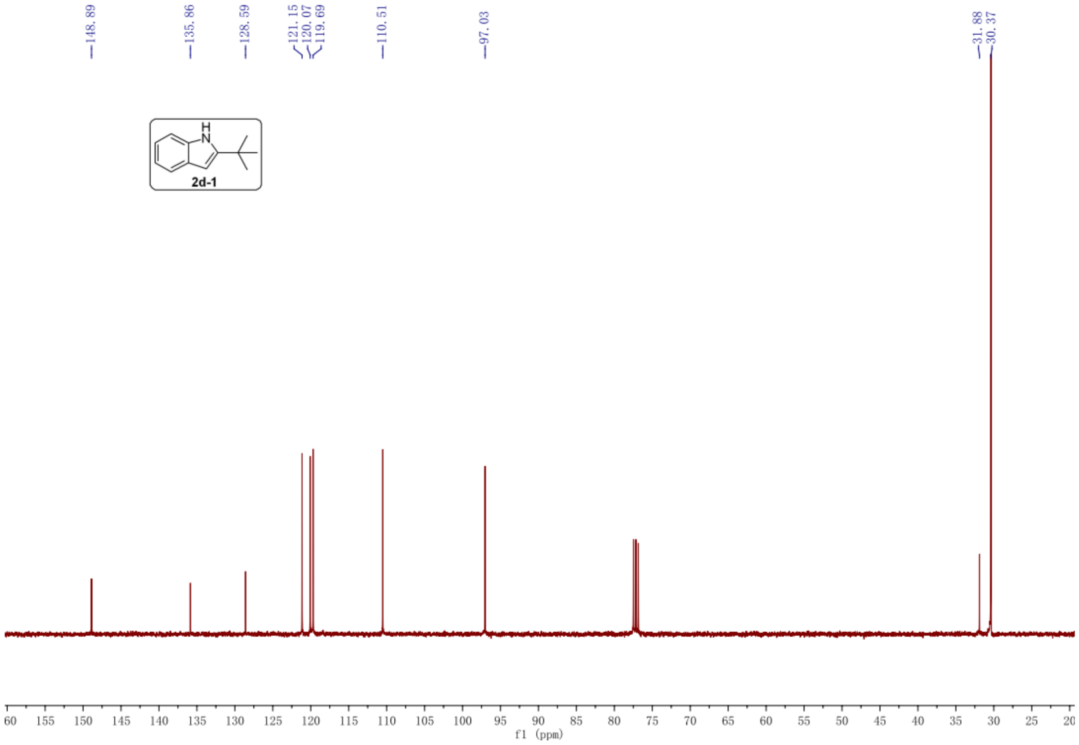

Supplementary Figure 166. ^{1}H NMR of **2d-2**.

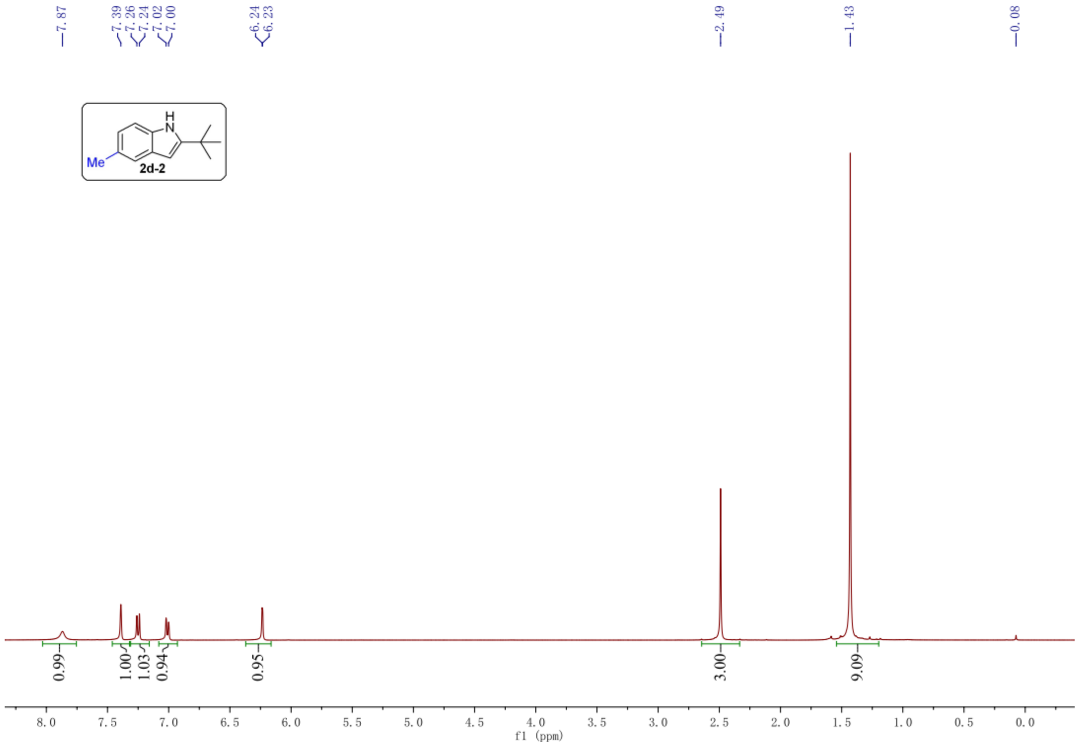

Supplementary Figure 167. ^{13}C NMR of **2d-2**.

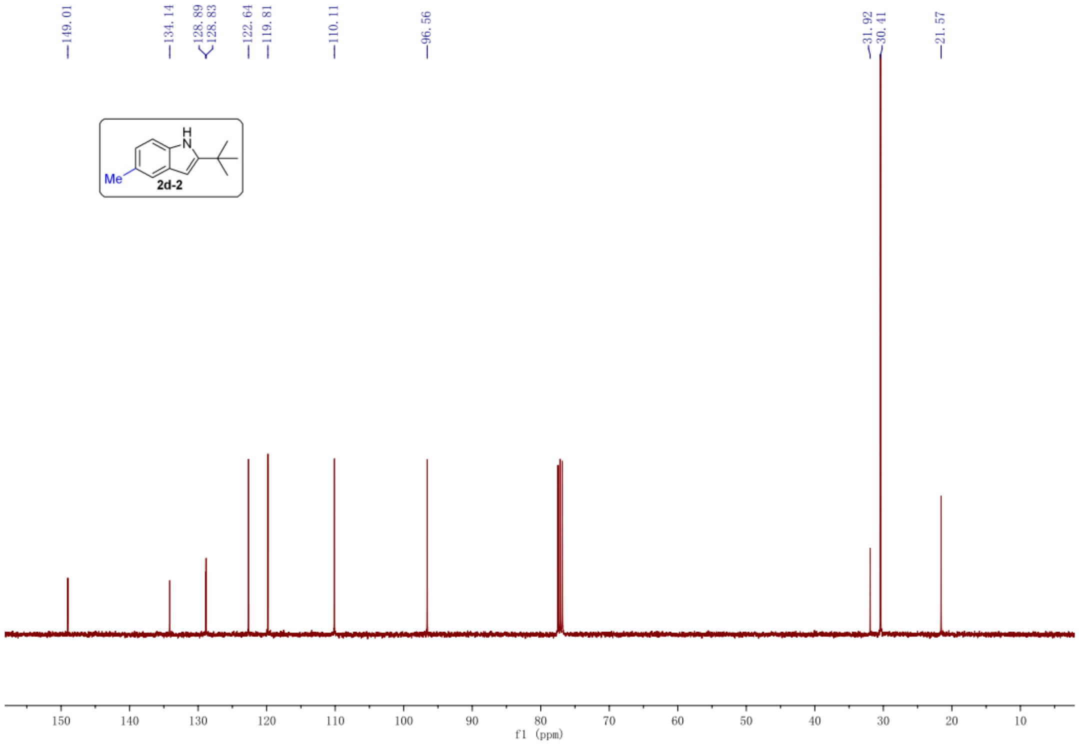

Supplementary Figure 168. ^{1}H NMR of **2d-3**.

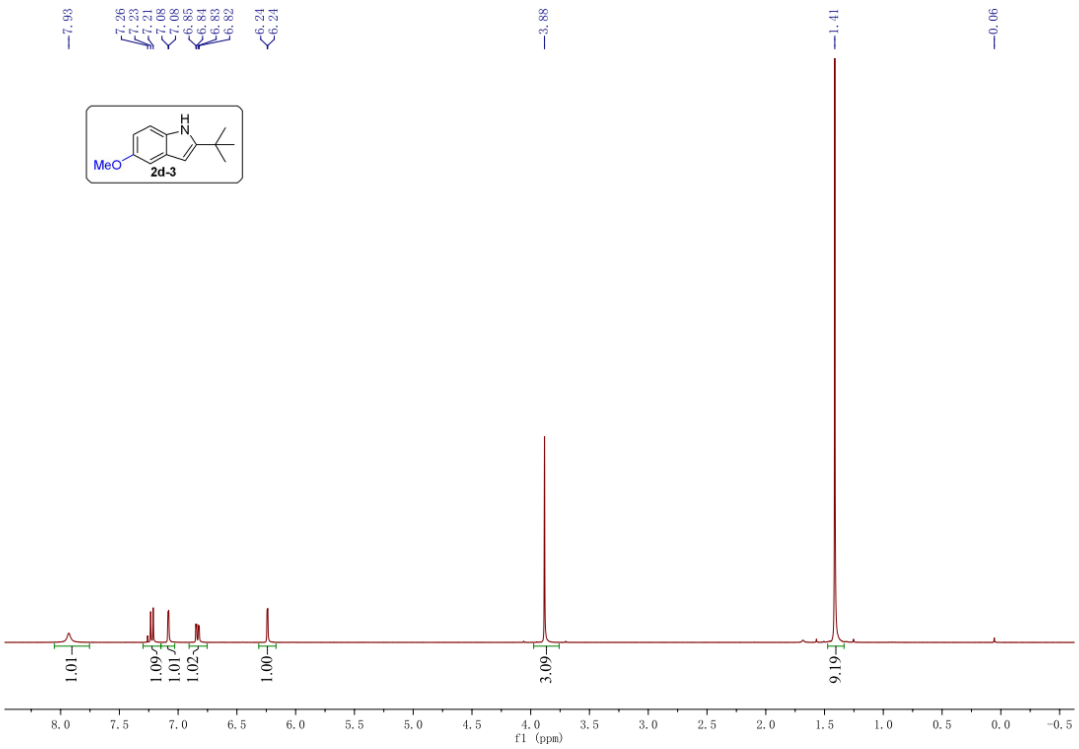

Supplementary Figure 169. ^{13}C NMR of **2d-3**.

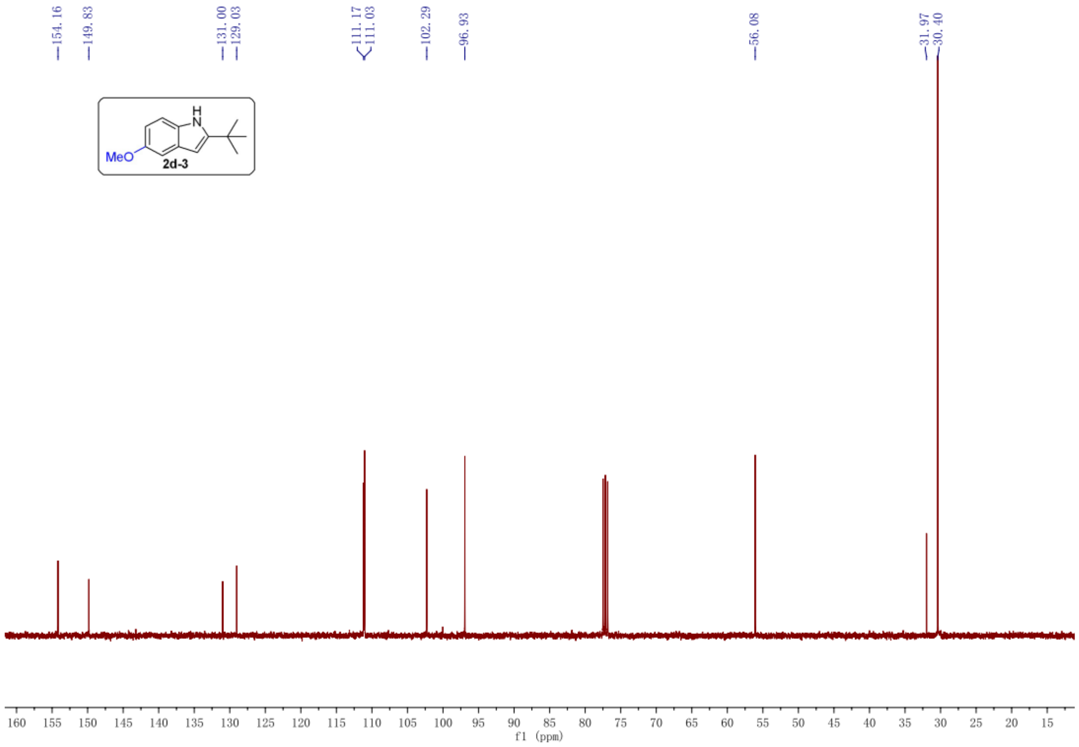

Supplementary Figure 170. ^{1}H NMR of **2d-4**.

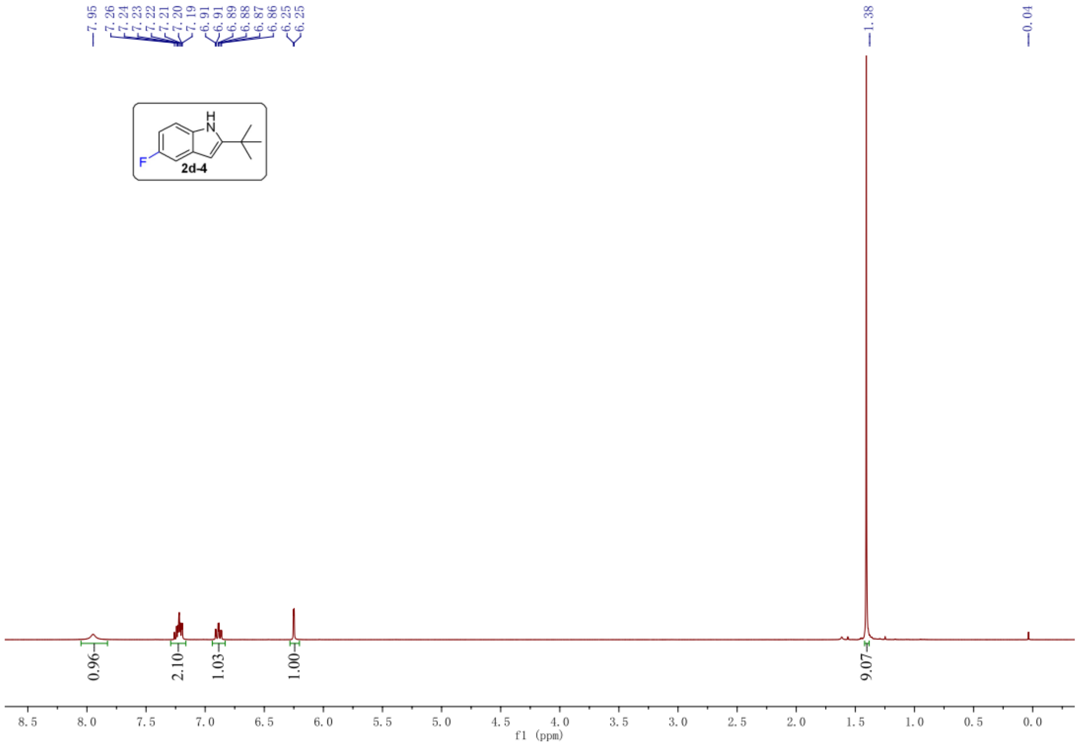

Supplementary Figure 171. ^{13}C NMR of **2d-4**.

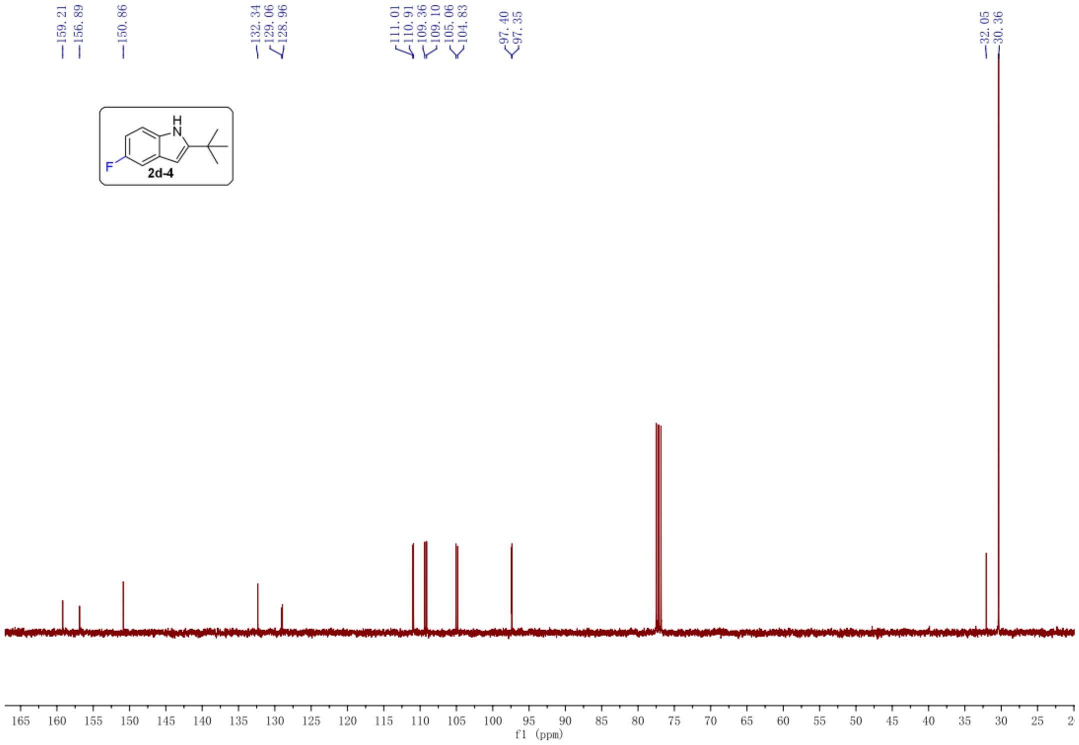

Supplementary Figure 172. ^{1}H NMR of **2d-5**.

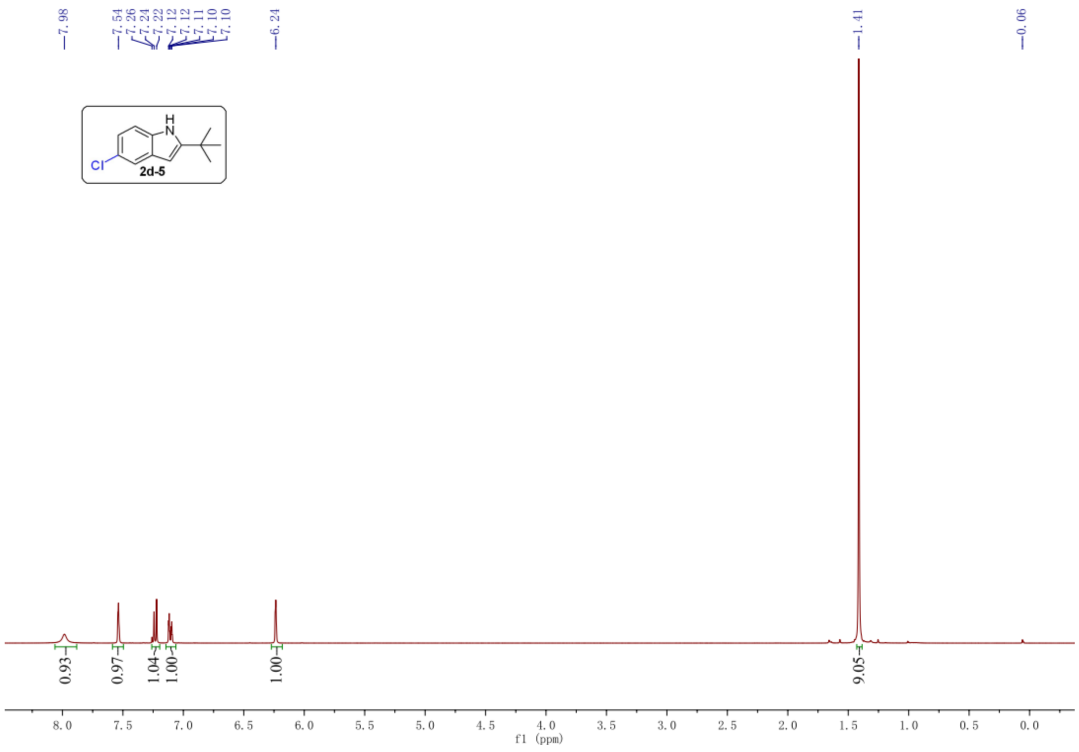

Supplementary Figure 173. ^{13}C NMR of **2d-5**.

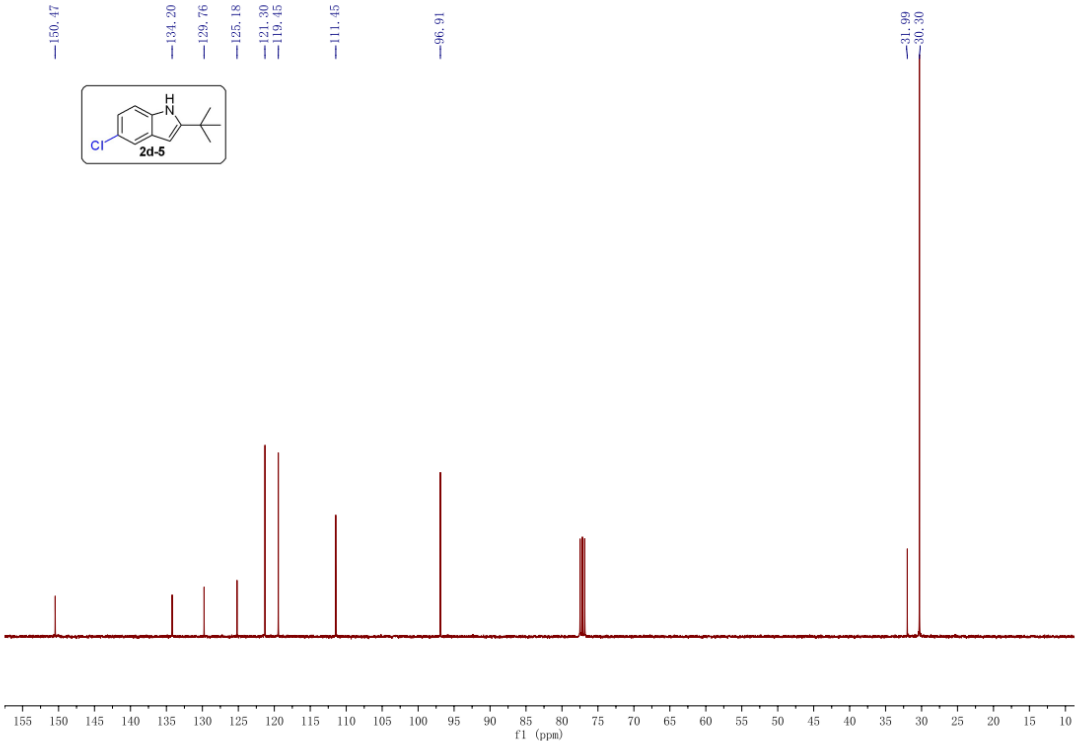

Supplementary Figure 174. ^{1}H NMR of **2d-6**.

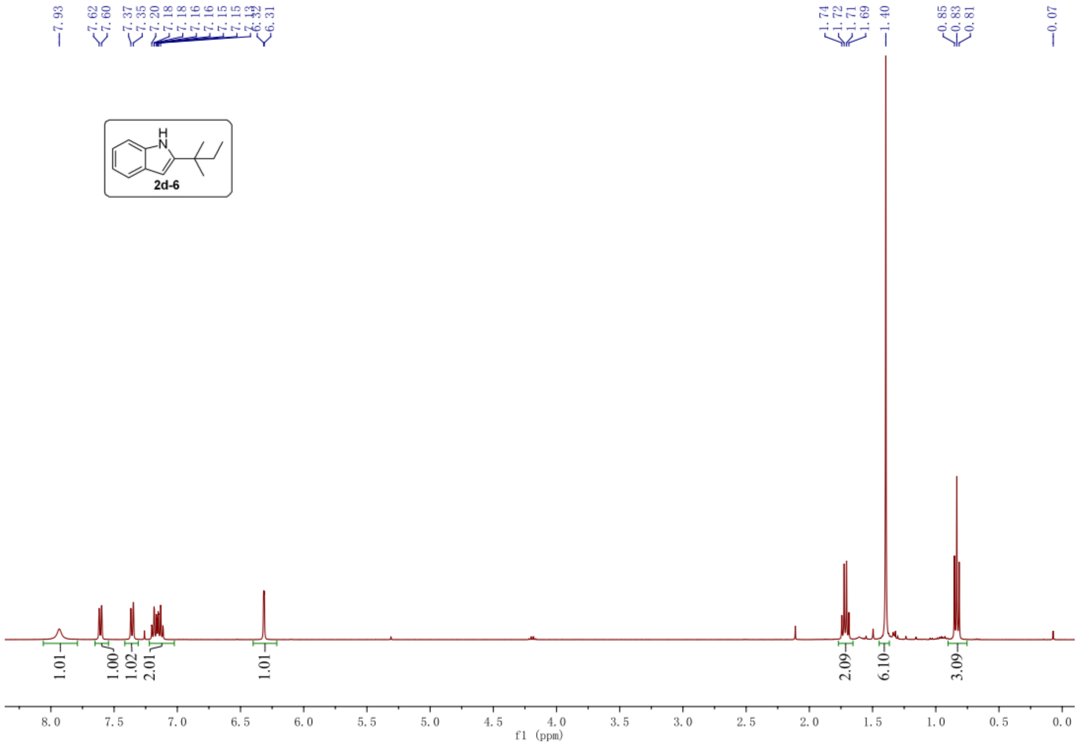

Supplementary Figure 175. ^{13}C NMR of **2d-6**.

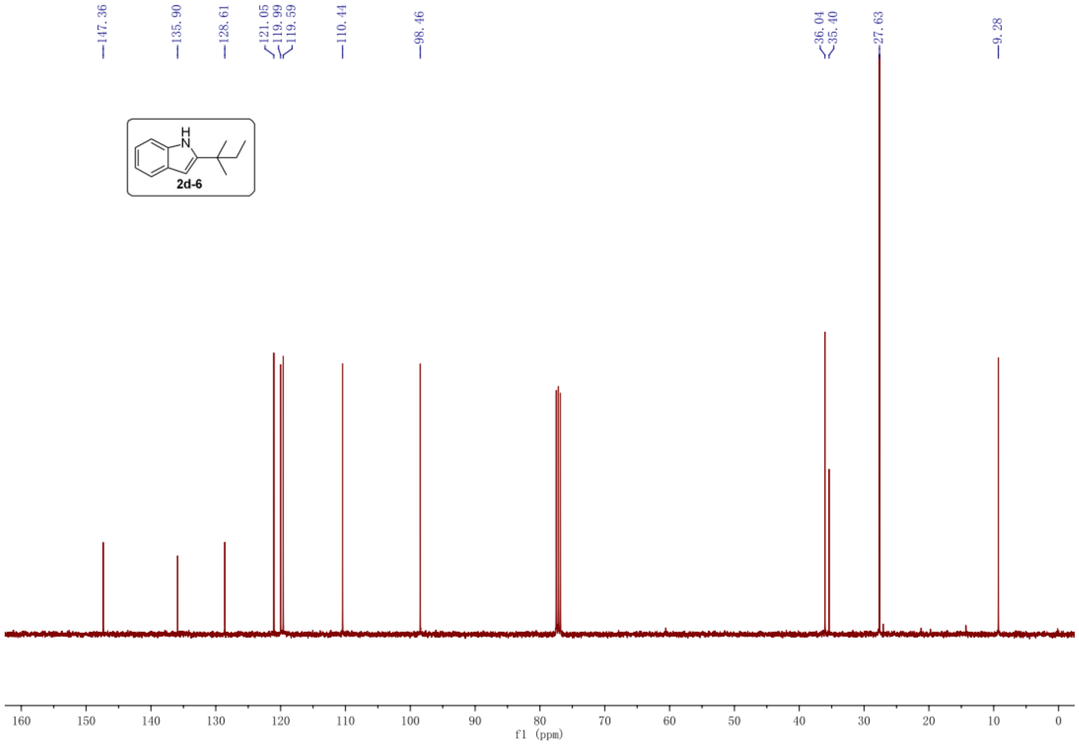

Supplementary Figure 176. ^{1}H NMR of **3a-1**.

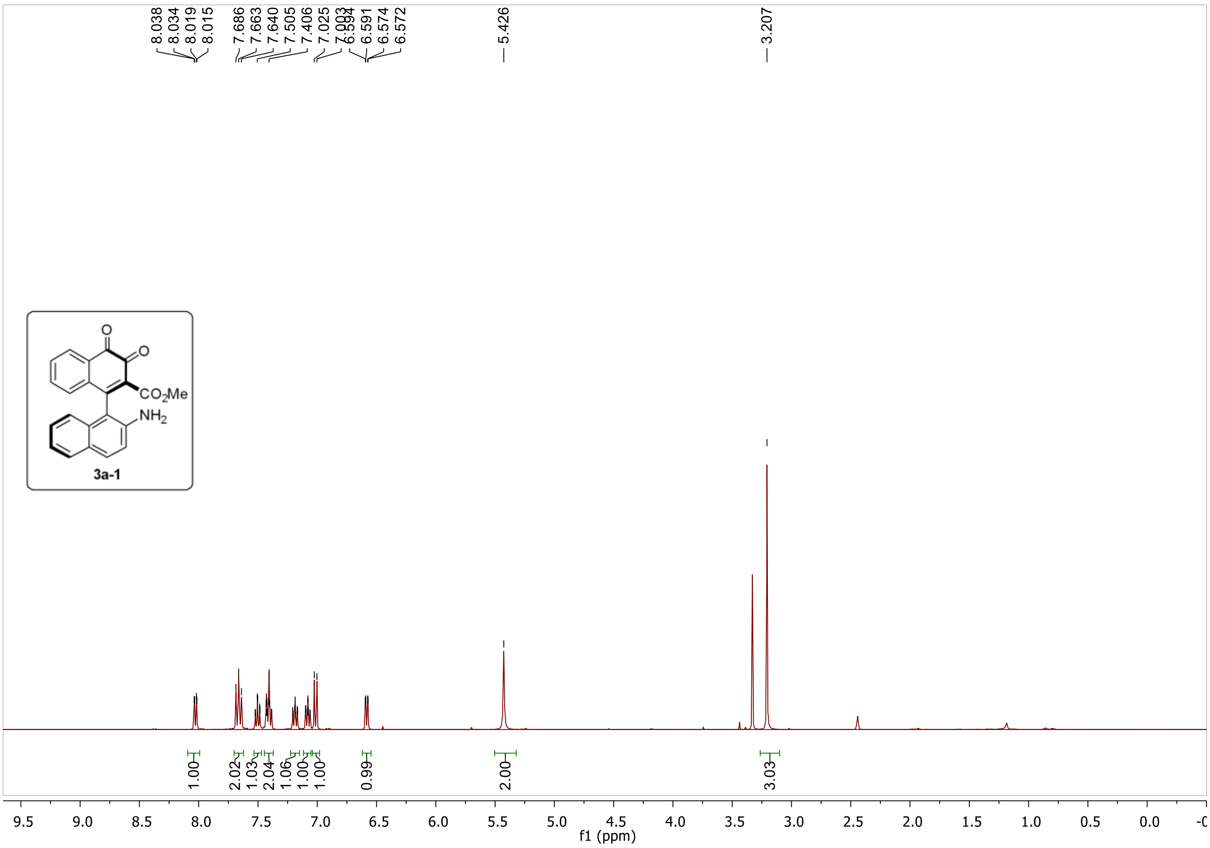

Supplementary Figure 177. ^{13}C NMR of **3a-1**.

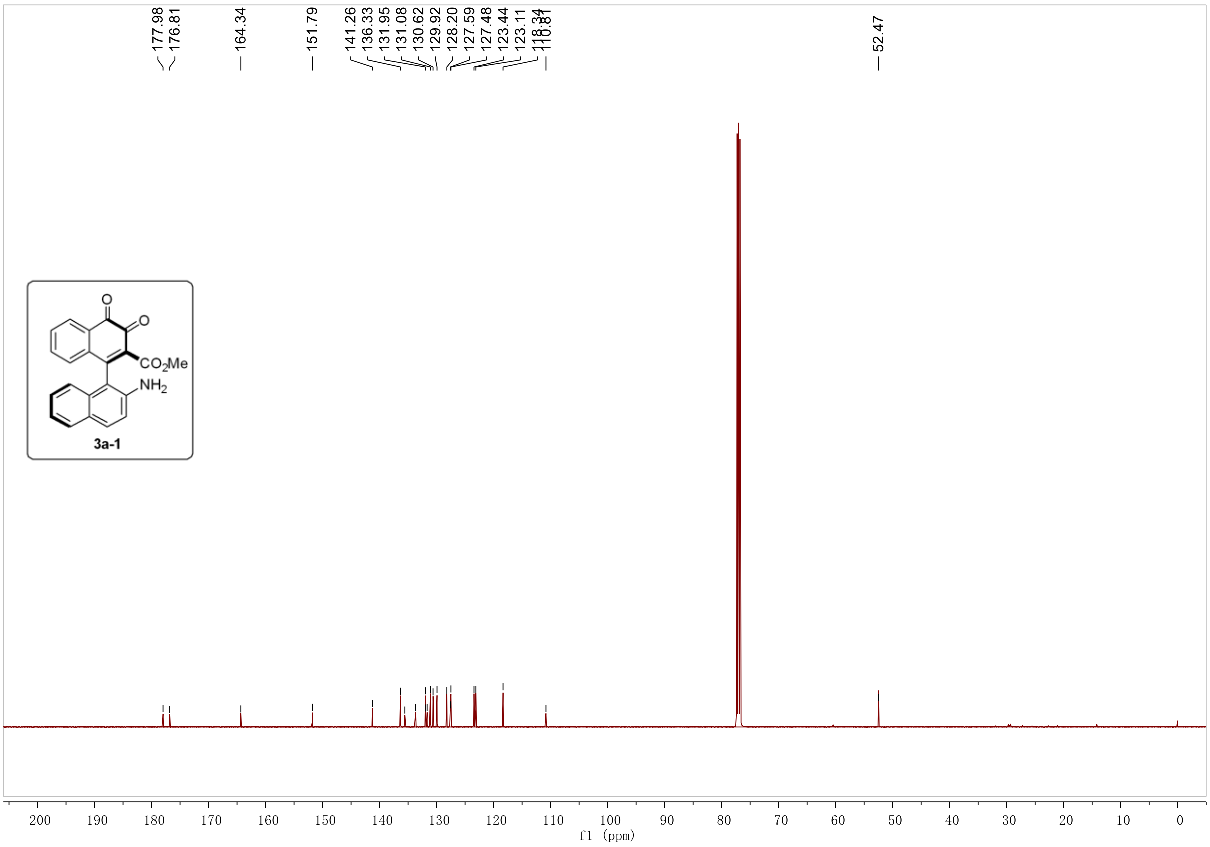

Supplementary Figure 178. ^{1}H NMR of **3a-2**.

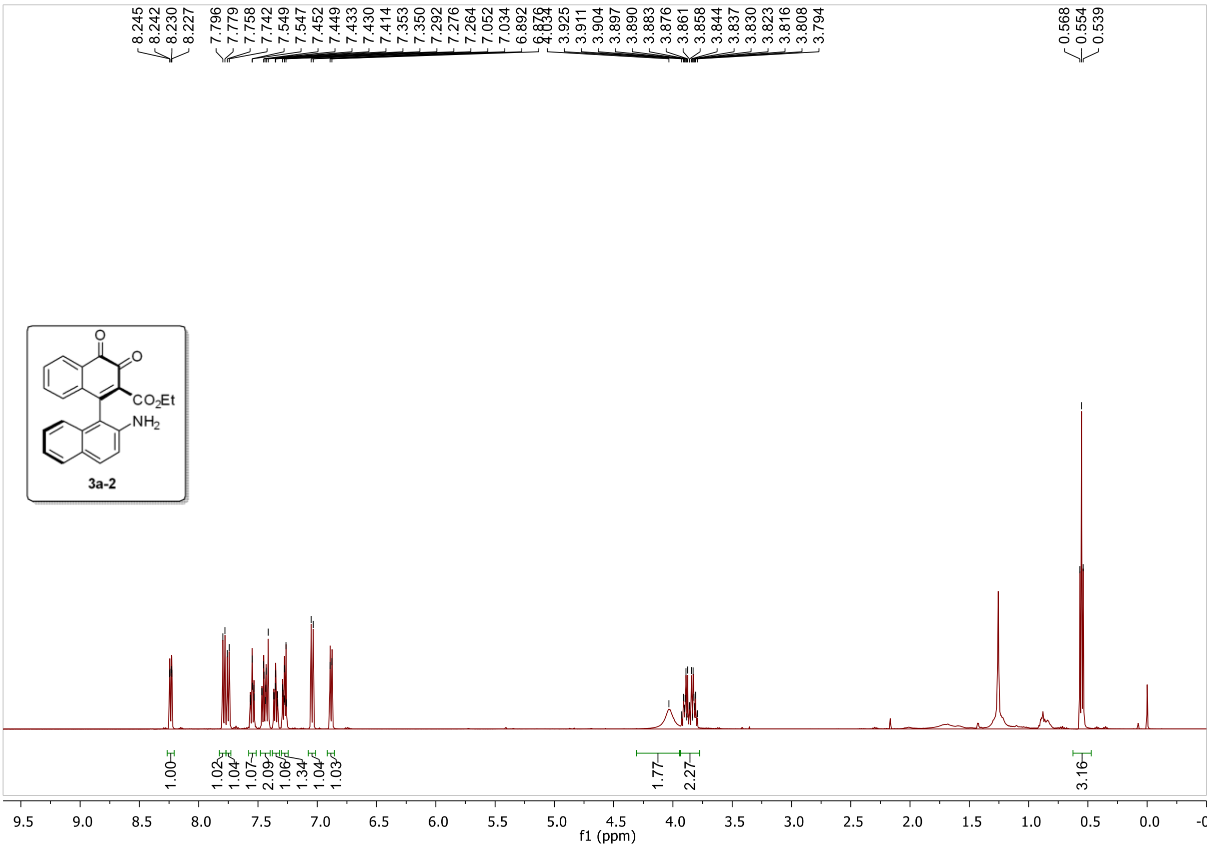

Supplementary Figure 179. ^{13}C NMR of **3a-2**.

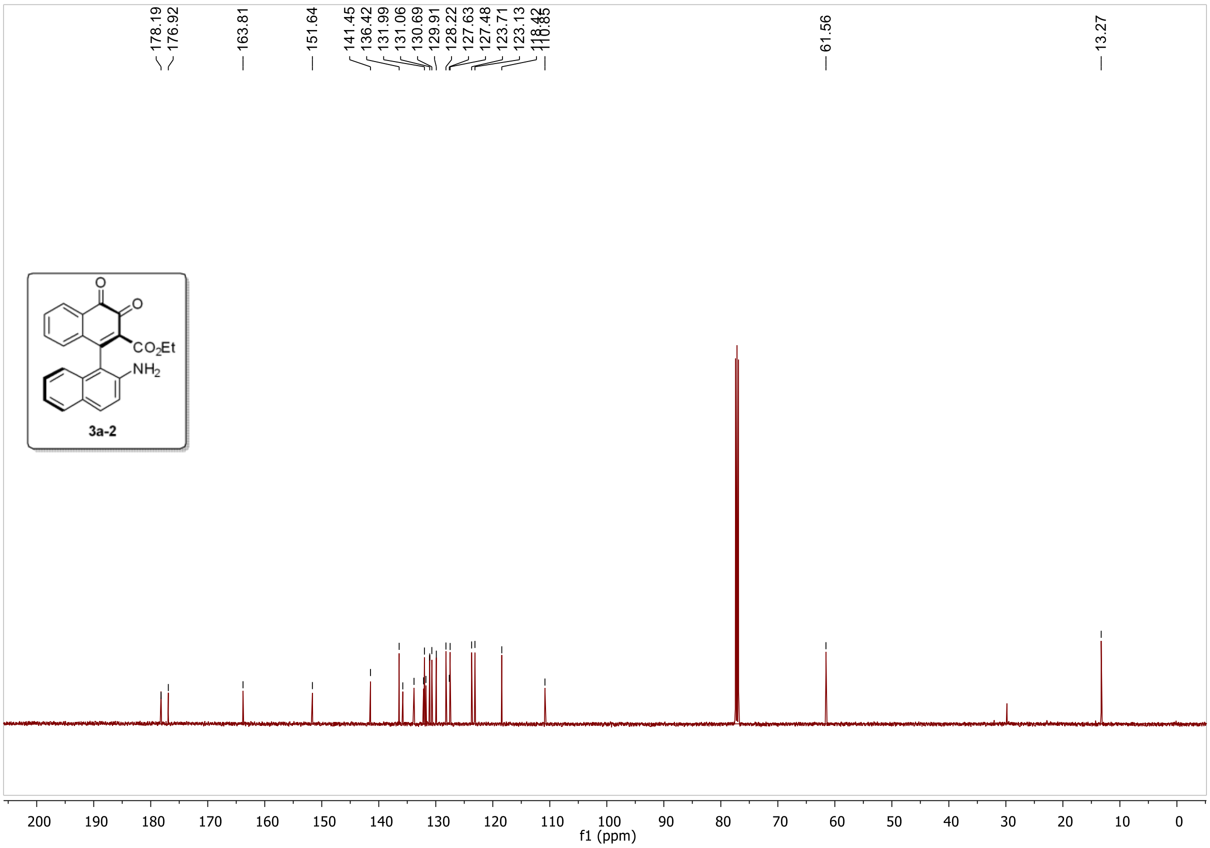

Supplementary Figure 180. ^{1}H NMR of **3a-3**.

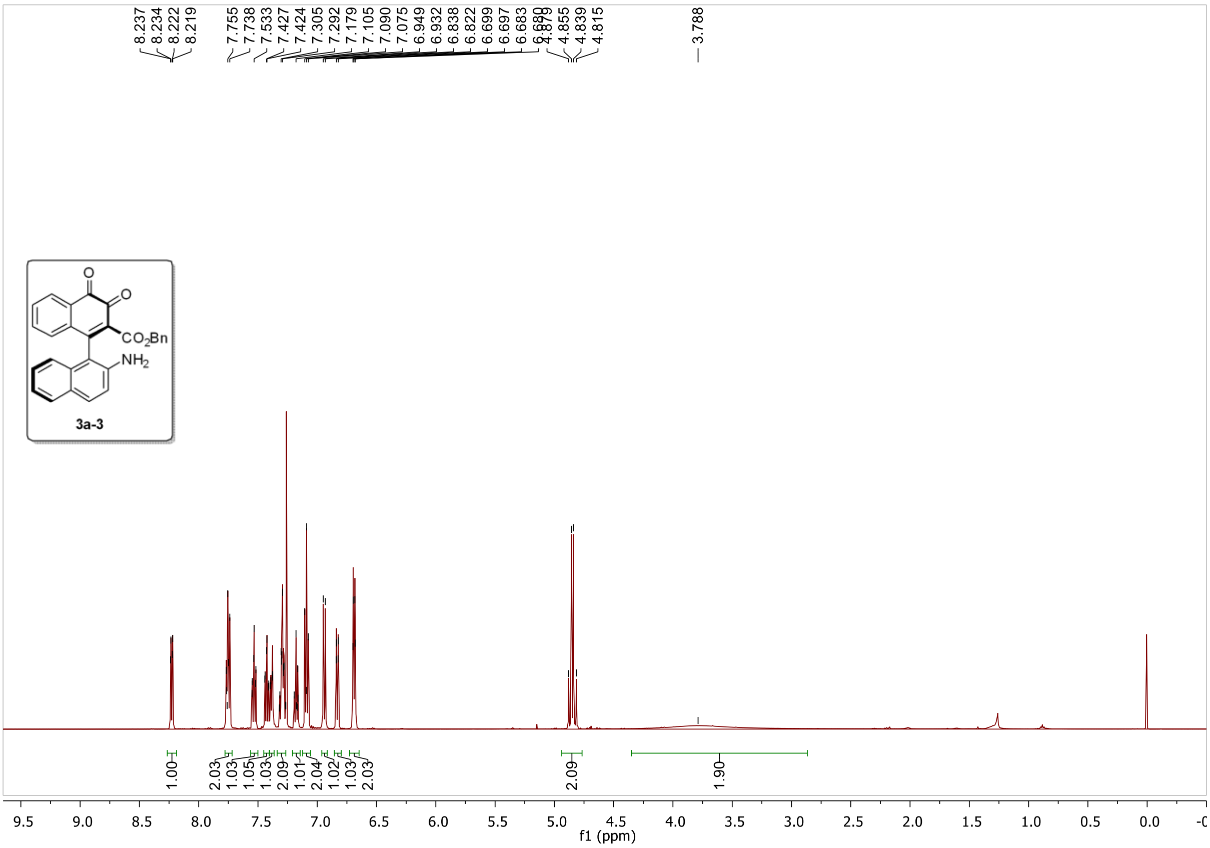

Supplementary Figure 181. ^{13}C NMR of **3a-3**.

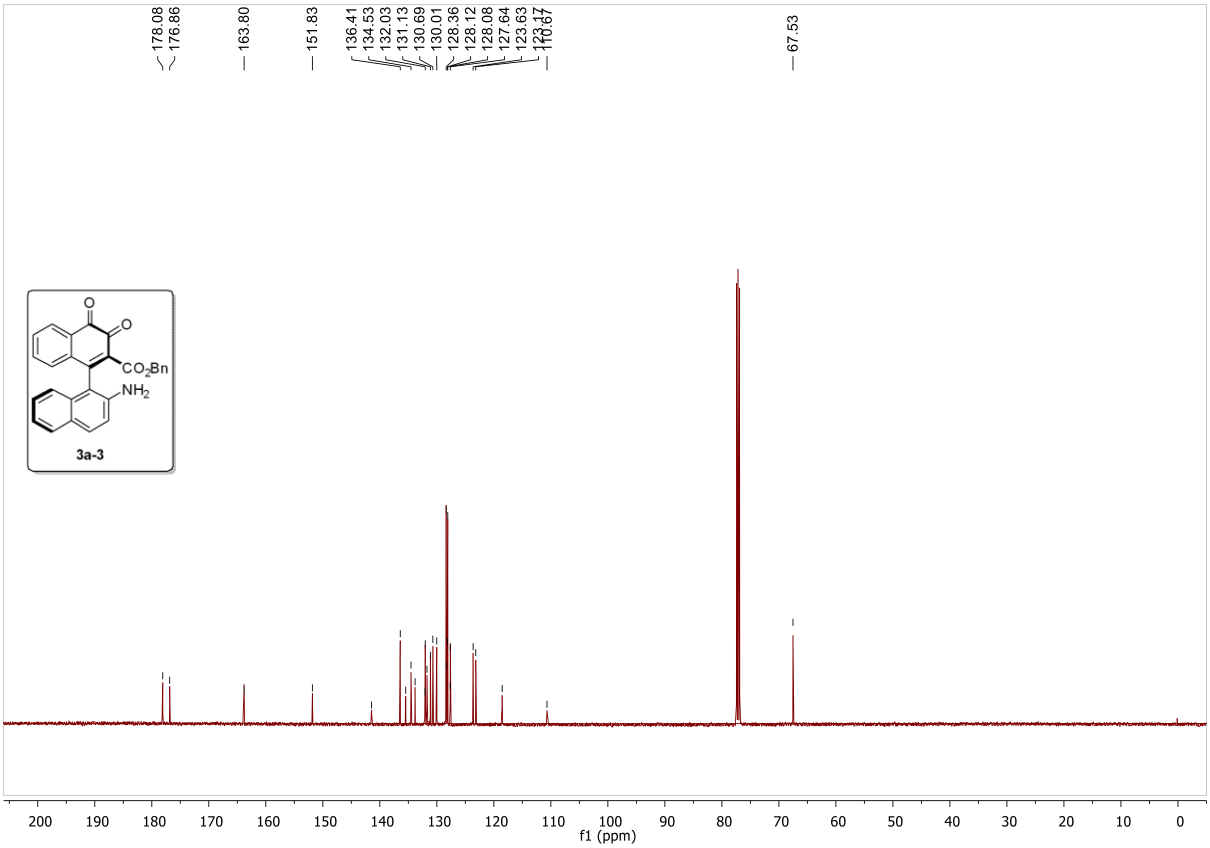

Supplementary Figure 182. ^{1}H NMR of **3a-4**.

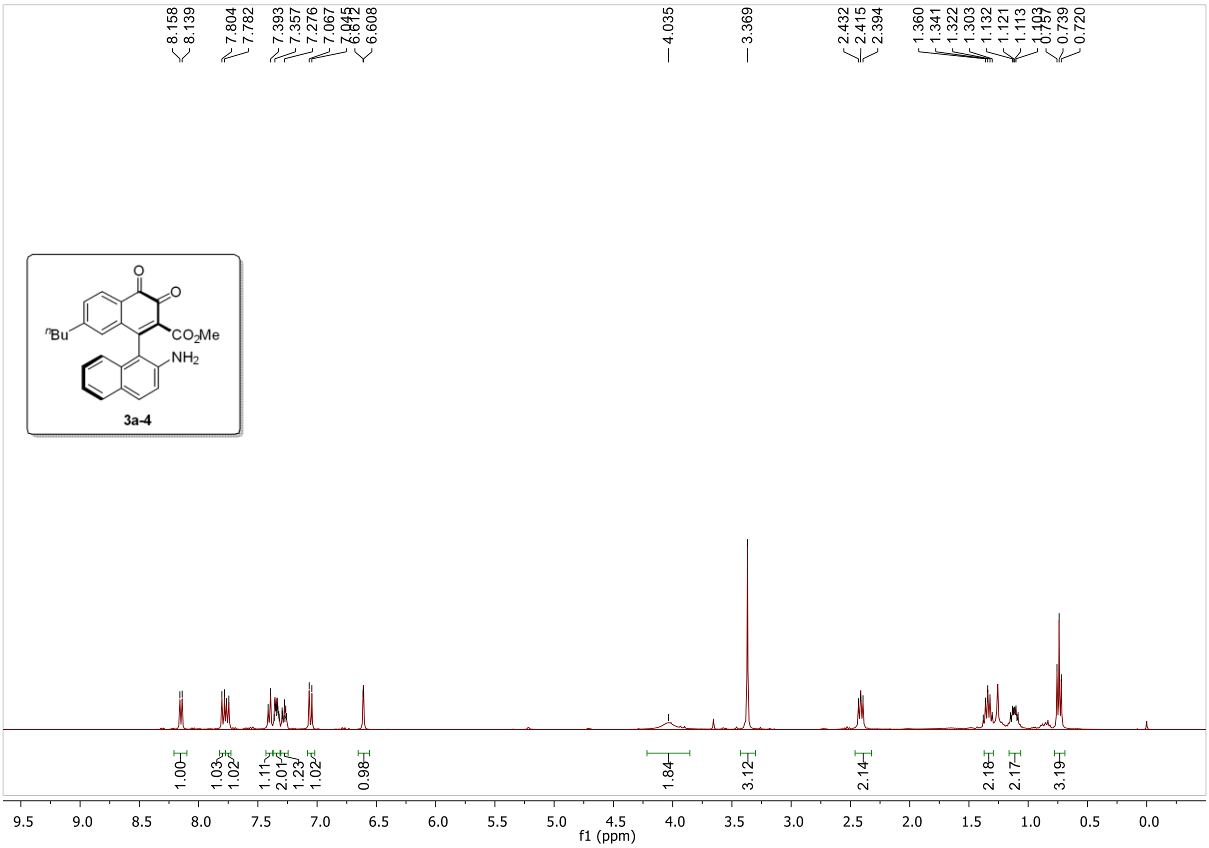

Supplementary Figure 183. ^{13}C NMR of **3a-4**.

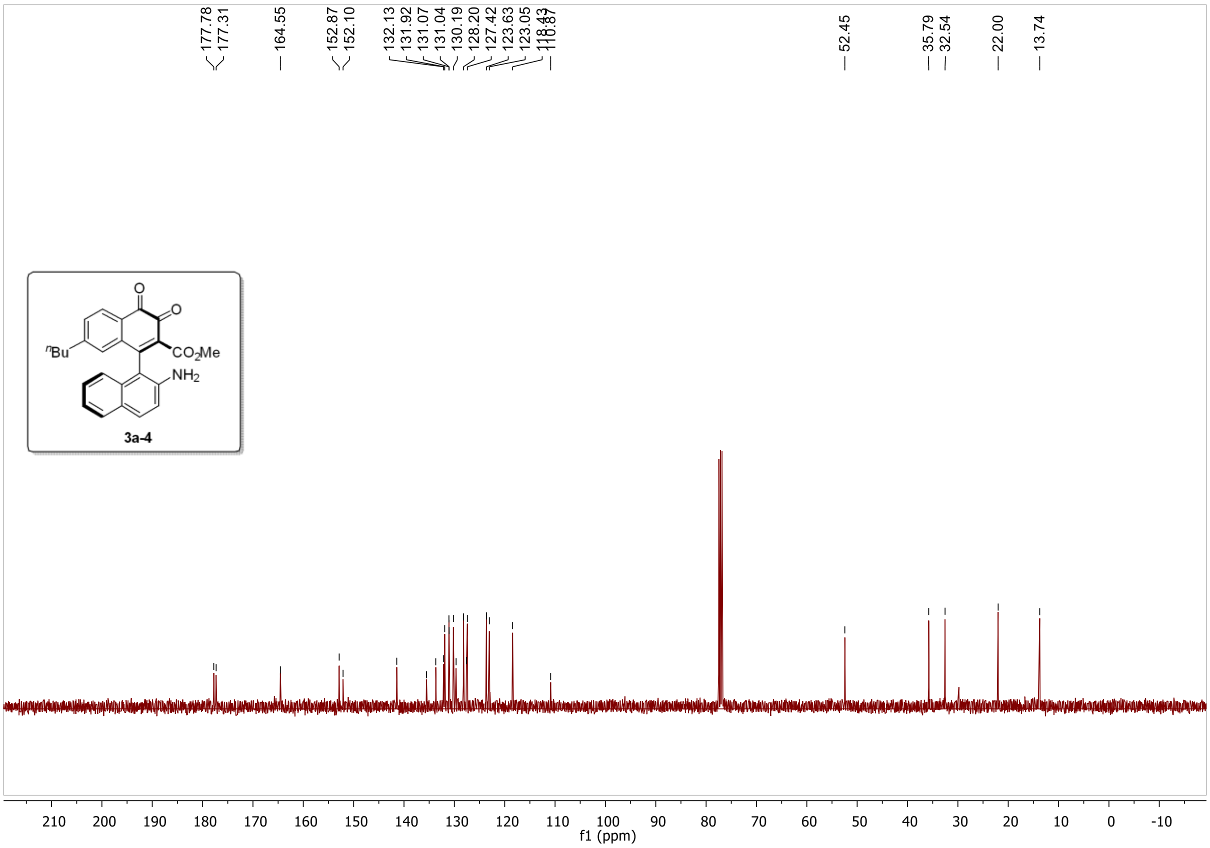

Supplementary Figure 184. ¹H NMR of **3a-5**.

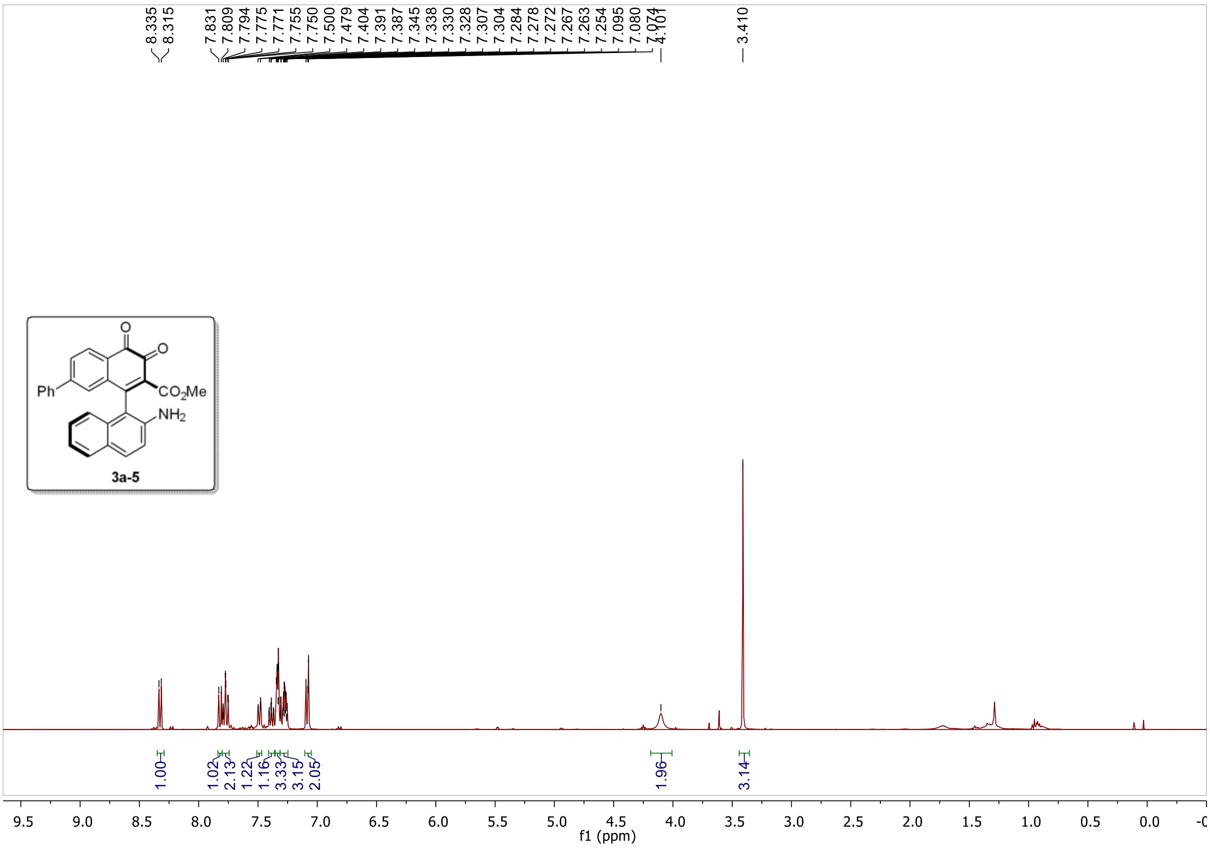

Supplementary Figure 185. ¹³C NMR of **3a-5**.

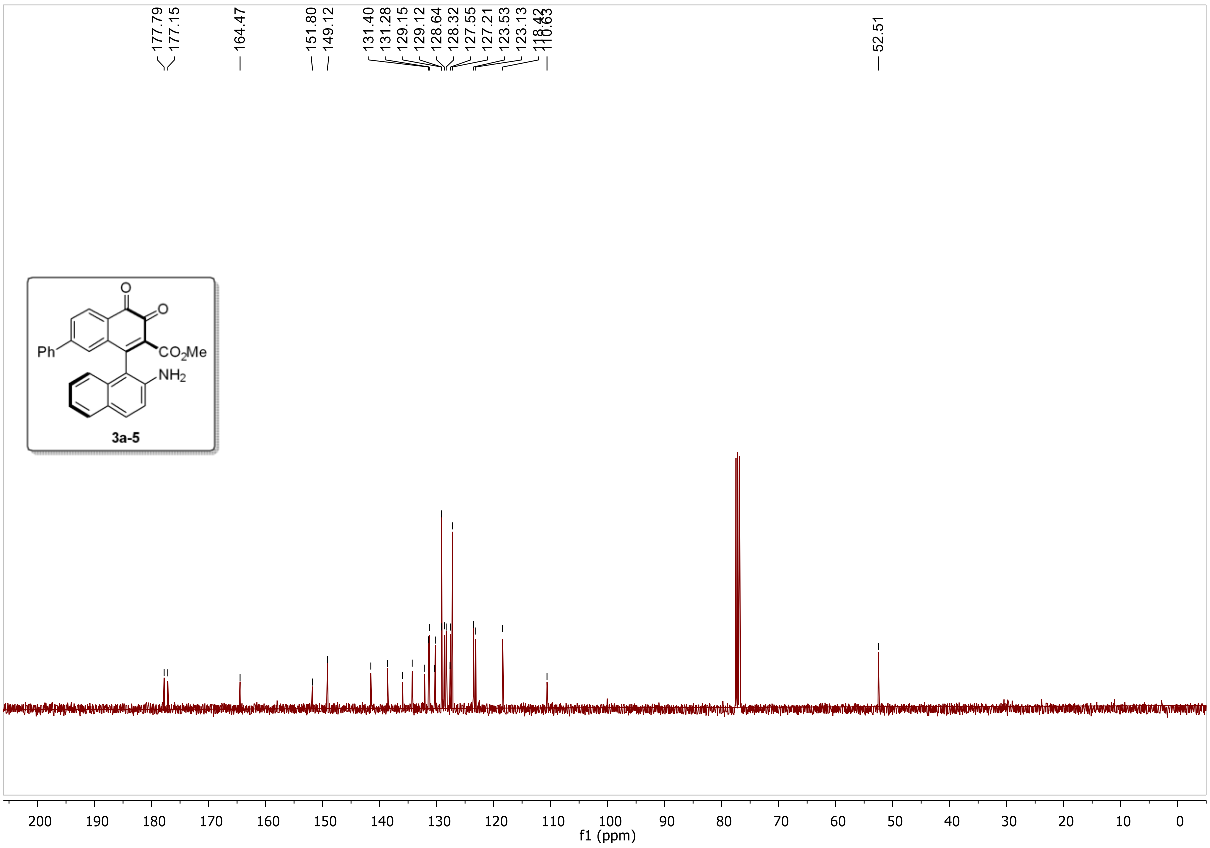

Supplementary Figure 186. ^{1}H NMR of **3a-6**.

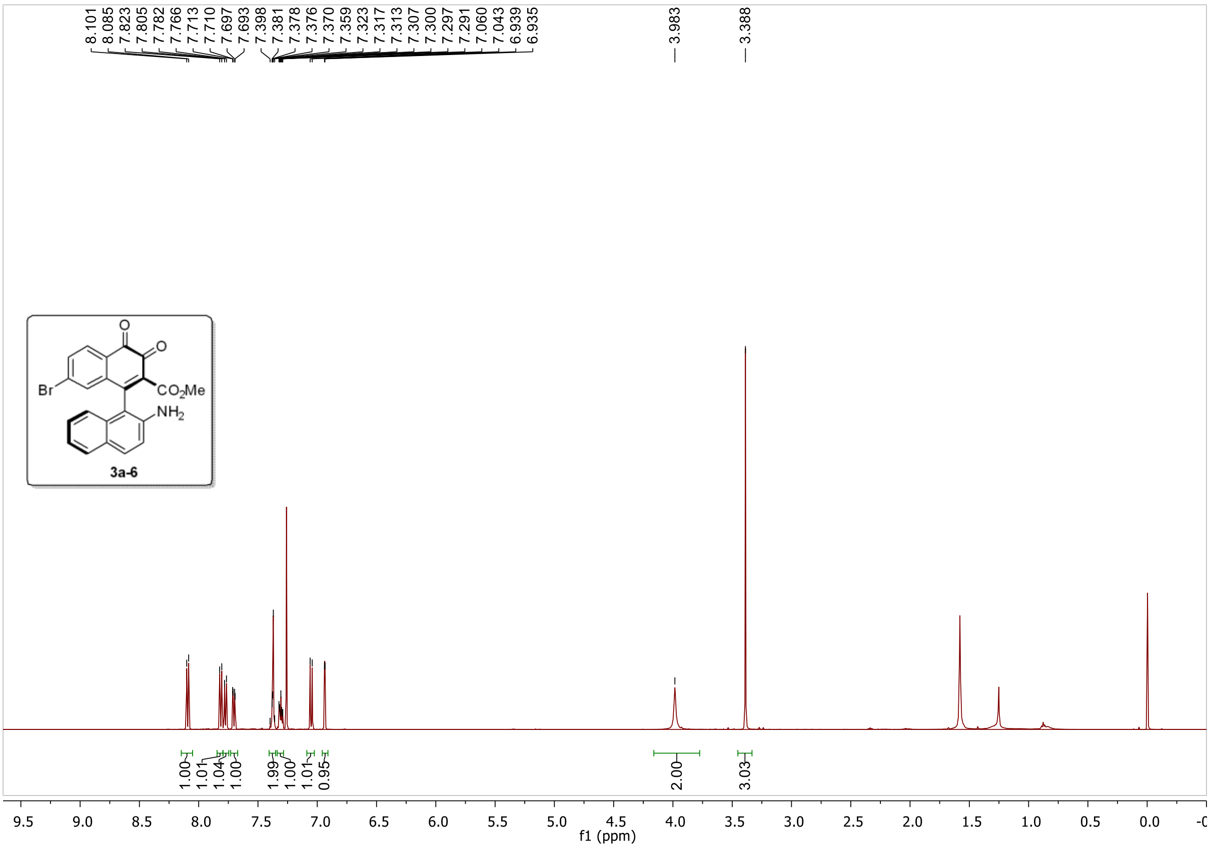

Supplementary Figure 187. ^{13}C NMR of **3a-6**.

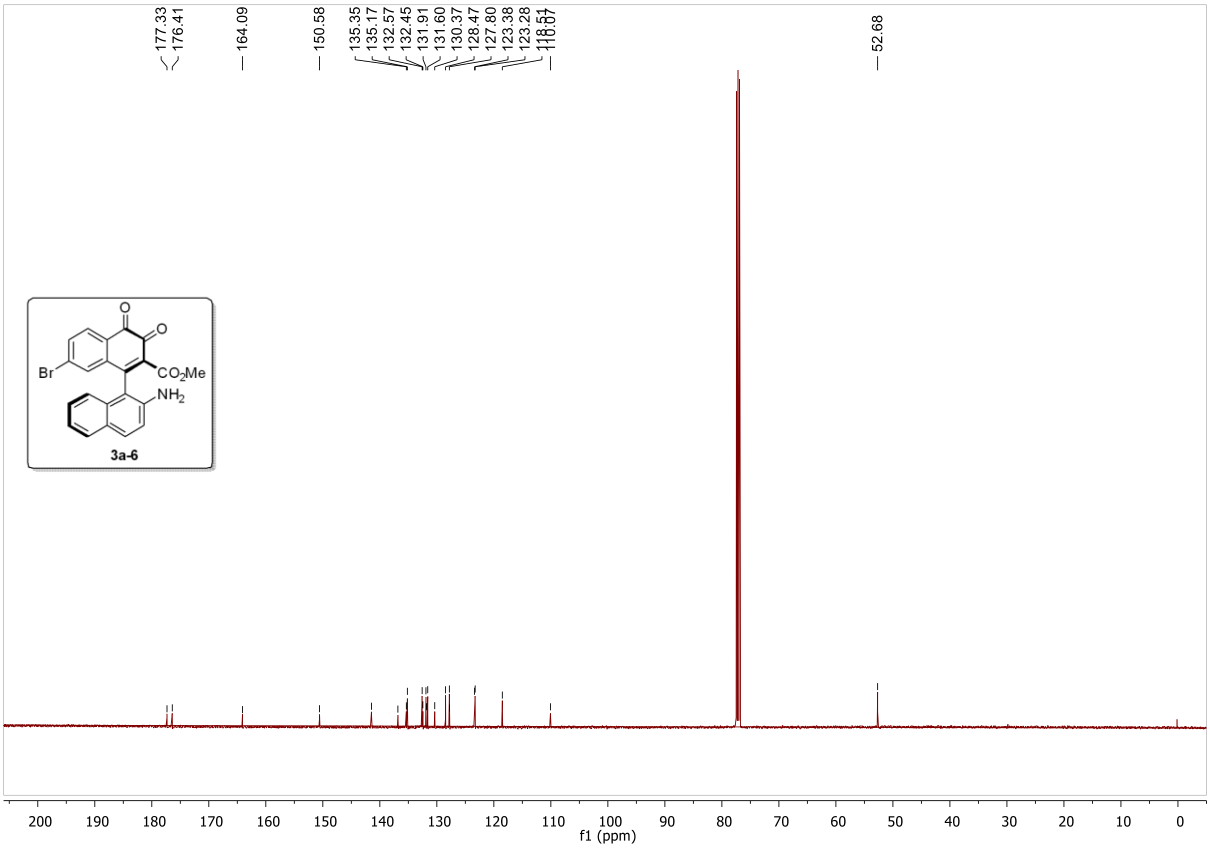

Supplementary Figure 188. ¹H NMR of **3a-7**.

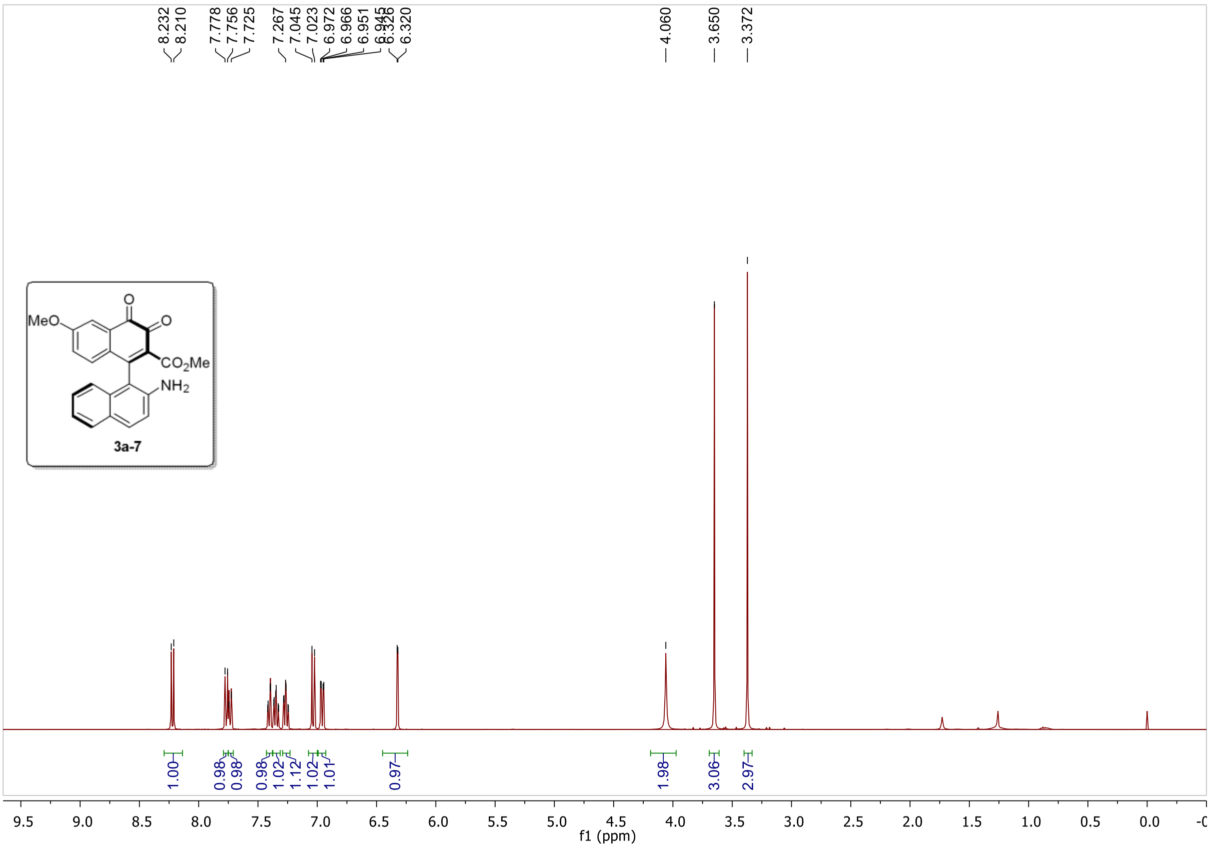

Supplementary Figure 189. ¹³C NMR of **3a-7**.

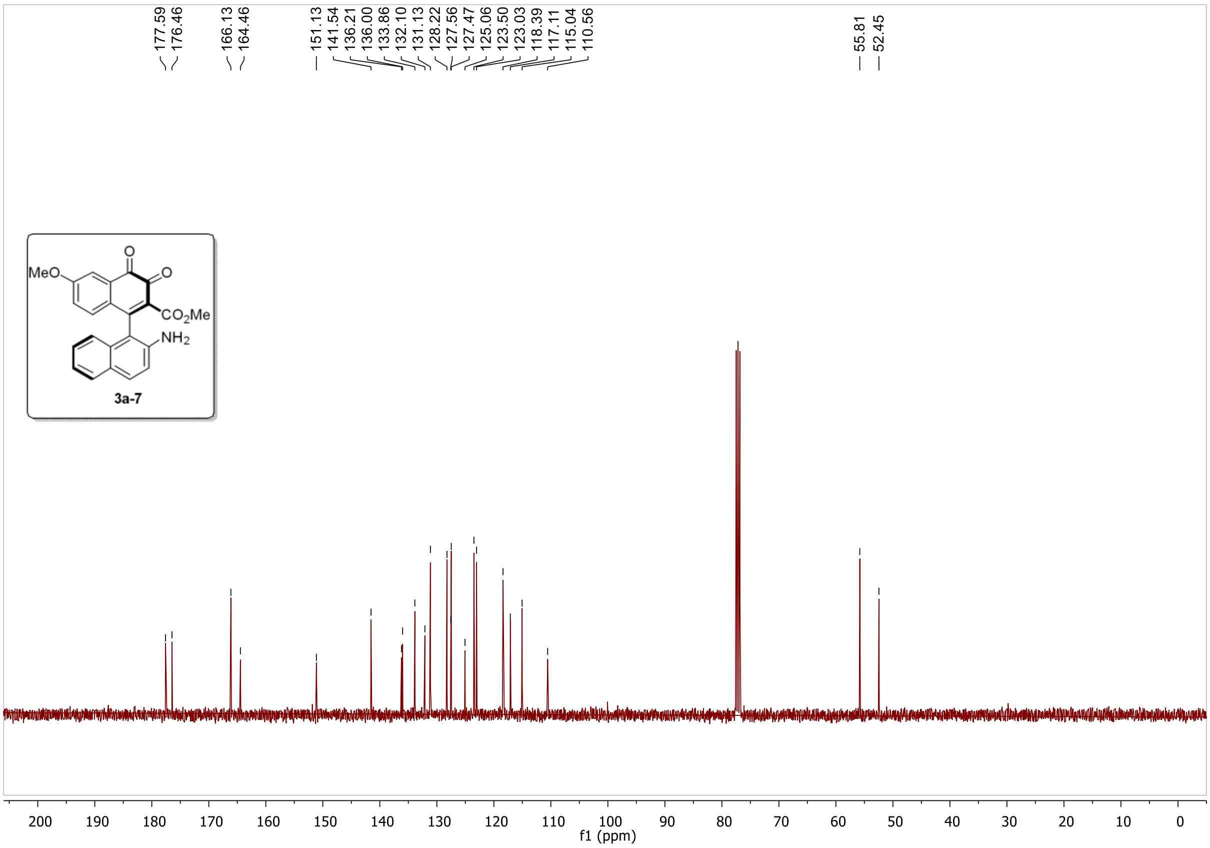

Supplementary Figure 190. ^{1}H NMR of **3a-8**.

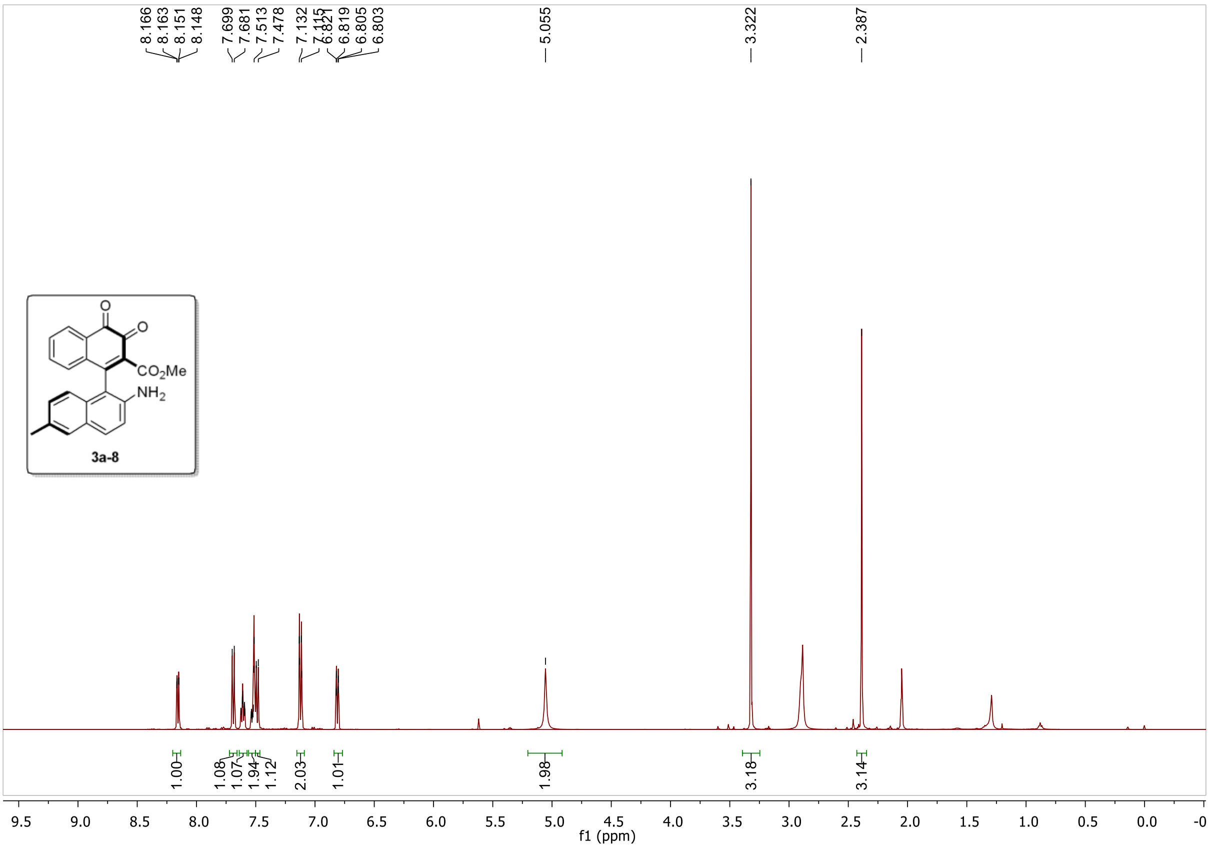

Supplementary Figure 191. ^{13}C NMR of **3a-8**.

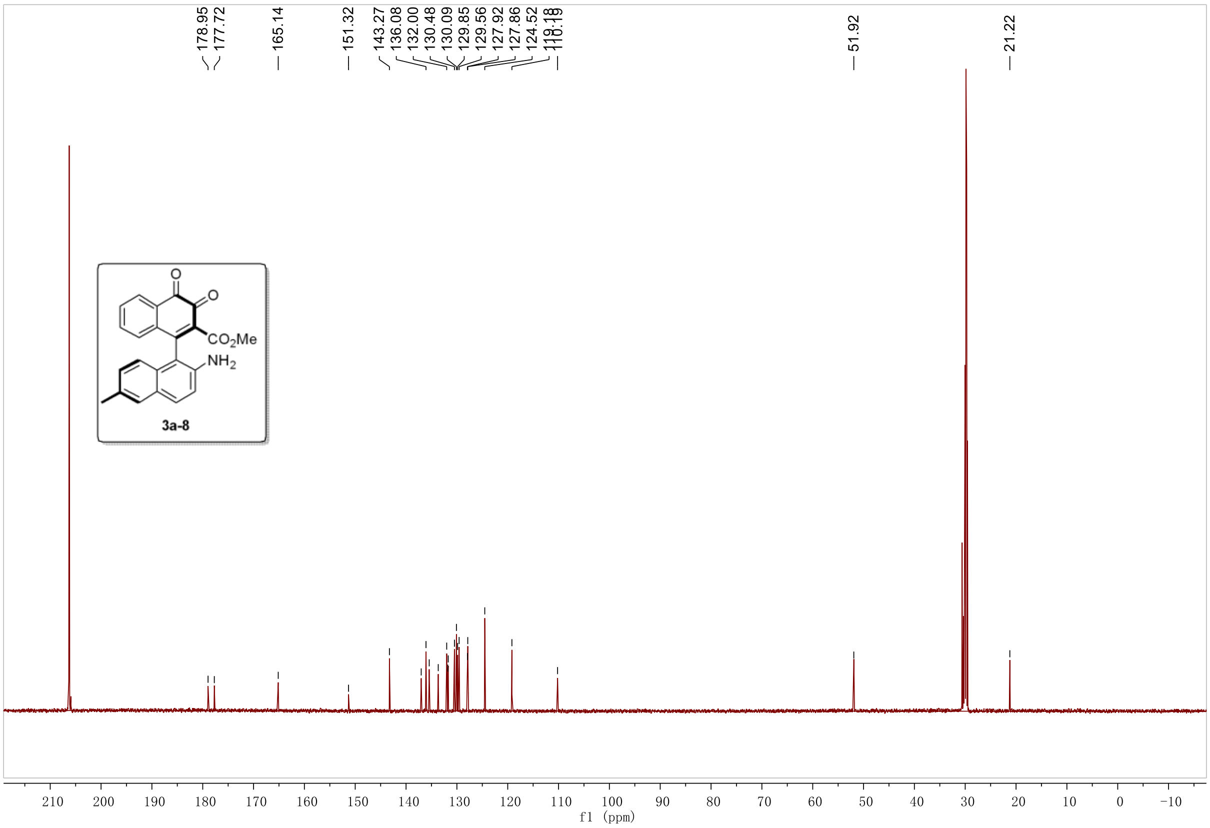

Supplementary Figure 192. ^{1}H NMR of **3a-9**.

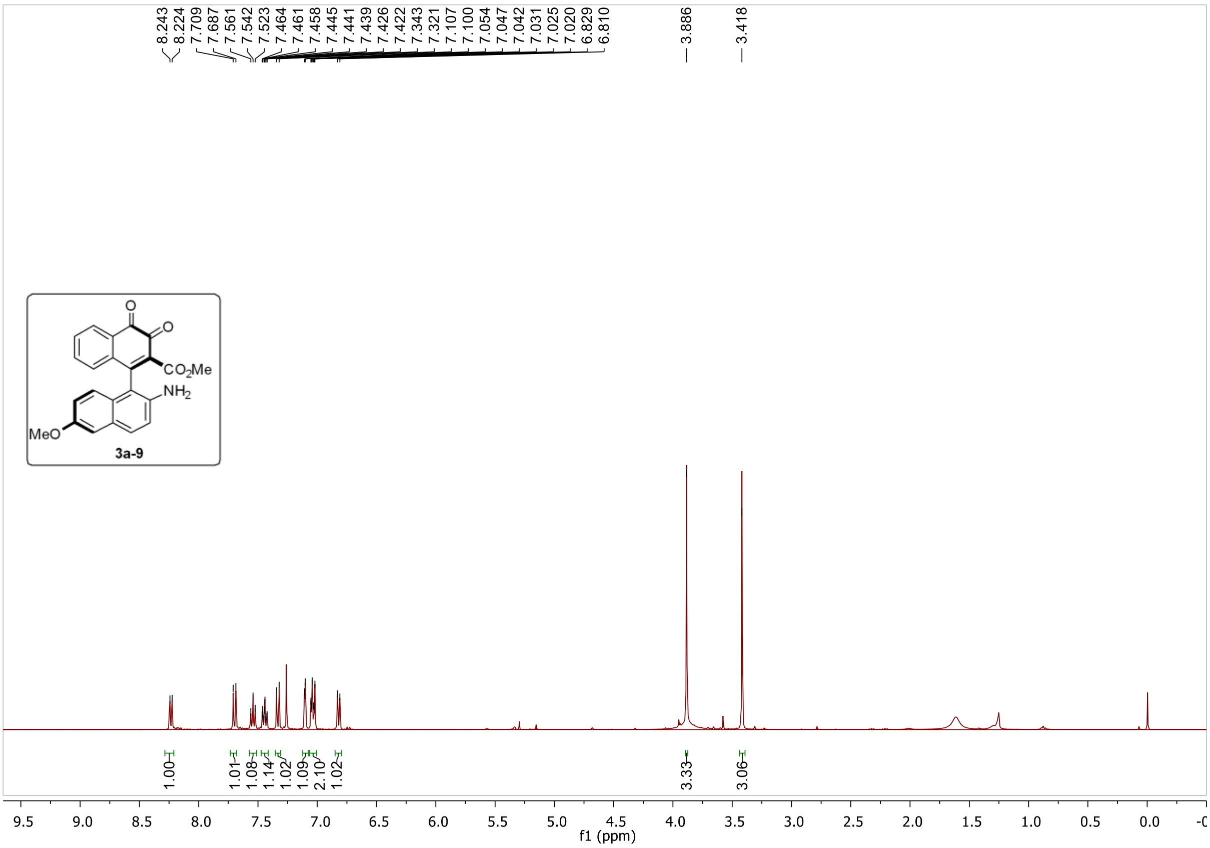

Supplementary Figure 193. ^{13}C NMR of **3a-9**.

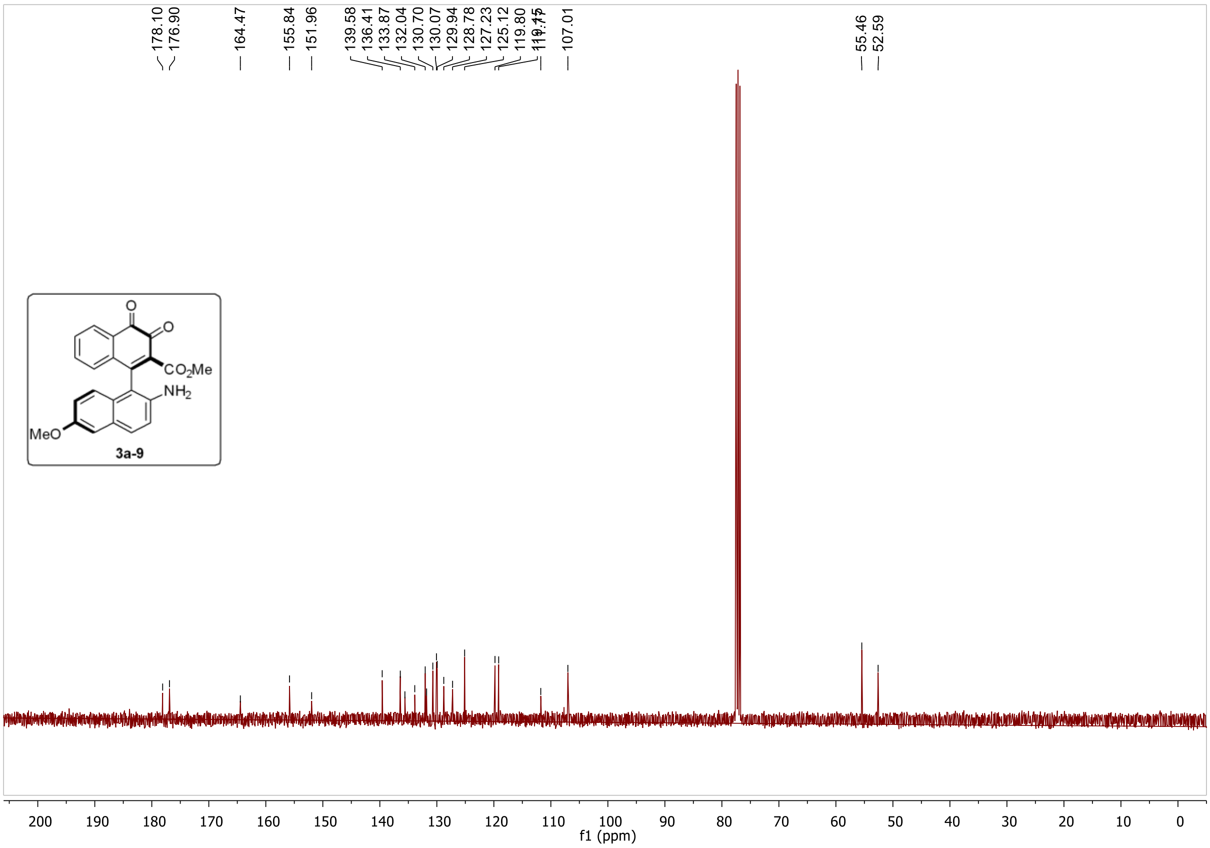

Supplementary Figure 194. ^{1}H NMR of **3a-10**.

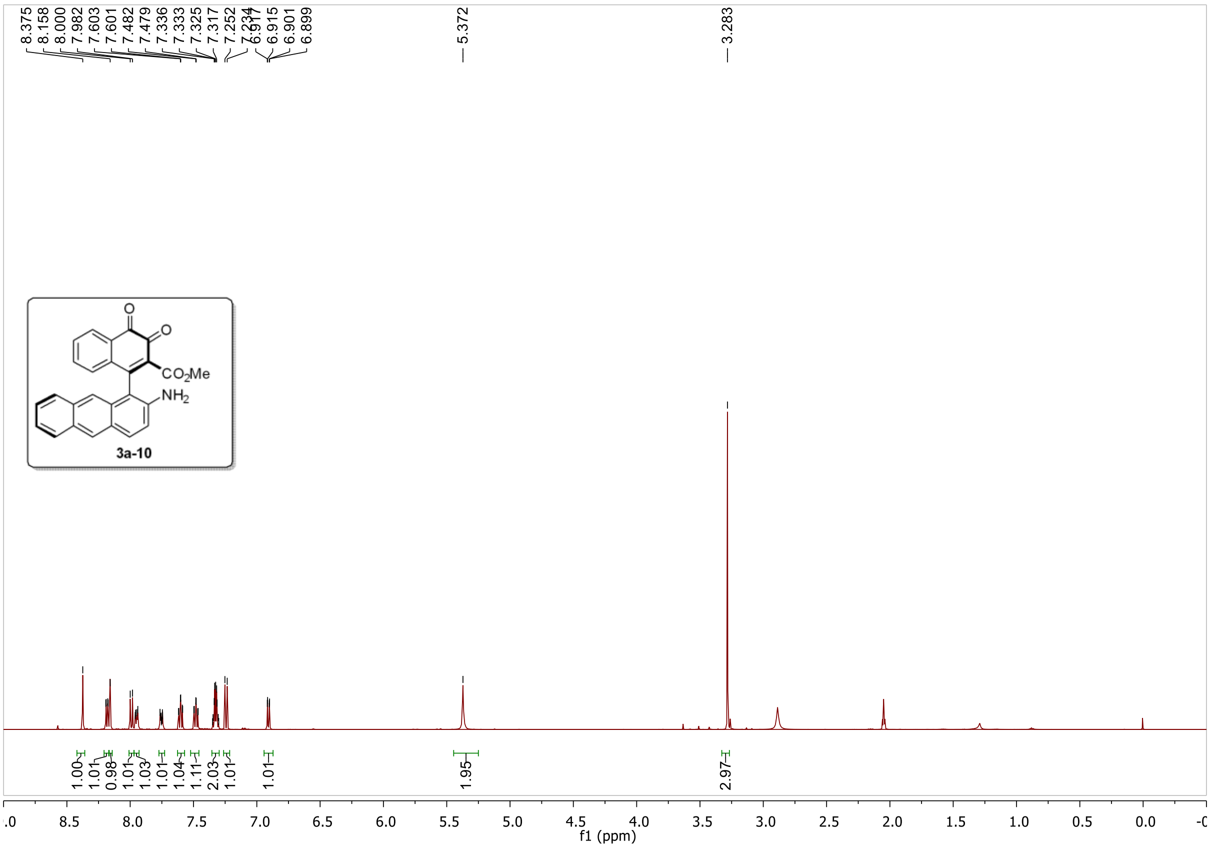

Supplementary Figure 195. ^{13}C NMR of **3a-10**.

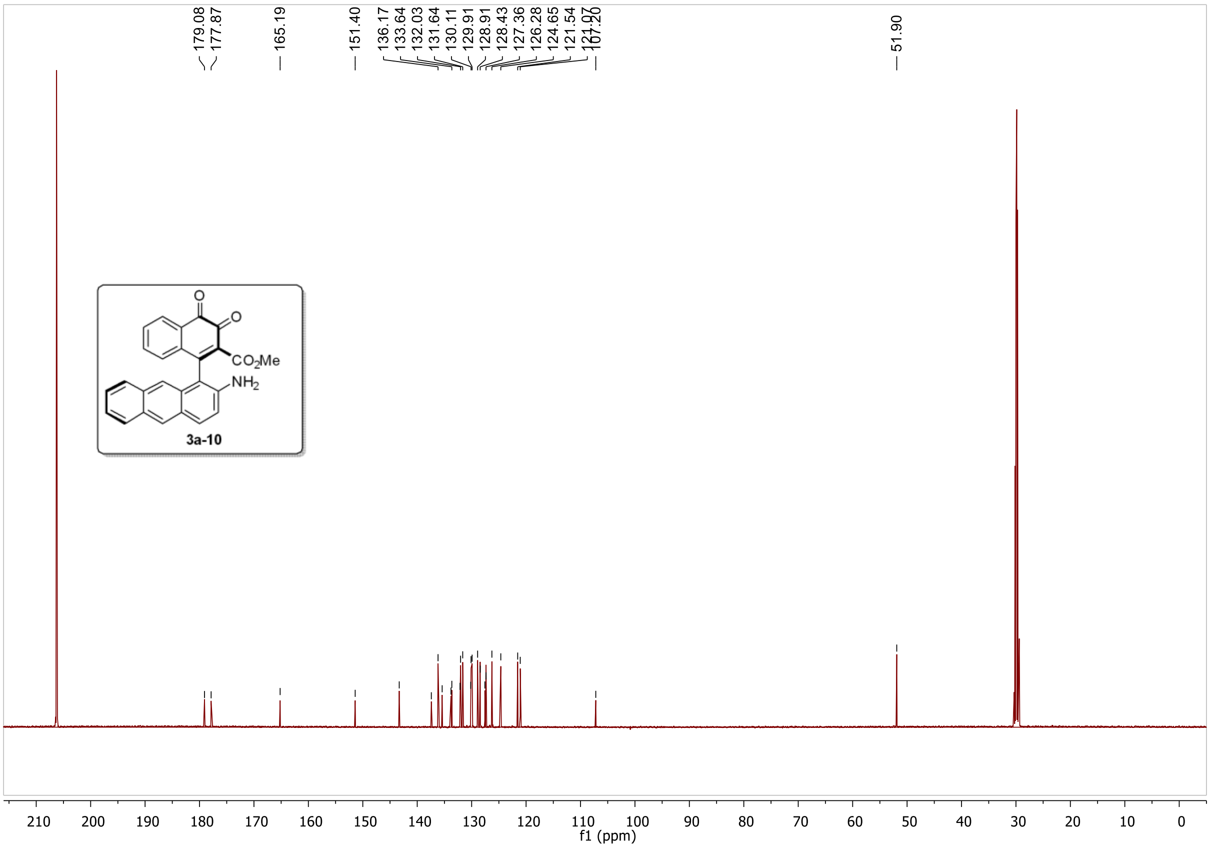

Supplementary Figure 196. ^{1}H NMR of **3a-11**.

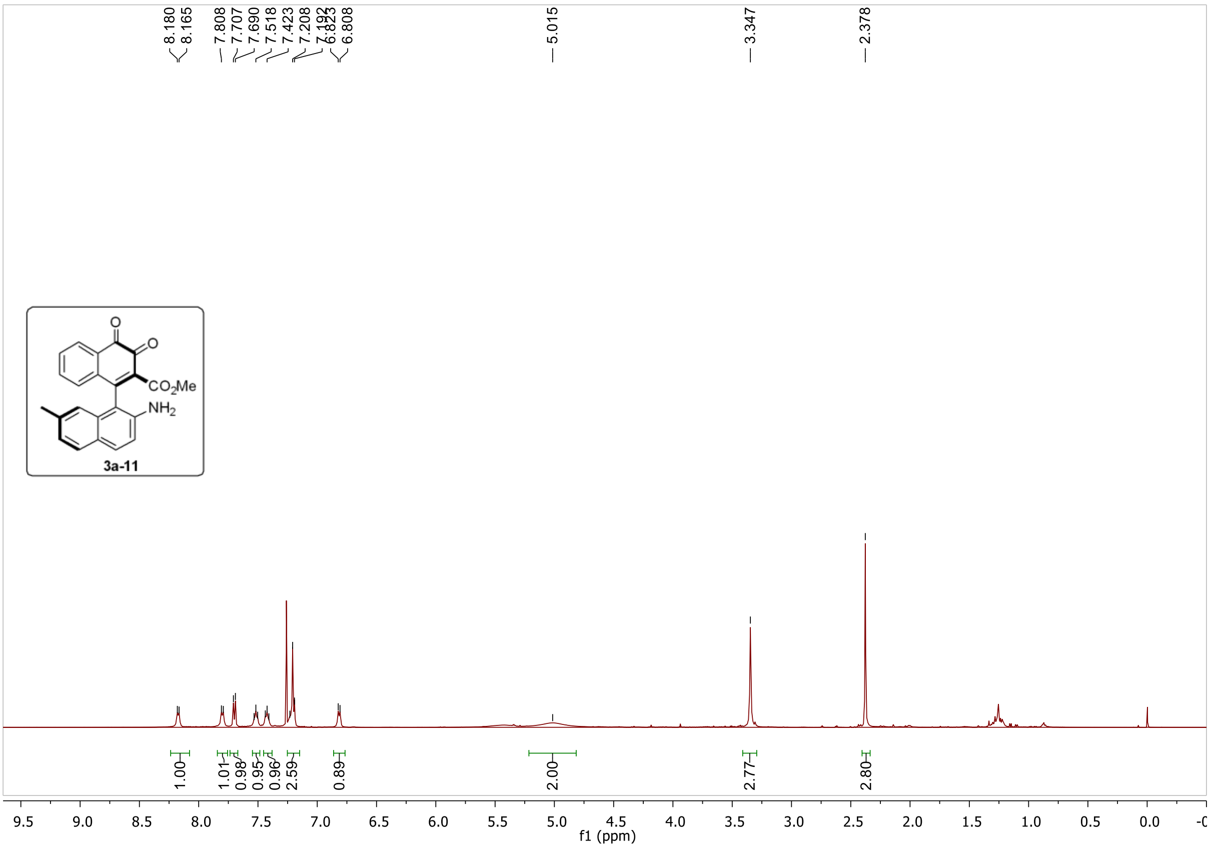

Supplementary Figure 197. ^{13}C NMR of **3a-11**.

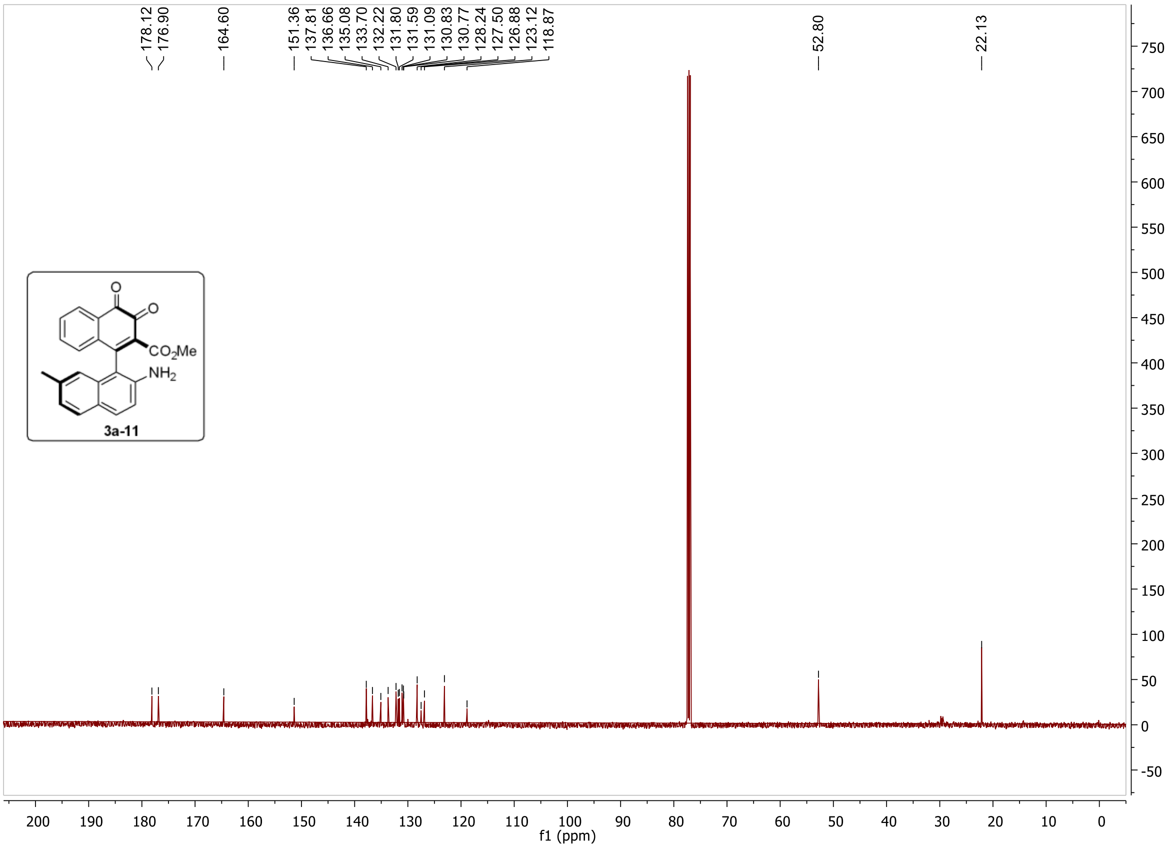

Supplementary Figure 198. ^{1}H NMR of **3a-12**.

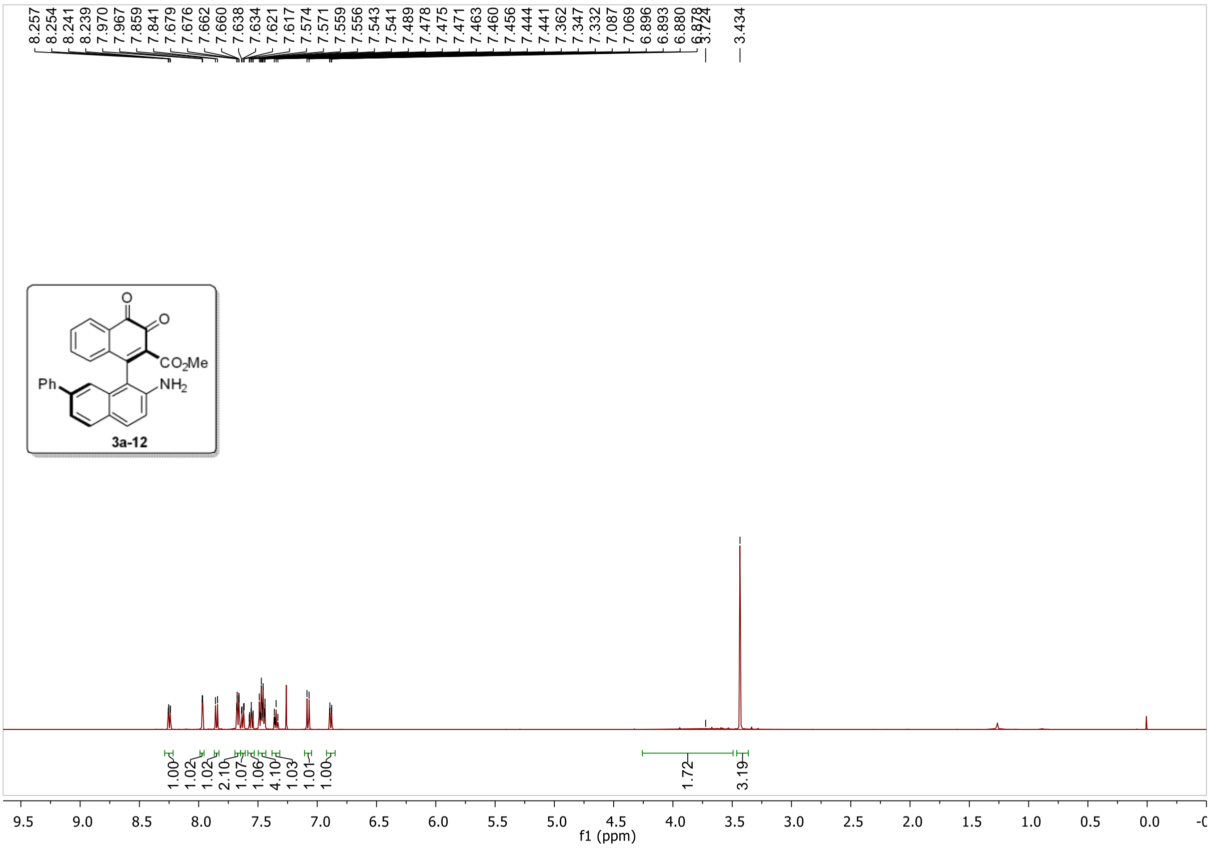

Supplementary Figure 199. ^{13}C NMR of **3a-12**.

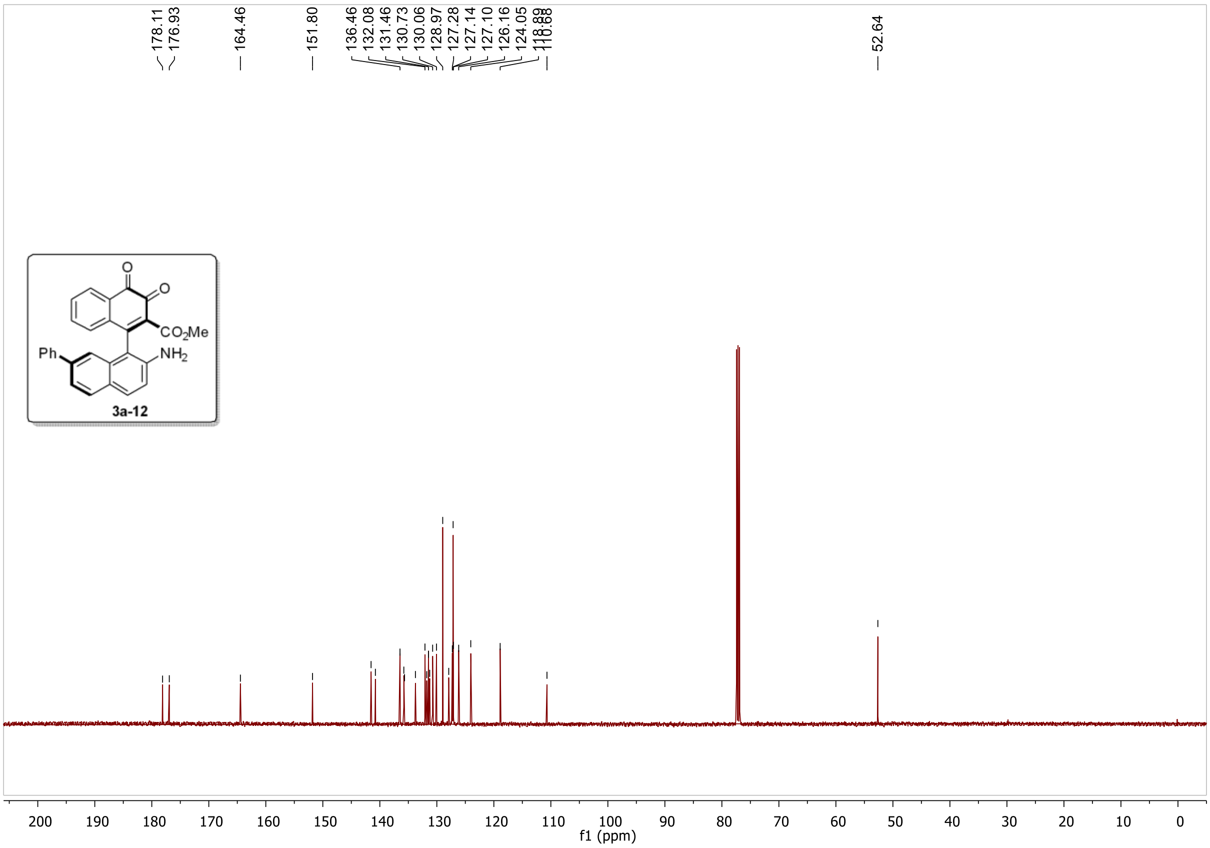

Supplementary Figure 200. ^{1}H NMR of **3a-13**.

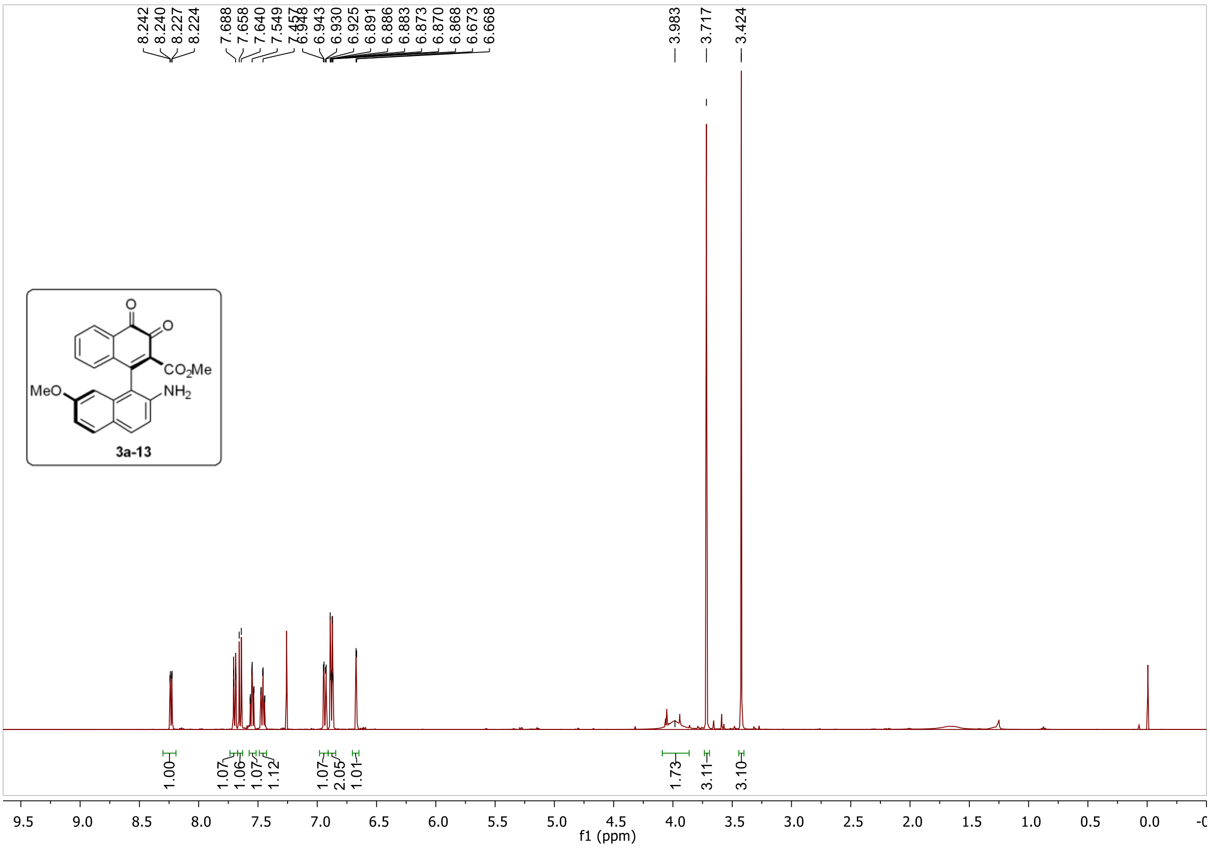

Supplementary Figure 201. ^{13}C NMR of **3a-13**.

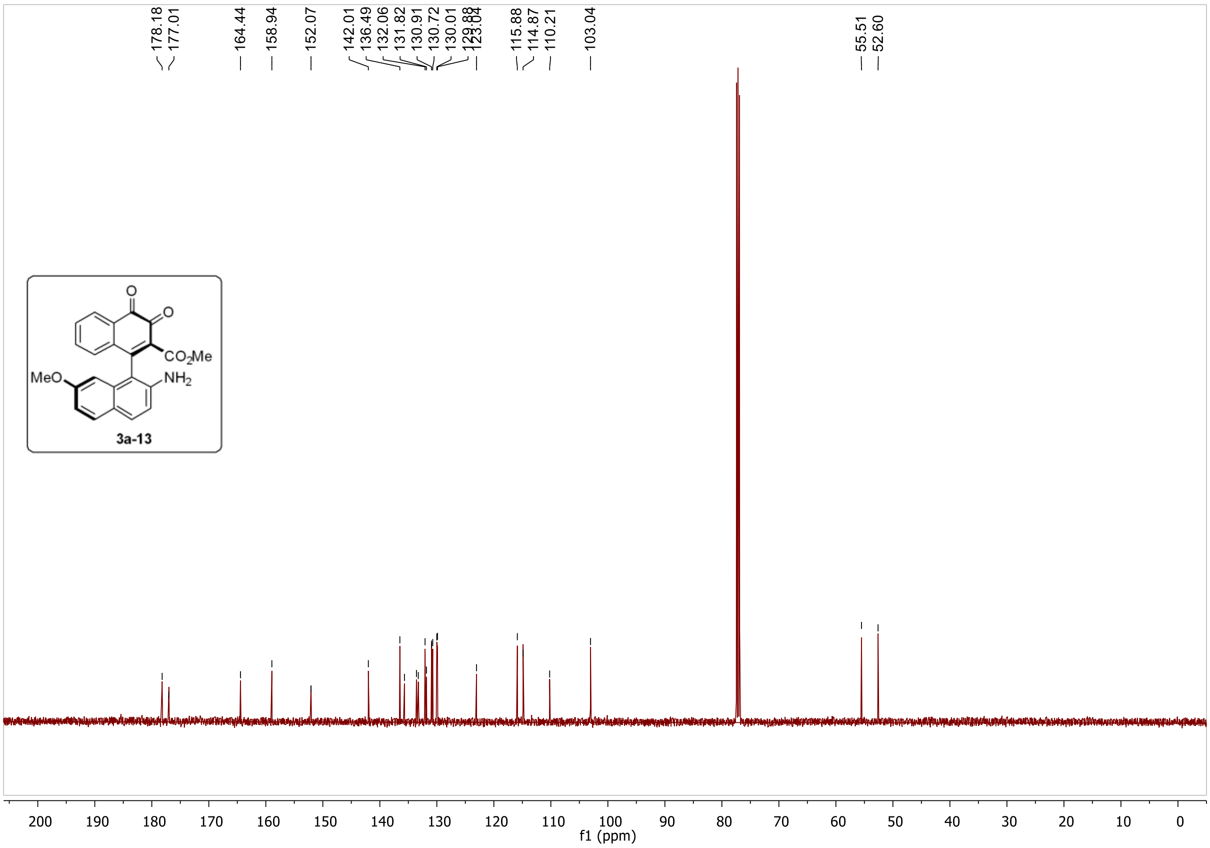

Supplementary Figure 202. ^{1}H NMR of **3a-14**.

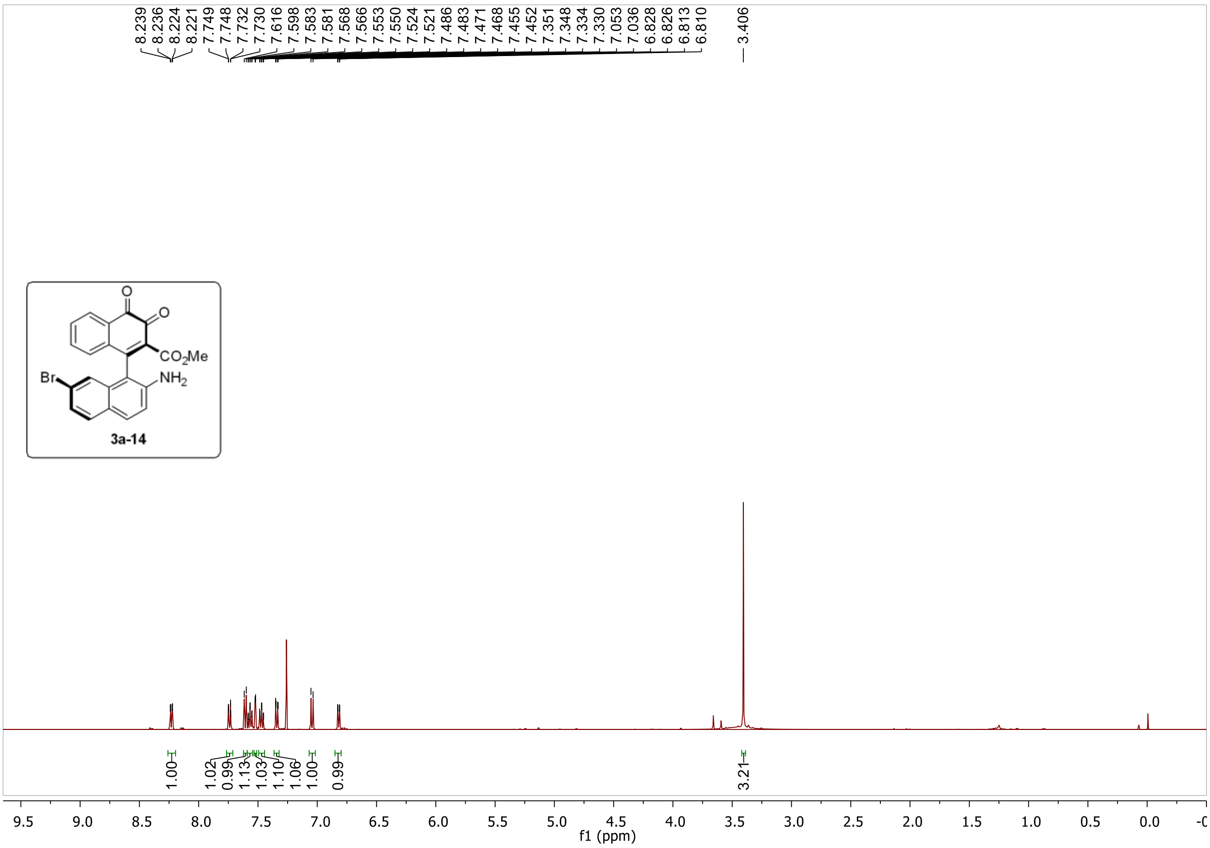

Supplementary Figure 203. ^{13}C NMR of **3a-14**.

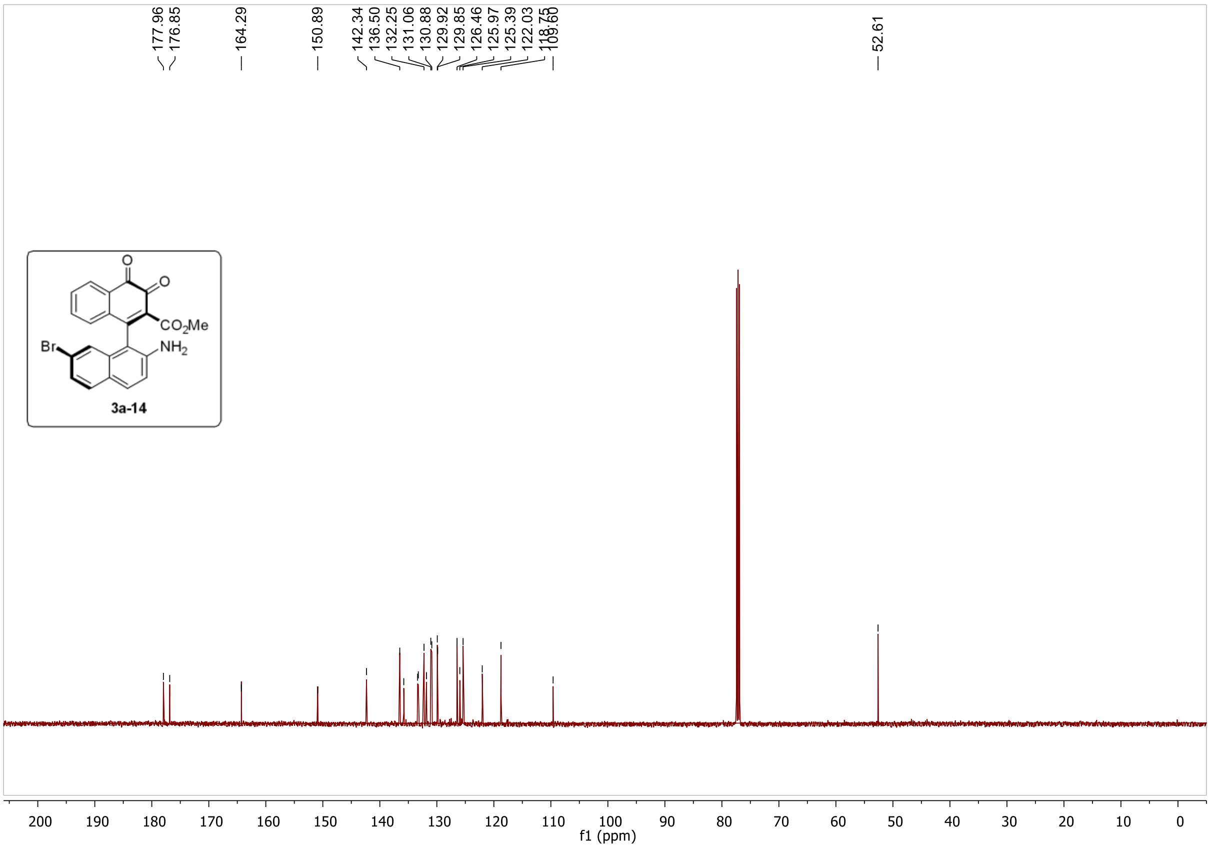

Supplementary Figure 204. ^{1}H NMR of **3b-1**.

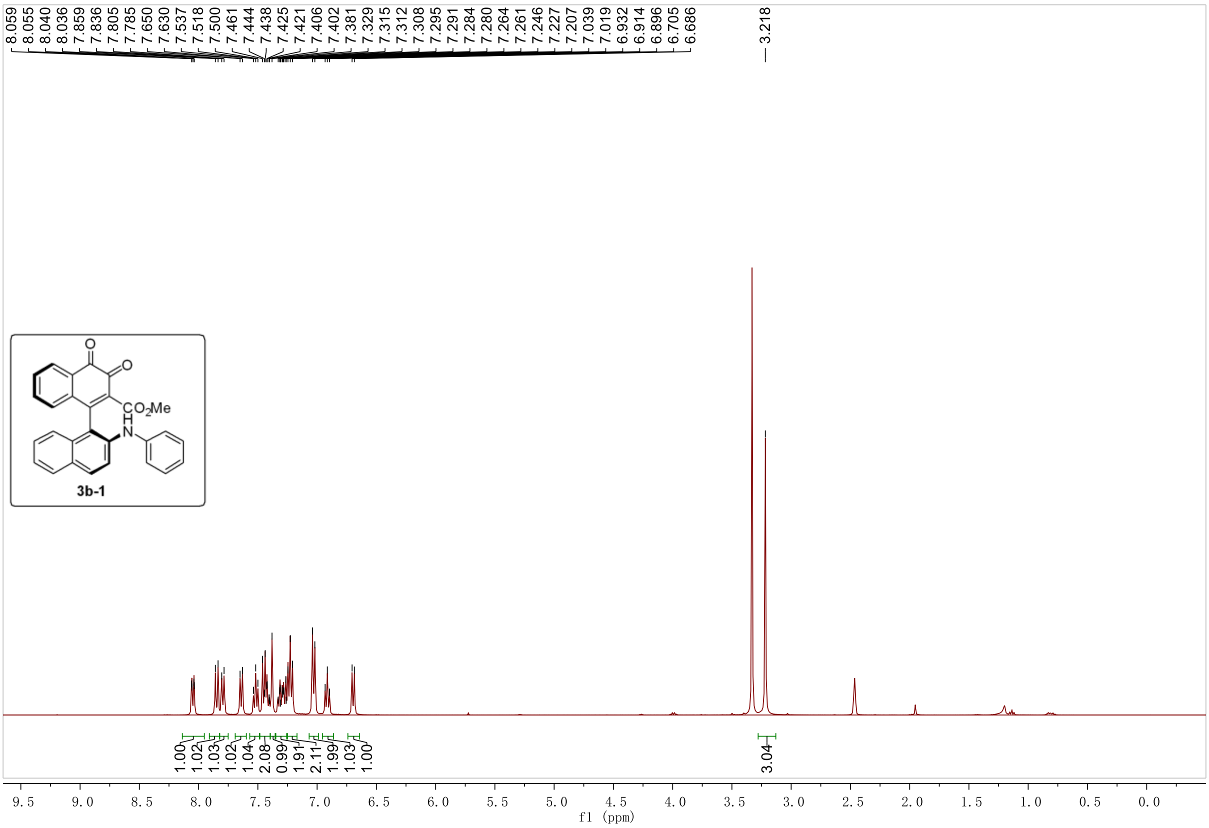

Supplementary Figure 205. ^{13}C NMR of **3b-1**.

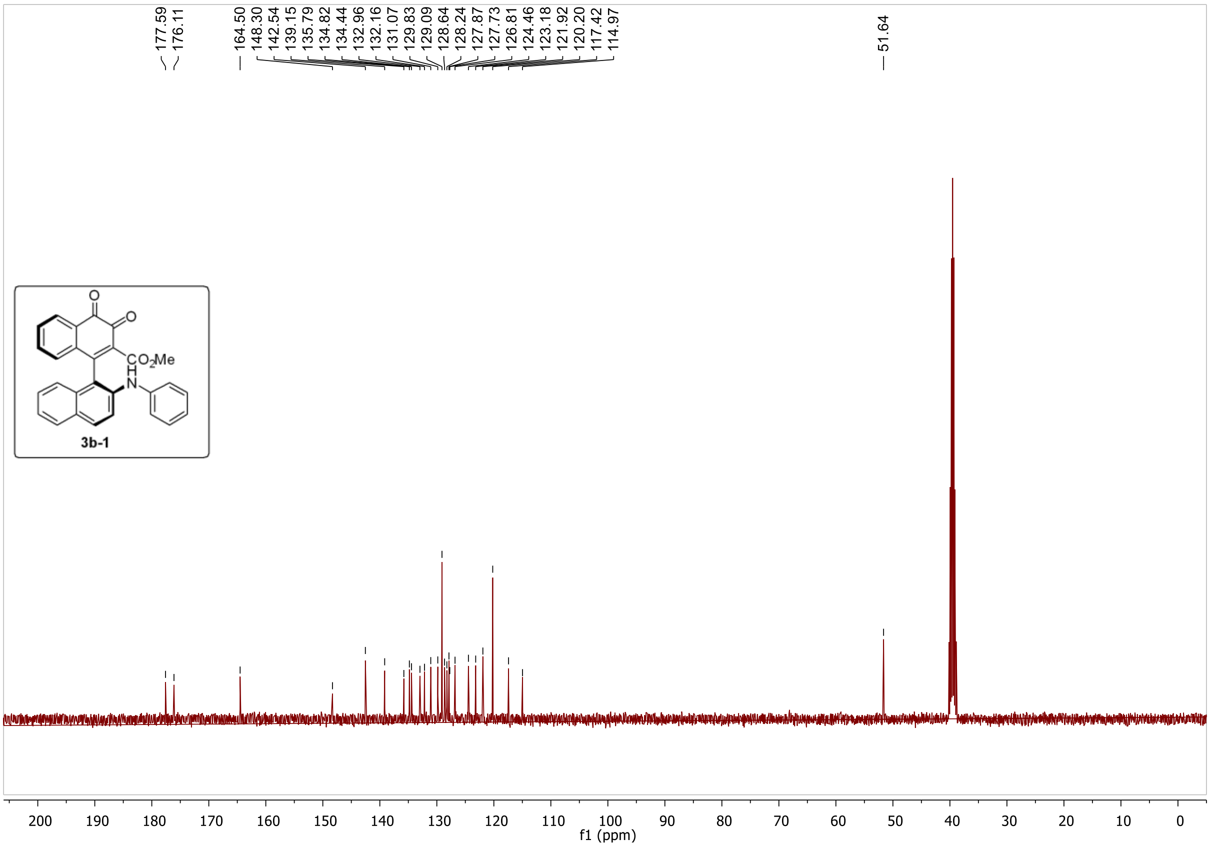

Supplementary Figure 206. ^{1}H NMR of **3b-2**.

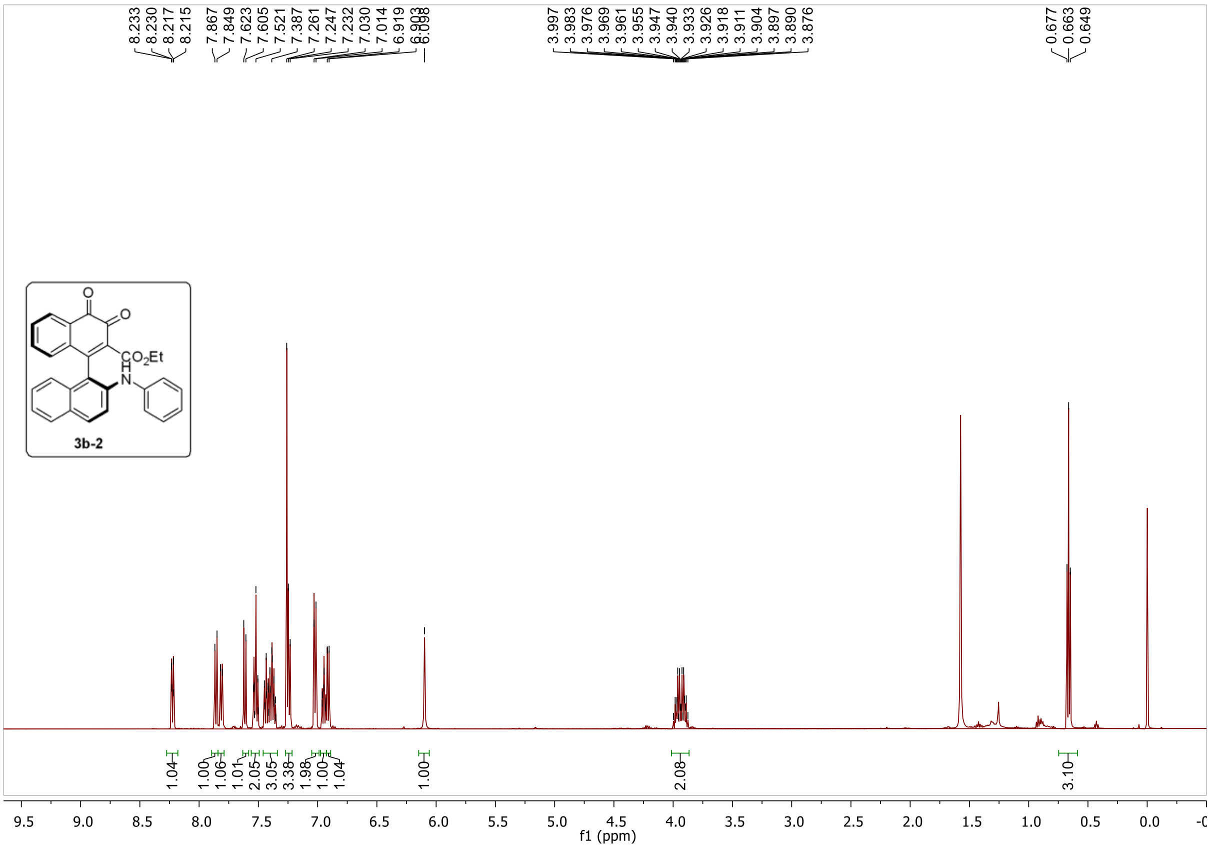

Supplementary Figure 207. ^{13}C NMR of **3b-2**.

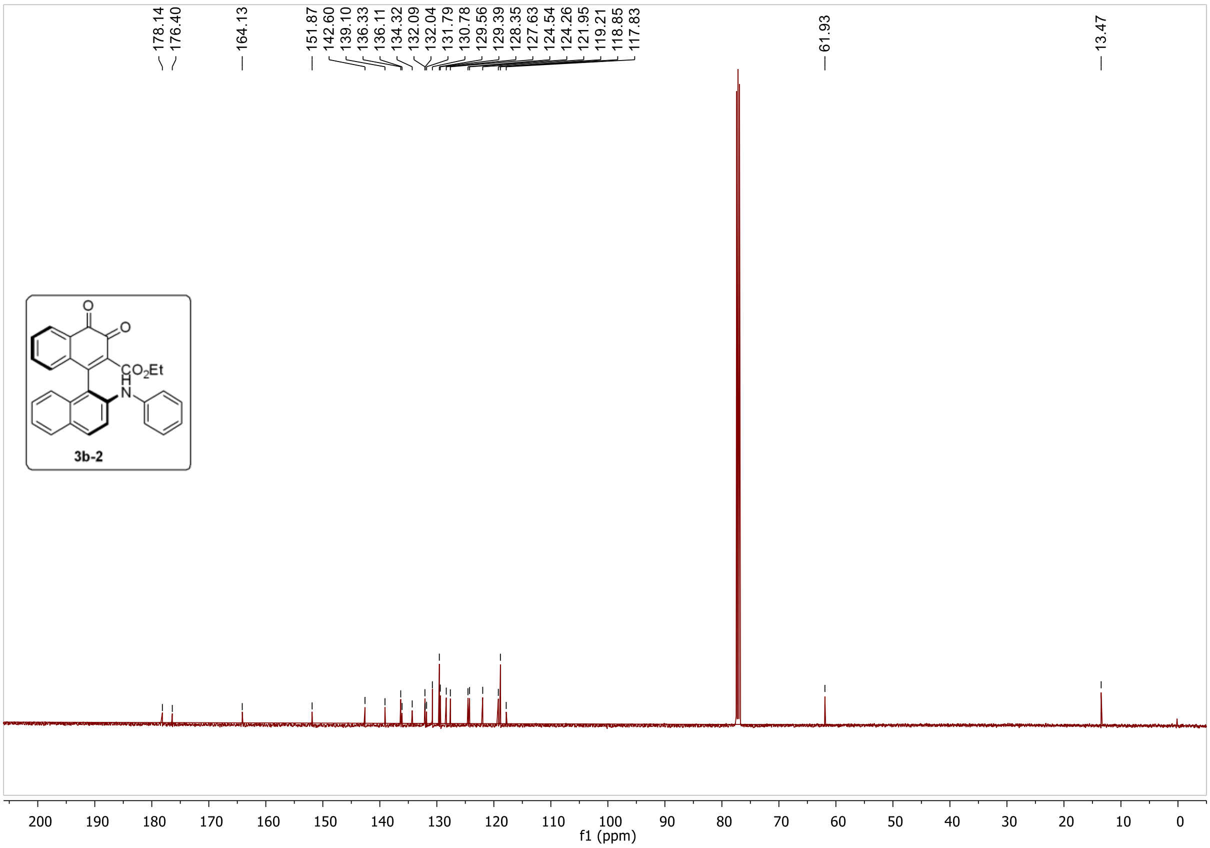

Supplementary Figure 208. ^{1}H NMR of **3b-3**.

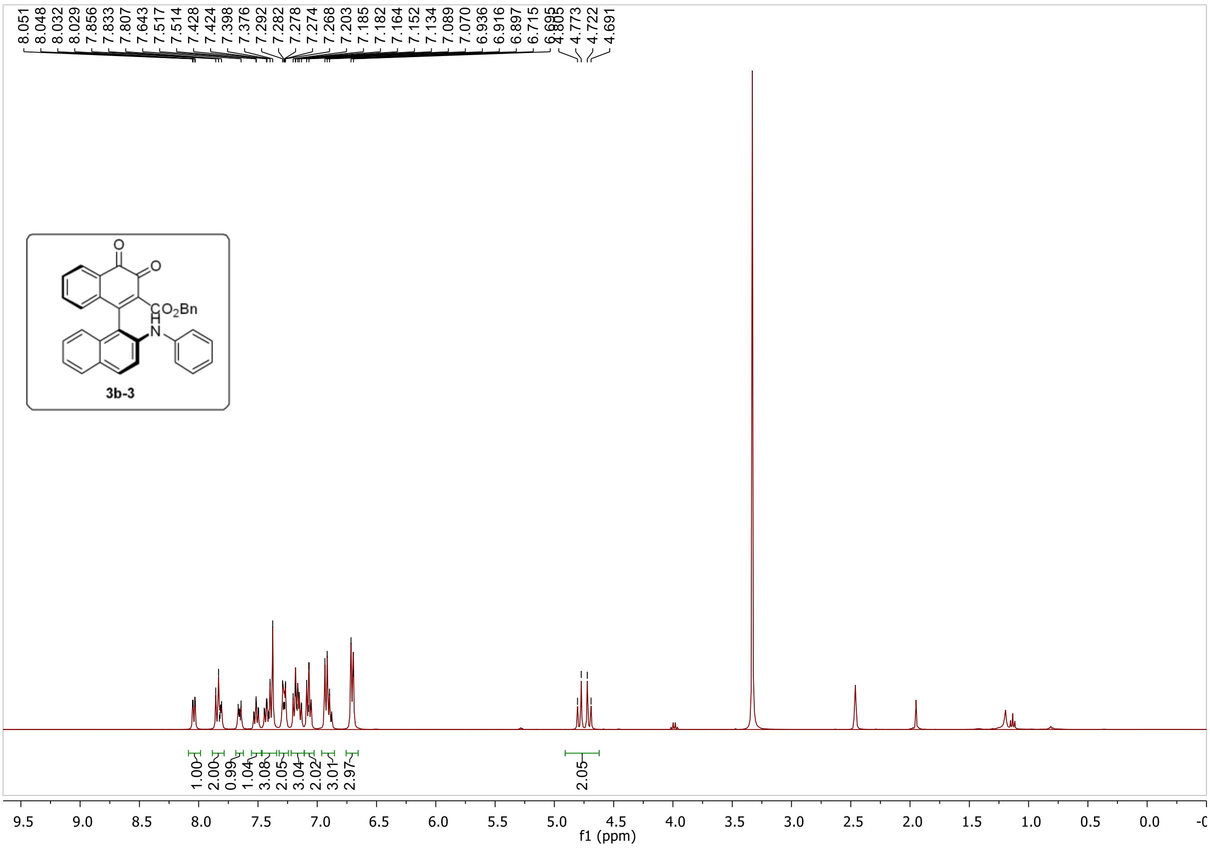

Supplementary Figure 209. ^{13}C NMR of **3b-3**.

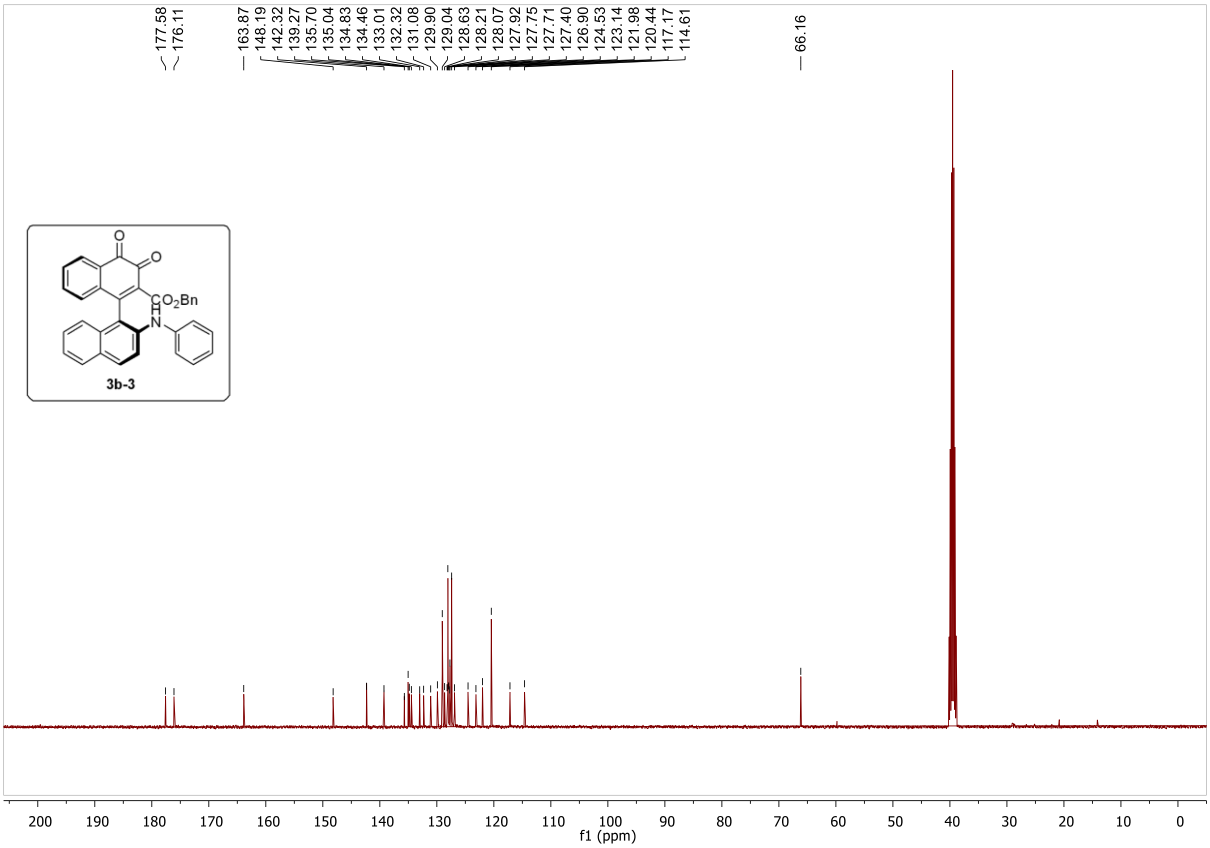

Supplementary Figure 210. ¹H NMR of **3b-4**.

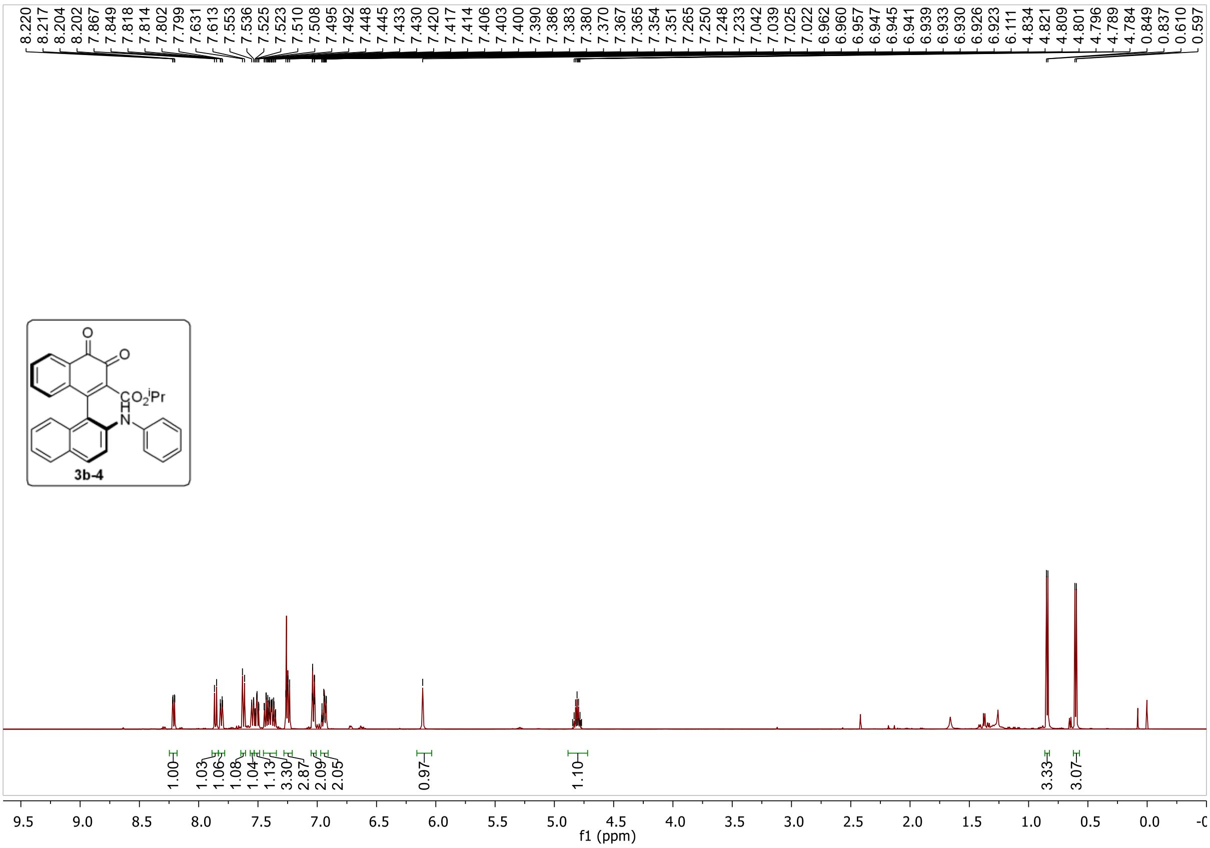

Supplementary Figure 211. ¹³C NMR of **3b-4**.

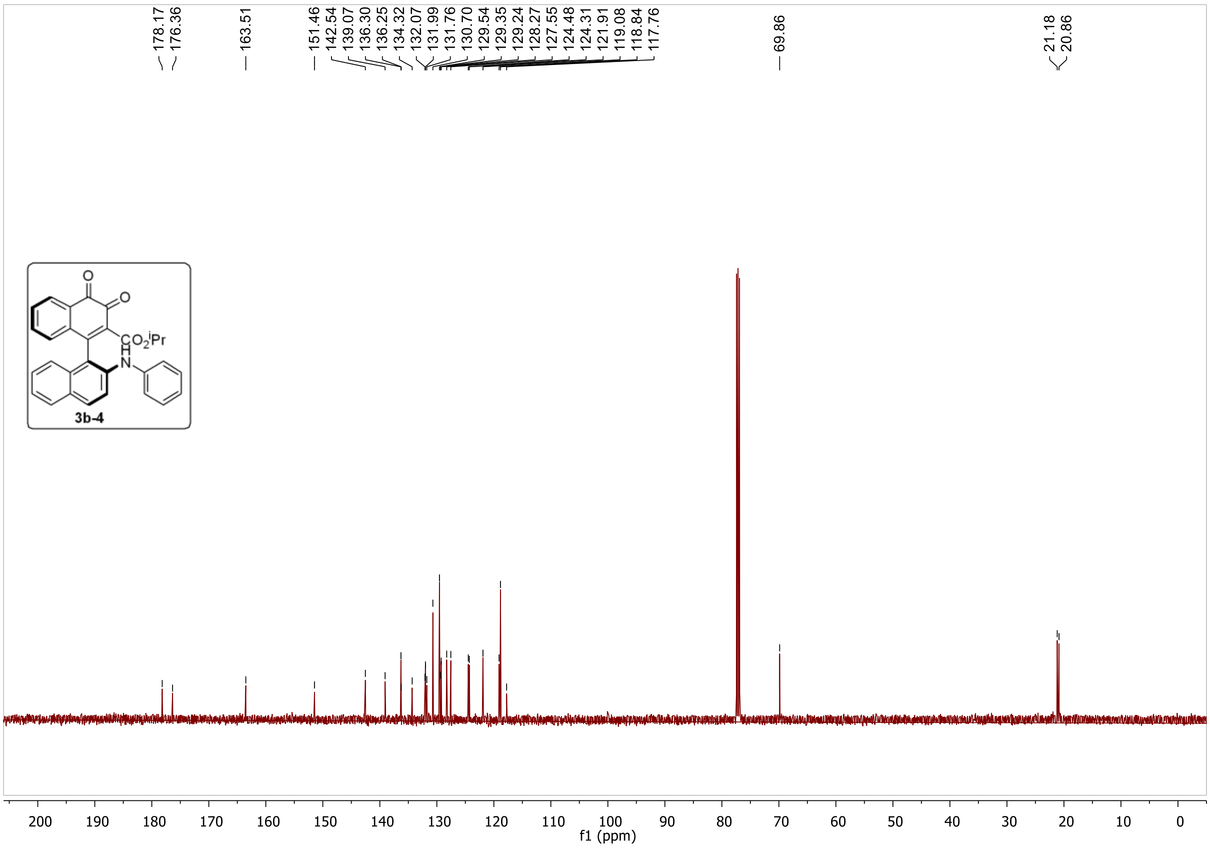

Supplementary Figure 212. ^{1}H NMR of **3b-5**.

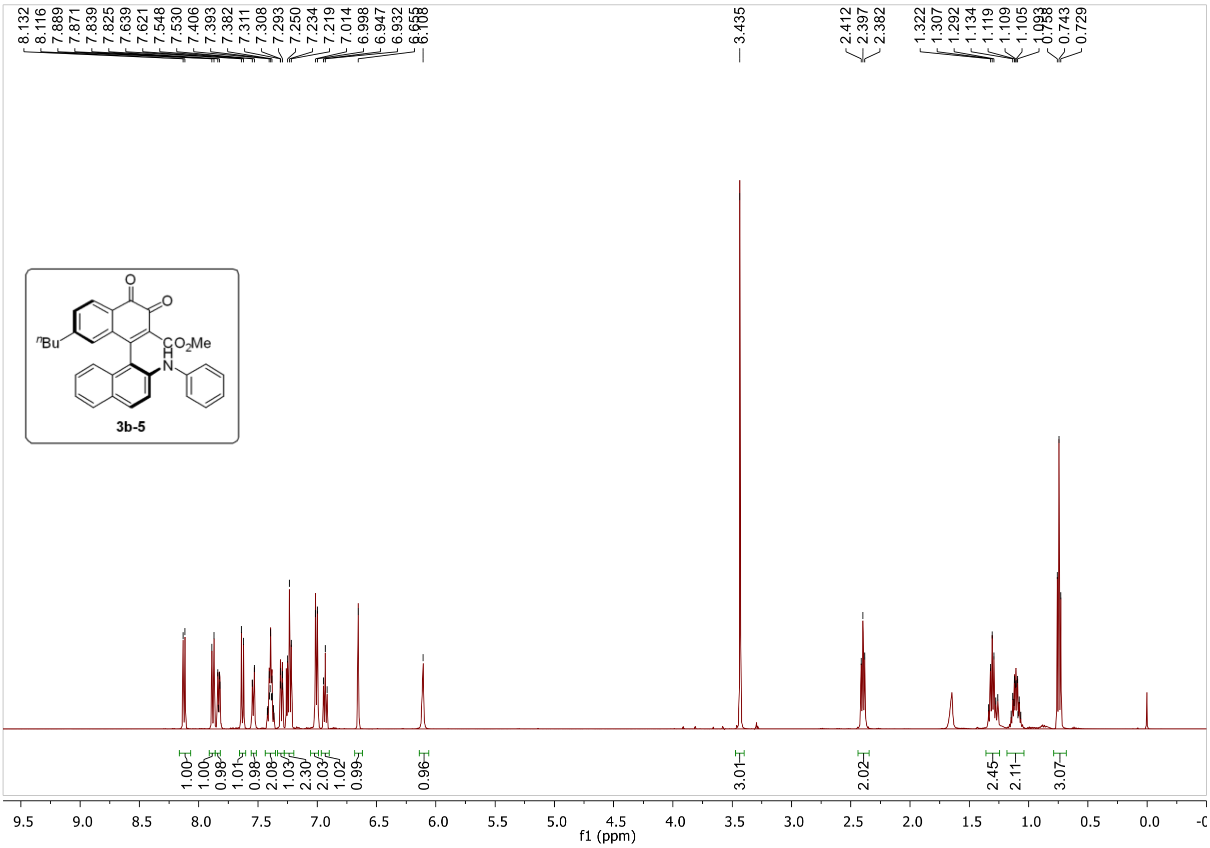

Supplementary Figure 213. ^{13}C NMR of **3b-5**.

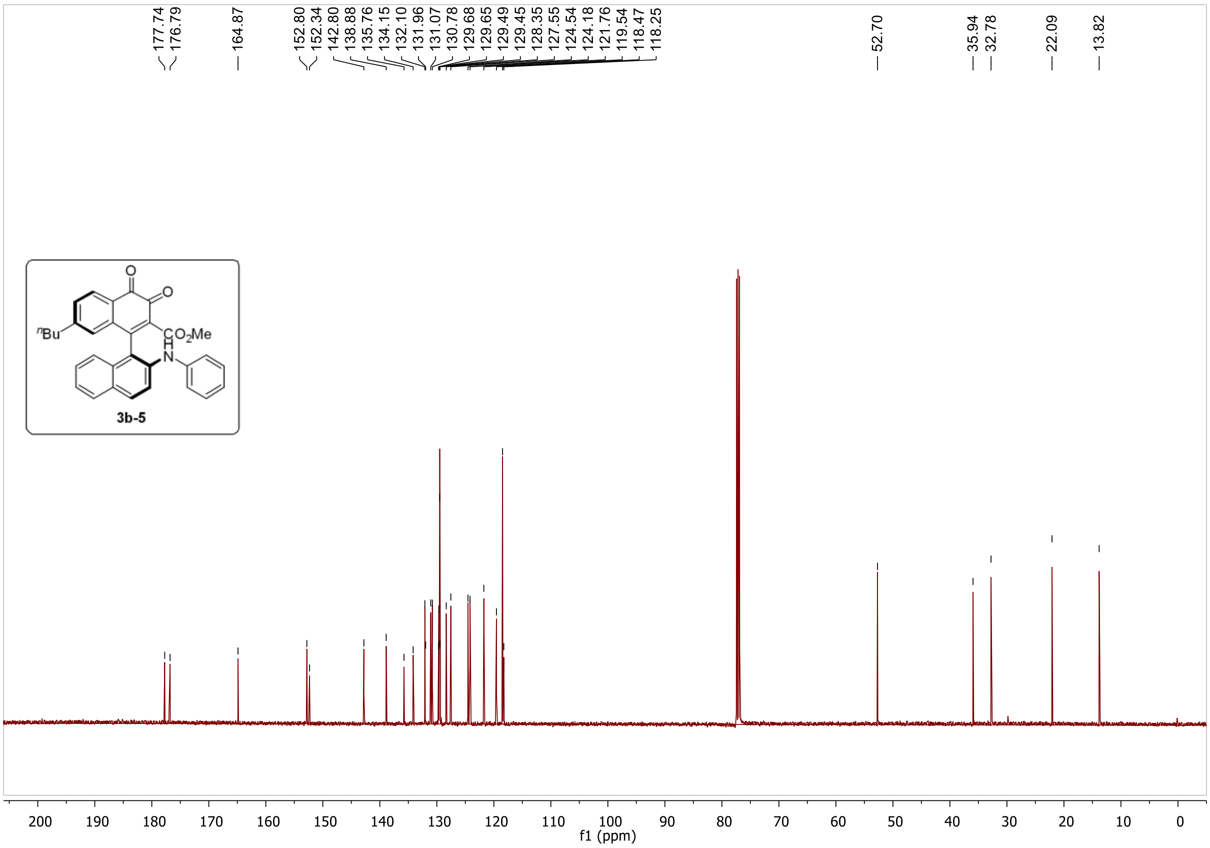

Supplementary Figure 214. ^1H NMR of **3b-6**.

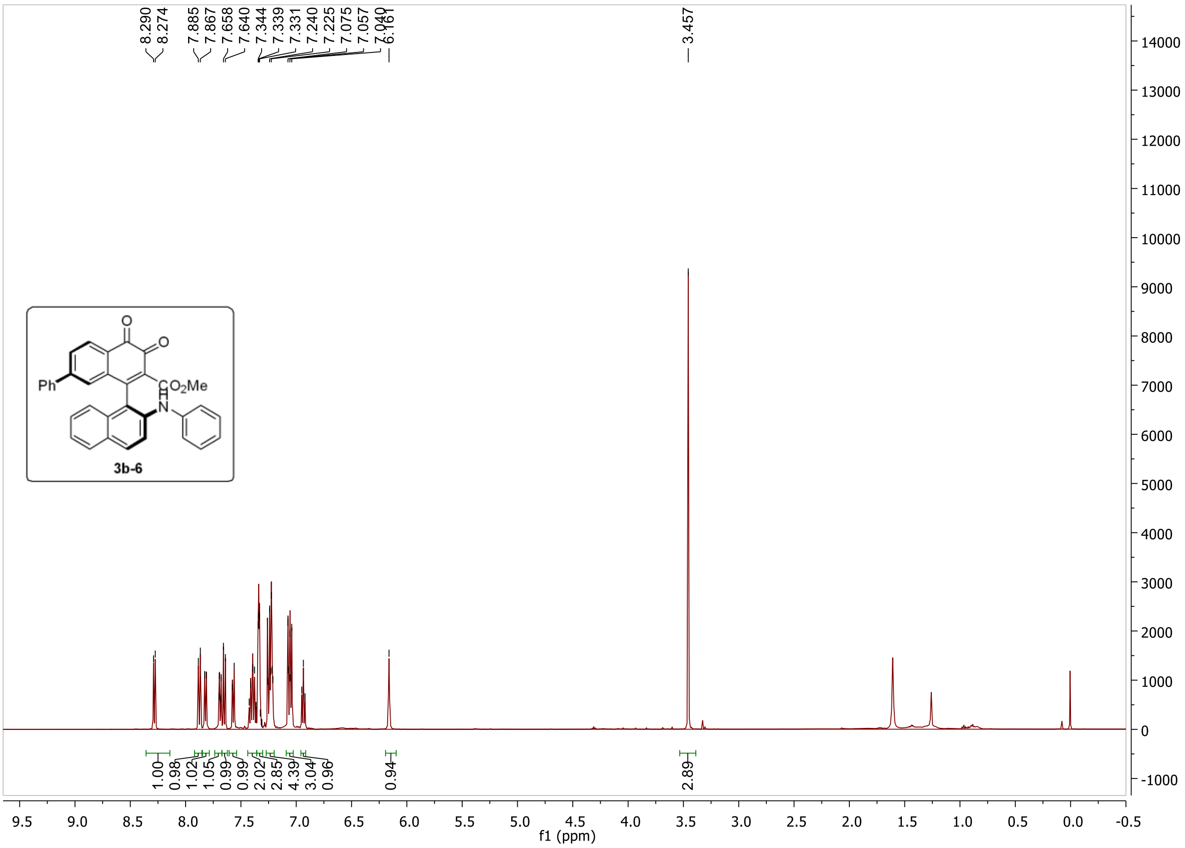

Supplementary Figure 215. ^13C NMR of **3b-6**.

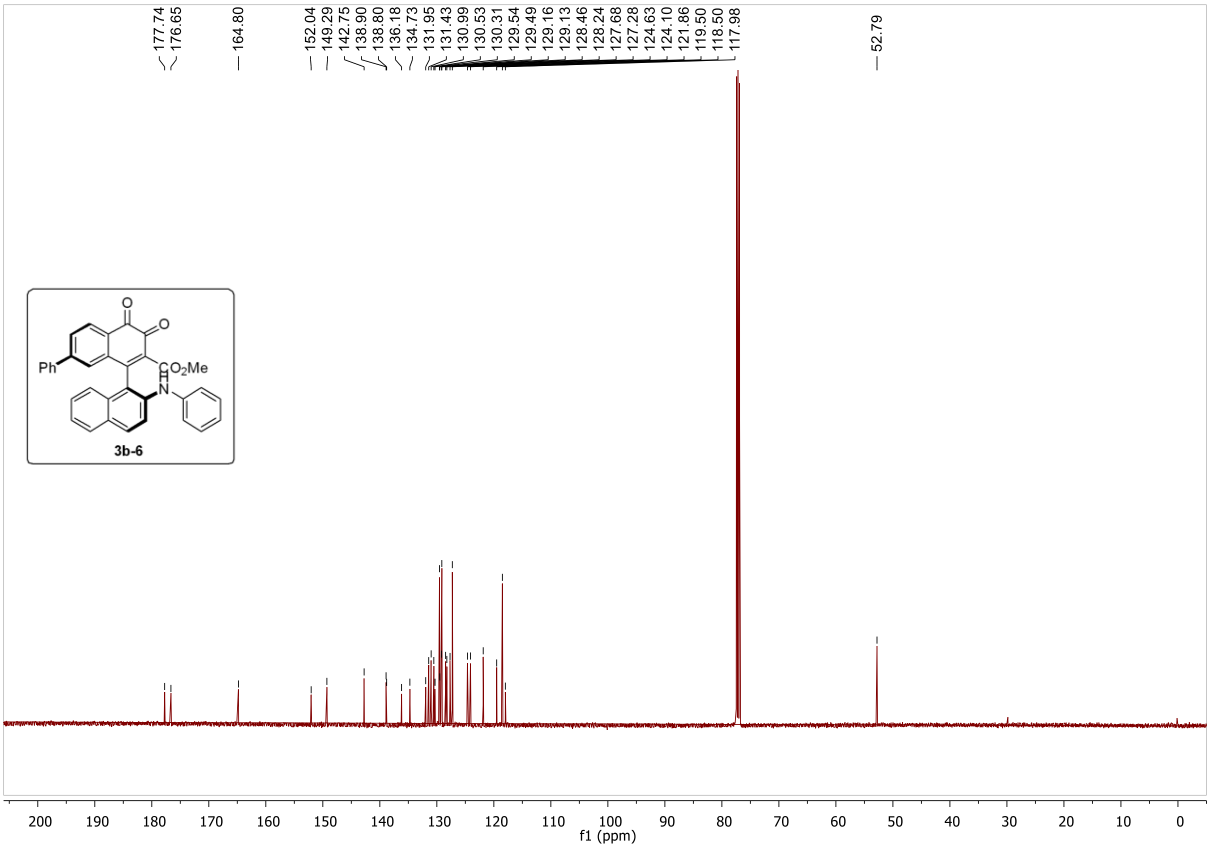

Supplementary Figure 216. ^{1}H NMR of **3b-7**.

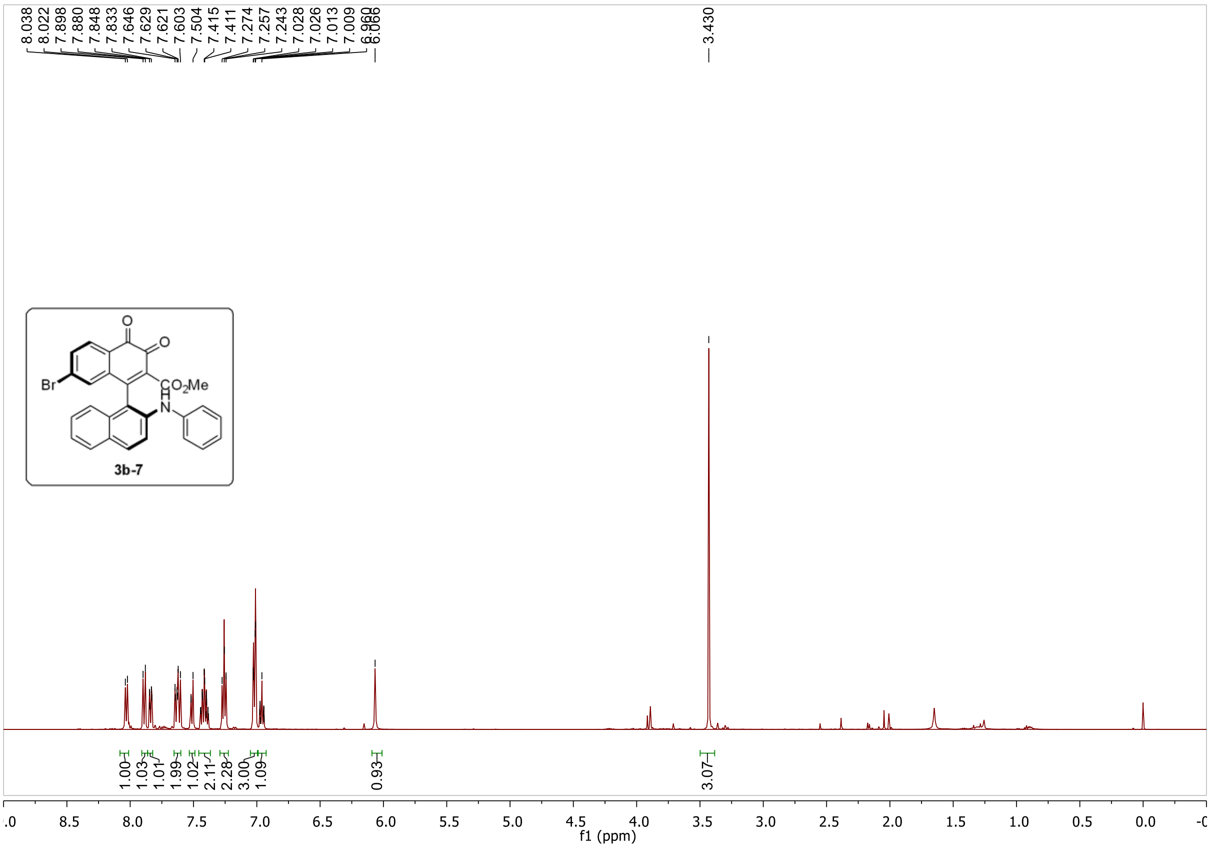

Supplementary Figure 217. ^{13}C NMR of **3b-7**.

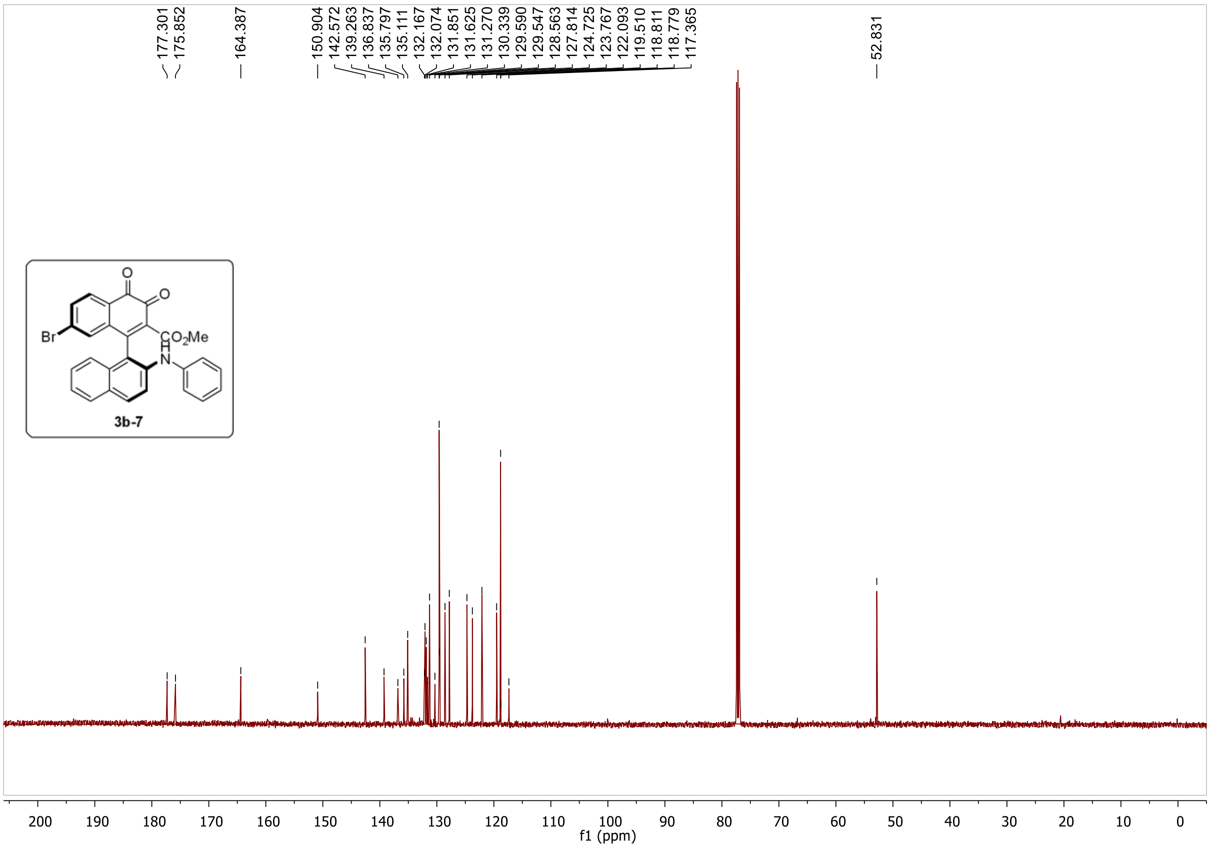

Supplementary Figure 218. ^{1}H NMR of **3b-8**.

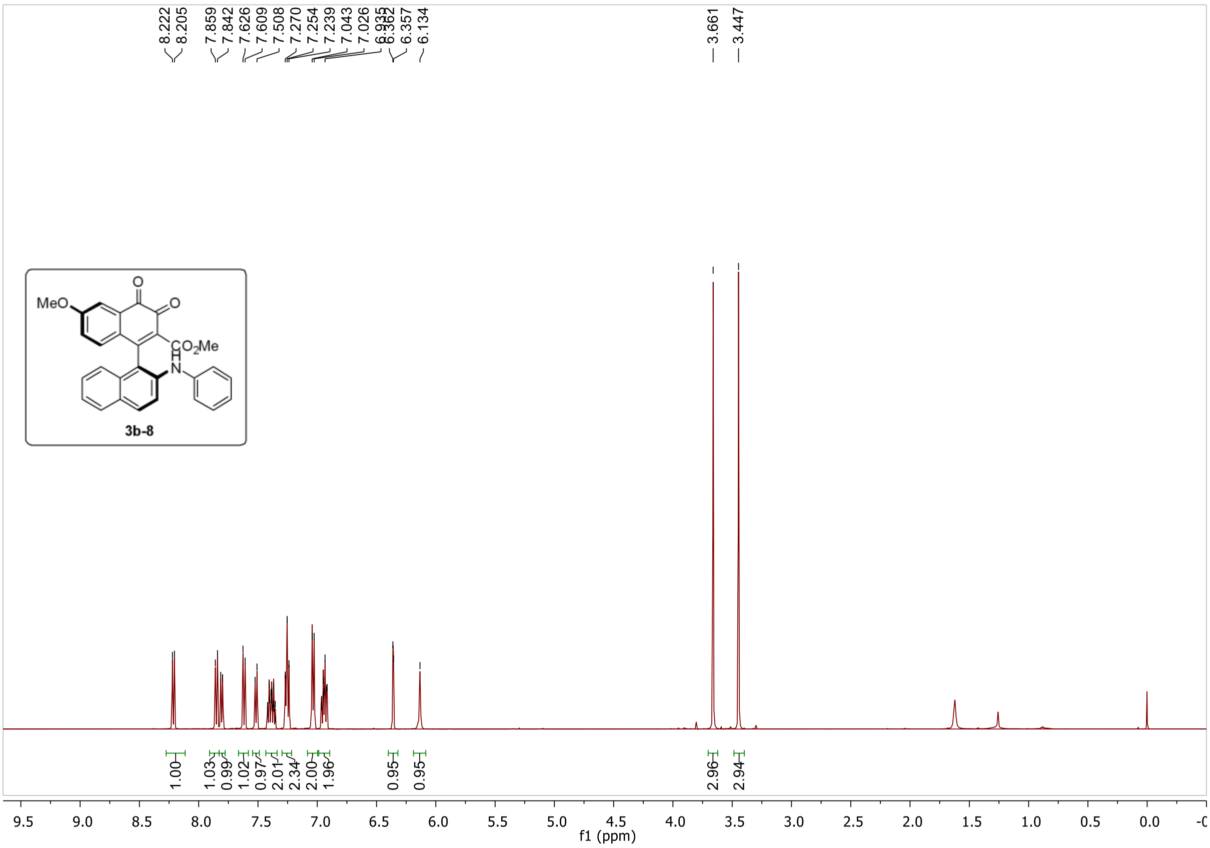

Supplementary Figure 219. ^{13}C NMR of **3b-8**.

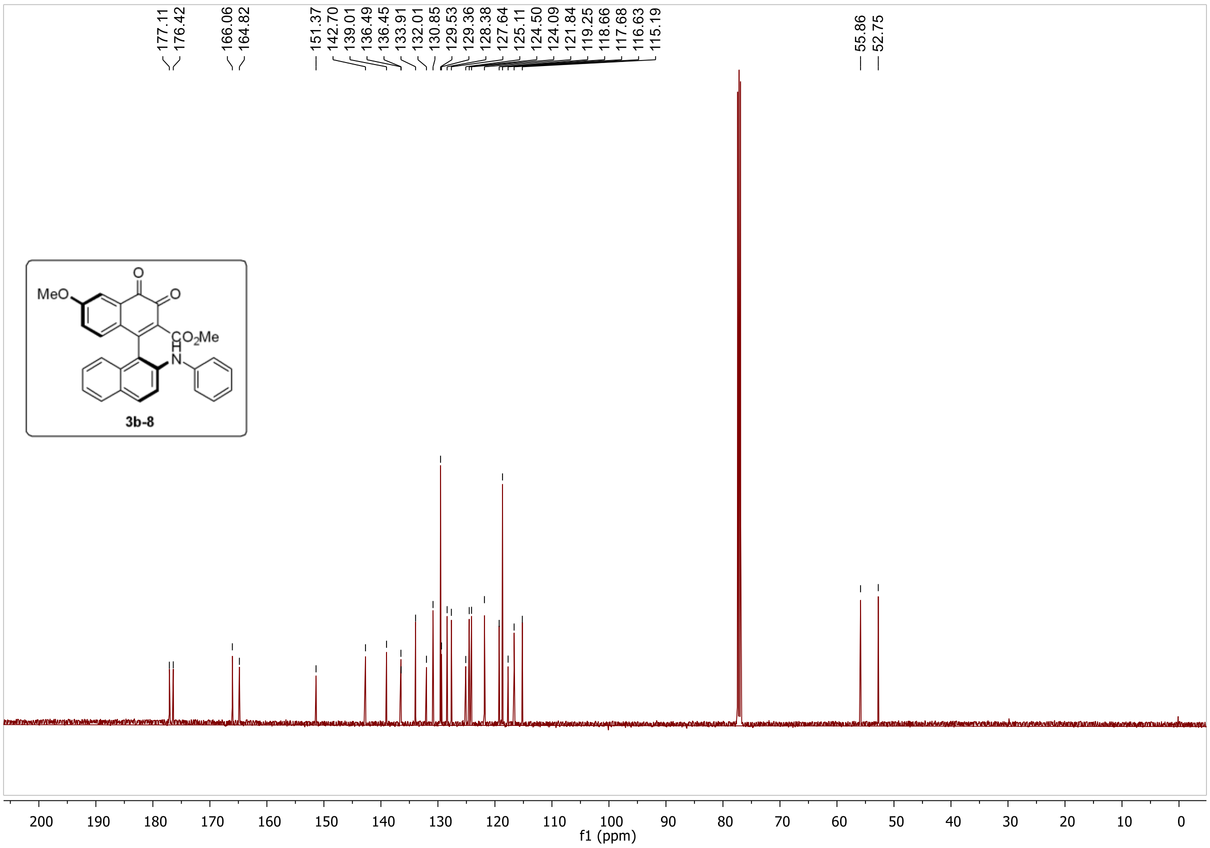

Supplementary Figure 220. ^{1}H NMR of **3b-9**.

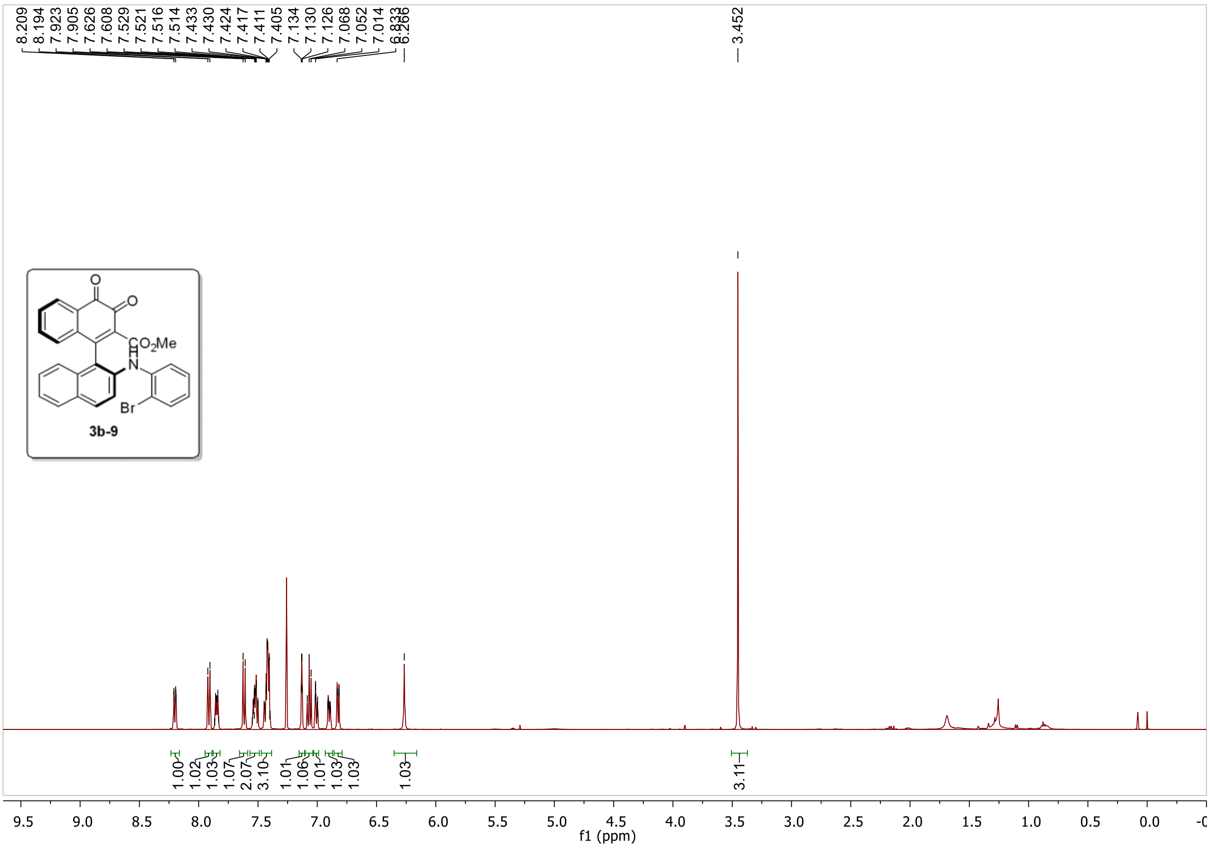

Supplementary Figure 221. ^{13}C NMR of **3b-9**.

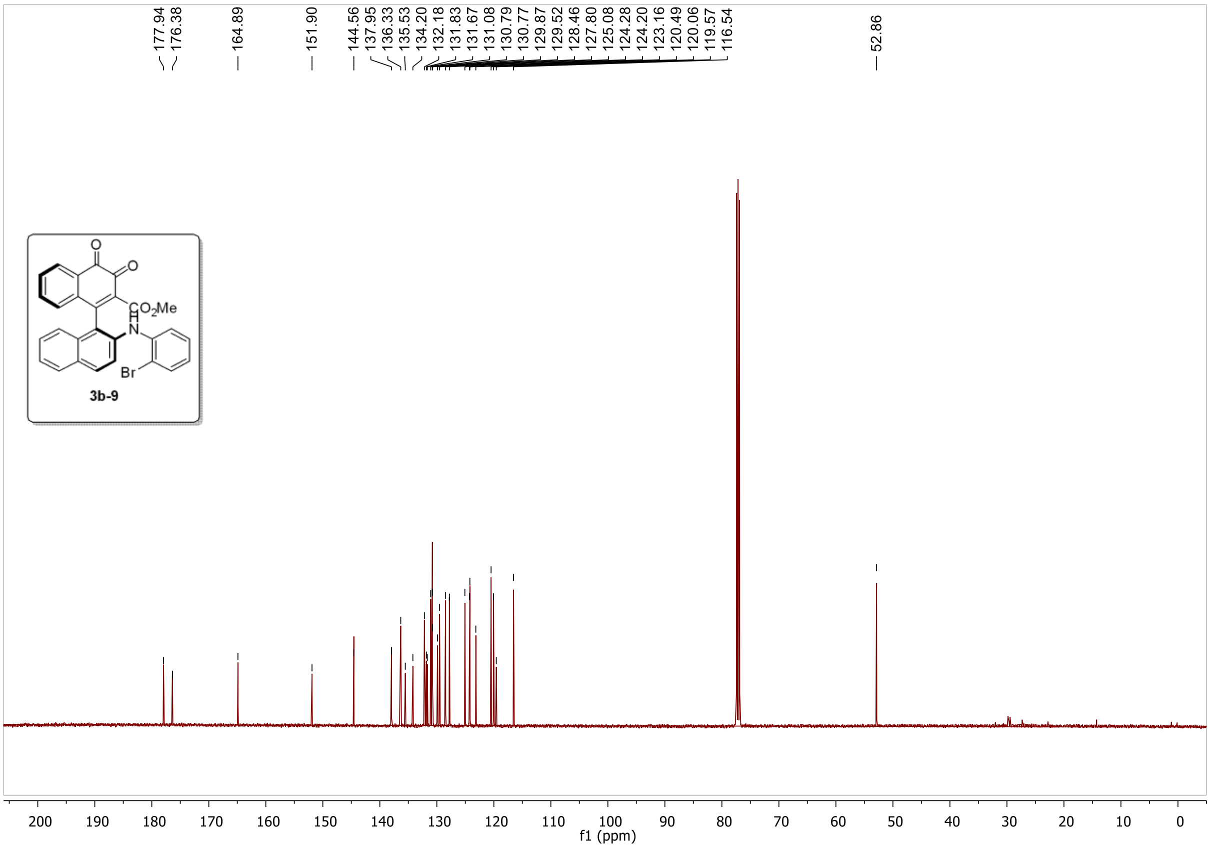

Supplementary Figure 222. ^{1}H NMR of **3b-10**.

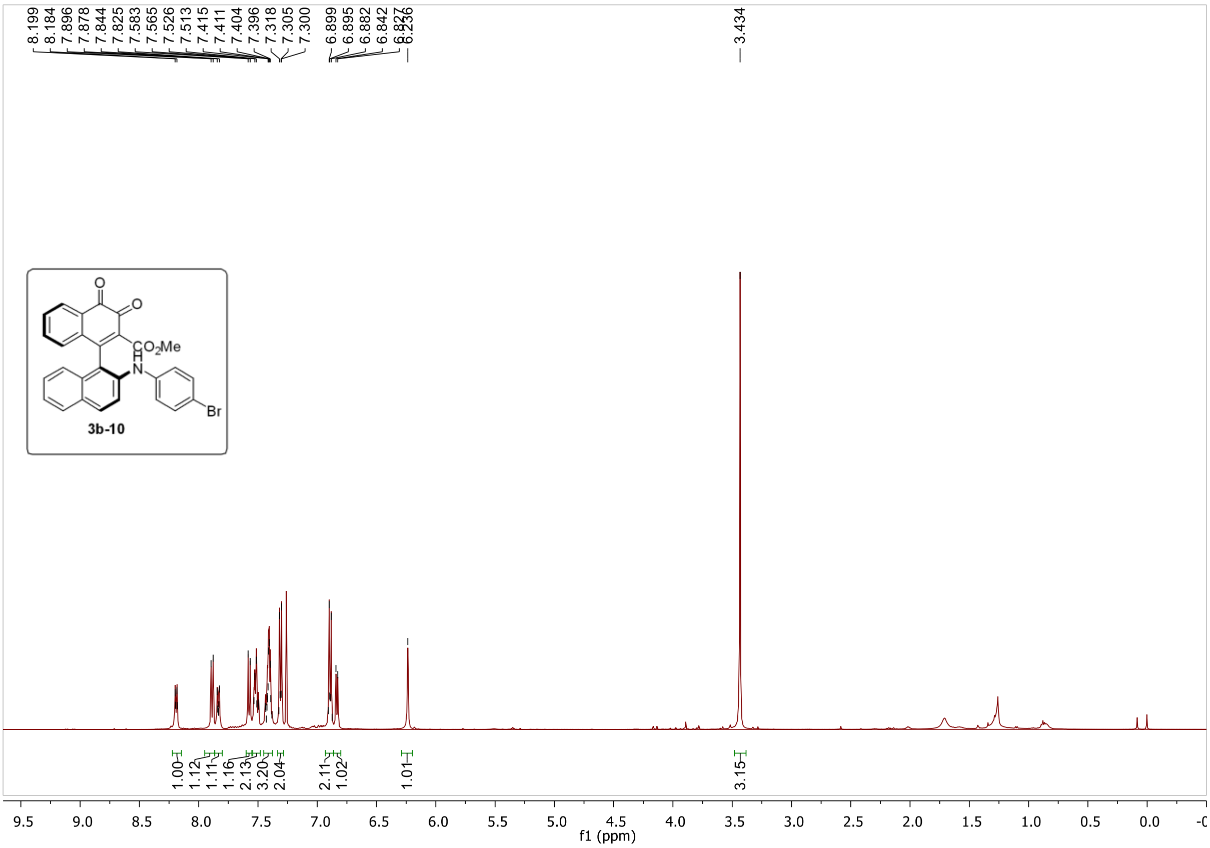

Supplementary Figure 223. ^{13}C NMR of **3b-10**.

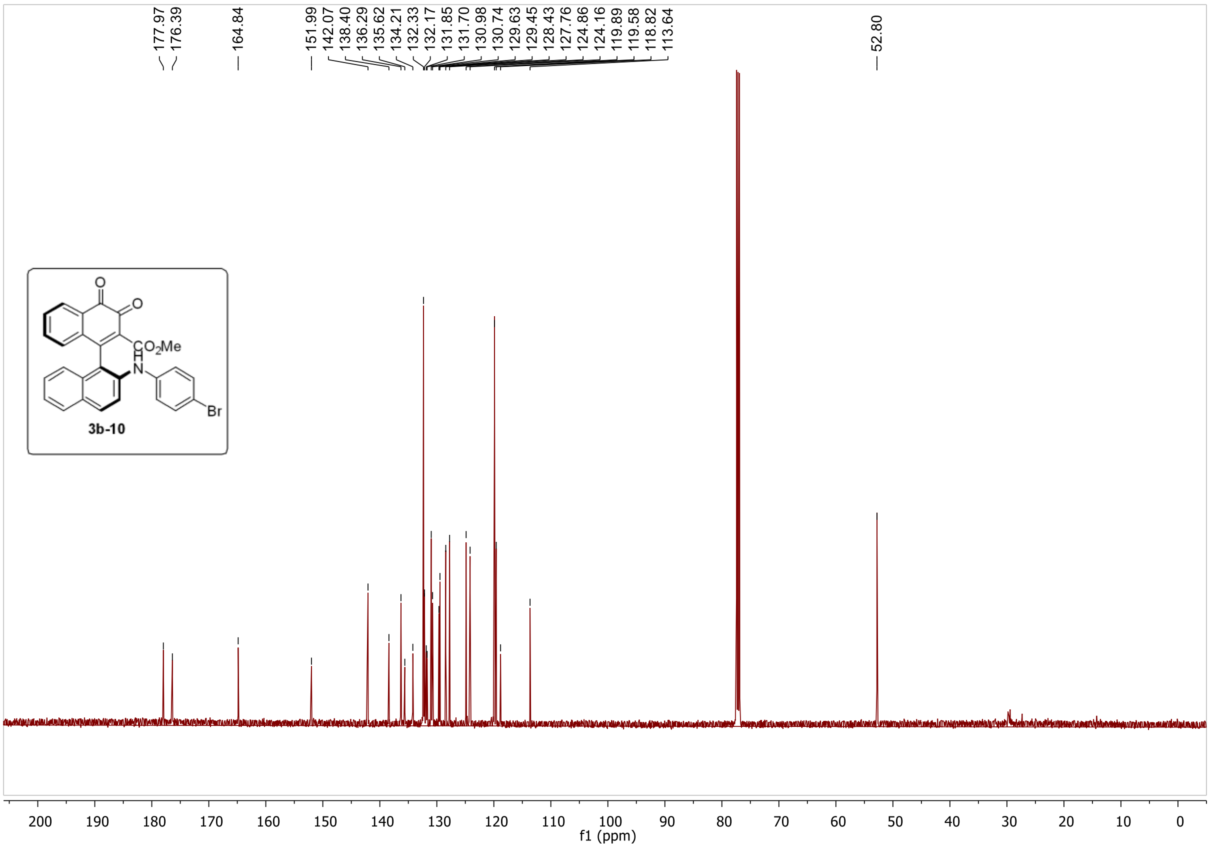

Supplementary Figure 224. ^{1}H NMR of **3b-11**.

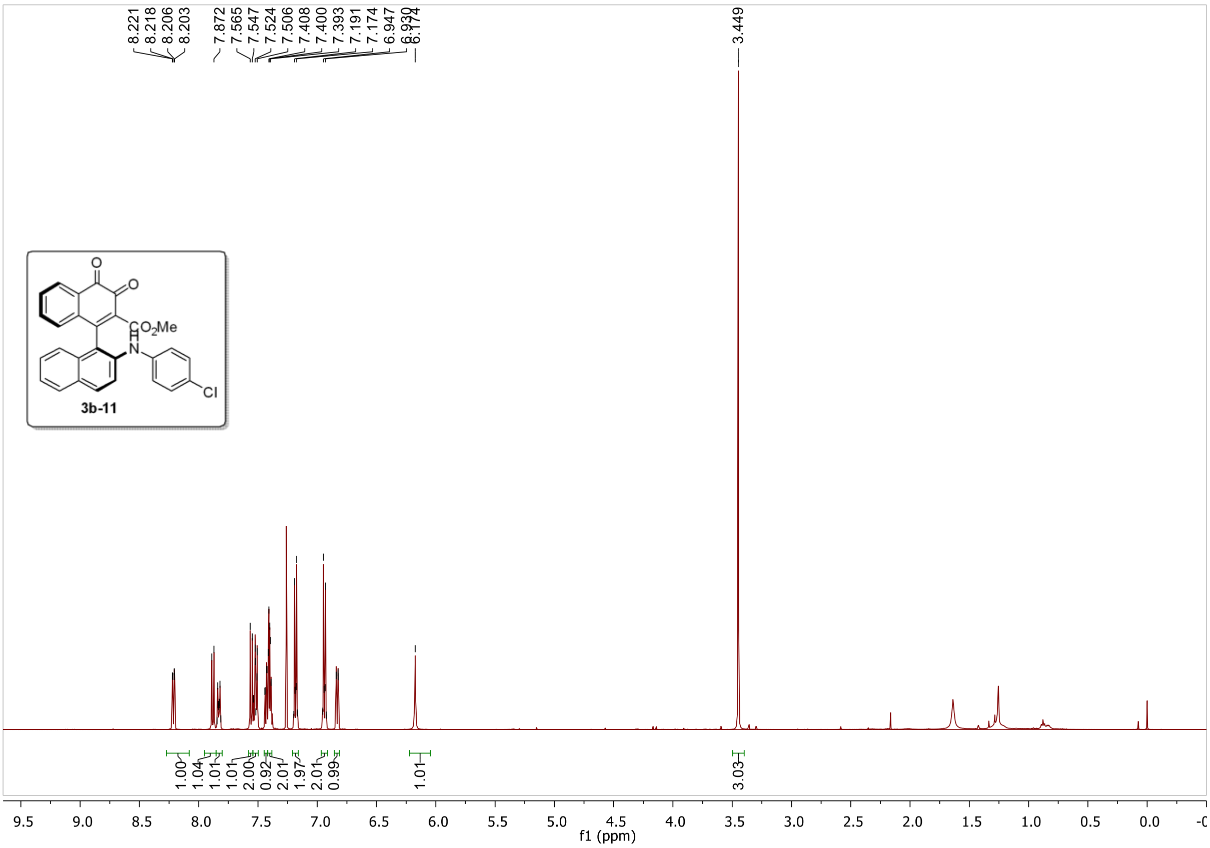

Supplementary Figure 225. ^{13}C NMR of **3b-11**.

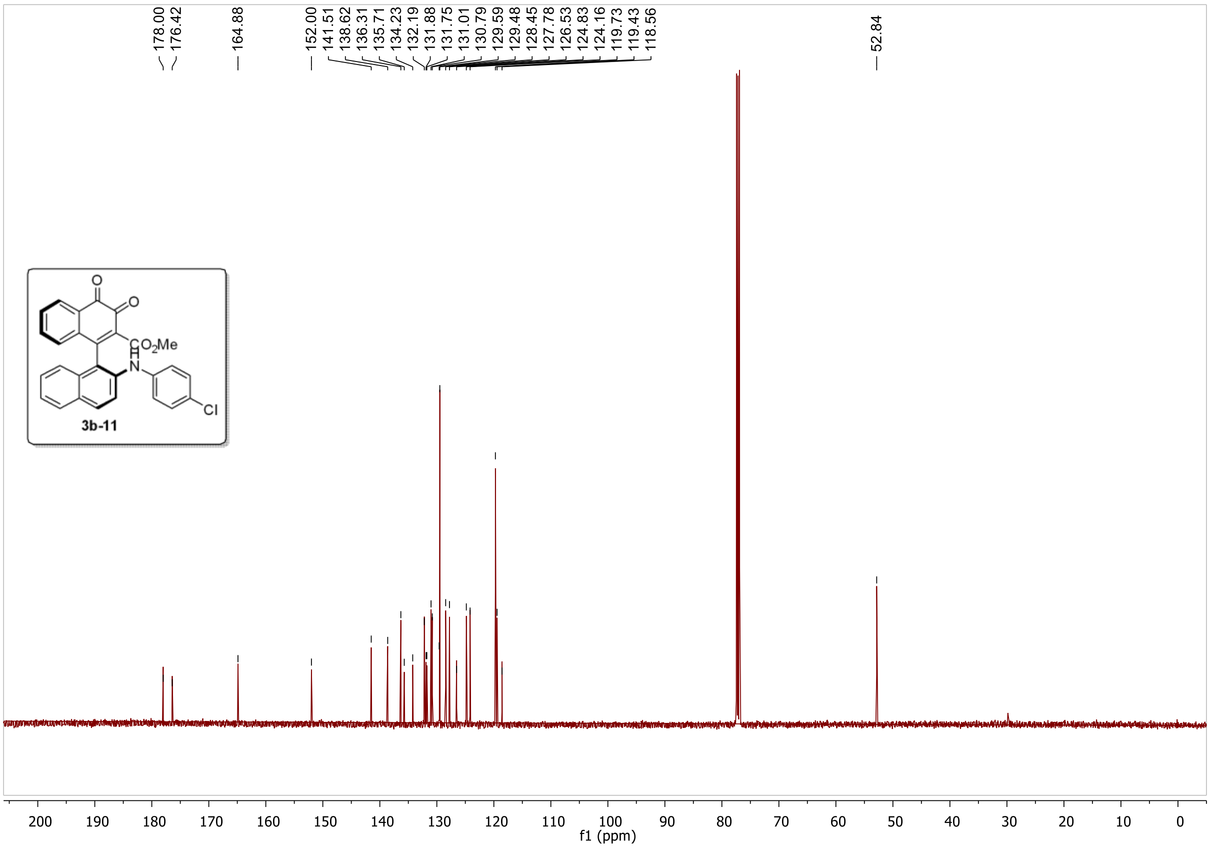

Supplementary Figure 226. ^{1}H NMR of **3b-12**.

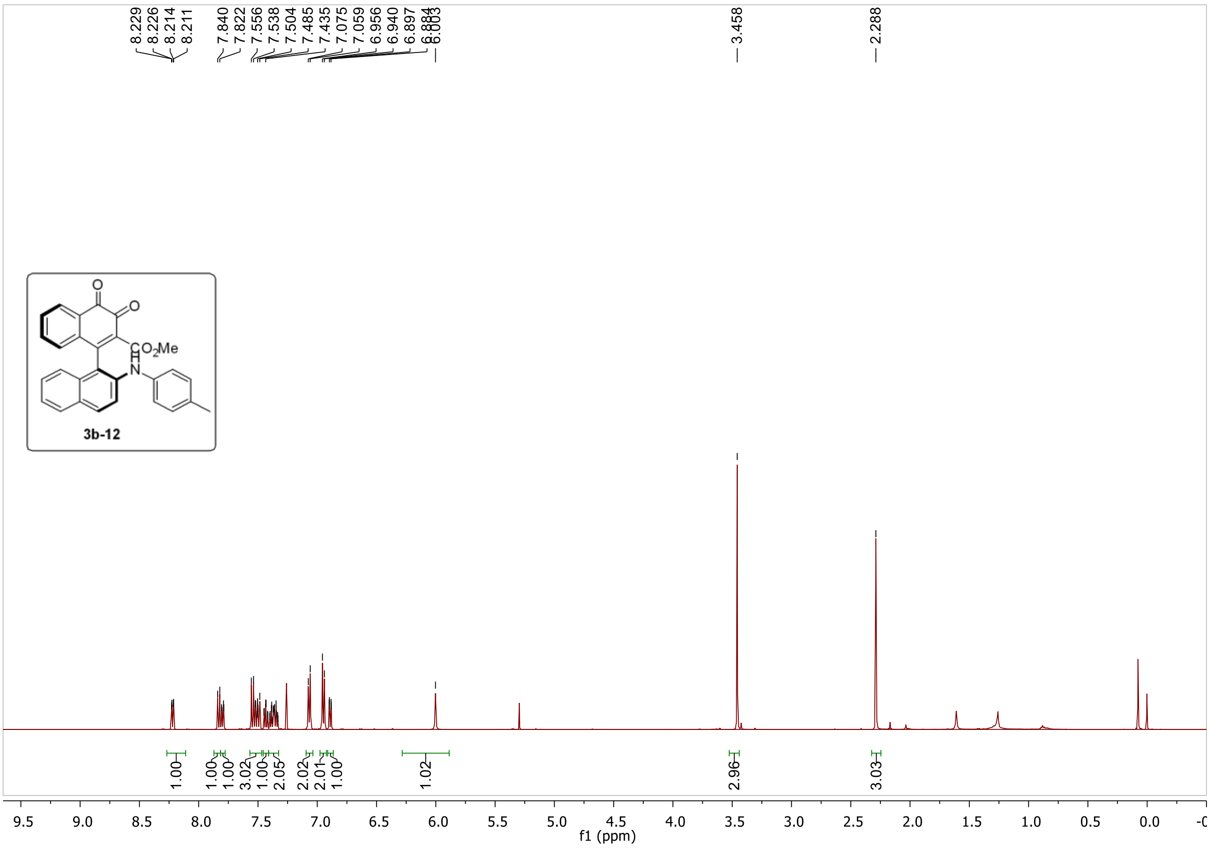

Supplementary Figure 227. ^{13}C NMR of **3b-12**.

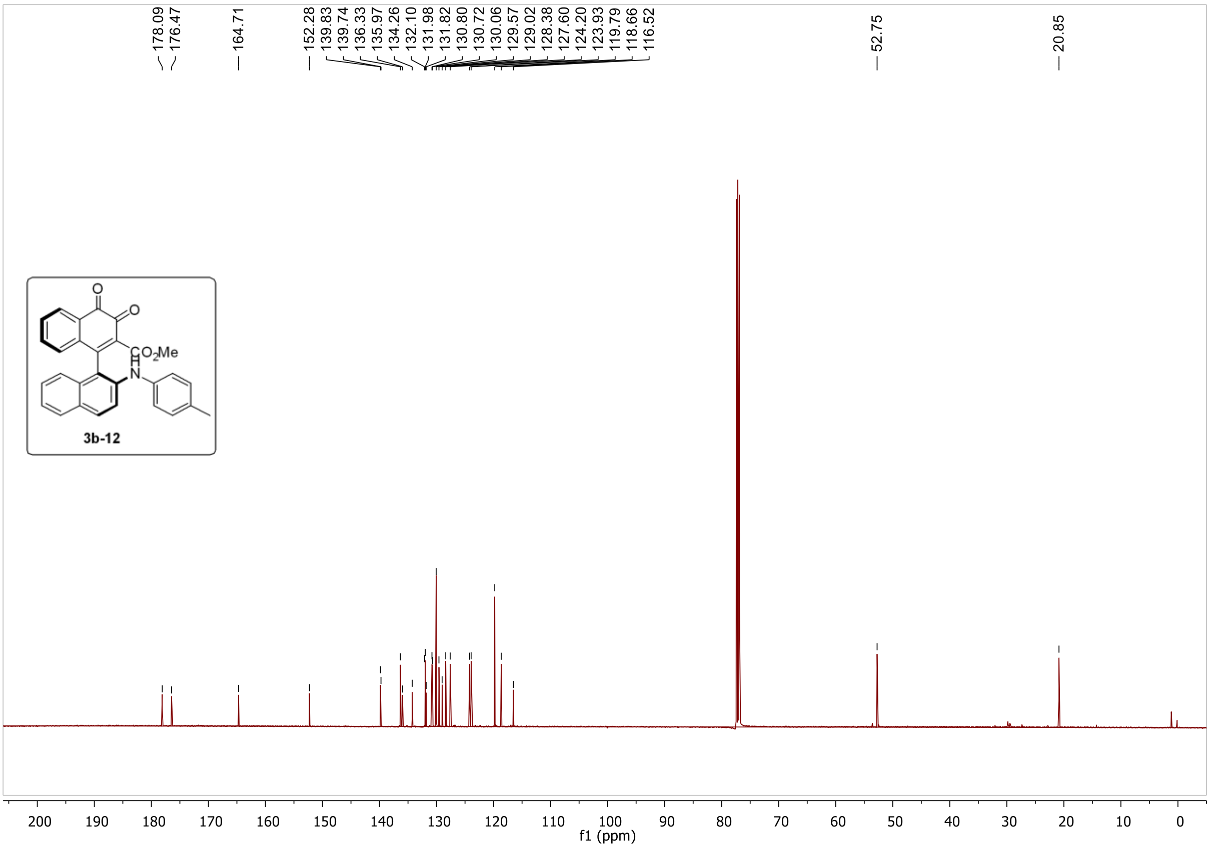

Supplementary Figure 228. ^{1}H NMR of **3b-13**.

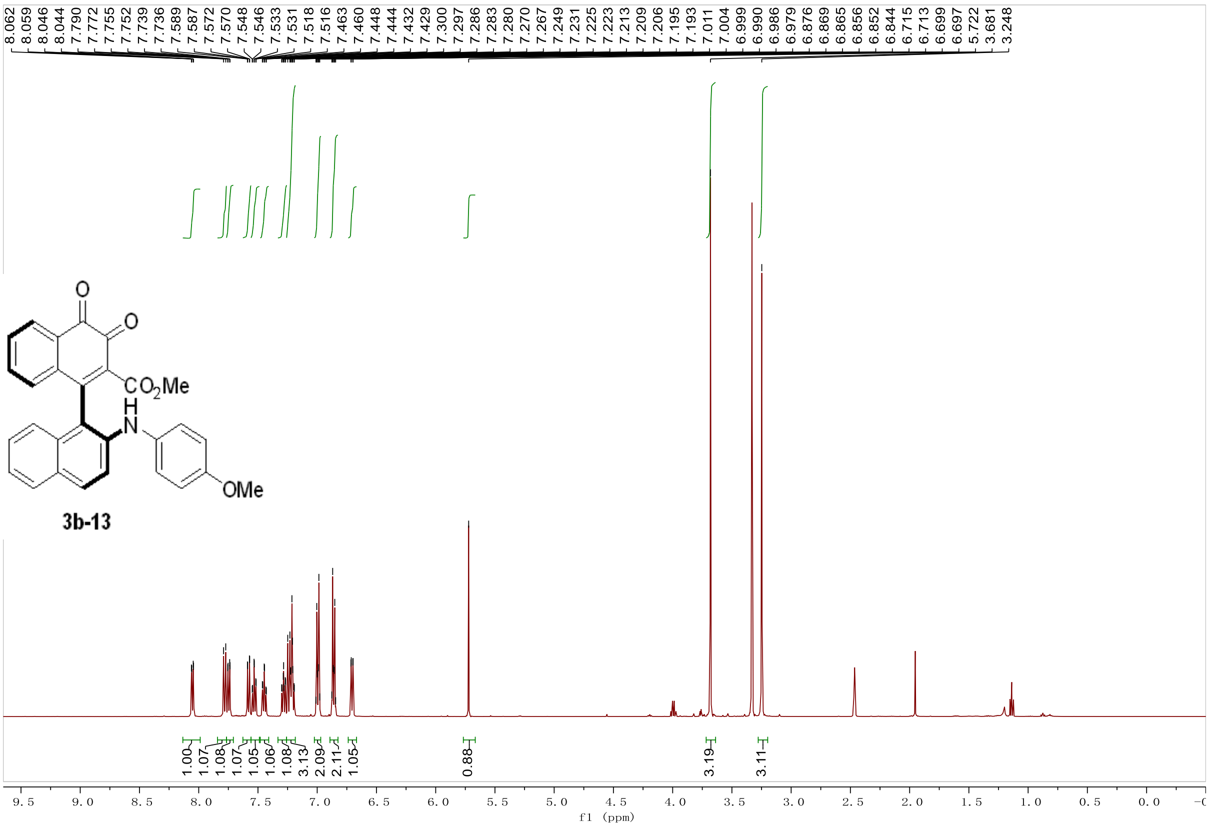

Supplementary Figure 229. ^{13}C NMR of **3b-13**.

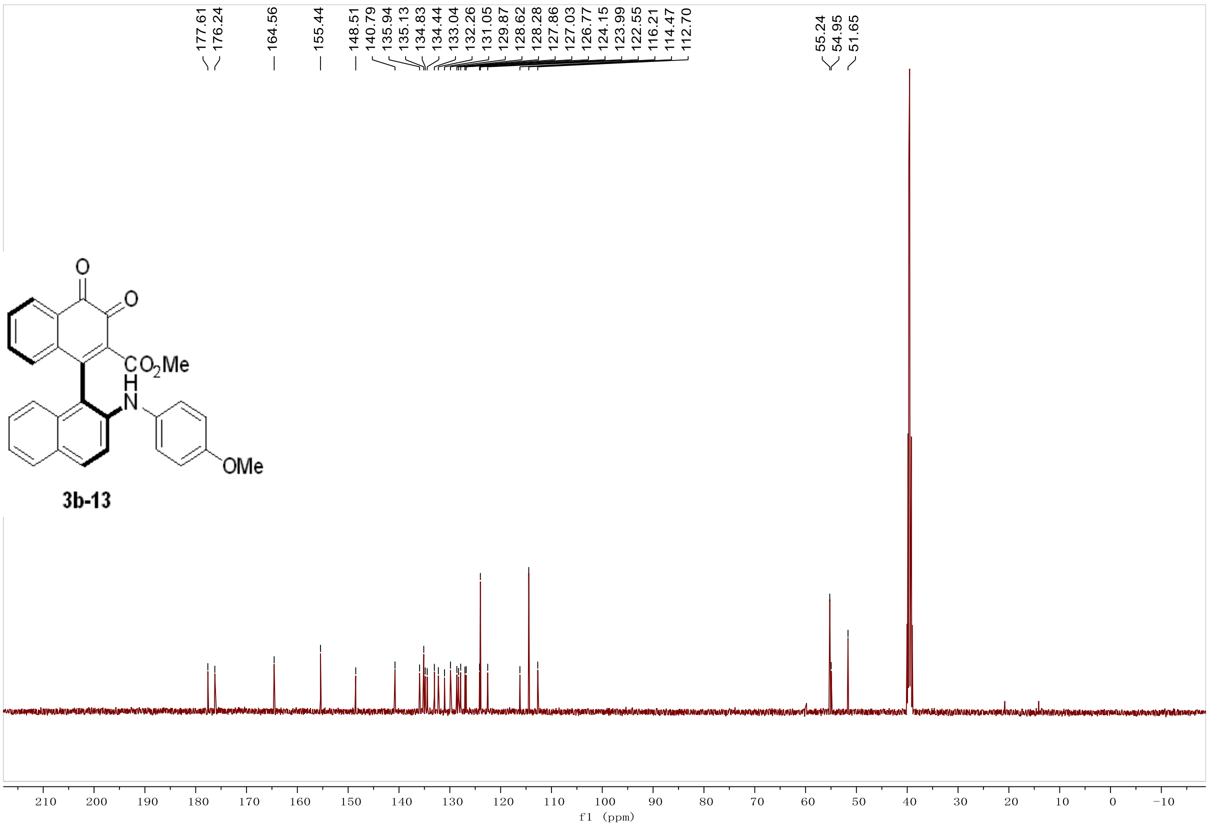

Supplementary Figure 230. ^{1}H NMR of **3b-14**.

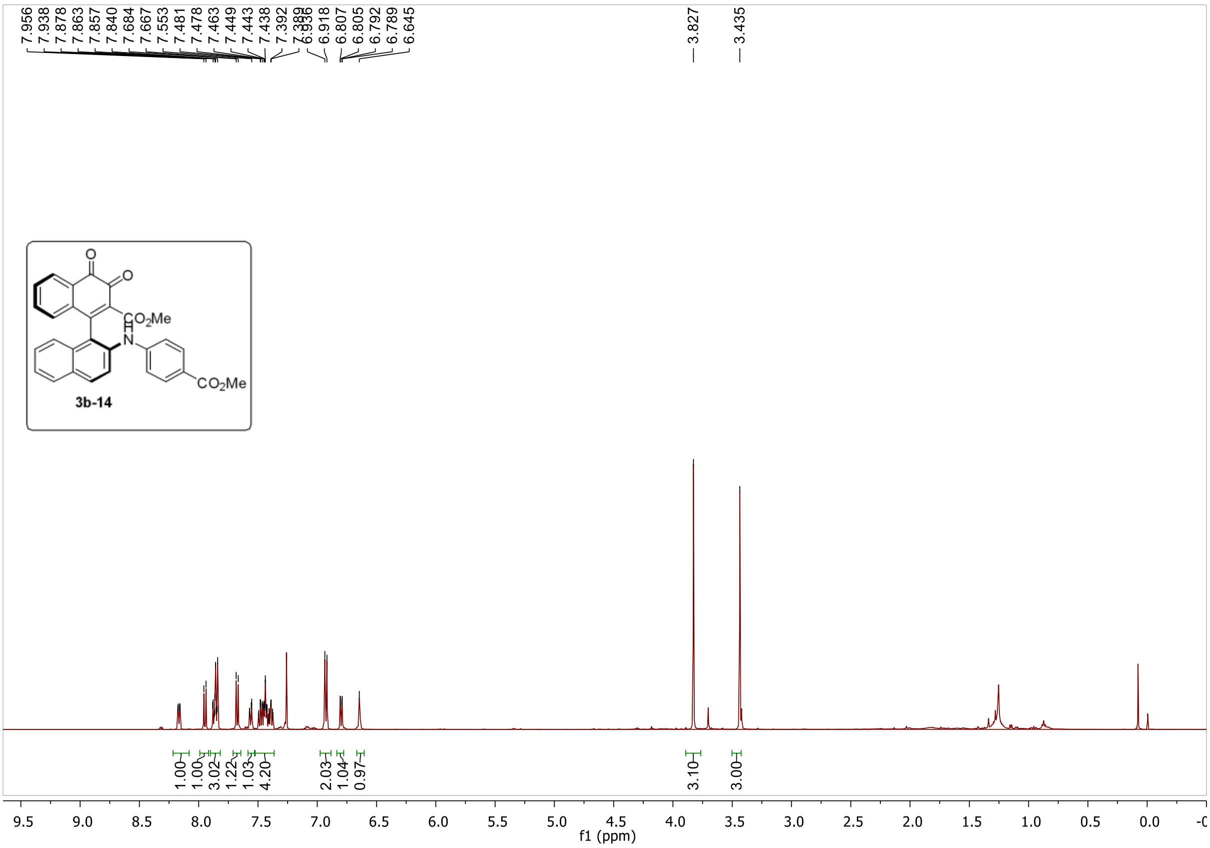

Supplementary Figure 231. ^{13}C NMR of **3b-14**.

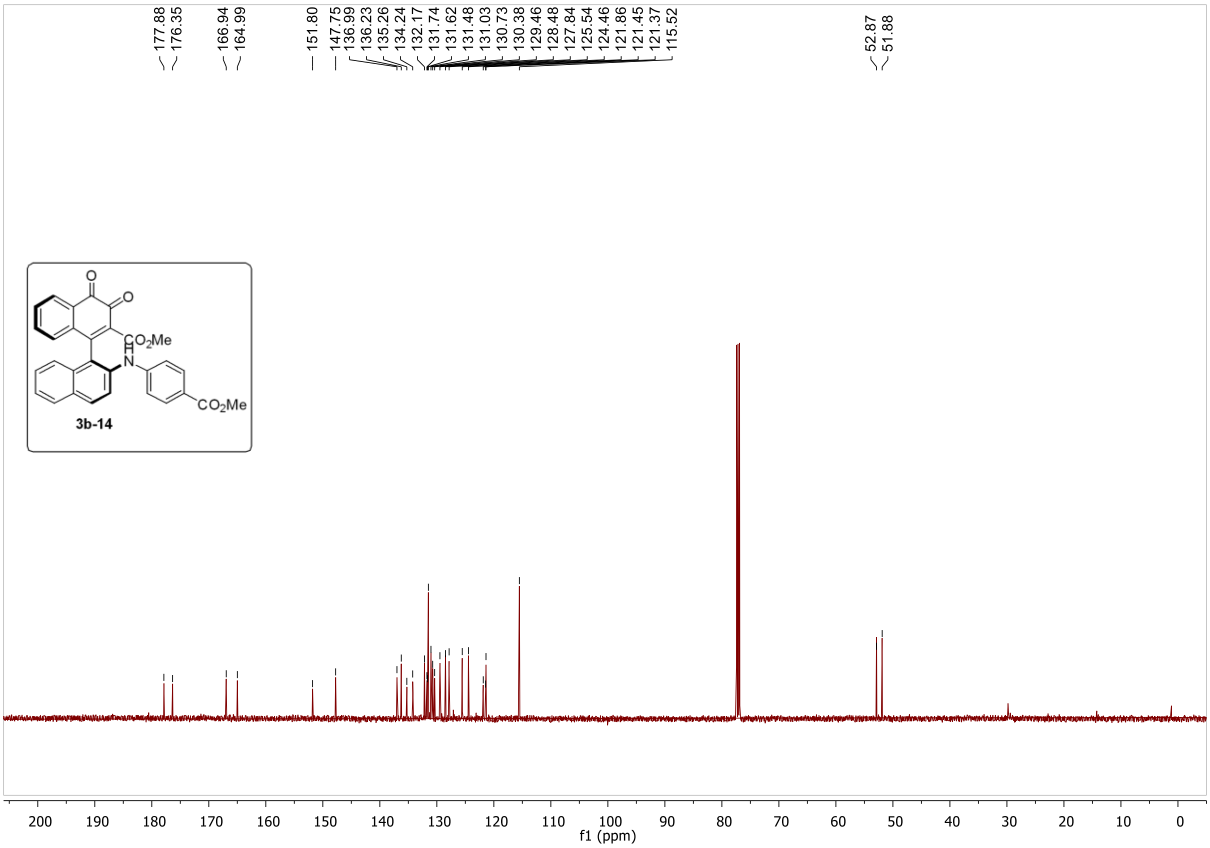

Supplementary Figure 232. ^{1}H NMR of **3c-1**.

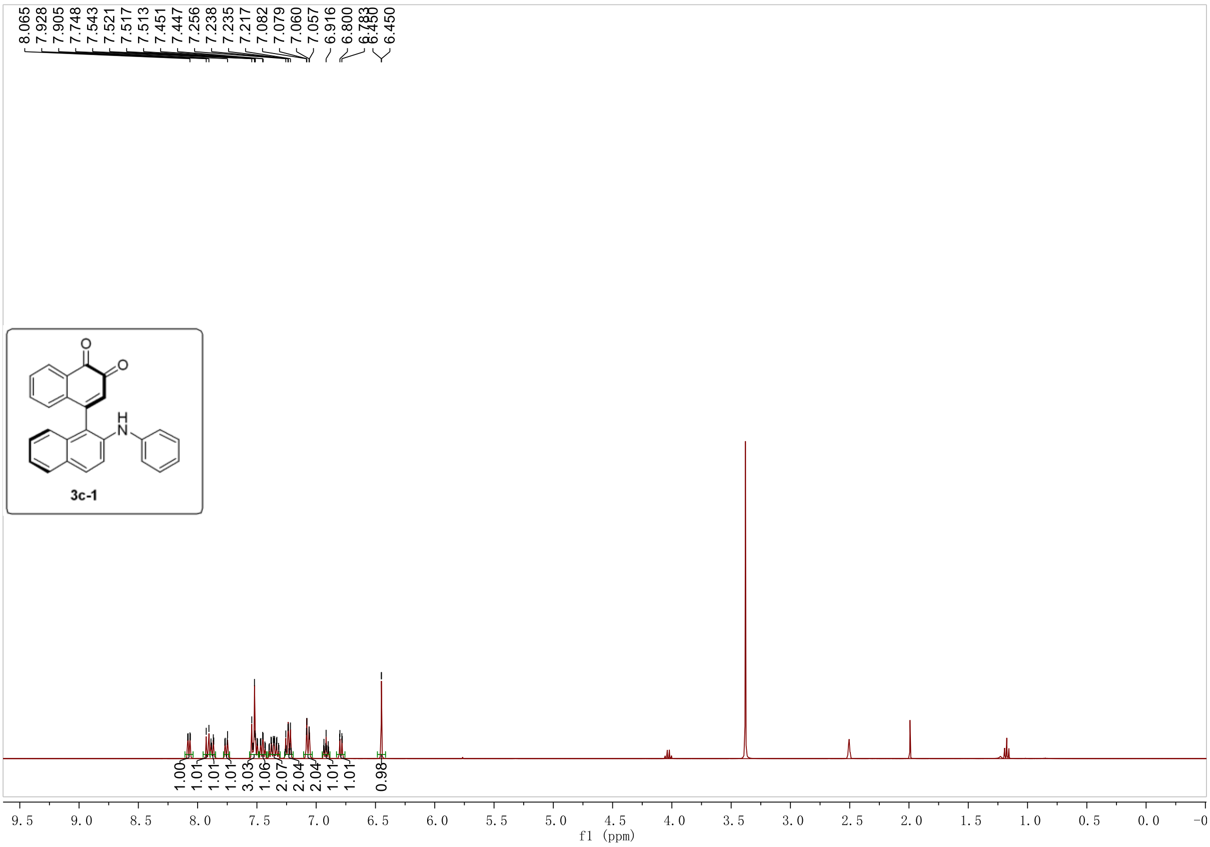

Supplementary Figure 233. ^{13}C NMR of **3c-1**.

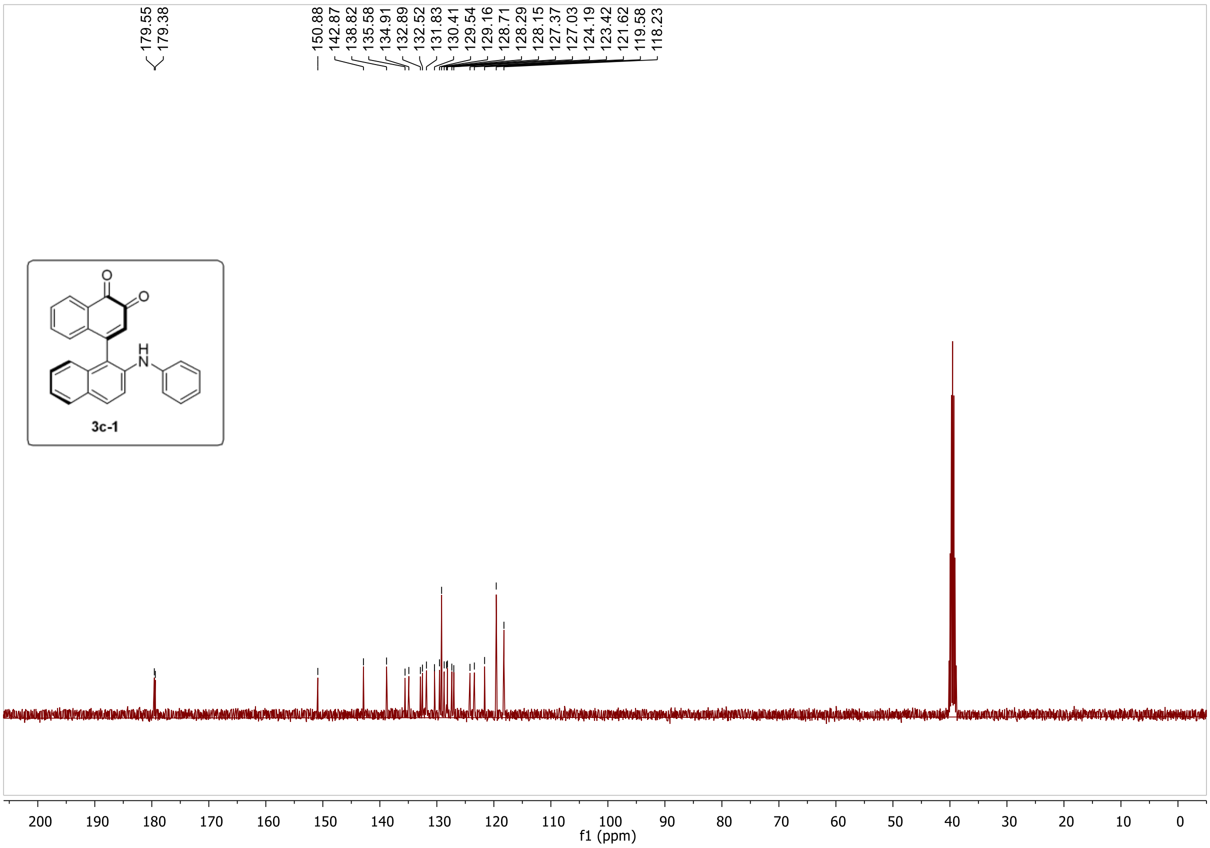

Supplementary Figure 234. ^{1}H NMR of **3c-2**.

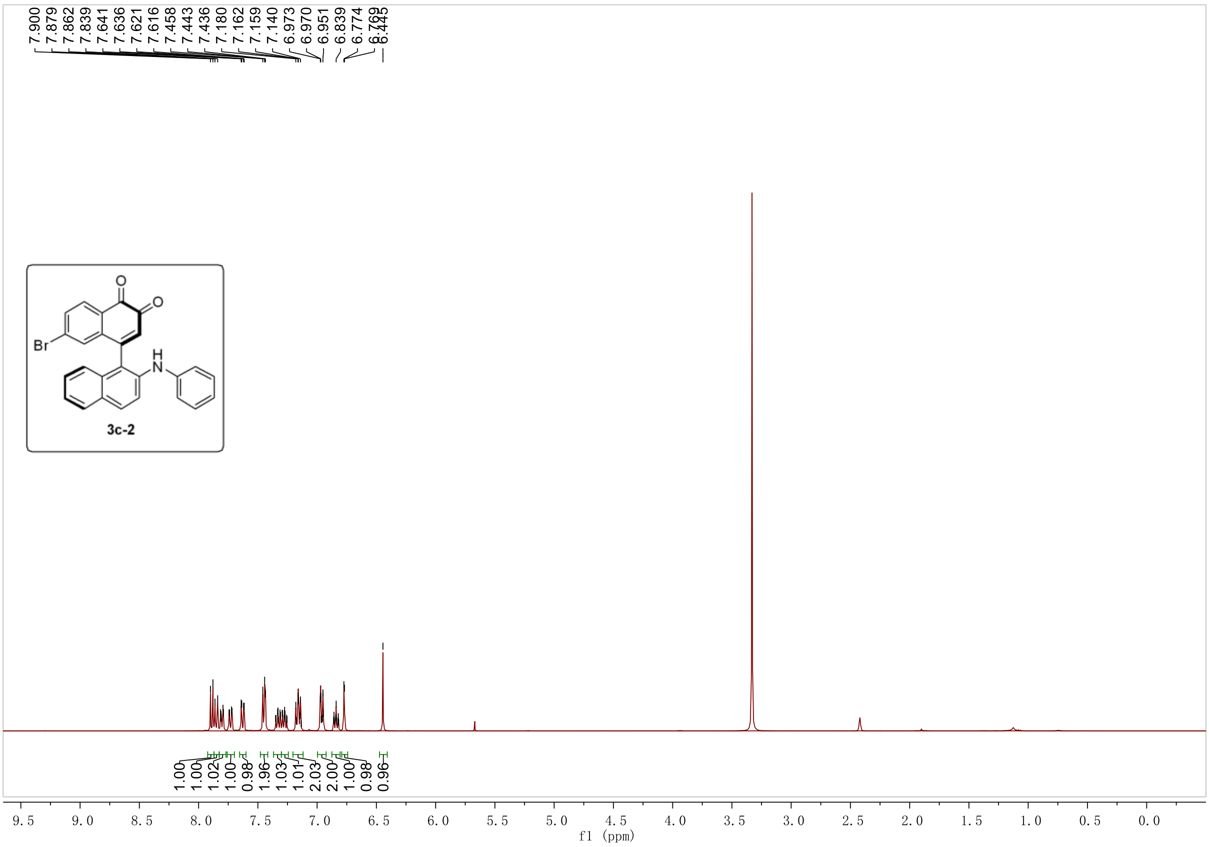

Supplementary Figure 235. ^{13}C NMR of **3c-2**.

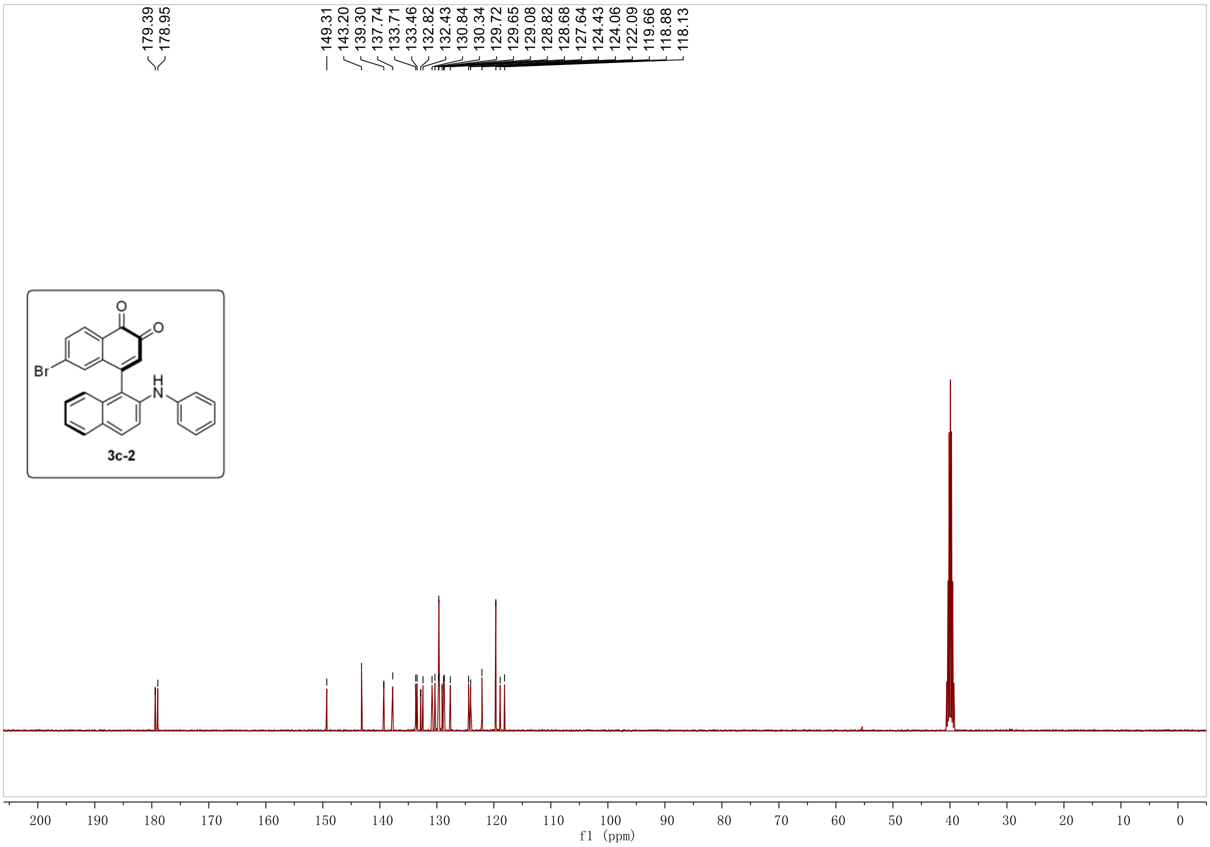

Supplementary Figure 236. ^{1}H NMR of **3c-3**.

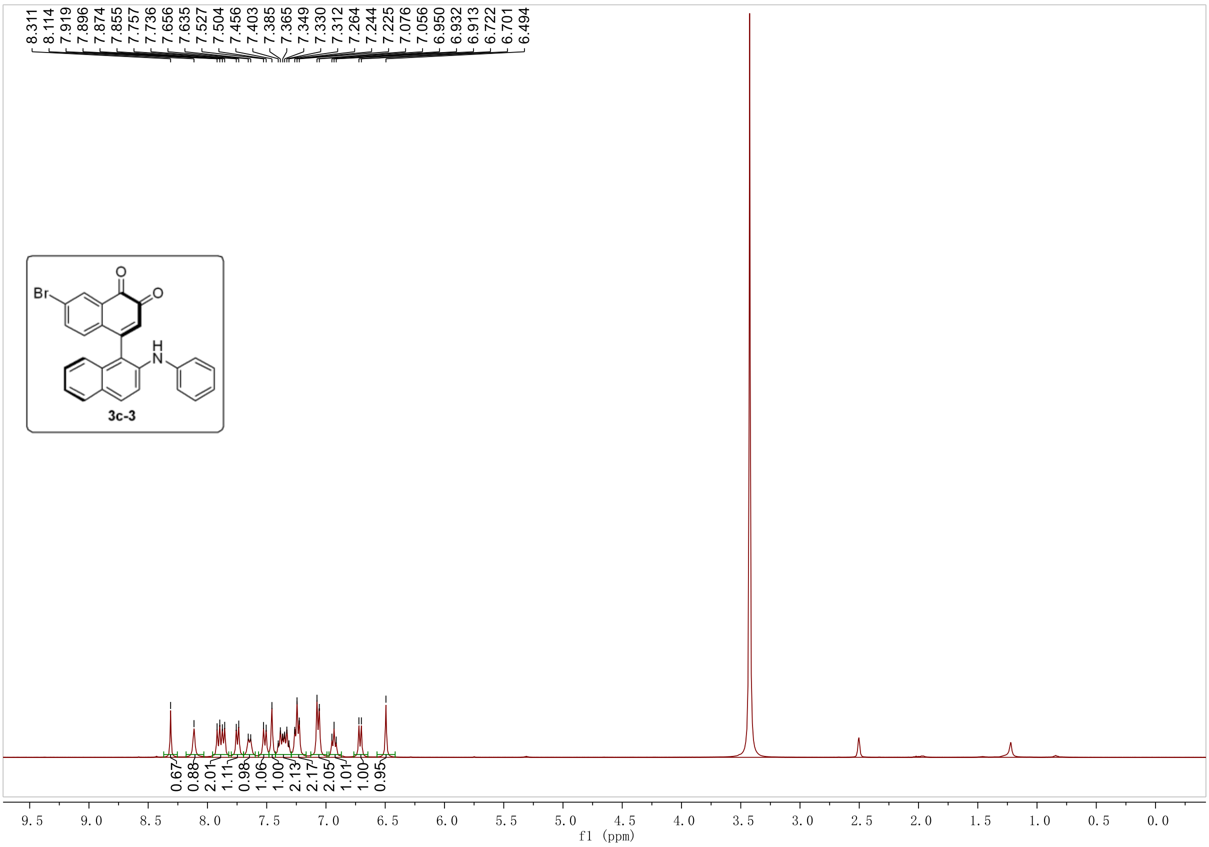

Supplementary Figure 237. ^{13}C NMR of **3c-3**.

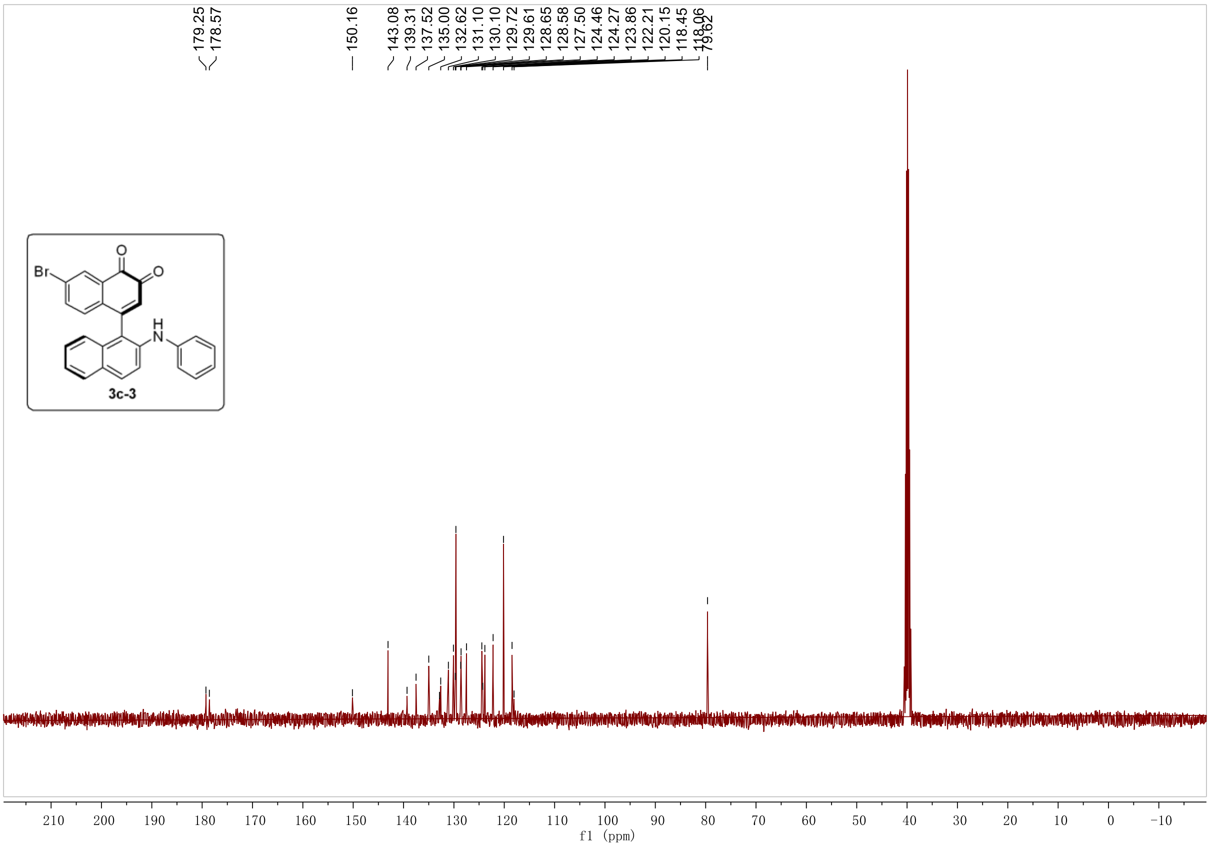

Supplementary Figure 238. ^{1}H NMR of **3c-4**.

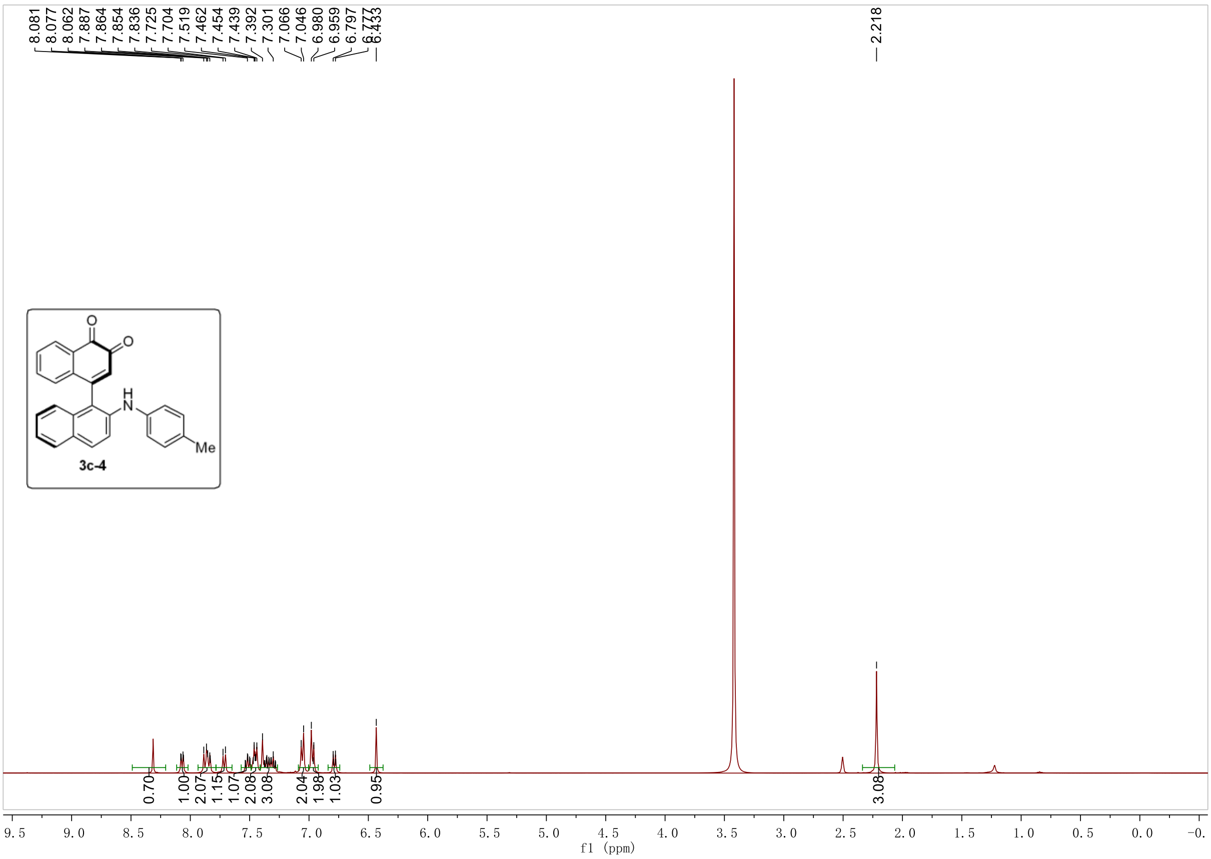

Supplementary Figure 239. ^{13}C NMR of **3c-4**.

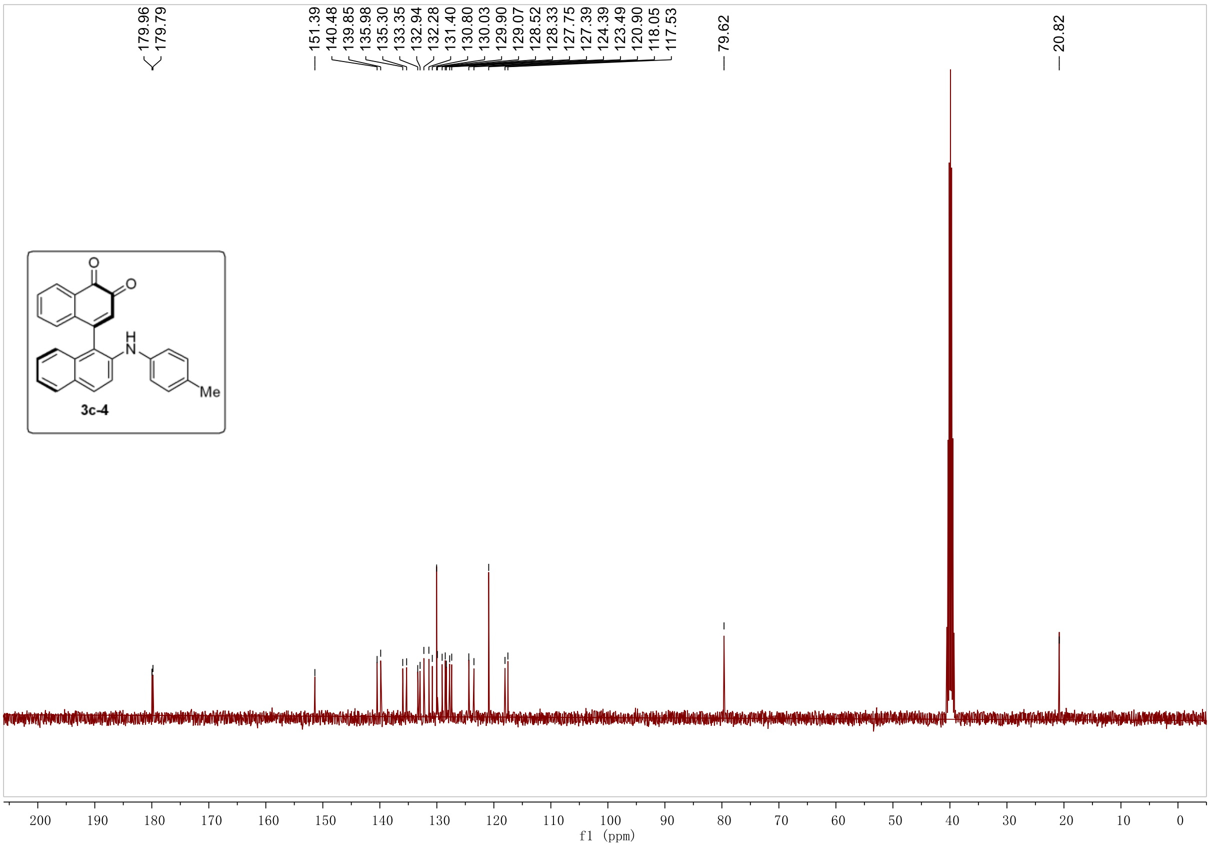

Supplementary Figure 240. ^{1}H NMR of **3c-5**.

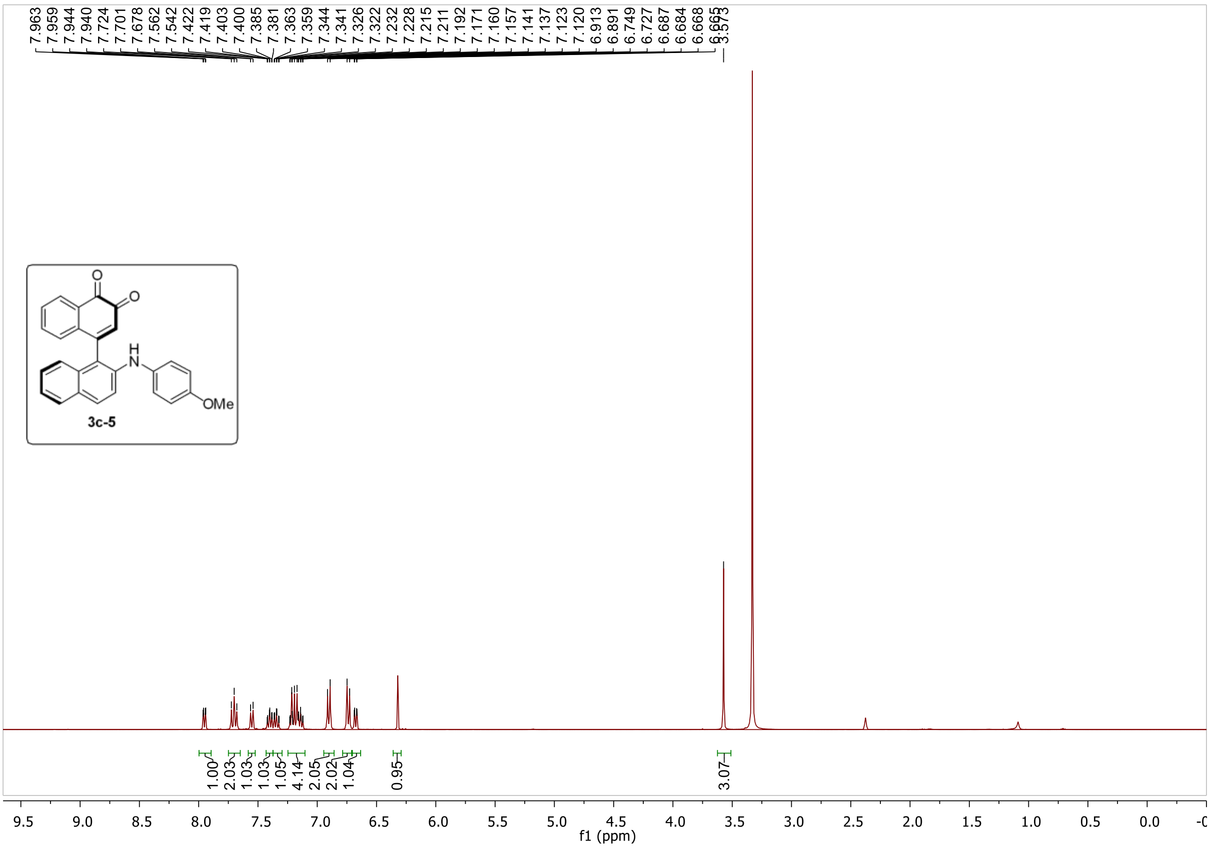

Supplementary Figure 241. ^{13}C NMR of **3c-5**.

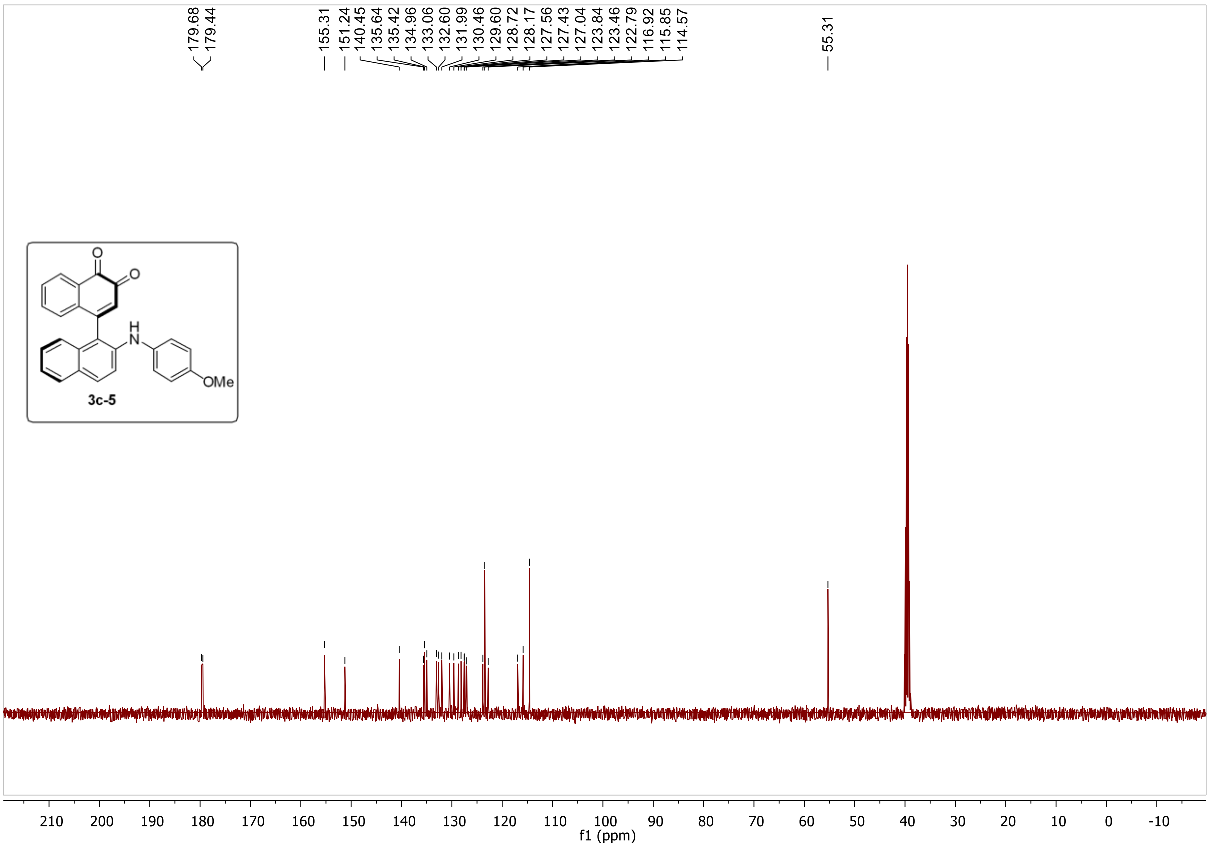

Supplementary Figure 242. ^{1}H NMR of **3d-1**.

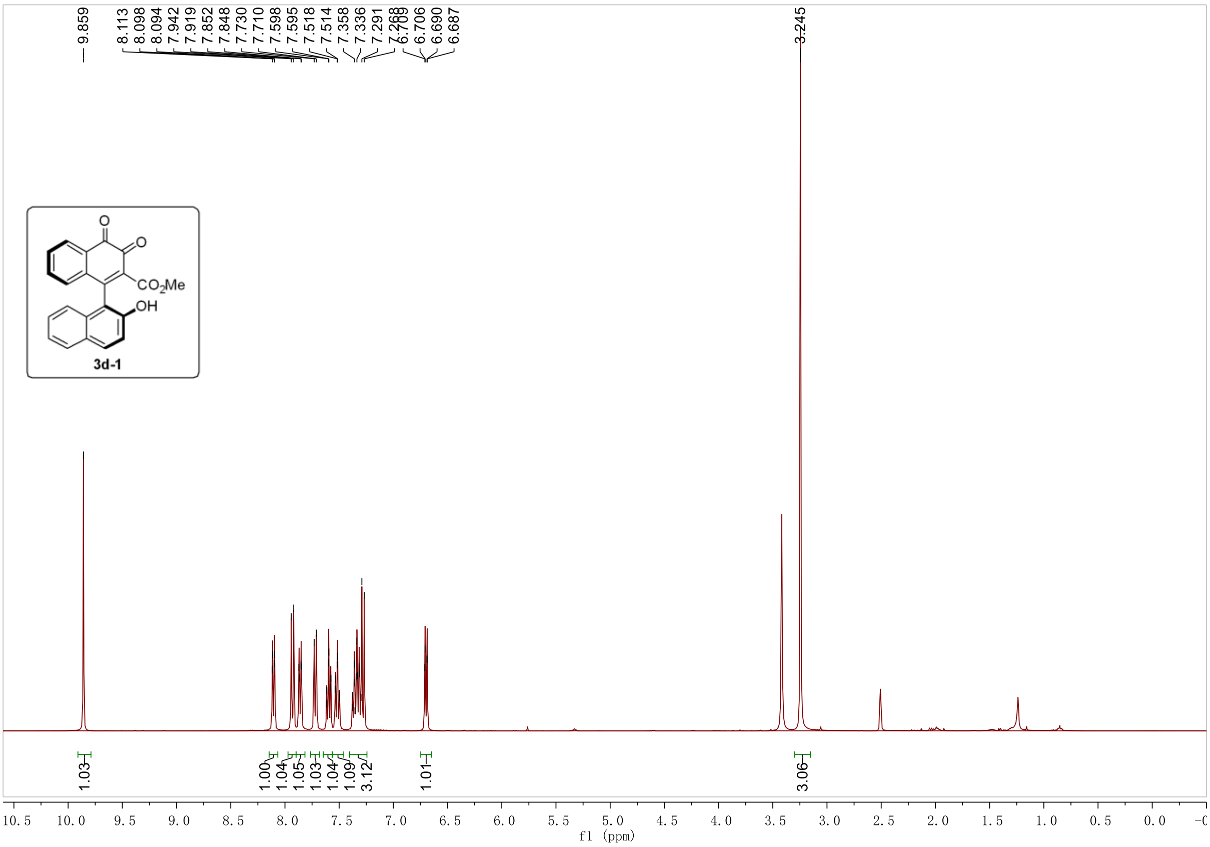

Supplementary Figure 243. ^{13}C NMR of **3d-1**.

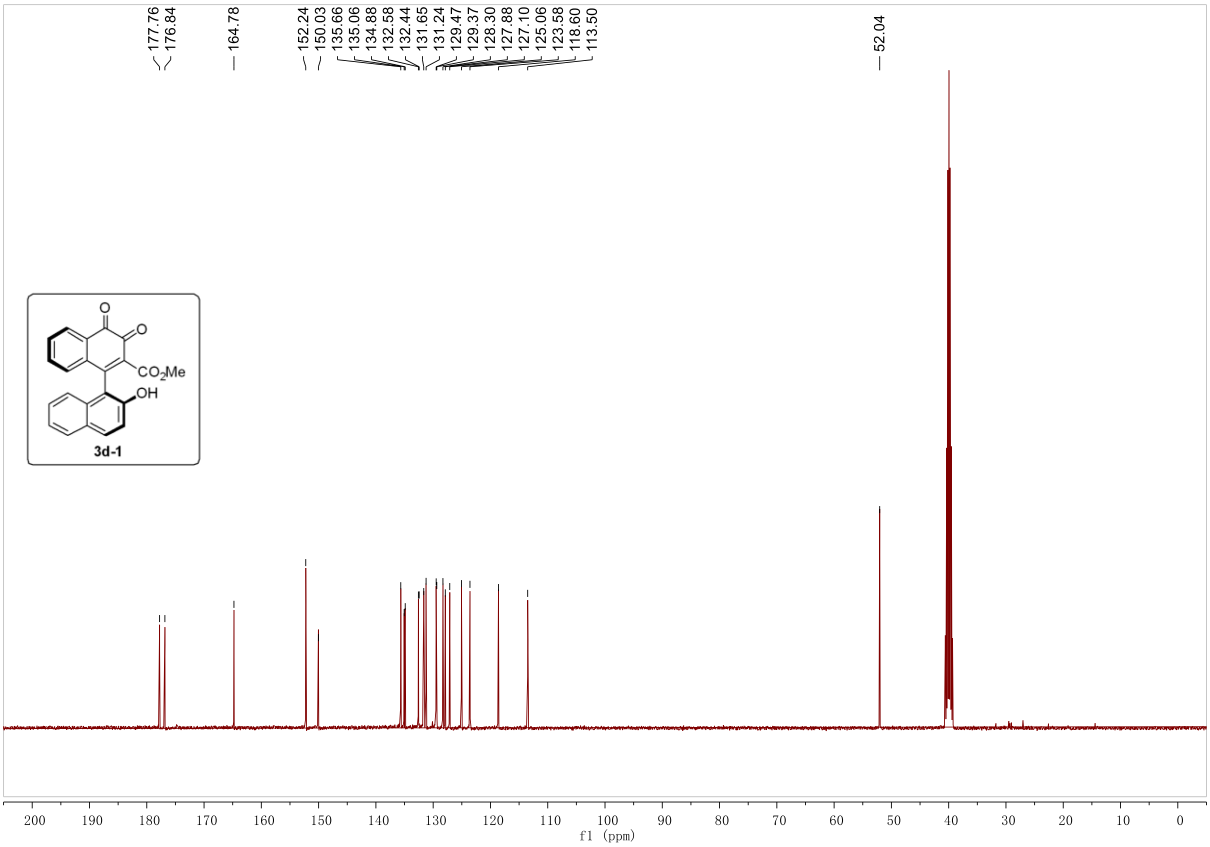

Supplementary Figure 244. ^{1}H NMR of **3d-2**.

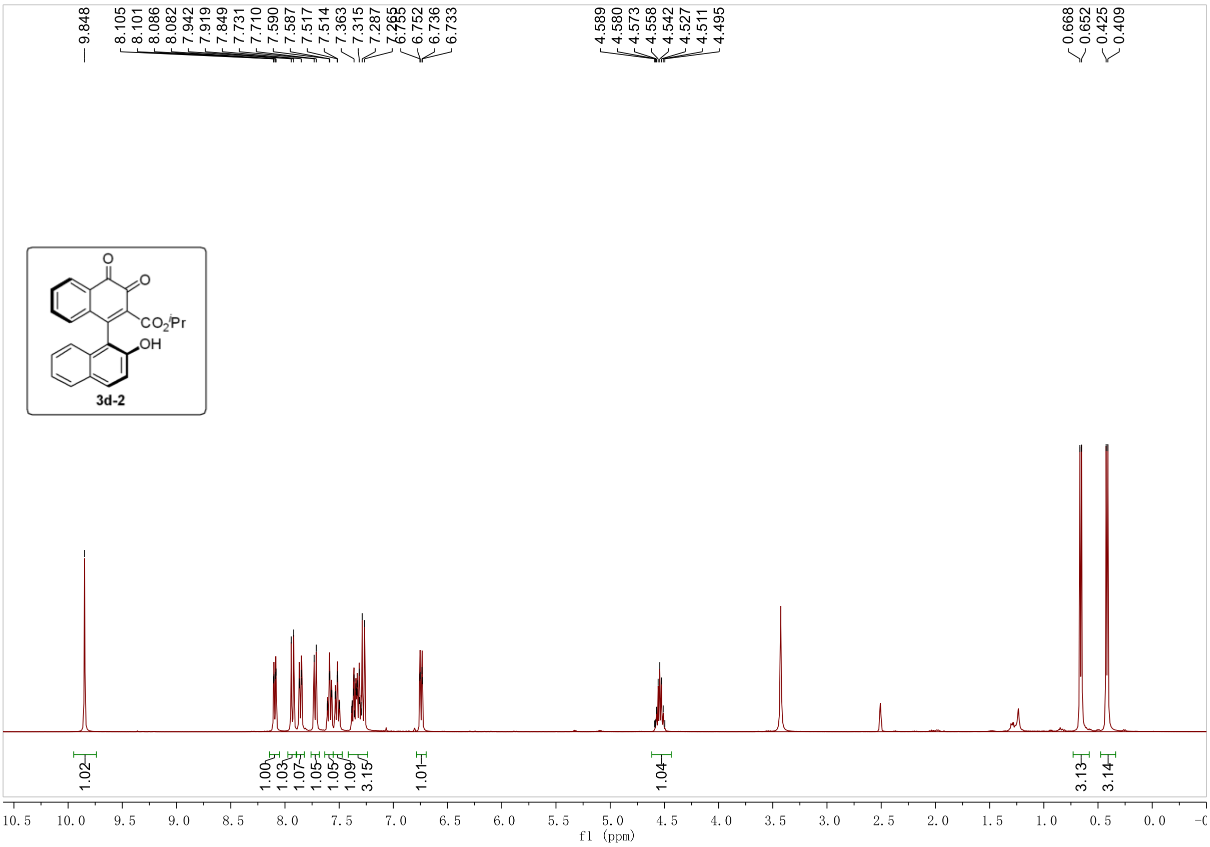

Supplementary Figure 245. ^{13}C NMR of **3d-2**.

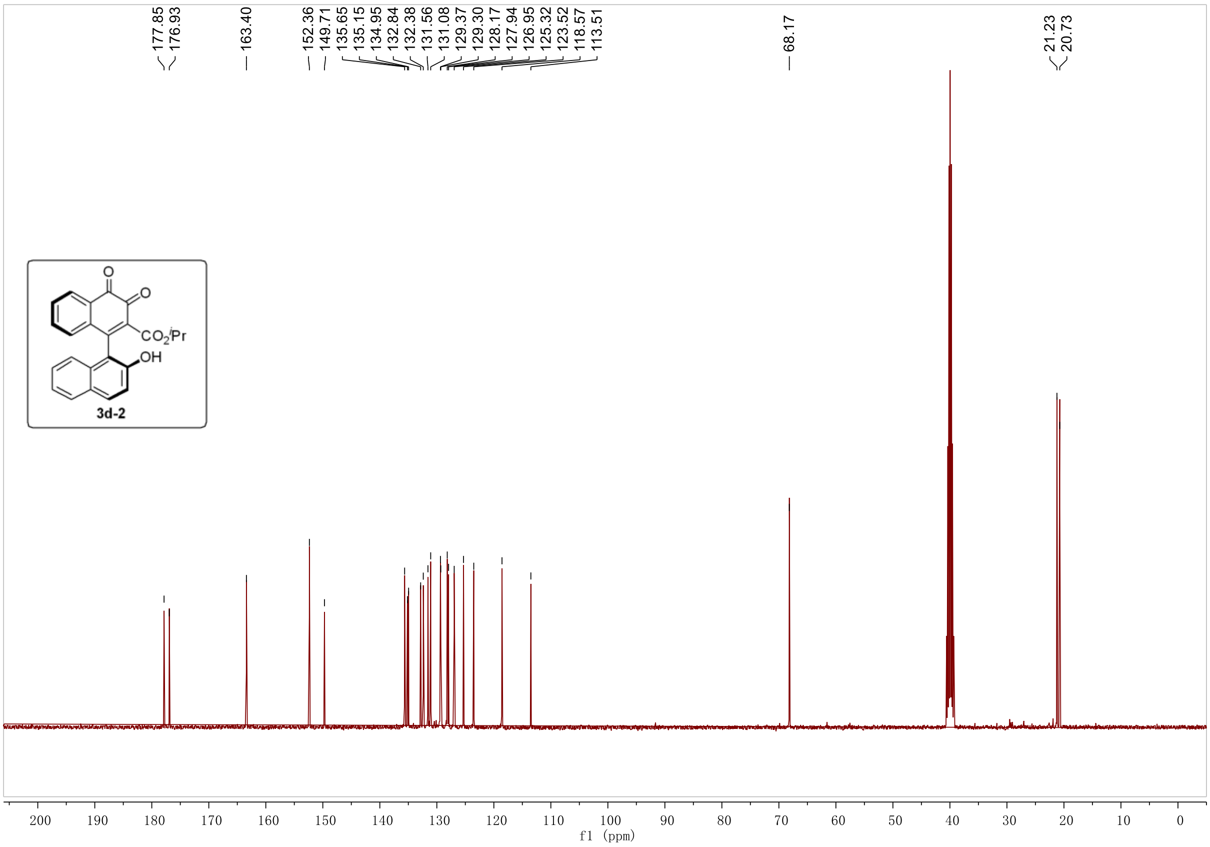

Supplementary Figure 246. ^{1}H NMR of **3d-3**.

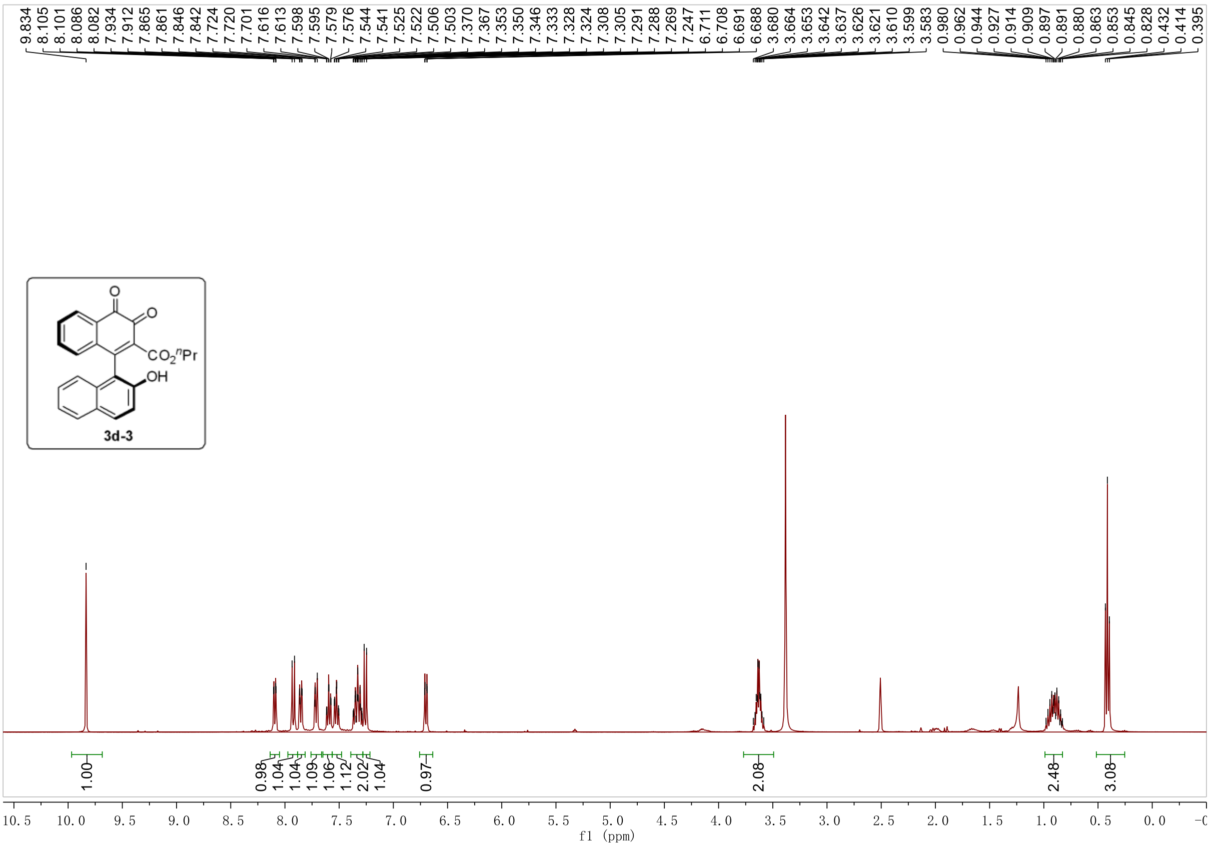

Supplementary Figure 247. ^{13}C NMR of **3d-3**.

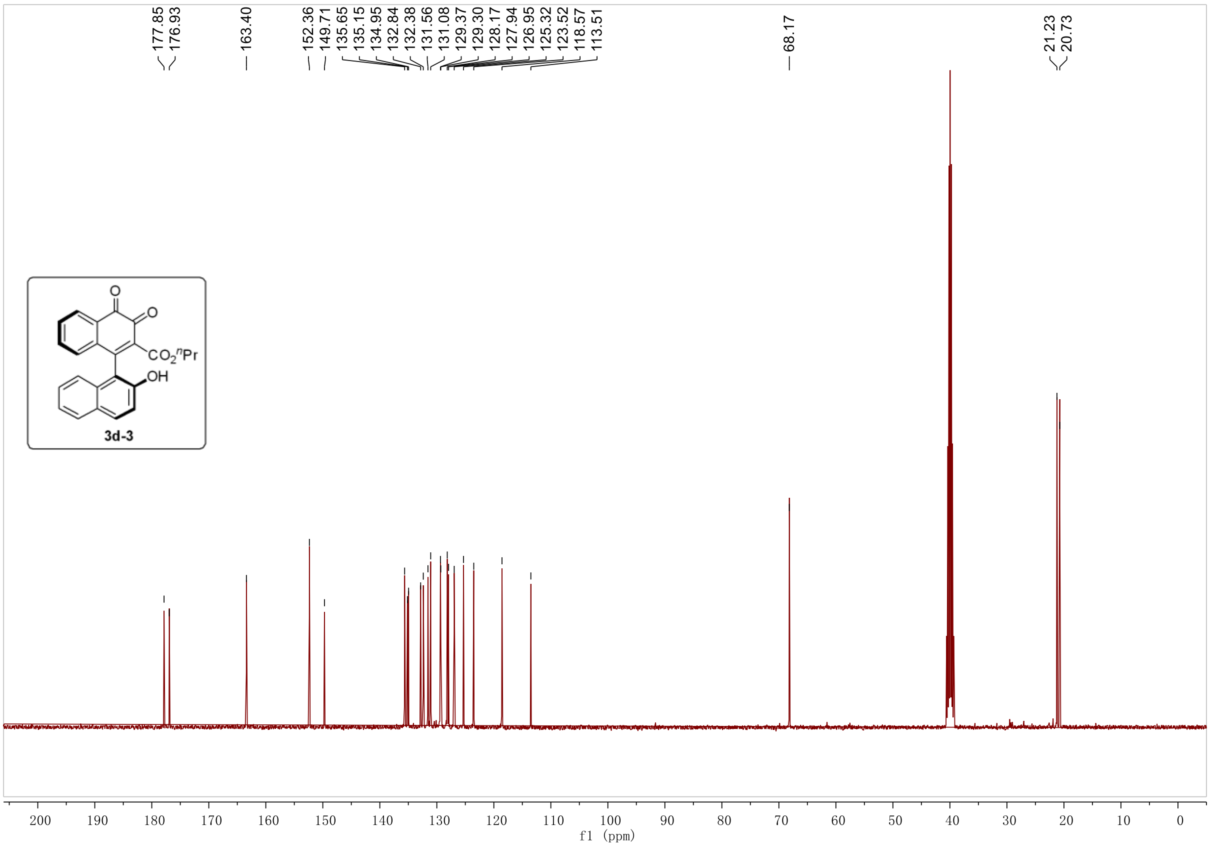

Supplementary Figure 248. ^{1}H NMR of **3d-4**.

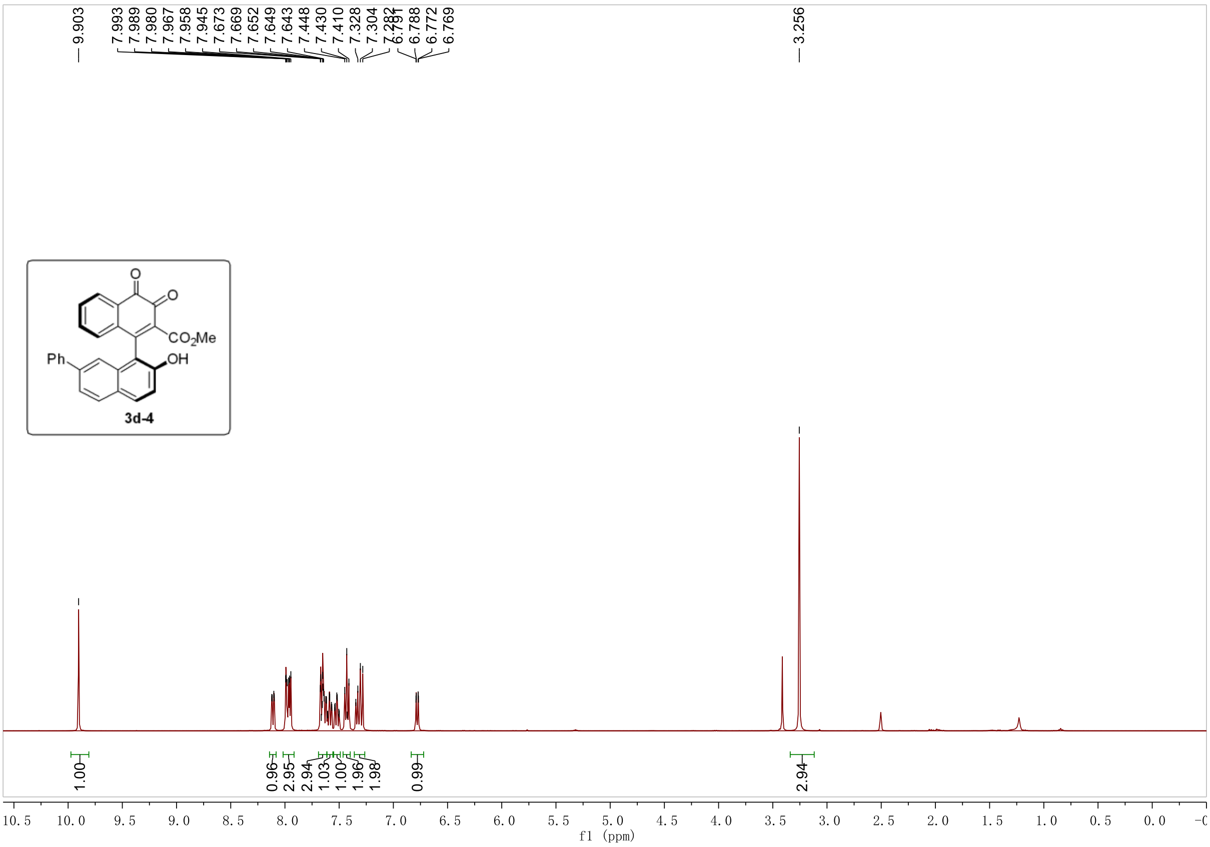

Supplementary Figure 249. ^{13}C NMR of **3d-4**.

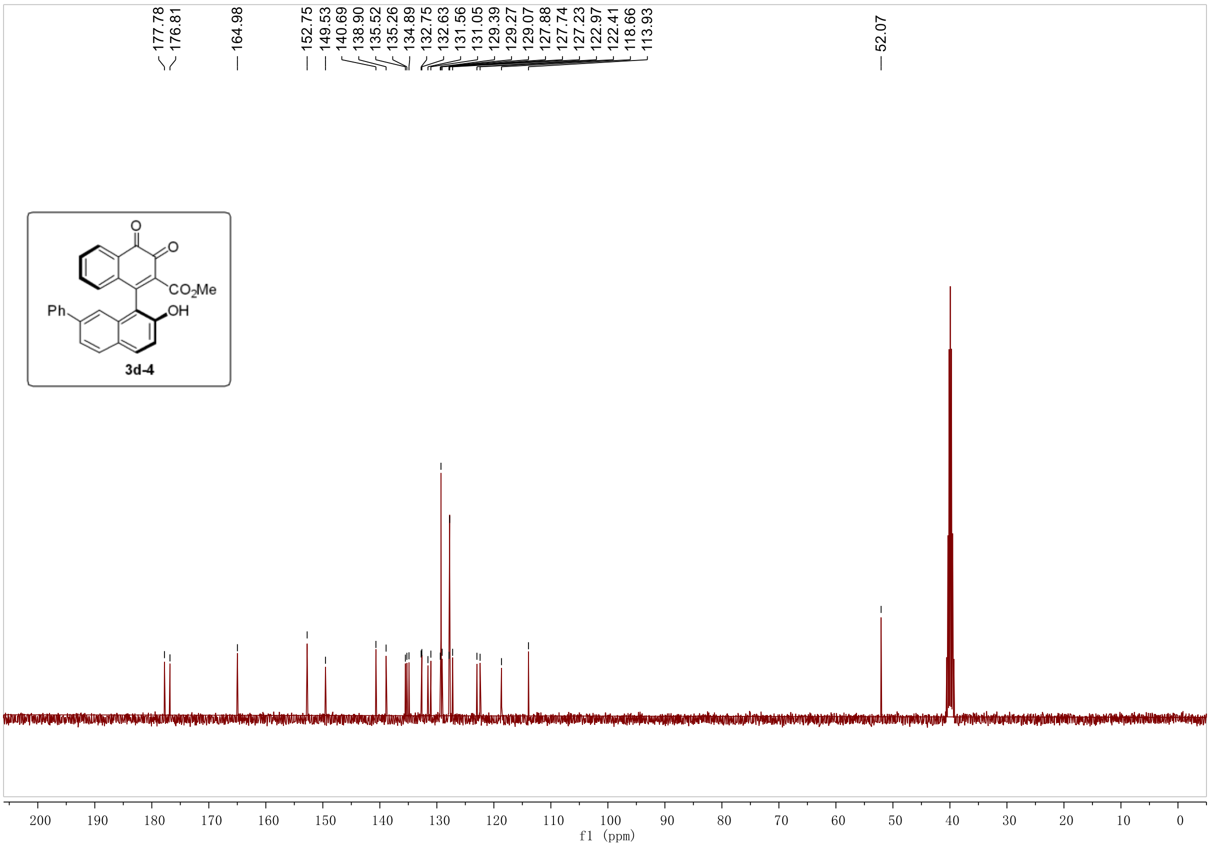

Supplementary Figure 250. ^{1}H NMR of **3d-5**.

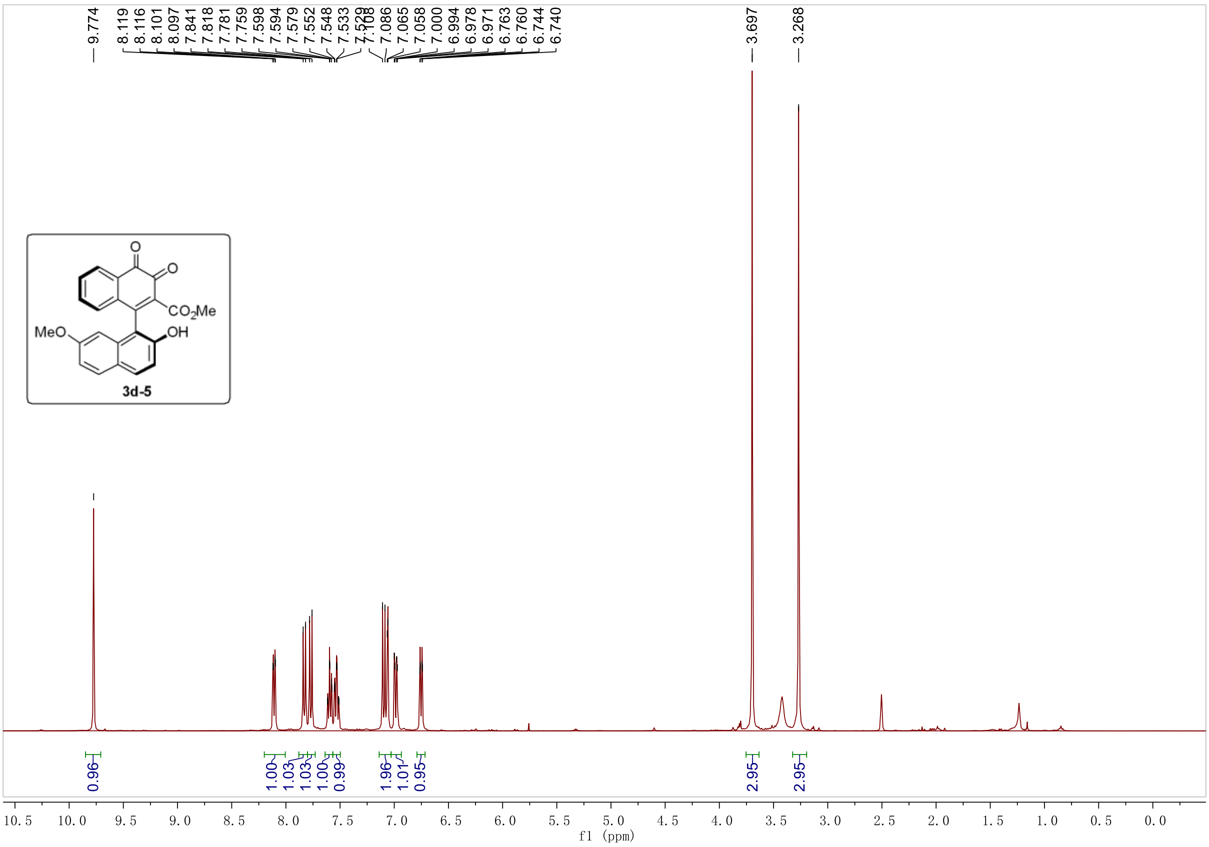

Supplementary Figure 251. ^{13}C NMR of **3d-5**.

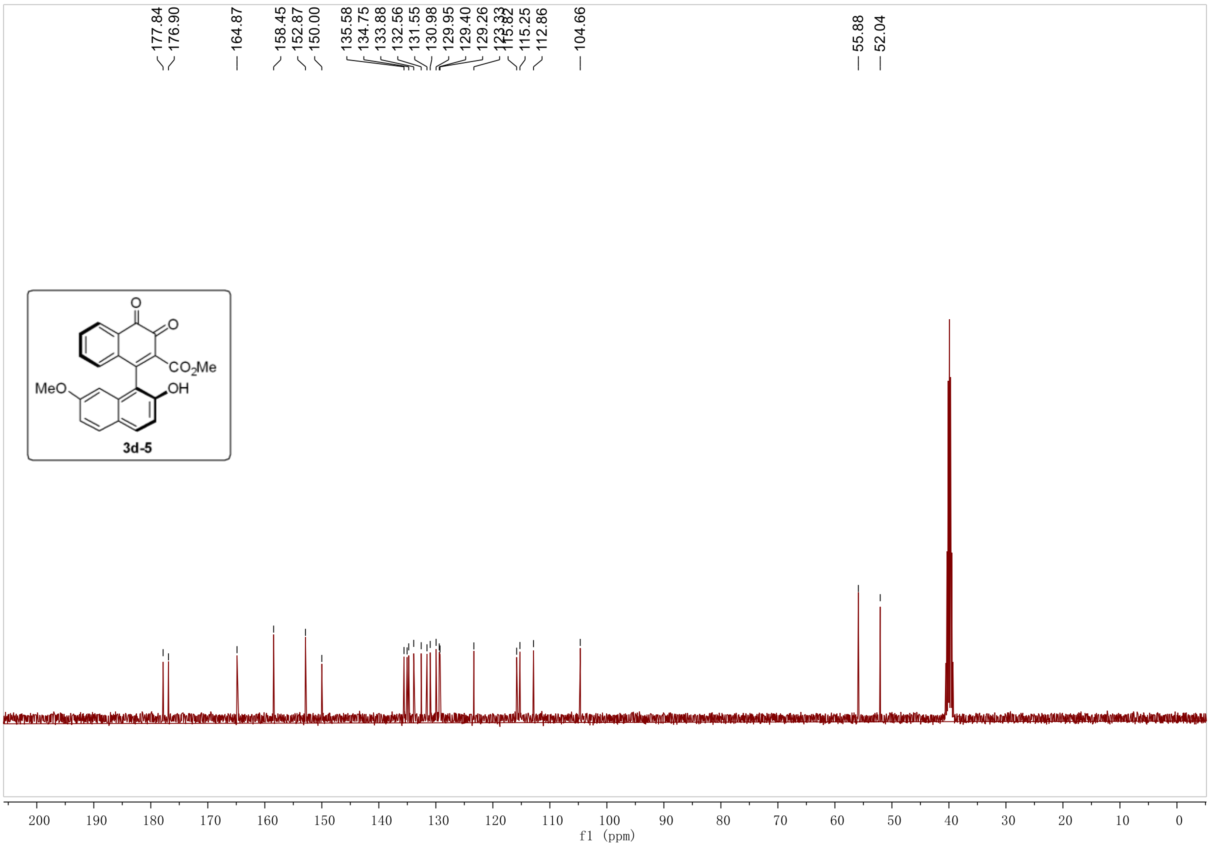

Supplementary Figure 252. ^{1}H NMR of **3d-6**.

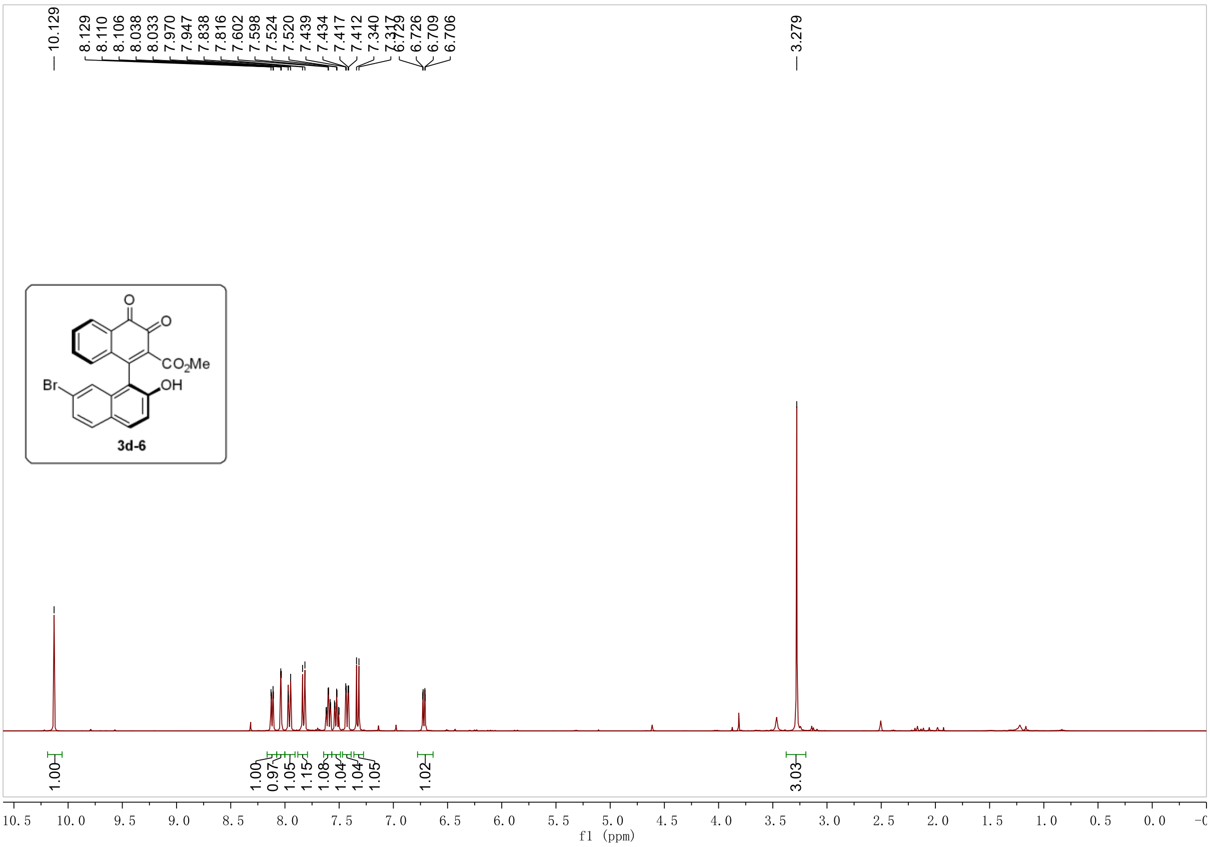

Supplementary Figure 253. ^{13}C NMR of **3d-6**.

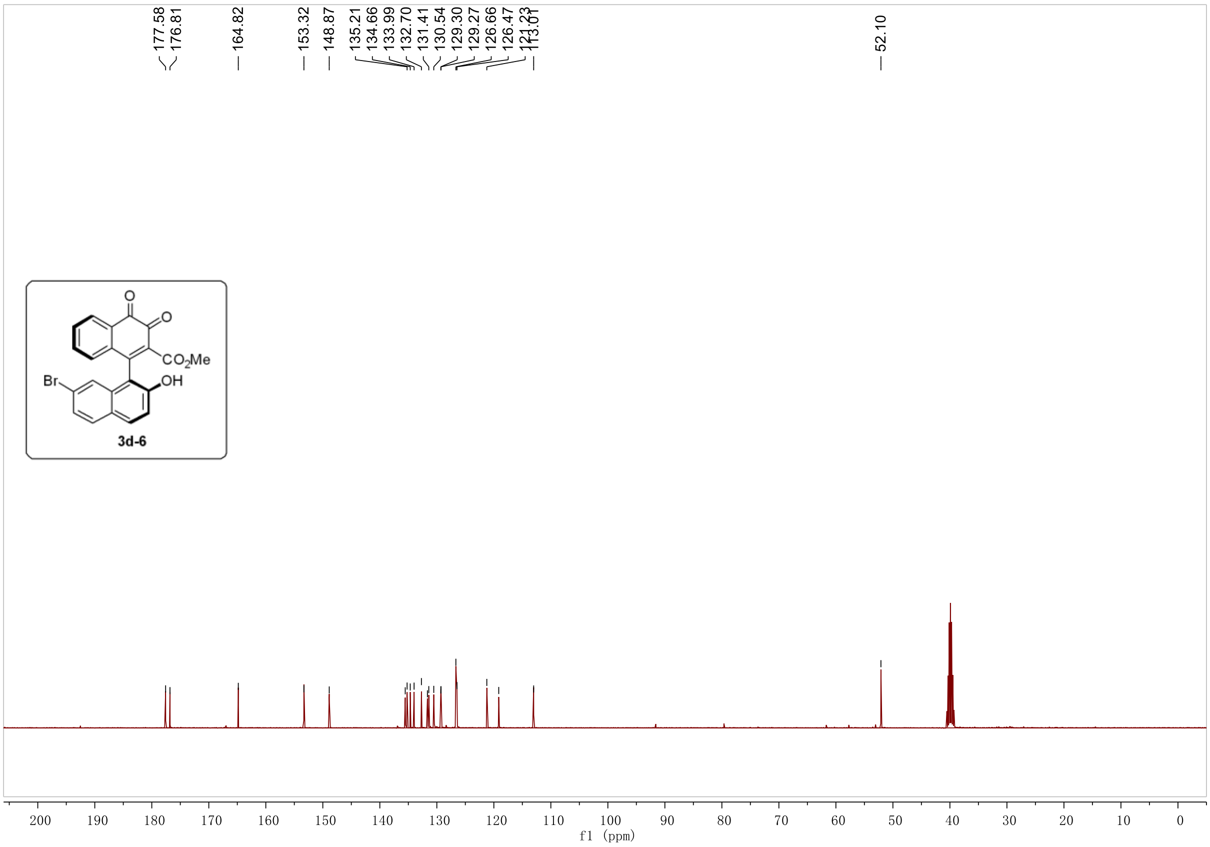

Supplementary Figure 254. ^{1}H NMR of **3d-7**.

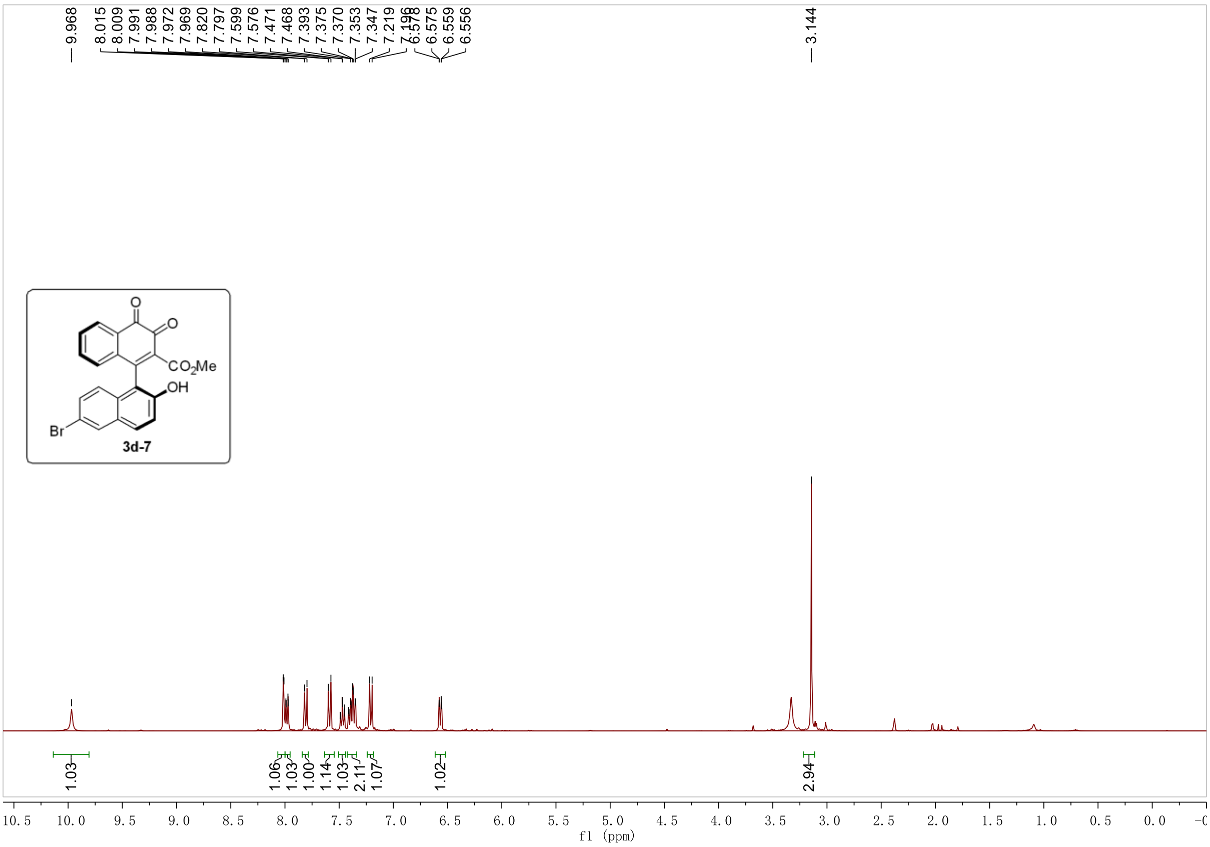

Supplementary Figure 255. ^{13}C NMR of **3d-7**.

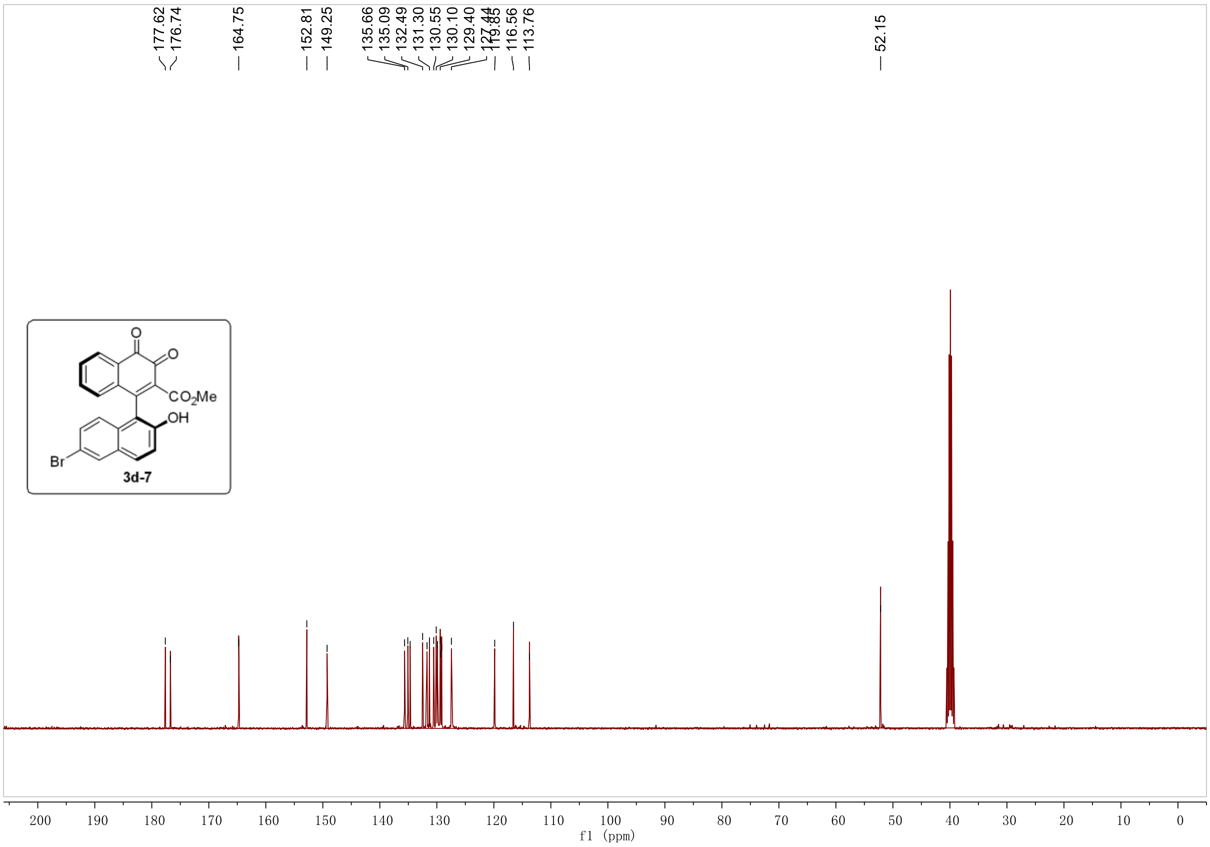

Supplementary Figure 256. ^{1}H NMR of **3e-0**.

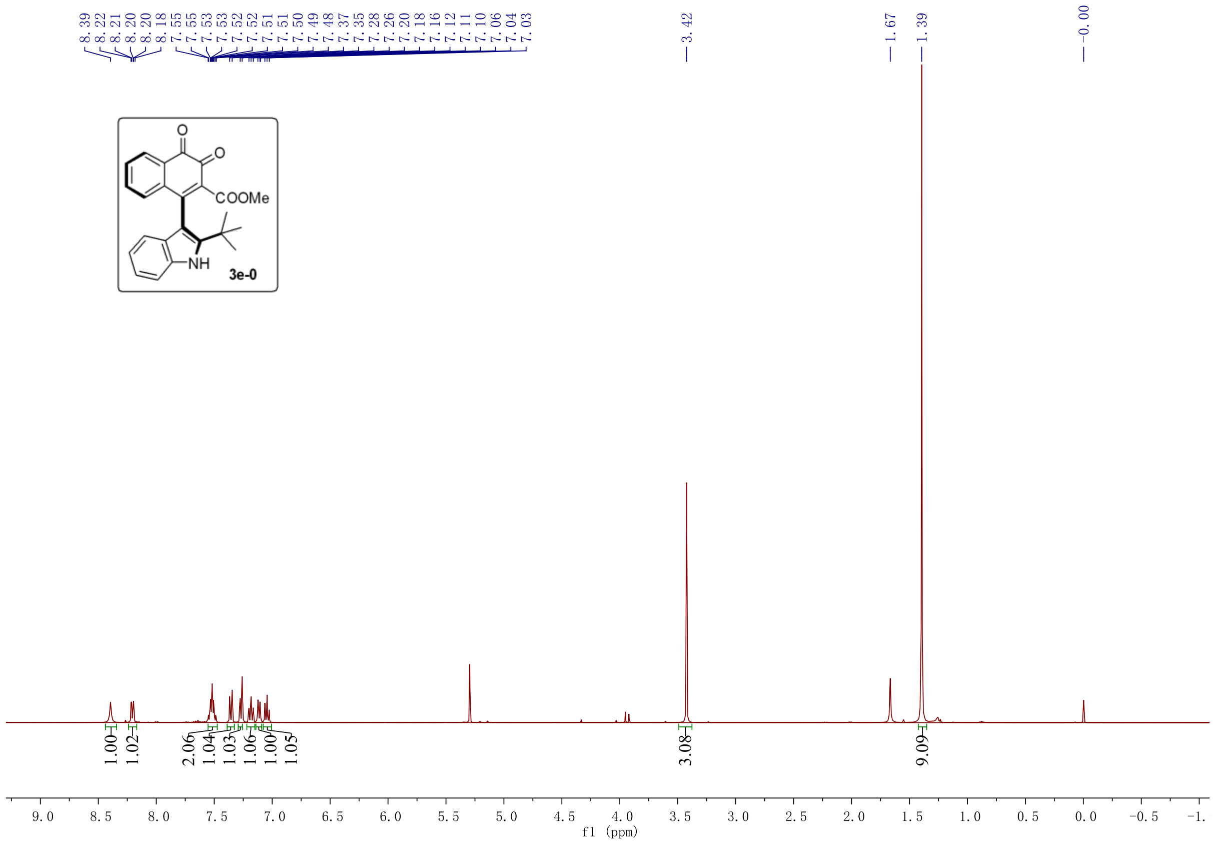

Supplementary Figure 257. ^{13}C NMR of **3e-0**.

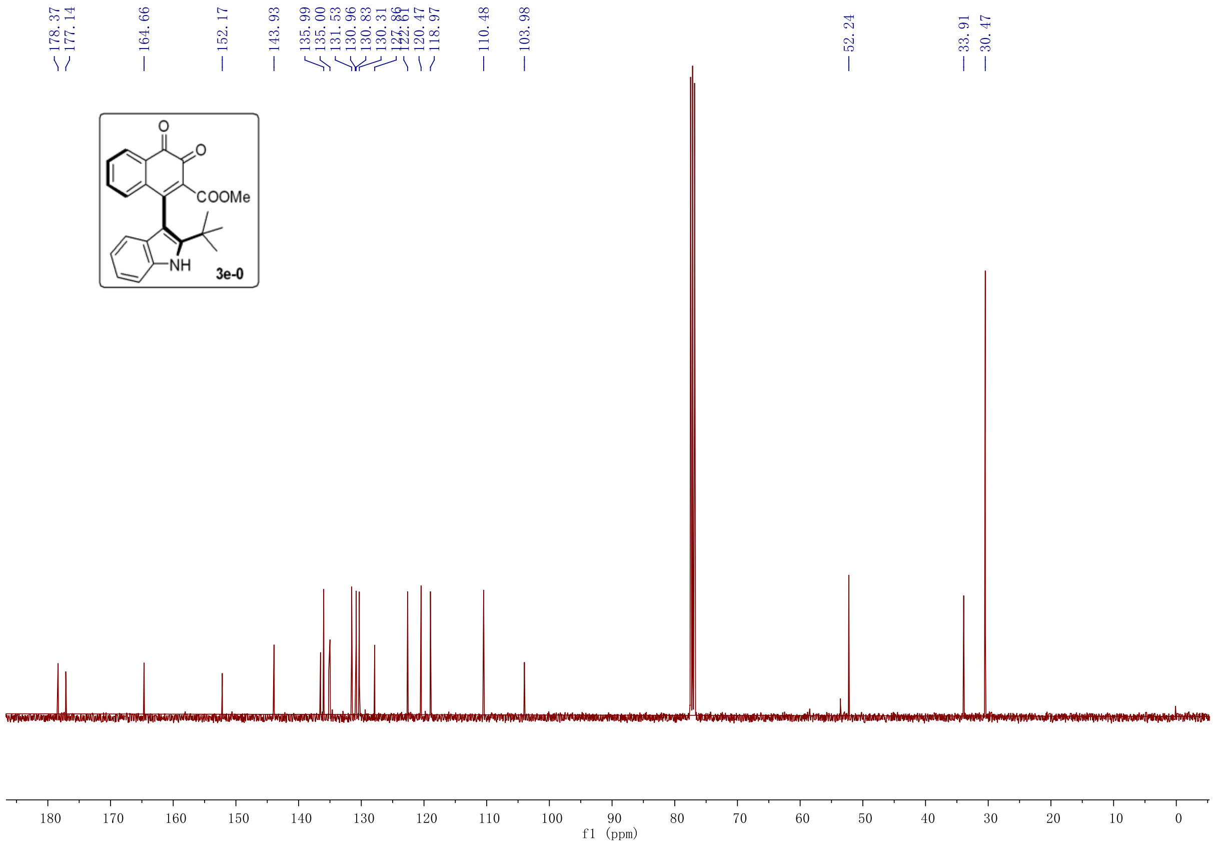

Supplementary Figure 258. ^{1}H NMR of **3e-1**.

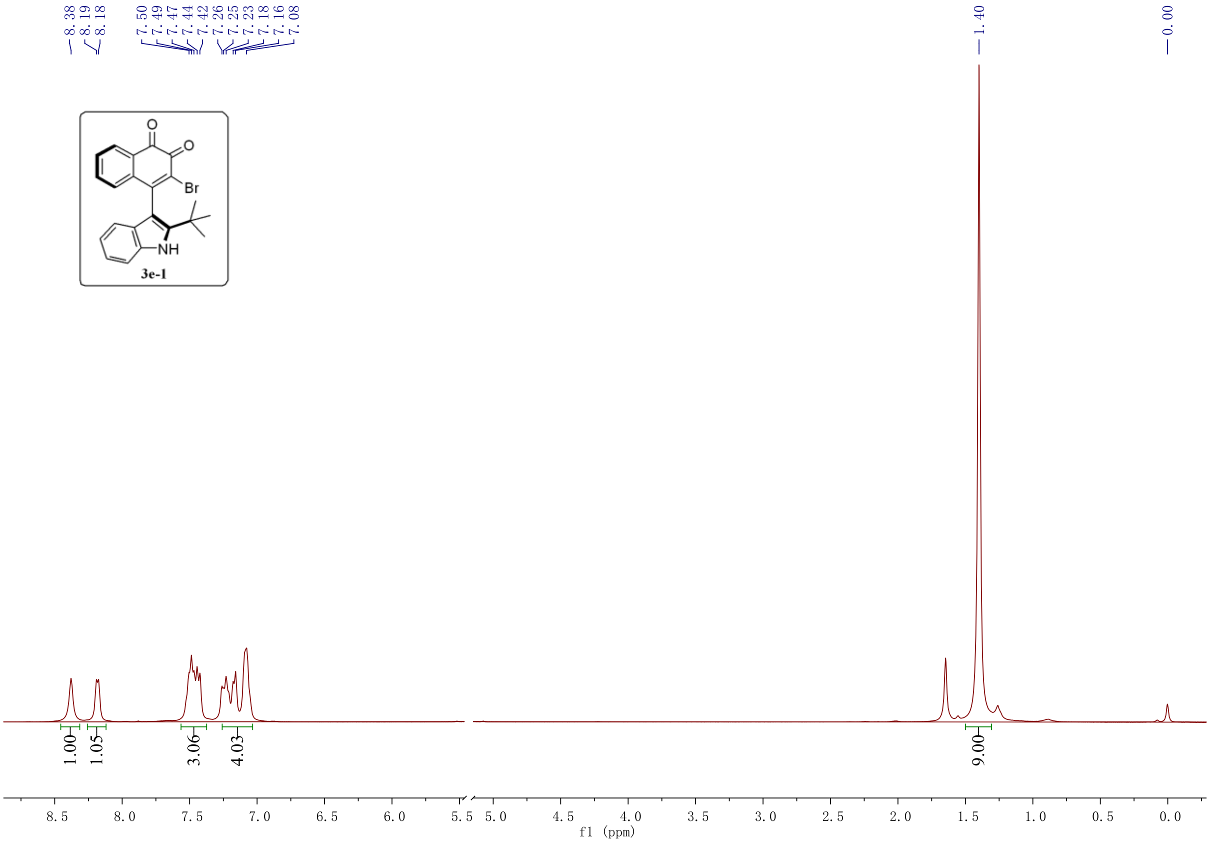

Supplementary Figure 259. ^{13}C NMR of **3e-1**.

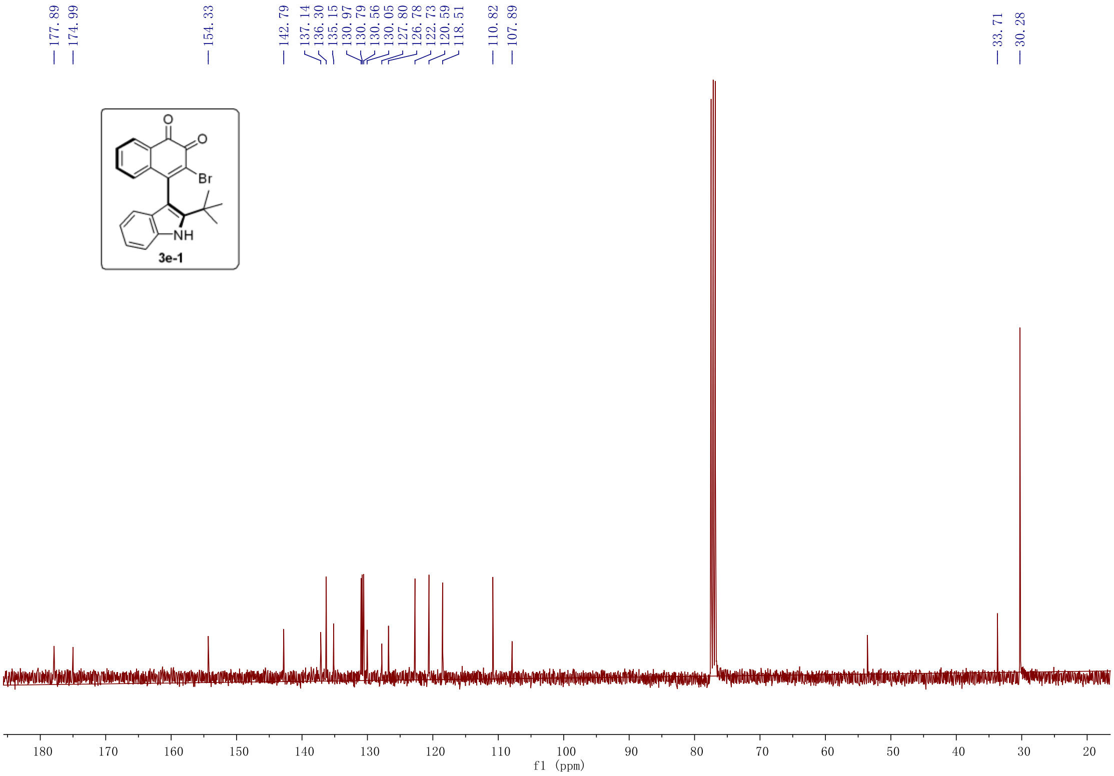

Supplementary Figure 260. ^{1}H NMR of **3e-2**.

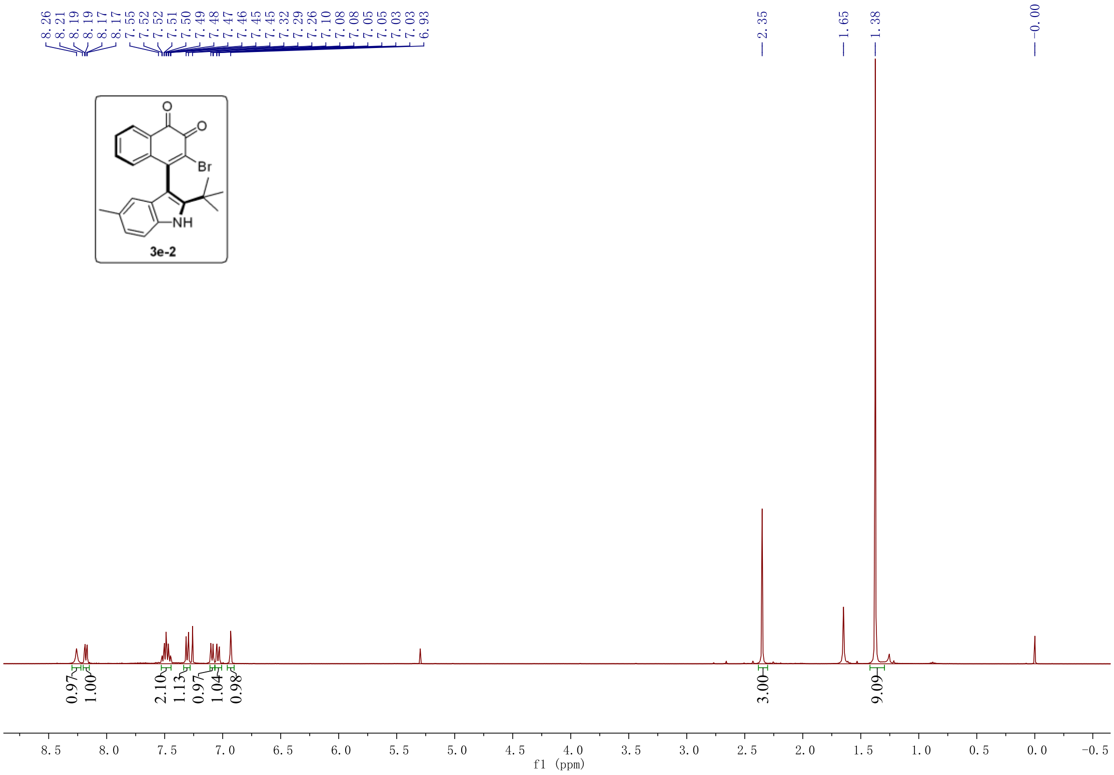

Supplementary Figure 261. ^{13}C NMR of **3e-2**.

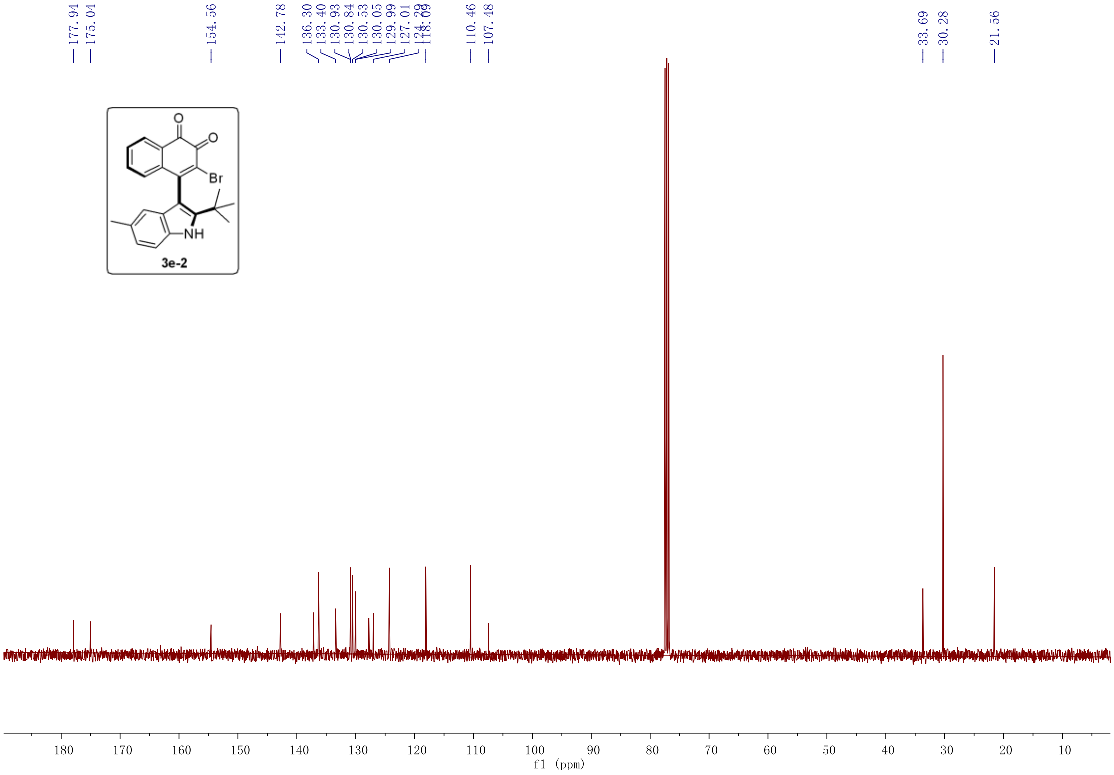

Supplementary Figure 262. ^{1}H NMR of **3e-3**.

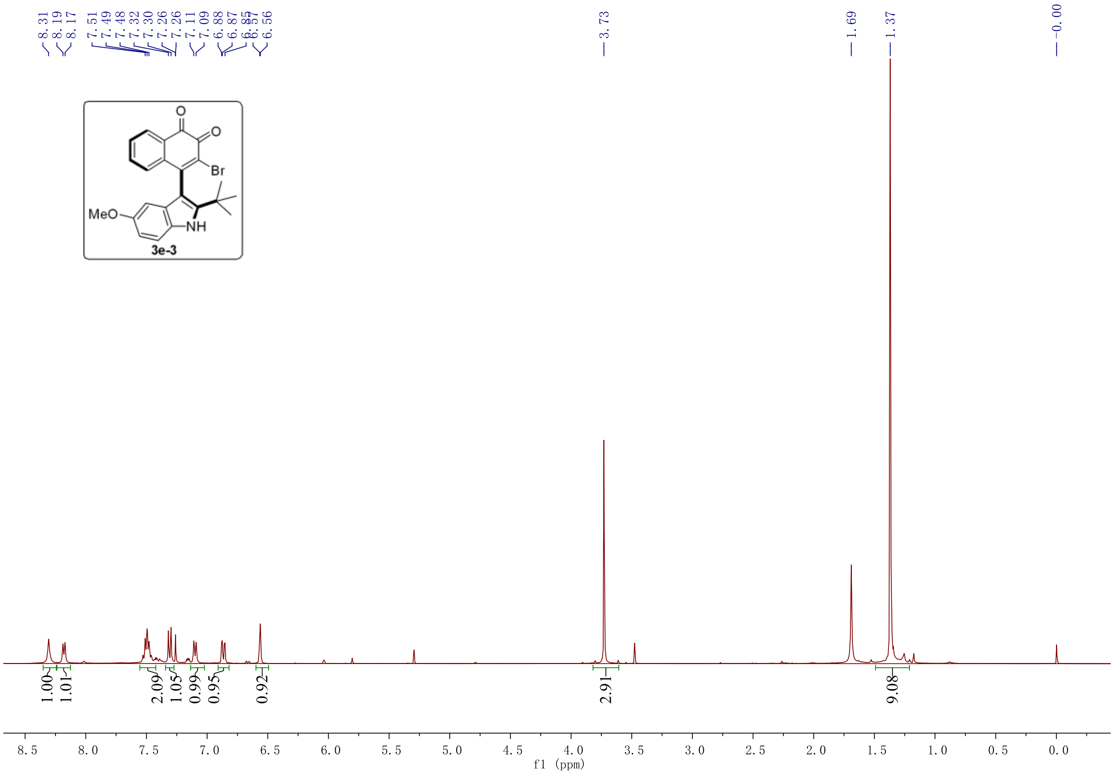

Supplementary Figure 263. ^{13}C NMR of **3e-3**.

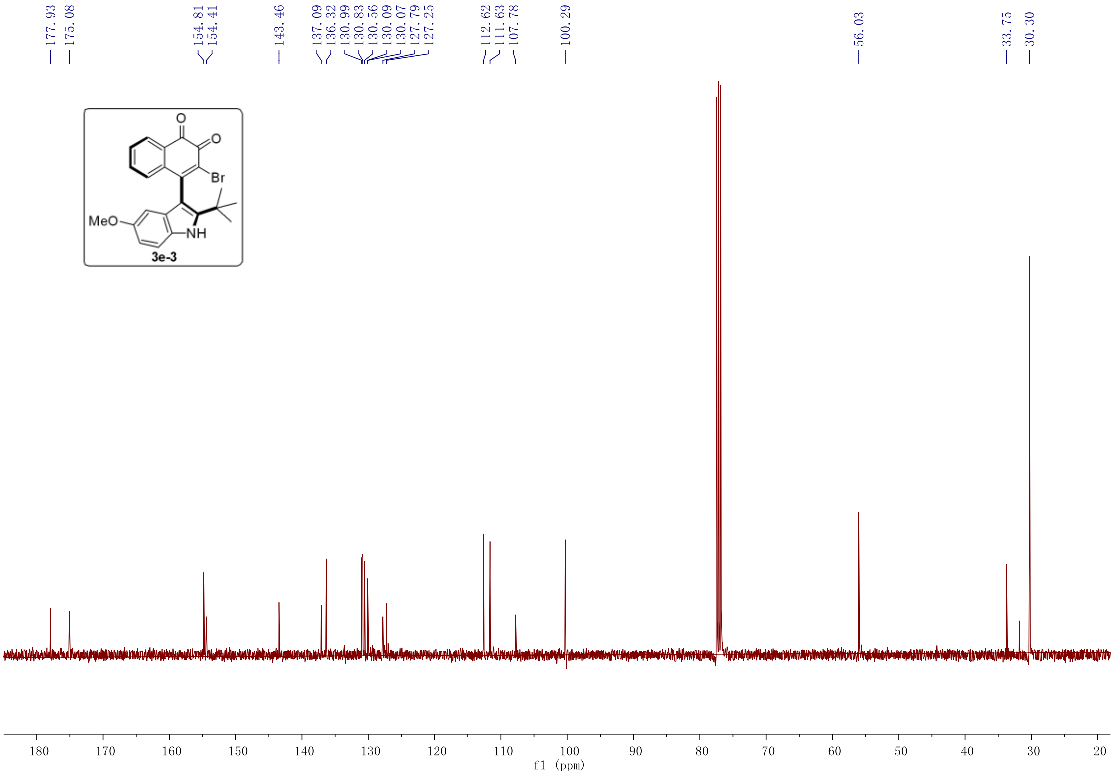

Supplementary Figure 264. ^{1}H NMR of **3e-4**.

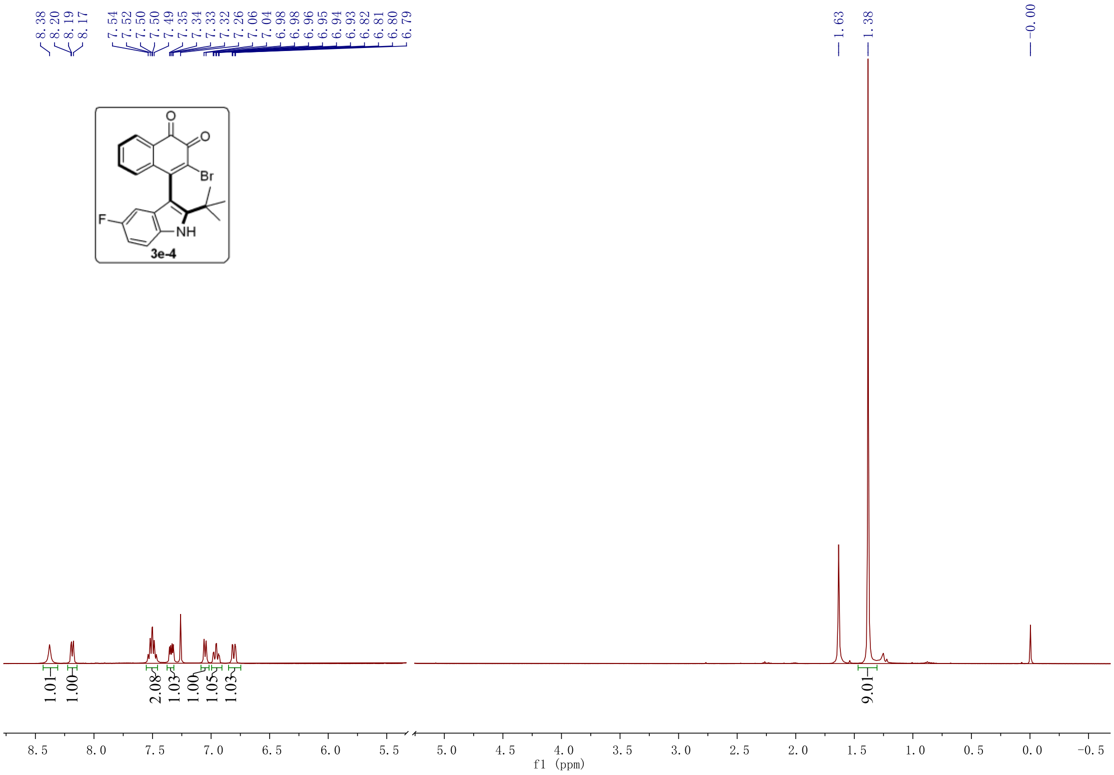

Supplementary Figure 265. ^{13}C NMR of **3e-4**.

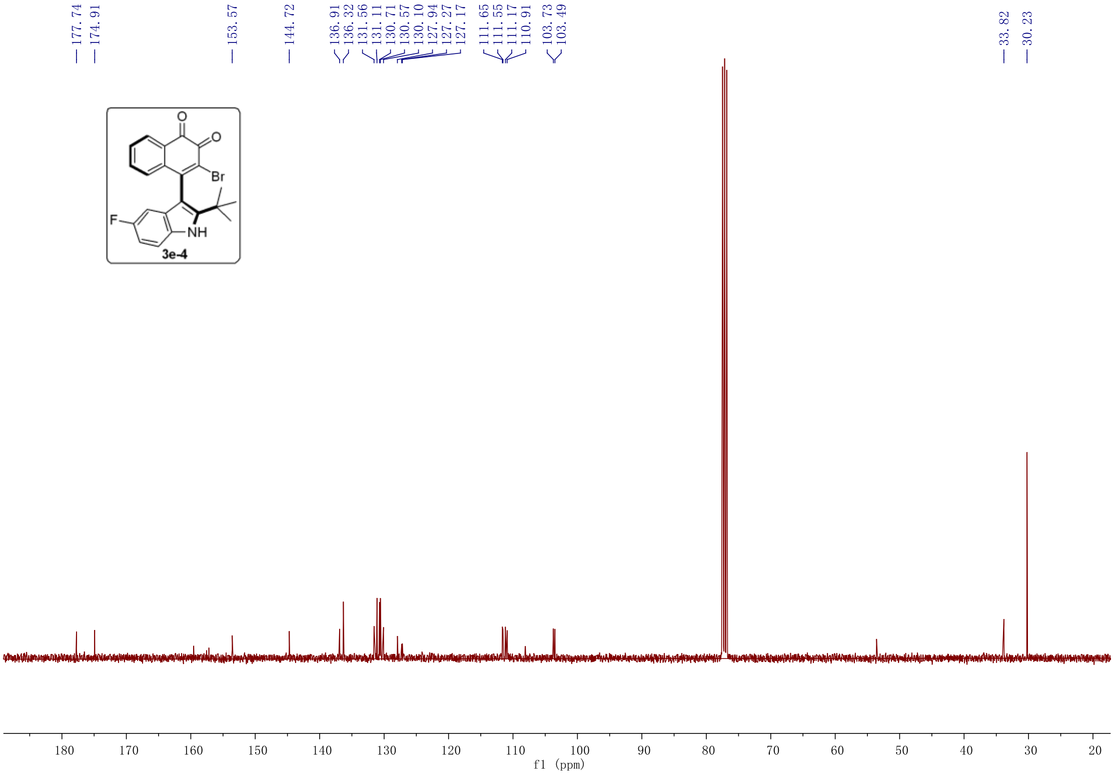

Supplementary Figure 266. ^{1}H NMR of **3e-5**.

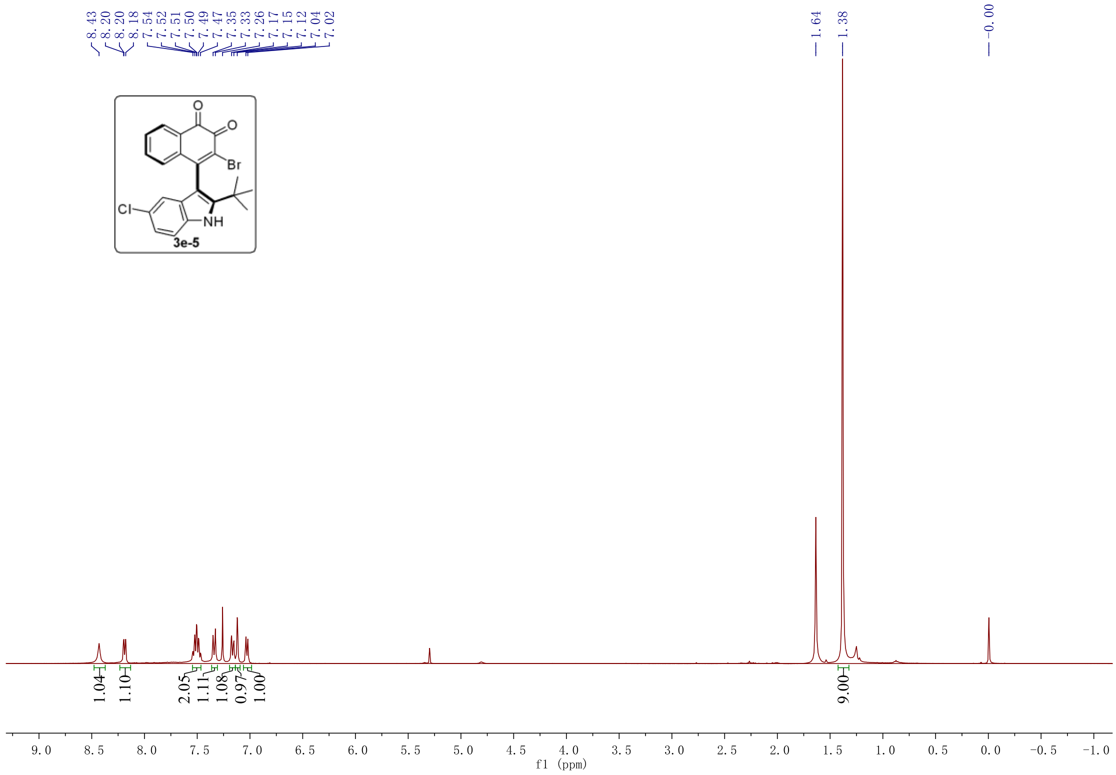

Supplementary Figure 267. ^{13}C NMR of **3e-5**.

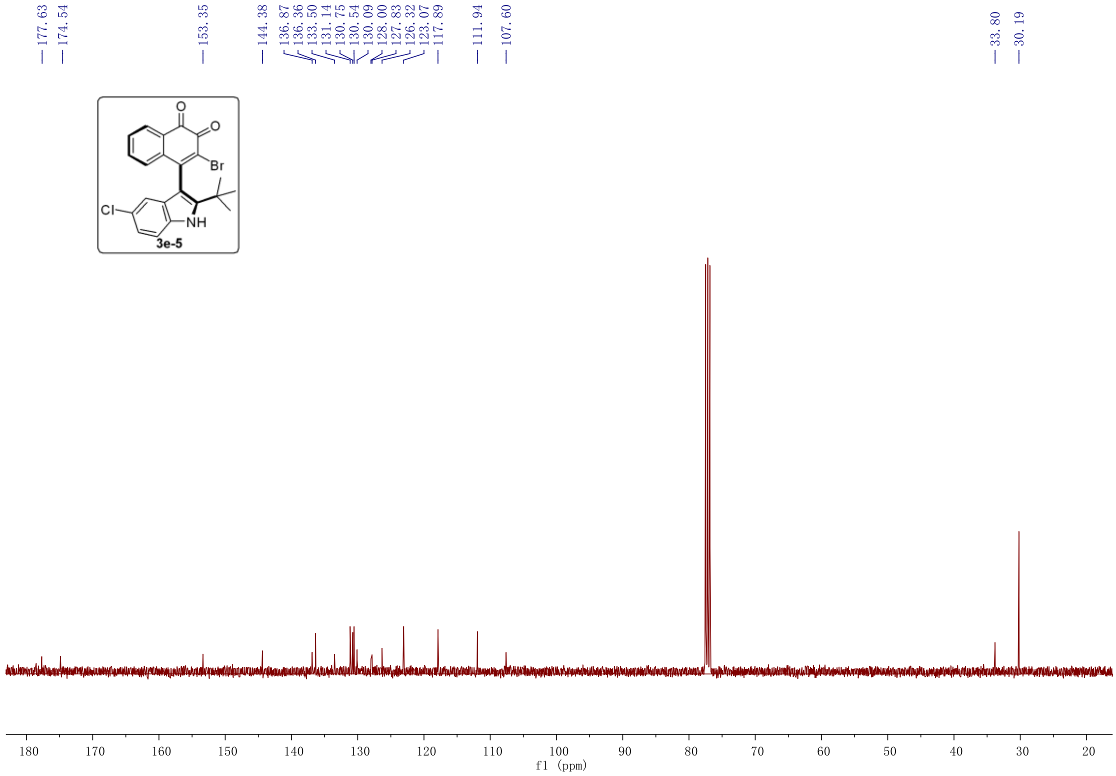

Supplementary Figure 268. ^{1}H NMR of **3e-6**.

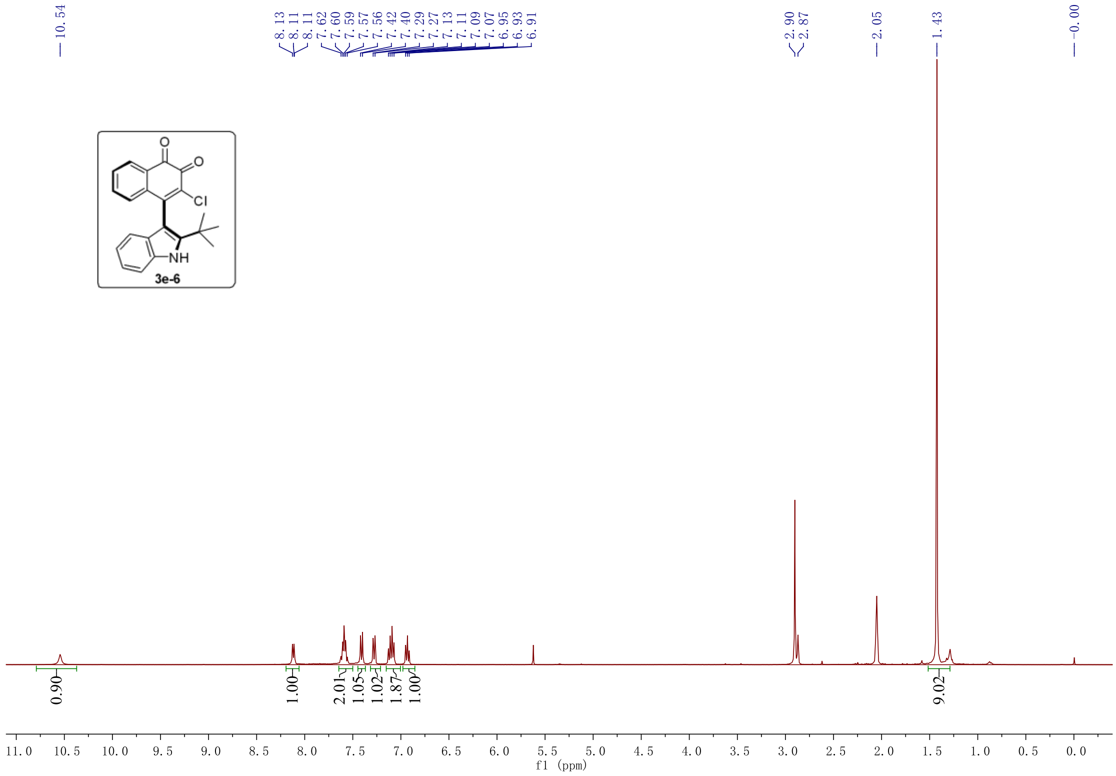

Supplementary Figure 269. ^{13}C NMR of **3e-6**.

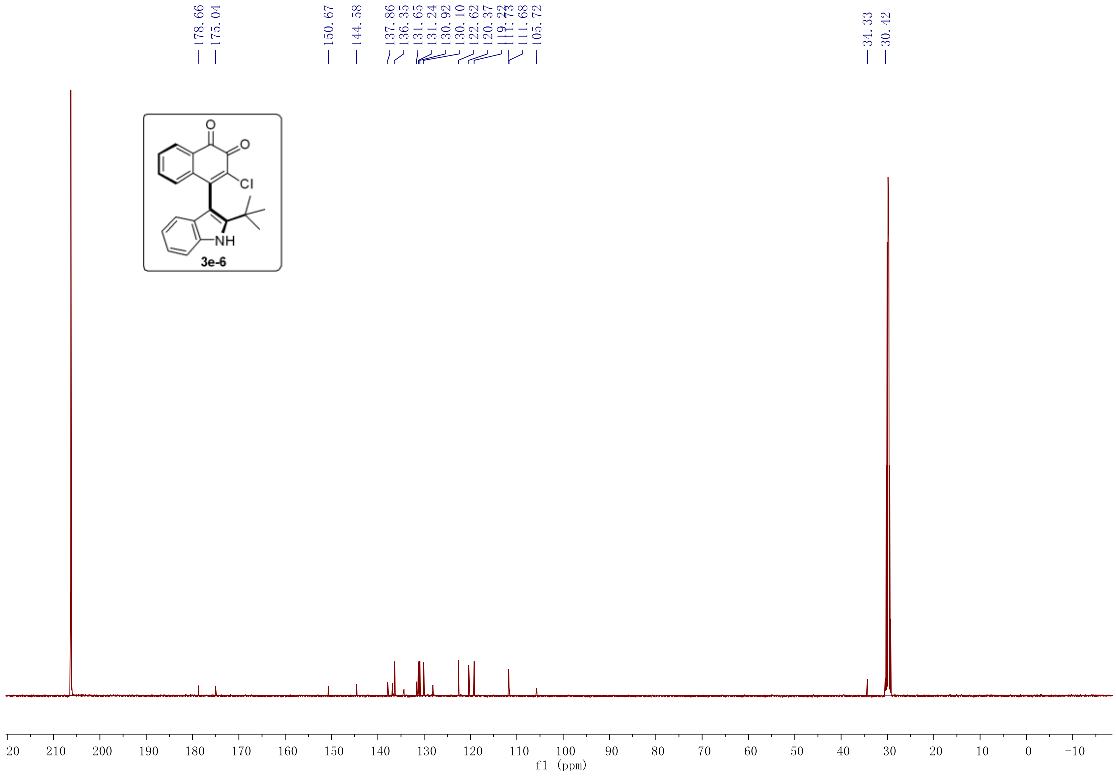

Supplementary Figure 270. ^{1}H NMR of **3e-7**.

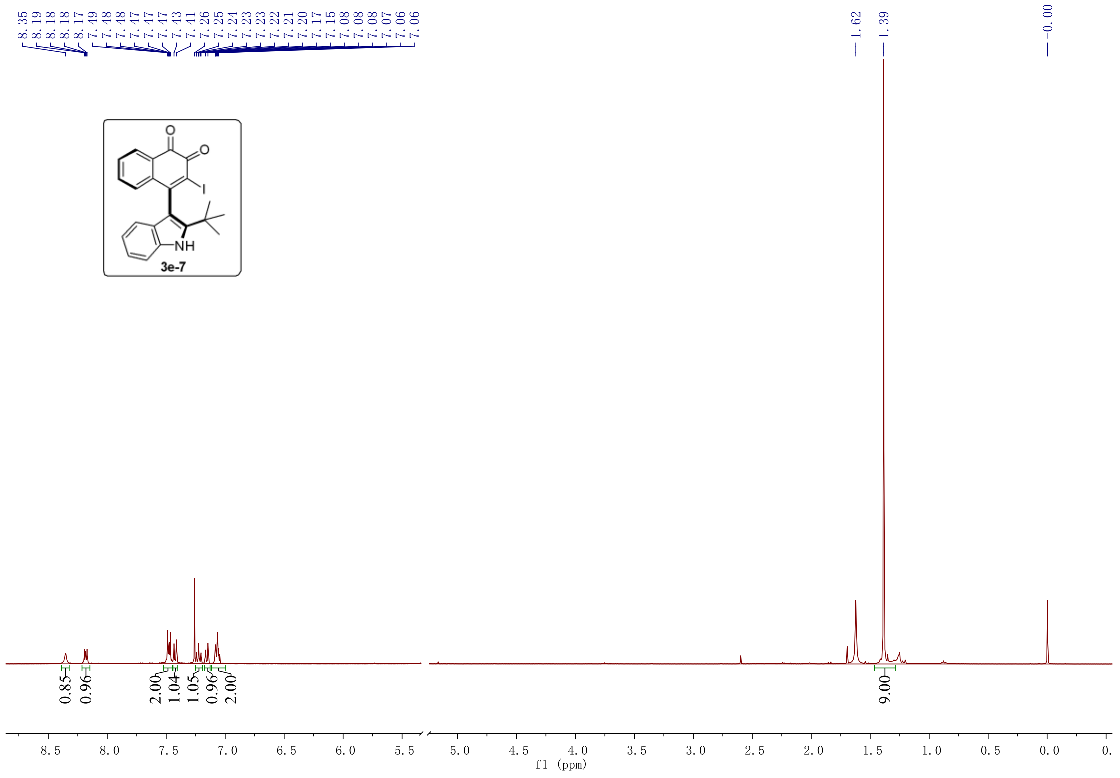

Supplementary Figure 271. ^{13}C NMR of **3e-7**.

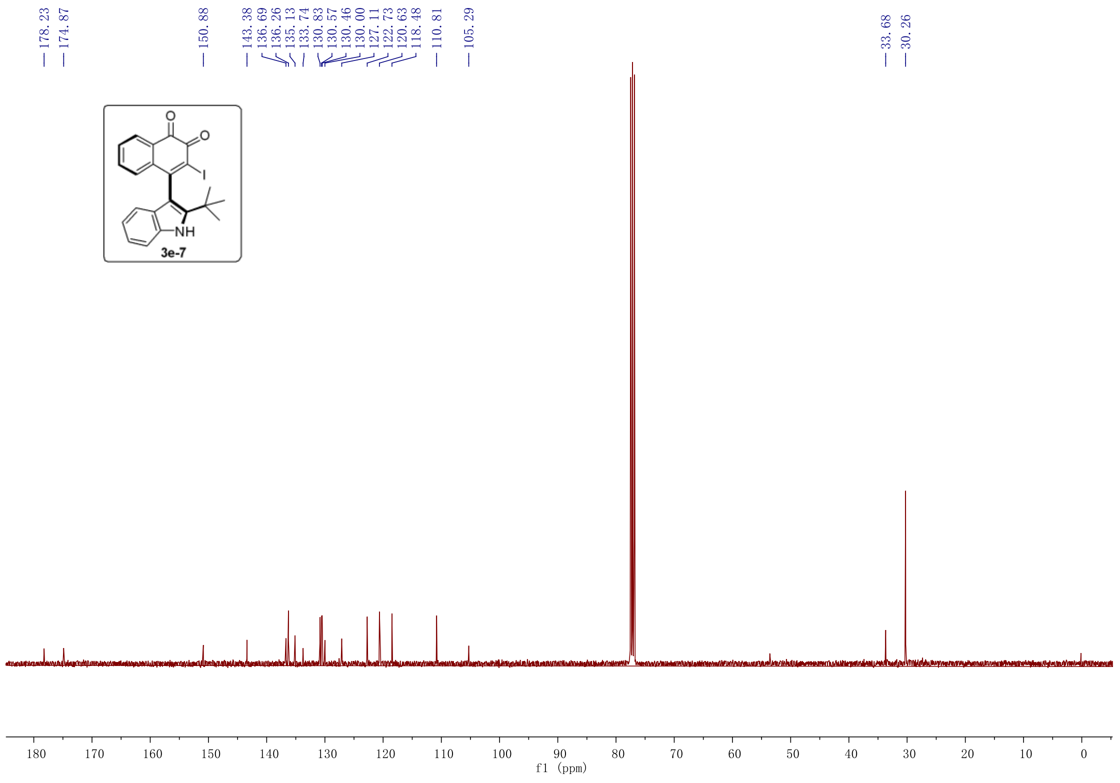

Supplementary Figure 272. ^{1}H NMR of **3e-8**.

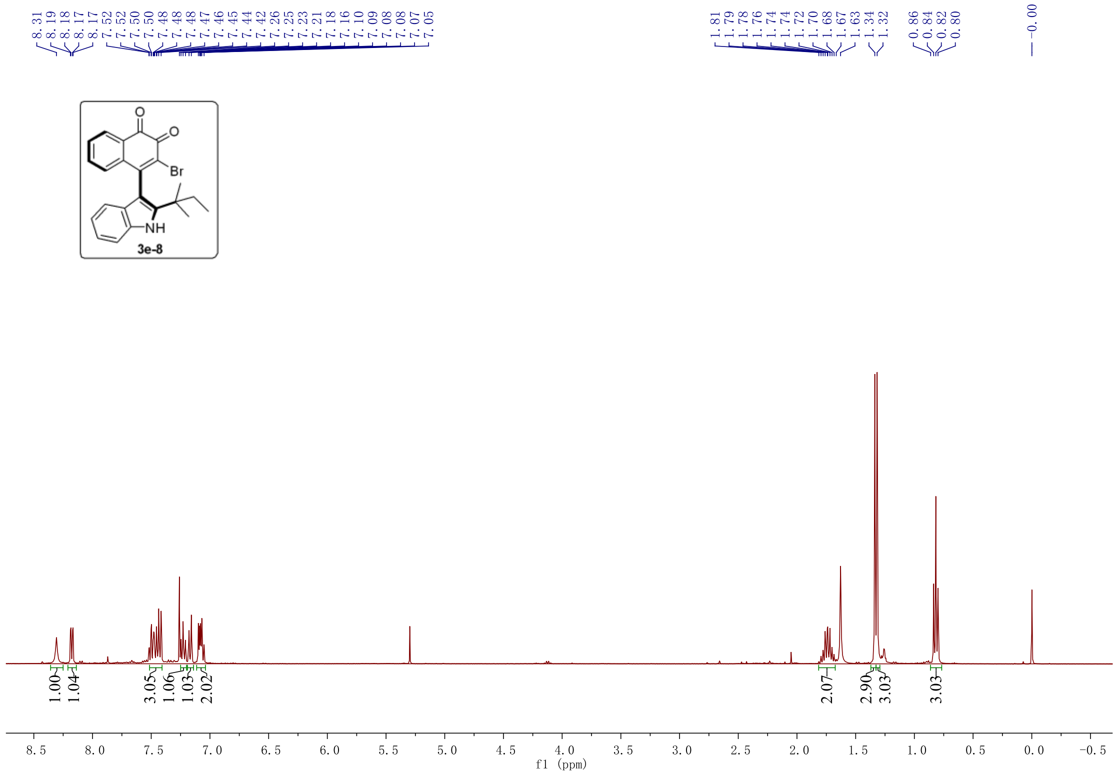

Supplementary Figure 273. ^{13}C NMR of **3e-8**.

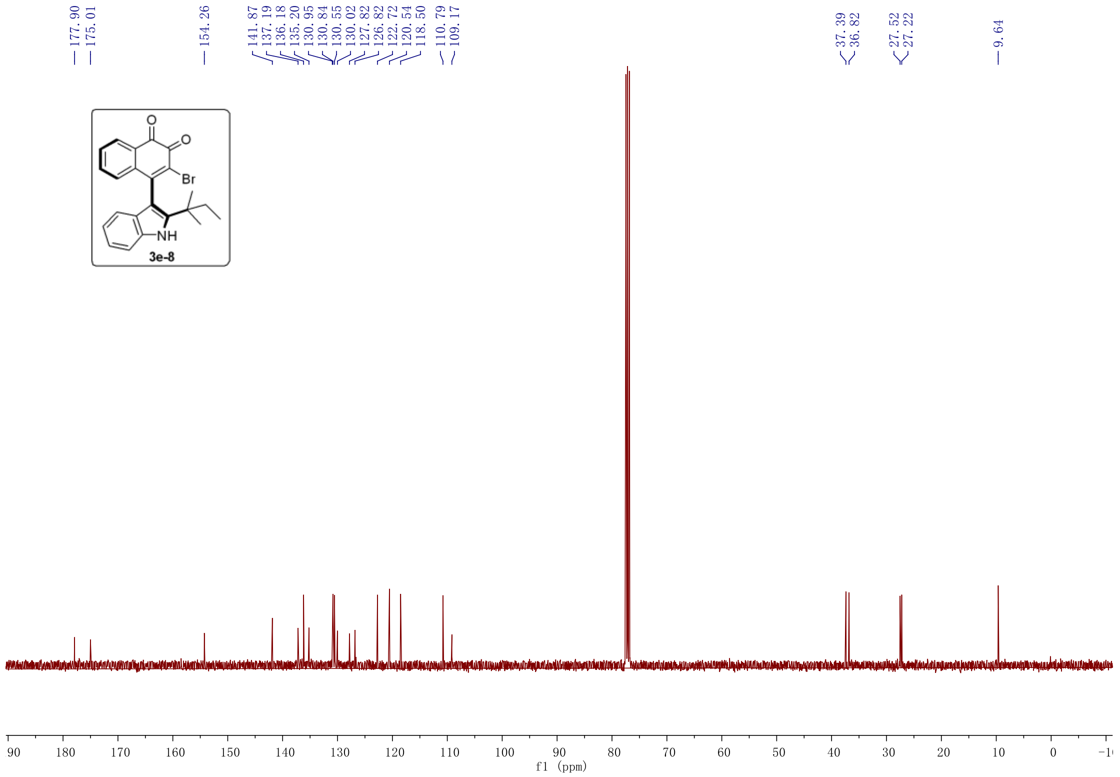

Supplementary Figure 274. ^{1}H NMR of **3e-9**.

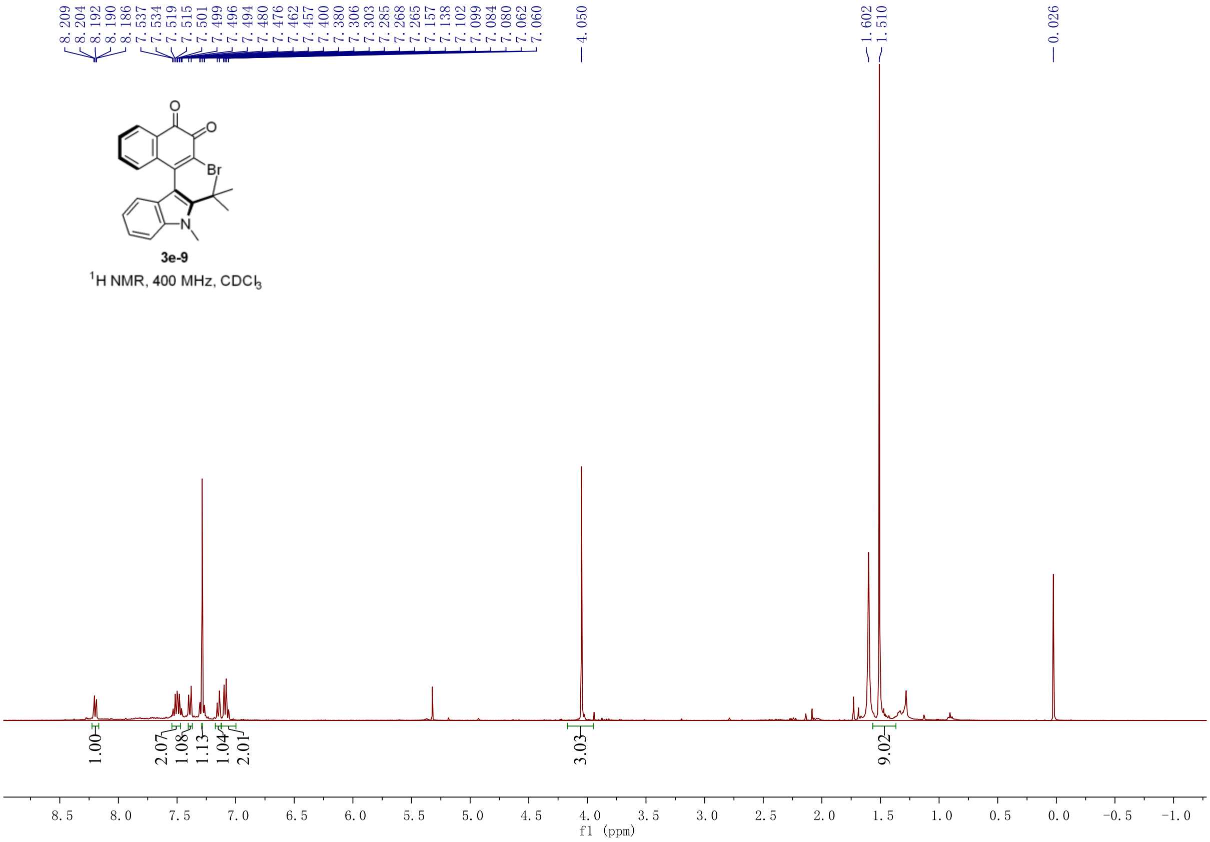

Supplementary Figure 275. ^{13}C NMR of **3e-9**.

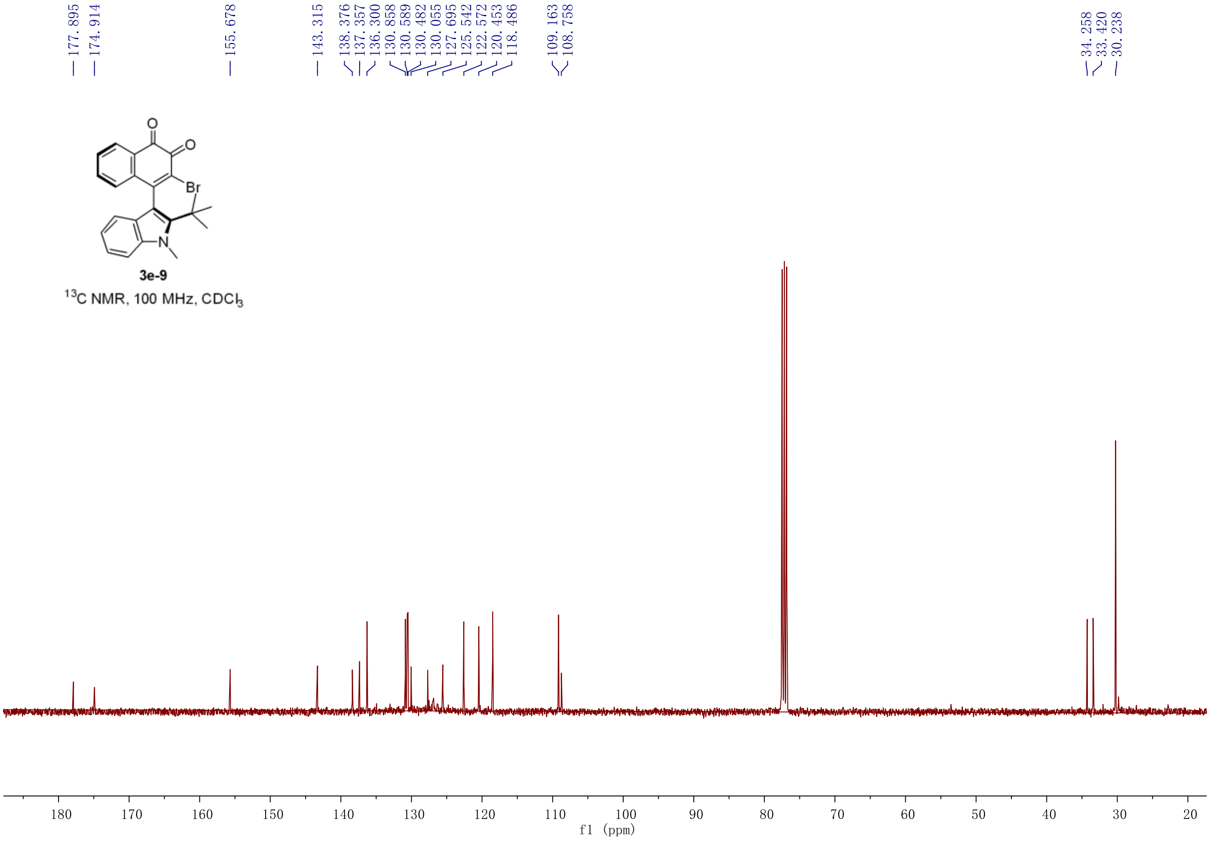

Supplementary Figure 276. ^{1}H NMR of **4b-1**.

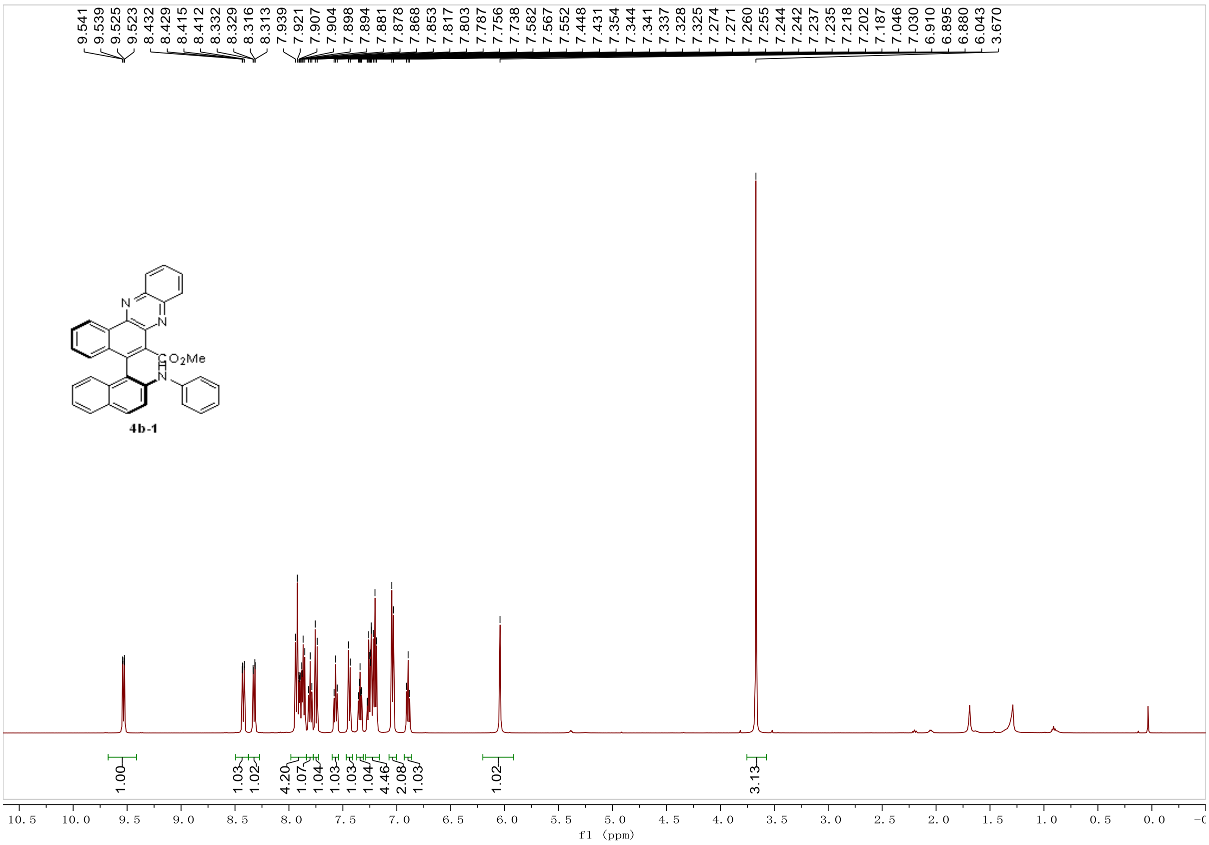

Supplementary Figure 277. ^{13}C NMR of **4b-1**.

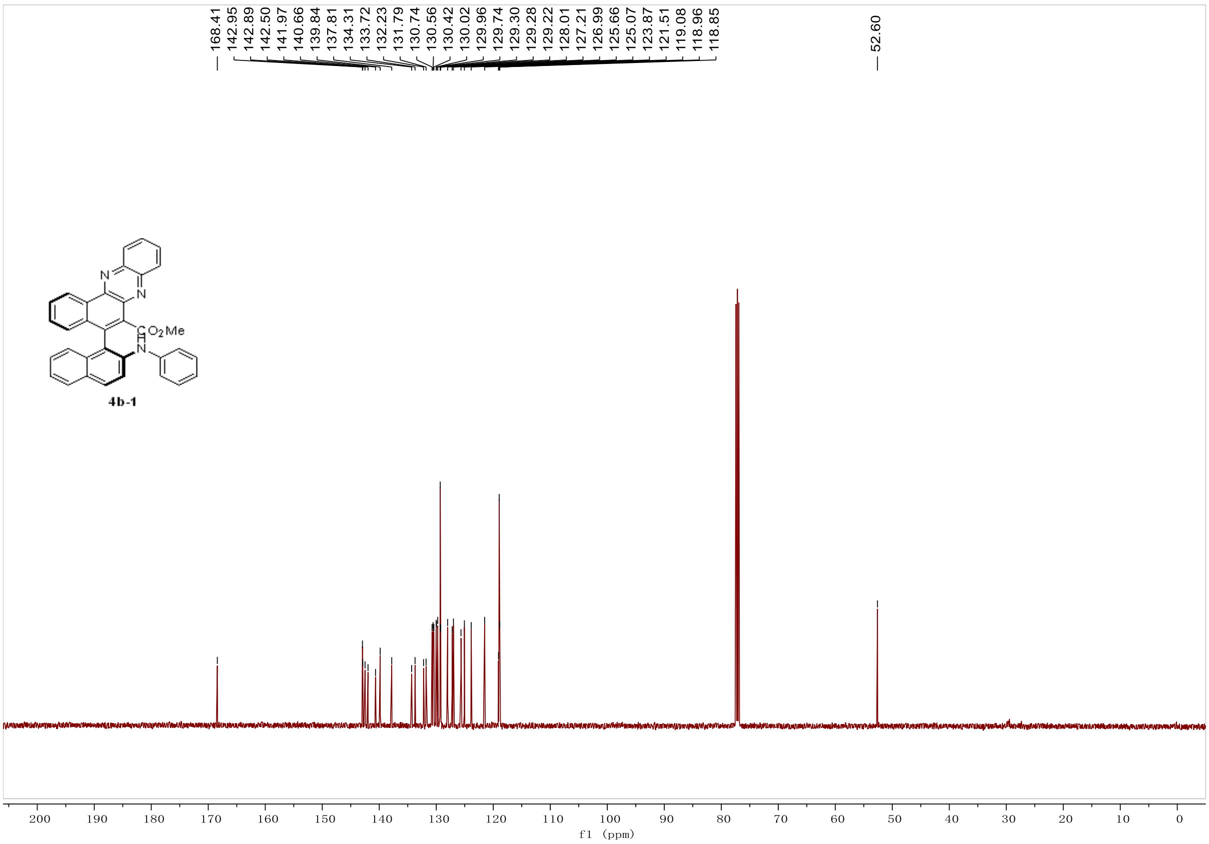

Supplementary Figure 278. ^{1}H NMR of **4b-7**.

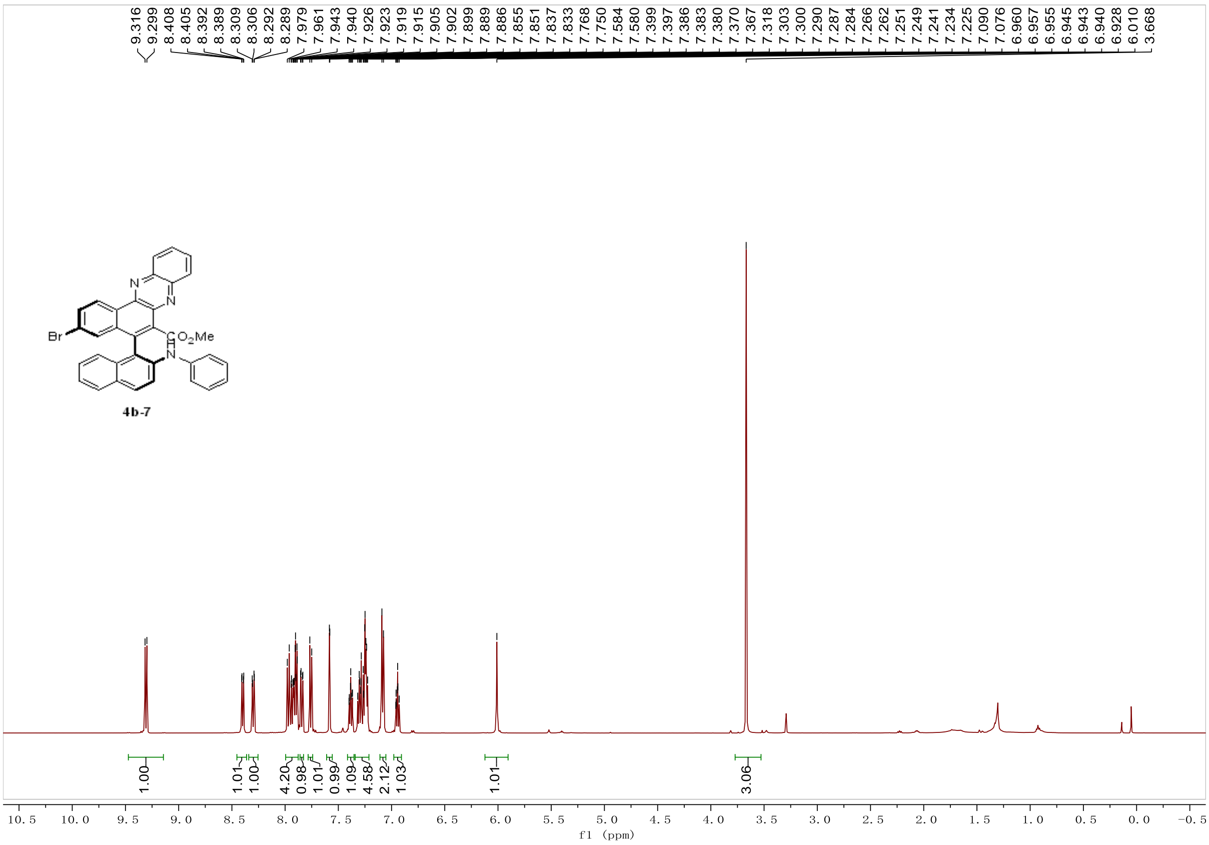

Supplementary Figure 279. ^{13}C NMR of **4b-7**.

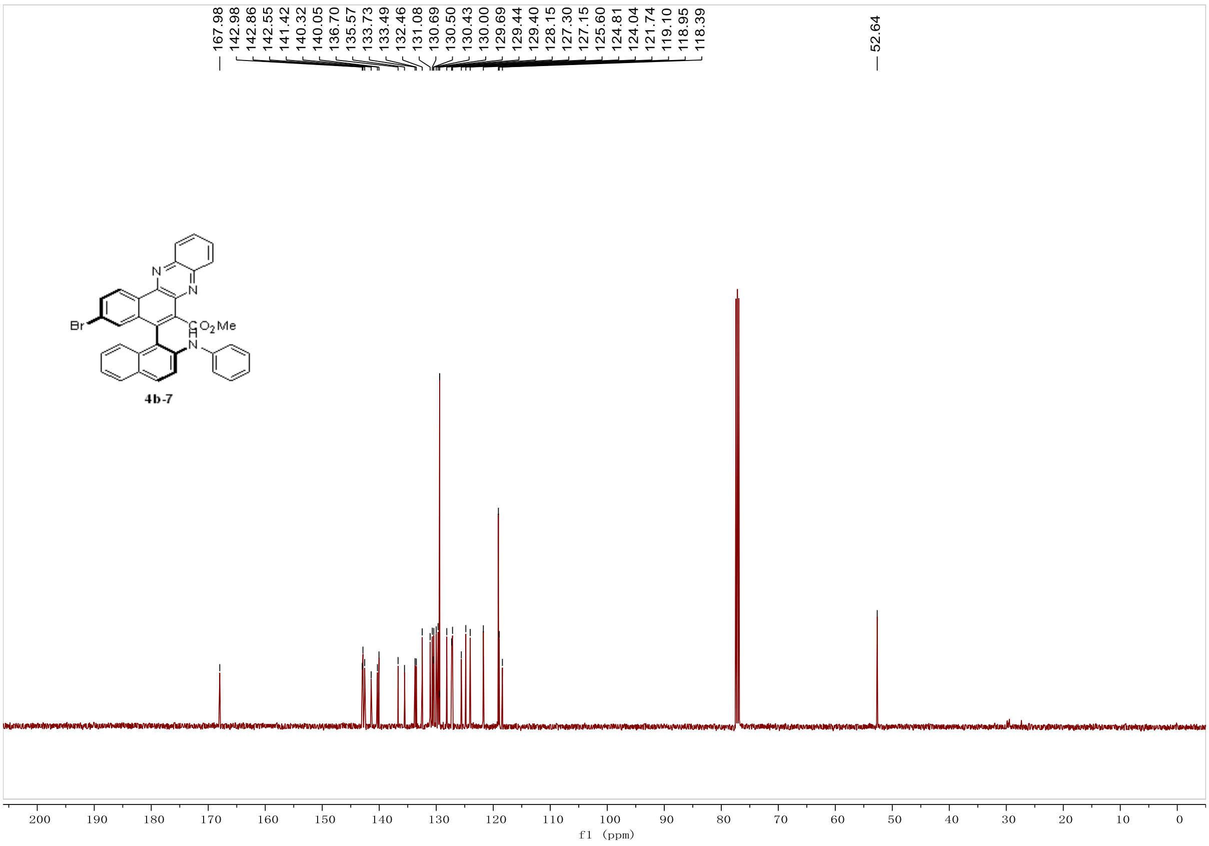

Supplementary Figure 280. ^{1}H NMR of **4b-12**.

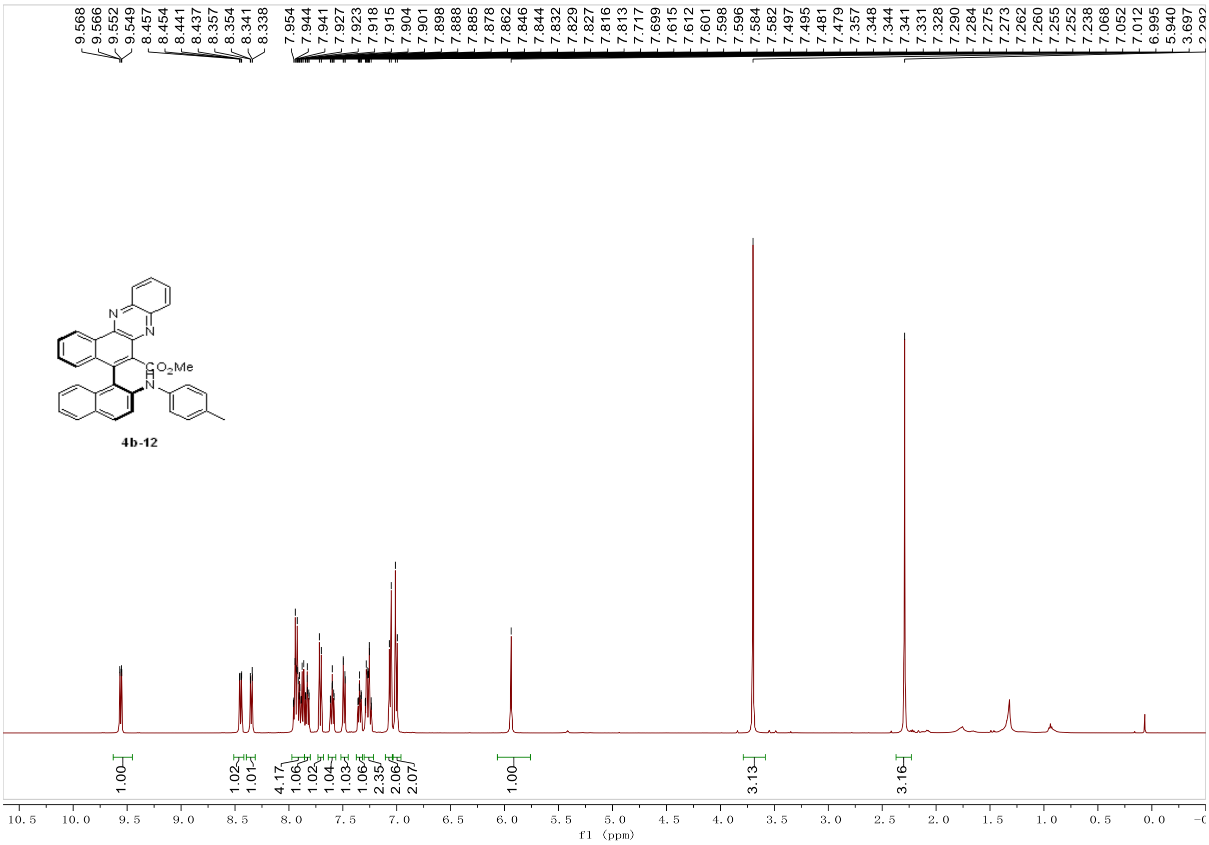

Supplementary Figure 281. ^{13}C NMR of **4b-12**.

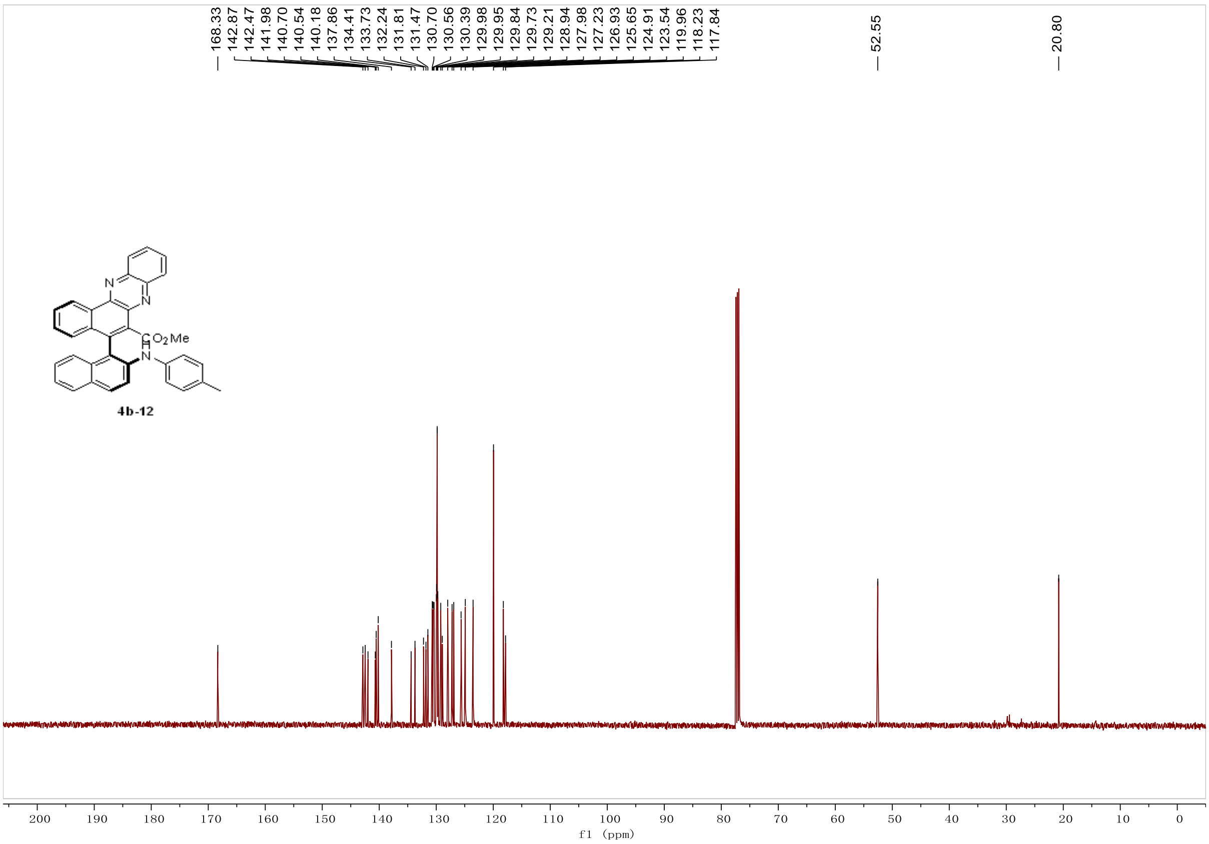

Supplementary Figure 282. ¹H NMR of **4b-14**.

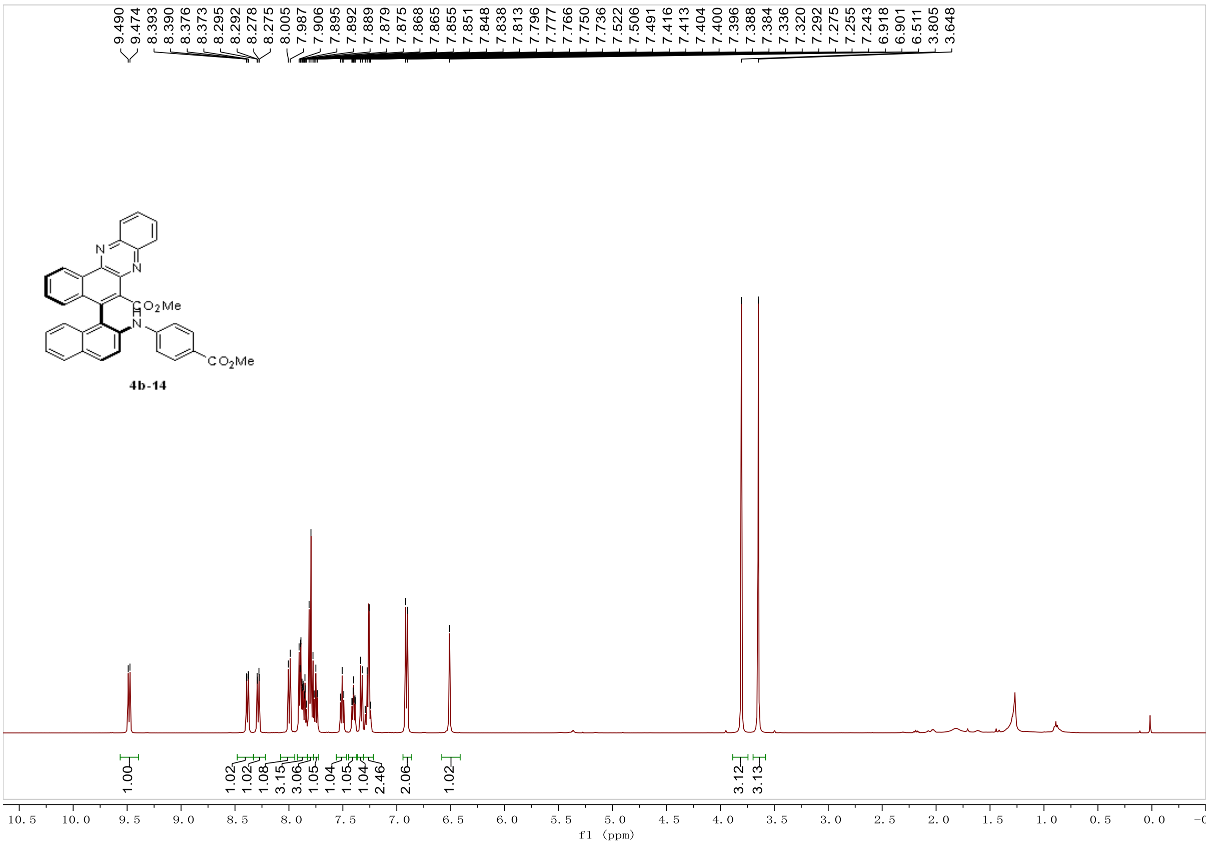

Supplementary Figure 283. ¹³C NMR of **4b-14**.

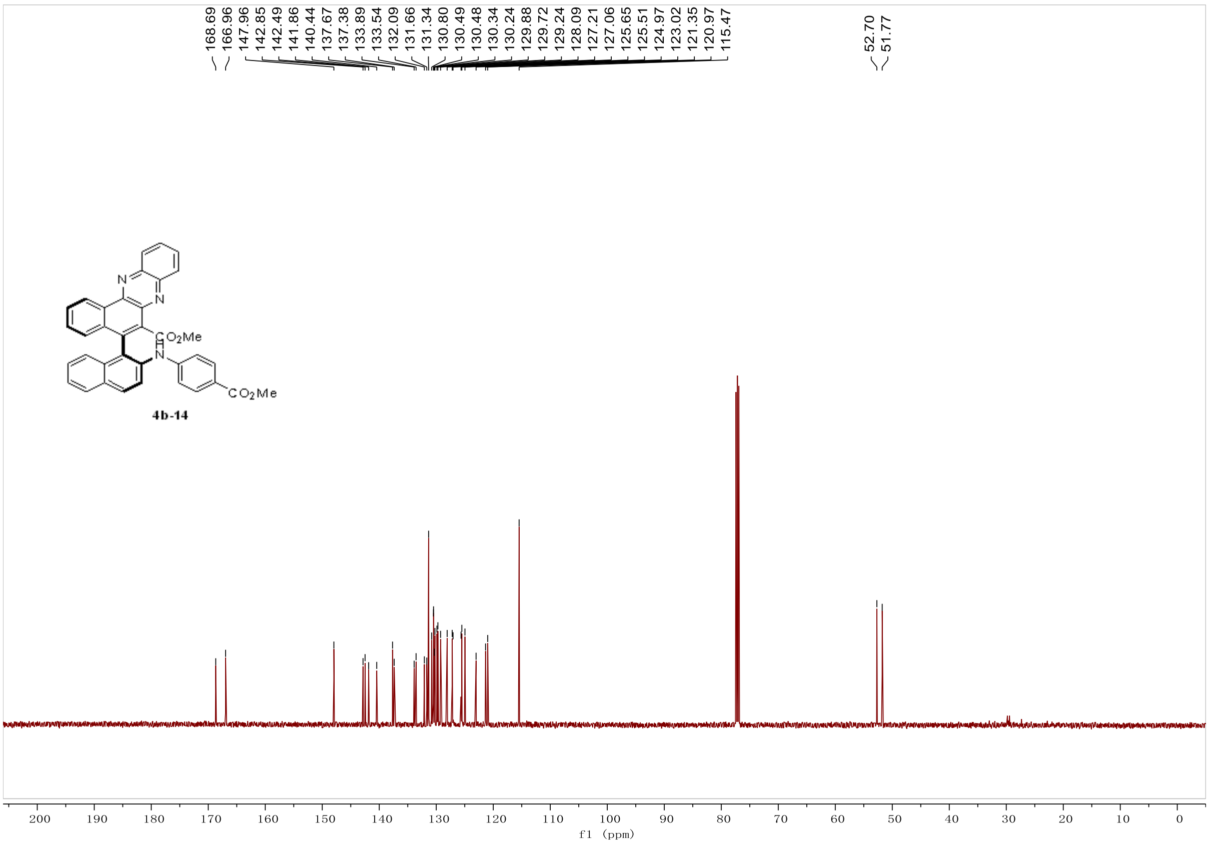

Supplementary Figure 284. ^{1}H NMR of **5b-1**.

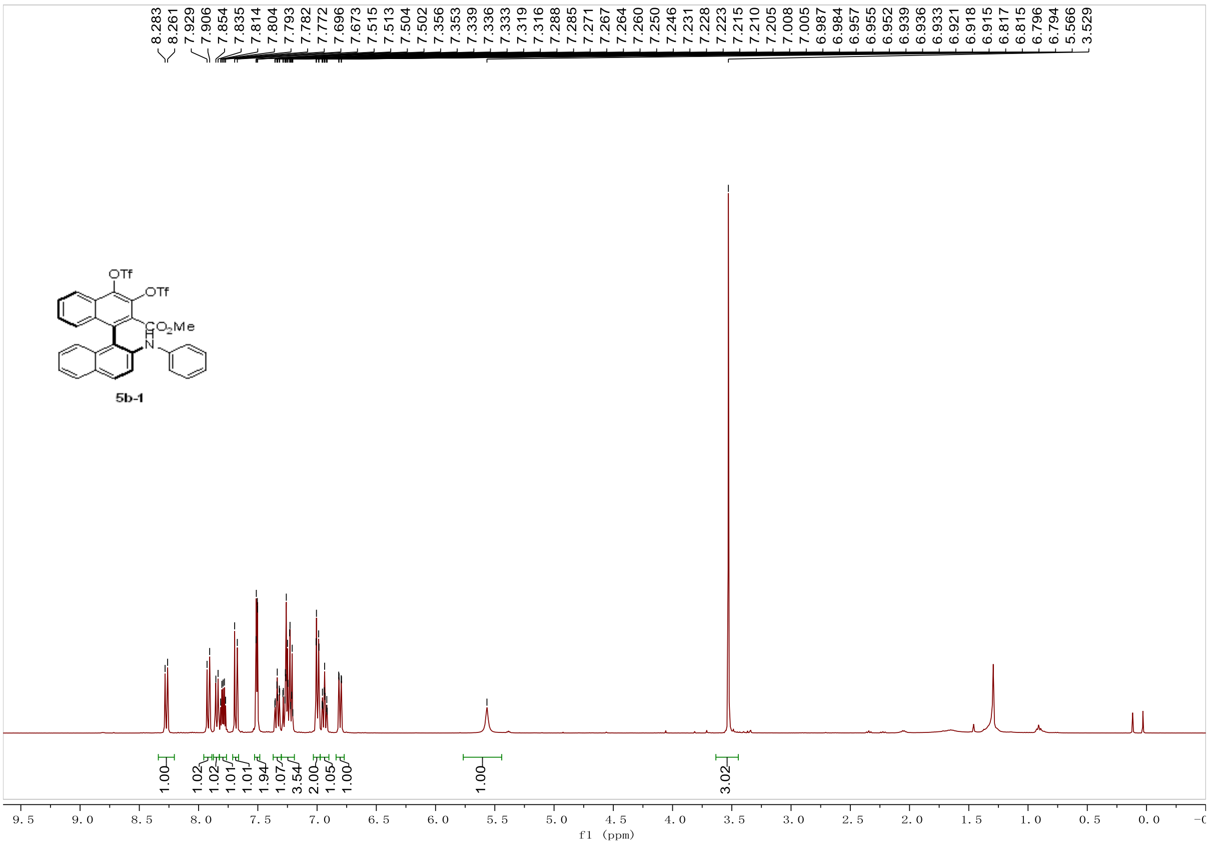

Supplementary Figure 285. ^{13}C NMR of **5b-1**.

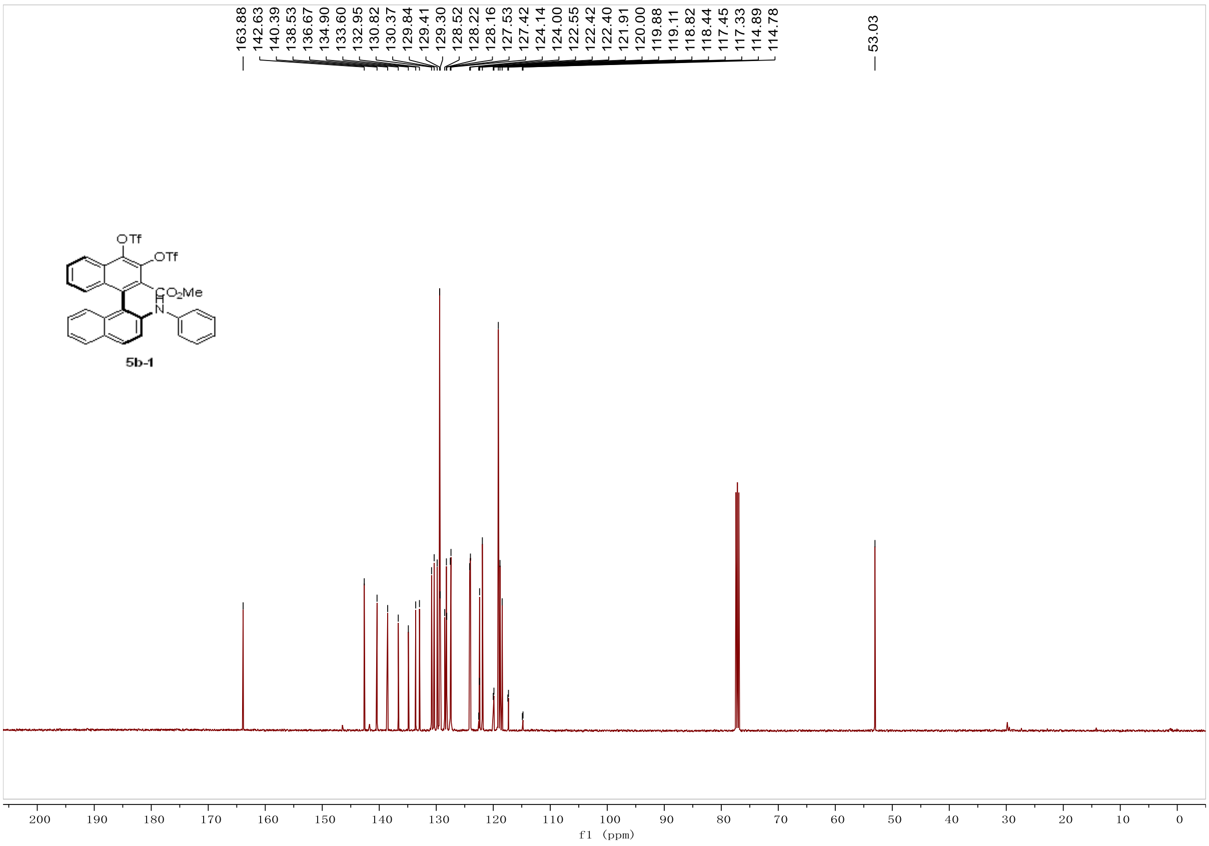

Supplementary Figure 286. ^{19}F NMR of **5b-1**.

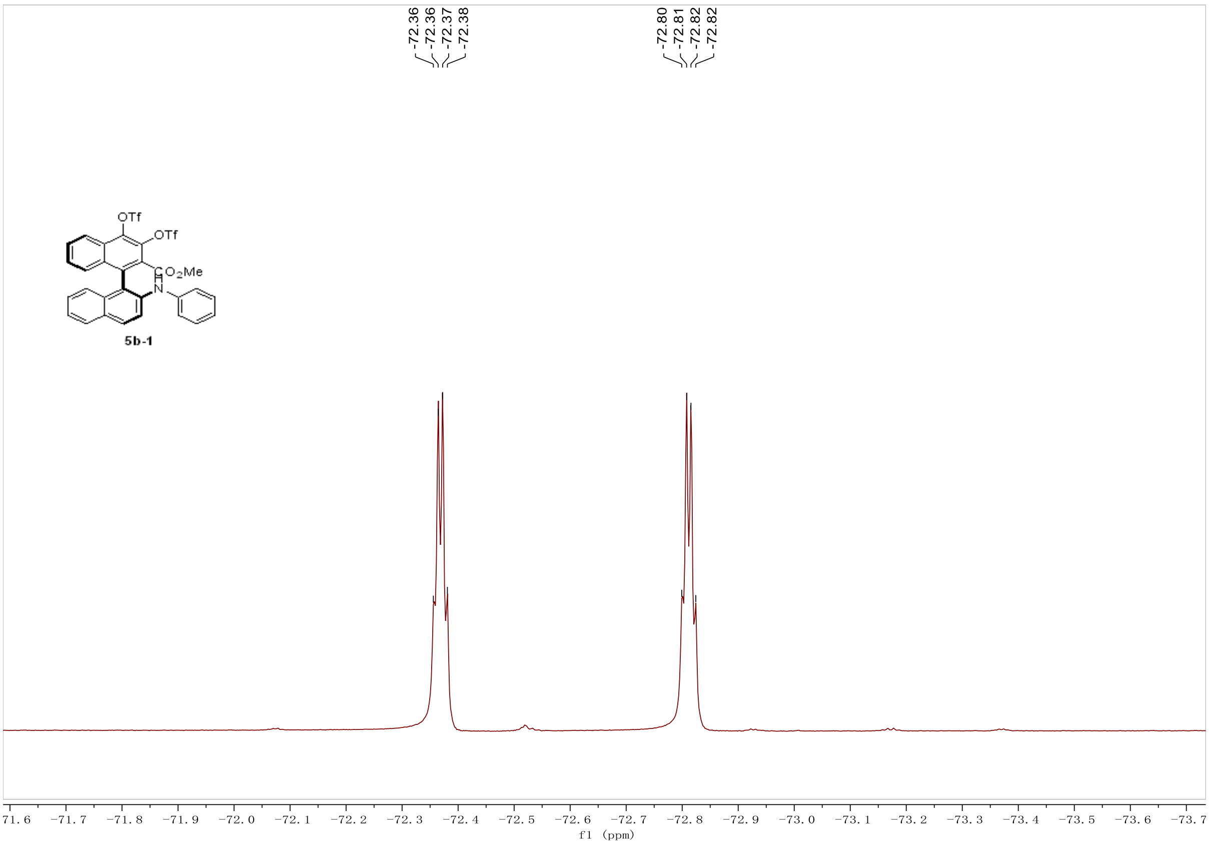

Supplementary Figure 287. ^{1}H NMR of **5b-7**.

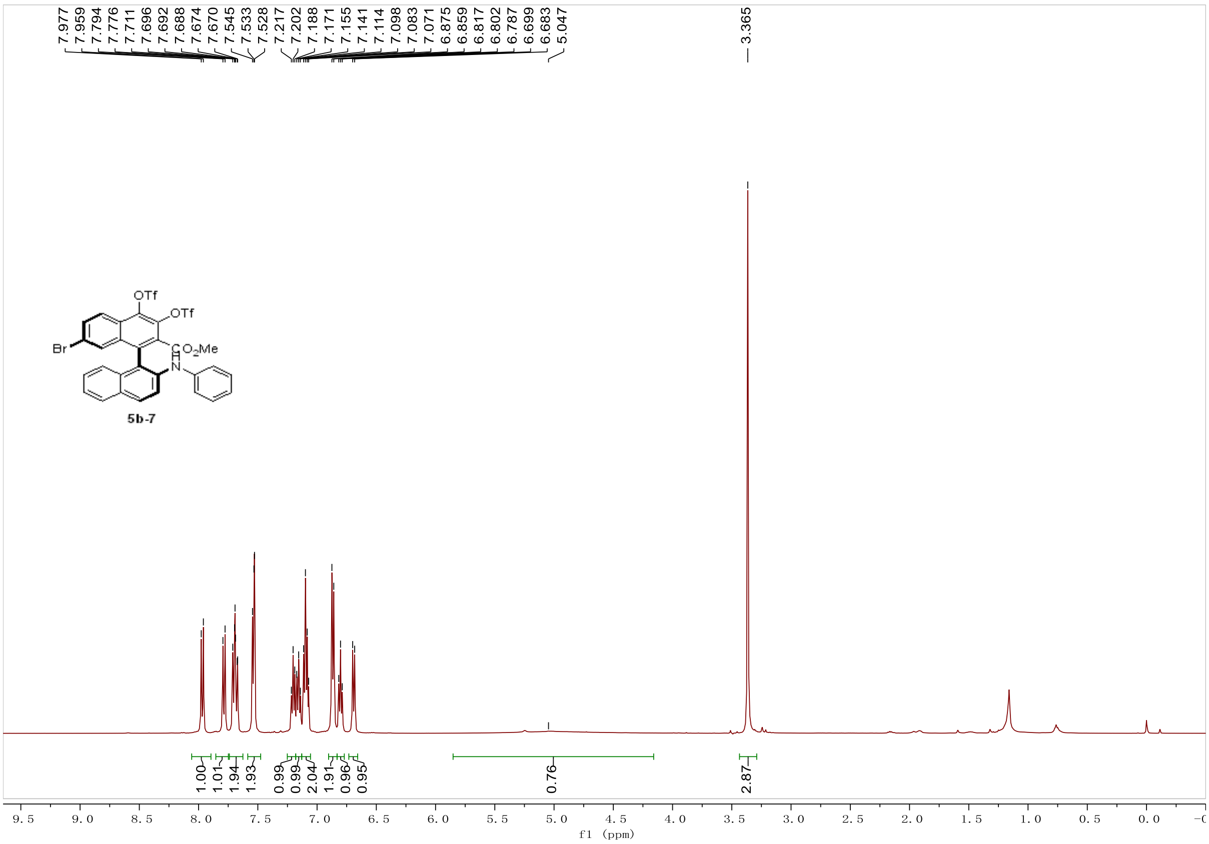

Supplementary Figure 288. ^{13}C NMR of **5b-7**.

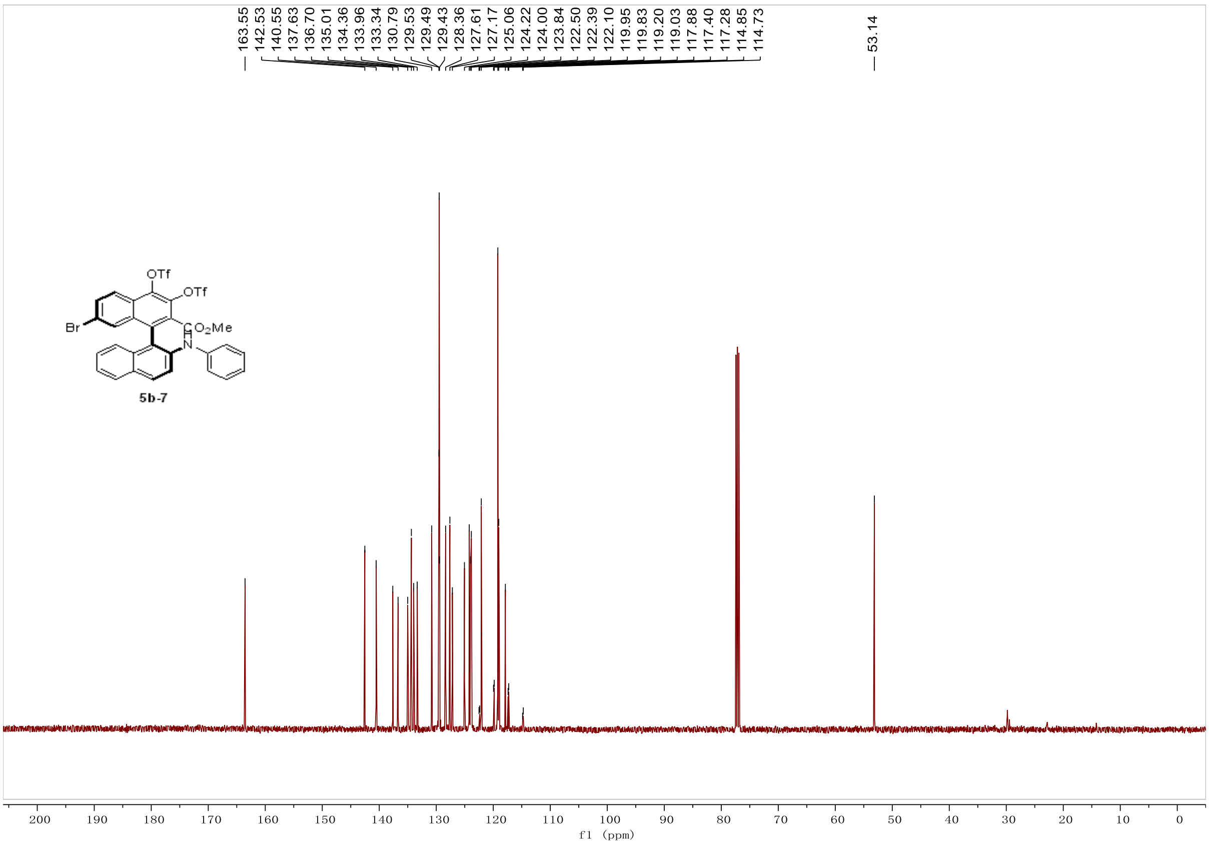

Supplementary Figure 289. ^{19}F NMR of **5b-7**.

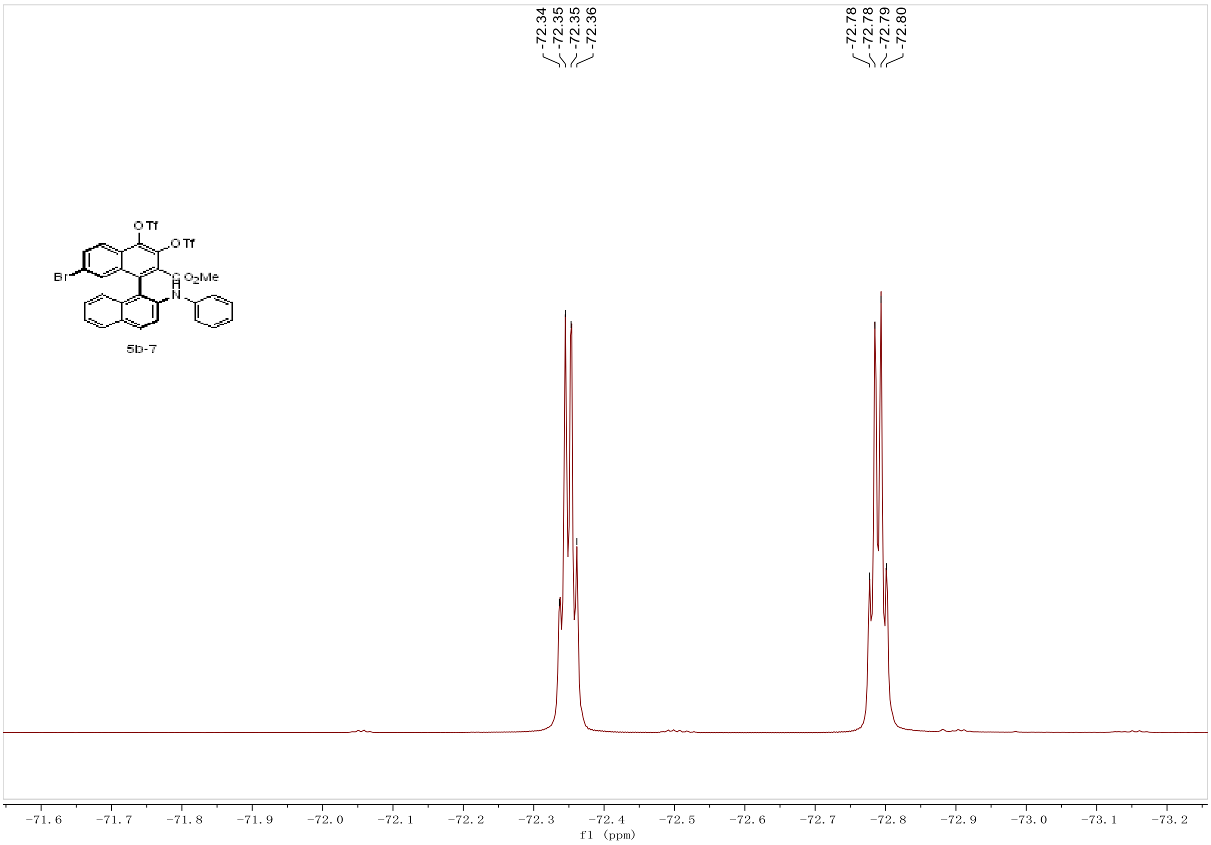

Supplementary Figure 290. ^{1}H NMR of **5b-12**.

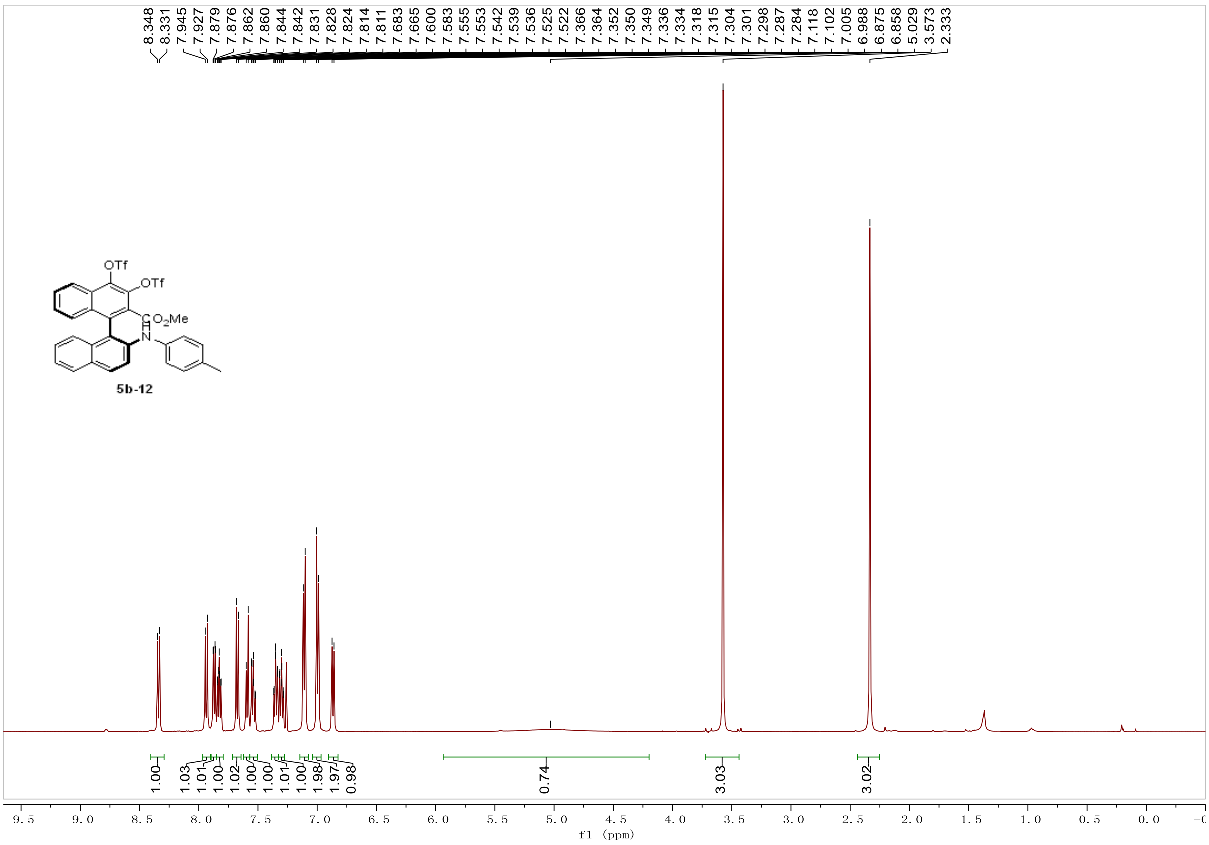

Supplementary Figure 291. ^{13}C NMR of **5b-12**.

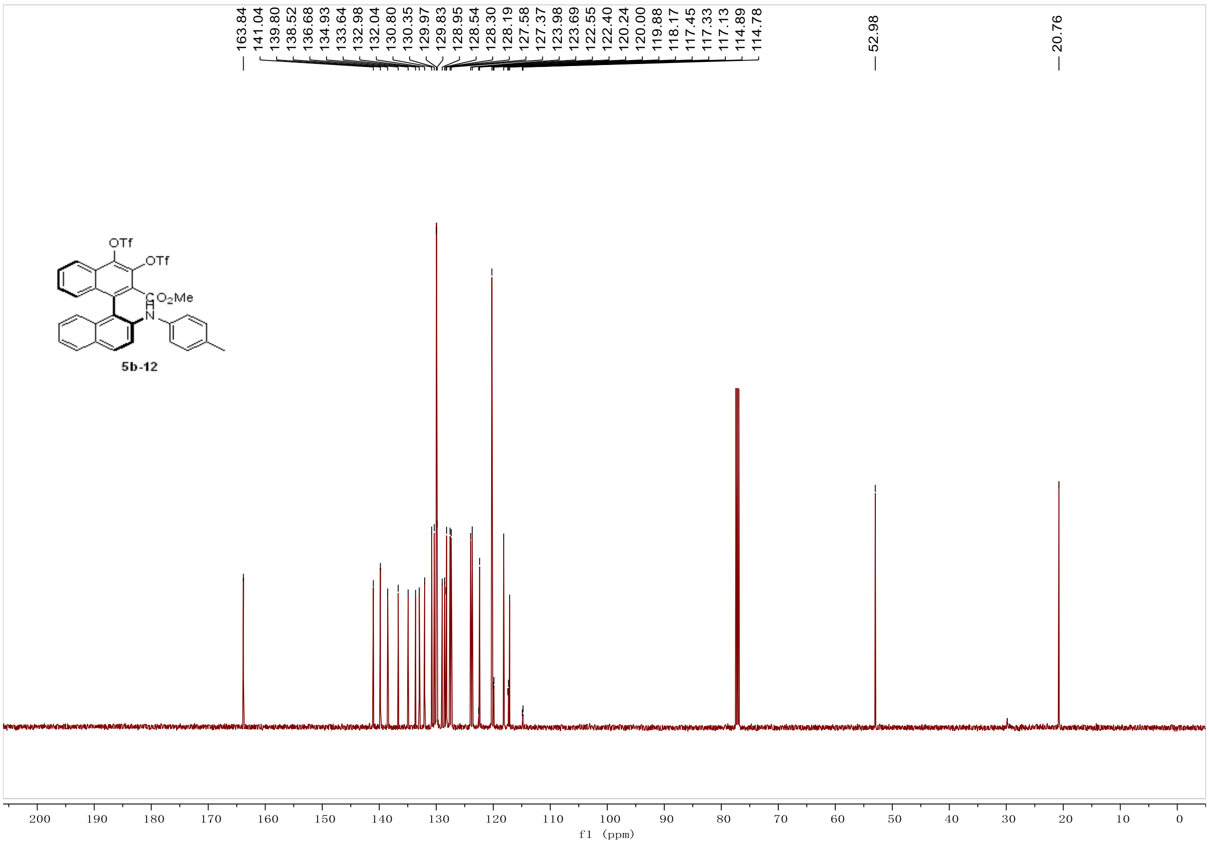

Supplementary Figure 292. ^{19}F NMR of **5b-12**.

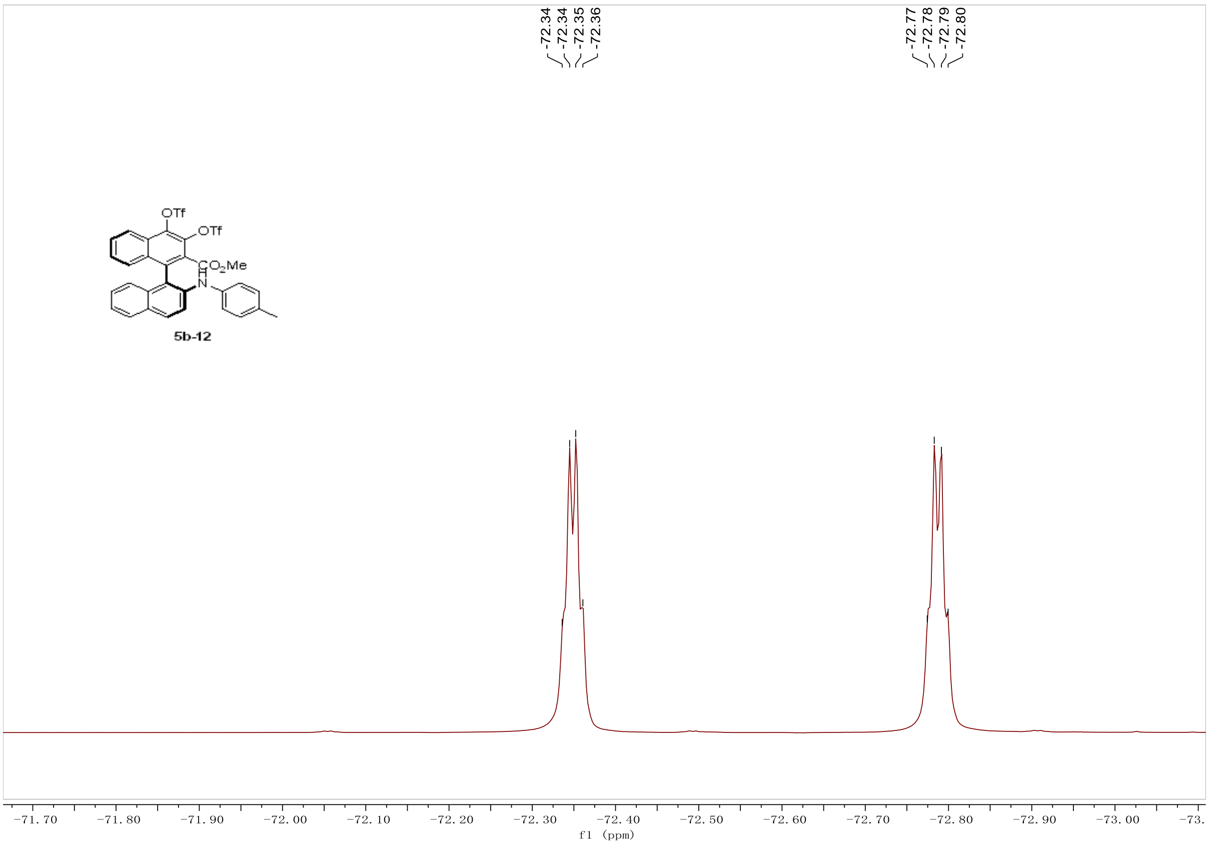

Supplementary Figure 293. ^{1}H NMR of **5b-14**.

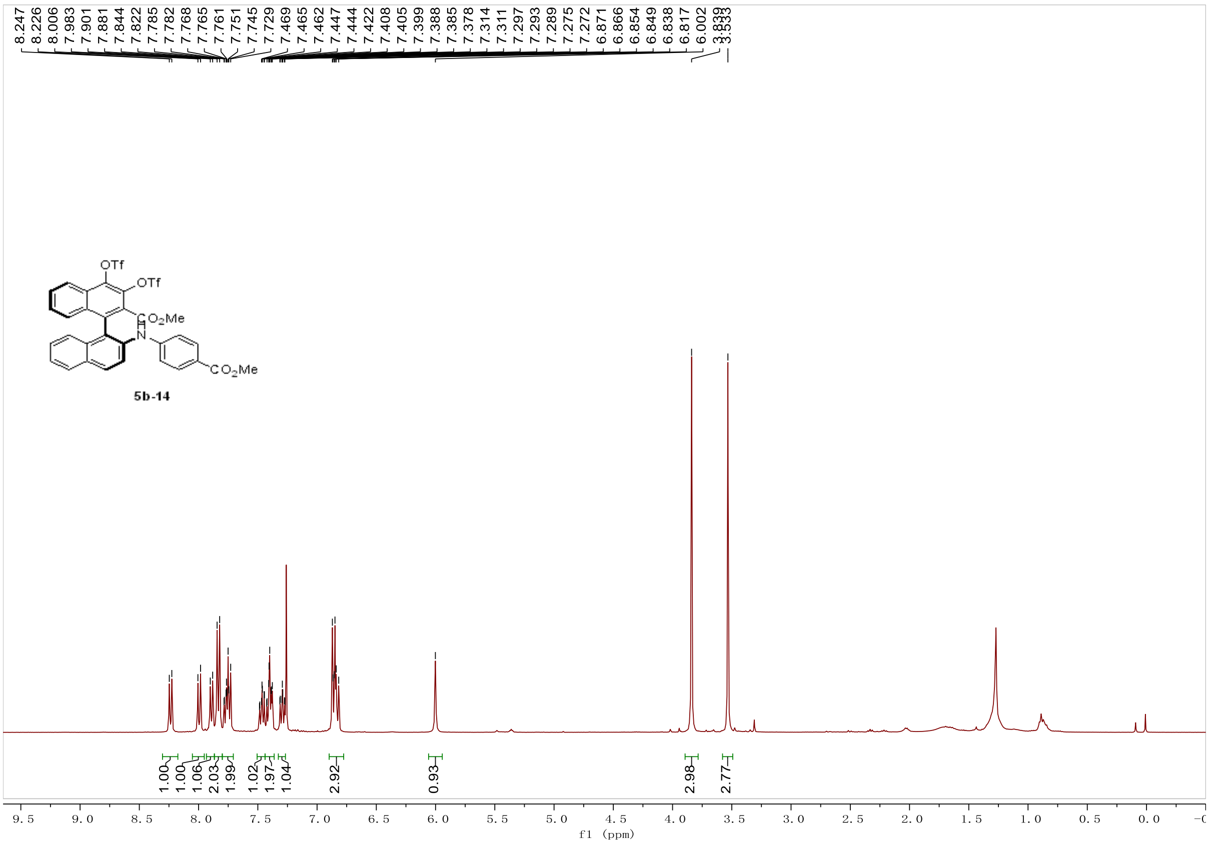

Supplementary Figure 294. ^{13}C NMR of **5b-14**.

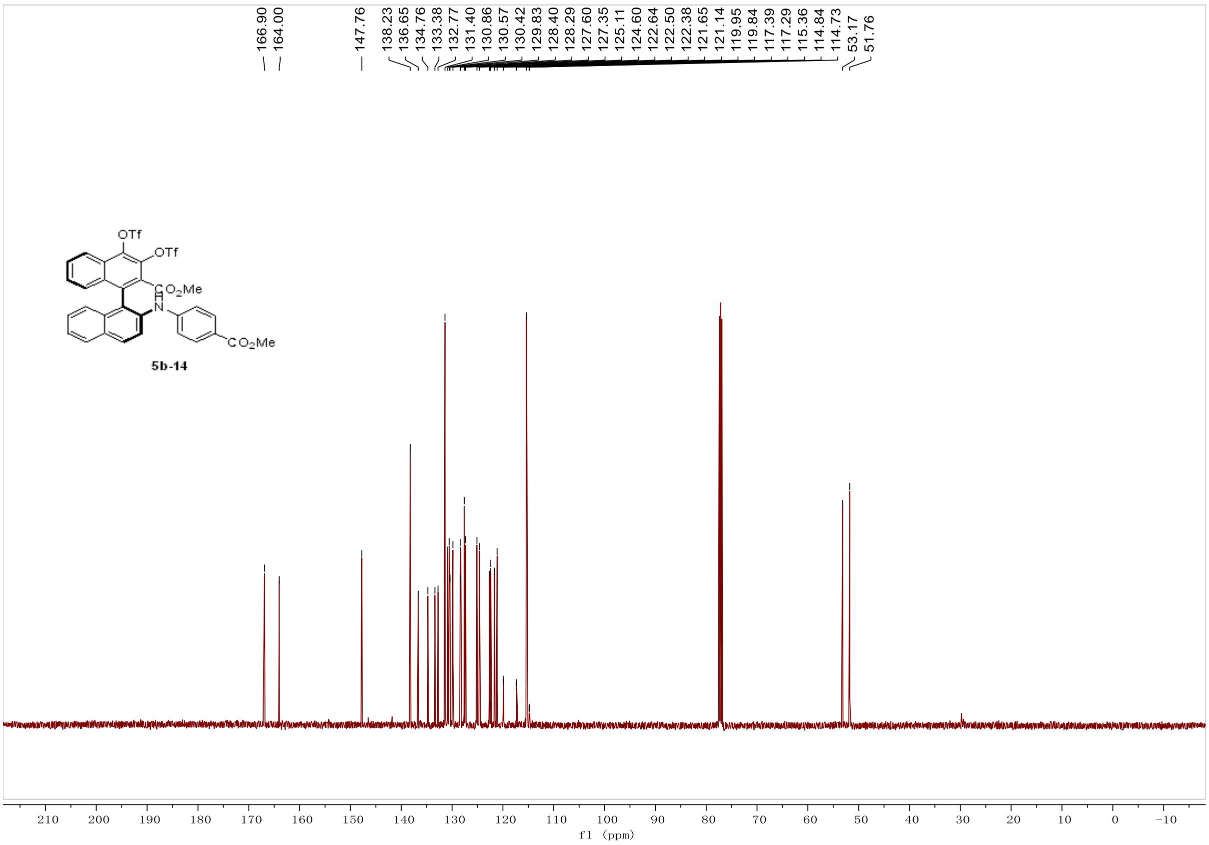

Supplementary Figure 295. ^{19}F NMR of **5b-14**.

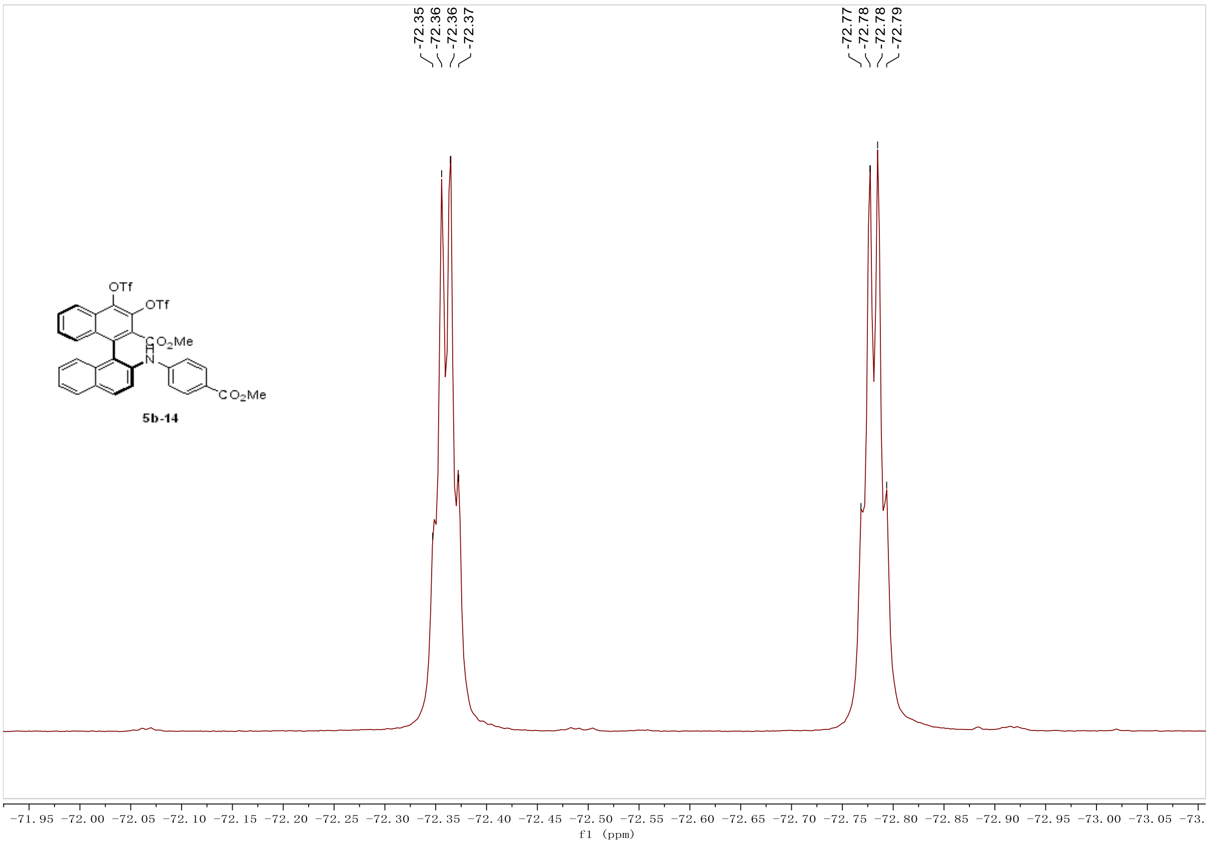

Supplementary Figure 296. ^{1}H NMR of **6**.

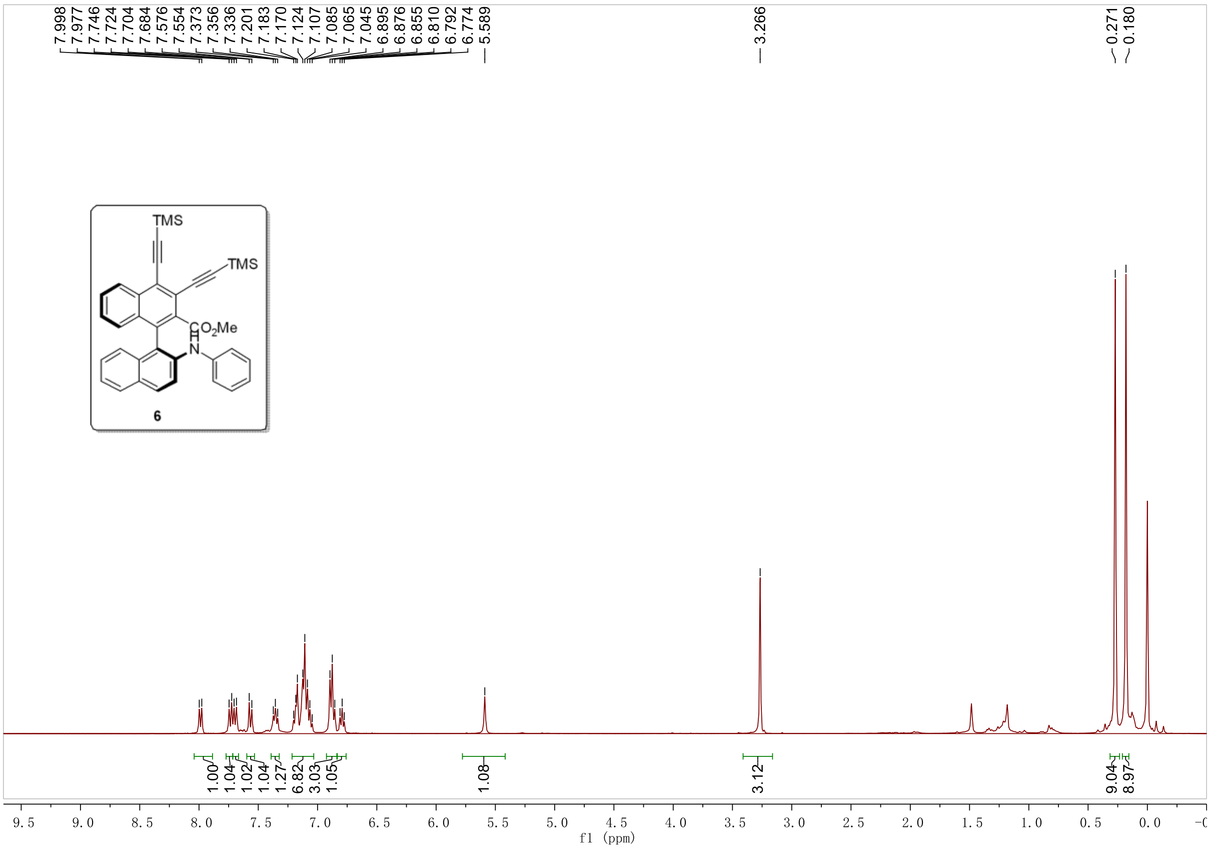

Supplementary Figure 297. ^{13}C NMR of **6**.

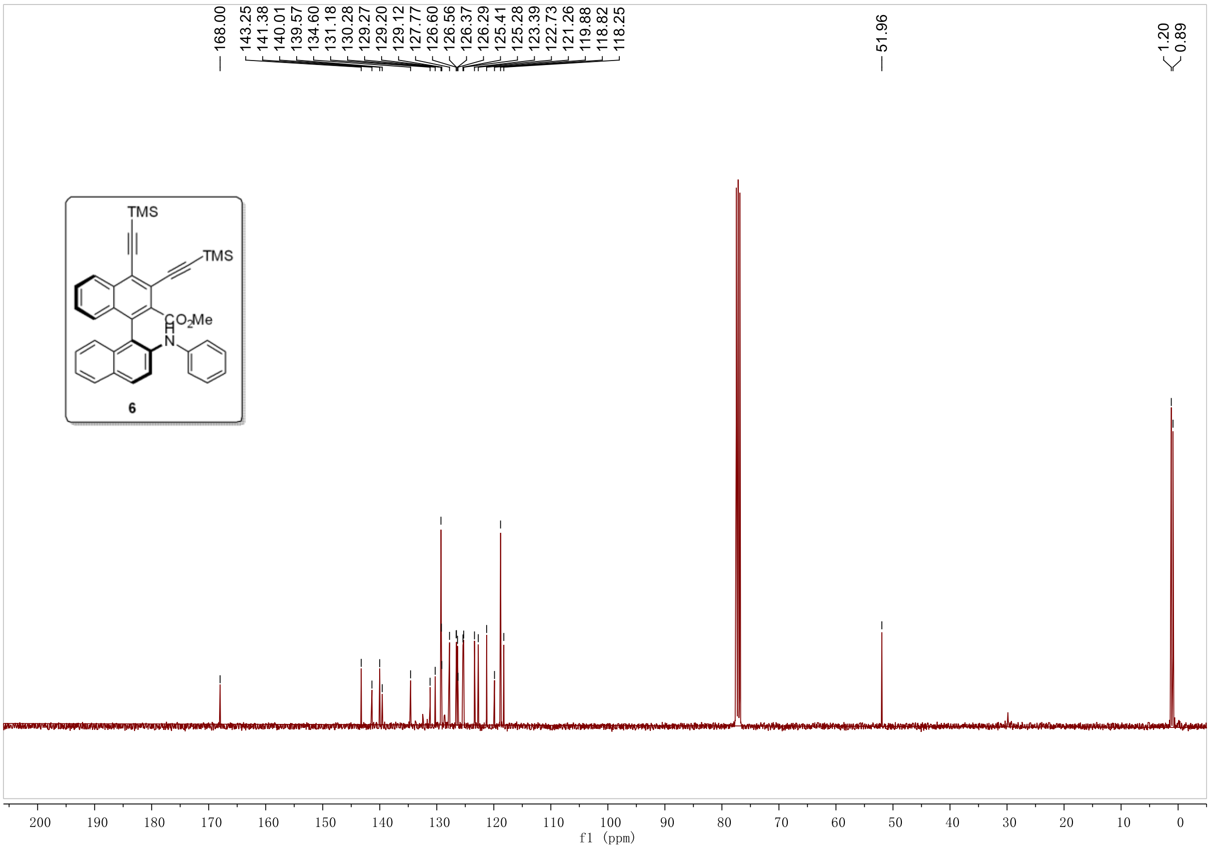

Supplementary Figure 298. ^{1}H NMR of **7**.

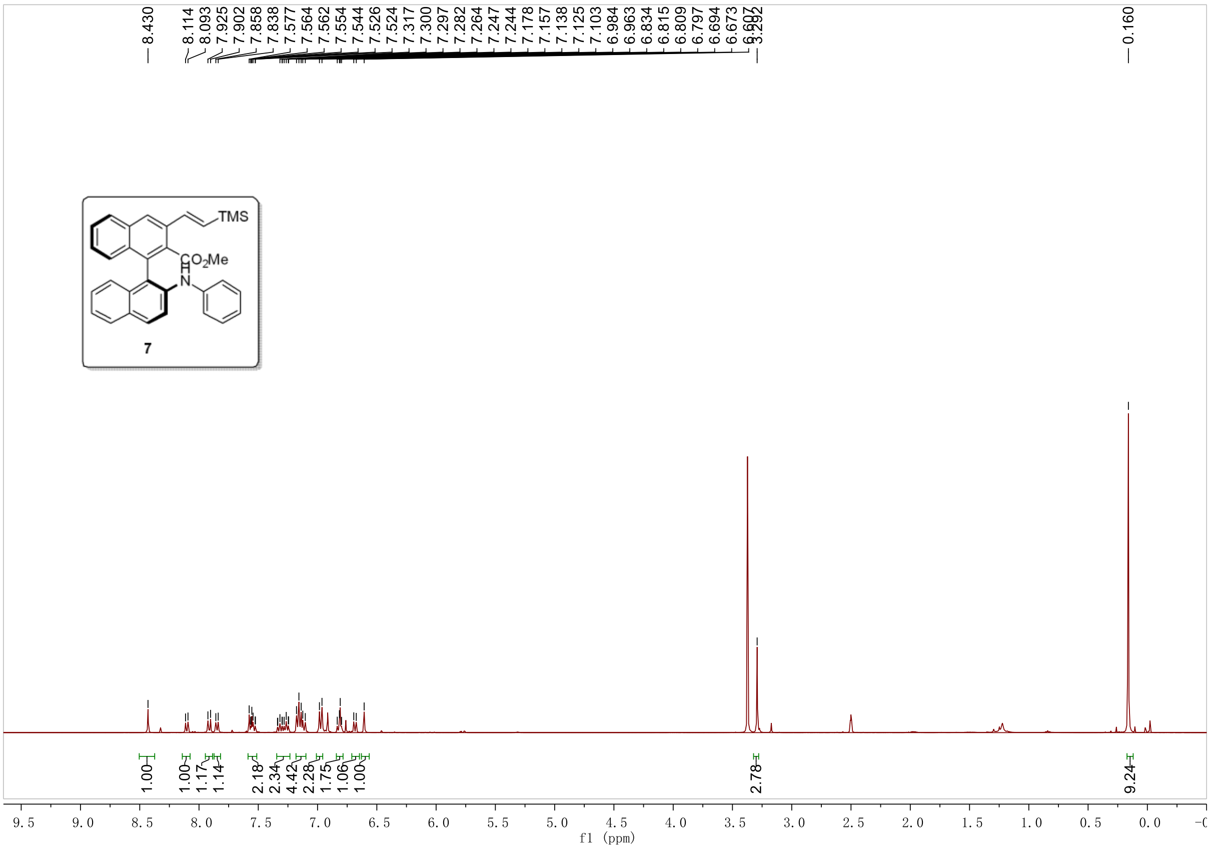

Supplementary Figure 299. ^{13}C NMR of **7**.

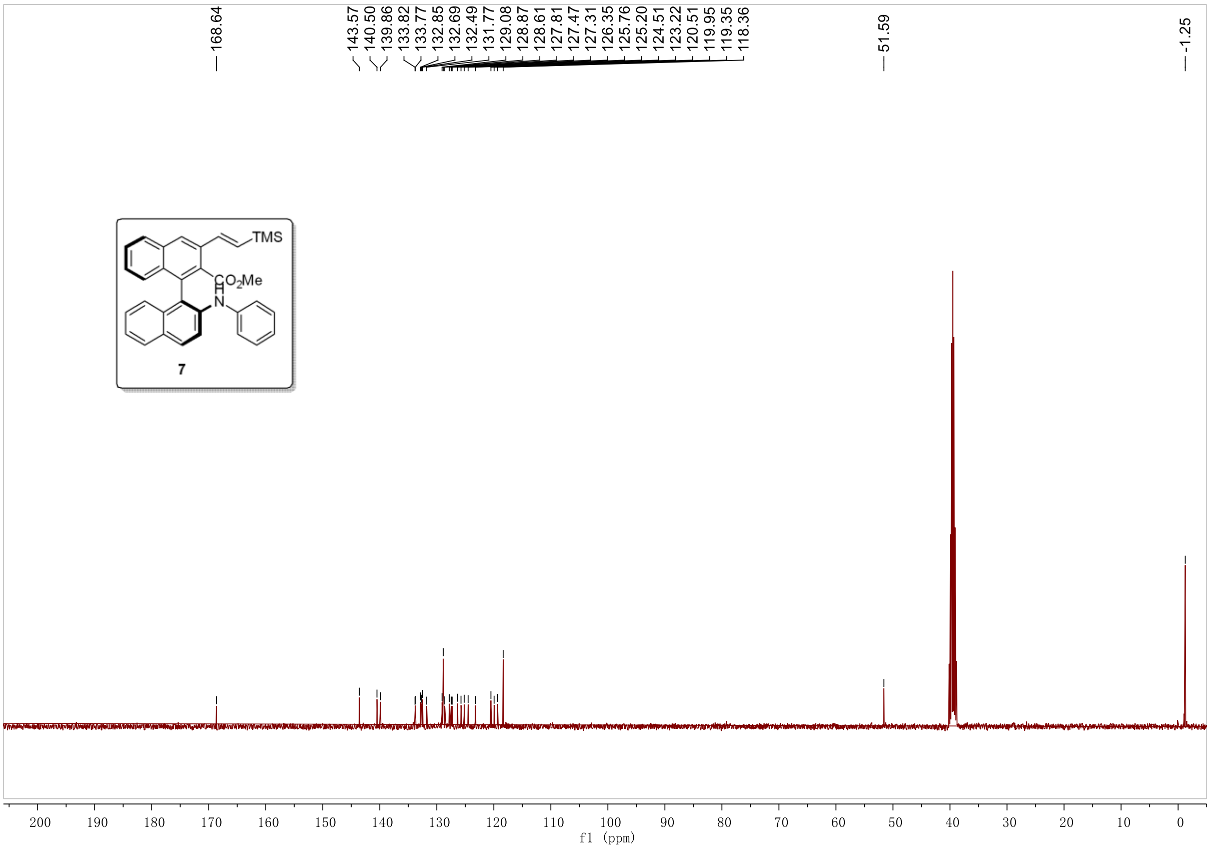

Supplementary Figure 300. ^{1}H NMR of **8a**.

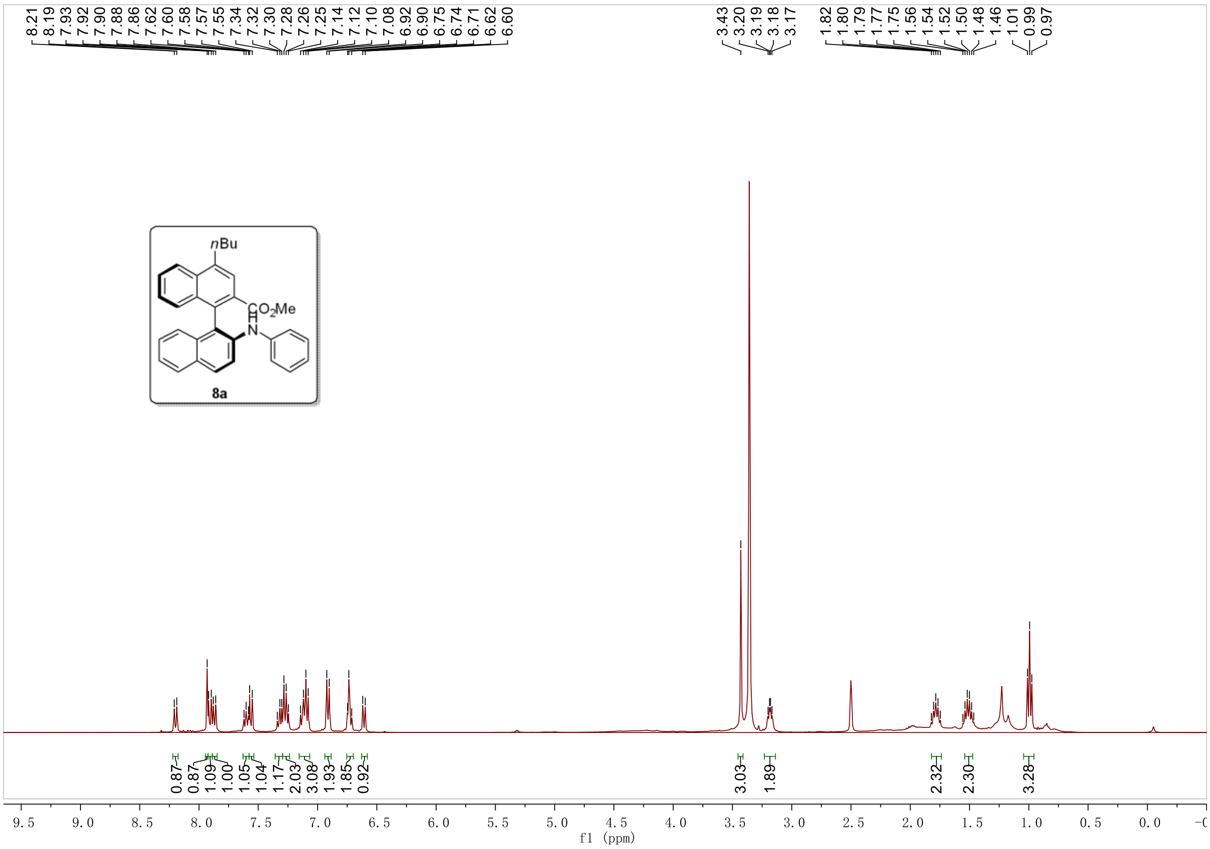

Supplementary Figure 301. ^{13}C NMR of **8a**.

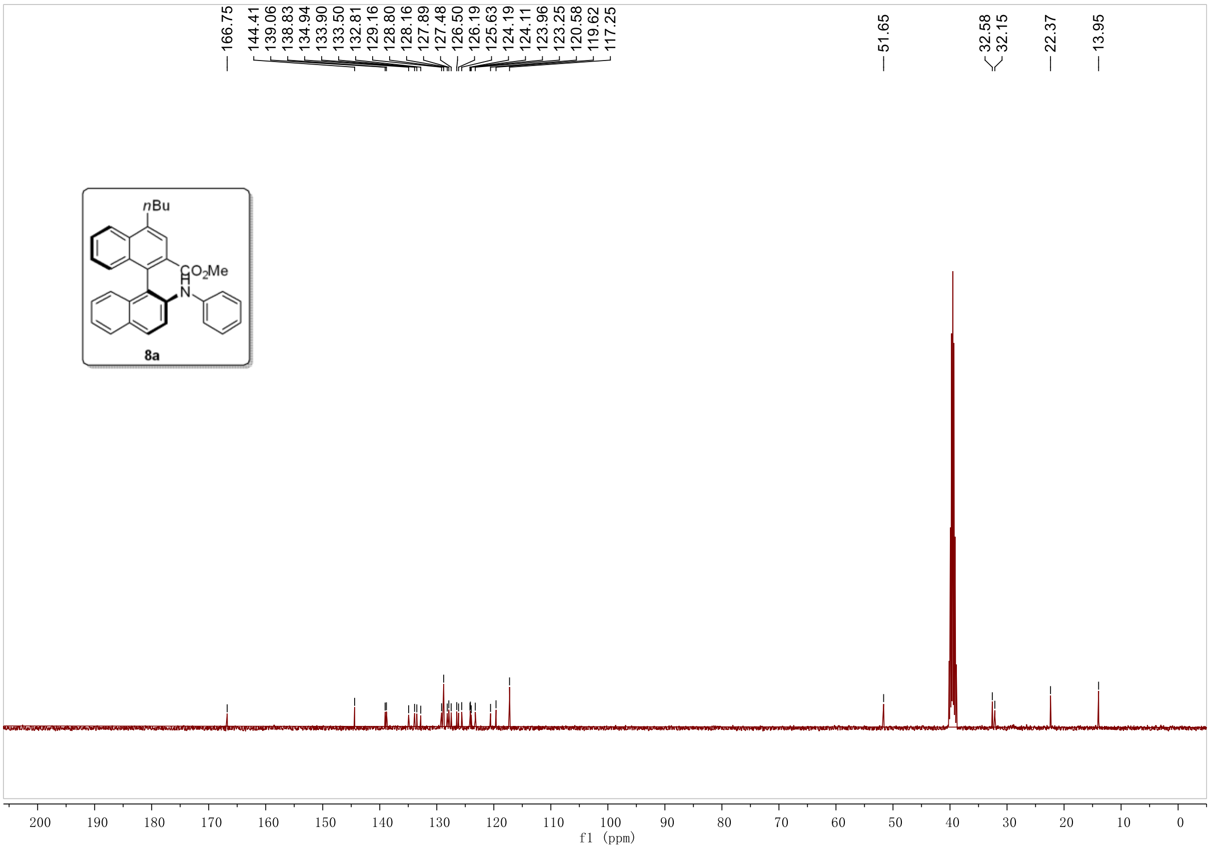

Supplementary Figure 302. ^{1}H NMR of **8b**.

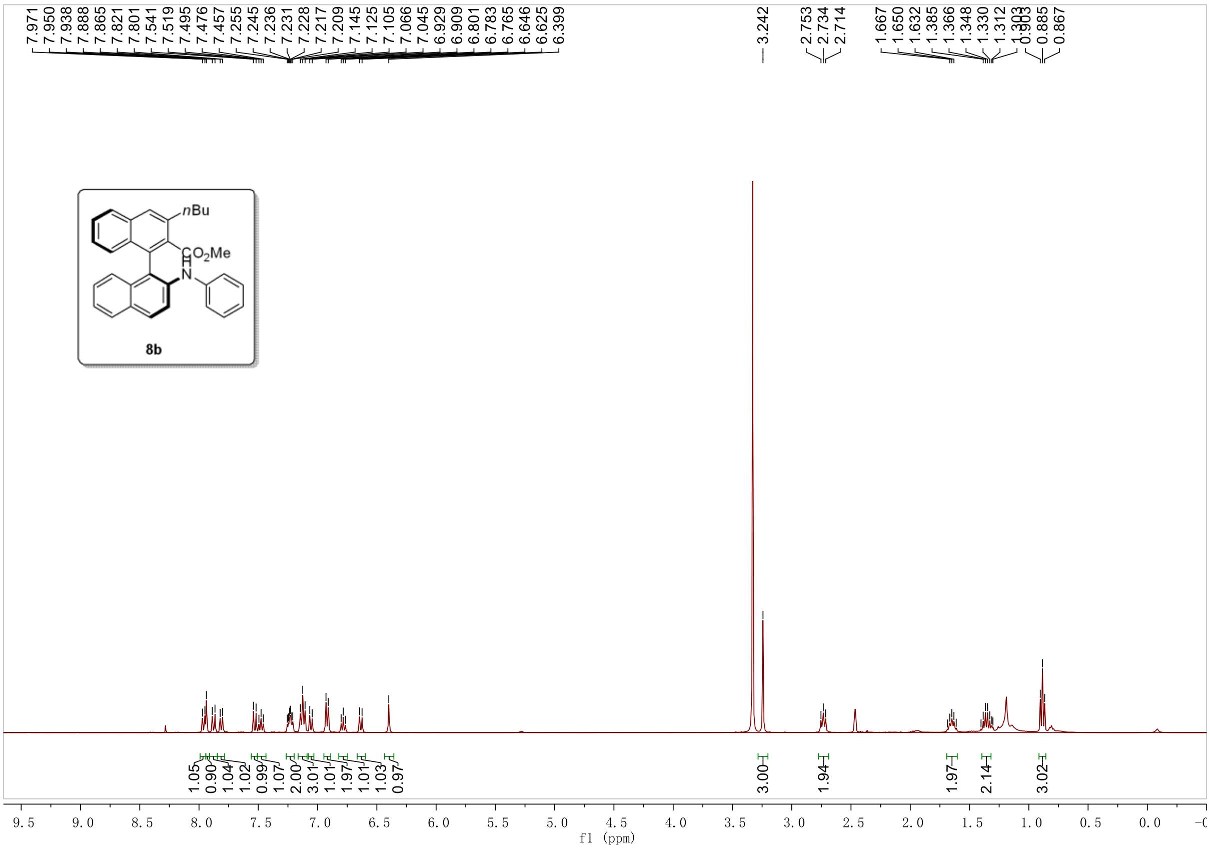

Supplementary Figure 303. ^{13}C NMR of **8b**.

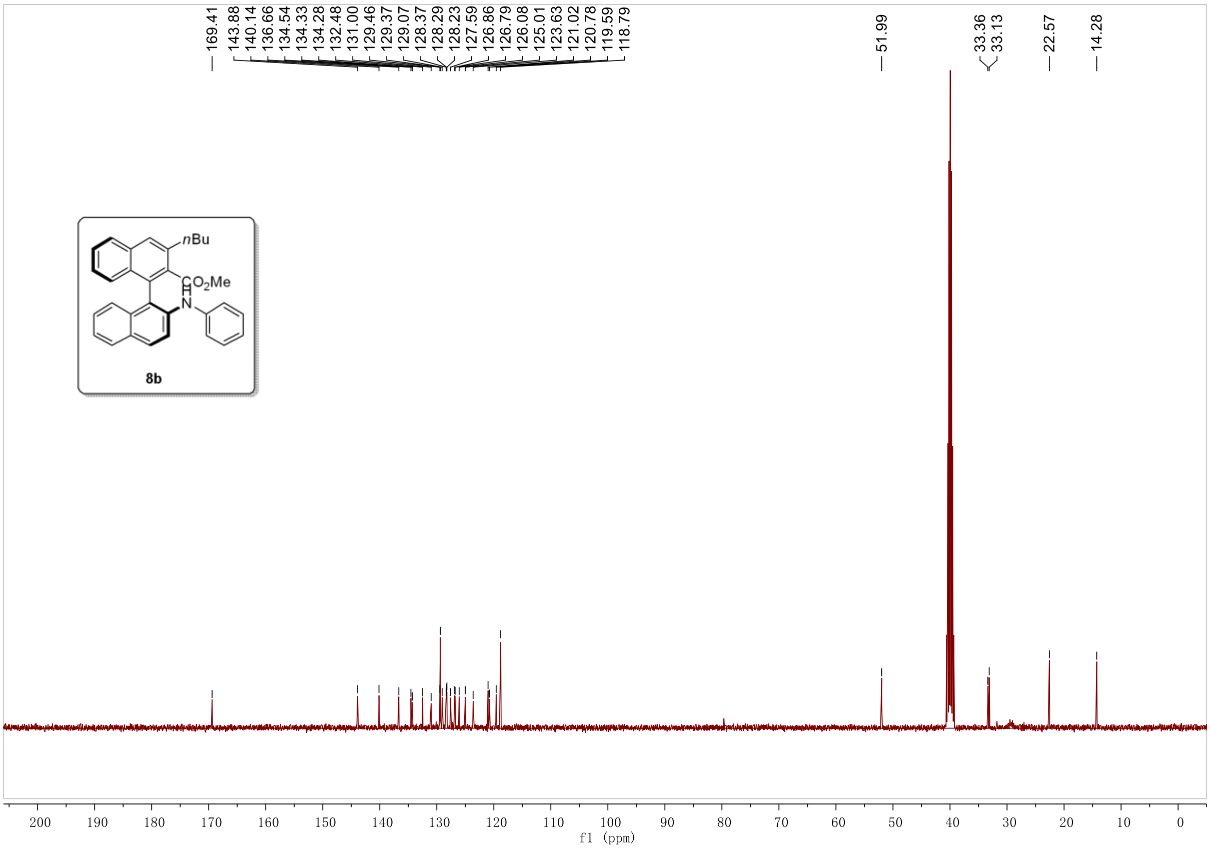

Supplementary Figure 304. ^{1}H NMR of **9**.

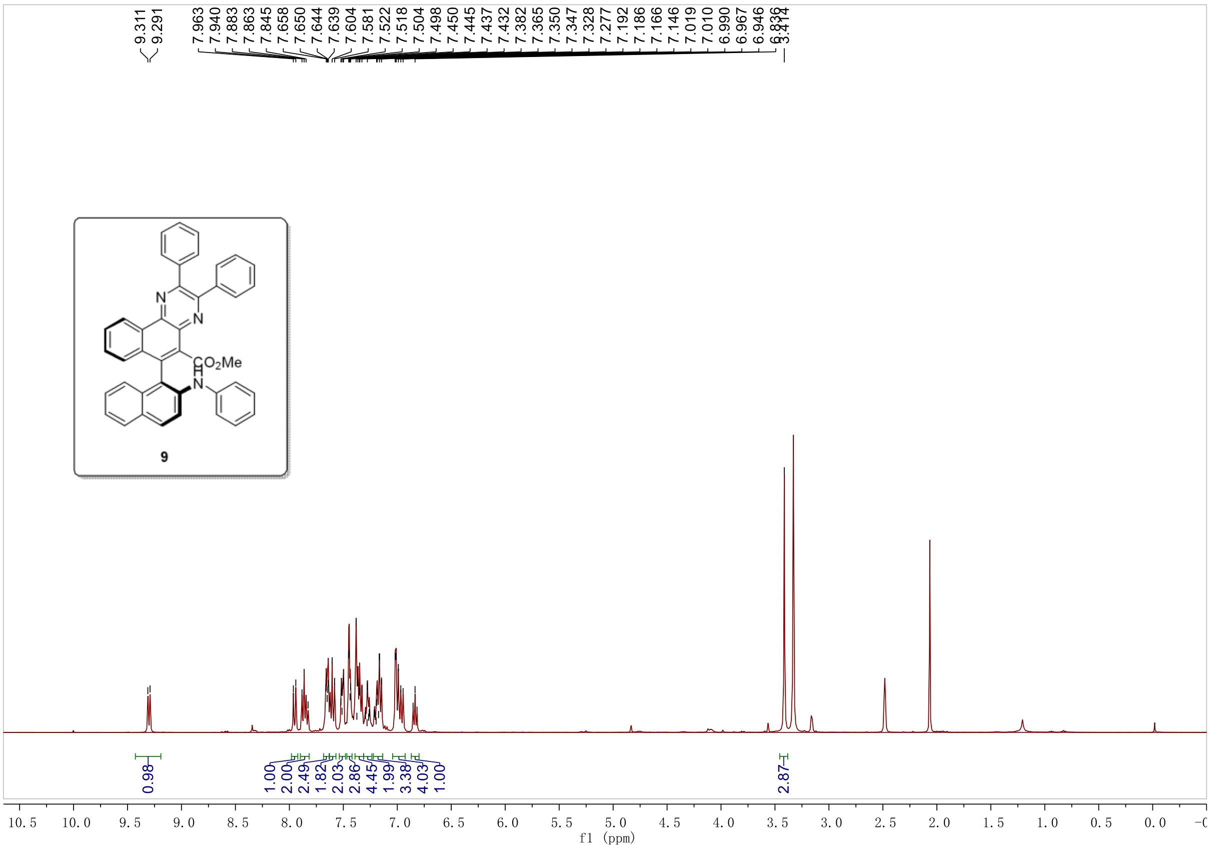

Supplementary Figure 305. ^{13}C NMR of **9**.

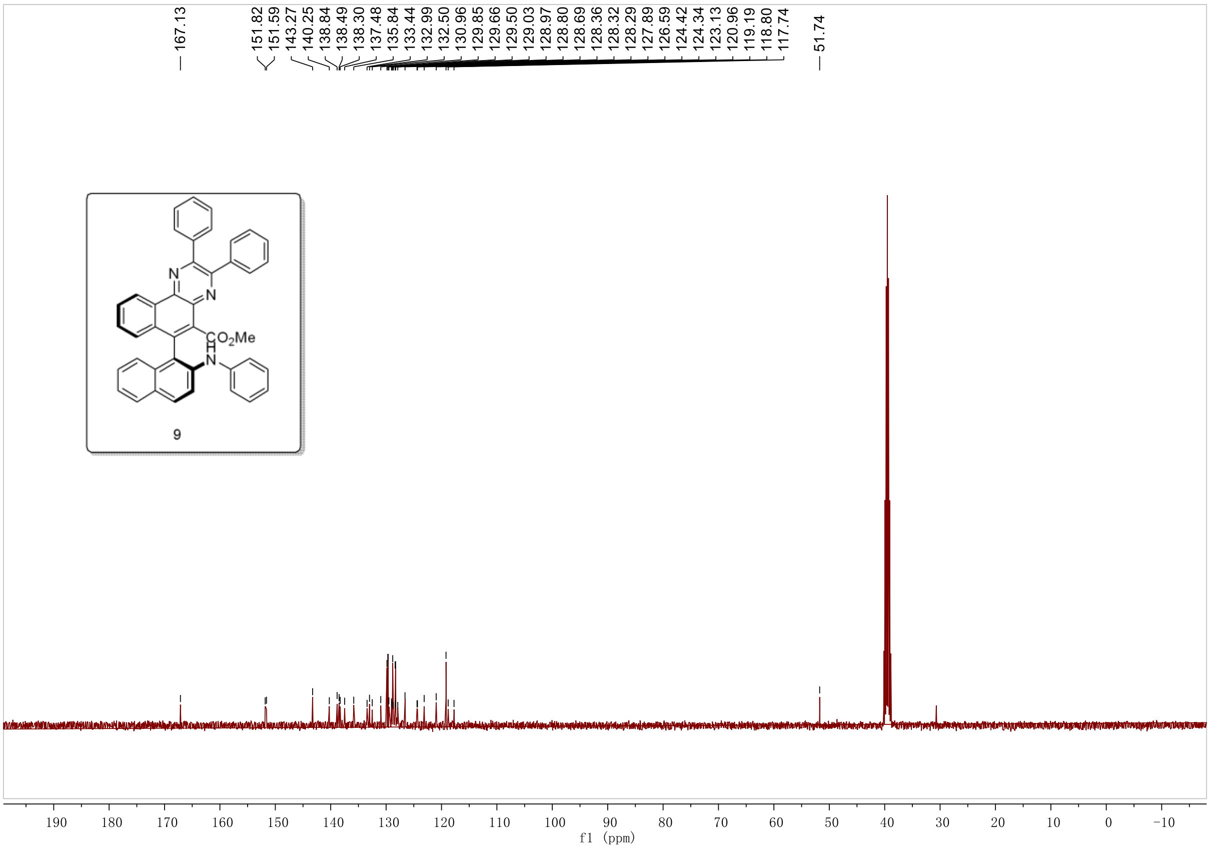

Supplementary Figure 306. ^{1}H NMR of **10**.

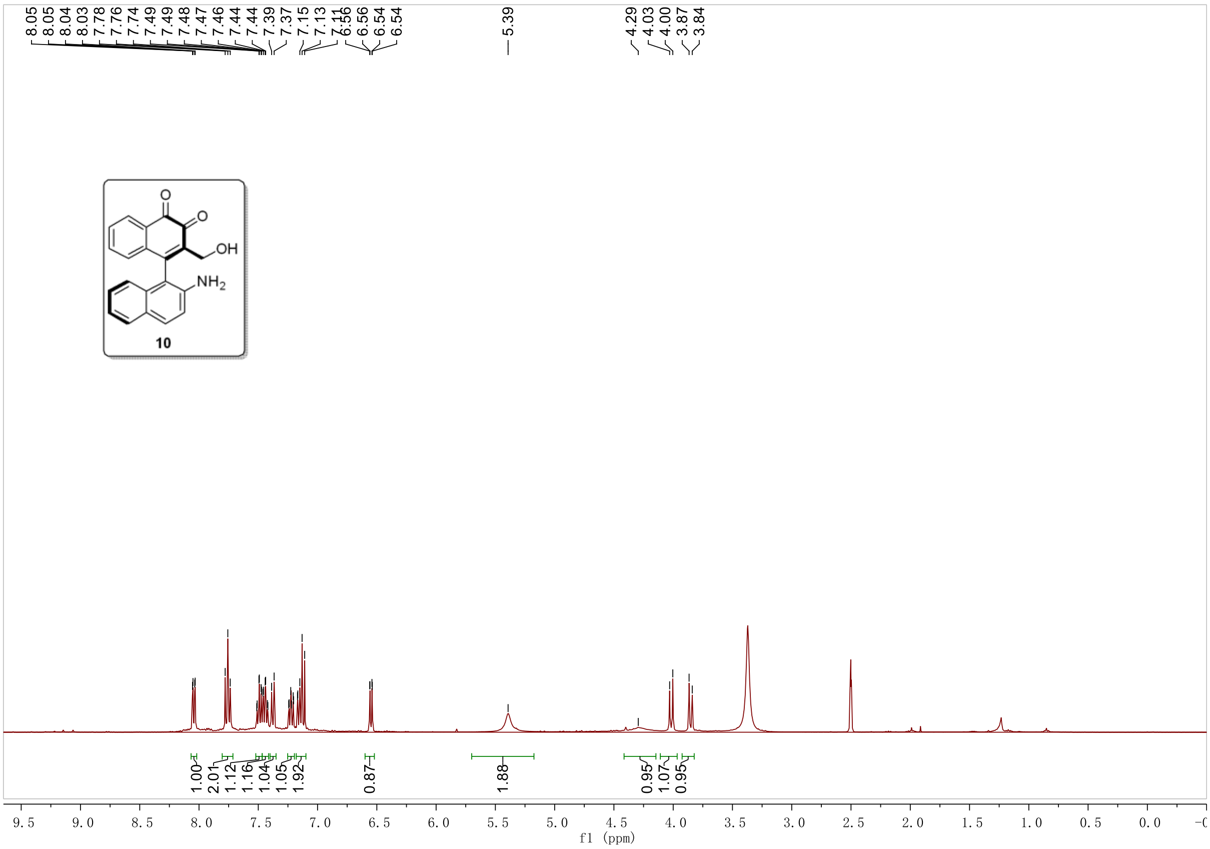

Supplementary Figure 307. ^{13}C NMR of **10**.

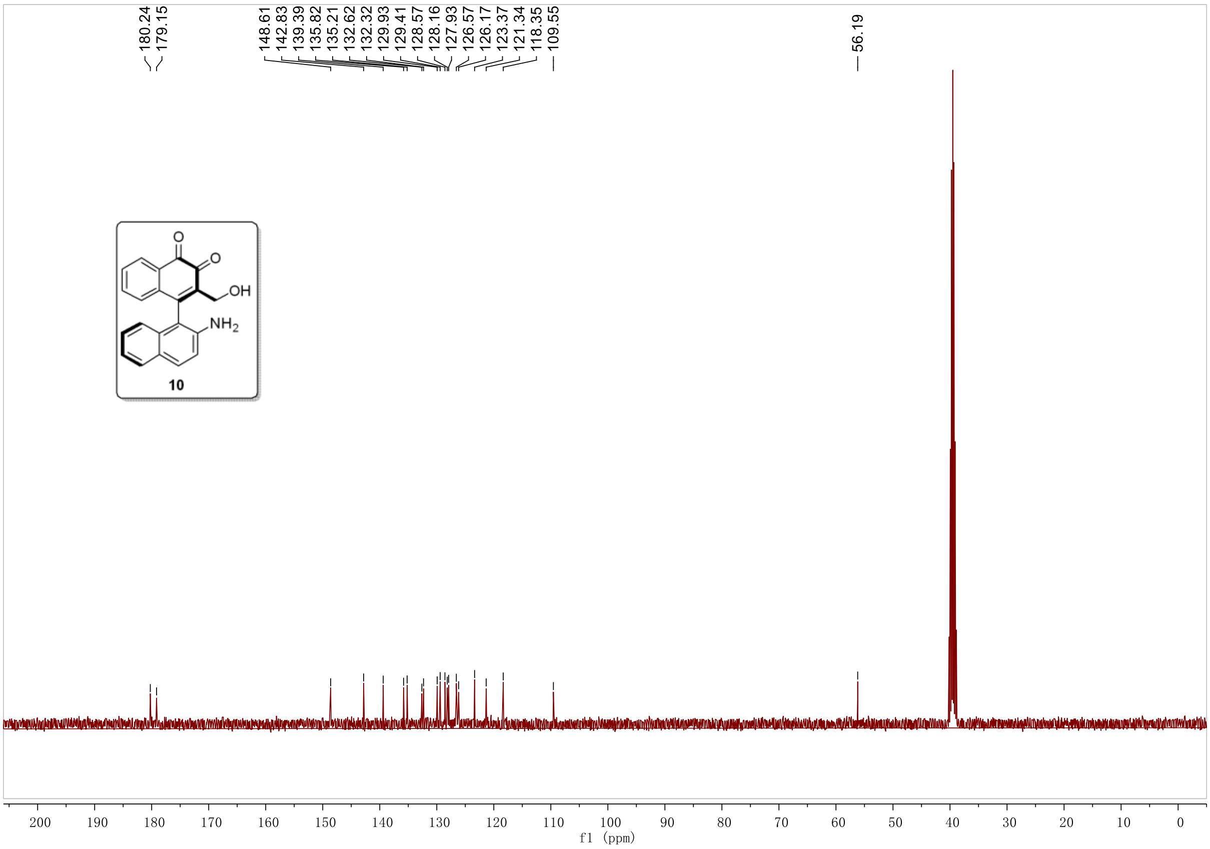

Supplementary Figure 308. ^{1}H NMR of **11**.

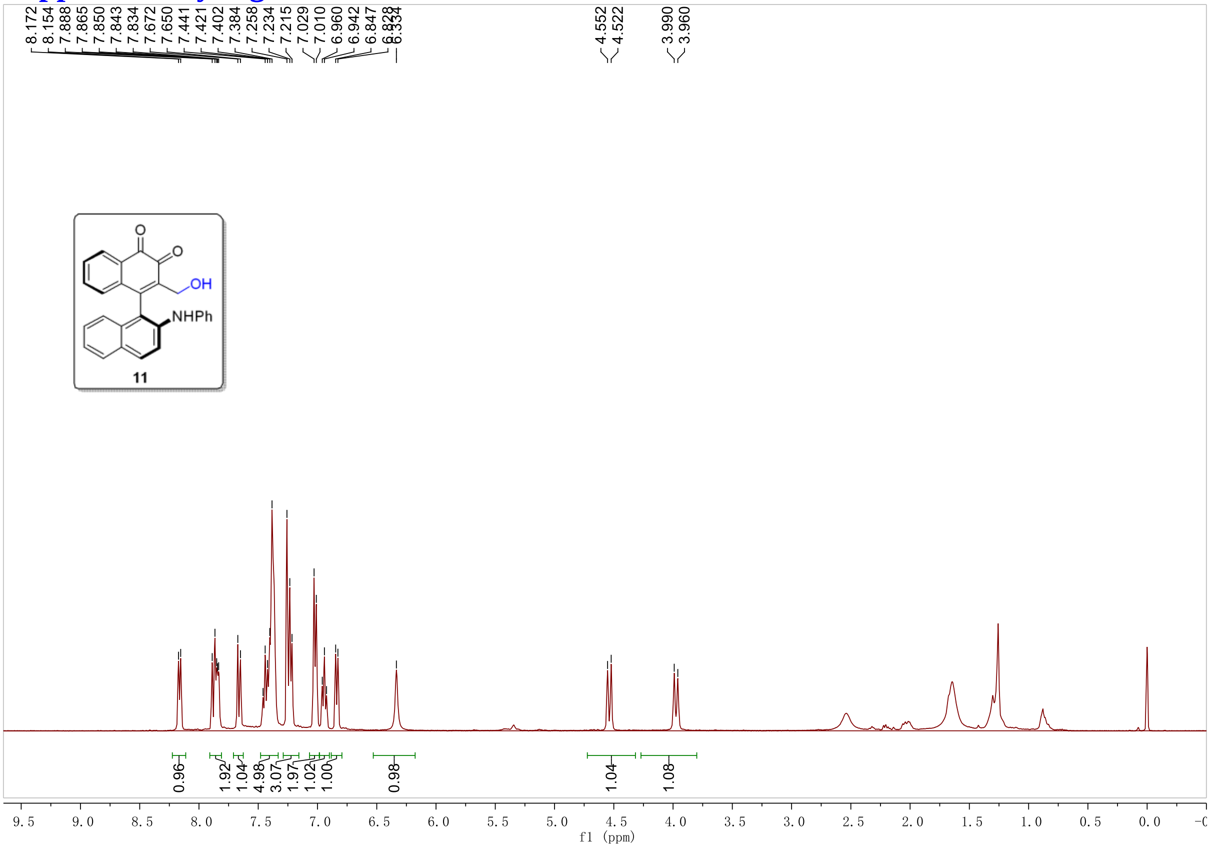

Supplementary Figure 309. ^{13}C NMR of **11**.

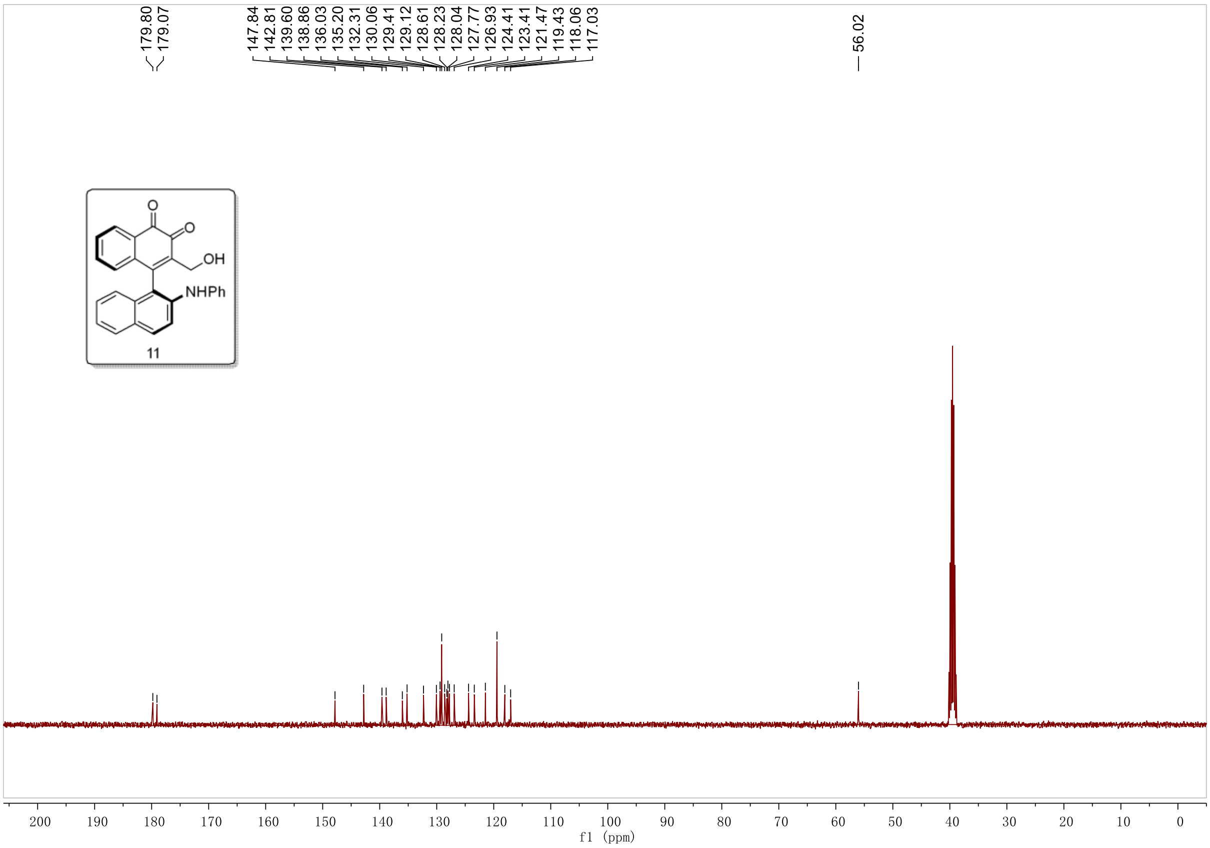

Supplementary Figure 310. ^{1}H NMR of **12**.

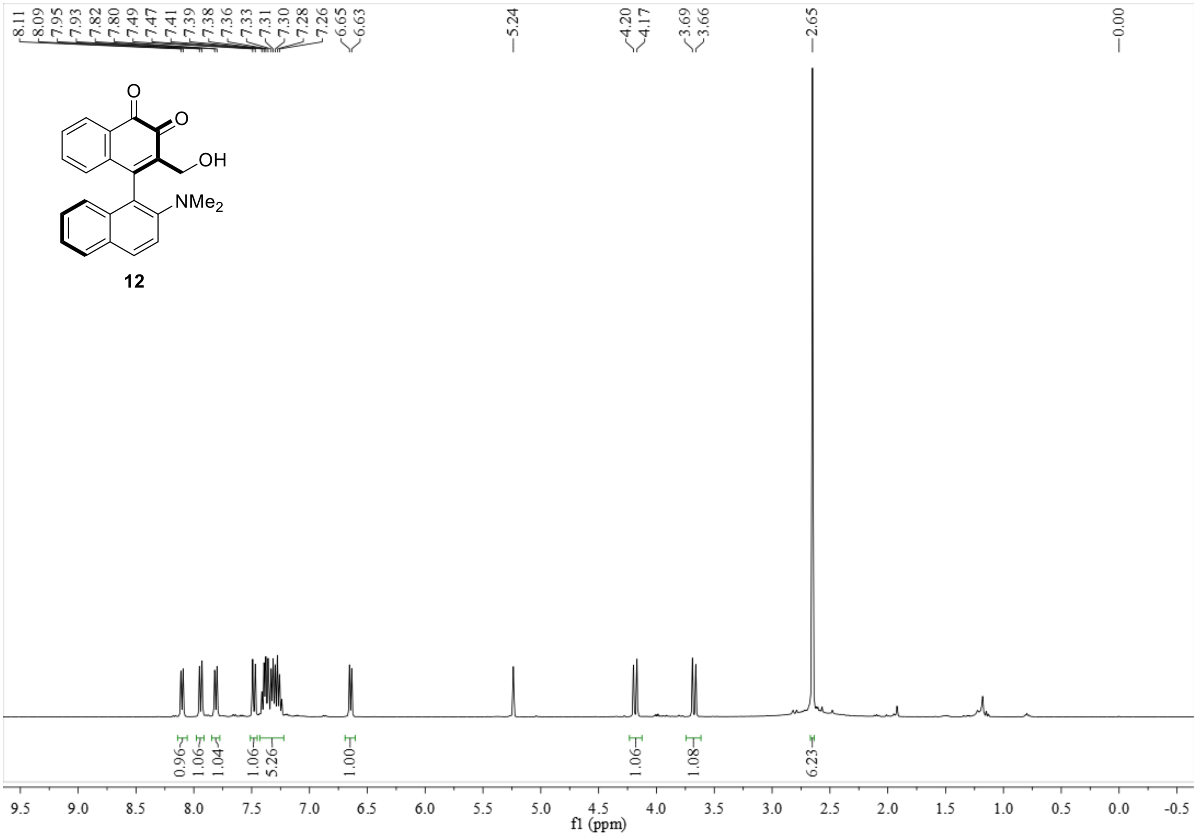

Supplementary Figure 311. ^{13}C NMR of **12**

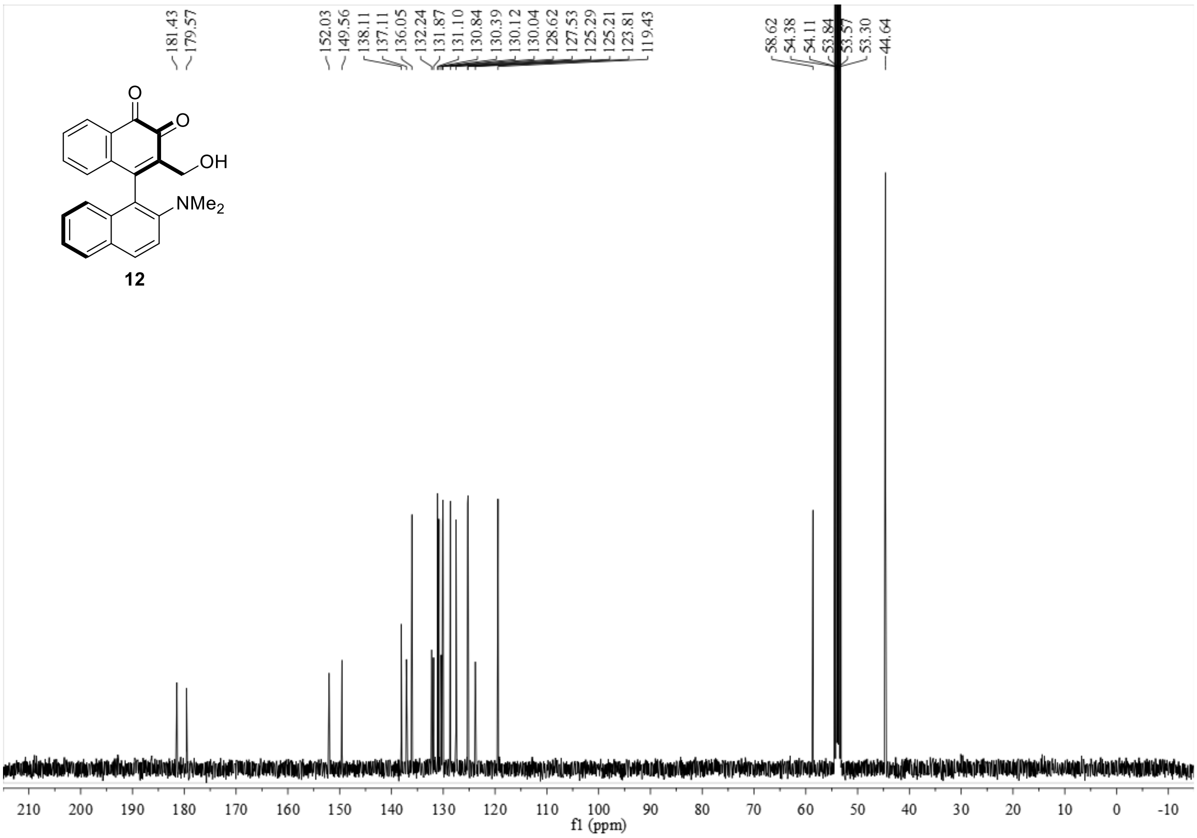

Supplementary Figure 312. ^{1}H NMR of **13**.

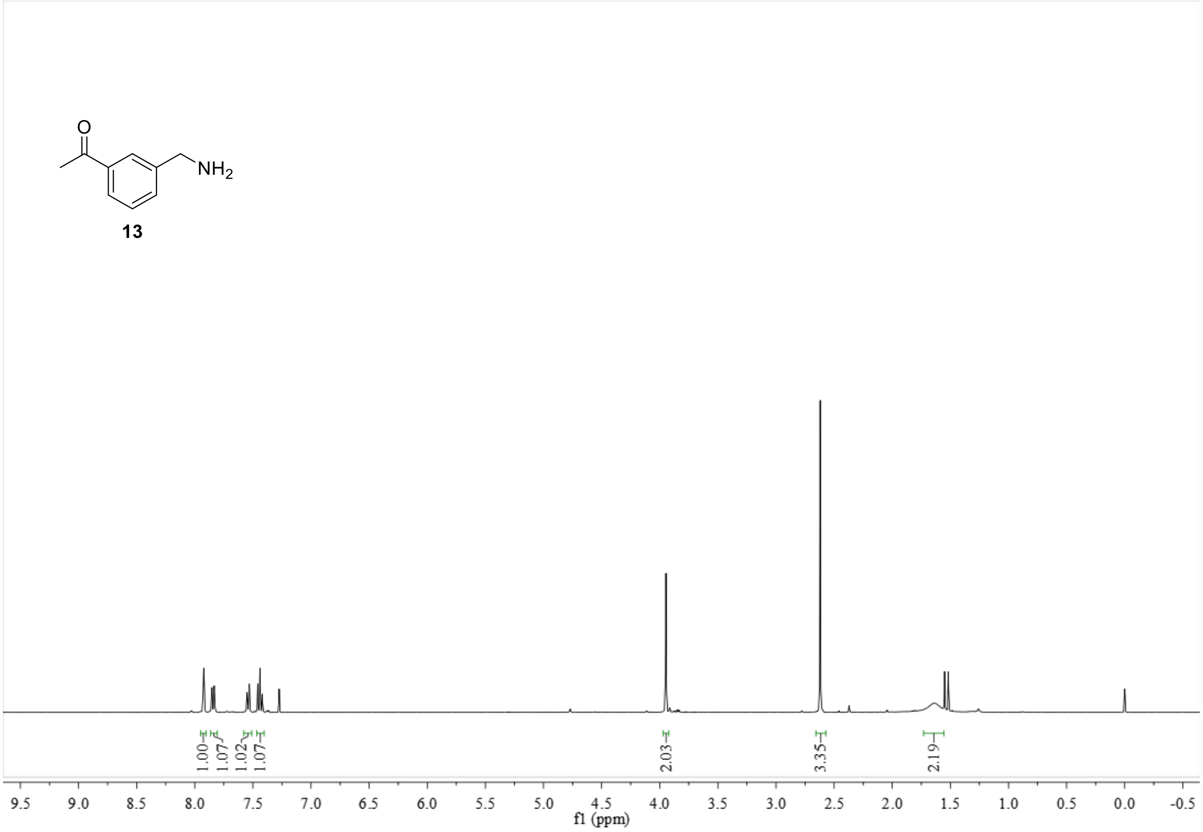

Supplementary Figure 313. ^{13}C NMR of **13**.

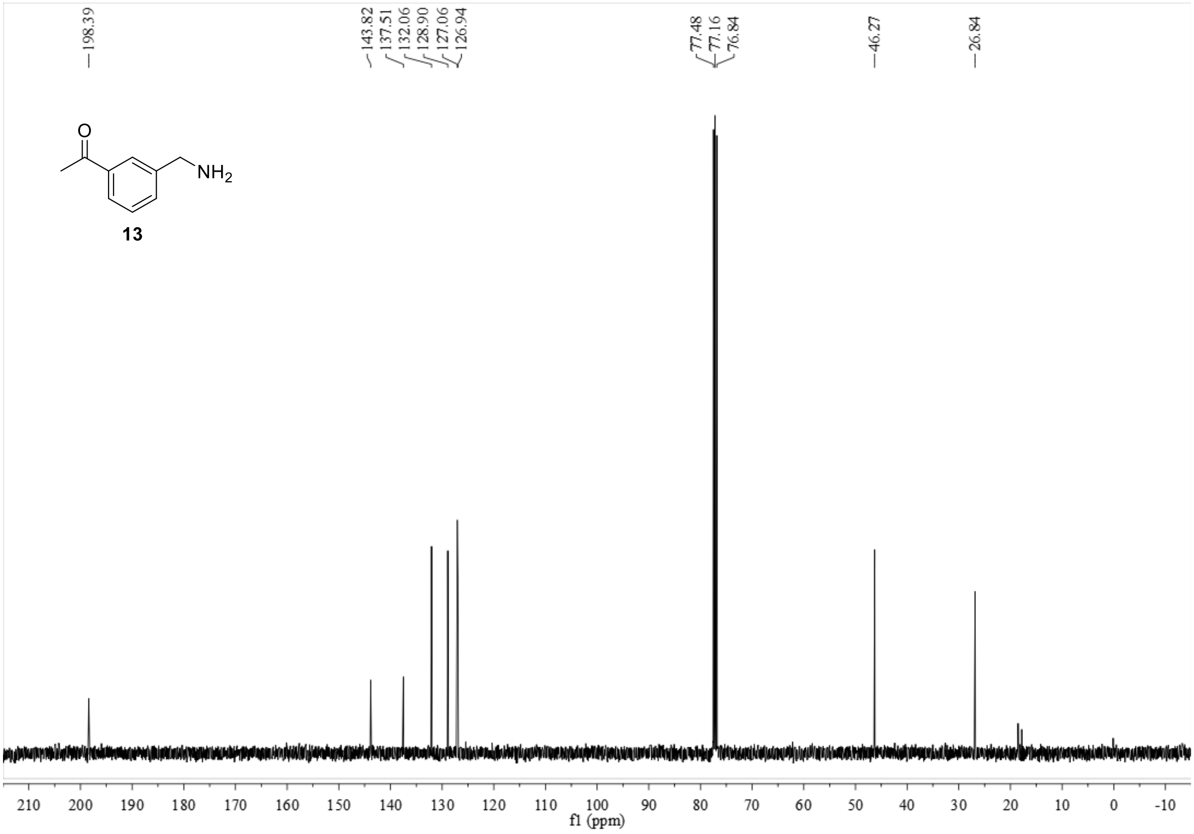

Supplementary Figure 314. ^{1}H NMR of **14**.

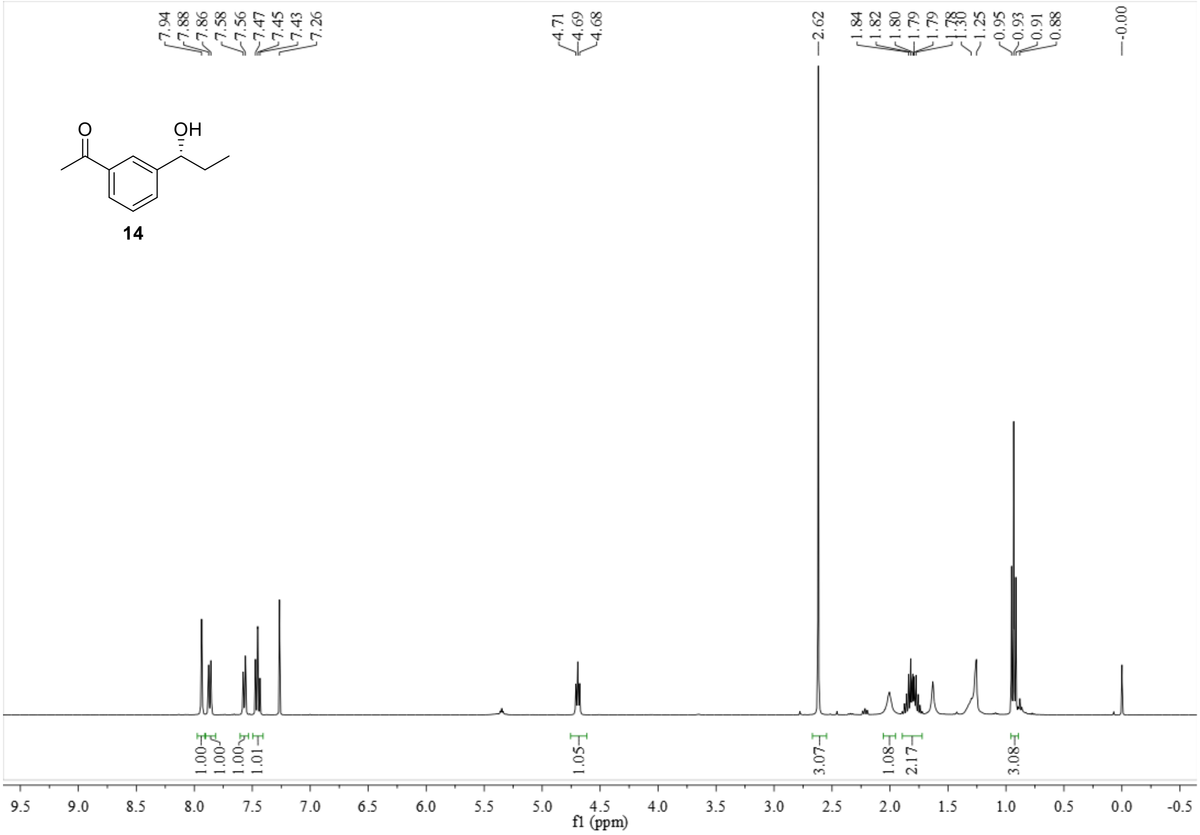

Supplementary Figure 315. ^{13}C NMR of **14**.

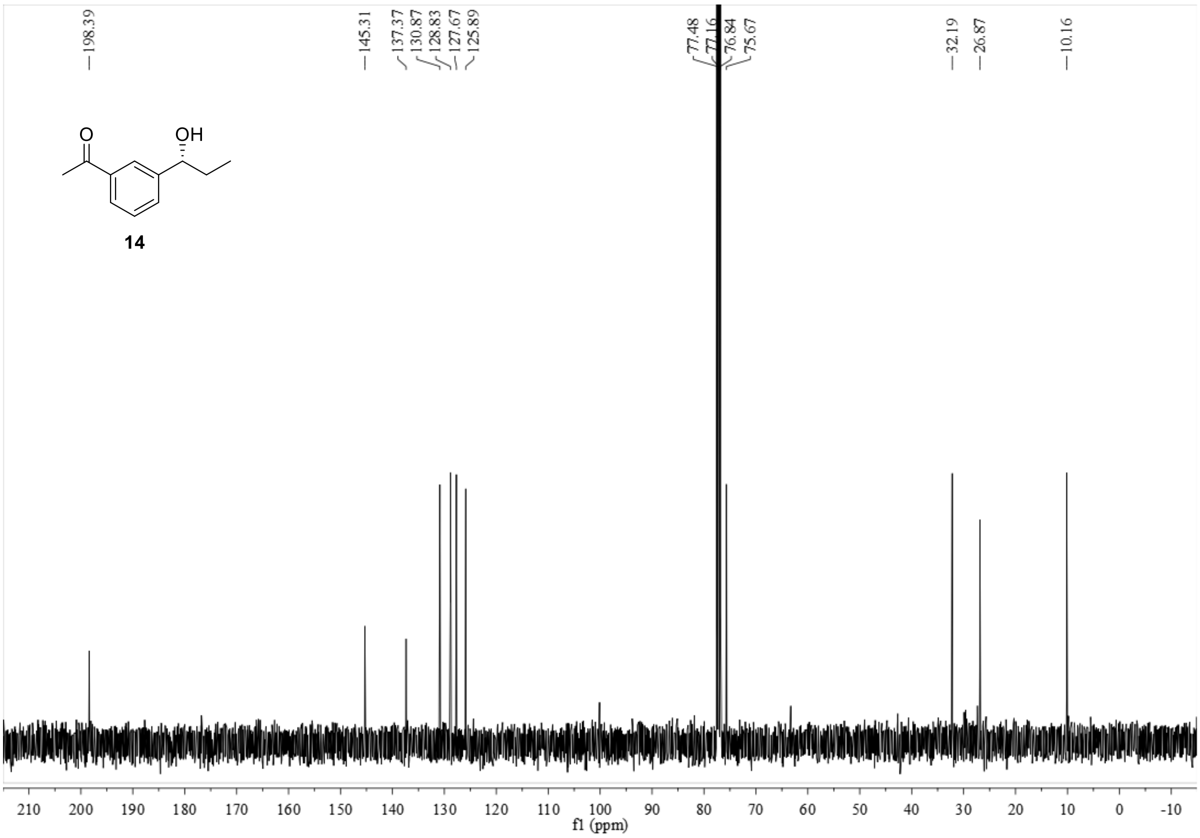

Supplementary References

1. Chen, Y.-H., Qi, L-W., Fang, F. & Tan, B. Organocatalytic atroposelective arylation of 2-naphthylamines as a practical approach to axially chiral biaryl amino alcohols. *Angew. Chem. Int. Ed.* **56**, 16308-16312 (2017).
2. Cui, H.-Q. *et al.* Discovery of potent and selective sirtuin 2 (SIRT2) inhibitors using a fragment-based approach. *J. Med. Chem.* **57**, 8340-8357 (2014).
3. Qi, L.-W., Mao, J.-H., Zhang, J. & Tan, B. Organocatalytic asymmetric arylation of indoles enabled by azo groups. *Nat. Chem.* **10**, 58-64 (2018).
